# Supplementary material for: Silver-catalyzed direct conversion of epoxides into cyclopropanes using N-triftosylhydrazones
Source: Nat Commun. 2024 Mar 2;15:1951. doi: 10.1038/s41467-024-46188-w (PMC10908805; doi:10.1038/s41467-024-46188-w)
Supplement: Supplementary file 1 — Supplementary Information [file 41467_2024_46188_MOESM1_ESM.pdf]

Supplementary Information for

Silver-catalyzed direct conversion of epoxides into cyclopropanes using *N*-triftosylhydrazones

Linxuan Li[1], Paramasivam Sivaguru[1], Dandan Wei[1], Menglin Liu[1], Qingwen Zhu[1], Shuai Dong[1], Emanuele Casali[2], Nan Li[1], Giuseppe Zanoni[2] and Xihe Bi[*1,3]

[1] Department of Chemistry, Northeast Normal University, Changchun 130024, China. [2] Department of Chemistry, University of Pavia, Pavia 27100, Italy. [3] State Key Laboratory of Elemento-Organic Chemistry, Nankai University, Tianjin 300071, China.

*Corresponding author. Email: bixh507@nenu.edu.cn

Table of Contents

1. General Information.....1

2. Experimental Procedure.....2

3. Limitations and Supplements.....18

4. Computational Details.....21

5. Characterization Data for the Products.....23

6. X-ray Crystallographic Data of Compounds **46** and **69**.....99

7. Copies of ^{1}H-, ^{13}C-, ^{19}F- and NOESY Spectra.....101

8. References.....234

1. General information

General. All reactions dealing with air- or moisture-sensitive compounds were carried out in a flame-dried, sealed Schlenk reaction tube under an atmosphere of argon. Analytical thin-layer chromatography was performed on glass plates coated with 0.25 mm 230-400 mesh silica gel containing a fluorescent indicator (Merck). Flash silica gel column chromatography was performed on silica gel 60N (spherical and neutral, 140-325 mesh) as described by Still. NMR spectra were recorded in parts per million using a Varian I NOVA 500 or Bruker AV-600 spectrometer. The ^{1}H NMR (500 MHz or 600 MHz) and ^{13}C NMR (125 MHz or 150 MHz) chemical shifts were measured relative to TMS, DMSO or $CDCl_3$ as the internal standard. High resolution mass spectra (HRMS) were obtained using an Exactive Mass Spectrometer (Agilent 1200HPLC/MicrOTOF II) equipped with an ESI ionization source. Melting points were determined using XRC-1 and are uncorrected.

Materials. Unless otherwise noted, materials were purchased and used as received from Tokyo Chemical Industry Co., Aldrich Inc., Alfa Aesar, and other commercial suppliers. Solvents were dried over CaH_2 (for DCE, $CHCl_3$, and DMF) or sodium (for toluene and 1,4-dioxane) by refluxing overnight and freshly distilled prior to use.

2. Experimental Procedure

2.1 Preparation of epoxides

Epoxides **1b-1k** were purchased commercially.

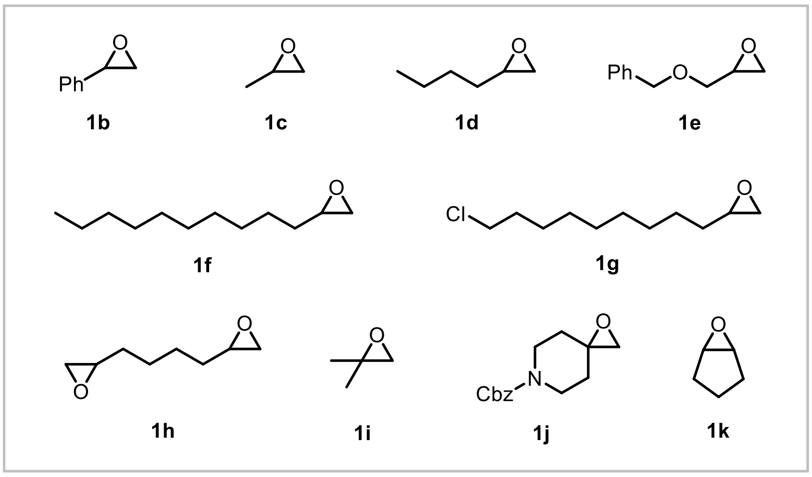

General Procedure 1 (GP1): Oxidation of olefins to epoxides[1-4]

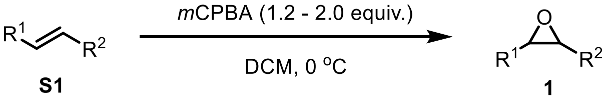

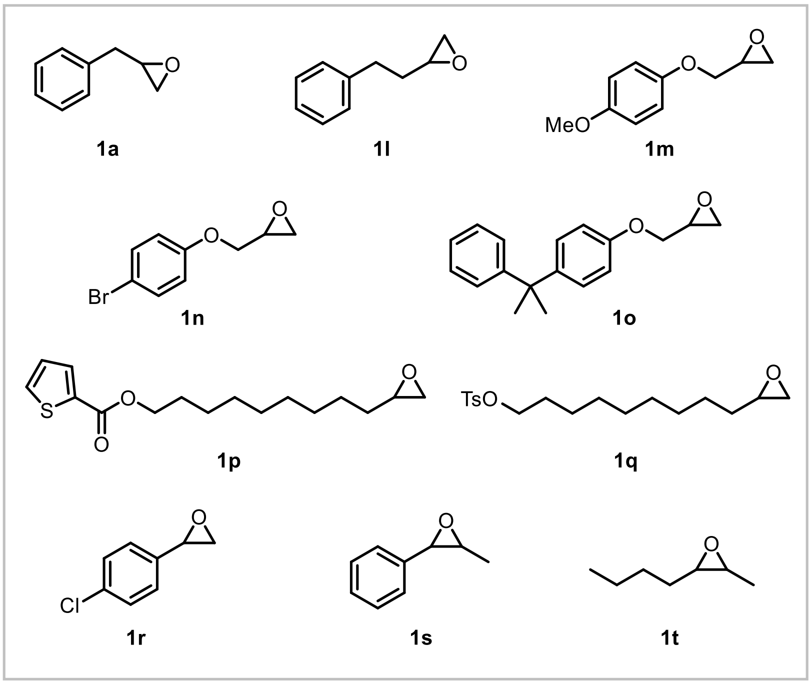

To an oven-dried 10.0 mL Schlenk tube equipped with a stir bar, olefins (2.0 mmol, 1.0 equiv.) in dry DCM (15.0 mL) were added and stirred at 0 °C. Subsequently, *m*CPBA (1.2 to 2.0 equiv.) was added in batches over 10 min and the reaction mixture was stirred until the complete consumption of starting materials (as evidenced by TLC). The reaction mixture was quenched with saturated $NaHCO_3$ solution and

the mixture was extracted with DCM (10.0 mL x 3). The organic layer was then washed with saturated $NaHCO_3$ solution and saturated saline, dried over Na_2SO_4, and concentrated under reduced pressure. The crude product was then purified by flash column chromatography using Et_3N (1%) deactivated silica gel (petroleum ether as eluent) to afford desired epoxides (yield is 50-90%).

2-((4-methoxyphenoxy)methyl)oxirane (1m)

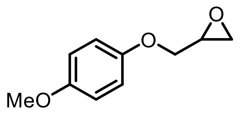

Following the **GP1**: The product **1m** was obtained as a colorless oil (288 mg, 80% yield).

^{1}H NMR (500 MHz, $CDCl_3$) δ 6.92-6.78 (m, 4H), 4.17 (dd, J = 11.0, 3.0 Hz, 1H), 3.92 (dd, J = 11.0, 5.5 Hz, 1H), 3.77 (s, 3H), 3.37-3.30 (m, 1H), 2.89 (dd, J = 5.0, 4.0 Hz, 1H), 2.74 (dd, J = 5.0, 3.0 Hz, 1H).

^{13}C NMR (125 MHz, $CDCl_3$) δ 154.2, 152.6, 115.7, 114.6, 69.5, 55.7, 50.3, 44.7.

HRMS (ESI$^+$) m/z calcd for $C_{10}H_{13}O_3$ $[M+H]^+$ 181.0859, found 181.0860.

2-((4-bromophenoxy)methyl)oxirane (1n)

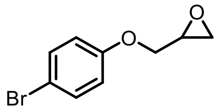

Following the **GP1**: The product **1n** was obtained as a colorless oil (283 mg, 62% yield).

^{1}H NMR (500 MHz, $CDCl_3$) δ 7.40-7.35 (m, 2H), 6.83-6.77 (m, 2H), 4.21 (dd, J = 11.0, 3.0 Hz, 1H), 3.91 (dd, J = 11.0, 6.0 Hz, 1H), 3.38-3.30 (m, 1H), 2.91 (dd, J = 5.0, 4.5 Hz, 1H), 2.75 (dd, J = 5.0, 2.5 Hz, 1H).

^{13}C NMR (125 MHz, $CDCl_3$) δ 157.6, 132.3, 116.5, 113.4, 69.0, 50.0, 44.6.

HRMS (ESI$^+$) m/z calcd for $C_9H_{10}BrO_2$ $[M+H]^+$ 228.9859, found 228.9864.

2-((4-(2-phenylpropan-2-yl)phenoxy)methyl)oxirane (1o)

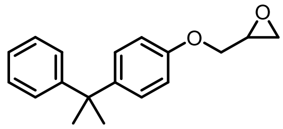

Following the **GP1**: The product **1o** was obtained as a colorless oil (450 mg, 84% yield).

^{1}H NMR (500 MHz, $CDCl_3$) δ 7.28-7.24 (m, 2H), 7.22 (d, J = 7.5 Hz, 2H), 7.18-7.12 (m, 3H), 6.82 (d, J = 8.5 Hz, 2H), 4.17 (dd, J = 11.0, 3.0 Hz, 1H), 3.95 (dd, J = 11.0, 5.5 Hz, 1H), 3.36-3.30 (m, 1H), 2.90-2.87 (m, 1H), 2.73 (dd, J = 5.0, 3.0 Hz, 1H), 1.65 (s, 6H).

^{13}C NMR (125 MHz, $CDCl_3$) δ 156.3, 150.8, 143.5, 128.0, 127.8, 126.7, 125.5, 114.0, 68.7, 50.2, 44.8, 42.3, 30.8.

HRMS (ESI$^+$) m/z calcd for $C_{18}H_{21}O_2$ $[M+H]^+$ 269.1536, found 269.1540.

9-(oxiran-2-yl)nonyl thiophene-2-carboxylate (1p)

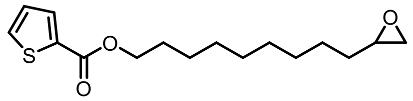

Following the **GP1**: The product **1p** was obtained as a colorless oil (338 mg, 57% yield).

^{1}H NMR (600 MHz, $CDCl_3$) δ 7.79 (dd, *J* = 3.6, 0.6 Hz, 1H), 7.54 (dd, *J* = 4.8, 0.6 Hz, 1H), 7.09 (dd, *J* = 4.8, 3.6 Hz, 1H), 4.29 (t, *J* = 6.6 Hz, 2H), 2.94-2.86 (m, 1H), 2.77-2.71 (m, 1H), 2.46 (dd, *J* = 5.4, 3.0 Hz, 1H), 1.77-1.71 (m, 2H), 1.55-1.50 (m, 2H), 1.50-1.39 (m, 4H), 1.37-1.29 (m, 8H).

^{13}C NMR (150 MHz, $CDCl_3$) δ 162.2, 134.0, 133.1, 132.1, 127.6, 65.2, 52.3, 47.0, 32.4, 29.4, 29.3, 29.1, 28.6, 25.9, 25.9.

HRMS (ESI$^+$) m/z calcd for $C_{16}H_{25}O_3S$ $[M+H]^+$ 297.1519, found 297.1520.

9-(oxiran-2-yl)nonyl 4-methylbenzenesulfonate (1q)

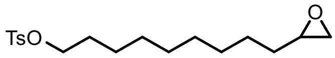

Following the **GP1**: The product **1q** was obtained as a colorless oil (422 mg, 62% yield).

^{1}H NMR (600 MHz, $CDCl_3$) δ 7.79 (d, *J* = 7.8 Hz, 2H), 7.35 (d, *J* = 7.8 Hz, 2H), 4.02 (t, *J* = 6.6 Hz, 2H), 2.93-2.87 (m, 1H), 2.77-2.73 (m, 1H), 2.46 (dd, *J* = 4.8, 2.4 Hz, 1H), 2.45 (s, 3H), 1.66-1.61 (m, 2H), 1.56-1.49 (m, 2H), 1.48-1.38 (m, 2H), 1.34-1.21 (m, 10H).

^{13}C NMR (125 MHz, $CDCl_3$) δ 144.6, 133.2, 129.8, 127.9, 70.7, 52.4, 47.1, 32.4, 29.3, 29.2, 28.9, 28.8, 25.9, 25.3, 21.6.

HRMS (ESI$^+$) m/z calcd for $C_{18}H_{29}O_4S$ $[M+H]^+$ 341.1781, found 341.1790.

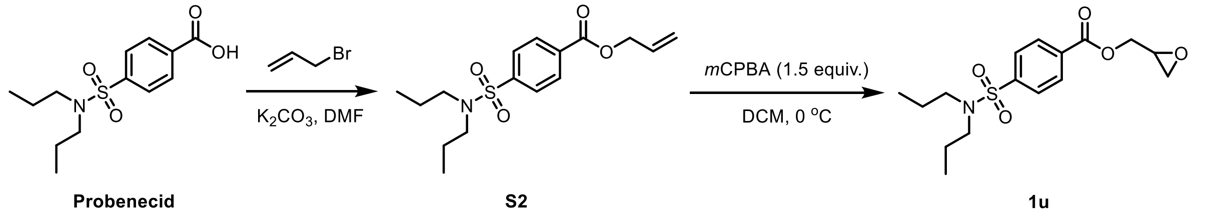

To a round-bottomed flask, K_2CO_3 (3.0 mmol, 1.5 equiv.), DMF (5.0 mL), Probenecid (2.0 mmol, 1.0 equiv.), and 3-bromoprop-1-ene (3.0 mmol, 1.5 equiv.) were added at room temperature. After stirring for 10 hours at room temperature, the organic phase was washed with water, extracted with ethyl acetate, dried over $MgSO_4$, and concentrated under reduced pressure. The crude produce was then purified by column chromatography over silica gel using petroleum ether/ethyl acetate (PE/EtOAc = 20:1) as the eluent afforded the product (84% yield). The corresponding epoxide **1u** was prepared using the **GP1**.

General Procedure 2 (GP2): Preparation of epoxides by Corey-Chaykovsky reaction[4]

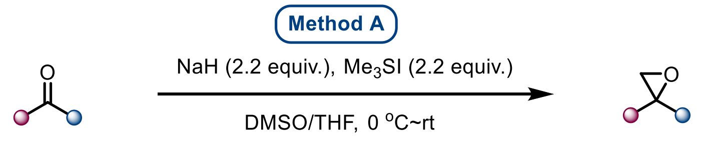

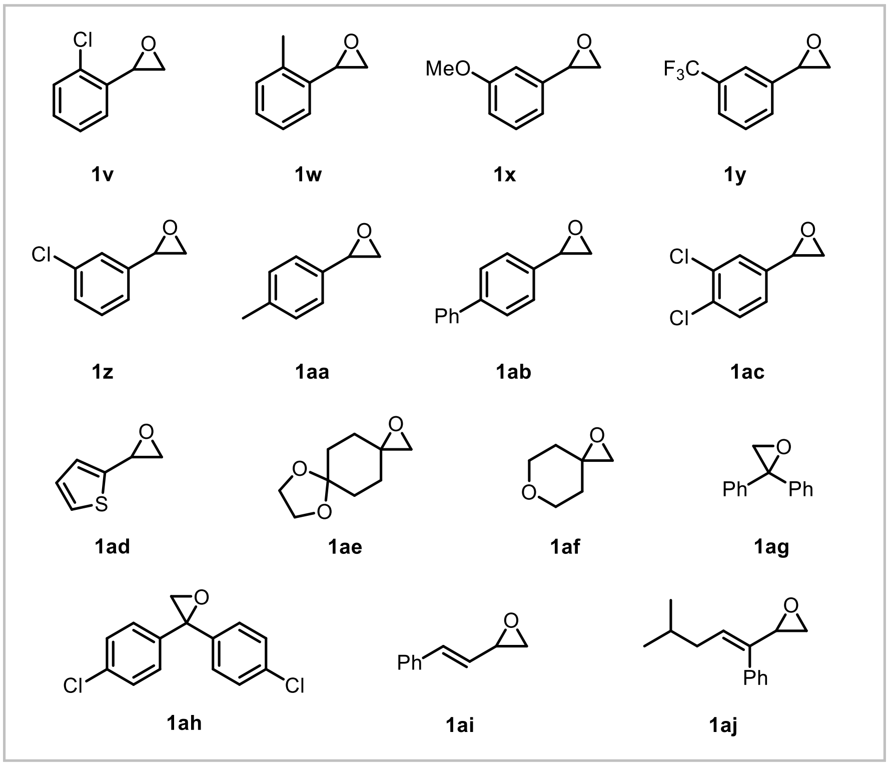

Method A: In an oven-dried flask, NaH (4.4 mmol, 2.2 equiv.) was dissolved in a mixture of anhydrous DMSO (3.0 mL) and anhydrous THF (2.0 mL), and then trimethylsulfonium iodide (4.4 mmol, 2.2 equiv.) was added and stirred at room temperature for 30 minutes. After cooling the reaction mixture to 0 °C, **S3** (2 mmol, 1.0 equiv.) dissolved in anhydrous THF was added, and the mixture was allowed to reach room temperature and stirred overnight. After completion of the reaction, as monitored by TLC, the reaction was quenched with saturated NH_4Cl solution and the mixture was extracted with DCM (5.0 mL × 3). The combined organic layer was then washed with saturated $NaHCO_3$ solution and saturated brine, dried over Na_2SO_4, and concentrated under reduced pressure. The crude product was then purified by column chromatography using Et_3N (1%) deactivated silica gel (petroleum ether as eluent) to afford the desired epoxides.

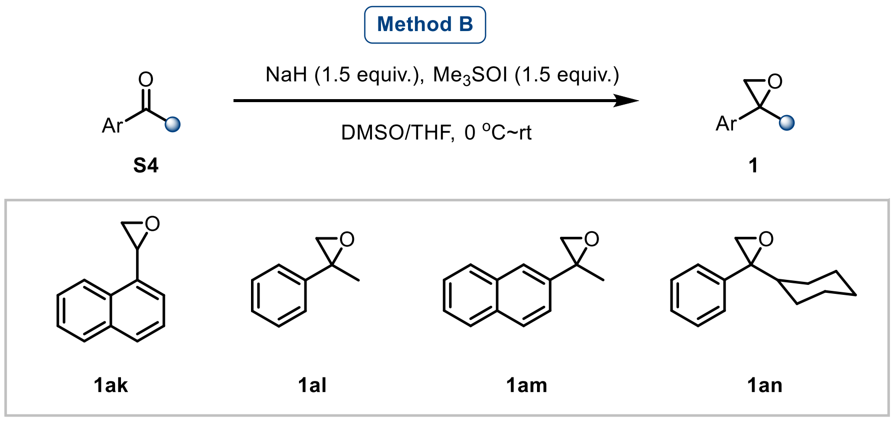

Method B: In an oven-dried flask, NaH (4.4 mmol, 2.2 equiv.) was dissolved in a mixture of anhydrous DMSO (3.0 mL) and anhydrous THF (2.0 mL), and then trimethylsulfoxonium iodide (3.0 mmol, 1.5 equiv.) was added and stirred at room temperature for 30 minutes. After cooling the reaction mixture to 0 °C, **S4** (2.0 mmol, 1.0 equiv.) dissolved in anhydrous THF was added, and the mixture was allowed to reach room temperature and stirred overnight. After completion of the reaction, as monitored by TLC, the reaction was quenched with saturated NH_4Cl solution and the mixture was extracted with DCM (5.0 mL × 3). The combined organic layer was then washed with saturated $NaHCO_3$ solution and saturated brine, dried over Na_2SO_4, and concentrated under reduced pressure. The crude product was then purified by column chromatography using Et_3N (1%) deactivated silica gel (petroleum ether as eluent) to afford the desired epoxide**s**.

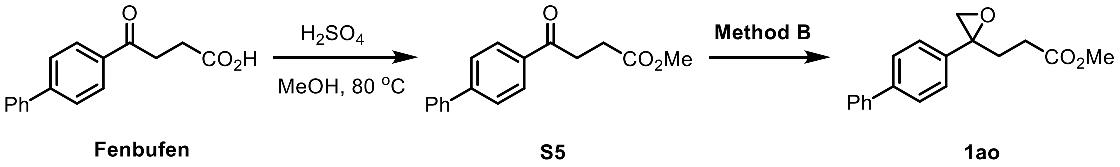

Under a nitrogen atmosphere, 0.8 mL of concentrated sulfuric acid was added to fenbufen (2.0 mmol) in 10.0 mL of anhydrous methanol in an oven-dried flask and the mixture was stirred at 80 °C overnight. After cooling to room temperature, the methanol was concentrated and a saturated sodium bicarbonate solution was added to neutralize the excess acid to a pH of 7. Then the mixture was extracted with ethyl acetate (5.0 mL x 3) and the combined organic phase was dried over anhydrous sodium sulfate and proceeded directly to the next step using method B.

methyl 3-(2-([1,1'-biphenyl]-4-yl)oxiran-2-yl)propanoate (1ao) Following the **GP2** (method B) using **S5** (268 mg, 1.0 mmol, 1.0 equiv.) and trimethylsulfoxonium iodide (330 mg, 1.5 mmol, 1.5 equiv.), the crude product was purified by flash column chromatography on deactivated silica gel with Et_3N (1%) to provide the analytically pure product **1ao** in 84% yield. White solid; mp: 77-78 °C.

^{1}H NMR (500 MHz, $CDCl_3$) δ 7.60-7.54 (m, 4H), 7.46-7.40 (m, 4H), 7.34 (t, J = 7.0 Hz, 1H), 3.64 (s, 3H), 3.02

(d, J = 5.0 Hz, 1H), 2.79 (d, J = 5.0 Hz, 1H), 2.67-2.57 (m, 1H), 2.50-2.33 (m, 2H), 2.15-2.05 (m, 1H).

^{13}C NMR (150 MHz, $CDCl_3$) δ 173.4, 140.6, 140.6, 138.1, 128.8, 127.4, 127.2, 127.0, 126.3, 59.3, 55.7, 51.6, 30.5, 29.5.

IR (Film): 3031, 2993, 2951, 1737, 1487, 1437, 1257, 1198, 1168, 840, 766, 732, 699 cm^{-1}.

HRMS (ESI$^+$) m/z calcd for $C_{18}H_{19}O_3$ [M+H]$^+$ 283.1329, found 283.1333.

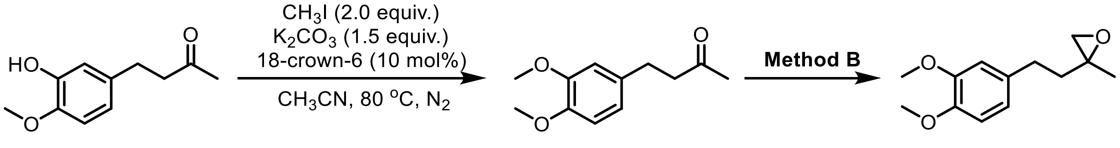

S6 **1ap**

In a 50.0 mL flask, K_2CO_3 (1.5 equiv.) and curcumone (2.0 mmol) were added. The system was purged with nitrogen three times, then acetonitrile (15.0 mL), 18-crown-6, and iodomethane (2.0 equiv.) were added and the mixture was heated at 80 °C. After complete consumption of starting material, the mixture was cooled to room temperature, and extracted with ethyl acetate (10.0 mL x 3). The combined organic phases were dried over sodium sulfate and the filtrate was concentrated under reduced pressure. The crude product was then purified by silica gel column chromatography to provide **S6** (96% yield) as a white solid.

2-(3,4-dimethoxyphenethyl)-2-methyloxirane (1ap) Following the **GP2** (method B) using **S6** (208 mg, 1 mmol, 1.0 equiv.) and trimethylsulfoxonium iodide (330 mg, 1.5 mmol, 1.5 equiv.), the crude product was purified by flash column chromatography on deactivated silica gel with Et_3N (1%) to provide the analytically pure product **1ap** in 91% yield. Colorless oil.

^{1}H NMR (500 MHz, $CDCl_3$) δ 6.79 (d, J = 8.0 Hz, 1H), 6.74-6.69 (m, 2H), 3.87 (s, 3H), 3.85 (s, 3H), 2.70-2.63 (m, 2H), 2.62 (d, J = 4.5 Hz, 1H), 2.58 (d, J = 4.5 Hz, 1H), 1.95-1.78 (m, 2H), 1.38 (s, 3H).

^{13}C NMR (150 MHz, $CDCl_3$) δ 148.8, 147.2, 134.1, 120.0, 111.6, 111.2, 56.6, 55.8, 55.8, 53.9, 38.7, 30.9, 21.0.

IR (Film): 2935,2835, 1607, 1590, 1516, 1464, 1264, 1236, 1156, 1142, 1030, 806, 765 cm^{-1}.

HRMS (ESI$^+$) m/z calcd for $C_{13}H_{19}O_3$ [M+H]$^+$ 223.1329, found 223.1341.

2-(2-(6-methoxynaphthalen-2-yl)ethyl)-2-methyloxirane (1aq)

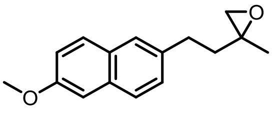

Following the **GP2** (method B) using nabumetone (228.0 mg, 1.0 mmol, 1.0 equiv.) and trimethylsulfoxonium iodide (330.0 mg, 1.5 mmol, 1.5 equiv.), the crude product was purified by flash column chromatography on deactivated silica gel with Et_3N (1%) to provide the analytically pure product **1aq** in 88% yield. White solid; mp: 88-89 °C.

^{1}H NMR (500 MHz, $CDCl_3$) δ 7.67 (d, J = 8.5 Hz, 2H), 7.55 (s, 1H), 7.29 (d, J = 8.0 Hz, 1H), 7.16-7.06 (m,

2H), 3.90 (s, 3H), 2.85 (t, J = 7.5 Hz, 2H), 2.62 (d, J = 4.5 Hz, 1H), 2.58 (d, J = 4.5 Hz, 1H), 2.05-1.85 (m, 2H), 1.41 (s, 3H).

^{13}C NMR (150 MHz, $CDCl_3$) δ 157.2, 136.7, 133.0, 129.1, 128.8, 127.5, 126.8, 126.1, 118.7, 105.6, 56.7, 55.2, 53.9, 38.5, 31.3, 21.0.

IR (Film): 2935, 2835, 1607, 1516, 1464, 1264, 1236, 1156, 1142, 1030, 806, 765 cm^{-1}.

HRMS (ESI^+) m/z calcd for $C_{16}H_{19}O_2$ $[M+H]^+$ 243.1380, found 243.1391.

isopropyl 2-(4-(2-(4-chlorophenyl)oxiran-2-yl)phenoxy)-2-methylpropanoate (1ar)

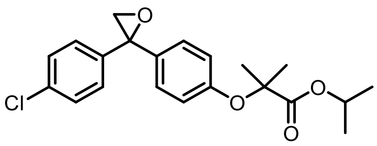

Following the **GP2** (method B) using fenofibrate (361.0 mg, 1.0 mmol, 1.0 equiv.) and trimethylsulfoxonium iodide (330.0 mg, 1.5 mmol, 1.5 equiv.), the crude product was purified by flash column chromatography on deactivated silica gel with Et_3N (1%) to provide the analytically pure product **1ar** in 70% yield. White solid; mp: 102-103 °C.

^{1}H NMR (500 MHz, $CDCl_3$) δ 7.26 (q, J = 8.5 Hz, 4H), 7.18 (d, J = 8.5 Hz, 2H), 6.81 (d, J = 8.5 Hz, 2H), 5.13-5.02 (m, 1H), 3.28 (d, J = 5.5 Hz, 1H), 3.15 (d, J = 5.5 Hz, 1H), 1.59 (s, 6H), 1.21 (s, 3H), 1.20 (s, 3H).

^{13}C NMR (150 MHz, $CDCl_3$) δ 173.4, 155.5, 138.4, 133.7, 132.1, 128.7, 128.4, 128.3, 118.5, 79.1, 68.9, 60.9, 56.7, 25.3, 25.2, 21.4.

HRMS (ESI^+) m/z calcd for $C_{21}H_{24}ClO_4$ $[M+H]^+$ 375.1358, found 375.1361.

Process for synthesis of 1as[5] and 1at[6]:

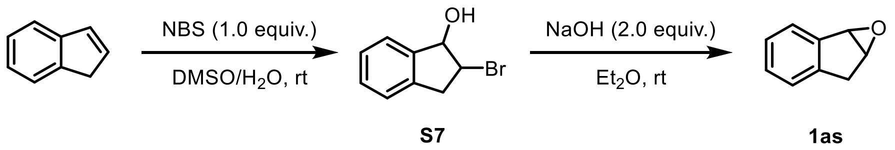

Indene (5.0 mmol, 1.0 equiv.), 10.0 mL DMSO, and 0.5 mL H_2O were added to a round-bottom flask containing a magnetic bar. The solution was then cooled to 0 °C, NBS (5.0 mmol, 1.0 equiv.) was added slowly and the mixture was stirred at room temperature for 2 h. After completion, the reaction mixture was diluted with water and extracted with DCM (10.0 mL x 3). The combined organic phases were dried over sodium sulfate and concentrated under reduced pressure. The crude product was purified by silica gel column chromatography afforded the colorless liquid **S7** (85% yield).

To a solution of **S7** (3.0 mmol, 1.0 equiv.) in ether (10.0 mL), NaOH (6.0 mmol, 2.0 equiv.) was added in batches and stirred at room temperature overnight. After completion, the reaction mixture was diluted with water and extracted with DCM (10.0 mL x 3). The combined organic phases were dried over sodium sulfate and concentrated under reduced pressure. The crude product was purified by column

chromatography using Et_3N (1%) deactivated silica gel to obtain the desired epoxide **1as** in 65% yield.

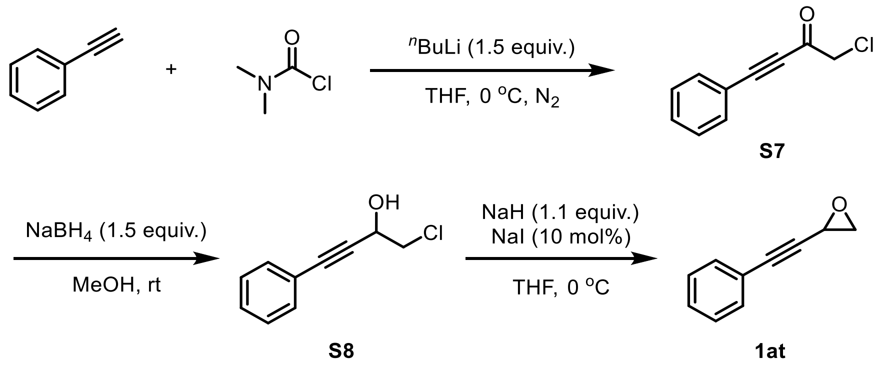

To a stirred solution of phenylacetylene (1.94 mL, 15 mmol) in THF (30.0 mL), ^{n}BuLi (1.65 M in hexane, 9.1 mL, 15 mmol) was added dropwise at 0 °C and stirred for 30 minutes. Then a solution of amide (1.38 g, 10 mmol) in THF (20.0 mL) was added dropwise at the same temperature and the reaction mixture was stirred for 30 minutes. After completion, the reaction mixture was quenched with 1 N HCl and extracted three times with Et_2O. The combined organic phases were dried over Na_2SO_4 and concentrated under reduced pressure. The crude product was purified by silica gel column chromatography (hexane/AcOEt=20/1 to 10/1) to give **S7** (80%).

To the stirred solution of **S7** (1.53 g, 8.5 mmol) in MeOH (50.0 mL), $NaBH_4$ (0.48 g, 12.8 mmol) was added at 0 °C. The resulting mixture was stirred at room temperature for 2 hours. After completion, the reaction was quenched with water and concentrated before extraction with DCM. The combined organic phases were washed with salt water, dried over Na_2SO_4, and concentrated under reduced pressure. The crude product was purified by silica gel column chromatography (hexane/AcOEt=10/1 to 5/1) to obtain **S8** (94% yield).

In an oven-dried flask, NaH (2.5 mmol) and NaI (0.23 mmol) were dissolved in THF (3.0 mL) and then **S8** (2.3 mmol) in THF was added dropwise at 0 °C THF. After 1 h of reaction, the reaction mixture was quenched with water, extracted with DCM, and dried over Na_2SO_4. The crude product was purified by column chromatography using Et_3N (1%) deactivated silica gel afforded the desired epoxide **1at** in 77% yield.

2.2 **General procedure for the synthesis of *N*-sulfonylhydrazones**

Synthesis of *o*-(trifluoromethyl)benzenesulfonohydrazide

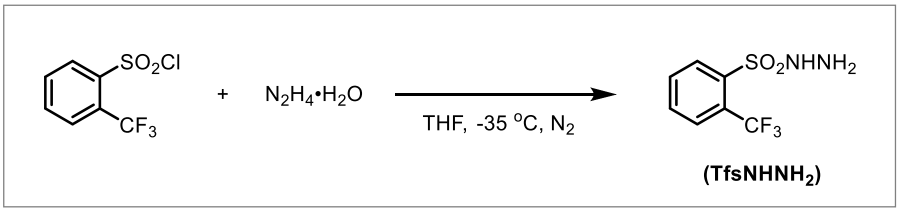

To a round bottom flask, *o*-trifluoromethylbenzenesulfonyl chloride (24.4 g, 100.0 mmol, 1.0 equiv.) was dissolved in THF (120.0 mL) under an argon atmosphere. The reaction mixture was then cooled to -35 °C and hydrazine hydrate (24.0 mL, 250.0 mmol, 2.5 equiv.) was added dropwise. After complete consumption of starting materials (as evidenced by TLC), the reaction mixture was extracted with ethyl acetate (60.0 mL x 3), and washed with saturated sodium chloride solution. The combined organic phases were dried over anhydrous Na_2SO_4 and concentrated under vacuum. The remaining solution was slowly added to 500.0 mL of cold petroleum ether to precipitate the product, filtered, and the precipitate was dried under vacuum to obtain a white solid with a yield of 88%.

Synthesis of *N*-triftosylhydrazone 2a

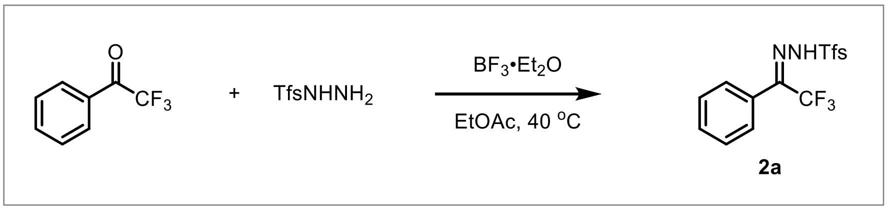

Under air conditions, 2,2,2-trifluoroacetophenone (1.92 g, 11 mmol, 1.1 equiv.) and 20.0 mL EtOAc was added in an oven-dried 50.0 mL round-bottomed flask, and then $TfsNHNH_2$ (2.4 g, 10 mmol, 1.0 equiv.) was added and the reaction mixture was stirred at 40 °C. Upon complete dissolution, 2.0 mL of boron trifluoride ether (48 wt% BF_3) was added. After complete consumption of the starting material (as evidenced by TLC), saturated sodium chloride solution was added and then extracted with EtOAc (20.0 mL x 3). The combined organic layers were dried over anhydrous Na_2SO_4 and concentrated under reduced pressure. The crude product was purified by silica gel column chromatography (PE/EtOAc = 10/1) to obtain a white solid (3.33 g, 84% yield).

Note: *1,1,1-trifluoro-4-phenylbut-3-en-2-one can obtain the corresponding N-o-trifluorobenzenesulfonylhydrazone by using the same method with a yield of 90%.*

Synthesis of *N*-sulfonylhydrazones 2b and 2c

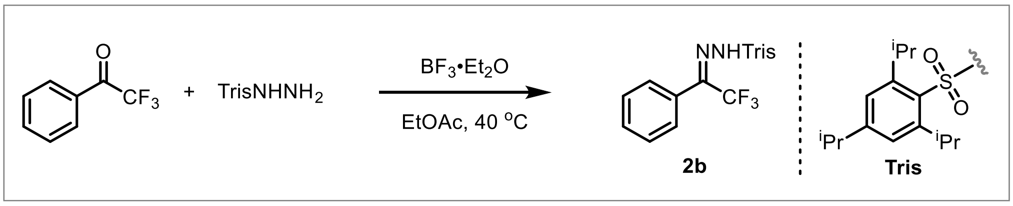

Under air conditions, 2,2,2-trifluoroacetophenone (1.92 g, 11.0 mmol, 1.1 equiv.) and 20.0 mL EtOAc were added in an oven-dried 50.0 mL round-bottomed flask and then 2,4,6-trisopropylbenzenesulphonylhydrazide ($TrisNHNH_2$) (2.98 g, 10.0 mmol, 1.0 equiv.) was added and the reaction mixture was stirred at 40 °C. Upon complete dissolution, 2.0 mL of boron trifluoride ether (48 wt% BF_3) was added. After complete consumption of the starting material (as evidenced by TLC), saturated sodium chloride solution was added and then extracted with EtOAc (20.0 mL x 3). The combined organic layers were dried over anhydrous Na_2SO_4 and concentrated under reduced pressure. The crude product was purified by silica gel column chromatography (PE/EtOAc = 10:1) to obtain a white solid (4.07 g, 82% yield).

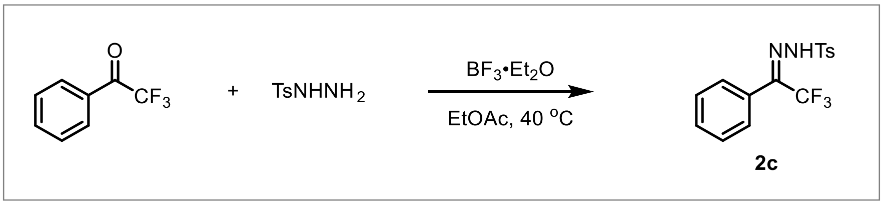

Under air conditions, 2,2,2-trifluoroacetophenone (1.92 g, 11.0 mmol, 1.1 equiv.) and 20.0 mL EtOAc was added in an oven-dried 50.0 mL round-bottomed flask and then 4-toluenesulfonyl hydrazine (1.86 g, 10.0 mmol, 1.0 equiv.) was added and the reaction mixture was stirred at 40 °C. Upon complete dissolution, 2.0 mL of boron trifluoride ether (48 wt% BF_3) was added. After complete consumption of the starting material (as evidenced by TLC), saturated sodium chloride solution was added and then extracted with EtOAc (20.0 mL x 3). The combined organic layers were dried over anhydrous Na_2SO_4 and concentrated under reduced pressure. The crude product was purified by silica gel column chromatography (PE/EtOAc = 10/1) to obtain a white solid (2.74 g, 80% yield).

Synthesis of *N*-triftosylhydrazones 2e-2g

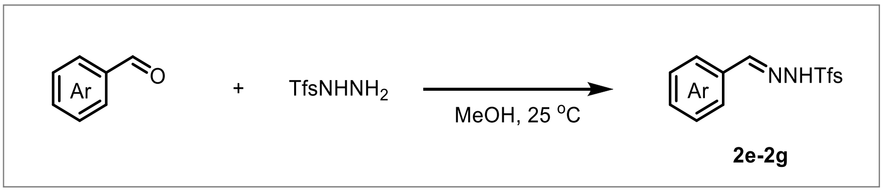

To a 50.0 mL oven-dried round bottom flask, benzaldehyde (1.17 g, 11.0 mmol, 1.1 equiv.), methanol (15.0 mL), and $TfsNHNH_2$ (2.4 g, 10.0 mmol, 1.0 equiv.) were added under air and the mixture was stirred at room temperature for 1 hour. The solid generated was filtered and washed with petroleum ether:diethyl

ether (10:1) to obtain white powdery solid with a yield of 95%. The same procedure followed for the preparation of other aryl *N*-triftosylhydrazones.

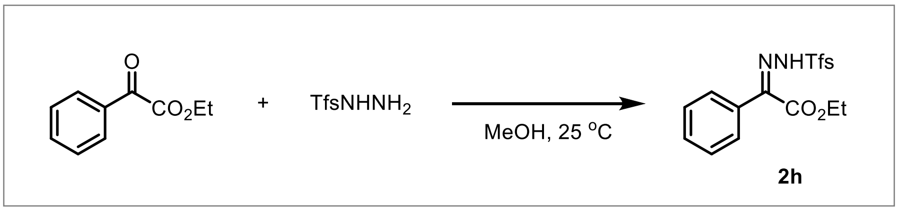

To a 50.0 mL oven-dried round bottom flask, ethyl benzoylformate (1.81 g, 11.0 mmol, 1.1 equiv.), methanol (15.0 mL), and $TfsNHNH_2$ (2.4 g, 10.0 mmol, 1.0 equiv.) were added under air and stirred at room temperature for 1 hour. The solid generated was filtered and washed with petroleum ether:diethyl ether (10:1) to obtain a white powdery solid with a yield of 92%.

Preparation of *N*-triftosylhydrazones derived from drugs and natural products

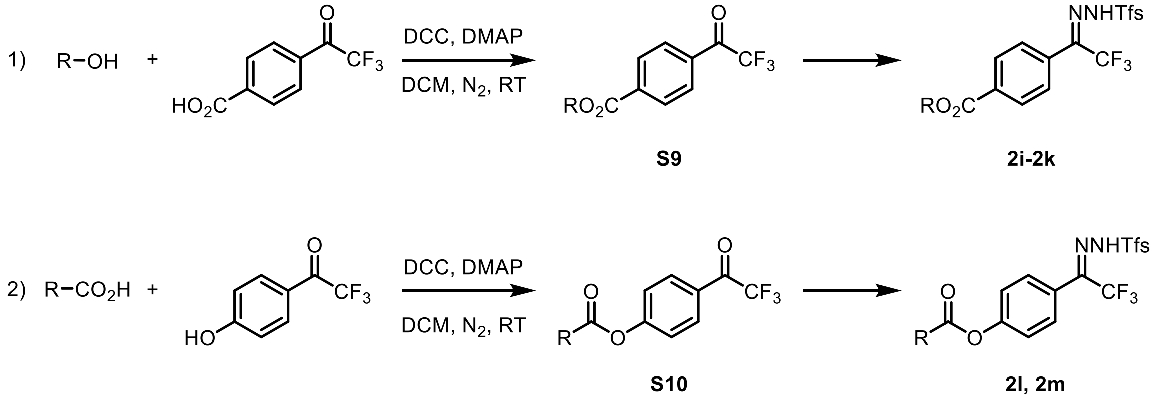

General procedure: (with 2l as an example)

To an oven-dried Schlenk tube, 2,2,2-trifluoro-1-(4-hydroxyphenyl)ethan-1-one (1.0 mmol, 1.0 equiv.), indometacin (1.0 equiv.), DCC (1.1 equiv.), and DMAP (20 mol%) were added. The flask was purged with nitrogen (3 times), dichloromethane (5.0 mL) was added and the solution was stirred at room temperature overnight. After completion of the reaction, the reaction mixture was washed with water and extracted with DCM. The combined organic phases were dried over Na_2SO_4 and concentrated under reduced pressure to obtain the condensation product **S11**.

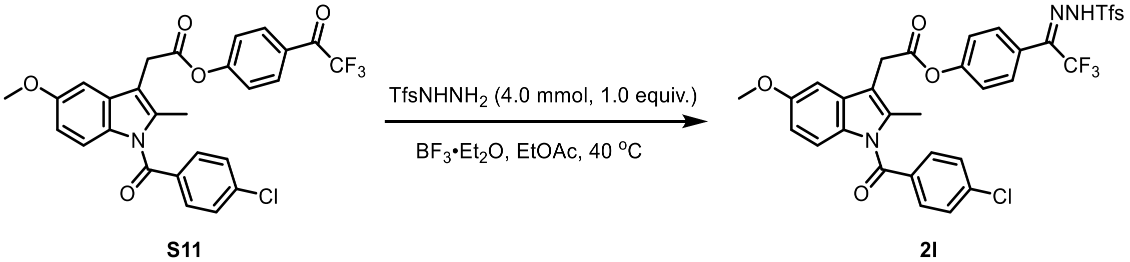

***N*-*o*-trifluorobenzenesulfonylhydrazone derived from indometacin (2l)**

Under air conditions, **S11** (2.12 g, 4.0 mmol, 1.0 equiv.) and 15.0 mL EtOAc were added in an oven-dried

50.0 mL round-bottomed flask and then 2,2,2-trifluoroacetophenone (0.77 g, 4.4 mmol, 1.1 equiv.) was added and the reaction mixture was stirred at 40 °C. Upon complete dissolution, 2.0 mL of boron trifluoride ether (48 wt% BF_3) was added. After complete consumption of the starting material (as evidenced by TLC), saturated sodium chloride solution was added and then extracted with EtOAc (20.0 mL x 3). The combined organic layers were dried over anhydrous Na_2SO_4 and concentrated under reduced pressure. The crude product was purified by silica gel column chromatography (PE/EtOAc = 10/1) to obtain a white solid (422 mg, 62% yield; mp: 179-180 °C).

^{1}H NMR (600 MHz, $CDCl_3$) δ 8.46-8.37 (m, 1H), 8.16 (s, 1H), 7.93-7.87 (m, 1H), 7.84-7.76 (m, 2H), 7.68 (d, *J* = 8.4 Hz, 2H), 7.48 (d, *J* = 8.4 Hz, 2H), 7.29 (t, *J* = 9.0 Hz, 3H), 7.05 (d, *J* = 2.4 Hz, 1H), 6.89 (d, *J* = 9.0 Hz, 1H), 6.71 (dd, *J* = 9.0, 2.4 Hz, 1H), 3.95 (s, 2H), 3.85 (s, 3H), 2.48 (s, 3H).

^{13}C NMR (150 MHz, $CDCl_3$) δ 168.6, 168.3, 156.2, 153.0, 141.1 (q, *J* = 36.0 Hz), 139.5, 136.4, 135.8, 134.0, 133.9, 133.7, 132.7, 131.2, 129.6, 129.2, 128.5 (q, *J* = 6.0 Hz), 127.7 (q, *J* = 33.0 Hz), 123.3, 122.6 (q, *J* = 273.0 Hz), 122.3, 119.7 (q, *J* = 273.0 Hz), 115.1, 111.8, 111.4, 101.2, 55.8, 30.5, 13.4.

^{19}F NMR (564 MHz, $CDCl_3$) δ -58.26 (s), -68.49 (s).

IR (Film): 3189, 2933, 1760, 1684, 1602, 1479, 1458, 1371, 1310, 1172, 1130, 755, 738, 717 cm^{-1}.

HRMS (ESI^+) m/z calcd for $C_{34}H_{25}ClF_6N_3O_6S$ $[M+H]^+$ 752.1051, found 752.1060.

2.3 Synthesis of silver catalyst

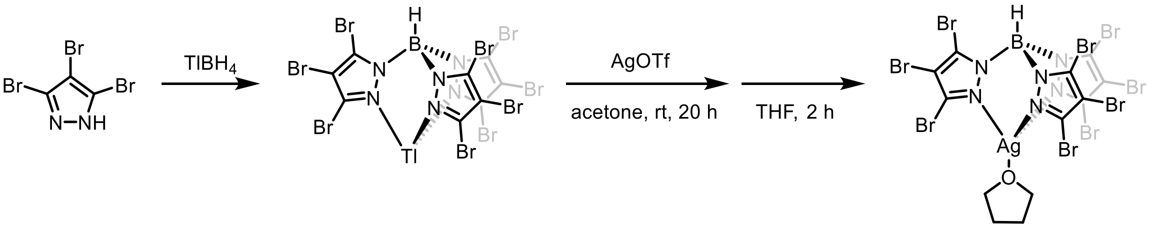

Tp^{Br3}Ag(thf) was synthesized according to the previously reported literature[7] To a 500.0 mL Schlenk tube, pre-sublimed 1*H*-3,4,5-tribromopyrazole (12.19 g, 40.0 mmol) and $TlBH_4$ (2.19 g, 10.0 mmol) were added. The tube was closed with a rubber septum and purged with nitrogen (3 times) and then the septum was replaced with a reflux condenser fitted with a bubbler on top. The nitrogen flow was stopped and the solid mixture was warmed to 180-185 °C for 2 hours, and then the temperature was raised to 200 °C and the reaction was continued for an additional 2 h. After cooling to room temperature, the obtained white solid was directly purified by sublimation to remove the unreacted pyrazole (150 °C, 2 mbar) to give TlTp^{Br3} as a white solid (13.4 g, 89% yield).

Next, the silver triflate (1.29 g, 5.0 mmol) was added to a solution of TlTp^{Br3} (5.64 g, 5.0 mmol) in acetone and the solution was stirred in the dark for 20 hours. The white solid generated was filtered off and dried under vacuum to give complex [Tp^{Br3}Ag]$_2$·CH_3COCH_3. [Tp^{Br3}Ag]$_2$·CH_3COCH_3 (5.0 mmol) was stirred for 2 hours in the dark with fresh distilled THF (100.0 mL). After the removal of the volatiles under reduced pressure, a white solid was obtained (5.46 g, quantitative yield).

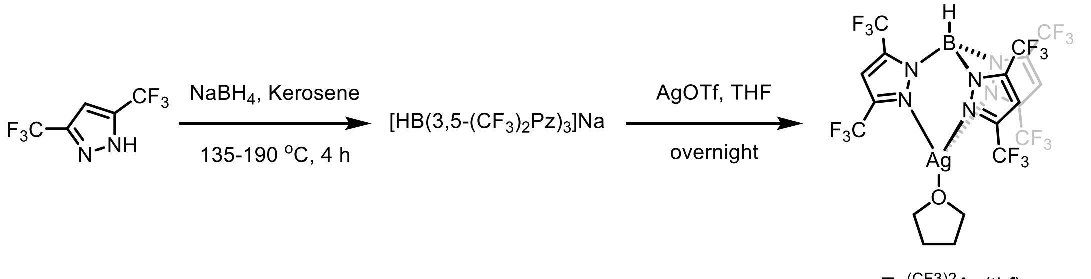

Tp$^{(CF3)2}$Ag(thf) was synthesized according to the previously reported literature[8] To a 500.0 mL Schlenk tube, pre-sublimated 3,5-bis(trifluoromethyl)pyrazole (3.57 g, 17.5 mmol) and sodium borohydride (0.19 g, 5.02 mmol) were mixed in 2.5 mL of kerosene. The reaction mixture was initially heated at 135 °C with stirring for 1 hour and then at 190 °C for 4 hours to give a yellow solid. The solid generated was filtered, washed with light petroleum, and dried under vacuum. The solid was suspended in hexane (30.0 mL) and refluxed for 2 hours and the solid separated was filtered to give [HB(3,5-$(CF_3)_2$Pz)$_3$]Na in 86% yield.

Under nitrogen atmosphere, [HB(3,5-$(CF_3)_2$Pz)$_3$]Na (2.0 mmol, 1.0 equiv.), AgOTf (2.0 mmol, 1.0

equiv.), and 50.0 mL of THF were added in an oven-dried 100.0 mL round-bottomed flask. The mixture was stirred at room temperature overnight. After completion, the solvent was removed under reduced pressure to obtain a gray solid. Then 150.0 mL of *n*-hexane was added to dissolve the solid and filtered through short pad silica to obtain a colorless transparent liquid. The evaporation of *n*-hexane under reduced pressure to obtain pure $Tp^{(CF3)2}Ag(thf)$ (1.28 g, 80%) as white solid.

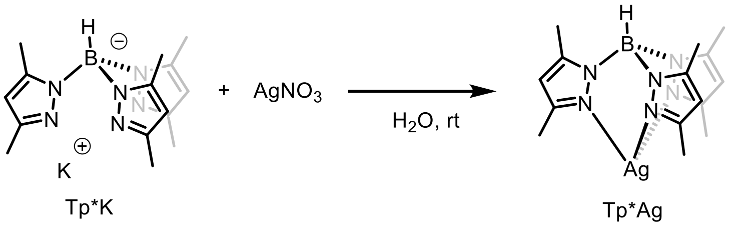

Tp*Ag was synthesized according to the previously reported literature[9] In an oven-dried round bottom flask, the solution of $AgNO_3$ (83.0 mg, 0.5 mmol) in ionic water (5.0 mL) was added to the solution of hydrotris(3,5-dimethylpyrazol-1-yl)borate (160.0 mg, 0.5 mol) in ionic water (40.0 mL). The white precipitate obtained was centrifuged, washed with water and methanol, and dried to obtain Tp*Ag (172.0 mg, 62%) as a white solid.

2.4 **General procedure for the synthesis of cyclopropanation products**

Method C: Procedure for the reaction of alkyl-substituted epoxides

In the glove box, *N*-triftosylhydrazones derived from aryl trifluoromethyl ketones (0.60 mmol, 4.0 equiv.), NaH (0.68 mmol, 4.5 equiv.), and 4.0 mL of DCM were added to the dry-sealed tube. Then the alkyl-substituted epoxides (0.15 mmol) and $Tp^{Br3}Ag(thf)$ (5-10 mol%) were added under stirring conditions. The resulting mixture was sealed and heated to 60 °C. After the reaction was completed and cooled to room temperature, the reaction mixture was filtered through diatomaceous earth under reduced pressure and the filter pad was washed with DCM (5.0 mL × 3). The combined residue was then concentrated under reduced pressure and the crude residue was purified by flash silica gel column chromatography to give cyclopropanation products.

Method D: Procedure for the reaction of aryl-substituted epoxides

In the glove box, *N*-triftosylhydrazones derived from aryl trifluoromethyl ketones (0.38 mmol, 2.5 equiv.), NaH (0.38 mmol, 2.5 equiv.), and 4.0 mL of DCM were added to the dry sealed tube. Then the aryl-substituted epoxides (0.15 mmol) and $Tp^{Br3}Ag(thf)$ (2.5-5 mol%) were added under stirring conditions. The resulting mixture was sealed and heated to 60 °C. After the reaction was completed and cooled to room temperature, the reaction mixture was filtered through diatomaceous earth under reduced pressure and the filter pad was washed with DCM (5.0 mL × 3). The combined residue was then concentrated under reduced pressure and the crude residue was purified by flash silica gel column chromatography to give cyclopropanation products.

Method E: Procedure for the reaction of *N*-triftosylhydrazones derived from aryl aldehyde and epoxides

In the glove box, *N*-triftosylhydrazones derived from aryl aldehydes (0.45 mmol, 3.0 equiv.), NaH (0.45 mmol, 3.0 equiv.), and 4.0 mL of DCM were added to the dry sealed tube. Then the aryl-substituted epoxides (0.15 mmol) and $Tp^{Br3}Ag(thf)$ (5 mol%) were added under stirring conditions. The resulting mixture was sealed and heated to 60 °C. After the reaction was completed and cooled to room temperature, the reaction mixture was filtered through diatomaceous earth under reduced pressure and the filter pad was washed with DCM (5.0 mL × 3). The combined residue was then concentrated under reduced pressure and the crude residue was purified by flash silica gel column chromatography to give cyclopropanation products.

Method F: Gram-scale reaction of styrene oxide and *N*-triftosylhydrazone 2a

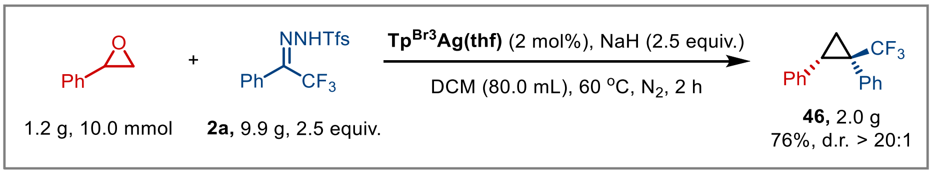

In the glove box, *N*-triftosylhydrazone **2a** (2.5 equiv.), NaH (2.5 equiv.), and 80.0 mL DCM were added to the dry-sealed tube. Then the styrene oxide (10 mmol) and Tp^{Br3}Ag(thf) (2 mol%) were added sequentially under stirring conditions. The resulting mixture was sealed and heated to 60 °C for 2 hours with stirring. After cooling to room temperature, the reaction mixture was filtered through diatomaceous earth under reduced pressure and the filter pad was washed with DCM (5.0 mL × 3). The combined residue was then concentrated under reduced pressure and the crude residue was purified by flash silica gel column chromatography (petroleum ether as eluent) to give product **46** (2.0 g, 76%, d.r. > 20:1).

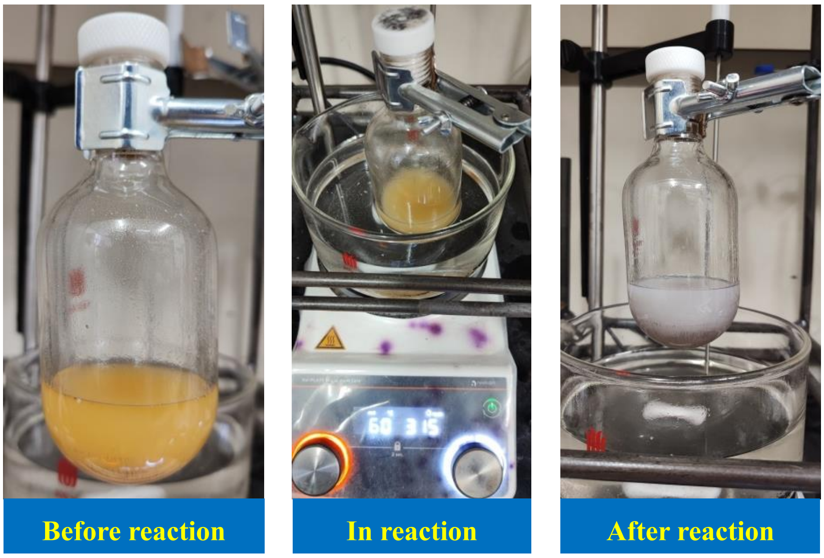

Supplementary Fig. 1a Different time period images of gram-scale reaction.

3 Limitations and supplements

3.1 Non-reactive substrates

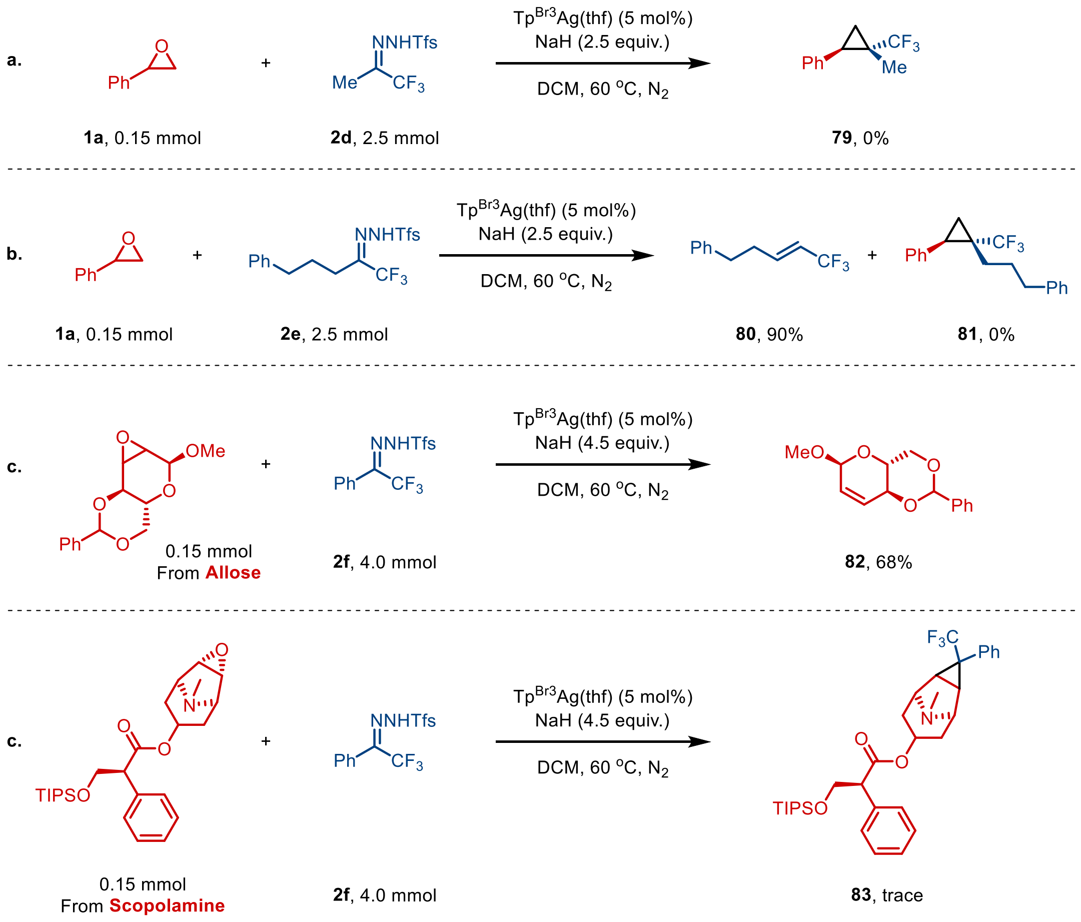

Supplementary Fig. 1b Non-reactive substrates including alkyl *N*-triftosylhydrazones and bioactive molecules.

3.2 (*R*)-styrene oxide 1b' react with *N*-triftosylhydrazone 2a

We observed a complete loss of optical purity in the reaction of non-racemic epoxides.

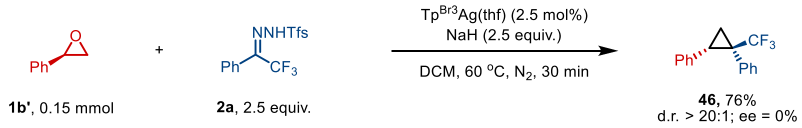

Chiralcel OJ-H, 0 % ^{i}Pr-OH in hexane, 0.8 mL/min λ= 210 nm, RT = 9.375 min; RT = 14.651 min.

Racemate:

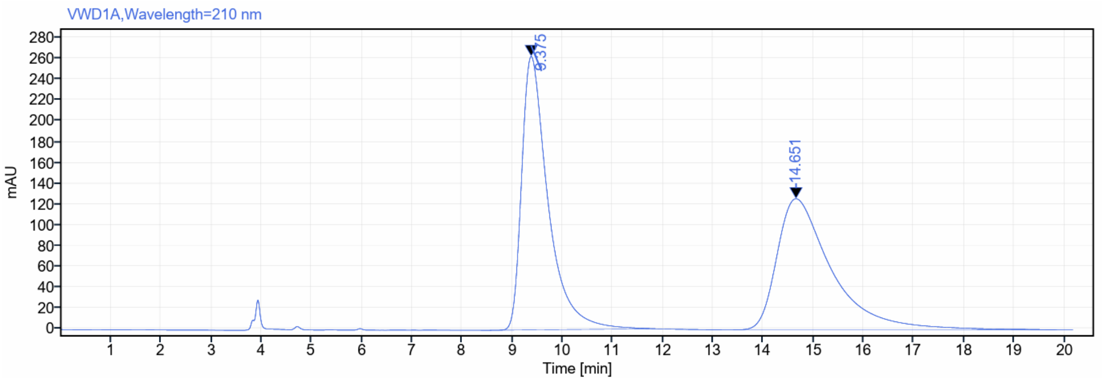

Signal:	VWD1A,Wavelength=210 nm				
RT [min]	Type	Width [min]	Area	Height	Area%
9.375	BM m	3.30	9613.39	262.75	50.26
14.651	BM m	6.39	9512.83	125.90	49.74
		**Sum**	**19126.22**		

Enantiomer:

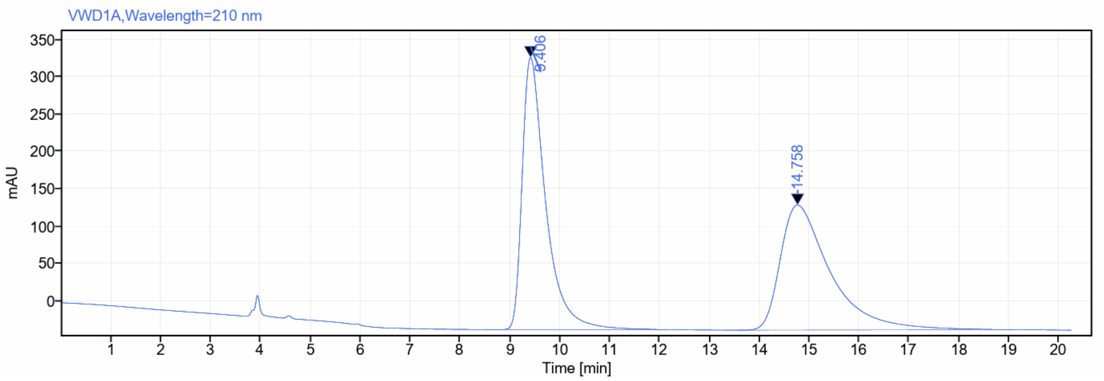

Signal:	VWD1A,Wavelength=210 nm				
RT [min]	Type	Width [min]	Area	Height	Area%
9.406	BM m	3.26	11910.30	364.74	50.50
14.758	BM m	5.52	11672.97	167.55	49.50
		**Sum**	**23583.26**		

3.3 Rh_2(*S*-PTAD)$_4$ instead of Tp^{Br3}Ag(thf)

We tried to realize an enantioselective version of the reaction using chiral rhodium catalyst, Rh_2(*S*-PTAD)$_4$, which afforded the corresponding chiral cyclopropane product **46** in 54% yield with 88% ee. It should be noted that the incompatibility of alkyl-substituted carbenes in the reactions under rhodium-catalyzed conditions is a significant limitation of the process.

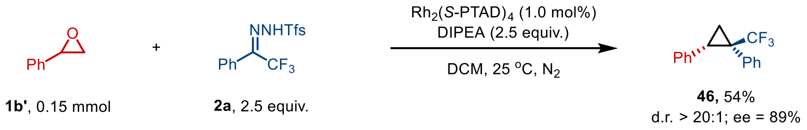

Chiralcel OJ-H, 0 % ^{i}Pr-OH in hexane, 0.8 mL/min λ= 210 nm, RT = 9.566 min, minor; RT = 14.531 min, major.

Racemate:

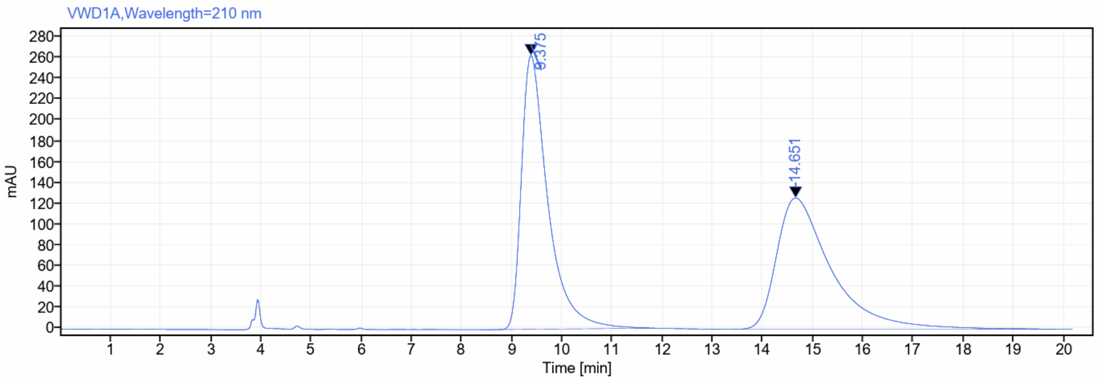

Signal:	VWD1A,Wavelength=210 nm				
**RT [min]**	**Type**	**Width [min]**	**Area**	**Height**	**Area%**
9.375	BM m	3.30	9613.39	262.75	50.26
14.651	BM m	6.39	9512.83	125.90	49.74
		**Sum**	**19126.22**		

Enantiomer:

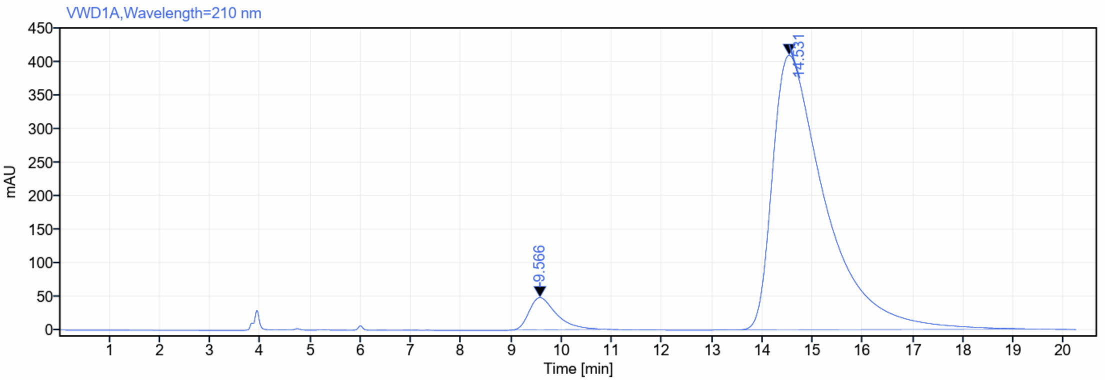

Signal:	VWD1A,Wavelength=210 nm				
**RT [min]**	**Type**	**Width [min]**	**Area**	**Height**	**Area%**
9.566	MM m	1.88	1813.58	48.05	5.73
14.531	MM m	6.50	29859.36	409.30	94.27
		**Sum**	**31672.94**		

4 Computational details

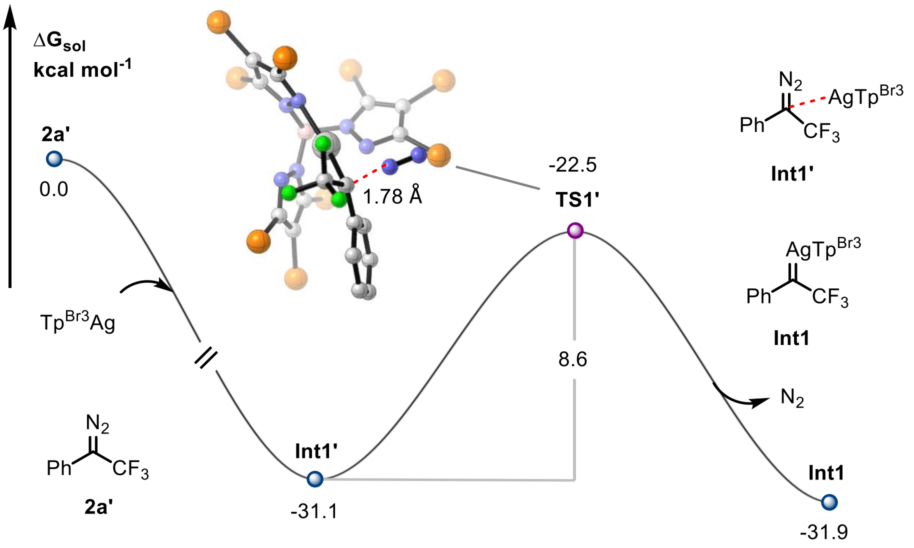

Supplementary Fig. 2a Gibbs free energy profile (in kcal mol^{-1}) of the formation for phenyltrifluoromethyl silver carbine.

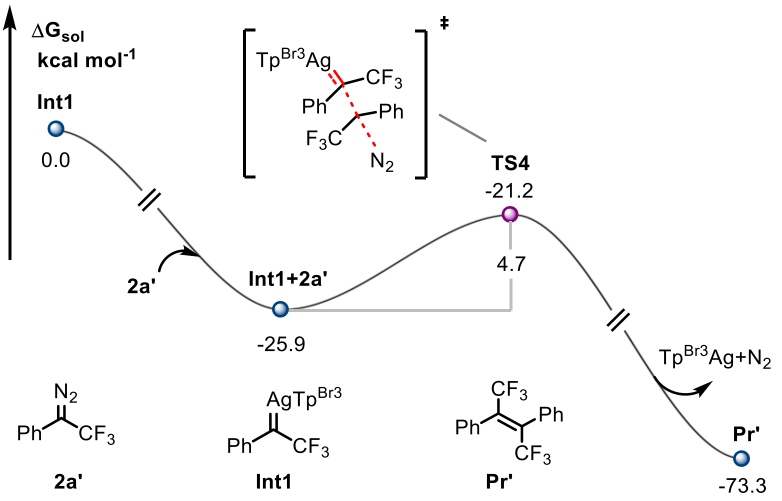

Supplementary Fig. 2b Gibbs free energy profile (in kcal mol^{-1}) of the diazo self-coupling

DFT Calculations

Theoretical methodology:

All DFT calculations described in this work were carried out with Gaussian16 suite of programs[10]. All geometry optimizations and single point calculations were presented by using the B3LYP functional[11,12] and GD3BJ empirical dispersion[13] by using the PCM solvent model[14] for dichloromethane. The Br and Ag atoms were represented with the effective core potential SDD basis set[15,16] and all the other atoms B, C, H, N, O, and F were described with 6-31G(d,p) basis set[17-19].

Intrinsic Reaction Paths (IRPs)[20,21] were traced from the various transition structures to obtain the connected intermediates. The nature of the local minima was established with analytical frequency calculations and geometry optimizations were computed without any symmetry constraints. 3D structures of optimized geometries were generated using CYLview visualization software[22].

5 Characterization data for the products

Note: The stereoselectivity ratio in this part represents the purified value, while the value in the text (Table 1 and Figure 2, 3) is directly measured by NMR for the reaction system.

(2-benzyl-1-(trifluoromethyl)cyclopropyl)benzene (3)

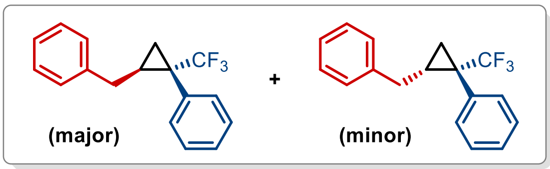

According to **Method C**:

N-triftosylhydrazone derived from 2,2,2-trifluoro-1-phenylethan-1-one (237.6 mg, 0.6 mmol), 2-benzyloxirane (20.1 mg, 0.15 mmol), NaH (27.0 mg, 0.68 mmol, 60 wt.% dispersion in mineral oil) and $Tp^{Br3}Ag(thf)$ (8.2 mg, 5 mol%) were reacted in dichloromethane (4.0 mL) for 6 h to afford compound **3** as a colorless oil. The product was obtained as a mixture of stereoisomers (10:1) determined by NMR.

Run: 38.1 mg, 92% yield.

Purification: Silica gel column chromatography (petroleum ether as eluent).

Major isomer:

^{1}H NMR (500 MHz, $CDCl_3$) δ 7.47-7.42 (m, 2H), 7.41-7.36 (m, 3H), 7.31 (t, *J* = 7.5 Hz, 2H), 7.23 (t, *J* = 7.5 Hz, 1H), 7.18 (d, *J* = 7.0 Hz, 2H), 2.85-2.76 (m, 1H), 1.98-1.87 (m, 2H), 1.59 (dd, *J* = 8.0, 5.0 Hz, 1H), 1.12-1.07 (m, 1H).

^{13}C NMR (125 MHz, $CDCl_3$) δ 140.2, 132.8, 131.9, 128.42, 128.39, 128.35, 128.2, 126.24 (q, *J* = 272.5 Hz), 126.23, 35.7, 32.8 (q, *J* = 32.5 Hz), 21.3 (q, *J* = 1.3 Hz), 15.3 (q, *J* = 2.5 Hz).

^{19}F NMR (470 MHz, $CDCl_3$) δ -69.34 (s).

IR (Film): 3030, 1498, 1346, 1164, 1140, 720, 700 cm^{-1}.

HRMS (ESI^+) m/z calcd for $C_{17}H_{14}F_3$ $[M-H]^-$ 275.1053, found 275.1023.

1-chloro-4-(2-methyl-1-(trifluoromethyl)cyclopropyl)benzene (4)

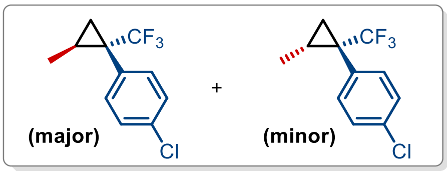

According to **Method C**:

N-triftosylhydrazone derived from 1-(4-chlorophenyl)-2,2,2-trifluoroethan-1-one (258.0 mg, 0.6 mmol), propylene oxide (8.7 mg, 0.15 mmol), NaH (27.0 mg, 0.68 mmol, 60 wt.% dispersion in mineral oil) and $Tp^{Br3}Ag(thf)$ (16.4 mg, 10 mol%) were reacted in dichloromethane (4.0 mL) for 10 h to afford compound **4** as a colorless oil. The product was obtained as a mixture of stereoisomers (10:1) determined by NMR.

Run: 30.9 mg, 88% yield.

Purification: Silica gel column chromatography (petroleum ether as eluent).

Major isomer:

^{1}H NMR (500 MHz, $CDCl_3$) δ 7.36-7.29 (m, 4H), 1.66-1.56 (m, 1H), 1.48 (dd, *J* = 9.0, 5.0 Hz, 1H), 0.85 (d, *J* = 6.0 Hz, 3H), 0.80-0.76 (m, 1H).

^{13}C NMR (150 MHz, $CDCl_3$) δ 134.3, 133.4, 131.5, 128.6, 126.2 (q, *J* = 271.5 Hz), 31.8 (q, *J* = 33.0 Hz), 16.2 (q, *J* = 3.0 Hz), 15.2 (q, *J* = 1.5 Hz), 14.8.

^{19}F NMR (564 MHz, $CDCl_3$) δ -69.44 (s).

IR (Film): 2927, 1495, 1295, 1158, 1136, 1093, 823, 744 cm^{-1}.

HRMS (ESI^+) m/z calcd for $C_{11}H_9ClF_3$ $[M-H]^-$ 233.0350, found 233.0344.

1-(2-butyl-1-(trifluoromethyl)cyclopropyl)-4-chlorobenzene (5)

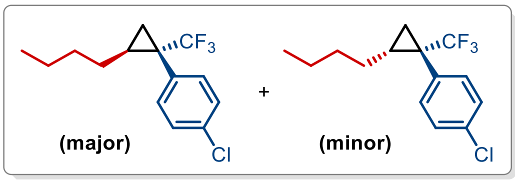

According to **Method C**:

N-triftosylhydrazone derived from 1-(4-chlorophenyl)-2,2,2-trifluoroethan-1-one (258.0 mg, 0.6 mmol), 1,2-epoxyhexane (15.0 mg, 0.15 mmol), NaH (27.0 mg, 0.68 mmol, 60 wt.% dispersion in mineral oil) and $Tp^{Br3}Ag(thf)$ (16.4 mg, 10 mol%) were reacted in dichloromethane (4.0 mL) for 10 h to afford compound **5** as a colorless oil. The product was obtained as a mixture of stereoisomers (10:1) determined by NMR.

Run: 39.3 mg, 95% yield.

Purification: Silica gel column chromatography (petroleum ether as eluent).

Major isomer:

^{1}H NMR (500 MHz, $CDCl_3$) δ 7.35-7.29 (m, 4H), 1.54-1.50 (m, 1H), 1.48-1.40 (m, 2H), 1.39-1.32 (m, 2H), 1.28-1.23 (m, 3H), 0.84 (t, J = 7.5 Hz, 3H), 0.55-0.45 (m, 1H).

^{13}C NMR (150 MHz, $CDCl_3$) δ 134.3, 133.2, 131.8, 128.5, 126.2 (q, J = 271.5 Hz), 31.9 (q, J = 31.5 Hz), 31.2, 29.5, 22.4, 21.0, 15.2, 13.9.

^{19}F NMR (564 MHz, $CDCl_3$) δ -69.42 (s).

IR (Film): 2959, 2931, 1494, 1292, 1193, 1155, 1136, 1017, 827 cm^{-1}.

HRMS (ESI^+) m/z calcd for $C_{14}H_{17}ClF_3$ $[M+H]^+$ 277.0965, found 277.0962.

1-chloro-4-(2-decyl-1-(trifluoromethyl)cyclopropyl)benzene (6)

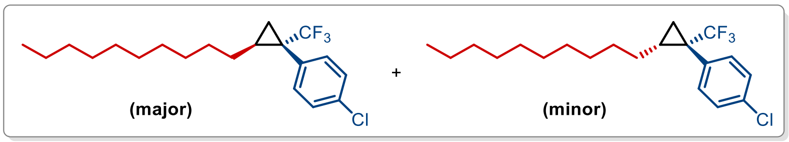

According to **Method C**:

N-triftosylhydrazone derived from 1-(4-chlorophenyl)-2,2,2-trifluoroethan-1-one (258.0 mg, 0.6 mmol), 1,2-epoxydodecane (27.6 mg, 0.15 mmol), NaH (27.0 mg, 0.68 mmol, 60 wt.% dispersion in mineral oil) and $Tp^{Br3}Ag(thf)$ (16.4 mg, 10 mol%) were reacted in dichloromethane (4.0 mL) for 10 h to afford compound **6** as a colorless oil. The product was obtained as a mixture of stereoisomers (12:1) determined by NMR.

Run: 48.6 mg, 90% yield.

Purification: Silica gel column chromatography (petroleum ether as eluent).

Major isomer:

^{1}H NMR (500 MHz, $CDCl_3$) δ 7.36-7.26 (m, 4H), 1.55-1.49 (m, 1H), 1.49-1.17 (m, 19H), 0.87 (t, *J* = 7.0 Hz, 3H), 0.56-0.43 (m, 1H).

^{13}C NMR (150 MHz, $CDCl_3$) δ 134.3, 133.2, 131.8, 128.5, 126.2 (q, *J* = 270.6 Hz), 31.90, 31.86 (q, *J* = 32.3 Hz), 29.9, 29.6, 29.56, 29.55, 29.52, 29.3, 29.1, 22.7, 21.1, 15.1, 14.1.

^{19}F NMR (564 MHz, $CDCl_3$) δ -69.41 (s).

IR (Film): 2927, 2856, 1495, 1307, 1162, 1136, 1094, 1016, 821, 744 cm^{-1}.

HRMS (ESI^+) m/z calcd for $C_{20}H_{29}ClF_3$ $[M+H]^+$ 361.1904, found 361.1942.

1-(2-benzyl-1-(trifluoromethyl)cyclopropyl)-4-chlorobenzene (7)

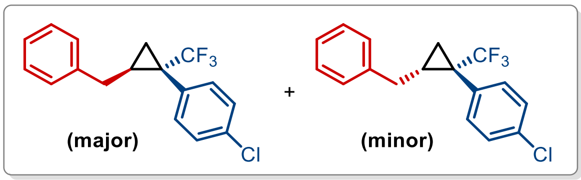

According to **Method C**:

N-triftosylhydrazone derived from 1-(4-chlorophenyl)-2,2,2-trifluoroethan-1-one (258.0 mg, 0.6 mmol), 2-benzyloxirane (20.1 mg, 0.15 mmol), NaH (27.0 mg, 0.68 mmol, 60 wt.% dispersion in mineral oil) and $Tp^{Br3}Ag(thf)$ (8.2 mg, 5 mol%) were reacted in dichloromethane (4.0 mL) for 6 h to afford compound **7** as a colorless oil. The product was obtained as a mixture of stereoisomers (15:1) determined by NMR.

Run: 42.8 mg, 92% yield.

Purification: Silica gel column chromatography (petroleum ether as eluent).

Major isomer:

^{1}H NMR (500 MHz, $CDCl_3$) δ 7.33 (s, 4H), 7.29 (t, *J* = 7.5 Hz, 2H), 7.21 (t, *J* = 7.5 Hz, 1H), 7.15 (d, *J* = 7.0 Hz, 2H), 2.76-2.66 (m, 1H), 1.99-1.85 (m, 2H), 1.58 (dd, *J* = 8.0, 5.5 Hz, 1H), 1.06-1.01 (m, 1H).

^{13}C NMR (150 MHz, $CDCl_3$) δ 139.8, 134.6, 133.2, 131.3, 128.7, 128.5, 128.1, 126.4, 126.0 (q, *J* = 271.5 Hz), 35.6, 32.3 (q, *J* = 31.5 Hz), 21.3, 15.4.

^{19}F NMR (564 MHz, $CDCl_3$) δ -69.45 (s).

IR (Film): 3030, 2924, 1495, 1347, 1165, 1140, 822, 744, 723 cm^{-1}.

HRMS (ESI$^+$) m/z calcd for $C_{17}H_{15}ClF_3$ $[M+H]^+$ 311.0809, found 311.0822.

1-methoxy-4-(2-phenethyl-1-(trifluoromethyl)cyclopropyl)benzene (8)

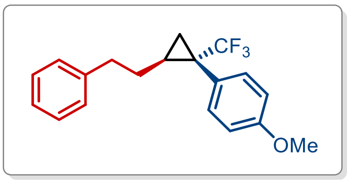

According to **Method C**:

N-triftosylhydrazone derived from 2,2,2-trifluoro-1-(4-methoxyphenyl)ethan-1-one (255.6 mg, 0.6 mmol), 2-phenethyloxirane (22.2 mg, 0.15 mmol), NaH (27.0 mg, 0.68 mmol, 60 wt.% dispersion in mineral oil) and Tp^{Br3}Ag(thf) (8.2 mg, 5 mol%) were reacted in dichloromethane (4.0 mL) for 6 h to afford compound **8** as a colorless oil.

Run: 41.8 mg, 87% yield.

Purification: Silica gel column chromatography (petroleum ether as eluent).

^{1}H NMR (500 MHz, $CDCl_3$) δ 7.29 (d, *J* = 8.5 Hz, 2H), 7.24 (t, *J* = 7.5 Hz, 2H), 7.16 (t, *J* = 7.0 Hz, 1H), 7.09 (d, *J* = 7.5 Hz, 2H), 6.86 (d, *J* = 8.5 Hz, 2H), 3.79 (s, 3H), 2.77-2.59 (m, 2H), 1.79-1.68 (m, 1H), 1.59-1.49 (m, 1H), 1.43 (dd, *J* = 9.5, 5.0 Hz, 1H), 0.97-0.85 (m, 1H), 0.79 (t, *J* = 5.0 Hz, 1H).

^{13}C NMR (150 MHz, $CDCl_3$) δ 159.5, 141.6, 132.9, 128.4, 128.3, 126.4 (q, *J* = 271.5 Hz), 125.9, 124.9, 113.7, 55.2, 35.4, 31.83, 31.77 (q, *J* = 33.0 Hz), 20.6, 15.0.

^{19}F NMR (564 MHz, $CDCl_3$) δ -69.60 (s).

IR (Film): 2937, 1516, 1295, 1251, 1160, 1134, 913, 827, 746, 699 cm^{-1}.

HRMS (ESI^+) m/z calcd for $C_{19}H_{20}F_3O$ $[M+H]^+$ 321.1461, found 321.1428.

1-(2-((benzyloxy)methyl)-1-(trifluoromethyl)cyclopropyl)-4-chlorobenzene (9)

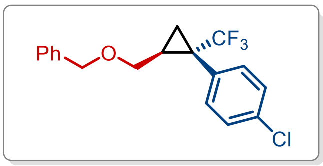

According to **Method C**:

N-triftosylhydrazone derived from 1-(4-chlorophenyl)-2,2,2-trifluoroethan-1-one (258.0 mg, 0.6 mmol), 2-((benzyloxy)methyl)oxirane (24.6 mg, 0.15 mmol), NaH (27.0 mg, 0.68 mmol, 60 wt.% dispersion in mineral oil) and $Tp^{Br3}Ag(thf)$ (16.4 mg, 10 mol%) were reacted in dichloromethane (4.0 mL) for 10 h to afford compound **9** as a colorless oil.

Run: 35.7 mg, 70% yield.

Purification: Silica gel column chromatography (petroleum ether as eluent).

^{1}H NMR (500 MHz, $CDCl_3$) δ 7.50-7.44 (m, 2H), 7.37-7.30 (m, 5H), 7.29-7.25 (m, 2H), 4.35 (ABq, *J* = 12.0 Hz, 2H), 3.22 (dd, *J* = 10.0, 6.0 Hz, 1H), 3.06 (dd, *J* = 10.0, 8.0 Hz, 1H), 2.01-1.89 (m, 1H), 1.53 (dd, *J* = 9.5, 5.5 Hz, 1H), 1.02 (t, *J* = 5.0 Hz, 1H).

^{13}C NMR (150 MHz, $CDCl_3$) δ 138.0, 132.3, 132.0, 128.5, 128.4, 128.3, 127.69, 127.65, 126.0 (q, *J* = 271.5 Hz), 73.0, 69.6, 32.8 (q, *J* = 33.0 Hz), 20.4, 12.8.

^{19}F NMR (564 MHz, $CDCl_3$) δ -69.96 (s).

IR (Film): 2925, 2857, 1346, 1301, 1153, 1134, 1098, 736, 699 cm^{-1}.

HRMS (ESI^+) m/z calcd for $C_{18}H_{15}ClF_3$ $[M-H]^-$ 339.0769, found 339.0722.

1-chloro-4-(2-((4-methoxyphenoxy)methyl)-1-(trifluoromethyl)cyclopropyl)benzene (10)

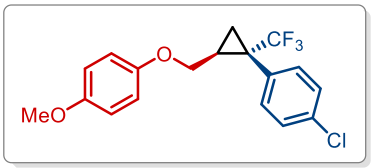

According to **Method C**:

N-triftosylhydrazone derived from 1-(4-chlorophenyl)-2,2,2-trifluoroethan-1-one (258.0 mg, 0.6 mmol), 2-((4-methoxyphenoxy)methyl)oxirane (27.0 mg, 0.15 mmol), NaH (27.0 mg, 0.68 mmol, 60 wt.% dispersion in mineral oil) and $Tp^{Br3}Ag(thf)$ (16.4 mg, 10 mol%) were reacted in dichloromethane (4.0 mL) for 6 h to afford compound **10** as a colorless oil.

Run: 39.5 mg, 74% yield.

Purification: Silica gel column chromatography (1% EtOAc in petroleum ether).

^{1}H NMR (600 MHz, $CDCl_3$) δ 7.44 (d, J = 8.4 Hz, 2H), 7.29 (d, J = 8.4 Hz, 2H), 6.78 (d, J = 9.0 Hz, 2H), 6.71 (d, J = 9.0 Hz, 2H), 3.79-3.76 (m, 1H), 3.75 (s, 3H), 3.37 (t, J = 9.0 Hz, 1H), 2.14-2.06 (m, 1H), 1.61 (dd, J = 9.6, 6.0 Hz, 1H), 1.07 (t, J = 6.0 Hz, 1H).

^{13}C NMR (150 MHz, $CDCl_3$) δ 154.2, 152.5, 134.8, 133.4, 130.5, 128.7, 125.7 (q, J = 271.5 Hz), 115.7, 114.6, 67.8, 55.7, 32.5 (q, J = 33.0 Hz), 20.2 (q, J = 3.0 Hz), 12.7 (q, J = 1.5 Hz).

^{19}F NMR (564 MHz, $CDCl_3$) δ -70.14 (s).

HRMS (ESI^+) m/z calcd for $C_{18}H_{17}ClF_3O_2$ $[M+H]^+$ 357.0864, found 357.0865.

1-bromo-4-((2-(4-chlorophenyl)-2-(trifluoromethyl)cyclopropyl)methoxy)benzene (11)

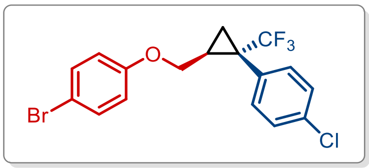

According to **Method C**:

N-triftosylhydrazone derived from 1-(4-chlorophenyl)-2,2,2-trifluoroethan-1-one (258.0 mg, 0.6 mmol), 2-((4-bromophenoxy)methyl)oxirane (34.2 mg, 0.15 mmol), NaH (27.0 mg, 0.68 mmol, 60 wt.% dispersion in mineral oil) and $Tp^{Br3}Ag(thf)$ (16.4 mg, 10 mol%) were reacted in dichloromethane (4.0 mL) for 6 h to afford compound **11** as a colorless oil.

Run: 43.6 mg, 72% yield.

Purification: Silica gel column chromatography (petroleum ether as eluent).

^{1}H NMR (500 MHz, $CDCl_3$) δ 7.41 (d, J = 8.5 Hz, 2H), 7.32 (d, J = 8.5 Hz, 2H), 7.28 (d, J = 8.5 Hz, 2H), 6.65 (d, J = 9.0 Hz, 2H), 3.86-3.75 (m, 1H), 3.36 (t, J = 9.0 Hz, 1H), 2.17-2.06 (m, 1H), 1.67-1.59 (m, 1H), 1.09 (t, J = 6.0 Hz, 1H).

^{13}C NMR (150 MHz, $CDCl_3$) δ 157.4, 134.9, 133.3, 132.3, 130.3, 128.8, 125.6 (q, J = 273.0 Hz), 116.3, 113.4, 67.2, 32.6 (q, J = 33.0 Hz), 19.9 (q, J = 3.0 Hz), 12.6 (q, J = 1.5 Hz).

^{19}F NMR (564 MHz, $CDCl_3$) δ -70.17 (s).

HRMS (ESI$^+$) m/z calcd for $C_{17}H_{14}BrClF_3O$ $[M+H]^+$ 404.9863, found 404.9865.

1-chloro-4-(2-((4-(2-phenylpropan-2-yl)phenoxy)methyl)-1-(trifluoromethyl)cyclopropyl)benzene (12)

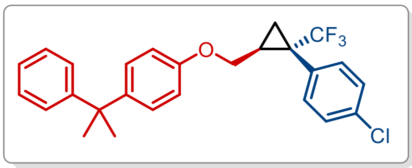

According to **Method C**:

N-triftosylhydrazone derived from 1-(4-chlorophenyl)-2,2,2-trifluoroethan-1-one (258.0 mg, 0.6 mmol), 2-((4-(2-phenylpropan-2-yl)phenoxy)methyl)oxirane (40.2 mg, 0.15 mmol), NaH (27.0 mg, 0.68 mmol, 60 wt.% dispersion in mineral oil) and $Tp^{Br3}Ag(thf)$ (16.4 mg, 10 mol%) were reacted in dichloromethane (4.0 mL) for 6 h to afford compound **12** as a colorless oil.

Run: 52.0 mg, 78% yield.

Purification: Silica gel column chromatography (petroleum ether as eluent).

^{1}H NMR (600 MHz, $CDCl_3$) δ 7.42 (d, *J* = 8.4 Hz, 2H), 7.27 (d, *J* = 8.4 Hz, 2H), 7.24 (d, *J* = 7.2 Hz, 2H), 7.20 (d, *J* = 7.8 Hz, 2H), 7.15 (t, *J* = 7.2 Hz, 1H), 7.09 (d, *J* = 8.4 Hz, 2H), 6.66 (d, *J* = 8.4 Hz, 2H), 3.76 (dd, *J* = 9.6, 5.4 Hz, 1H), 3.40 (t, *J* = 9.0 Hz, 1H), 2.14-2.06 (m, 1H), 1.64 (s, 6H), 1.68-1.58 (m, 1H), 1.07 (t, *J* = 5.4 Hz, 1H).

^{13}C NMR (125 MHz, $CDCl_3$) δ 156.2, 150.8, 143.4, 134.8, 133.3, 130.5, 128.7, 128.0, 127.8, 126.7, 125.6 (q, *J* = 271.3 Hz), 125.5, 113.9, 67.0, 42.3, 32.5 (q, *J* = 32.5 Hz), 30.8, 20.1 (q, *J* = 1.3 Hz), 12.7 (q, *J* = 2.5 Hz).

^{19}F NMR (470 MHz, $CDCl_3$) δ -70.09 (s).

HRMS (ESI^+) m/z calcd for $C_{26}H_{25}ClF_3O$ $[M+H]^+$ 445.1541, found 445.1562.

9-(2-(4-chlorophenyl)-2-(trifluoromethyl)cyclopropyl)nonyl thiophene-2-carboxylate (13)

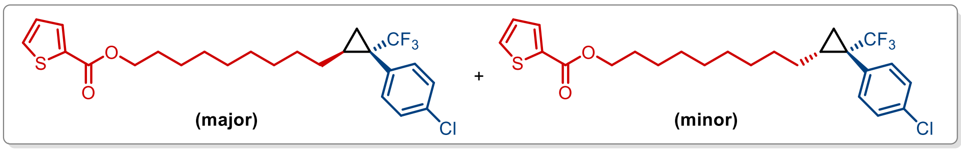

According to **Method C**:

N-triftosylhydrazone derived from 1-(4-chlorophenyl)-2,2,2-trifluoroethan-1-one (258.0 mg, 0.6 mmol), 9-(oxiran-2-yl)nonyl thiophene-2-carboxylate (44.4 mg, 0.15 mmol), NaH (27.0 mg, 0.68 mmol, 60 wt.% dispersion in mineral oil) and $Tp^{Br3}Ag(thf)$ (16.4 mg, 10 mol%) were reacted in dichloromethane (4.0 mL) for 6 h to afford compound **13** as a colorless oil. The product was obtained as a mixture of stereoisomers (9:1) determined by NMR.

Run: 68.0 mg, 96% yield.

Purification: Silica gel column chromatography (2% EtOAc in petroleum ether).

Major isomer:

^{1}H NMR (500 MHz, $CDCl_3$) δ 7.79 (d, *J* = 3.5 Hz, 1H), 7.53 (d, *J* = 5.0 Hz, 1H), 7.36-7.27 (m, 4H), 7.08 (t, *J* = 4.5 Hz, 1H), 4.27 (t, *J* = 7.0 Hz, 2H), 1.76-1.68 (m, 2H), 1.55-1.48 (m, 1H), 1.45 (dd, *J* = 9.0, 5.0 Hz, 1H), 1.43-1.17 (m, 13H), 0.80 (t, *J* = 5.0 Hz, 1H), 0.56-0.42 (m, 1H).

^{13}C NMR (125 MHz, $CDCl_3$) δ 162.3, 134.2, 134.1, 133.19, 133.15, 132.1, 131.7, 128.5, 127.7, 126.1 (q, *J* = 273.5 Hz), 65.2, 31.8 (q, *J* = 32.4 Hz), 29.8, 29.4, 29.3, 29.2, 29.1, 29.0, 28.6, 25.9, 21.0 (q, *J* = 1.6 Hz), 15.1 (q, *J* = 2.3 Hz).

^{19}F NMR (470 MHz, $CDCl_3$) δ -69.36 (s).

HRMS (ESI^+) m/z calcd for $C_{24}H_{29}ClF_3O_2S$ $[M+H]^+$ 473.1523, found 473.1531.

1-chloro-4-(2-(9-chlorononyl)-1-(trifluoromethyl)cyclopropyl)benzene (14)

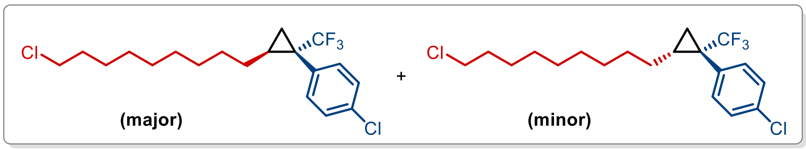

According to **Method C**:

N-triftosylhydrazone derived from 1-(4-chlorophenyl)-2,2,2-trifluoroethan-1-one (258.0 mg, 0.6 mmol), 2-(9-chlorononyl)oxirane (30.6 mg, 0.15 mmol), NaH (27.0 mg, 0.68 mmol, 60 wt.% dispersion in mineral oil) and $Tp^{Br3}Ag(thf)$ (16.4 mg, 10 mol%) were reacted in dichloromethane (4.0 mL) for 10 h to afford compound **14** as a colorless oil. The product was obtained as a mixture of stereoisomers (10:1) determined by NMR.

Run: 48.5 mg, 85% yield.

Purification: Silica gel column chromatography (petroleum ether as eluent).

Major isomer:

^{1}H NMR (600 MHz, $CDCl_3$) δ 7.36-7.27 (m, 4H), 3.52 (t, *J* = 7.2 Hz, 2H), 1.79-1.71 (m, 2H), 1.54-1.49 (m, 1H), 1.46 (dd, *J* = 9.0, 5.4 Hz, 1H), 1.44-1.17 (m, 13H), 0.81 (t, *J* = 5.4 Hz, 1H), 0.57-0.45 (m, 1H).

^{13}C NMR (150 MHz, $CDCl_3$) δ 134.3, 133.2, 132.3, 131.8, 128.52, 128.49, 126.1 (q, *J* = 271.5 Hz), 45.1, 32.6, 31.9 (q, *J* = 31.5 Hz), 29.8, 29.4, 29.3, 29.3, 29.0, 28.8, 26.8, 21.0 (q, *J* = 1.5 Hz), 15.1.

^{19}F NMR (564 MHz, $CDCl_3$) δ -69.39 (s).

HRMS (ESI^+) m/z calcd for $C_{19}H_{27}ClF_3$ $[M+H]^+$ 347.1748, found 347.1747.

9-(2-(4-chlorophenyl)-2-(trifluoromethyl)cyclopropyl)nonyl 4-methylbenzenesulfonate (15)

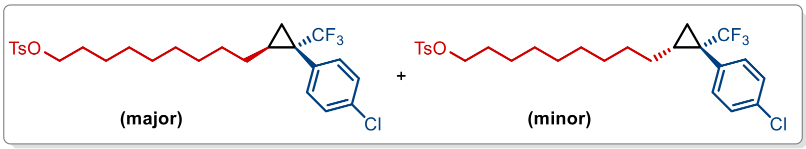

According to **Method C**:

N-triftosylhydrazone derived from 1-(4-chlorophenyl)-2,2,2-trifluoroethan-1-one (258.0 mg, 0.6 mmol), 9-(oxiran-2-yl)nonyl 4-methylbenzenesulfonate (51.0 mg, 0.15 mmol), NaH (27.0 mg, 0.68 mmol, 60 wt.% dispersion in mineral oil) and $Tp^{Br3}Ag(thf)$ (16.4 mg, 10 mol%) were reacted in dichloromethane (4.0 mL) for 10 h to afford compound **15** as a colorless oil. The product was obtained as a mixture of stereoisomers (9:1) determined by NMR.

Run: 69.7 mg, 90% yield.

Purification: Silica gel column chromatography (1% EtOAc in petroleum ether).

Major isomer:

^{1}H NMR (500 MHz, $CDCl_3$) δ 7.77 (d, *J* = 8.0 Hz, 2H), 7.32 (d, *J* = 8.0 Hz, 2H), 7.31 (s, 4H), 4.00 (t, *J* = 6.5 Hz, 2H), 2.42 (s, 3H), 1.65-1.57 (m, 2H), 1.55-1.48 (m, 1H), 1.45 (dd, *J* = 9.0, 5.0 Hz, 1H), 1.43-1.10 (m, 13H), 0.80 (t, *J* = 5.0 Hz, 1H), 0.54-0.43 (m, 1H).

^{13}C NMR (125 MHz, $CDCl_3$) δ 144.6, 134.2, 133.1, 132.3, 131.7, 129.7, 128.5, 128.4, 127.8, 126.1 (q, *J* = 271.3 Hz), 70.6, 31.8 (q, *J* = 32.5 Hz), 29.8, 29.3, 29.2, 28.9, 28.8, 28.7, 25.2, 21.5, 21.0, 15.1 (q, *J* = 2.5 Hz).

^{19}F NMR (470 MHz, $CDCl_3$) δ -69.35 (s).

HRMS (ESI^+) m/z calcd for $C_{26}H_{33}ClF_3O_3S$ $[M+H]^+$ 517.1786, found 517.1797.

4,4'-(3-phenyl-2,2'-bis(trifluoromethyl)-[1,1'-bi(cyclopropane)]-2,2'-diyl)bis(chlorobenzene) (16)

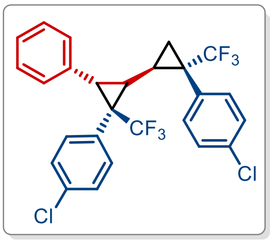

According to **Method D**:

N-triftosylhydrazone derived from 1-(4-chlorophenyl)-2,2,2-trifluoroethan-1-one (161.2 mg, 0.38 mmol), 2-styryloxirane (21.9 mg, 0.15 mmol), NaH (15.2 mg, 0.38 mmol, 60 wt.% dispersion in mineral oil) and $Tp^{Br3}Ag(thf)$ (8.2 mg, 5 mol%) were reacted in dichloromethane (4.0 mL) for 2 h to afford compound **16** as a colorless oil.

Run: 32.4 mg, 42% yield.

Purification: Silica gel column chromatography (petroleum ether as eluent).

^{1}H NMR (500 MHz, $CDCl_3$) δ 7.28 (d, *J* = 8.0 Hz, 2H), 7.17-7.11 (m, 3H), 7.08 (t, *J* = 7.5 Hz, 2H), 7.02 (d, *J* = 8.5 Hz, 2H), 6.70 (d, *J* = 8.0 Hz, 2H), 6.43 (d, *J* = 7.5 Hz, 2H), 3.01 (d, *J* = 7.5 Hz, 1H), 1.94-1.86 (m, 1H), 1.84 (dd, *J* = 9.5, 6.0 Hz, 1H), 1.29 (t, *J* = 5.5 Hz, 1H), 1.00 (t, *J* = 8.0 Hz, 1H).

^{13}C NMR (150 MHz, $CDCl_3$) δ 135.0, 134.4, 134.0, 133.6, 130.5, 130.4, 128.9, 128.2, 128.0, 127.8, 127.1, 125.5 (q, *J* = 271.5 Hz), 39.5 (q, *J* = 30.0 Hz), 32.8 (q, *J* = 33.0 Hz), 32.3 (q, *J* = 3.0 Hz), 30.0, 19.1, 15.6.

^{19}F NMR (564 MHz, $CDCl_3$) δ -63.44 (s), -69.77 (s).

IR (Film): 1495, 1355, 1311, 1255, 1149, 1093, 823, 730, 696 cm^{-1}.

HRMS (ESI^+) m/z calcd for $C_{26}H_{17}Cl_2F_3$ $[M-H]^-$ 513.0617, found 513.0614.

1-chloro-4-(2-(phenylethynyl)-1-(trifluoromethyl)cyclopropyl)benzene (17)

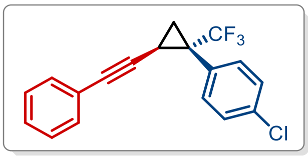

According to **Method D**:

N-triftosylhydrazone derived from 1-(4-chlorophenyl)-2,2,2-trifluoroethan-1-one (161.2 mg, 0.38 mmol), 2-(phenylethynyl)oxirane (21.6 mg, 0.15 mmol), NaH (15.2 mg, 0.38 mmol, 60 wt.% dispersion in mineral oil) and $Tp^{Br3}Ag(thf)$ (8.2 mg, 5 mol%) were reacted in dichloromethane (4.0 mL) for 2 h to afford compound **17** as a colorless oil.

Run: 25.9 mg, 54% yield.

Purification: Silica gel column chromatography (petroleum ether as eluent).

^{1}H NMR (500 MHz, $CDCl_3$) δ 7.48 (d, *J* = 8.5 Hz, 2H), 7.38 (d, *J* = 8.5 Hz, 2H), 7.25-7.18 (m, 3H), 7.12-7.07 (m, 2H), 2.41 (dd, *J* = 9.5, 6.5 Hz, 1H), 1.85 (dd, *J* = 9.5, 5.5 Hz, 1H), 1.45-1.41 (m, 1H).

^{13}C NMR (150 MHz, $CDCl_3$) δ 134.9, 133.5, 131.3, 130.6, 128.4, 128.2, 128.1, 125.1 (q, *J* = 273.0 Hz), 122.7, 86.4, 82.9, 34.1 (q, *J* = 31.5 Hz), 18.3 (q, *J* = 3.0 Hz), 11.8 (q, *J* = 3.0 Hz).

^{19}F NMR (564 MHz, $CDCl_3$) δ -70.08 (s).

IR (Film): 1599, 1494, 1295, 1157, 1092, 846, 756 cm^{-1}.

HRMS (ESI^+) m/z calcd for $C_{18}H_{11}ClF_3$ $[M-H]^-$ 319.0507, found 319.0563.

1-chloro-4-(2-(hex-5-en-1-yl)-1-(trifluoromethyl)cyclopropyl)benzene (18)

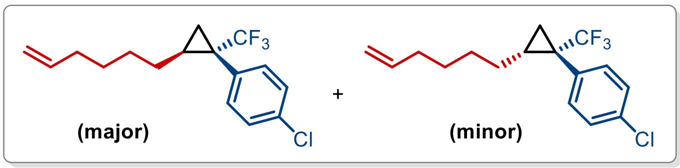

According to **Method C**:

N-triftosylhydrazone derived from 1-(4-chlorophenyl)-2,2,2-trifluoroethan-1-one (258.0 mg, 0.6 mmol), 1,4-di(oxiran-2-yl)butane (21.3 mg, 0.15 mmol), NaH (27.0 mg, 0.68 mmol, 60 wt.% dispersion in mineral oil) and $Tp^{Br3}Ag(thf)$ (16.4 mg, 10 mol%) were reacted in dichloromethane (4.0 mL) for 10 h to afford compound **18** as a colorless oil. The product was obtained as a mixture of stereoisomers (12:1) determined by NMR.

Run: 36.3 mg, 80% yield.

Purification: Silica gel column chromatography (petroleum ether as eluent).

Major isomer:

^{1}H NMR (500 MHz, $CDCl_3$) δ 7.35-7.27 (m, 4H), 5.80-5.69 (m, 1H), 4.99-4.89 (m, 2H), 2.01-1.95 (q, *J* = 6.5 Hz, 2H), 1.54-1.49 (m, 1H), 1.49-1.29 (m, 6H), 0.83-0.78 (t, *J* = 4.5 Hz, 1H), 0.57-0.44 (m, 1H).

^{13}C NMR (150 MHz, $CDCl_3$) δ 138.7, 134.3, 133.2, 131.7, 128.5, 126.1 (q, *J* = 271.5 Hz), 114.5, 33.6, 31.9 (q, *J* = 33.0 Hz), 29.7, 28.50, 28.47, 21.0, 15.1.

^{19}F NMR (564 MHz, $CDCl_3$) δ -69.42 (s).

IR (Film): 3079, 2932, 2860, 1495, 1162, 1134, 1093, 912, 821, 744 cm^{-1}.

HRMS (ESI^+) m/z calcd for $C_{16}H_{17}ClF_3$ $[M-H]^-$ 301.0976, found 301.0920.

1,4-bis(2-phenyl-2-(trifluoromethyl)cyclopropyl)butane (19)

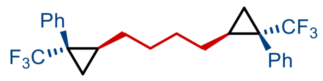

(major)

According to **Method C**:

N-triftosylhydrazone derived from 2,2,2-trifluoro-1-phenylethan-1-one (356.4 mg, 0.9 mmol), 1,4-di(oxiran-2-yl)butane (21.3 mg, 0.15 mmol), NaH (36.0 mg, 0.9 mmol, 60 wt.% dispersion in mineral oil) and $Tp^{Br3}Ag(thf)$ (16.4 mg, 10 mol%) were reacted in dichloromethane (6.0 mL) for 10 h to afford compound **19** as a colorless oil. The product was obtained as a mixture of stereoisomers (8:1) determined by NMR.

Run: 47.3 mg, 74% yield.

Purification: Silica gel column chromatography (petroleum ether as eluent).

Major isomer:

^{1}H NMR (500 MHz, $CDCl_3$) δ 7.38-7.30 (m, 10H), 1.51-1.40 (m, 5H), 1.39-1.26 (m, 5H), 0.93-0.72 (m, 2H), 0.65-0.37 (m, 2H).

^{13}C NMR (150 MHz, $CDCl_3$) δ 133.1, 131.9, 131.8, 131.0, 128.3, 128.24, 128.17, 128.0, 126.4 (q, J = 273.0 Hz), 32.4 (q, J = 31.5 Hz), 29.8, 29.7, 28.8, 28.7, 20.8, 14.9.

^{19}F NMR (564 MHz, $CDCl_3$) δ -69.34 (s), -69.35 (s).

HRMS (ESI^+) m/z calcd for $C_{24}H_{25}F_6$ $[M+H]^+$ 427.1855, found 427.1870.

1-chloro-4-(2-phenyl-1-(trifluoromethyl)cyclopropyl)benzene (20)[23]

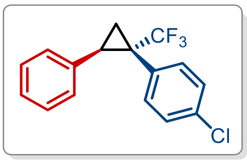

According to **Method D**:

N-triftosylhydrazone derived from 1-(4-chlorophenyl)-2,2,2-trifluoroethan-1-one (161.2 mg, 0.38 mmol), styrene oxide (18.0 mg, 0.15 mmol), NaH (15.2 mg, 0.38 mmol, 60 wt.% dispersion in mineral oil) and $Tp^{Br3}Ag(thf)$ (8.2 mg, 5 mol%) were reacted in dichloromethane (4.0 mL) for 30 min to afford compound **20** as a colorless oil.

Run: 34.6 mg, 78% yield.

Purification: Silica gel column chromatography (petroleum ether as eluent).

^{1}H NMR (500 MHz, $CDCl_3$) δ 7.13 (d, *J* = 8.5 Hz, 2H), 7.12-7.09 (m, 3H), 7.06 (d, *J* = 8.5 Hz, 2H), 6.81-6.74 (m, 2H), 2.85 (dd, *J* = 9.5, 7.0 Hz, 1H), 1.87 (dd, *J* = 9.5, 6.0 Hz, 1H), 1.65 (t, *J* = 7.0 Hz, 1H).

^{13}C NMR (125 MHz, $CDCl_3$) δ 135.1, 134.3, 133.8, 130.1, 128.3, 128.0, 127.9, 126.7, 125.6 (q, *J* = 271.3 Hz), 35.1 (q, *J* = 32.5 Hz), 25.7 (q, *J* = 2.5 Hz), 14.5 (q, *J* = 2.5 Hz).

^{19}F NMR (470 MHz, $CDCl_3$) δ -69.85 (s).

HRMS (ESI^+) m/z calcd for $C_{16}H_{13}ClF_3$ $[M+H]^+$ 297.0652, found 297.0655.

1-chloro-4-(2-(p-tolyl)-1-(trifluoromethyl)cyclopropyl)benzene (21)

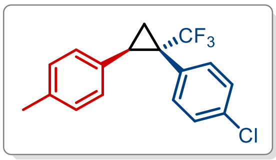

According to **Method D**:

N-triftosylhydrazone derived from 1-(4-chlorophenyl)-2,2,2-trifluoroethan-1-one (161.2 mg, 0.38 mmol), 2-(*p*-tolyl)oxirane (20.1 mg, 0.15 mmol), NaH (15.2 mg, 0.38 mmol, 60 wt.% dispersion in mineral oil) and $Tp^{Br3}Ag(thf)$ (8.2 mg, 5 mol%) were reacted in dichloromethane (4.0 mL) for 30 min to afford compound **21** as a colorless oil.

Run: 34.9 mg, 75% yield.

Purification: Silica gel column chromatography (petroleum ether as eluent).

^{1}H NMR (500 MHz, $CDCl_3$) δ 7.13 (d, *J* = 8.5 Hz, 2H), 7.06 (d, *J* = 8.5 Hz, 2H), 6.91 (d, *J* = 8.0 Hz, 2H), 6.66 (d, *J* = 8.0 Hz, 2H), 2.80 (dd, *J* = 9.0, 7.5 Hz, 1H), 2.22 (s, 3H), 1.84 (dd, *J* = 9.5, 6.0 Hz, 1H), 1.59 (t, *J* = 7.0 Hz, 1H).

^{13}C NMR (150 MHz, $CDCl_3$) δ 136.3, 134.2, 133.8, 132.0, 130.3, 128.8, 128.3, 127.8, 125.7 (q, *J* = 273.0 Hz), 34.9 (q, *J* = 33.0 Hz), 25.4 (q, *J* = 1.5 Hz), 20.9, 14.5 (q, *J* = 1.5 Hz).

^{19}F NMR (564 MHz, $CDCl_3$) δ -69.77 (s).

IR (Film): 2924, 1599, 1519, 1495, 1381, 1320, 1295, 1282, 1153, 1138, 1091, 1054 858, 819, 745, 731 cm^{-1}.

HRMS (ESI^{+}) m/z calcd for $C_{17}H_{13}ClF_3$ $[M-H]^{-}$ 309.0663, found 309.0632.

4-(2-(4-chlorophenyl)-2-(trifluoromethyl)cyclopropyl)-1,1'-biphenyl (22)

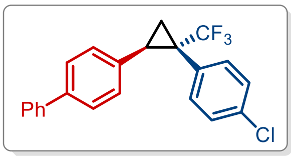

According to **Method D**:

N-triftosylhydrazone derived from 1-(4-chlorophenyl)-2,2,2-trifluoroethan-1-one (161.2 mg, 0.38 mmol), 2-([1,1'-biphenyl]-4-yl)oxirane (29.4 mg, 0.15 mmol), NaH (15.2 mg, 0.38 mmol, 60 wt.% dispersion in mineral oil) and $Tp^{Br3}Ag(thf)$ (8.2 mg, 5 mol%) were reacted in dichloromethane (4.0 mL) for 30 min to afford compound **22** as a white solid (mp: 94-95 °C).

Run: 43.0 mg, 77% yield.

Purification: Silica gel column chromatography (petroleum ether as eluent).

^{1}H NMR (500 MHz, $CDCl_3$) δ 7.52 (d, *J* = 7.5 Hz, 2H), 7.40 (t, *J* = 7.5 Hz, 2H), 7.36 (d, *J* = 8.0 Hz, 2H), 7.31 (t, *J* = 7.5 Hz, 1H), 7.16 (d, *J* = 8.5 Hz, 2H), 7.10 (d, *J* = 8.5 Hz, 2H), 6.84 (d, *J* = 8.0 Hz, 2H), 2.88 (dd, *J* = 9.0, 7.0 Hz, 1H), 1.92 (dd, *J* = 9.5, 6.0 Hz, 1H), 1.67 (t, *J* = 6.0 Hz, 1H).

^{13}C NMR (150 MHz, $CDCl_3$) δ 140.4, 139.5, 134.4, 134.3, 133.8, 130.1, 128.7, 128.4, 128.3, 127.3, 126.9, 126.6, 125.6 (q, *J* = 271.5 Hz), 35.3 (q, *J* = 33.0 Hz), 25.5, 14.9.

^{19}F NMR (564 MHz, $CDCl_3$) δ -69.86 (s).

IR (Film): 1600, 1523, 1494, 1447, 1384, 1322, 1295, 1217, 1152, 1138, 1091 976, 860, 821, 766, 745 cm^{-1}.

HRMS (ESI^+) m/z calcd for $C_{22}H_{17}ClF_3$ $[M+H]^+$ 373.0965, found 373.0939.

4,4'-(1-(trifluoromethyl)cyclopropane-1,2-diyl)bis(chlorobenzene) (23)

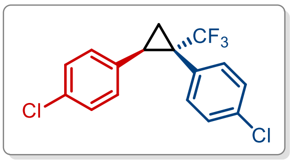

According to **Method D**:

N-triftosylhydrazone derived from 1-(4-chlorophenyl)-2,2,2-trifluoroethan-1-one (161.2 mg, 0.38 mmol), 2-(4-chlorophenyl)oxirane (23.1 mg, 0.15 mmol), NaH (15.2 mg, 0.38 mmol, 60 wt.% dispersion in mineral oil) and $Tp^{Br3}Ag(thf)$ (4.1 mg, 2.5 mol%) were reacted in dichloromethane (4.0 mL) for 30 min to afford compound **23** as a colorless oil.

Run: 39.1 mg, 79% yield.

Purification: Silica gel column chromatography (petroleum ether as eluent).

^{1}H NMR (500 MHz, $CDCl_3$) δ 7.17 (d, J = 8.5 Hz, 2H), 7.09 (d, J = 8.5 Hz, 2H), 7.05 (d, J = 8.5 Hz, 2H), 6.71 (d, J = 8.5 Hz, 2H), 2.81 (dd, J = 9.5, 7.0 Hz, 1H), 1.90 (dd, J = 9.5, 6.0 Hz, 1H), 1.64-1.58 (m, 1H).

^{13}C NMR (125 MHz, $CDCl_3$) δ 134.5, 133.8, 133.7, 132.6, 129.7, 129.1, 128.5, 128.2, 125.5 (q, J = 272.5 Hz), 35.3 (q, J = 32.5 Hz), 25.1 (q, J = 1.3 Hz), 14.8 (q, J = 2.5 Hz).

^{19}F NMR (470 MHz, $CDCl_3$) δ -69.94 (s).

IR (Film): 1496, 1314, 1292, 1153, 1139, 1092, 1015, 821, 745, 729 cm^{-1}.

HRMS (ESI^+) m/z calcd for $C_{16}H_{10}Cl_2F_3$ $[M-H]^+$ 329.0117, found 329.0084.

1-(2-(4-chlorophenyl)-2-(trifluoromethyl)cyclopropyl)-3-methoxybenzene (24)

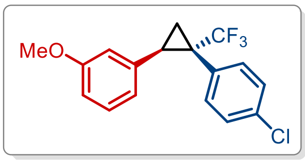

According to **Method D**:

N-triftosylhydrazone derived from 1-(4-chlorophenyl)-2,2,2-trifluoroethan-1-one (161.2 mg, 0.38 mmol), 2-(3-methoxyphenyl)oxirane (22.5 mg, 0.15 mmol), NaH (15.2 mg, 0.38 mmol, 60 wt.% dispersion in mineral oil) and $Tp^{Br3}Ag(thf)$ (8.2 mg, 5 mol%) were reacted in dichloromethane (4.0 mL) for 30 min to afford compound **24** as a colorless oil.

Run: 45.5 mg, 93% yield.

Purification: Silica gel column chromatography (1% EtOAc in petroleum ether).

^{1}H NMR (500 MHz, $CDCl_3$) δ 7.15 (d, *J* = 8.5 Hz, 2H), 7.08 (d, *J* = 8.5 Hz, 2H), 7.02 (t, *J* = 8.0 Hz, 1H), 6.65 (dd, *J* = 8.5, 2.5 Hz, 1H), 6.38 (d, *J* = 8.0 Hz, 1H), 6.30 (t, *J* = 2.5 Hz, 1H), 3.63 (s, 3H), 2.81 (dd, *J* = 9.5, 7.0 Hz, 1H), 1.87 (dd, *J* = 9.5, 6.5 Hz, 1H), 1.65-1.60 (m, 1H).

^{13}C NMR (150 MHz, $CDCl_3$) δ 159.3, 136.8, 134.3, 133.7, 130.2, 129.0, 128.3, 125.6 (q, *J* = 271.5 Hz), 120.4, 113.7, 112.2, 55.0, 35.1 (q, *J* = 33.0 Hz), 25.7 (q, *J* = 3.0 Hz), 14.7 (q, *J* = 1.5 Hz).

^{19}F NMR (564 MHz, $CDCl_3$) δ -69.87 (s).

IR (Film): 2938, 2837, 1601, 1586, 1495, 1307, 1167, 820, 745, 727 cm^{-1}.

HRMS (ESI^+) m/z calcd for $C_{17}H_{15}ClF_3O$ $[M+H]^+$ 327.0758, found 327.0741.

1-(2-(4-chlorophenyl)-2-(trifluoromethyl)cyclopropyl)-3-(trifluoromethyl)benzene (25)

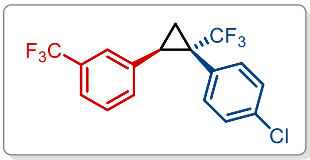

According to **Method D**:

N-triftosylhydrazone derived from 1-(4-chlorophenyl)-2,2,2-trifluoroethan-1-one (161.2 mg, 0.38 mmol), 2-(3-(trifluoromethyl)phenyl)oxirane (28.2 mg, 0.15 mmol), NaH (15.2 mg, 0.38 mmol, 60 wt.% dispersion in mineral oil) and $Tp^{Br3}Ag(thf)$ (4.1 mg, 2.5 mol%) were reacted in dichloromethane (4.0 mL) for 30 min to afford compound **25** as a colorless oil.

Run: 45.9 mg, 84% yield.

Purification: Silica gel column chromatography (petroleum ether as eluent).

^{1}H NMR (500 MHz, $CDCl_3$) δ 7.37 (d, *J* = 8.0 Hz, 1H), 7.22 (t, *J* = 7.5 Hz, 1H), 7.16 (d, *J* = 8.5 Hz, 2H), 7.06-7.03 (m, 3H), 6.90 (d, *J* = 8.0 Hz, 1H), 2.90 (dd, *J* = 9.5, 7.0 Hz, 1H), 1.96 (dd, *J* = 9.5, 6.0 Hz, 1H), 1.70 (t, *J* = 6.0 Hz, 1H).

^{13}C NMR (125 MHz, $CDCl_3$) δ 136.4, 134.7, 133.6, 130.9, 130.5 (q, *J* = 32.5 Hz), 129.4, 128.50, 128.45, 125.4 (q, *J* = 272.5 Hz), 124.8 (q, *J* = 3.8 Hz), 123.8 (q, *J* = 271.3 Hz), 123.5 (q, *J* = 3.8 Hz), 35.6 (q, *J* = 32.5 Hz), 25.4 (q, *J* = 2.5 Hz), 14.9 (q, *J* = 2.5 Hz).

^{19}F NMR (470 MHz, $CDCl_3$) δ -62.90 (s), -69.96 (s).

IR (Film): 1496, 1330, 1133, 821, 801, 745, 701 cm^{-1}.

HRMS (ESI^+) m/z calcd for $C_{17}H_{10}ClF_6$ $[M-H]^-$ 363.0381, found 363.0344.

1-chloro-3-(2-(4-chlorophenyl)-2-(trifluoromethyl)cyclopropyl)benzene (26)

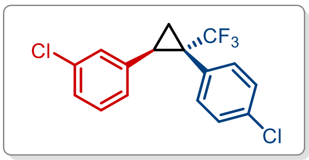

According to **Method D**:

N-triftosylhydrazone derived from 1-(4-chlorophenyl)-2,2,2-trifluoroethan-1-one (161.2 mg, 0.38 mmol), 2-(3-chlorophenyl)oxirane (23.1 mg, 0.15 mmol), NaH (15.2 mg, 0.38 mmol, 60 wt.% dispersion in mineral oil) and $Tp^{Br3}Ag(thf)$ (4.1 mg, 2.5 mol%) were reacted in dichloromethane (4.0 mL) for 30 min to afford compound **26** as a colorless oil.

Run: 40.1 mg, 81% yield.

Purification: Silica gel column chromatography (petroleum ether as eluent).

^{1}H NMR (500 MHz, $CDCl_3$) δ 7.16 (d, *J* = 8.5 Hz, 2H), 7.10-7.04 (m, 3H), 7.01 (t, *J* = 8.0 Hz, 1H), 6.86 (s, 1H), 6.58 (d, *J* = 7.5 Hz, 1H), 2.81 (dd, *J* = 9.5, 7.5 Hz, 1H), 1.89 (dd, *J* = 9.5, 6.0 Hz, 1H), 1.66-1.61 (m, 1H).

^{13}C NMR (150 MHz, $CDCl_3$) δ 137.4, 134.6, 134.0, 133.6, 129.7, 129.2, 128.5, 128.3, 127.0, 125.8, 125.4 (q, *J* = 273.0 Hz), 35.4 (q, *J* = 33.0 Hz), 25.3 (q, *J* = 3.0 Hz), 14.7 (q, *J* = 1.5 Hz).

^{19}F NMR (564 MHz, $CDCl_3$) δ -69.95 (s).

IR (Film): 1600, 1495, 1307, 1296, 1260, 1154, 1093, 821, 785, 725 cm^{-1}.

HRMS (ESI$^+$) m/z calcd for $C_{16}H_{10}Cl_2F_3$ $[M-H]^+$ 329.0117, found 329.0082.

1-(2-(4-chlorophenyl)-2-(trifluoromethyl)cyclopropyl)-2-methylbenzene (27)

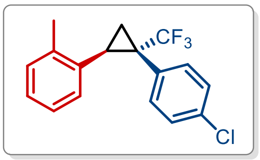

According to **Method D**:

N-triftosylhydrazone derived from 1-(4-chlorophenyl)-2,2,2-trifluoroethan-1-one (161.2 mg, 0.38 mmol), 2-(*o*-tolyl)oxirane (20.1 mg, 0.15 mmol), NaH (15.2 mg, 0.38 mmol, 60 wt.% dispersion in mineral oil) and $Tp^{Br3}Ag(thf)$ (8.2 mg, 5 mol%) were reacted in dichloromethane (4.0 mL) for 30 min to afford compound **27** as a colorless oil.

Run: 37.2 mg, 80% yield.

Purification: Silica gel column chromatography (petroleum ether as eluent).

^{1}H NMR (500 MHz, $CDCl_3$) δ 7.15 (d, *J* = 7.5 Hz, 1H), 7.08 (d, *J* = 8.5 Hz, 2H), 7.03 (t, *J* = 7.5 Hz, 1H), 6.99 (d, *J* = 8.0 Hz, 2H), 6.84 (t, *J* = 7.5 Hz, 1H), 6.35 (d, *J* = 8.0 Hz, 1H), 2.88 (dd, *J* = 9.0, 8.0 Hz, 1H), 2.54 (s, 3H), 1.85-1.78 (m, 2H).

^{13}C NMR (150 MHz, $CDCl_3$) δ 137.6, 134.1, 133.2, 133.0, 130.5, 130.0, 128.2, 126.8, 125.9 (q, *J* =273.0 Hz), 125.7, 125.6, 34.7 (q, *J* = 33.0 Hz), 23.7 (q, *J* = 1.5 Hz), 19.8, 13.1 (q, *J* = 1.5 Hz).

^{19}F NMR (564 MHz, $CDCl_3$) δ -69.37 (s).

IR (Film): 2929, 1495, 1153, 1138, 819, 744, 725 cm^{-1}.

HRMS (ESI^+) m/z calcd for $C_{17}H_{15}ClF_3$ $[M+H]^+$ 311.0809, found 311.0828.

1-chloro-2-(2-(4-chlorophenyl)-2-(trifluoromethyl)cyclopropyl)benzene (28)

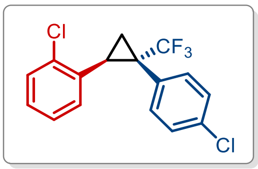

According to **Method D**:

N-triftosylhydrazone derived from 1-(4-chlorophenyl)-2,2,2-trifluoroethan-1-one (161.2 mg, 0.38 mmol), 2-(2-chlorophenyl)oxirane (23.1 mg, 0.15 mmol), NaH (15.2 mg, 0.38 mmol, 60 wt.% dispersion in mineral oil) and $Tp^{Br3}Ag(thf)$ (8.2 mg, 5 mol%) were reacted in dichloromethane (4.0 mL) for 30 min to afford compound **28** as a colorless oil.

Run: 39.1 mg, 79% yield.

Purification: Silica gel column chromatography (petroleum ether as eluent).

^{1}H NMR (500 MHz, $CDCl_3$) δ 7.36 (d, *J* = 8.0 Hz, 1H), 7.14 (d, *J* = 8.5 Hz, 2H), 7.09 (d, *J* = 8.5 Hz, 2H), 7.06 (t, *J* = 8.0 Hz, 1H), 6.91 (t, *J* = 8.0 Hz, 1H), 6.48 (d, *J* = 8.0 Hz, 1H), 3.14 (dd, *J* = 9.0, 8.0 Hz, 1H), 1.86 (dd, *J* = 9.5, 6.0 Hz, 1H), 1.78 (t, *J* = 6.5 Hz, 1H).

^{13}C NMR (150 MHz, $CDCl_3$) δ 135.9, 134.3, 133.14, 133.05, 130.3, 129.4, 128.3, 128.2, 127.5, 126.5, 125.6 (q, *J* = 273.0 Hz), 34.9 (q, *J* = 33.0 Hz), 24.2 (q, *J* = 1.5 Hz), 12.8 (q, *J* = 1.5 Hz).

^{19}F NMR (564 MHz, $CDCl_3$) δ -69.76 (s).

IR (Film): 1495, 1307, 1156, 1141, 822, 756, 745, 732 cm^{-1}.

HRMS (ESI^+) m/z calcd for $C_{16}H_{10}Cl_2F_3$ $[M-H]^-$ 329.0117, found 329.0179.

1,2-dichloro-4-(2-(4-chlorophenyl)-2-(trifluoromethyl)cyclopropyl)benzene (29)

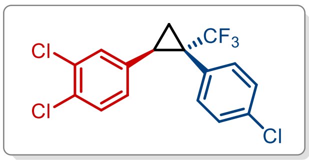

According to **Method D**:

N-triftosylhydrazone derived from 1-(4-chlorophenyl)-2,2,2-trifluoroethan-1-one (161.2 mg, 0.38 mmol), 2-(3,4-dichlorophenyl)oxirane (28.2 mg, 0.15 mmol), NaH (15.2 mg, 0.38 mmol, 60 wt.% dispersion in mineral oil) and Tp^{Br3}Ag(thf) (4.1 mg, 2.5 mol%) were reacted in dichloromethane (4.0 mL) for 30 min to afford compound **29** as a white solid (mp: 96-97 °C).

Run: 43.7 mg, 80% yield.

Purification: Silica gel column chromatography (petroleum ether as eluent).

^{1}H NMR (500 MHz, $CDCl_3$) δ 7.39 (d, *J* = 2.0 Hz, 1H), 7.13 (s, 4H), 6.91 (dd, *J* = 8.5, 2.0 Hz, 1H), 6.41 (d, *J* = 8.0 Hz, 1H), 3.08 (dd, *J* = 9.0, 7.0 Hz, 1H), 1.88 (dd, *J* = 9.5, 6.0 Hz, 1H), 1.74 (t, *J* = 6.5 Hz, 1H).

^{13}C NMR (150 MHz, $CDCl_3$) δ 136.4, 134.6, 133.4, 133.0, 131.8, 129.9, 129.3, 128.5, 128.4, 126.8, 125.4 (q, *J* = 273.0 Hz), 35.0 (q, *J* = 33.0 Hz), 23.8 (q, *J* = 3.0 Hz), 13.0 (q, *J* = 1.5 Hz).

^{19}F NMR (564 MHz, $CDCl_3$) δ -69.86 (s).

IR (Film): 1592, 1559, 1495, 1380, 1307, 1297, 1281, 1159, 1141, 1094, 858, 822, 748, 737, 720 cm^{-1}.

HRMS (ESI^+) m/z calcd for $C_{16}H_9Cl_3F_3$ $[M-H]^-$ 362.9727, found 362.9629.

1-(2-(4-chlorophenyl)-2-(trifluoromethyl)cyclopropyl)naphthalene (30)

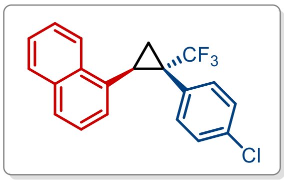

According to **Method D**:

N-triftosylhydrazone derived from 1-(4-chlorophenyl)-2,2,2-trifluoroethan-1-one (161.2 mg, 0.38 mmol), 2-(naphthalen-1-yl)oxirane (25.5 mg, 0.15 mmol), NaH (15.2 mg, 0.38 mmol, 60 wt.% dispersion in mineral oil) and $Tp^{Br3}Ag(thf)$ (8.2 mg, 5 mol%) were reacted in dichloromethane (4.0 mL) for 1 h to afford compound **30** as a white solid (mp: 56-57 °C).

Run: 32.2 mg, 62% yield.

Purification: Silica gel column chromatography (petroleum ether as eluent).

^{1}H NMR (500 MHz, $CDCl_3$) δ 8.38 (d, *J* = 8.5 Hz, 1H), 7.84 (d, *J* = 8.0 Hz, 1H), 7.68 (t, *J* = 7.5 Hz, 1H), 7.64 (d, *J* = 8.0 Hz, 1H), 7.55 (t, *J* = 7.5 Hz, 1H), 7.14 (t, *J* = 7.5 Hz, 1H), 6.94 (d, *J* = 8.5 Hz, 2H), 6.89 (d, *J* = 8.5 Hz, 2H), 6.77 (d, *J* = 7.0 Hz, 1H), 3.39 (t, *J* = 8.5 Hz, 1H), 1.96 (d, *J* = 8.5 Hz, 2H).

^{13}C NMR (125 MHz, $CDCl_3$) δ 134.1, 133.5, 133.3, 132.8, 130.8, 130.4, 128.8, 128.0, 127.8, 126.7, 126.0 (q, *J* = 272.5 Hz), 125.9, 125.1, 124.1, 123.3, 35.0 (q, *J* = 32.5 Hz), 23.7 (q, *J* = 2.5 Hz), 12.8 (q, *J* = 1.3 Hz).

^{19}F NMR (564 MHz, $CDCl_3$) δ -69.09 (s).

IR (Film): 2926, 1598, 1495, 1343, 1295, 1146, 1093, 1016, 821, 800, 777, 745, 723 cm^{-1}.

HRMS (ESI^+) m/z calcd for $C_{20}H_{13}ClF_3$ $[M-H]^-$ 345.0663, found 345.0627.

2-(2-(4-chlorophenyl)-2-(trifluoromethyl)cyclopropyl)thiophene (31)

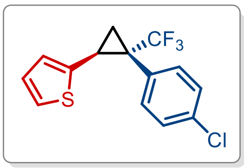

According to **Method D**:

N-triftosylhydrazone derived from 1-(4-chlorophenyl)-2,2,2-trifluoroethan-1-one (161.2 mg, 0.38 mmol), 2-(thiophen-2-yl)oxirane (18.9 mg, 0.15 mmol), NaH (15.2 mg, 0.38 mmol, 60 wt.% dispersion in mineral oil) and $Tp^{Br3}Ag(thf)$ (8.2 mg, 5 mol%) were reacted in dichloromethane (4.0 mL) for 30 min to afford compound **31** as a colorless oil.

Run: 34.0 mg, 75% yield.

Purification: Silica gel column chromatography (petroleum ether as eluent).

^{1}H NMR (500 MHz, $CDCl_3$) δ 7.23-7.14 (m, 4H), 7.00 (dd, *J* = 5.0, 1.0 Hz, 1H), 6.76 (dd, *J* = 5.0, 3.5 Hz, 1H), 6.48 (d, *J* = 3.5 Hz, 1H), 3.03 (dd, *J* = 9.5, 6.5 Hz, 1H), 1.98 (dd, *J* = 9.5, 6.0 Hz, 1H), 1.57 (td, *J* = 6.5, 1.5 Hz, 1H).

^{13}C NMR (150 MHz, $CDCl_3$) δ 139.2, 134.6, 133.5, 130.1, 128.3, 126.8, 125.4, 125.4 (q, *J* = 273.0 Hz), 124.2, 35.4 (q, *J* = 33.0 Hz), 21.3 (q, *J* = 3.0 Hz), 16.9 (q, *J* = 1.5 Hz).

^{19}F NMR (564 MHz, $CDCl_3$) δ -69.78 (s).

HRMS (ESI^+) m/z calcd for $C_{14}H_{11}ClF_3S$ $[M+H]^+$ 303.0217, found 303.0221.

1-chloro-4-(2,2-dimethyl-1-(trifluoromethyl)cyclopropyl)benzene (32)

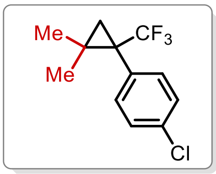

According to **Method C**:

N-triftosylhydrazone derived from 1-(4-chlorophenyl)-2,2,2-trifluoroethan-1-one (258.0 mg, 0.6 mmol), 2,2-dimethyloxirane (10.8 mg, 0.15 mmol), NaH (27.0 mg, 0.68 mmol, 60 wt.% dispersion in mineral oil) and $Tp^{Br3}Ag(thf)$ (16.4 mg, 10 mol%) were reacted in dichloromethane (4.0 mL) for 10 h to afford compound **32** as a colorless oil.

Run: 31.3 mg, 84% yield.

Purification: Silica gel column chromatography (petroleum ether as eluent).

^{1}H NMR (500 MHz, $CDCl_3$) δ 7.37-7.26 (m, 3H), 7.22-7.13 (m, 1H), 1.43-1.39 (m, 4H), 1.10-1.04 (m, 1H), 0.79 (s, 3H).

^{13}C NMR (150 MHz, $CDCl_3$) δ 134.1, 133.8, 132.5, 132.0, 128.7, 128.3, 126.6 (q, *J* = 273.0 Hz), 36.3 (q, *J* =33.0 Hz), 25.3, 23.0 (q, *J* = 2.5 Hz), 22.4, 20.3 (q, *J* = 2.5 Hz).

^{19}F NMR (564 MHz, $CDCl_3$) δ -60.51 (s).

IR (Film): 2921, 2851, 1656, 1469, 1260, 1905, 934, 799 cm^{-1}.

HRMS (ESI^+) m/z calcd for $C_{12}H_{11}ClF_3$ $[M-H]^-$ 247.0507, found 247.0557.

1-(4-chlorophenyl)-1-(trifluoromethyl)-7,10-dioxadispiro[2.2.4^6.2^3]dodecane (33)

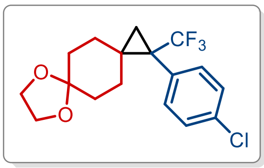

According to **Method C**:

N-triftosylhydrazone derived from 1-(4-chlorophenyl)-2,2,2-trifluoroethan-1-one (258.0 mg, 0.6 mmol), 1,7,10-trioxadispiro[2.2.4^6.2^3]dodecane (25.5 mg, 0.15 mmol), NaH (27.0 mg, 0.68 mmol, 60 wt.% dispersion in mineral oil) and Tp^{Br3}Ag(thf) (16.4 mg, 10 mol%) were reacted in dichloromethane (4.0 mL) for 10 h to afford compound **33** as a colorless oil.

Run: 40.0 mg, 77% yield.

Purification: Silica gel column chromatography (petroleum ether as eluent).

^{1}H NMR (500 MHz, $CDCl_3$) δ 7.39-7.33 (m, 1H), 7.32-7.25 (m, 2H), 7.20-7.12 (m, 1H), 3.98-3.86 (m, 4H), 2.11-1.99 (m, 1H), 1.86-1.75 (m, 2H), 1.74-1.66 (m, 1H), 1.65-1.58 (m, 1H), 1.55-1.46 (m, 2H), 1.42 (d, J = 5.5 Hz, 1H), 1.10 (d, J = 3.0 Hz, 1H), 0.69-0.50 (m, 1H).

^{13}C NMR (150 MHz, $CDCl_3$) δ 134.0, 133.4, 132.2, 132.0, 128.7, 128.3, 126.5 (q, J = 274.5 Hz), 108.2, 64.4, 64.3, 37.2 (q, J = 33.0 Hz), 34.0, 33.6, 31.9, 28.2, 26.7 (q, J = 3.0 Hz), 20.6 (q, J = 1.5 Hz).

^{19}F NMR (564 MHz, $CDCl_3$) δ -59.74 (s).

HRMS (ESI$^+$) m/z calcd for $C_{18}H_{21}ClF_3O_2$ $[M+H]^+$ 361.1177, found 361.1185.

1-(4-chlorophenyl)-1-(trifluoromethyl)-6-oxaspiro[2.5]octane (34)

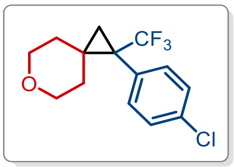

According to **Method C**:

N-triftosylhydrazone derived from 1-(4-chlorophenyl)-2,2,2-trifluoroethan-1-one (258.0 mg, 0.6 mmol), 1,6-dioxaspiro[2.5]octane (17.1 mg, 0.15 mmol), NaH (27.0 mg, 0.68 mmol, 60 wt.% dispersion in mineral oil) and $Tp^{Br3}Ag(thf)$ (16.4 mg, 10 mol%) were reacted in dichloromethane (4.0 mL) for 10 h to afford compound **34** as a colorless oil.

Run: 31.8 mg, 73% yield.

Purification: Silica gel column chromatography (petroleum ether as eluent).

^{1}H NMR (500 MHz, $CDCl_3$) δ 7.38 (d, *J* = 8.0 Hz, 1H), 7.35-7.29 (m, 2H), 7.22-7.16 (m, 1H), 4.00 (dt, *J* = 11.5, 3.5 Hz, 1H), 3.81 (dt, *J* = 11.5, 3.5 Hz, 1H), 3.58 (td, *J* = 11.0, 2.5 Hz, 1H), 3.43 (td, *J* = 11.0, 2.5 Hz, 1H), 2.20-2.10 (m, 1H), 1.71-1.61 (m, 2H), 1.52 (d, *J* = 5.5 Hz, 1H), 1.19 (d, *J* = 3.5 Hz, 1H), 0.40 (dd, *J* = 14.0, 2.5 Hz, 1H).

^{13}C NMR (150 MHz, $CDCl_3$) δ 134.2, 132.6, 132.24, 132.17, 128.8, 128.5, 126.2 (q, *J* = 274.5 Hz), 67.1, 66.9, 36.6 (q, *J* = 31.5 Hz), 34.8, 30.70 (q, *J* = 1.5 Hz), 27.2, 20.7 (q, *J* = 1.5 Hz).

^{19}F NMR (564 MHz, $CDCl_3$) δ -60.19 (s).

HRMS (ESI^+) m/z calcd for $C_{15}H_{16}ClF_3O$ $[M+H]^+$ 304.0842, found 304.0843.

benzyl 1-(4-chlorophenyl)-1-(trifluoromethyl)-6-azaspiro[2.5]octane-6-carboxylate (35)

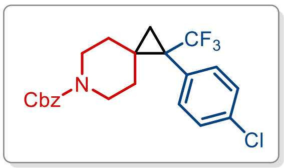

According to **Method C**:

N-triftosylhydrazone derived from 1-(4-chlorophenyl)-2,2,2-trifluoroethan-1-one (258.0 mg, 0.6 mmol), benzyl 1-oxa-6-azaspiro[2.5]octane-6-carboxylate (37.1 mg, 0.15 mmol), NaH (27.0 mg, 0.68 mmol, 60 wt.% dispersion in mineral oil) and $Tp^{Br3}Ag(thf)$ (16.4 mg, 10 mol%) were reacted in dichloromethane (4.0 mL) for 10 h to afford compound **35** as a colorless oil.

Run: 41.3 mg, 65% yield.

Purification: Silica gel column chromatography (2% EtOAc in petroleum ether).

^{1}H NMR (500 MHz, $CDCl_3$) δ 7.42-7.27 (m, 9H), 5.63 (d, *J* = 19.0 Hz, 1H), 5.14 (s, 2H), 4.57 (q, *J* = 6.5 Hz, 1H), 4.07-3.92 (m, 3H), 3.87 (d, *J* = 11.5 Hz, 1H), 3.63-3.48 (m, 2H), 2.20-2.01 (m, 2H).

^{13}C NMR (150 MHz, $CDCl_3$) δ 155.4, 136.7, 135.6, 131.1, 129.6, 128.9, 128.5, 128.0, 127.9, 123.6 (q, *J* = 280.5 Hz), 123.3, 122.3, 77.4, 73.3, 67.1, 43.1 (d, *J* = 18.0 Hz), 40.3 (d, *J* = 51.0 Hz), 25.6 (d, *J* = 42.0 Hz).

^{19}F NMR (564 MHz, $CDCl_3$) δ -76.49 (d, *J* = 5.7 Hz).

HRMS (ESI$^+$) m/z calcd for $C_{23}H_{24}ClF_3NO_2$ $[M+H]^+$ 438.1442, found 438.1444.

(2-(4-chlorophenyl)-2-(trifluoromethyl)cyclopropane-1,1-diyl)dibenzene (36)

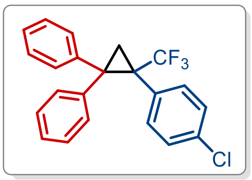

According to **Method D**:

N-triftosylhydrazone derived from 1-(4-chlorophenyl)-2,2,2-trifluoroethan-1-one (161.2 mg, 0.38 mmol), 2,2-diphenyloxirane (29.4 mg, 0.15 mmol), NaH (15.2 mg, 0.38 mmol, 60 wt.% dispersion in mineral oil) and $Tp^{Br3}Ag(thf)$ (8.2 mg, 5 mol%) were reacted in dichloromethane (4.0 mL) for 1 h to afford compound **36** as a white solid (mp: 105-106 °C).

Run: 50.8 mg, 91% yield.

Purification: Silica gel column chromatography (petroleum ether as eluent).

^{1}H NMR (500 MHz, $CDCl_3$) δ 7.59 (d, *J* = 7.5 Hz, 2H), 7.34 (t, *J* = 7.5 Hz, 2H), 7.29 (d, *J* = 8.0 Hz, 2H), 7.25 (t, *J* = 6.0 Hz, 1H), 7.14 (d, *J* = 8.0 Hz, 2H), 7.09 (d, *J* = 8.5 Hz, 2H), 7.01 (t, *J* = 7.5 Hz, 2H), 6.95 (t, *J* = 7.5 Hz, 1H), 2.39-2.35 (m, 1H), 2.24 (d, *J* = 6.5 Hz, 1H).

^{13}C NMR (150 MHz, $CDCl_3$) δ 140.8, 139.7, 133.9, 132.8, 131.7, 129.8, 128.8, 128.4, 128.2, 128.0, 127.1, 126.5, 125.8 (q, *J* = 274.5 Hz), 42.3, 39.6 (q, *J* = 31.5 Hz), 19.5.

^{19}F NMR (564 MHz, $CDCl_3$) δ -61.65 (s).

IR (Film): 1494, 1332, 1221, 1167, 1124, 1093, 746, 705, 695 cm^{-1}.

HRMS (ESI^+) m/z calcd for $C_{22}H_{15}ClF_3$ $[M-H]^-$ 371.0820, found 371.0782.

4,4',4''-(2-(trifluoromethyl)cyclopropane-1,1,2-triyl)tris(chlorobenzene) (37)

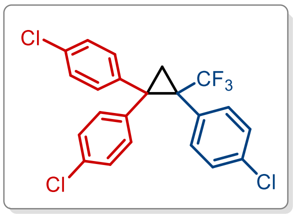

According to **Method D**:

N-triftosylhydrazone derived from 1-(4-chlorophenyl)-2,2,2-trifluoroethan-1-one (161.2 mg, 0.38 mmol), 2,2-bis(4-chlorophenyl)oxirane (39.6 mg, 0.15 mmol), NaH (15.2 mg, 0.38 mmol, 60 wt.% dispersion in mineral oil) and Tp^{Br3}Ag(thf) (8.2 mg, 5 mol%) were reacted in dichloromethane (4.0 mL) for 1 h to afford compound **37** as a white solid (mp: 145-146 °C).

Run: 58.1 mg, 88% yield.

Purification: Silica gel column chromatography (petroleum ether as eluent).

^{1}H NMR (500 MHz, $CDCl_3$) δ 7.42 (d, *J* = 8.0 Hz, 2H), 7.36 (d, *J* = 8.5 Hz, 2H), 7.23 (d, *J* = 8.5 Hz, 2H), 7.20-7.12 (m, 4H), 6.83 (d, *J* = 8.5 Hz, 2H), 3.31 (d, *J* = 7.5 Hz, 1H), 3.26 (d, *J* = 7.5 Hz, 1H).

^{13}C NMR (150 MHz, $CDCl_3$) δ 134.9, 133.9, 133.5, 133.19, 133.16, 133.1, 130.5, 130.4, 129.3, 128.8, 128.6, 128.5, 124.9 (q, *J* = 274.5 Hz), 41.9 (q, *J* = 30.0 Hz), 33.3, 30.0.

^{19}F NMR (564 MHz, $CDCl_3$) δ -63.83 (s).

IR (Film): 2959, 2930, 2860, 1495, 1465, 1400, 1346, 1307, 1156, 1135, 1094, 1016, 820, 744, 724, 680 cm^{-1}.

HRMS (ESI^+) m/z calcd for $C_{22}H_{13}Cl_3F_3$ $[M-H]^-$ 438.0040, found 439.9997.

1-chloro-4-(2-methyl-2-phenyl-1-(trifluoromethyl)cyclopropyl)benzene (38)

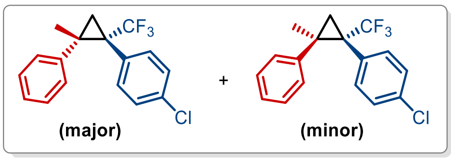

According to **Method D**:

N-triftosylhydrazone derived from 1-(4-chlorophenyl)-2,2,2-trifluoroethan-1-one (161.2 mg, 0.38 mmol), 2-methyl-2-phenyloxirane (20.1 mg, 0.15 mmol), NaH (15.2 mg, 0.38 mmol, 60 wt.% dispersion in mineral oil) and $Tp^{Br3}Ag(thf)$ (8.2 mg, 5 mol%) were reacted in dichloromethane (4.0 mL) for 1 h to afford compound **38** as a colorless oil. The product was obtained as a mixture of stereoisomers (10:1) determined by NMR.

Run: 39.5 mg, 85% yield.

Purification: Silica gel column chromatography (petroleum ether as eluent).

Major isomer:

^{1}H NMR (500 MHz, $CDCl_3$) δ 7.11-6.97 (m, 9H), 2.11-2.07 (m, 1H), 1.79 (s, 3H), 1.70 (d, *J* = 6.5 Hz, 1H).

^{13}C NMR (125 MHz, $CDCl_3$) δ 141.2, 133.3, 132.9, 132.1, 128.0, 127.9, 127.8, 126.5, 126.4 (q, *J* = 273.8 Hz), 38.1 (q, *J* = 31.3 Hz), 31.9, 21.7 (q, *J* = 2.0 Hz), 20.1 (q, *J* = 2.5 Hz).

^{19}F NMR (470 MHz, $CDCl_3$) δ -59.44 (s).

IR (Film): 1496, 1344, 1260, 1159, 1134, 1093, 819, 699 cm^{-1}.

HRMS (ESI^+) m/z calcd for $C_{17}H_{15}ClF_3$ $[M+H]^+$ 311.0809, found 311.0826.

1-chloro-4-(2-cyclohexyl-2-phenyl-1-(trifluoromethyl)cyclopropyl)benzene (39)

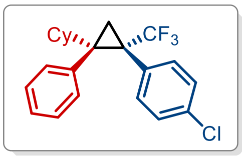

According to **Method D**:

N-triftosylhydrazone derived from 1-(4-chlorophenyl)-2,2,2-trifluoroethan-1-one (161.2 mg, 0.38 mmol), 2-cyclohexyl-2-phenyloxirane (30.3 mg, 0.15 mmol), NaH (15.2 mg, 0.38 mmol, 60 wt.% dispersion in mineral oil) and $Tp^{Br3}Ag(thf)$ (8.2 mg, 5 mol%) were reacted in dichloromethane (4.0 mL) for 1 h to afford compound **39** as a white solid (mp: 103-104 °C).

Run: 37.4 mg, 66% yield.

Purification: Silica gel column chromatography (petroleum ether as eluent).

^{1}H NMR (500 MHz, $CDCl_3$) δ 7.19-6.79 (m, 9H), 2.12-2.06 (m, 1H), 1.99-1.93 (m, 1H), 1.79-1.64 (m, 5H), 1.60-1.53 (m, 1H), 1.32-1.12 (m, 3H), 0.96-0.84 (m, 1H), 0.75-0.64 (m, 1H).

^{13}C NMR (125 MHz, $CDCl_3$) δ 136.9, 133.1, 133.0, 131.3, 127.9, 127.1 (q, J = 275.0 Hz) 127.0, 126.9, 126.5, 44.2, 42.2 (q, J = 3.0 Hz), 38.6 (q, J = 31.0 Hz), 31.7, 31.1, 26.9, 26.7, 26.1, 20.3.

^{19}F NMR (564 MHz, $CDCl_3$) δ -56.58 (s).

IR (Film): 2930, 2855, 1495, 1343, 1295, 1146, 1093, 1016, 821, 800, 777, 745, 723 cm^{-1}.

HRMS (ESI^+) m/z calcd for $C_{22}H_{21}ClF_3$ $[M-H]^-$ 377.1289, found 377.1250.

2-(2-(4-chlorophenyl)-1-methyl-2-(trifluoromethyl)cyclopropyl)naphthalene (40)

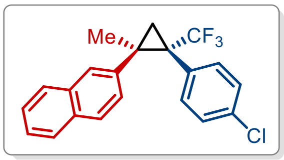

According to **Method D**:

N-triftosylhydrazone derived from 1-(4-chlorophenyl)-2,2,2-trifluoroethan-1-one (161.2 mg, 0.38 mmol), 2-methyl-2-(naphthalen-2-yl)oxirane (27.6 mg, 0.15 mmol), NaH (15.2 mg, 0.38 mmol, 60 wt.% dispersion in mineral oil) and $Tp^{Br3}Ag(thf)$ (8.2 mg, 5 mol%) were reacted in dichloromethane (4.0 mL) for 1 h to afford compound **40** as a colorless oil.

Run: 48.6 mg, 90% yield.

Purification: Silica gel column chromatography (petroleum ether as eluent).

^{1}H NMR (500 MHz, $CDCl_3$) δ 7.70-7.62 (m, 2H), 7.58 (d, *J* = 8.5 Hz, 1H), 7.43 (s, 1H), 7.41-7.34 (m, 2H), 7.26 (dd, *J* = 8.5, 1.5 Hz, 1H), 7.14 (d, *J* = 8.0 Hz, 2H), 6.97 (d, *J* = 8.5 Hz, 2H), 2.25-2.21 (m, 1H), 1.85 (s, 3H), 1.80 (d, *J* = 6.0 Hz, 1H).

^{13}C NMR (150 MHz, $CDCl_3$) δ 139.0, 133.4, 133.0, 132.7, 132.0, 128.0, 127.7, 127.6, 127.5, 126.6, 126.5 (q, *J* = 274.5 Hz), 126.1, 126.0, 125.8, 38.3 (q, *J* = 31.5 Hz), 32.3, 21.9 (q, *J* = 1.5 Hz), 20.4 (q, *J* = 3.0 Hz).

^{19}F NMR (564 MHz, $CDCl_3$) δ -59.43 (s).

IR (Film): 1495, 1344, 1261, 1158, 1128, 1094, 815, 749 cm^{-1}.

HRMS (ESI^+) m/z calcd for $C_{21}H_{15}ClF_3$ $[M-H]^-$ 359.0819, found 359.0790.

6-(4-chlorophenyl)-6-(trifluoromethyl)bicyclo[3.1.0]hexane (41)

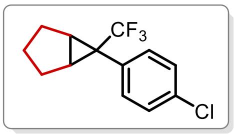

According to **Method C**:

N-triftosylhydrazone derived from 1-(4-chlorophenyl)-2,2,2-trifluoroethan-1-one (258.0 mg, 0.6 mmol), 6-oxabicyclo[3.1.0]hexane (12.6 mg, 0.15 mmol), NaH (27.0 mg, 0.68 mmol, 60 wt.% dispersion in mineral oil) and $Tp^{Br3}Ag(thf)$ (16.4 mg, 10 mol%) were reacted in dichloromethane (4.0 mL) for 12 h to afford compound **41** as a colorless oil.

Run: 34.3 mg, 88% yield.

Purification: Silica gel column chromatography (petroleum ether as eluent).

^{1}H NMR (500 MHz, $CDCl_3$) δ 7.34 (d, *J* = 8.5 Hz, 2H), 7.26 (d, *J* = 6.5 Hz, 2H), 2.11-2.03 (m, 2H), 1.91 (dd, *J* = 20.7, 10.1 Hz, 2H), 1.72 (dd, *J* = 13.5, 9.0 Hz, 2H), 1.23-1.21 (m, 2H).

^{13}C NMR (150 MHz, $CDCl_3$) δ 133.2, 131.3, 127.7, 127.4, 124.7 (d, *J* = 271.5 Hz), 33.3, 32.5 (q, *J* = 31.5 Hz), 26.5, 25.0, 24.7, 21.1.

^{19}F NMR (564 MHz, $CDCl_3$) δ -68.93 (s).

IR (Film): 2928, 1494, 1343, 1261, 1162, 1133, 823, 742 cm^{-1}.

HRMS (ESI^+) m/z calcd for $C_{13}H_{11}ClF_3$ $[M-H]^-$ 259.0507, found 259.0559.

1-(2-butyl-3-methyl-1-(trifluoromethyl)cyclopropyl)-4-chlorobenzene (42)

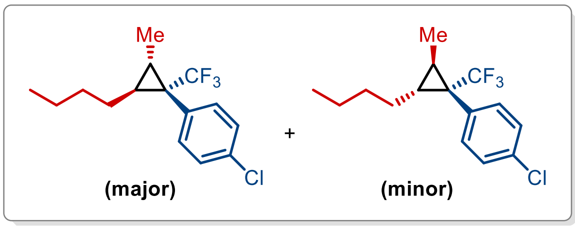

According to **Method C**:

N-triftosylhydrazone derived from 1-(4-chlorophenyl)-2,2,2-trifluoroethan-1-one (258.0 mg, 0.6 mmol), 2-butyl-3-methyloxirane (17.1 mg, 0.15 mmol), NaH (27.0 mg, 0.68 mmol, 60 wt.% dispersion in mineral oil) and $Tp^{Br3}Ag(thf)$ (16.4 mg, 10 mol%) were reacted in dichloromethane (4.0 mL) for 12 h to afford compound **42** as a colorless oil. The product was obtained as a mixture of stereoisomers (5:1) determined by NMR.

Run: 39.2 mg, 90% yield.

Purification: Silica gel column chromatography (petroleum ether as eluent).

Major isomer:

^{1}H NMR (600 MHz, $CDCl_3$) δ 7.36-7.32 (m, 2H), 7.23 (d, *J* = 8.5 Hz, 2H), 1.68-1.61 (m, 1H), 1.56-1.47 (m, 2H), 1.45-1.39 (m, 1H), 1.37-1.26 (m, 3H), 0.94 (d, *J* = 6.5 Hz, 3H), 0.88 (t, *J* = 7.0 Hz, 3H), 0.87-0.81 (m, 1H).

^{13}C NMR (150 MHz, $CDCl_3$) δ 134.6, 132.5, 128.5, 128.4, 126.2 (q, *J* = 273.0 Hz), 33.3 (q, *J* = 31.5 Hz), 31.6, 24.7, 23.5 (q, *J* = 1.5 Hz), 22.7, 17.6, 14.0, 9.7.

^{19}F NMR (564 MHz, $CDCl_3$) δ -69.95 (s).

IR (Film): 2959, 2930, 2860, 1495, 1346, 1307, 1156, 1135, 1096, 820, 744 cm^{-1}.

HRMS (ESI^+) m/z calcd for $C_{15}H_{19}ClF_3$ $[M+H]^+$ 291.1122, found 291.1176.

1-chloro-4-(2-methyl-3-phenyl-1-(trifluoromethyl)cyclopropyl)benzene (43)

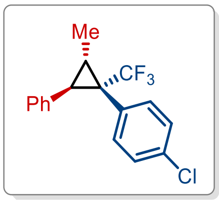

According to **Method D**:

N-triftosylhydrazone derived from 1-(4-chlorophenyl)-2,2,2-trifluoroethan-1-one (161.2 mg, 0.38 mmol), 2-methyl-3-phenyloxirane (20.1 mg, 0.15 mmol), NaH (15.2 mg, 0.38 mmol, 60 wt.% dispersion in mineral oil) and $Tp^{Br3}Ag(thf)$ (8.2 mg, 5 mol%) were reacted in dichloromethane (4.0 mL) for 1 h to afford compound **43** as a colorless oil.

Run: 41.4 mg, 89% yield.

Purification: Silica gel column chromatography (petroleum ether as eluent).

^{1}H NMR (500 MHz, $CDCl_3$) δ 7.15-7.08 (m, 5H), 6.99 (d, *J* = 8.5 Hz, 2H), 6.77-6.70 (m, 2H), 2.69 (d, *J* = 7.5 Hz, 1H), 2.04-1.95 (m, 1H), 1.56 (d, *J* = 6.0 Hz, 3H).

^{13}C NMR (125 MHz, $CDCl_3$) δ 135.7, 134.1, 134.0, 131.9, 128.1, 128.0, 127.9, 126.6, 125.8 (q, *J* = 273.8 Hz), 39.3 (q, *J* = 31.3 Hz), 33.4 (q, *J* = 2.5 Hz), 24.2, 12.6 (q, *J* = 1.3 Hz).

^{19}F NMR (470 MHz, $CDCl_3$) δ -62.57 (s).

IR (Film): 2939, 2887, 1599, 1494, 1363, 1275, 1177, 1154, 1093, 822, 744,723 cm^{-1}.

HRMS (ESI^+) m/z calcd for $C_{17}H_{15}ClF_3$ $[M+H]^+$ 311.0809, found 311.0828.

(3-(4-chlorophenyl)-3-(trifluoromethyl)cyclopropane-1,2-diyl)dibenzene (44)

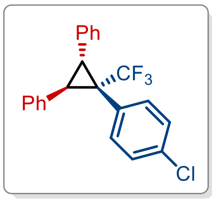

According to **Method D**:

N-triftosylhydrazone derived from 1-(4-chlorophenyl)-2,2,2-trifluoroethan-1-one (161.2 mg, 0.38 mmol), 2,3-diphenyloxirane (29.4 mg, 0.15 mmol), NaH (15.2 mg, 0.38 mmol, 60 wt.% dispersion in mineral oil) and $Tp^{Br3}Ag(thf)$ (8.2 mg, 5 mol%) were reacted in dichloromethane (4.0 mL) for 1 h to afford compound **44** as a white solid (mp: 133-134 °C).

Run: 26.2 mg, 47% yield.

Purification: Silica gel column chromatography (petroleum ether as eluent).

^{1}H NMR (500 MHz, $CDCl_3$) δ 7.51 (d, *J* = 7.5 Hz, 2H), 7.39 (t, *J* = 7.5 Hz, 2H), 7.32 (t, *J* = 7.5 Hz, 1H), 7.22-7.16 (m, 7H), 6.96-6.89 (m, 2H), 3.43-3.34 (m, 2H).

^{13}C NMR (150 MHz, $CDCl_3$) δ 134.9, 134.5, 134.0, 131.3, 129.1, 128.5, 128.4, 128.3, 128.2, 127.4, 127.1, 125.1 (q, *J* = 273.0 Hz), 41.7 (q, *J* = 30.0 Hz), 33.7, 30.4 (q, *J* = 1.5 Hz).

^{19}F NMR (564 MHz, $CDCl_3$) δ -63.73 (s).

IR (Film): 1494, 1168, 1140, 1091, 824, 756, 722, 696 cm^{-1}.

HRMS (ESI^+) m/z calcd for $C_{22}H_{17}ClF_3$ $[M+H]^+$ 373.0965, found 373.0935.

1-(4-chlorophenyl)-1-(trifluoromethyl)-1,1a,6,6a-tetrahydrocyclopropa[a]indene (45)

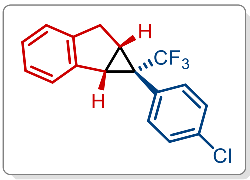

According to **Method D**:

N-triftosylhydrazone derived from 1-(4-chlorophenyl)-2,2,2-trifluoroethan-1-one (161.2 mg, 0.38 mmol), 1a,6a-dihydro-6H-indeno[1,2-*b*]oxirene (19.8 mg, 0.15 mmol), NaH (15.2 mg, 0.38 mmol, 60 wt.% dispersion in mineral oil) and $Tp^{Br3}Ag(thf)$ (8.2 mg, 5 mol%) were reacted in dichloromethane (4.0 mL) for 1 h to afford compound **45** as a white solid (mp: 89-90 °C).

Run: 42.0 mg, 91% yield.

Purification: Silica gel column chromatography (petroleum ether as eluent).

^{1}H NMR (600 MHz, $CDCl_3$) δ 7.38 (d, *J* = 7.8 Hz, 1H), 7.09 (t, *J* = 7.8 Hz, 1H), 7.06 (d, *J* = 8.4 Hz, 2H), 7.00 (d, *J* = 7.8 Hz, 2H), 6.94 (t, *J* = 7.2 Hz, 1H), 6.75 (d, *J* = 7.2 Hz, 1H), 3.28 (d, *J* = 6.6 Hz, 1H), 3.23 (dd, *J* = 18.0, 7.2 Hz, 1H), 2.73 (d, *J* = 18.0 Hz, 1H), 2.61 (t, *J* = 7.2 Hz, 1H).

^{13}C NMR (150 MHz, $CDCl_3$) δ 142.4, 140.1, 134.2, 133.8, 128.0, 127.4, 126.8, 126.5, 125.5 (q, *J* = 273.0 Hz), 125.0, 124.5, 36.3 (q, *J* = 31.5 Hz), 33.6 (q, *J* = 1.5 Hz), 32.4, 25.1 (q, *J* = 1.5 Hz).

^{19}F NMR (564 MHz, $CDCl_3$) δ -68.70 (s).

IR (Film): 2923, 2360, 1598, 1494, 1481, 1340, 1280, 1268, 1224, 1155, 1090, 1013, 821, 725 cm^{-1}.

HRMS (ESI^+) m/z calcd for $C_{17}H_{11}ClF_3$ $[M-H]^-$ 307.0507, found 307.0560.

(1-(trifluoromethyl)cyclopropane-1,2-diyl)dibenzene (46)

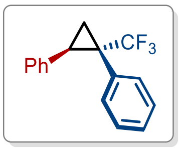

According to **Method D**:

N-triftosylhydrazone derived from 2,2,2-trifluoro-1-phenylethan-1-one (150.5 mg, 0.38 mmol), styrene oxide (18.0 mg, 0.15 mmol), NaH (15.2 mg, 0.38 mmol, 60 wt.% dispersion in mineral oil) and $Tp^{Br3}Ag(thf)$ (8.2 mg, 5 mol%) were reacted in dichloromethane (4.0 mL) for 30 min to afford compound **46** as a white solid (mp: 68-69 °C).

Run: 31.5 mg, 80% yield.

Purification: Silica gel column chromatography (petroleum ether as eluent).

^{1}H NMR (500 MHz, $CDCl_3$) δ 7.22-7.04 (m, 8H), 6.82-6.72 (m, 2H), 2.84 (dd, *J* = 9.5, 7.0 Hz, 1H), 1.88 (dd, *J* = 9.5, 6.0 Hz, 1H), 1.69-1.66 (m, 1H).

^{13}C NMR (150 MHz, $CDCl_3$) δ 135.6, 132.5, 131.5, 128.1, 127.93, 127.89, 127.8, 126.5, 125.9 (q, *J* = 273.0 Hz), 35.8 (q, *J* = 31.5 Hz), 25.6 (q, *J* = 1.5 Hz), 14.6 (q, *J* = 3.0 Hz).

^{19}F NMR (564 MHz, $CDCl_3$) δ -69.80 (s).

1-methyl-4-(2-phenyl-1-(trifluoromethyl)cyclopropyl)benzene (47)[23]

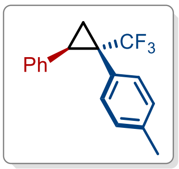

According to **Method D**:

N-triftosylhydrazone derived from 2,2,2-trifluoro-1-(p-tolyl)ethan-1-one (155.8 mg, 0.38 mmol), styrene oxide (18.0 mg, 0.15 mmol), NaH (15.2 mg, 0.38 mmol, 60 wt.% dispersion in mineral oil) and $Tp^{Br3}Ag(thf)$ (8.2 mg, 5 mol%) were reacted in dichloromethane (4.0 mL) for 30 min to afford compound **47** as a white solid (mp: 71-72 °C).

Run: 32.3 mg, 78% yield.

Purification: Silica gel column chromatography (petroleum ether as eluent).

^{1}H NMR (500 MHz, $CDCl_3$) δ 7.13-7.05 (m, 3H), 7.01 (d, *J* = 8.0 Hz, 2H), 6.96 (d, *J* = 8.0 Hz, 2H), 6.82-6.74 (m, 2H), 2.81 (dd, *J* = 8.5, 7.5 Hz, 1H), 2.24 (s, 3H), 1.85 (dd, *J* = 9.5, 6.0 Hz, 1H), 1.64 (t, *J* = 6.0 Hz, 1H).

^{13}C NMR (150 MHz, $CDCl_3$) δ 137.9, 135.8, 132.3, 128.7, 128.4, 128.0, 127.8, 126.4, 126.0 (q, *J* = 273.0 Hz), 35.4 (q, *J* = 31.5 Hz), 25.6, 21.1, 14.7 (q, *J* = 1.5 Hz).

^{19}F NMR (564 MHz, $CDCl_3$) δ -69.94 (s).

IR (Film): 2925, 1607, 1517, 1384, 1341, 1316, 1297, 1285, 1137, 1083, 1049, 811, 725, 695 cm^{-1}.

1-methoxy-4-(2-phenyl-1-(trifluoromethyl)cyclopropyl)benzene (48)[23]

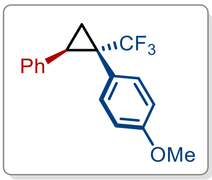

According to **Method D**:

N-triftosylhydrazone derived from 2,2,2-trifluoro-1-(4-methoxyphenyl)ethan-1-one (161.9 mg, 0.38 mmol), styrene oxide (18.0 mg, 0.15 mmol), NaH (15.2 mg, 0.38 mmol, 60 wt.% dispersion in mineral oil) and $Tp^{Br3}Ag(thf)$ (8.2 mg, 5 mol%) were reacted in dichloromethane (4.0 mL) for 1 h to afford compound **48** as a white solid (mp: 55-56 °C).

Run: 36.4 mg, 83% yield.

Purification: Silica gel column chromatography (1% EtOAc in petroleum ether).

^{1}H NMR (500 MHz, $CDCl_3$) δ 7.12-7.06 (m, 3H), 7.04 (d, *J* = 8.5 Hz, 2H), 6.79-6.76 (m, 2H), 6.68 (d, *J* = 9.0 Hz, 2H), 3.71 (s, 3H), 2.80 (dd, *J* = 9.5, 7.0 Hz, 1H), 1.84 (dd, *J* = 9.5, 6.0 Hz, 1H), 1.61 (t, *J* = 6.0 Hz, 1H).

^{13}C NMR (150 MHz, $CDCl_3$) δ 159.3, 135.8, 133.6, 128.0, 127.8, 126.4, 126.0 (q, *J* = 273.0 Hz), 123.5, 113.4, 55.1, 35.0 (q, *J* = 33.0 Hz), 25.6 (q, *J* =1.5 Hz), 14.9 (q, *J* = 3.0 Hz).

^{19}F NMR (564 MHz, $CDCl_3$) δ -70.10 (s).

1-(*tert*-butyl)-4-(2-phenyl-1-(trifluoromethyl)cyclopropyl)benzene (49)

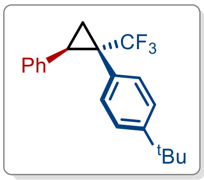

According to **Method D**:

N-triftosylhydrazone derived from 1-(4-(*tert*-butyl)phenyl)-2,2,2-trifluoroethan-1-one (171.8 mg, 0.38 mmol), styrene oxide (18.0 mg, 0.15 mmol), NaH (15.2 mg, 0.38 mmol, 60 wt.% dispersion in mineral oil) and $Tp^{Br3}Ag(thf)$ (8.2 mg, 5 mol%) were reacted in dichloromethane (4.0 mL) for 1 h to afford compound **49** as a colorless oil.

Run: 39.6 mg, 83% yield.

Purification: Silica gel column chromatography (petroleum ether as eluent).

^{1}H NMR (500 MHz, $CDCl_3$) δ 7.16 (d, *J* = 8.5 Hz, 2H), 7.10-7.05 (m, 3H), 7.04 (d, *J* = 8.0 Hz, 2H), 6.78-6.72 (m, 2H), 2.80 (dd, *J* = 9.0, 7.0 Hz, 1H), 1.85 (dd, *J* = 9.5, 6.0 Hz, 1H), 1.63 (t, *J* = 6.0 Hz, 1H), 1.23 (s, 9H).

^{13}C NMR (150 MHz, $CDCl_3$) δ 151.1, 135.9, 132.1, 128.3, 127.9, 127.7, 126.3, 126.0 (q, *J* = 273.0 Hz), 124.8, 35.4 (q, *J* = 31.5 Hz), 34.5, 31.2, 25.6 (q, *J* = 1.5 Hz), 14.9 (q, *J* = 1.5 Hz).

^{19}F NMR (564 MHz, $CDCl_3$) δ -69.82 (s).

IR (Film): 2966, 2906, 2870, 1607, 1516, 1501, 1462, 1397, 1364, 1317, 1279, 1216, 825, 749, 697 cm^{-1}.

HRMS (ESI^+) m/z calcd for $C_{20}H_{20}F_3$ $[M-H]^-$ 317.1523, found 317.1493.

1-(2-phenyl-1-(trifluoromethyl)cyclopropyl)-4-(trifluoromethoxy)benzene (50)

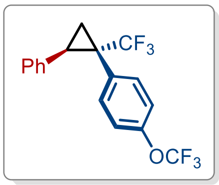

According to **Method D**:

N-triftosylhydrazone derived from 2,2,2-trifluoro-1-(4-(trifluoromethoxy)phenyl)ethan-1-one (182.4 mg, 0.38 mmol), styrene oxide (18.0 mg, 0.15 mmol), NaH (15.2 mg, 0.38 mmol, 60 wt.% dispersion in mineral oil) and $Tp^{Br3}Ag(thf)$ (8.2 mg, 5 mol%) were reacted in dichloromethane (4.0 mL) for 30 min to afford compound **50** as a colorless oil.

Run: 45.7 mg, 88% yield.

Purification: Silica gel column chromatography (petroleum ether as eluent).

^{1}H NMR (500 MHz, $CDCl_3$) δ 7.15 (d, *J* = 8.5 Hz, 2H), 7.12-7.07 (m, 3H), 6.99 (d, *J* = 8.5 Hz, 2H), 6.80-6.72 (m, 2H), 2.86 (dd, *J* = 9.5, 7.0 Hz, 1H), 1.89 (dd, *J* = 9.5, 6.0 Hz, 1H), 1.67 (t, *J* = 6.0 Hz, 1H).

^{13}C NMR (150 MHz, $CDCl_3$) δ 149.1, 135.0, 134.0, 130.3, 128.0, 127.9, 126.8, 125.6 (q, *J* =273.0 Hz), 120.4 (q, *J* =255.0 Hz), 120.3, 35.1 (q, *J* = 33.0 Hz), 25.7 (d, *J* = 1.5 Hz), 14.6 (d, *J* = 3.0 Hz).

^{19}F NMR (564 MHz, $CDCl_3$) δ -57.90 (s), -69.85 (s).

IR (Film): 1512, 1393, 1316, 1262, 1225, 1165, 836, 744, 697 cm^{-1}.

HRMS (ESI^+) m/z calcd for $C_{17}H_{13}F_6O$ $[M+H]^+$ 347.0865, found 347.0861.

1-(2-phenyl-1-(trifluoromethyl)cyclopropyl)-4-(trifluoromethyl)benzene (51)

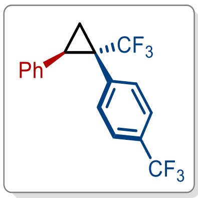

According to **Method D**:

N-triftosylhydrazone derived from 2,2,2-trifluoro-1-(4-(trifluoromethyl)phenyl)ethan-1-one (176.3 mg, 0.38 mmol), styrene oxide (18.0 mg, 0.15 mmol), NaH (15.2 mg, 0.38 mmol, 60 wt.% dispersion in mineral oil) and $Tp^{Br3}Ag(thf)$ (8.2 mg, 5 mol%) were reacted in dichloromethane (4.0 mL) for 30 min to afford compound **51** as a colorless oil.

Run: 42.1 mg, 85% yield.

Purification: Silica gel column chromatography (petroleum ether as eluent).

^{1}H NMR (500 MHz, $CDCl_3$) δ 7.42 (d, *J* = 8.0 Hz, 2H), 7.26 (d, *J* = 8.0 Hz, 2H), 7.14-7.08 (m, 3H), 6.81-6.74 (m, 2H), 2.90 (dd, *J* = 9.5, 7.5 Hz, 1H), 1.93 (dd, *J* = 9.5, 6.5 Hz, 1H), 1.74-1.69 (m, 1H).

^{13}C NMR (125 MHz, $CDCl_3$) δ 135.7, 134.8, 132.9, 130.4 (q, *J* = 31.3 Hz), 128.1, 127.8, 126.9, 125.5 (q, *J* = 272.5 Hz), 124.9 (q, *J* = 3.8 Hz), 123.9 (q, *J* = 271.3 Hz), 35.5 (q, *J* = 32.5 Hz), 25.8 (q, *J* = 2.5 Hz), 14.4 (q, *J* = 2.5 Hz).

^{19}F NMR (564 MHz, $CDCl_3$) δ -62.76 (s), -69.61 (s).

IR (Film): 1326, 1165, 1131, 1068, 832, 717, 697 cm^{-1}.

HRMS (ESI^+) m/z calcd for $C_{17}H_{13}F_6$ $[M+H]^+$ 331.0916, found 331.0909.

1-fluoro-4-(2-phenyl-1-(trifluoromethyl)cyclopropyl)benzene (52)

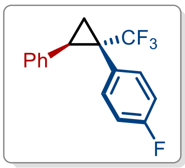

According to **Method D**:

N-triftosylhydrazone derived from 2,2,2-trifluoro-1-(4-fluorophenyl)ethan-1-one (157.3 mg, 0.38 mmol), styrene oxide (18.0 mg, 0.15 mmol), NaH (15.2 mg, 0.38 mmol, 60 wt.% dispersion in mineral oil) and $Tp^{Br3}Ag(thf)$ (8.2 mg, 5 mol%) were reacted in dichloromethane (4.0 mL) for 30 min to afford compound **52** as a light yellow solid (mp: 60-61 °C).

Run: 36.1 mg, 86% yield.

Purification: Silica gel column chromatography (petroleum ether as eluent).

^{1}H NMR (500 MHz, $CDCl_3$) δ 7.15-7.05 (m, 5H), 6.84 (t, *J* = 8.5 Hz, 2H), 6.80-6.73 (m, 2H), 2.84 (dd, *J* = 9.0, *J* = 7.0, 1H), 1.87 (dd, *J* = 9.5, 6.0 Hz, 1H), 1.64 (t, *J* = 6.0 Hz, 1H).

^{13}C NMR (125 MHz, $CDCl_3$) δ 162.5 (d, *J* = 247.2 Hz), 135.3, 134.2 (d, *J* = 8.0 Hz), 128.0, 127.9, 127.4 (d, *J* =3.0 Hz), 126.6, 125.7 (q, *J* = 274.0 Hz), 115.0 (d, *J* = 21.0 Hz), 35.0 (q, *J* = 33.0 Hz), 25.7 (q, *J* =3.0 Hz), 14.7 (q, *J* = 1.5 Hz).

^{19}F NMR (564 MHz, $CDCl_3$) δ -69.99 (s), (-113.26)-(-113.33) (m).

IR (Film): 1606, 1514, 1225, 1161, 830, 698 cm^{-1}.

HRMS (ESI^+) m/z calcd for $C_{16}H_{11}F_4$ $[M-H]^-$ 279.0802, found 279.0815.

1-methyl-3-(2-phenyl-1-(trifluoromethyl)cyclopropyl)benzene (53)

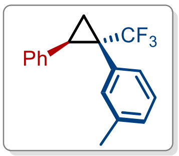

According to **Method D**:

N-triftosylhydrazone derived from 2,2,2-trifluoro-1-(*m*-tolyl)ethan-1-one (155.8 mg, 0.38 mmol), styrene oxide (18.0 mg, 0.15 mmol), NaH (15.2 mg, 0.38 mmol, 60 wt.% dispersion in mineral oil) and $Tp^{Br3}Ag(thf)$ (8.2 mg, 5 mol%) were reacted in dichloromethane (4.0 mL) for 30 min to afford compound **53** as a colorless oil.

Run: 29.4 mg, 71% yield.

Purification: Silica gel column chromatography (petroleum ether as eluent).

^{1}H NMR (500 MHz, $CDCl_3$) δ 7.11-7.05 (m, 3H), 7.04-6.97 (m, 2H), 6.96 (s, 1H), 6.89 (d, J = 7.0 Hz, 1H), 6.81-6.74 (m, 2H), 2.81 (dd, J = 9.5, 7.5 Hz, 1H), 2.20 (s, 3H), 1.85 (dd, J = 9.5, 6.0 Hz, 1H), 1.65 (t, J = 6.0 Hz, 1H).

^{13}C NMR (150 MHz, $CDCl_3$) δ 137.5, 135.8, 133.2, 131.4, 129.6, 128.9, 128.0, 127.8, 127.7, 126.4, 126.0 (q, J = 273.0 Hz), 35.7 (q, J = 33.0 Hz), 25.6 (q, J = 3.0 Hz), 21.2, 14.7 (q, J = 1.5 Hz).

^{19}F NMR (564 MHz, $CDCl_3$) δ -69.77 (s).

IR (Film): 2927, 1608, 1500, 1460, 1386, 1286, 1207, 1152, 1084, 976, 811, 785, 751 cm^{-1}.

HRMS (ESI^+) m/z calcd for $C_{17}H_{14}F_3$ $[M-H]^-$ 275.1053, found 275.1028.

1-fluoro-3-(2-phenyl-1-(trifluoromethyl)cyclopropyl)benzene (54)[23]

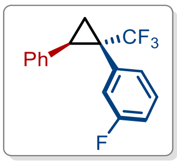

According to **Method D**:

N-triftosylhydrazone derived from 2,2,2-trifluoro-1-(3-fluorophenyl)ethan-1-one (157.3 mg, 0.38 mmol), styrene oxide (18.0 mg, 0.15 mmol), NaH (15.2 mg, 0.38 mmol, 60 wt.% dispersion in mineral oil) and $Tp^{Br3}Ag(thf)$ (8.2 mg, 5 mol%) were reacted in dichloromethane (4.0 mL) for 30 min to afford compound **54** as a colorless oil.

Run: 34.5 mg, 82% yield.

Purification: Silica gel column chromatography (petroleum ether as eluent).

^{1}H NMR (500 MHz, $CDCl_3$) δ 7.15-7.07 (m, 4H), 6.94-6.83 (m, 3H), 6.83-6.76 (m, 2H), 2.86 (dd, *J* = 9.5, 7.5 Hz, 1H), 1.88 (dd, *J* = 9.5, 6.5 Hz, 1H), 1.71-1.66 (m, 1H).

^{13}C NMR (125 MHz, $CDCl_3$) δ 162.2 (d, *J* = 246.0 Hz), 135.0, 133.9 (d, *J* = 9.0 Hz), 129.4 (d, *J* = 9.0 Hz), 128.1 (d, *J* = 3.0 Hz), 128.0, 127.8, 126.7, 125.6 (d, *J* = 274.0 Hz), 119.4 (d, *J* = 22.0 Hz), 115.3 (d, *J* =20.5 Hz), 35.4 (q, *J* = 32.5 Hz), 25.8, 14.4.

^{19}F NMR (564 MHz, $CDCl_3$) δ -69.72 (s), -113.47-(-113.43) (m).

1-fluoro-2-(2-phenyl-1-(trifluoromethyl)cyclopropyl)benzene (55)

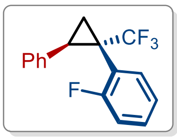

According to **Method D**:

N-triftosylhydrazone derived from 2,2,2-trifluoro-1-(2-fluorophenyl)ethan-1-one (157.3 mg, 0.38 mmol), styrene oxide (18.0 mg, 0.15 mmol), NaH (15.2 mg, 0.38 mmol, 60 wt.% dispersion in mineral oil) and $Tp^{Br3}Ag(thf)$ (8.2 mg, 5 mol%) were reacted in dichloromethane (4.0 mL) for 30 min to afford compound **55** as a light yellow solid (mp: 72-73 °C).

Run: 34.0 mg, 81% yield.

Purification: Silica gel column chromatography (petroleum ether as eluent).

^{1}H NMR (500 MHz, $CDCl_3$) δ 7.23-7.18 (m 1H), 7.15-6.83 (m, 6H), 6.82-6.80 (m, 2H), 2.88 (t, *J* = 8.0, 1H), 1.94 (dd, *J* = 9.0, 6.5 Hz, 1H), 1.69 (t, *J* = 6.5 Hz, 1H).

^{13}C NMR (150 MHz, $CDCl_3$) δ 163.1 (d, *J* = 241.5 Hz), 135.5, 134.7, 130.4 (d, *J* = 7.5 Hz), 127.8, 127.7, 126.7, 125.6 (q, *J* = 273.0 Hz), 123.6, 118.8 (d, *J* = 12.0 Hz), 115.7 (d, *J* = 22.5 Hz), 31.2 (q, *J* = 31.5 Hz), 25.6, 15.0.

^{19}F NMR (564 MHz, $CDCl_3$) δ -70.23 (s), -111.39 (br).

IR (Film): 1584, 1496, 1343, 1344, 1318, 1229, 1154, 1138, 759, 738 cm^{-1}.

HRMS (ESI^+) m/z calcd for $C_{16}H_{11}F_4$ $[M-H]^-$ 279.0802, found 279.0822.

1,3-dimethyl-5-(2-phenyl-1-(trifluoromethyl)cyclopropyl)benzene (56)

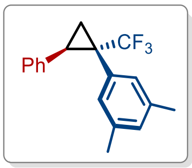

According to **Method D**:

N-triftosylhydrazone derived from 1-(3,5-dimethylphenyl)-2,2,2-trifluoroethan-1-one (161.1 mg, 0.38 mmol), styrene oxide (18.0 mg, 0.15 mmol), NaH (15.2 mg, 0.38 mmol, 60 wt.% dispersion in mineral oil) and $Tp^{Br3}Ag(thf)$ (8.2 mg, 5 mol%) were reacted in dichloromethane (4.0 mL) for 30 min to afford compound **56** as a colorless oil.

Run: 31.8 mg, 73% yield.

Purification: Silica gel column chromatography (petroleum ether as eluent).

^{1}H NMR (500 MHz, $CDCl_3$) δ 7.04-6.96 (m, 3H), 6.75-6.67 (m, 3H), 6.64 (s, 2H), 2.71 (dd, *J* = 9.5, 7.5 Hz, 1H), 2.06 (s, 6H), 1.75 (dd, *J* = 9.5, 6.0 Hz, 1H), 1.55 (t, *J* = 6.0 Hz, 1H).

^{13}C NMR (150 MHz, $CDCl_3$) δ 137.2, 135.9, 131.2, 130.3, 129.7, 128.0, 127.7, 126.4, 126.0 (q, *J* = 271.5 Hz), 35.5 (q, *J* = 33.0 Hz), 25.6 (q, *J* = 3.0 Hz), 21.1, 14.7 (q, *J* = 3.0 Hz).

^{19}F NMR (564 MHz, $CDCl_3$) δ -69.76 (s).

IR (Film): 2924, 1606, 1460, 1386, 1316, 1287, 1266, 1153, 1086, 848, 796, 751, 709 cm^{-1}.

HRMS (ESI^+) m/z calcd for $C_{18}H_{16}F_3$ $[M-H]^-$ 289.1210, found 289.1182.

2-(2-phenyl-1-(trifluoromethyl)cyclopropyl)naphthalene (57)[23]

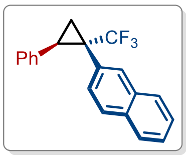

According to **Method D**:

N-triftosylhydrazone derived from 2,2,2-trifluoro-1-(naphthalen-2-yl)ethan-1-one (169.5 mg, 0.38 mmol), styrene oxide (18.0 mg, 0.15 mmol), NaH (15.2 mg, 0.38 mmol, 60 wt.% dispersion in mineral oil) and $Tp^{Br3}Ag(thf)$ (8.2 mg, 5 mol%) were reacted in dichloromethane (4.0 mL) for 1 h to afford compound **57** as a white solid (mp: 83-84 °C).

Run: 36.5 mg, 78% yield.

Purification: Silica gel column chromatography (petroleum ether as eluent).

^{1}H NMR (600 MHz, $CDCl_3$) δ 7.75-7.67 (m, 3H), 7.58 (d, J = 8.4 Hz, 1H), 7.45-7.40 (m, 2H), 7.16 (d, J = 8.4 Hz, 1H), 7.06-7.00 (m, 3H), 6.84-6.78 (m, 2H), 2.94-2.90 (m, 1H), 1.99-1.93 (m, 1H), 1.82-1.78 (m, 1H).

^{13}C NMR (150 MHz, $CDCl_3$) δ 135.5, 132.9, 132.3, 129.5, 129.2, 127.93, 127.90, 127.89, 127.6, 127.5, 126.5, 126.3, 125.98 (q, J = 273.0 Hz), 125.95, 35.9 (q, J = 33.0 Hz), 25.8, 14.9.

^{19}F NMR (564 MHz, $CDCl_3$) δ -69.52 (s).

2-(2-phenyl-1-(trifluoromethyl)cyclopropyl)furan (58)

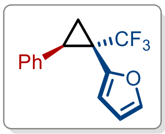

According to **Method D**:

N-triftosylhydrazone derived from 2,2,2-trifluoro-1-(furan-2-yl)ethan-1-one (146.7 mg, 0.38 mmol), styrene oxide (18.0 mg, 0.15 mmol), NaH (15.2 mg, 0.38 mmol, 60 wt.% dispersion in mineral oil) and $Tp^{Br3}Ag(thf)$ (8.2 mg, 5 mol%) were reacted in dichloromethane (4.0 mL) for 6 h to afford compound **58** as a colorless oil.

Run: 20.4 mg, 54% yield.

Purification: Silica gel column chromatography (1% EtOAc in petroleum ether).

^{1}H NMR (600 MHz, $CDCl_3$) δ 7.19 (d, J = 1.2 Hz, 1H), 7.18-7.10 (m, 3H), 7.00-6.85 (m, 2H), 6.16 (dd, J = 3.0, 1.8 Hz, 1H), 6.10 (d, J = 3.0 Hz, 1H), 2.85 (dd, J = 9.6, 7.8 Hz, 1H), 1.93-1.89 (m, 1H), 1.84 (dd, J = 9.6, 6.0 Hz, 1H).

^{13}C NMR (150 MHz, $CDCl_3$) δ 146.1, 142.6, 135.0, 128.0 127.9, 126.9, 125.1 (q, J = 271.5), 112.0, 110.4, 29.5 (q, J = 31.5 Hz), 27.0, 13.8.

^{19}F NMR (564 MHz, $CDCl_3$) δ -69.40 (s).

IR (Film): 2924, 2853, 1498, 1457, 1396, 1331, 1289, 1152, 815, 743, 697 cm^{-1}.

HRMS (ESI^+) m/z calcd for $C_{14}H_{10}F_3O$ $[M-H]^-$ 251.0689, found 251.0663.

(*E*)-1-methoxy-4-(2-(trifluoromethyl)-2-(undec-1-en-1-yl)cyclopropyl)benzene (59)

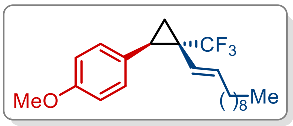

According to **Method D**:

N-triftosylhydrazone derived from (*E*)-1,1,1-trifluorotridec-3-en-2-one (179.4 mg, 0.38 mmol), 2-(4-methoxyphenyl)oxirane (22.5 mg, 0.15 mmol), NaH (15.2 mg, 0.38 mmol, 60 wt.% dispersion in mineral oil) and $Tp^{Br3}Ag(thf)$ (8.2 mg, 5 mol%) were reacted in dichloromethane (4.0 mL) for 1 h to afford compound **59** as a colorless oil.

Run: 46.4 mg, 84% yield.

Purification: Silica gel column chromatography (1% EtOAc in petroleum ether).

^{1}H NMR (500 MHz, $CDCl_3$) δ 7.01 (d, *J* = 8.5 Hz, 2H), 6.79 (d, *J* = 8.5 Hz, 2H), 5.63-5.49 (m, 1H), 5.11 (d, *J* = 15.5 Hz, 1H), 3.78 (s, 3H), 2.61 (t, *J* = 8.5 Hz, 1H), 1.95-1.78 (m, 2H), 1.51 (dd, *J* = 9.5, 6.0 Hz, 1H), 1.36 (t, *J* = 6.0 Hz, 1H), 1.33-1.02 (m, 14H), 0.88 (t, *J* = 7.0 Hz, 3H).

^{13}C NMR (150 MHz, $CDCl_3$) δ 158.3, 138.0, 130.1, 127.3, 126.3 (q, *J* = 271.5 Hz), 120.5, 113.4, 55.2, 32.4, 31.9, 31.2 (q, *J* = 33.0 Hz), 29.5, 29.4, 29.3, 28.9, 28.8, 25.4, 22.7, 14.12 (s), 12.8.

^{19}F NMR (564 MHz, $CDCl_3$) δ -70.13 (s).

HRMS (ESI^+) m/z calcd for $C_{22}H_{30}F_3O$ $[M-H]^-$ 367.2254, found 367.2246.

1-methoxy-4-(2-(2-methylprop-1-en-1-yl)-2-(trifluoromethyl)cyclopropyl)benzene (60)

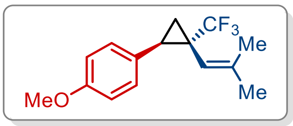

According to **Method D**:

N-triftosylhydrazone derived from 1,1,1-trifluoro-4-methylpent-3-en-2-one (142.2 mg, 0.38 mmol), 2-(4-methoxyphenyl)oxirane (22.5 mg, 0.15 mmol), NaH (15.2 mg, 0.38 mmol, 60 wt.% dispersion in mineral oil) and $Tp^{Br3}Ag(thf)$ (8.2 mg, 5 mol%) were reacted in dichloromethane (4.0 mL) for 1 h to afford compound **60** as a colorless oil.

Run: 30.4 mg, 75% yield.

Purification: Silica gel column chromatography (1% EtOAc in petroleum ether).

^{1}H NMR (500 MHz, $CDCl_3$) δ 6.99 (d, J = 8.5 Hz, 2H), 6.81 (d, J = 8.5 Hz, 2H), 4.80 (s, 1H), 3.79 (s, 3H), 2.51 (dd, J = 9.0, 7.5 Hz, 1H), 1.71-1.64 (m, 4H), 1.61 (s, 3H), 1.18 (t, J = 6.0 Hz, 1H).

^{13}C NMR (150 MHz, $CDCl_3$) δ 158.3, 144.5, 129.3, 127.8, 126.6 (q, J = 271.5 Hz), 115.1, 113.4, 55.2, 28.1 (q, J = 33.0 Hz), 25.5, 24.4, 19.4, 15.9.

^{19}F NMR (564 MHz, $CDCl_3$) δ -70.07 (s).

HRMS (ESI$^+$) m/z calcd for $C_{15}H_{18}F_3O$ $[M+H]^+$ 271.1304, found 271.1279.

(*E*)-(2-(2-phenyl-1-(trifluoromethyl)cyclopropyl)vinyl)benzene (61)

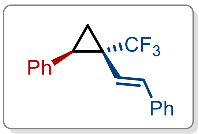

According to **Method D**:

N-triftosylhydrazone derived from (*E*)-1,1,1-trifluoro-4-phenylbut-3-en-2-one (158.3 mg, 0.38 mmol), styrene oxide (18.0 mg, 0.15 mmol), NaH (15.2 mg, 0.38 mmol, 60 wt.% dispersion in mineral oil) and $Tp^{Br3}Ag(thf)$ (8.2 mg, 5 mol%) were reacted in dichloromethane (4.0 mL) for 1 h to afford compound **61** as a colorless oil.

Run: 35.4 mg, 82% yield.

Purification: Silica gel column chromatography (petroleum ether as eluent).

^{1}H NMR (500 MHz, $CDCl_3$) δ 7.28-7.09 (m, 10H), 6.53 (d, *J* = 16.0 Hz, 1H), 5.78 (d, *J* = 16.0 Hz, 1H), 2.81 (dd, *J* = 9.0, 7.5 Hz, 1H), 1.70 (dd, *J* = 9.5, 6.0 Hz, 1H), 1.60-1.55 (m, 1H).

^{13}C NMR (150 MHz, $CDCl_3$) δ 136.4, 135.9, 135.0, 129.0, 128.5, 128.2, 127.8, 126.9, 126.3, 126.1 (q, *J* = 273.0 Hz), 121.0, 31.7 (q, *J* = 31.5 Hz), 26.8, 13.8.

^{19}F NMR (564 MHz, $CDCl_3$) δ -69.61 (s).

IR (Film): 3030, 1391, 1298, 1264, 1144, 966, 739, 695 cm^{-1}.

HRMS (ESI^+) m/z calcd for $C_{18}H_{14}F_3$ $[M-H]^-$ 287.1053, found 287.1026.

1-methoxy-4-(2-((1*E*,3*E*)-4-phenylbuta-1,3-dien-1-yl)-2-(trifluoromethyl)cyclopropyl)benzene (62)

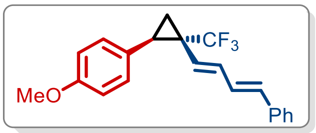

According to **Method D**:

N-triftosylhydrazone derived from (3*E*,5*E*)-1,1,1-trifluoro-6-phenylhexa-3,5-dien-2-one (170.3 mg, 0.38 mmol), 2-(4-methoxyphenyl)oxirane (22.5 mg, 0.15 mmol), NaH (15.2 mg, 0.38 mmol, 60 wt.% dispersion in mineral oil) and $Tp^{Br3}Ag(thf)$ (8.2 mg, 5 mol%) were reacted in dichloromethane (4.0 mL) for 1 h to afford compound **62** as a colorless oil.

Run: 45.9 mg, 89% yield.

Purification: Silica gel column chromatography (1% EtOAc in petroleum ether).

^{1}H NMR (500 MHz, $CDCl_3$) δ 7.31 (d, *J* = 7.5 Hz, 2H), 7.26 (t, *J* = 7.5 Hz, 2H), 7.19 (t, *J* = 7.5 Hz, 1H), 7.05 (d, *J* = 8.5 Hz, 2H), 6.81 (d, *J* = 8.5 Hz, 2H), 6.56 (dd, *J* = 15.5, 10.5 Hz, 1H), 6.45 (d, *J* = 15.5 Hz, 1H), 6.34 (dd, *J* = 15.5, 10.5 Hz, 1H), 5.34 (d, *J* = 15.4 Hz, 1H), 3.76 (s, 3H), 2.74 (t, *J* = 8.5 Hz, 1H), 1.65 (dd, *J* = 9.0, 6.0 Hz, 1H), 1.44 (t, *J* = 6.5 Hz, 1H).

^{13}C NMR (125 MHz, $CDCl_3$) δ 158.5, 136.9, 135.5, 132.8, 130.0, 128.6, 128.1, 127.6, 126.9, 126.4, 126.1 (q, *J* = 272.5 Hz), 124.9, 113.7, 55.2, 31.4 (q, *J* = 32.5 Hz), 26.5, 14.2.

^{19}F NMR (470 MHz, $CDCl_3$) δ -69.32 (s).

HRMS (ESI^+) m/z calcd for $C_{21}H_{20}F_3O$ $[M+H]^+$ 345.1461, found 345.1428.

1-chloro-4-(1-(perfluoroethyl)-2-phenylcyclopropyl)benzene (63)[23]

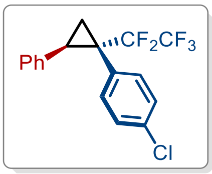

According to **Method D**:

N-triftosylhydrazone derived from 1-(4-chlorophenyl)-2,2,3,3,3-pentafluoropropan-1-one (182.4 mg, 0.38 mmol), styrene oxide (18.0 mg, 0.15 mmol), NaH (15.2 mg, 0.38 mmol, 60 wt.% dispersion in mineral oil) and $Tp^{Br3}Ag(thf)$ (8.2 mg, 5 mol%) were reacted in dichloromethane (4.0 mL) for 2 h to afford compound **63** as a colorless oil.

Run: 38.4 mg, 74% yield.

Purification: Silica gel column chromatography (petroleum ether as eluent).

^{1}H NMR (500 MHz, $CDCl_3$) δ 7.14-7.07 (m, 5H), 7.01 (d, J = 8.5 Hz, 2H), 6.82-6.75 (m, 2H), 2.96 (dd, J = 9.5, 7.5 Hz, 1H), 1.90 (dd, J = 9.5, 7.0 Hz, 1H), 1.77-1.70 (m, 1H).

^{13}C NMR (150 MHz, $CDCl_3$) δ 135.0, 134.2, 134.0, 130.1 (d, J =3.0 Hz), 128.1, 128.0, 126.8, 119.3 (qt, J = 287.0, 38.0 Hz), 114.4 (qt, J = 255.0, 335.8 Hz), 33.1 (t, J = 23.2 Hz), 25.0 (d, J =4.5 Hz), 14.8 (d, J = 4.5 Hz).

^{19}F NMR (564 MHz, $CDCl_3$) δ -80.09 (s), -115.47 (d, J = 265.1 Hz), -119.72 (d, J = 265.1 Hz).

ethyl 1,2-diphenylcyclopropane-1-carboxylate (64)[25]

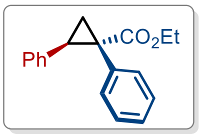

According to **Method D**:

N-triftosylhydrazone derived from ethyl 2-oxo-2-phenylacetate (150.0 mg, 0.38 mmol), styrene oxide (18.0 mg, 0.15 mmol), NaH (15.2 mg, 0.38 mmol, 60 wt.% dispersion in mineral oil) and $Tp^{Br3}Ag(thf)$ (8.2 mg, 5 mol%) were reacted in dichloromethane (4.0 mL) for 2 h to afford compound **64** as a colorless oil.

Run: 27.1 mg, 68% yield.

Purification: Silica gel column chromatography (2% EtOAc in petroleum ether).

^{1}H NMR (500 MHz, $CDCl_3$) δ 7.14-7.08 (m, 3H), 7.08-6.98 (m, 5H), 6.82-6.71 (m, 2H), 4.24-4.04 (m, 2H), 3.09 (dd, *J* = 9.0, 7.5 Hz, 1H), 2.13 (dd, *J* = 9.5, 5.0 Hz, 1H), 1.87 (dd, *J* = 7.0, 5.0 Hz, 1H), 1.18 (t, *J* = 7.0 Hz, 3H).

^{13}C NMR (150 MHz, $CDCl_3$) δ 173.7, 136.5, 134.8, 131.9, 128.0, 127.64, 127.55, 126.9, 126.2, 61.2, 37.6, 32.9, 20.1, 14.1.

1,2-diphenylcyclopropane (65)[24]

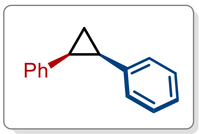

According to **Method E**:

N-triftosylhydrazone derived from benzaldehyde (147.6 mg, 0.45 mmol), styrene oxide (18.0 mg, 0.15 mmol), NaH (18.0 mg, 0.45 mmol, 60 wt.% dispersion in mineral oil) and $Tp^{Br3}Ag(thf)$ (8.2 mg, 5 mol%) were reacted in dichloromethane (4.0 mL) for 10 h to afford compound **65** as a colorless oil. The product was obtained as a mixture of stereoisomers (6.5:1) determined by NMR.

Run: 21.8 mg, 75% yield.

Purification: Silica gel column chromatography (petroleum ether as eluent).

60-*syn*:

^{1}H NMR (500 MHz, $CDCl_3$) δ 7.08 (t, *J* = 7.0 Hz, 4H), 7.03 (t, *J* = 7.0 Hz, 2H), 6.94 (d, *J* = 7.5 Hz, 4H), 2.48 (dd, *J* = 8.5, 6.5 Hz, 2H), 1.49-1.43 (m, 1H), 1.37 (q, *J* = 6.0 Hz, 1H).

^{13}C NMR (150 MHz, $CDCl_3$) δ 138.4, 129.0, 127.6, 125.6, 24.3, 11.4.

1-chloro-4-(2-phenylcyclopropyl)benzene (66)[24]

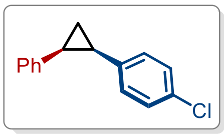

According to **Method E**:

N-triftosylhydrazone derived from 4-chlorobenzaldehyde (162.9 mg, 0.45 mmol), styrene oxide (18.0 mg, 0.15 mmol), NaH (18.0 mg, 0.45 mmol, 60 wt.% dispersion in mineral oil) and $Tp^{Br3}Ag(thf)$ (8.2 mg, 5 mol%) were reacted in dichloromethane (4.0 mL) for 10 h to afford compound **66** as a colorless oil. The product was obtained as a mixture of stereoisomers (4:1) determined by NMR.

Run: 28.7 mg, 84% yield.

Purification: Silica gel column chromatography (petroleum ether as eluent).

61-*syn*:

^{1}H NMR (500 MHz, $CDCl_3$) δ 7.14-7.09 (m, 2H), 7.09-7.03 (m, 3H), 6.94 (d, *J* = 7.0 Hz, 2H), 6.86 (d, *J* = 8.5 Hz, 2H), 2.55-2.47 (m, 1H), 2.46-2.39 (m, 1H), 1.48 (td, *J* = 8.5, 5.5 Hz, 1H), 1.36-1.32 (m, 1H).

^{13}C NMR (150 MHz, $CDCl_3$) δ 137.8, 137.0, 131.3, 130.2, 129.0, 127.78, 127.75, 125.8, 24.4, 23.6, 11.4.

1-(2-phenylcyclopropyl)-4-(trifluoromethyl)benzene (67)[24]

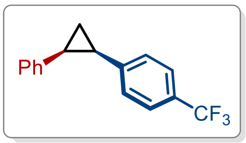

According to **Method E**:

N-triftosylhydrazone derived from 4-(trifluoromethyl)benzaldehyde (178.2 mg, 0.45 mmol), styrene oxide (18.0 mg, 0.15 mmol), NaH (18.0 mg, 0.45 mmol, 60 wt.% dispersion in mineral oil) and $Tp^{Br3}Ag(thf)$ (8.2 mg, 5 mol%) were reacted in dichloromethane (4.0 mL) for 10 h to afford compound **67** as a colorless oil. The product was obtained as a mixture of stereoisomers (3.5:1) determined by NMR.

Run: 25.6 mg, 65% yield.

Purification: Silica gel column chromatography (petroleum ether as eluent).

62-*syn*-:

^{1}H NMR (500 MHz, $CDCl_3$) δ 7.32 (d, *J* = 8.0 Hz, 2H), 7.16-7.09 (m, 3H), 6.99 (d, *J* = 8.0 Hz, 2H), 6.95 (d, *J* = 7.0 Hz, 2H), 2.62-2.55 (m, 1H), 2.52-2.45 (m, 1H), 1.55-1.50 (m, 1H), 1.43-1.39 (m, 1H).

^{13}C NMR (150 MHz, $CDCl_3$) δ 143.0, 137.4, 129.2, 128.8, 127.9, 127.7 (q, *J* = 33.0 Hz), 126.0, 124.5 (q, *J* = 3.0 Hz), 124.3 (q, *J* = 270.0 Hz), 25.0, 23.9, 11.8.

^{19}F NMR (564 MHz, $CDCl_3$) δ -62.29 (s).

62-*anti*-:

^{1}H NMR (500 MHz, $CDCl_3$) δ 7.53 (d, *J* = 8.0 Hz, 2H), 7.29 (t, *J* = 8.0 Hz, 2H), 7.24-7.18 (m, 3H), 7.09-7.04 (m, 2H), 2.20 (t, *J* = 7.5 Hz, 2H), 1.53-1.46 (m, 2H).

^{13}C NMR (150 MHz, $CDCl_3$) δ 146.8, 141.7, 128.5, 128.0 (q, *J* = 32.1 Hz), 126.1, 125.9, 125.8, 125.3 (q, *J* = 3.0 Hz), 124.5 (q, *J* = 272.2 Hz), 28.7, 27.7, 18.5.

^{19}F NMR (564 MHz, $CDCl_3$) δ -62.26 (s).

(*Z*)-1-chloro-4-(2-(4-methyl-1-phenylpent-1-en-1-yl)-1-(trifluoromethyl)cyclopropyl)benzene (68)

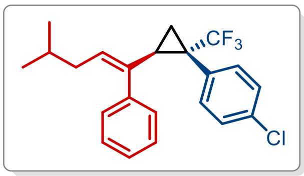

According to **Method D**:

N-triftosylhydrazone derived from 1-(4-chlorophenyl)-2,2,2-trifluoroethan-1-one (161.2 mg, 0.38 mmol), 2-(4-methyl-1-phenylpent-1-en-1-yl)oxirane (30.2 mg, 0.15 mmol), NaH (15.2 mg, 0.38 mmol, 60 wt.% dispersion in mineral oil) and $Tp^{Br3}Ag(thf)$ (8.2 mg, 5 mol%) were reacted in dichloromethane (4.0 mL) for 2 h to afford compound **68** as a colorless oil. The product was obtained as a mixture of stereoisomers (8:1) determined by NMR.

Run: 48.2 mg, 85% yield.

Purification: Silica gel column chromatography (petroleum ether as eluent).

Major isomer:

^{1}H NMR (500 MHz, $CDCl_3$) δ 7.27-7.20 (m, 5H), 7.12 (d, *J* = 8.0 Hz, 2H), 6.86-6.82 (m, 2H), 5.25 (t, *J* = 7.5 Hz, 1H), 2.62-2.57 (m, 1H), 1.77-1.67 (m, 2H), 1.63 (dd, *J* = 9.5, 6.0 Hz, 1H), 1.40-1.37 (m, 1H), 0.86 (dd, *J* = 9.5, 6.5 Hz, 1H), 0.67 (d, *J* = 6.5 Hz, 3H), 0.61 (d, *J* = 7.0 Hz, 3H).

^{13}C NMR (150 MHz, $CDCl_3$) δ 139.7, 134.2, 134.1, 133.4, 130.6, 128.8, 128.5 (d, *J* = 4.5 Hz), 128.1, 127.8, 126.6, 125.8 (q, *J* = 273.0 Hz), 37.8, 34.1 (q, *J* = 31.5 Hz), 28.8, 28.2, 22.1, 22.1, 13.4.

^{19}F NMR (564 MHz, $CDCl_3$) δ -69.15 (s).

HRMS (ESI^+) m/z calcd for $C_{22}H_{23}ClF_3$ $[M+H]^+$ 379.1435, found 379.1473.

methyl 3-(1-([1,1'-biphenyl]-4-yl)-2-(4-chlorophenyl)-2-(trifluoromethyl)cyclopropyl)propanoate (69)

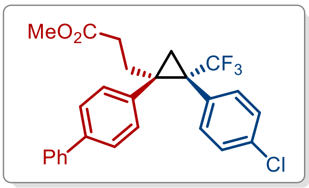

According to **Method D**:

N-triftosylhydrazone derived from 1-(4-chlorophenyl)-2,2,2-trifluoroethan-1-one (161.2 mg, 0.38 mmol), methyl 3-(2-([1,1'-biphenyl]-4-yl)oxiran-2-yl)propanoate (42.3 mg, 0.15 mmol), NaH (15.2 mg, 0.38 mmol, 60 wt.% dispersion in mineral oil) and $Tp^{Br3}Ag(thf)$ (8.2 mg, 5 mol%) were reacted in dichloromethane (4.0 mL) for 4 h to afford compound **69** as a white solid (mp: 135-136 °C).

Run: 64.6 mg, 94% yield.

Purification: Silica gel column chromatography (5% EtOAc in petroleum ether).

^{1}H NMR (500 MHz, $CDCl_3$) δ 7.50-7.45 (m, 2H), 7.38 (t, *J* = 7.0 Hz, 2H), 7.34 (d, *J* = 8.0 Hz, 2H), 7.30 (t, *J* = 7.5 Hz, 1H), 7.21-7.07 (m, 4H), 7.04 (d, *J* = 9.0 Hz, 2H), 3.61 (s, 3H), 2.72-2.61 (m, 1H), 2.33-2.18 (m, 3H), 2.13-2.07 (m, 1H), 1.78 (d, *J* = 6.0 Hz, 1H).

^{13}C NMR (150 MHz, $CDCl_3$) δ 173.3, 140.2, 139.6, 136.9, 133.5, 132.4, 131.9, 129.4, 128.7, 128.1, 127.3, 126.9, 126.8, 126.3 (q, *J* = 274.5 Hz), 51.6, 38.7 (q, *J* = 31.5 Hz), 36.5, 32.3, 29.2, 18.6.

^{19}F NMR (564 MHz, $CDCl_3$) δ -58.18 (s).

IR (Film): 2952, 1737, 1494, 1466, 1258, 1153, 1133, 1094, 1013, 753, 737 cm^{-1}.

HRMS (ESI^+) m/z calcd for $C_{26}H_{21}ClF_3O_2$ $[M-H]^-$ 457.1188, found 457.1145.

4-(2-(2-(4-chlorophenyl)-1-methyl-2-(trifluoromethyl)cyclopropyl)ethyl)-1,2-dimethoxybenzene (70)

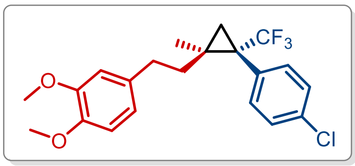

According to **Method C**:

N-triftosylhydrazone derived from 1-(4-chlorophenyl)-2,2,2-trifluoroethan-1-one (258.0 mg, 0.6 mmol), 2-(3,4-dimethoxyphenethyl)-2-methyloxirane (33.3 mg, 0.15 mmol), NaH (27.0 mg, 0.68 mmol, 60 wt.% dispersion in mineral oil) and $Tp^{Br3}Ag(thf)$ (16.4 mg, 10 mol%) were reacted in dichloromethane (4.0 mL) for 10 h to afford compound **70** as a colorless oil. **70-*anti*/70-*syn*** = 1.2/1.

Run: 46.0 mg, 77% yield.

Purification: Silica gel column chromatography (4% EtOAc in petroleum ether).

67-*syn*:

^{1}H NMR (500 MHz, $CDCl_3$) δ 7.38-7.29 (m, 3H), 7.22-7.14 (m, 2H), 6.81 (d, *J* = 8.0 Hz, 1H), 6.74-6.72 (m, 1H), 3.88 (s, 3H), 3.86 (s, 3H), 2.84-2.71 (m, 2H), 1.98-1.90 (m, 2H), 1.43 (d, *J* = 6.0 Hz, 1H), 1.10-1.08 (m, 1H), 0.85 (s, 3H).

^{13}C NMR (150 MHz, $CDCl_3$) δ 148.8, 147.3, 134.7, 133.9, 132.2, 132.0, 128.7, 126.7 (q, *J* = 273.0 Hz), 119.9, 111.4, 111.1, 55.9, 55.9, 40.1, 37.1 (q, *J* = 31.5 Hz), 32.0, 26.2, 22.7, 17.2.

^{19}F NMR (564 MHz, $CDCl_3$) δ -59.86 (s).

67-*anti*:

^{1}H NMR (500 MHz, $CDCl_3$) δ 7.47 (d, *J* = 8.0 Hz, 1H), 7.38-7.29 (m, 2H), 6.78-6.71 (m, 1H), 6.69 (d, *J* = 8.0 Hz, 1H), 6.47 (dd, *J* = 8.5, 2.0 Hz, 1H), 6.30 (d, *J* = 1.5 Hz, 1H), 3.81 (s, 3H), 3.76 (s, 3H), 2.70-2.60 (m, 1H), 2.53-2.43 (m, 1H), 1.48 (s, 3H), 1.39 (d, *J* = 5.5 Hz, 1H), 1.34-1.25 (m, 1H), 1.13-1.10 (m, 1H), 1.08-1.02 (m, 1H).

^{13}C NMR (150 MHz, $CDCl_3$) δ 148.9, 147.2, 134.3, 133.8, 132.8, 132.1, 128.3, 126.6 (q, *J* = 274.5 Hz), 120.1, 111.7, 111.3, 55.8, 55.7, 36.7 (q, *J* = 31.5 Hz), 36.1, 33.0, 26.9, 21.8, 18.4.

^{19}F NMR (564 MHz, $CDCl_3$) δ -59.70 (s).

mixture:

IR (Film): 2935, 1590, 1516, 1464, 1263, 1236, 1156, 1142, 1029, 806 cm^{-1}.

HRMS (ESI^+) m/z calcd for $C_{21}H_{23}ClF_3O_2$ $[M+H]^+$ 399.1333, found 399.1350.

2-(2-(2-(4-chlorophenyl)-1-methyl-2-(trifluoromethyl)cyclopropyl)ethyl)-6-methoxynaphthalene (71)

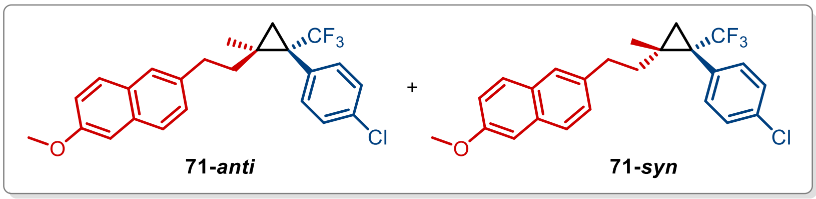

According to **Method C**:

N-triftosylhydrazone derived from 1-(4-chlorophenyl)-2,2,2-trifluoroethan-1-one (258.0 mg, 0.6 mmol), 2-(2-(6-methoxynaphthalen-2-yl)ethyl)-2-methyloxirane (36.3 mg, 0.15 mmol), NaH (27.0 mg, 0.68 mmol, 60 wt.% dispersion in mineral oil) and $Tp^{Br3}Ag(thf)$ (16.4 mg, 10 mol%) were reacted in dichloromethane (4.0 mL) for 10 h to afford compound **71** as a colorless oil. **71-*anti***/**71-*syn*** = 1/1.

Run: 43.3 mg, 69% yield.

Purification: Silica gel column chromatography (1% EtOAc in petroleum ether).

68-*anti*-:

^{1}H NMR (500 MHz, $CDCl_3$) δ 7.61-7.47 (m, 3H), 7.37-7.28 (m, 3H), 7.22 (d, *J* = 6.0 Hz, 1H), 7.07 (d, *J* = 9.0 Hz, 1H), 7.04 (s, 1H), 6.95 (d, *J* = 8.5 Hz, 1H), 3.87 (s, 3H), 2.86 (td, *J* = 13.0, 4.5 Hz, 1H), 2.65 (td, *J* = 12.0, 6.0 Hz, 1H), 1.53 (s, 3H), 1.50-1.42 (m, 1H), 1.39 (d, *J* = 5.5 Hz, 1H), 1.16 (d, *J* = 3.5 Hz, 1H), 1.08 (td, *J* = 13.0, 6.0 Hz, 1H).

^{13}C NMR (125 MHz, $CDCl_3$) δ 157.2, 137.1, 135.0, 132.9, 131.0, 130.6, 129.0, 128.8, 128.6, 128.1, 127.9, 127.5, 126.9 (q, *J* = 273.8 Hz), 126.7, 126.1, 118.7, 105.6, 55.3, 39.9, 37.7 (q, *J* = 31.3 Hz), 32.5, 26.1, 21.7 (q, *J* = 2.5 Hz), 17.4 (q, *J* = 2.5 Hz).

^{19}F NMR (470 MHz, $CDCl_3$) δ -59.61 (s).

68-*syn*-:

^{1}H NMR (500 MHz, $CDCl_3$) δ 7.69 (d, *J* = 8.5 Hz, 2H), 7.58 (s, 1H), 7.45 (d, *J* = 6.5 Hz, 1H), 7.39-7.29 (m, 3H), 7.26 (s, 1H), 7.13 (d, *J* = 8.0 Hz, 2H), 3.92 (s, 3H), 3.06-2.90 (m, 2H), 2.18-2.00 (m, 2H), 1.44 (d, *J* = 5.5 Hz, 1H), 1.18-1.10 (m, 1H), 0.88 (s, 3H).

^{13}C NMR (125 MHz, $CDCl_3$) δ 157.2, 137.4, 135.3, 133.0, 131.6, 130.7, 129.1, 128.9, 128.5, 128.0, 127.8, 127.7, 127.0 (q, *J* = 273.8 Hz), 126.8, 126.2, 118.7, 105.7, 55.3, 37.4 (q, *J* = 32.5 Hz), 36.1 (q, *J* = 2.5 Hz), 33.4, 26.8, 22.8, 22.5 (q, *J* = 2.5 Hz).

^{19}F NMR (470 MHz, $CDCl_3$) δ -59.73 (s).

mixture:

IR (Film): 2936, 1607, 1505, 1484, 1416, 1334, 1267, 1231, 1151, 1126, 851, 702 cm^{-1}.

HRMS (ESI^+) m/z calcd for $C_{24}H_{21}ClF_3O$ $[M-H]^-$ 417.1239, found 417.1241.

isopropyl 2-(4-(1,2-bis(4-chlorophenyl)-2-(trifluoromethyl)cyclopropyl)phenoxy)-2-methylpropanoate (72)

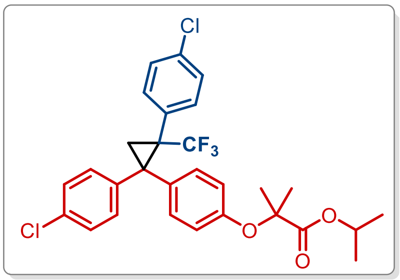

According to **Method D**:

N-triftosylhydrazone derived from 1-(4-chlorophenyl)-2,2,2-trifluoroethan-1-one (161.2 mg, 0.38 mmol), isopropyl 2-(4-(2-(4-chlorophenyl)oxiran-2-yl)phenoxy)-2-methylpropanoate (56.1 mg, 0.15 mmol), NaH (15.2 mg, 0.38 mmol, 60 wt.% dispersion in mineral oil) and $Tp^{Br3}Ag(thf)$ (16.4 mg, 10 mol%) were reacted in dichloromethane (4.0 mL) for 6 h to afford compound **72** as a colorless oil. The product was obtained as a mixture of stereoisomers (1:1) determined by NMR.

Run: 74.3 mg, 90% yield.

Purification: Silica gel column chromatography (5% EtOAc in petroleum ether).

69-*syn*:

^{1}H NMR (500 MHz, $CDCl_3$) δ 7.49 (d, *J* = 8.0 Hz, 2H), 7.31 (d, *J* = 8.5 Hz, 2H), 7.27-7.20 (m, 4H), 7.11 (d, *J* = 8.5 Hz, 2H), 6.89 (d, *J* = 8.5 Hz, 2H), 5.00-4.92 (m, 1H), 2.18 (dd, *J* = 19.0, 6.0 Hz, 2H), 1.44 (d, *J* = 2.5 Hz, 6H), 1.10 (d, *J* = 6.5 Hz, 3H), 1.04 (d, *J* = 6.5 Hz, 3H).

^{13}C NMR (150 MHz, $CDCl_3$) δ 173.5, 154.1, 139.4, 133.9, 132.9, 132.7, 132.3, 131.0, 13.0, 129.3, 128.6, 128.0, 125.7 (q, *J* = 273.0 Hz), 118.1, 78.9, 68.9, 40.9, 39.7 (q, *J* = 31.5 Hz), 25.3, 25.0, 21.40, 21.37, 19.5.

^{19}F NMR (564 MHz, $CDCl_3$) δ -61.62 (s).

69-*anti*:

^{1}H NMR (500 MHz, $CDCl_3$) δ 7.40 (d, *J* = 8.5 Hz, 2H), 7.16 (d, *J* = 9.0 Hz, 2H), 6.99-6.93 (m, 4H), 6.81 (d, *J* = 8.0 Hz, 2H), 6.49 (d, *J* = 9.0 Hz, 2H), 5.10-5.00 (m, 1H), 2.29 (dd, *J* = 20.5, 6.0 Hz, 2H), 1.59 (d, *J* = 2.0 Hz, 6H), 1.16 (d, *J* = 6.0 Hz, 3H), 1.15 (d, *J* = 6.0 Hz, 3H).

^{13}C NMR (150 MHz, $CDCl_3$) δ 173.6, 154.8, 138.6, 134.1, 133.3, 132.7, 131.4, 130.3, 128.3, 128.2, 125.6 (q, *J* = 273.0 Hz), 118.5, 79.1, 68.9, 40.8, 39.6 (q, *J* = 31.5 Hz), 25.5, 25.2, 21.4, 21.3, 19.8.

^{19}F NMR (564 MHz, $CDCl_3$) δ -61.70 (s).

IR (Film): 2983, 1729, 1656, 1599, 1508, 1492, 1384, 1287, 1179, 1149, 1103, 1015, 973, 928, 826, 764 cm^{-1}.

HRMS (ESI^+) m/z calcd for $C_{29}H_{26}Cl_2F_3O_3$ $[M-H]^-$ 549.1217, found 549.1264.

(2-phenyl-2-(trifluoromethyl)cyclopropyl)methyl 4-(*N*,*N*-dipropylsulfamoyl)benzoate (73)

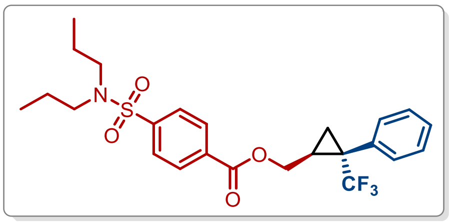

According to **Method C**:

N-triftosylhydrazone derived from 1-(4-chlorophenyl)-2,2,2-trifluoroethan-1-one (258.0 mg, 0.6 mmol), oxiran-2-ylmethyl 4-(*N*,*N*-dipropylsulfamoyl)benzoate (51.2 mg, 0.15 mmol), NaH (27.0 mg, 0.68 mmol, 60 wt.% dispersion in mineral oil) and $Tp^{Br3}Ag(thf)$ (16.4 mg, 10 mol%) were reacted in dichloromethane (4.0 mL) for 10 h to afford compound **73** as a white solid (mp: 110-111 °C).

Run: 48.9 mg, 63% yield.

Purification: Silica gel column chromatography (10% EtOAc in petroleum ether).

^{1}H NMR (500 MHz, $CDCl_3$) δ 8.12 (d, *J* = 8.5 Hz, 2H), 7.90 (d, *J* = 8.5 Hz, 2H), 7.50-7.42 (m, 2H), 7.38-7.31 (m, 3H), 4.14 (dd, *J* = 11.5, 6.5 Hz, 1H), 3.95 (dd, *J* = 11.5, 7.5 Hz, 1H), 3.16-3.04 (m, 4H), 2.15-2.07 (m, 1H), 1.65 (dd, *J* = 9.5, 6.0 Hz, 1H), 1.61-1.51 (m, 4H), 1.23 (t, *J* = 5.5 Hz, 1H), 0.88 (t, *J* = 7.5 Hz, 6H).

^{13}C NMR (150 MHz, $CDCl_3$) δ 164.9, 144.5, 133.1, 131.7, 131.5, 130.2, 128.9, 128.6, 127.1, 125.7 (q, *J* = 273.0 Hz), 64.8, 50.0, 33.1 (q, *J* = 31.5 Hz), 22.0, 19.2, 12.9, 11.1.

^{19}F NMR (470 MHz, $CDCl_3$) δ -70.07 (s).

IR (Film): 2966, 2933, 2877, 1726, 1345, 1270, 1157, 1136, 992, 741, 702, 603 cm^{-1}.

HRMS (ESI^+) m/z calcd for $C_{24}H_{29}F_3NO_4S$ $[M+H]^+$ 484.1764, found 484.1765.

3,7-dimethyloct-6-en-1-yl 4-(2-phenyl-1-(trifluoromethyl)cyclopropyl)benzoate (74)

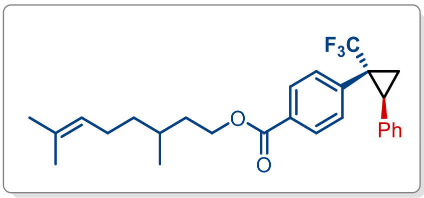

According to **Method D**:

N-triftosylhydrazone derived from citronellol (216.8 mg, 0.38 mmol), styrene oxide (18.0 mg, 0.15 mmol), NaH (15.2 mg, 0.38 mmol, 60 wt.% dispersion in mineral oil) and $Tp^{Br3}Ag(thf)$ (8.2 mg, 5 mol%) were reacted in dichloromethane (4.0 mL) for 2 h to afford compound **74** as a white solid (mp: 98-99 °C).

Run: 34.7 mg, 52% yield.

Purification: Silica gel column chromatography (5% EtOAc in petroleum ether).

^{1}H NMR (500 MHz, $CDCl_3$) δ 7.83 (d, *J* = 8.0 Hz, 2H), 7.21 (d, *J* = 8.0 Hz, 2H), 7.12-7.04 (m, 3H), 6.81-6.75 (m, 2H), 5.08 (t, *J* = 7.0 Hz, 1H), 4.38-4.23 (m, 2H), 2.89 (dd, *J* = 9.5, 7.5 Hz, 1H), 2.07-1.94 (m, 2H), 1.92 (dd, *J* = 9.5, 6.0 Hz, 1H), 1.81-1.70 (m, 2H), 1.60-1.55 (m, 3H), 1.42-1.34 (m, 1H), 1.34-1.26 (m, 1H), 1.25-1.18 (m, 1H), 1.18-1.07 (m, 1H), 0.94 (d, *J* = 6.5 Hz, 3H), 0.86 (t, *J* = 7.0 Hz, 3H).

^{13}C NMR (150 MHz, $CDCl_3$) δ 166.2, 136.5, 135.0, 132.5, 131.4, 130.3, 129.1, 128.0, 127.8, 126.7, 125.6 (q, *J* = 273.0 Hz), 124.5, 63.6, 36.9, 35.6 (q, *J* = 31.5 Hz), 35.5, 29.5 (d, *J* = 1.5 Hz), 25.8 (d, *J* = 1.5 Hz), 25.7, 25.4, 19.5 (d, *J* = 3.0 Hz), 17.6, 14.4 (d, *J* = 1.5 Hz).

^{19}F NMR (564 MHz, $CDCl_3$) δ -69.51 (s).

IR (Film): 2962, 2924, 1721, 1274, 1152, 1139, 750, 707 cm^{-1}.

HRMS (ESI^+) m/z calcd for $C_{27}H_{32}F_3O_2$ $[M+H]^+$ 445.2349, found 445.2312.

(3*S*,8*S*,9*S*,10*R*,13*R*,14*S*,17*R*)-10,13-dimethyl-17-((R)-6-methylheptan-2-yl)-2,3,4,7,8,9,10,11,12,13,14,15,16, 17-tetradecahydro-1*H*-cyclopenta[a]phenanthren-3-yl 4-(2-phenyl-1-(trifluoromethyl)cyclopropyl)benzoate (75)

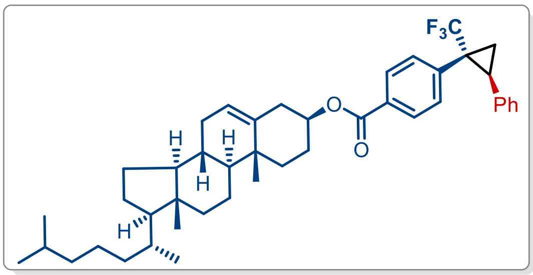

According to **Method D**:

N-triftosylhydrazone derived from cholesterol (307.2 mg, 0.38 mmol), styrene oxide (18.0 mg, 0.15 mmol), NaH (15.2 mg, 0.38 mmol, 60 wt.% dispersion in mineral oil) and $Tp^{Br3}Ag(thf)$ (8.2 mg, 5 mol%) were reacted in dichloromethane (4.0 mL) for 2 h to afford compound **75** as a white solid (mp: 188-189 °C).

Run: 72.8 mg, 72% yield.

Purification: Silica gel column chromatography (5% EtOAc in petroleum ether).

^{1}H NMR (600 MHz, $CDCl_3$) δ 7.83 (d, *J* = 8.4 Hz, 2H), 7.21 (d, *J* = 8.4 Hz, 2H), 7.14-7.04 (m, 3H), 6.82-6.74 (m, 2H), 5.39 (d, *J* = 4.2 Hz, 1H), 4.85-4.75 (m, 1H), 2.88 (dd, *J* = 9.6, 7.2 Hz, 1H), 2.41 (d, *J* = 7.8 Hz, 2H), 2.05-1.87 (m, 5H), 1.86-1.80 (m, 1H), 1.72 (t, *J* = 6.6 Hz, 1H), 1.70-1.64 (m, 1H), 1.62-1.44 (m, 6H), 1.39-1.31 (m, 3H), 1.30-1.23 (m, 2H), 1.22-1.14 (m, 3H), 1.13-1.06 (m, 3H), 1.04 (s, 3H), 1.02-0.95 (m, 3H), 0.92 (d, *J* = 6.6 Hz, 3H), 0.87 (d, *J* = 2.4 Hz, 3H), 0.86 (d, *J* = 2.4 Hz, 3H), 0.68 (s, 3H).

^{13}C NMR (150 MHz, $CDCl_3$) δ 165.6, 139.6, 136.4, 135.0, 132.4, 130.6, 129.2, 128.0, 127.8, 126.7, 125.6 (q, *J* = 33.0 Hz), 122.8, 74.7, 56.7, 56.1, 50.0, 42.3, 39.7, 39.5, 38.2, 37.0, 36.6, 36.2, 35.8, 35.7 (q, *J* = 33.0 Hz), 31.9, 31.9, 28.2, 28.0, 27.8, 25.8, 24.3, 23.8, 22.8, 22.6, 21.0, 19.3, 18.7, 14.4, 11.9.

^{19}F NMR (564 MHz, $CDCl_3$) δ -69.51 (s).

IR (Film): 2947, 2868, 1719, 1465, 1274, 1153, 1139, 1117, 774, 707, 697 cm^{-1}.

HRMS (ESI^+) m/z calcd for $C_{44}H_{58}F_3O_2$ $[M+H]^+$ 675.4383, found 675.4387.

(1*R*,2*S*,5*R*)-2-isopropyl-5-methylcyclohexyl 4-(2-phenyl-1-(trifluoromethyl)cyclopropyl)benzoate (76)

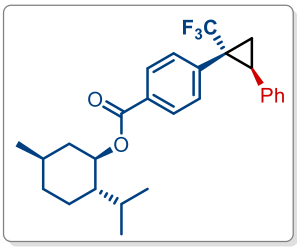

According to **Method D**:

N-triftosylhydrazone derived from DL-menthol (219.7 mg, 0.38 mmol), styrene oxide (18.0 mg, 0.15 mmol), NaH (15.2 mg, 0.38 mmol, 60 wt.% dispersion in mineral oil) and $Tp^{Br3}Ag(thf)$ (8.2 mg, 5 mol%) were reacted in dichloromethane (4.0 mL) for 2 h to afford compound **76** as a white solid (mp: 88-89 °C).

Run: 44.0 mg, 66% yield.

Purification: Silica gel column chromatography (5% EtOAc in petroleum ether).

^{1}H NMR (500 MHz, $CDCl_3$) δ 7.83 (d, *J* = 8.0 Hz, 2H), 7.22 (d, *J* = 8.0 Hz, 2H), 7.14-7.07 (m, 3H), 6.84-6.73 (m, 2H), 4.92-4.82 (m, 1H), 2.89 (dd, *J* = 9.0, 7.5 Hz, 1H), 2.08 (d, *J* = 12.0 Hz, 1H), 1.92 (dd, *J* = 9.5, 6.0 Hz, 2H), 1.75-1.67 (m, 3H), 1.56-1.46 (m, 2H), 1.16-0.99 (m, 2H), 0.95-0.87 (m, 7H), 0.76 (d, *J* = 7.0 Hz, 3H).

^{13}C NMR (150 MHz, $CDCl_3$) δ 165.7, 136.4, 135.0, 132.4, 130.7 (d, *J* = 3.0 Hz), 129.2, 128.1, 127.9 (d, *J* = 6.0 Hz), 127.4 (q, *J* = 273.0 Hz), 126.7, 75.0, 47.2, 40.9 (d, *J* = 4.5 Hz), 35.6 (q, *J* = 31.5 Hz), 34.3, 31.4, 26.4, 25.8, 23.5, 22.0, 20.8, 16.4, 14.5 (d, *J* = 10.5 Hz).

^{19}F NMR (564 MHz, $CDCl_3$) δ -69.53 (s).

IR (Film): 2956, 2929, 1715, 1274, 1152, 1139, 1115, 706, 697 cm^{-1}.

HRMS (ESI^+) m/z calcd for $C_{27}H_{32}F_3O_2$ $[M+H]^+$ 445.2349, found 445.2350.

4-(2-phenyl-1-(trifluoromethyl)cyclopropyl)phenyl 2-(1-(4-chlorobenzoyl)-5-methoxy-2-methyl-1*H*-indol-3-yl)acetate (77)

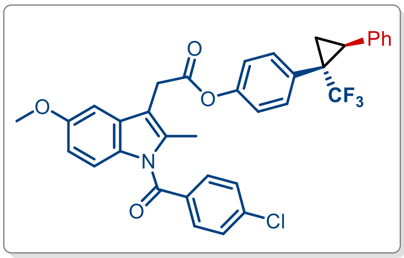

According to **Method D**:

N-triftosylhydrazone derived from indometacin (285.4 mg, 0.38 mmol), styrene oxide (18.0 mg, 0.15 mmol), NaH (15.2 mg, 0.38 mmol, 60 wt.% dispersion in mineral oil) and $Tp^{Br3}Ag(thf)$ (8.2 mg, 5 mol%) were reacted in dichloromethane (4.0 mL) for 2 h to afford compound **77** as a white solid (mp: 82-83 °C).

Run: 83.3 mg, 90% yield.

Purification: Silica gel column chromatography (10% EtOAc in petroleum ether).

^{1}H NMR (500 MHz, $CDCl_3$) δ 7.66 (d, *J* = 8.5 Hz, 2H), 7.46 (d, *J* = 8.5 Hz, 2H), 7.14-7.07 (m, 5H), 7.00 (d, *J* = 2.5 Hz, 1H), 6.88 (dd, *J* = 9.0, 5.5 Hz, 3H), 6.80-6.75 (dd, *J* = 7.2, 2.2 Hz, 2H), 6.68 (dd, *J* = 9.0, 2.5 Hz, 1H), 3.84 (s, 2H), 3.81 (s, 3H), 2.83 (dd, *J* = 9.5, 7.0 Hz, 1H), 2.42 (s, 3H), 1.87 (dd, *J* = 9.5, 6.0 Hz, 1H), 1.64 (t, *J* = 5.5 Hz, 1H).

^{13}C NMR (125 MHz, $CDCl_3$) δ 168.8, 168.3, 156.1, 150.5, 139.4, 136.2, 135.2, 131.2, 130.8, 130.4, 129.2, 129.1, 128.0, 127.9, 126.6, 125.7 (q, *J* = 272.5 Hz), 120.9, 115.0, 111.9, 111.8, 101.2, 55.7, 35.1 (q, *J* = 31.3 Hz), 30.5, 25.7, 14.7, 13.4.

^{19}F NMR (470 MHz, $CDCl_3$) δ -69.85 (s).

IR (Film): 2931, 2835, 1758, 1684, 1478, 1356, 1320, 1165, 1131, 1087, 835, 754, 698 cm^{-1}.

HRMS (ESI^+) m/z calcd for $C_{35}H_{26}ClF_3NO_4$ $[M-H]^-$ 616.1508, found 616.1494.

4-(2-phenyl-1-(trifluoromethyl)cyclopropyl)phenyl 2-(4-isobutylphenyl)propanoate (78)

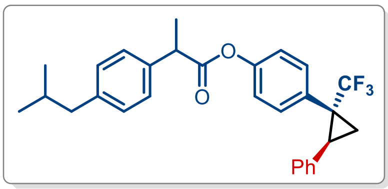

According to **Method D**:

N-triftosylhydrazone derived from ibuprofen (225.1 mg, 0.38 mmol), 2-(*p*-tolyl)oxirane (20.1 mg, 0.15 mmol), NaH (15.2 mg, 0.38 mmol, 60 wt.% dispersion in mineral oil) and $Tp^{Br3}Ag(thf)$ (8.2 mg, 5 mol%) were reacted in dichloromethane (4.0 mL) for 2 h to afford compound **78** as a colorless oil.

Run: 60.8 mg, 87% yield.

Purification: Silica gel column chromatography (4% EtOAc in petroleum ether).

^{1}H NMR (500 MHz, $CDCl_3$) δ 7.25 (d, *J* = 6.0 Hz, 2H), 7.14-7.06 (m, 7H), 6.81 (dd, *J* = 8.5, 1.0 Hz, 2H), 6.78-6.73 (m, 2H), 3.87 (q, *J* = 7.0 Hz, 1H), 2.82 (dd, *J* = 9.5, 7.0 Hz, 1H), 2.45 (d, *J* = 7.0 Hz, 2H), 1.88-1.81 (m, 2H), 1.63 (t, *J* = 6.0 Hz, 1H), 1.56 (dd, *J* = 7.0, 2.0 Hz, 3H), 0.90 (d, *J* = 6.5 Hz, 6H).

^{13}C NMR (150 MHz, $CDCl_3$) δ 172.8, 150.7, 140.8, 137.1 (d, *J* = 2.1 Hz), 135.3, 133.4, 129.5, 128.9, 127.9, 127.9, 127.2, 126.6, 125.7 (q, *J* = 273.0 Hz), 121.0, 45.2, 45.0, 35.2 (q, *J* = 33.0 Hz), 30.2, 25.7, 22.4, 18.4, 14.8.

^{19}F NMR (564 MHz, $CDCl_3$) δ -69.90 (s).

IR (Film): 2956, 2928, 2870, 1759, 1509, 1461, 1384, 1280, 1204, 1165, 1138, 1084, 752, 697 cm^{-1}.

HRMS (ESI^+) m/z calcd for $C_{29}H_{28}F_3O_2$ $[M-H]^-$ 465.2047, found 465.2002.

6 X-ray Crystallographic Data of Compound 46 and 69

Supplementary Table 1 Crystallography data and structure refinement for **46** (CCDC 2247410**)**

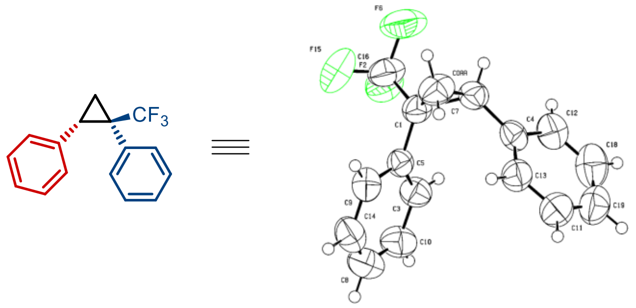

Empirical formula	$C_{15}F_{13}F_3$
Temperature	293(2) K
Formula weight	262.10
Unit cell dimensions	a = 14.915(4) Å alpha = 90 deg<br>b = 13.303(4) Å beta = 90 deg.<br>c = 6.8482(16) Å gamma = 90 deg.
Volume	1358.8(6) Å^3
Z	52
ρcalc	1.282 g/cm^3
μ/mm^{-1}	0.102
F(000)	544.0
Crystal size	0.1 x 0.1x 0.1 cm
Radiation	MoKα (λ = 0.71073 Å)
2θ range for data collection	7.228 to 59.34 /°
Reflections collected	6073
Independent reflections	2622[R_{int} = 0.0512, R_{sigma} = 0.0920]
Data/restraints/parameters	2922/0/172
Goodness-of-fit on F^2	1.044
Final R indexes [I>=2σ (I)]	R_1 = 0.0506, wR_2 = 0.0991
Final R indexes [all data]	R_1 = 0.1587, wR_2 = 0.1538

Compound 46 was crystallized as a colorless crystal via vaporization of a hexane/EtOAc solution, and its structure was determined by X-ray structure analysis. The crystallographic data that can be obtained free of charge from The Cambridge Crystallographic Data Centre via www.ccdc.cam.ac.uk/data request/cif.

Supplementary Table 2 Crystallography data and structure refinement for **69** **(**CCDC 2281663**)**

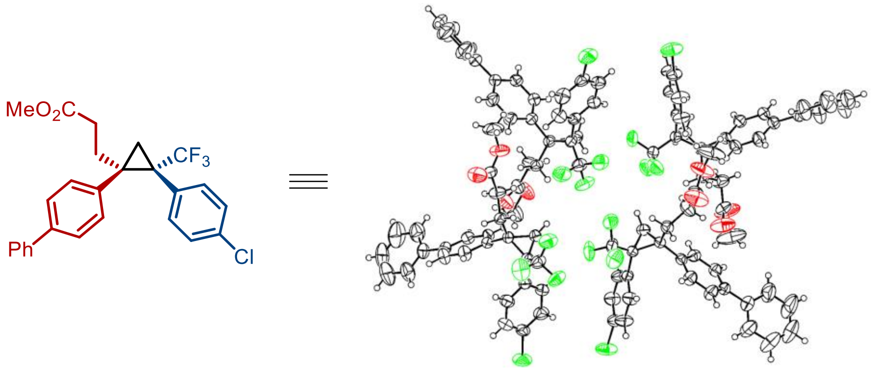

Empirical formula	$C_{26}H_{22}ClF_3O_2$
Temperature	300.03 K
Formula weight	458.88
Unit cell dimensions	a = 11.000(2) Å alpha = 90 deg<br>b = 11.303(2) Å beta = 90.122(10) deg.<br>c = 37.409(7) Å gamma = 90 deg.
Volume	4651.1(16) $Å^3$
Z	8
ρcalc	1.311 g/cm^3
μ/mm^{-1}	1.839
F(000)	1904.0
Crystal size	0.1 x 0.1x 0.1 cm
Radiation	MoKα (λ = 1.54178 Å)
2θ range for data collection	4.724 to 127.686 /°
Reflections collected	21467
Independent reflections	12084 [R_{int} = 0.0291, R_{sigma} = 0.0455]
Data/restraints/parameters	12084/2/1157
Goodness-of-fit on F^2	1.025
Final R indexes [I>=2σ (I)]	R_1 = 0.0442, wR_2 = 0.0995
Final R indexes [all data]	R_1 = 0.0637, wR_2 = 0.1092

Compound 69 was crystallized as a colorless crystal via vaporization of a hexane/EtOAc solution, and its structure was determined by X-ray structure analysis. The crystallographic data that can be obtained free of charge from The Cambridge Crystallographic Data Centre via www.ccdc.cam.ac.uk/data request/cif.

7 Copies of ¹H-, ¹³C-, ¹⁹F- and NOESY Spectra

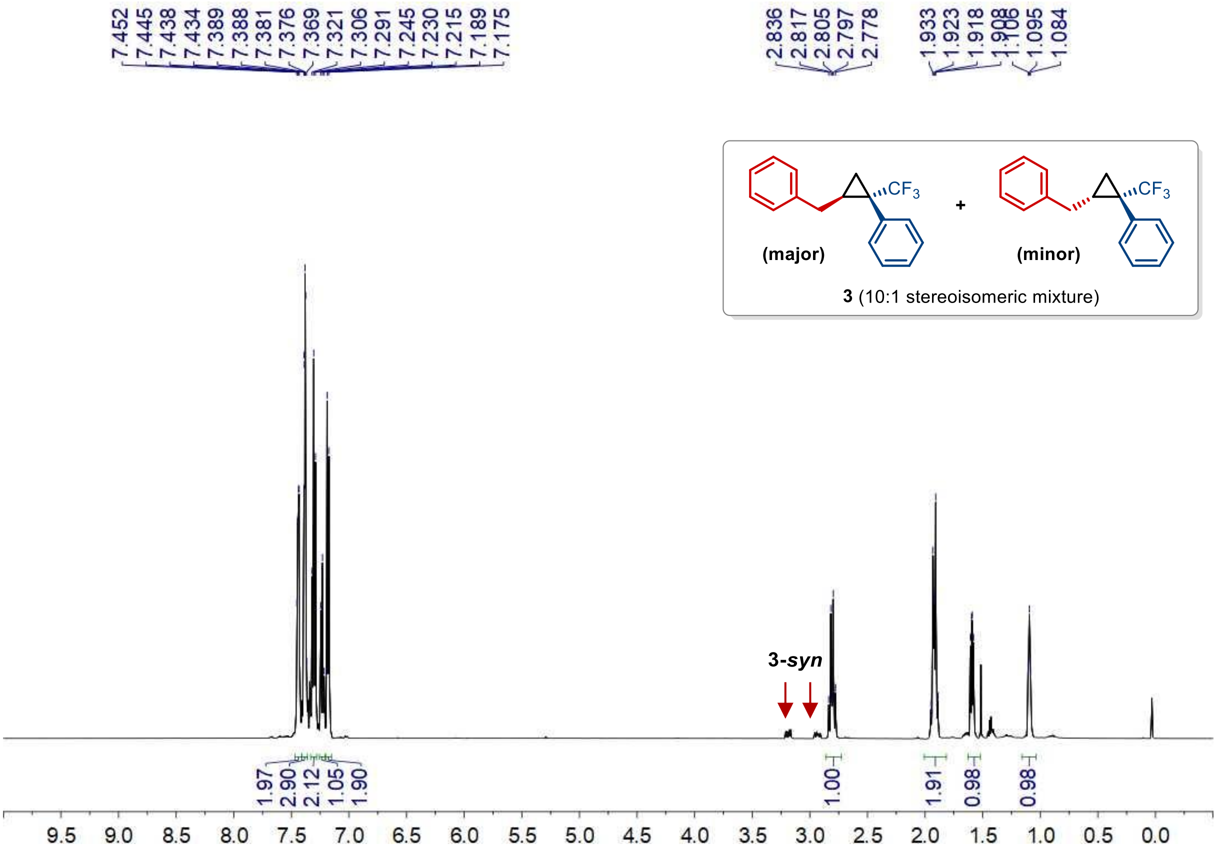

Supplementary Fig. 3 ¹H NMR (500 MHz, $CDCl_3$) spectrum of compound **3**.

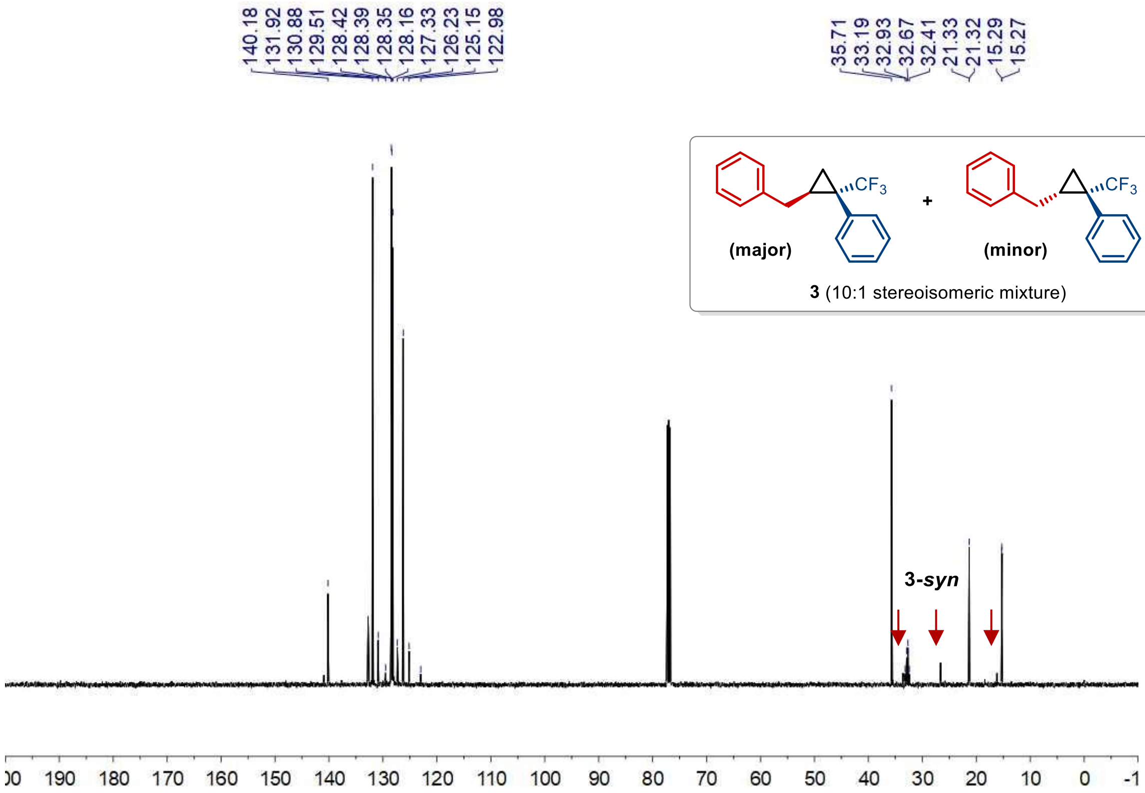

Supplementary Fig. 4 ¹³C NMR (125 MHz, $CDCl_3$) spectrum of compound **3**.

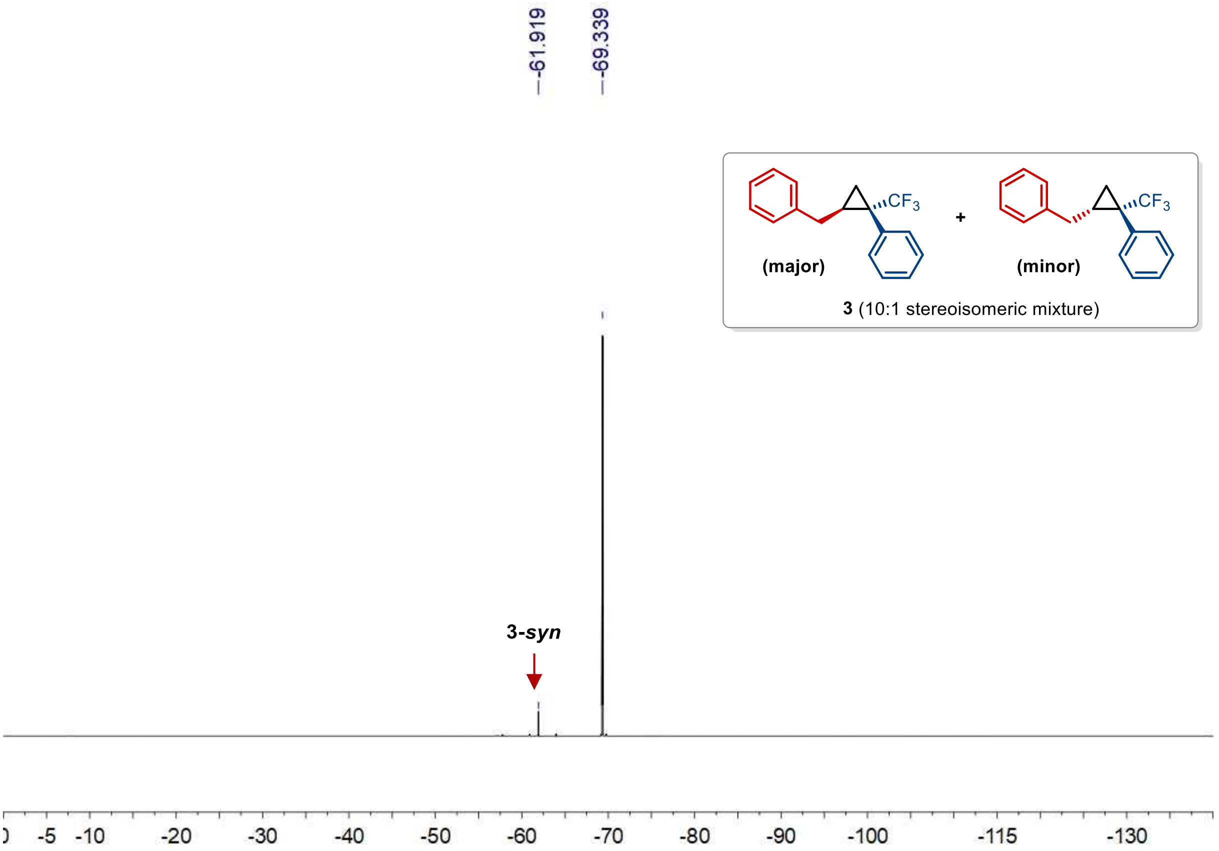

Supplementary Fig. 5 ^{19}**F NMR** (470 MHz, $CDCl_3$) spectrum of compound **3**.

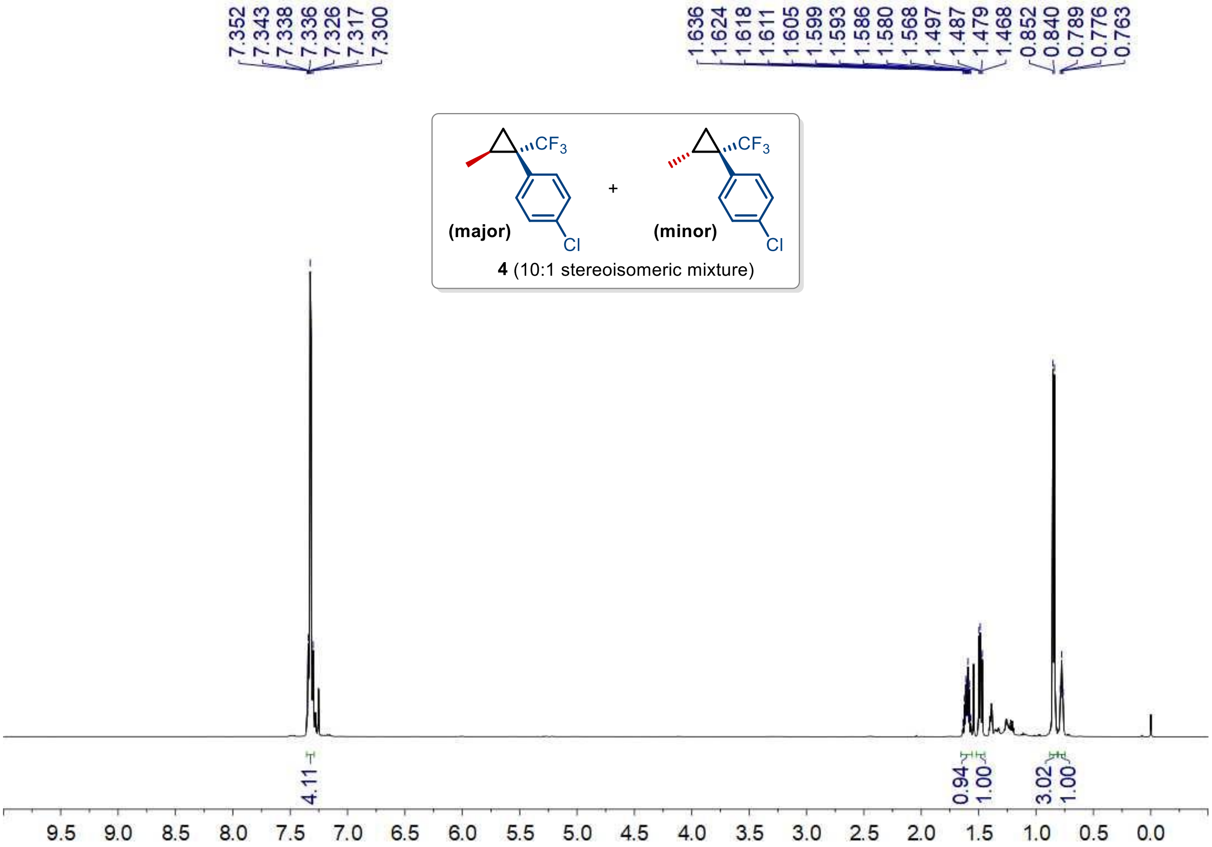

Supplementary Fig. 6 ^{1}**H NMR** (500 MHz, $CDCl_3$) spectrum of compound **4**.

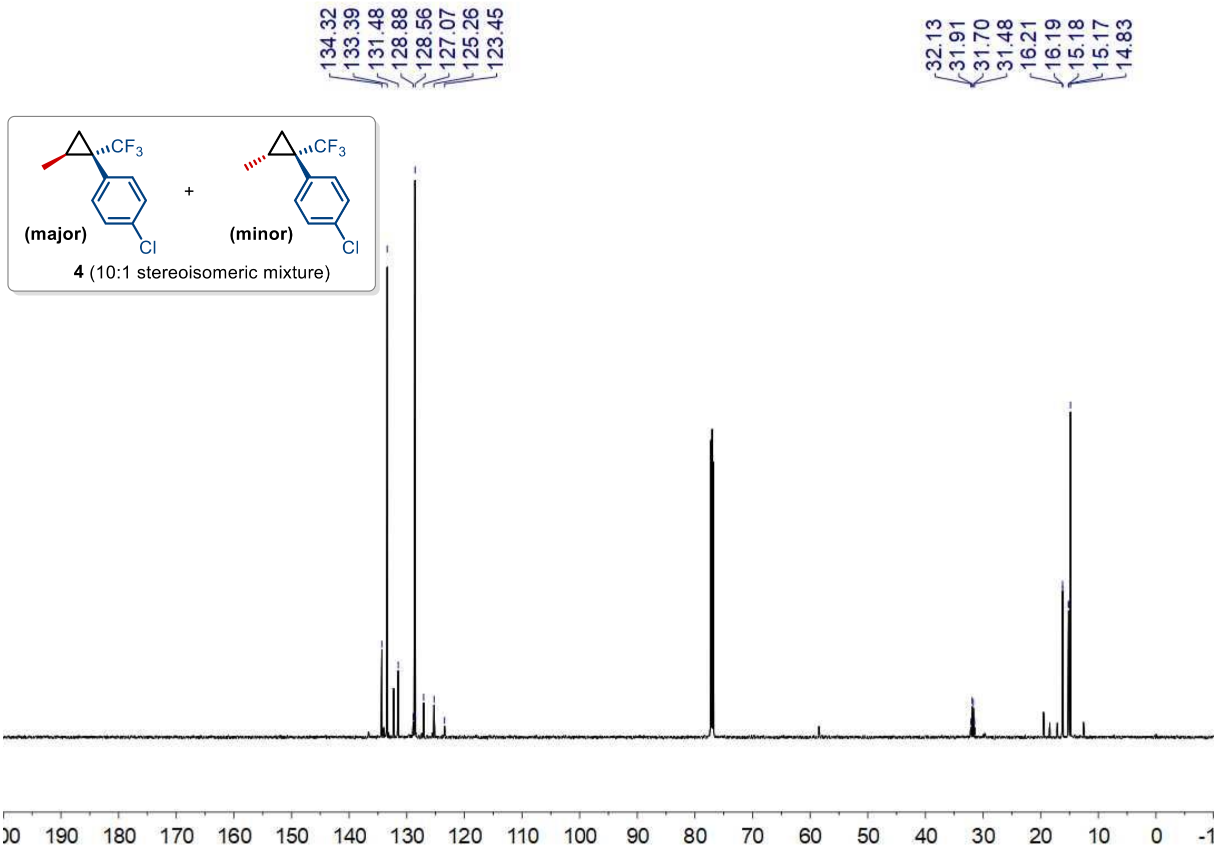

Supplementary Fig. 7 ^{13}**C NMR** (150 MHz, $CDCl_3$) spectrum of compound **4**.

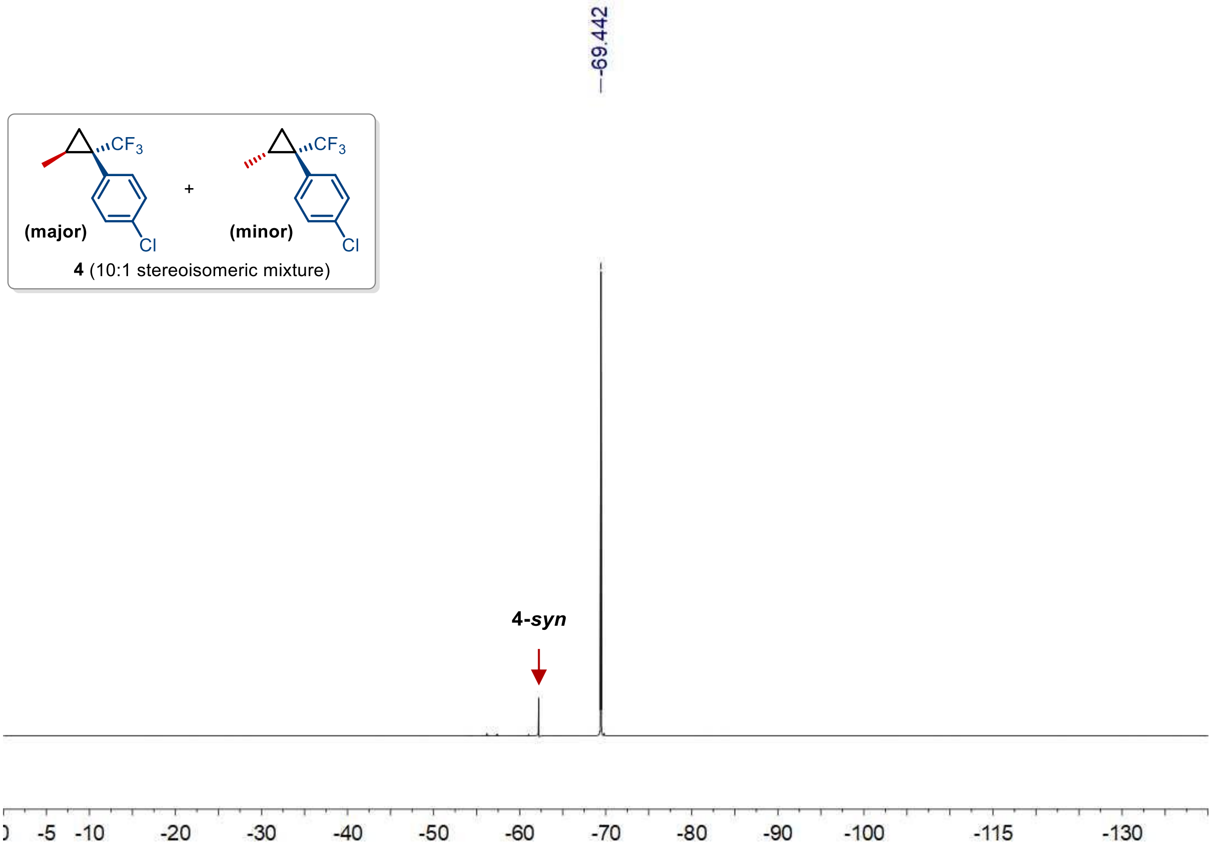

Supplementary Fig. 8 ^{19}**F NMR** (564 MHz, $CDCl_3$) spectrum of compound **4**.

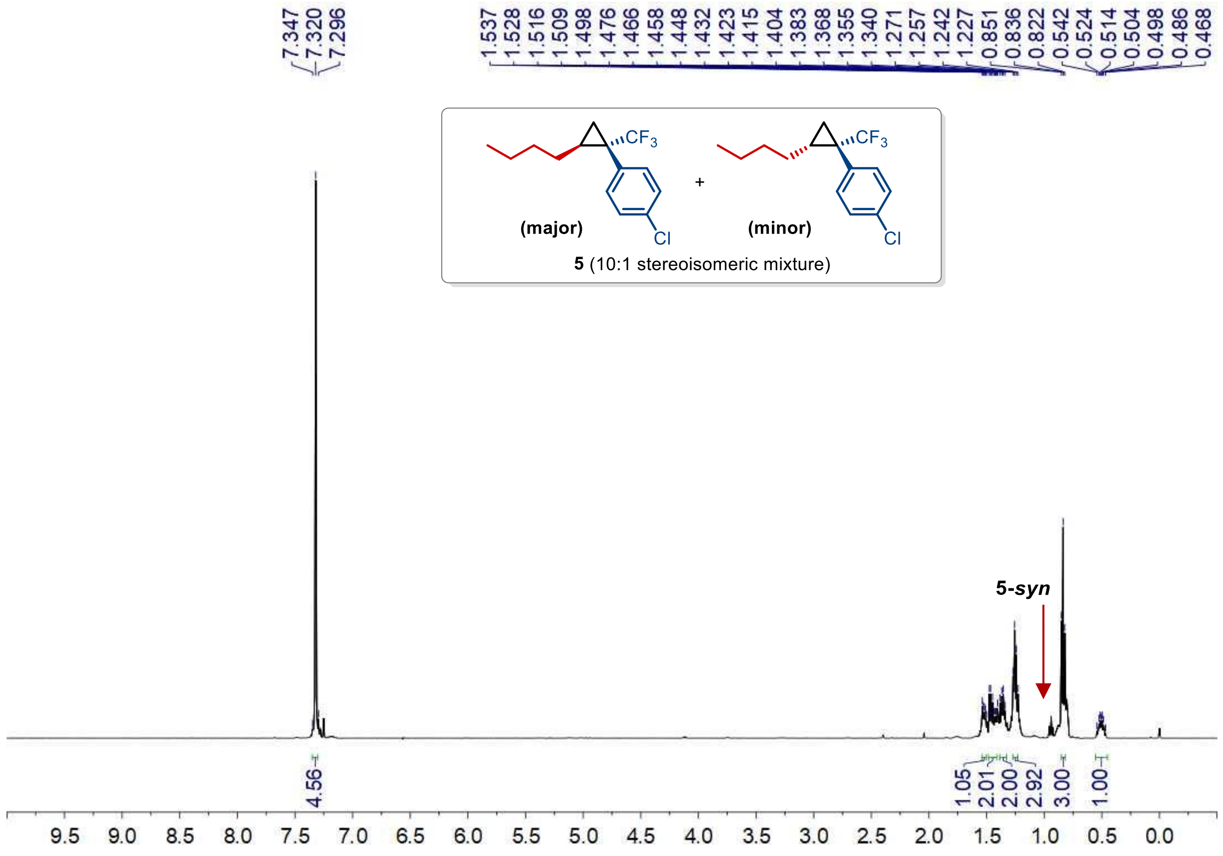

Supplementary Fig. 9 ^{1}H NMR (500 MHz, $CDCl_3$) spectrum of compound **5**.

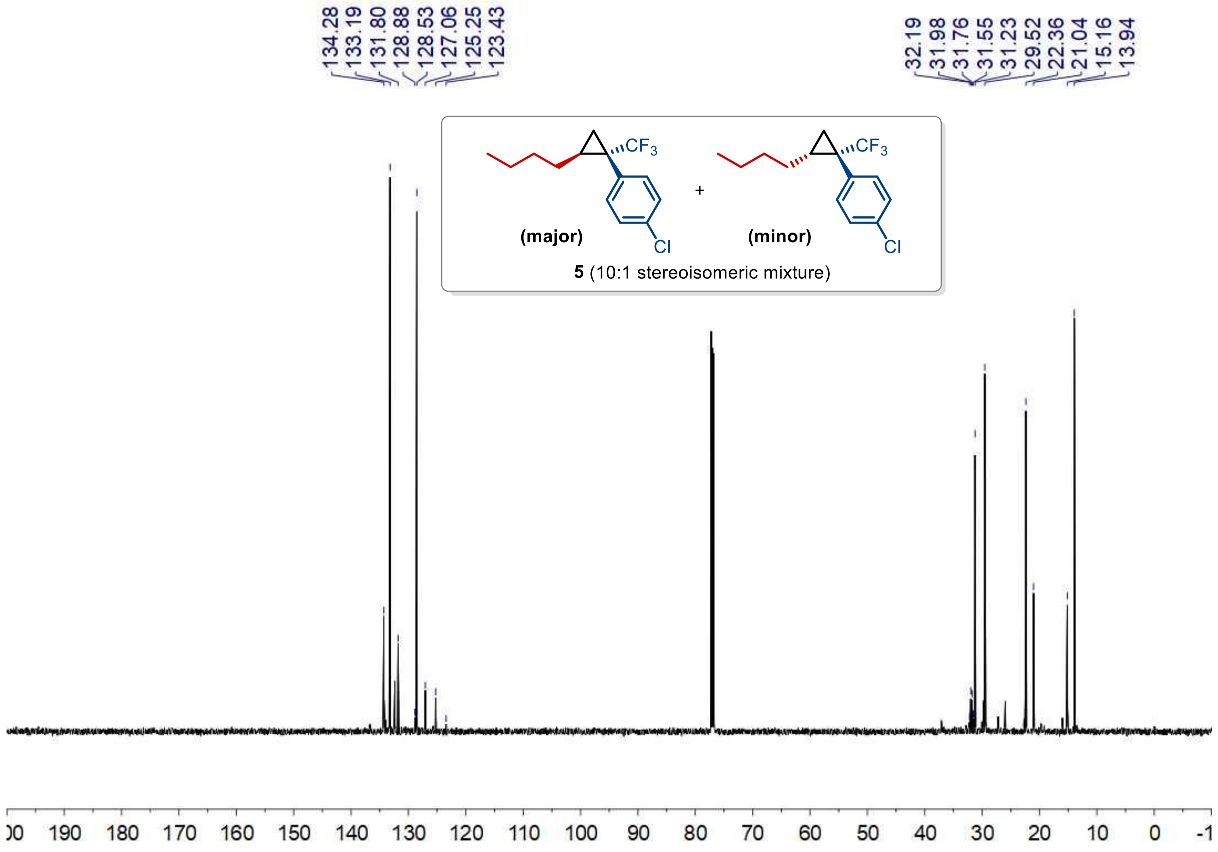

Supplementary Fig. 10 ^{13}C NMR (150 MHz, $CDCl_3$) spectrum of compound **5**.

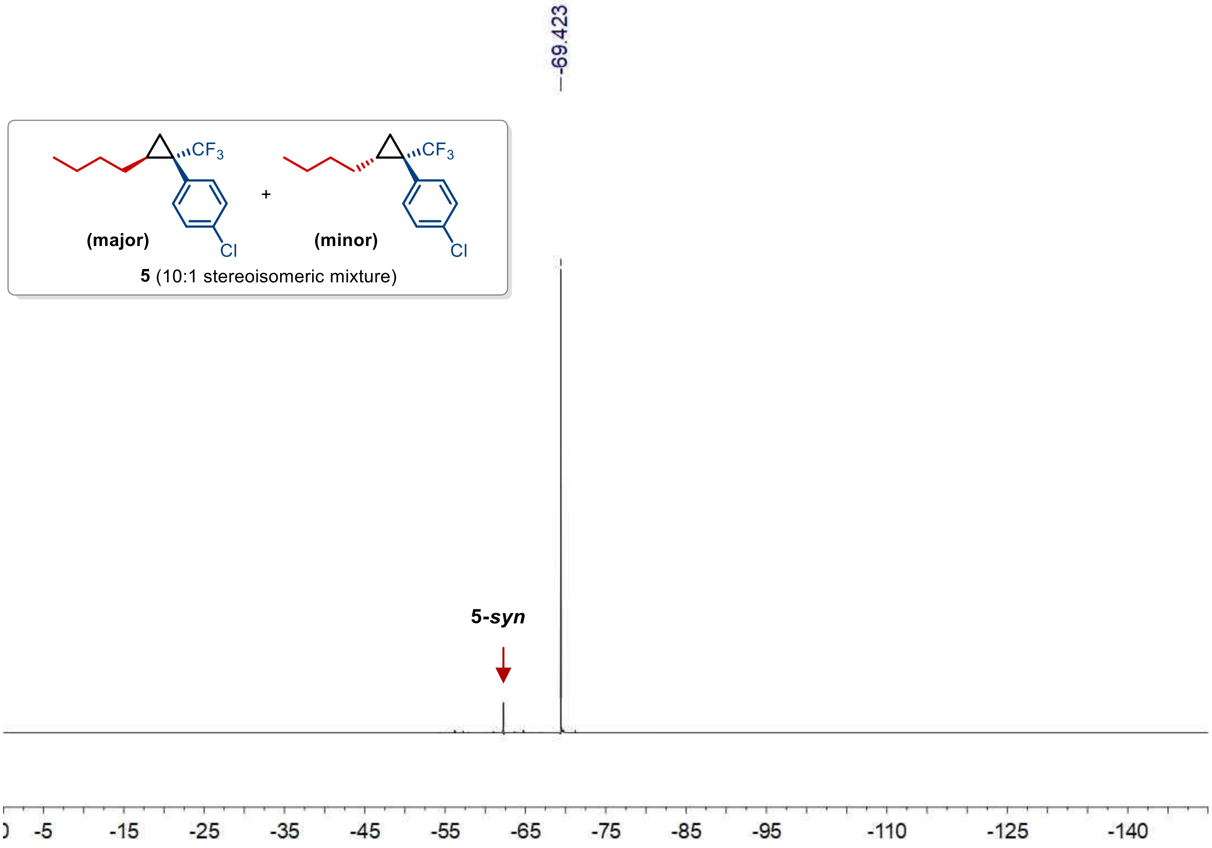

Supplementary Fig. 11 ^{19}F NMR (564 MHz, $CDCl_3$) spectrum of compound **5**.

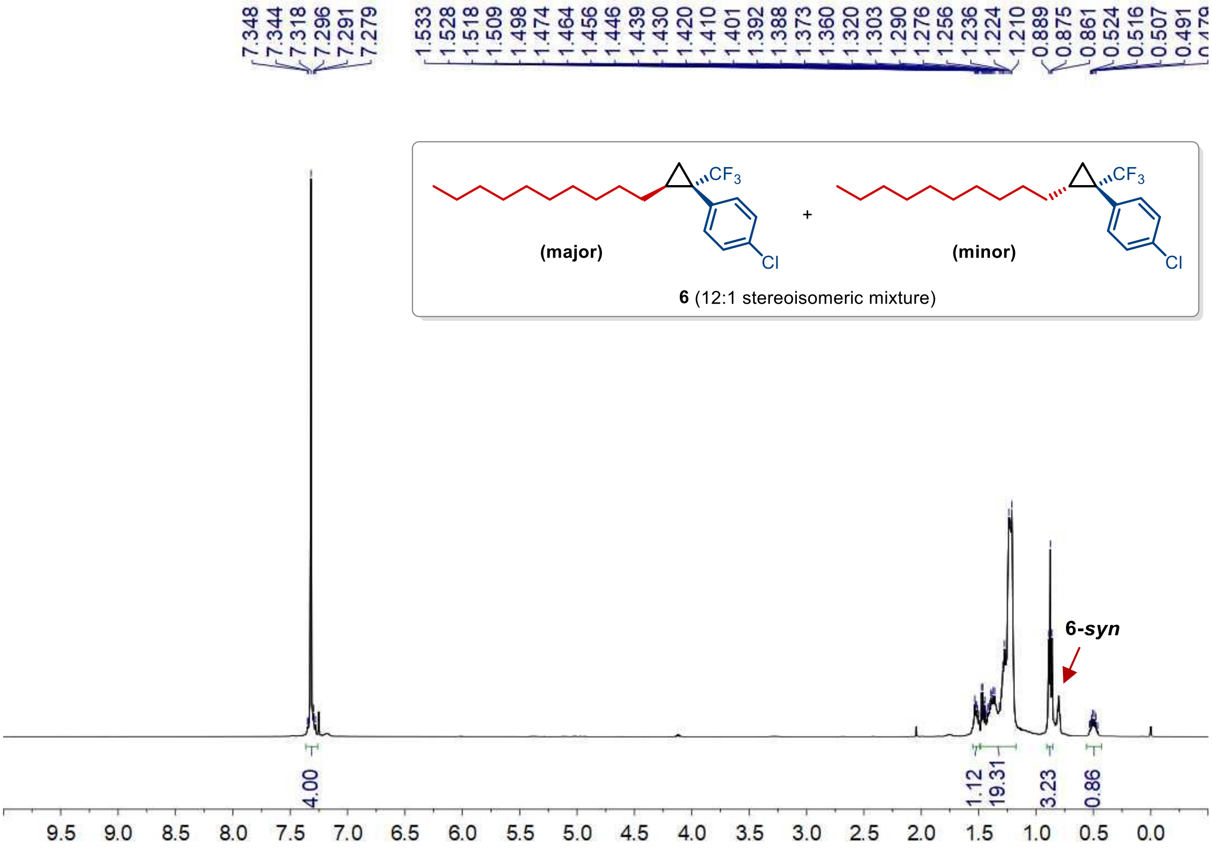

Supplementary Fig. 12 ^{1}H NMR (500 MHz, $CDCl_3$) spectrum of compound **6**.

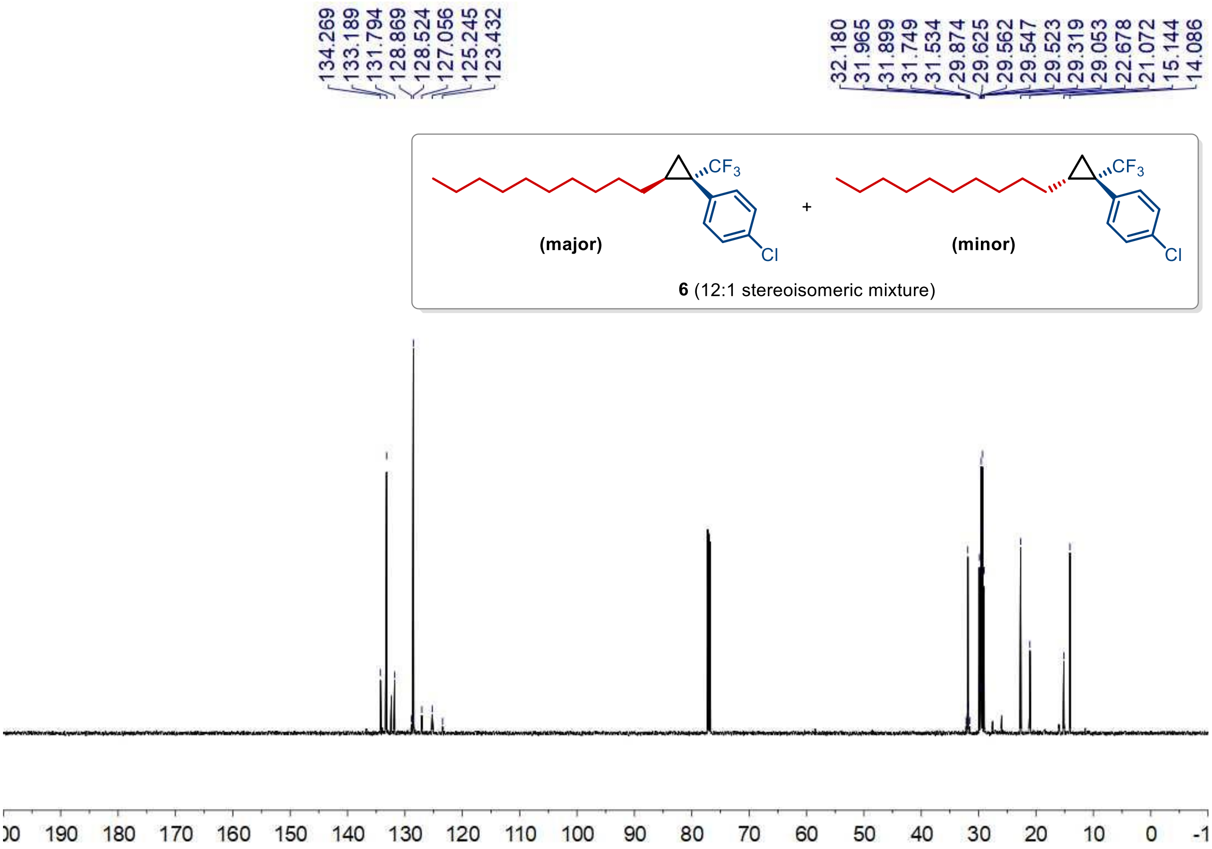

Supplementary Fig. 13 ^{13}**C NMR** (150 MHz, $CDCl_3$) spectrum of compound **6**.

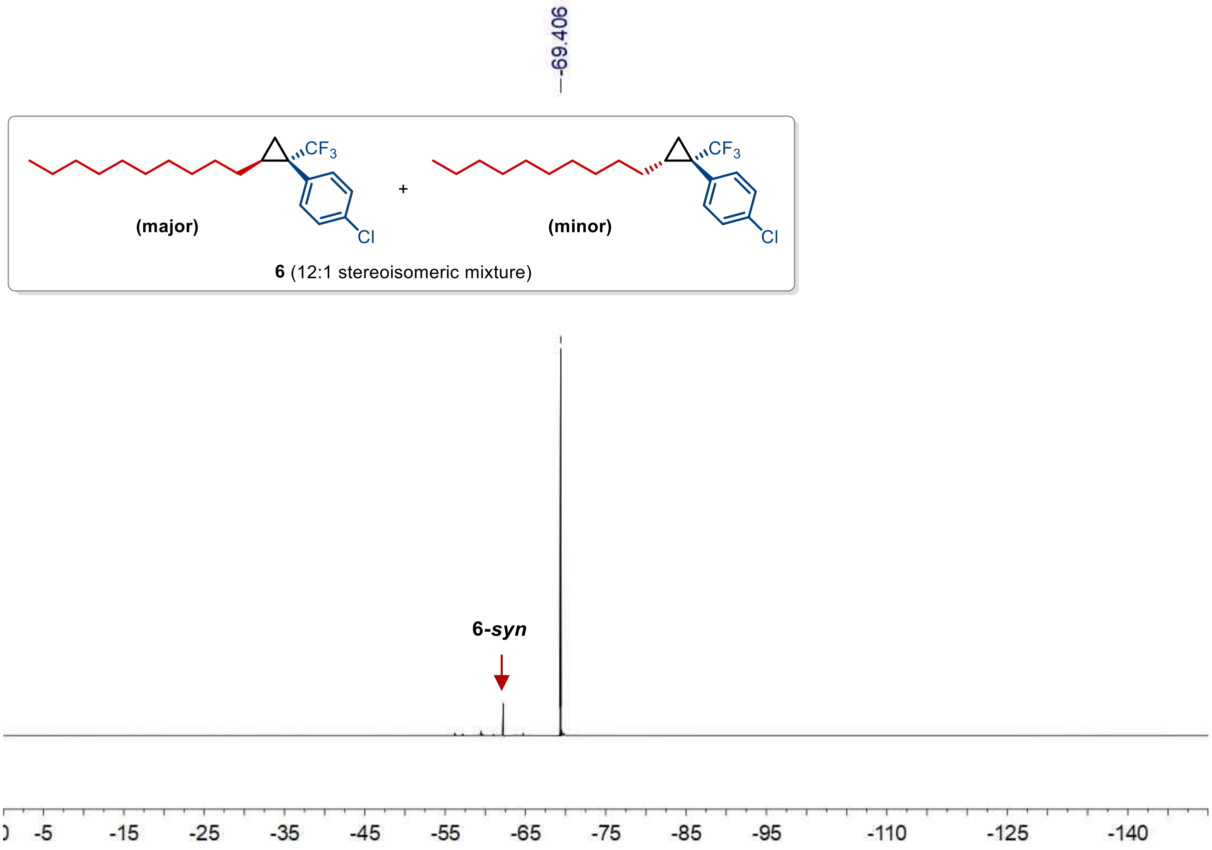

Supplementary Fig. 14 ^{19}**F NMR** (564 MHz, $CDCl_3$) spectrum of compound **6**.

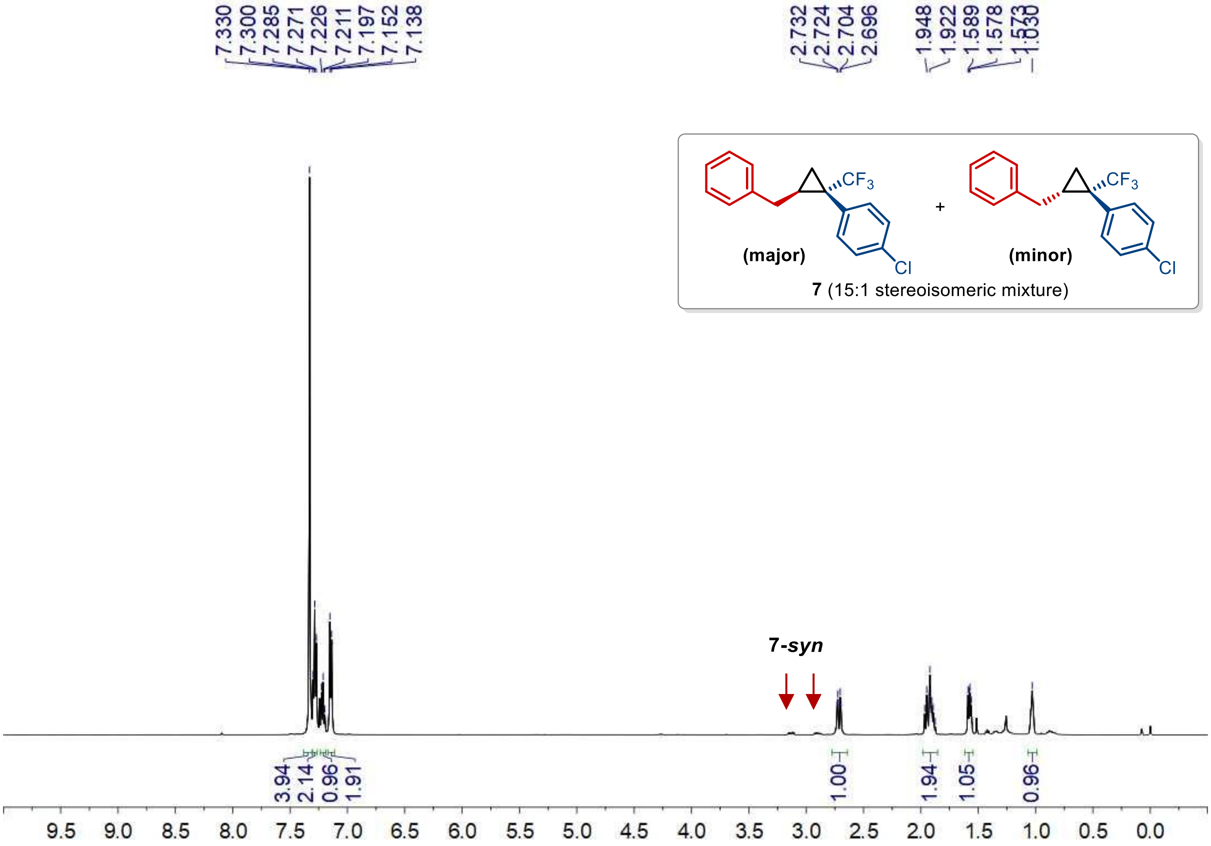

Supplementary Fig. 15 ^{1}H NMR (500 MHz, $CDCl_3$) spectrum of compound **7**.

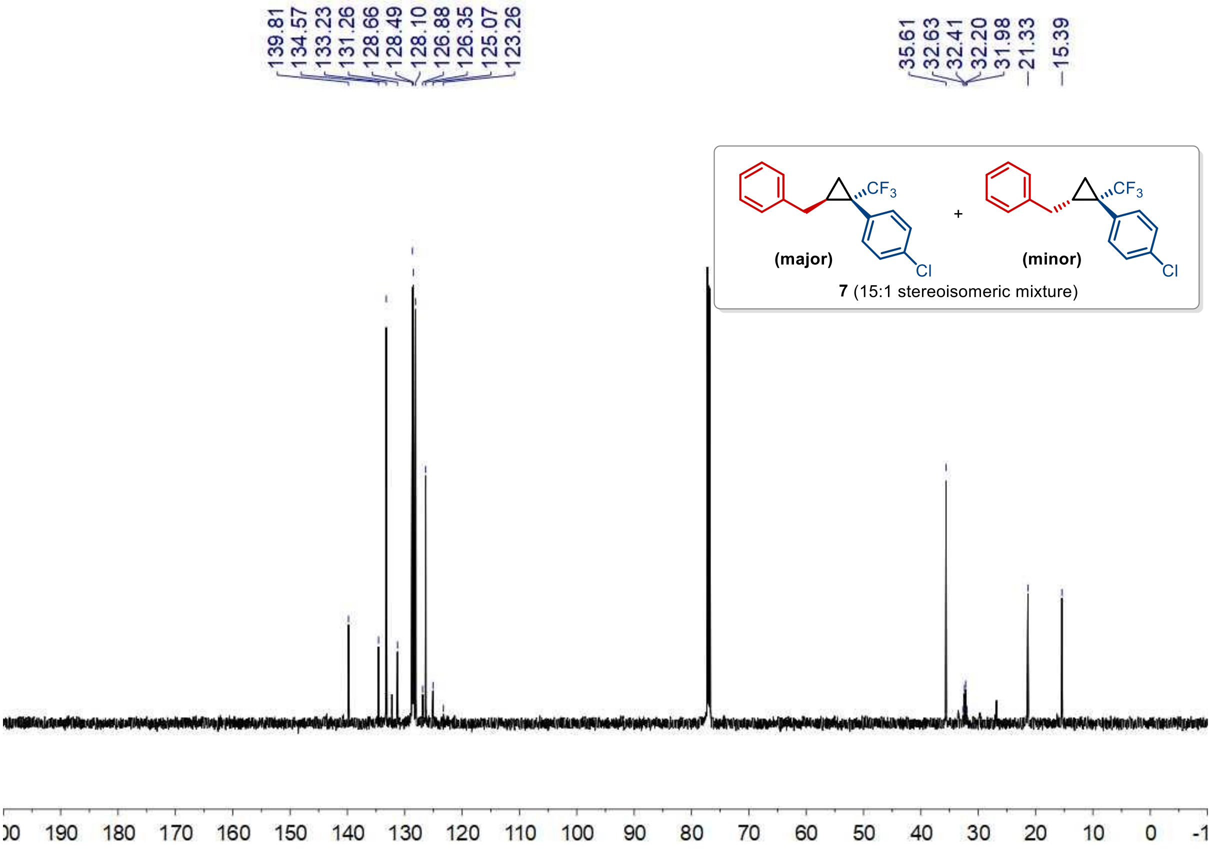

Supplementary Fig. 16 ^{13}C NMR (150 MHz, $CDCl_3$) spectrum of compound **7**.

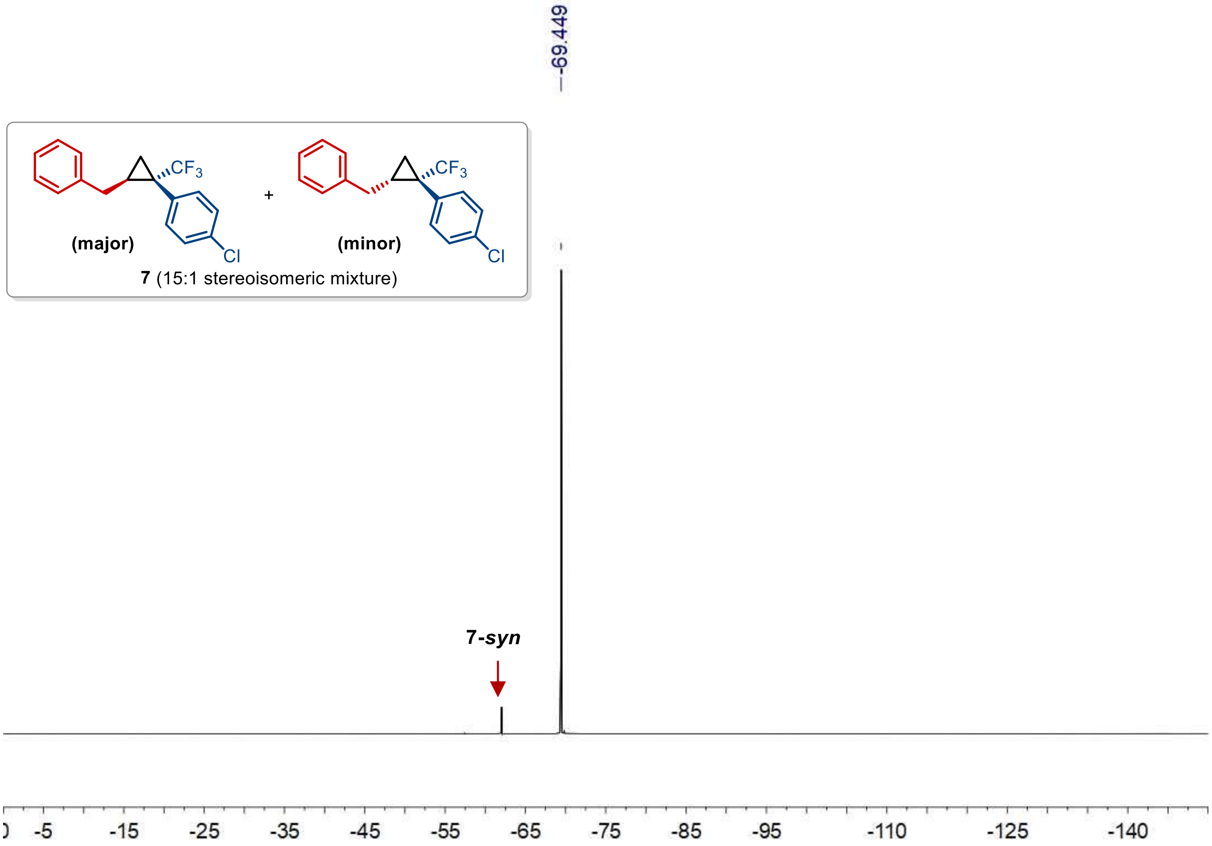

Supplementary Fig. 17 ^{19}**F NMR** (564 MHz, $CDCl_3$) spectrum of compound **7**.

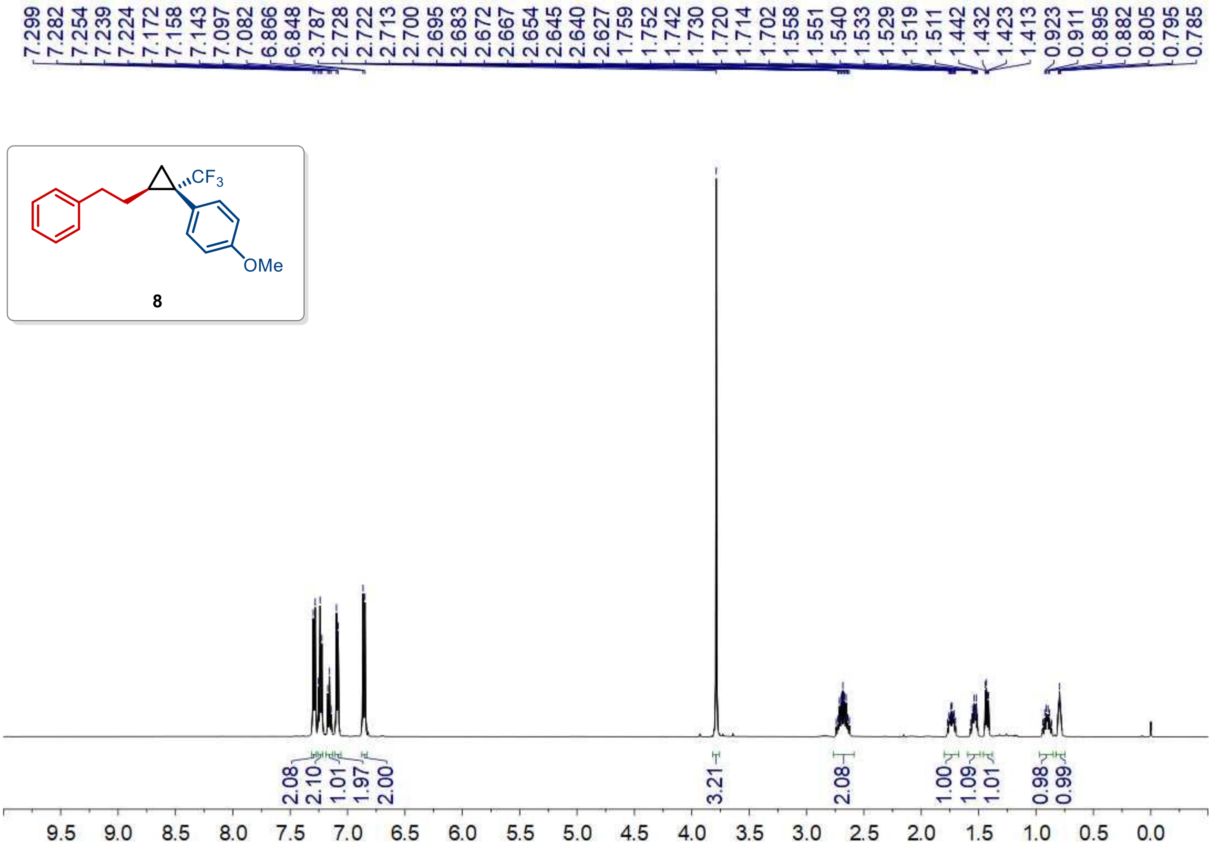

Supplementary Fig. 18 ^{1}**H NMR** (500 MHz, $CDCl_3$) spectrum of compound **8**.

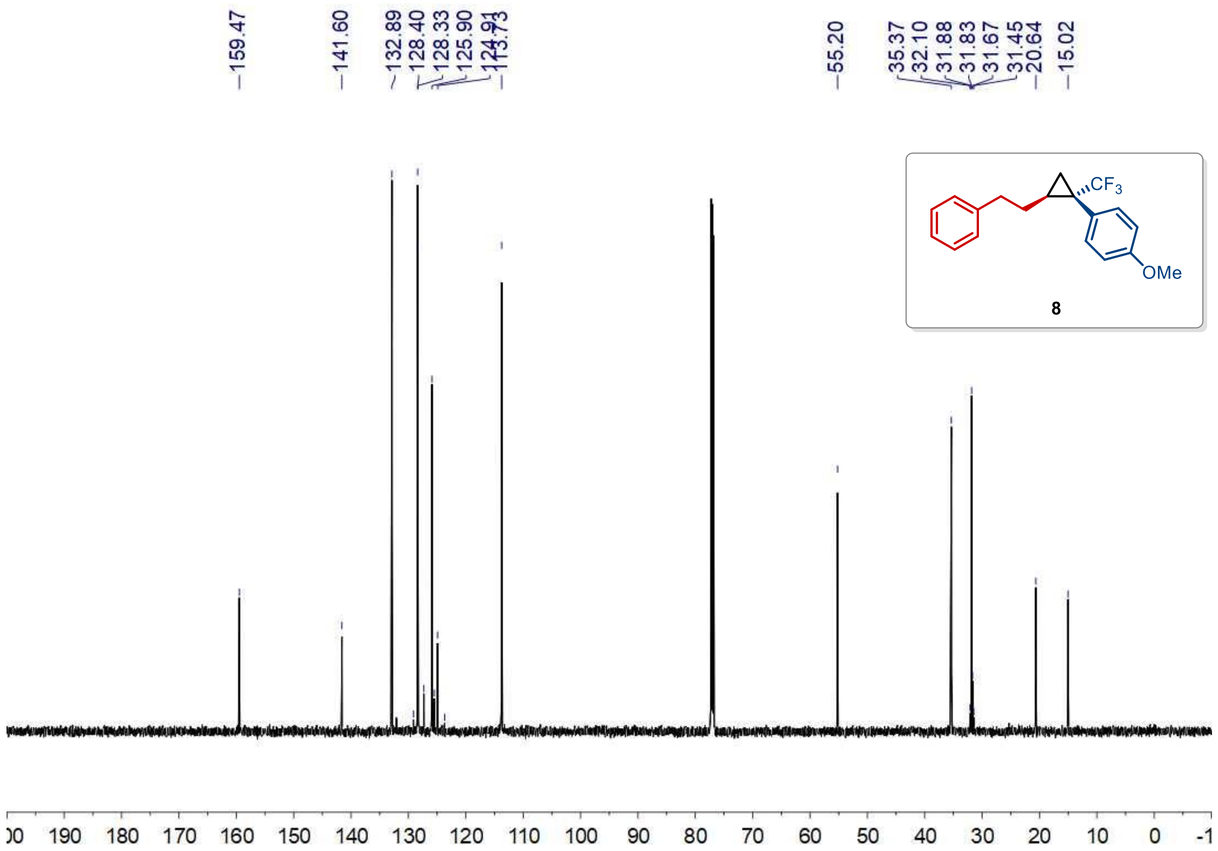

Supplementary Fig. 19 ^{13}**C NMR** (150 MHz, $CDCl_3$) spectrum of compound **8**.

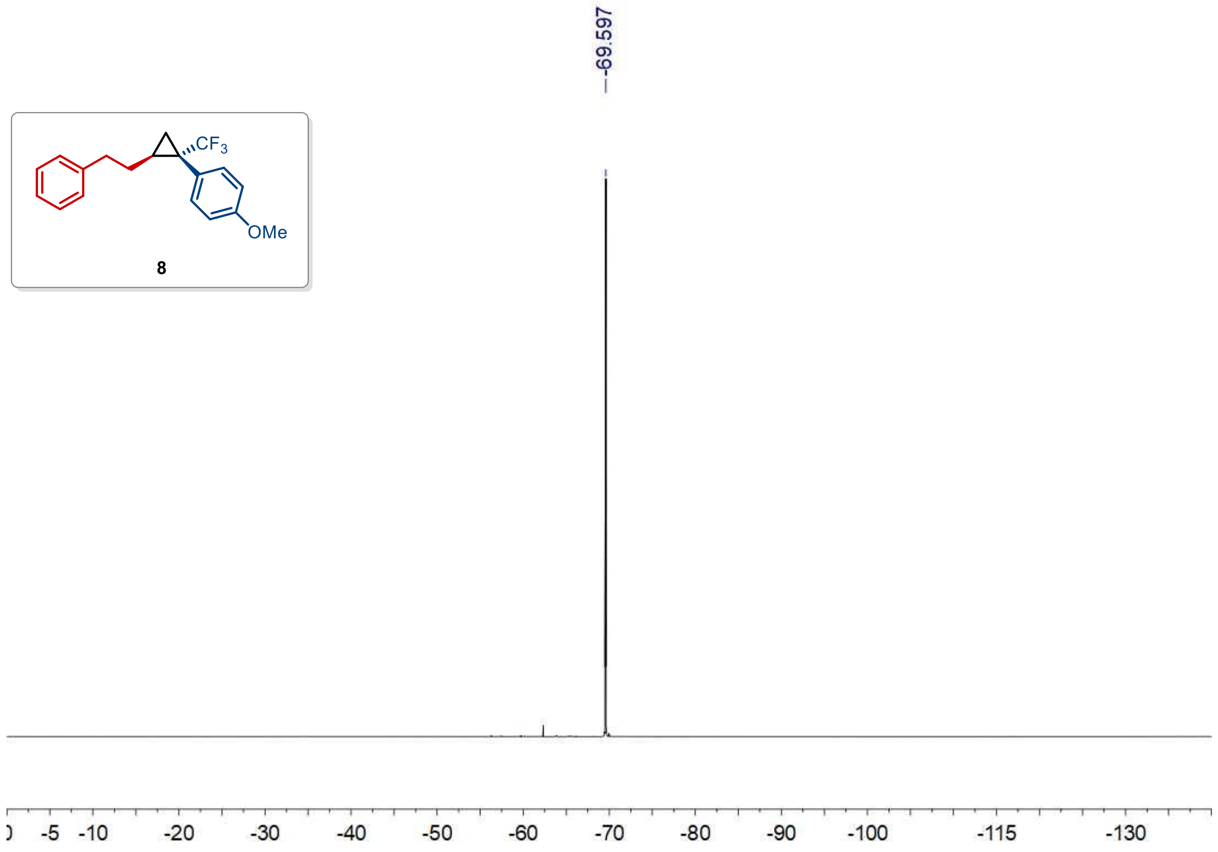

Supplementary Fig. 20 ^{19}**F NMR** (564 MHz, $CDCl_3$) spectrum of compound **8**.

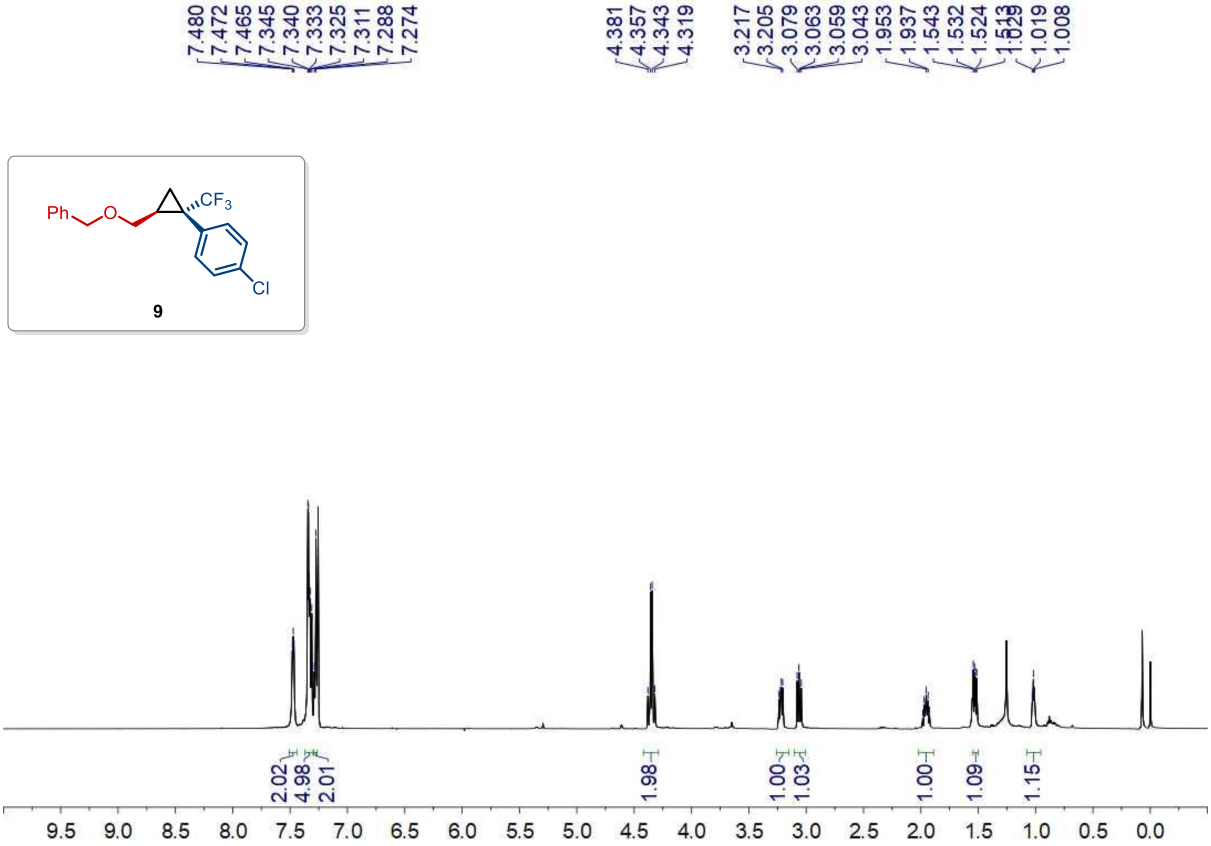

Supplementary Fig. 21 ^{1}**H NMR** (500 MHz, $CDCl_3$) spectrum of compound **9**.

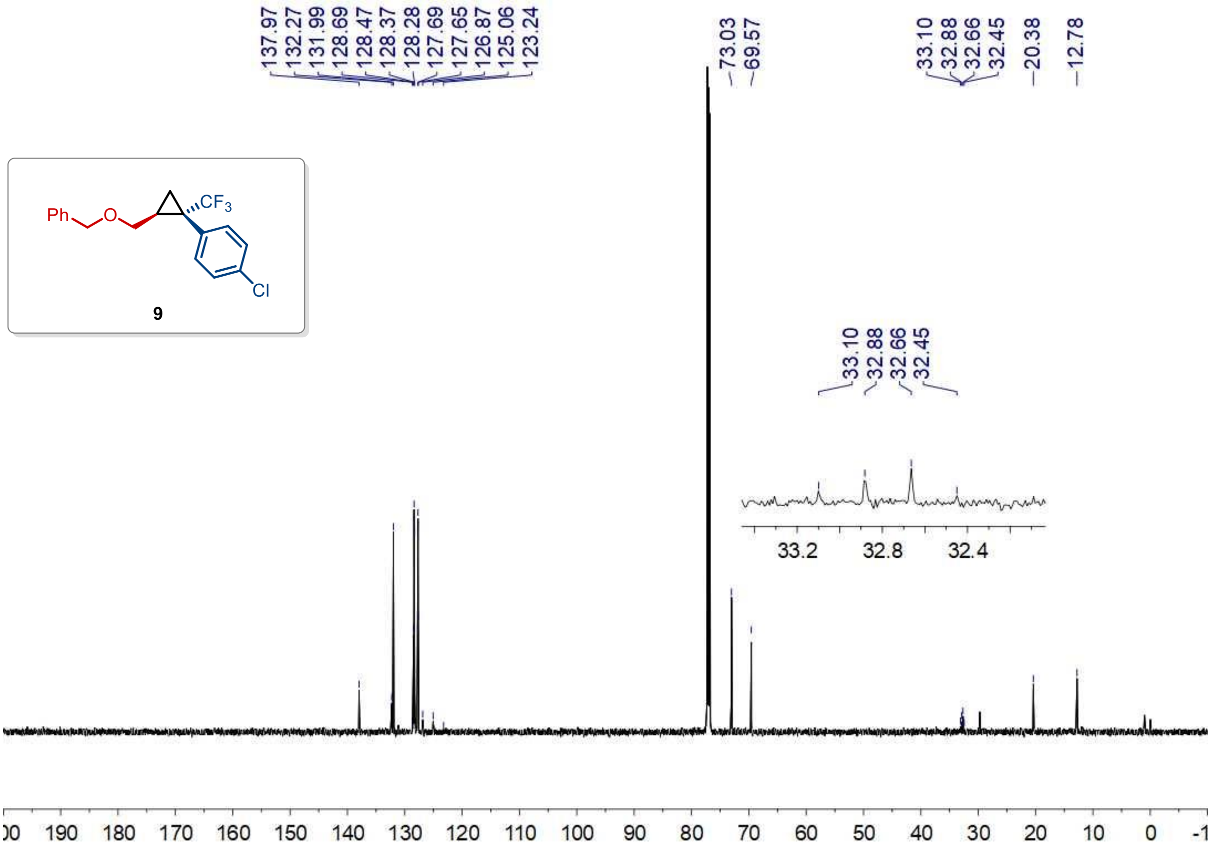

Supplementary Fig. 22 ^{13}**C NMR** (150 MHz, $CDCl_3$) spectrum of compound **9**.

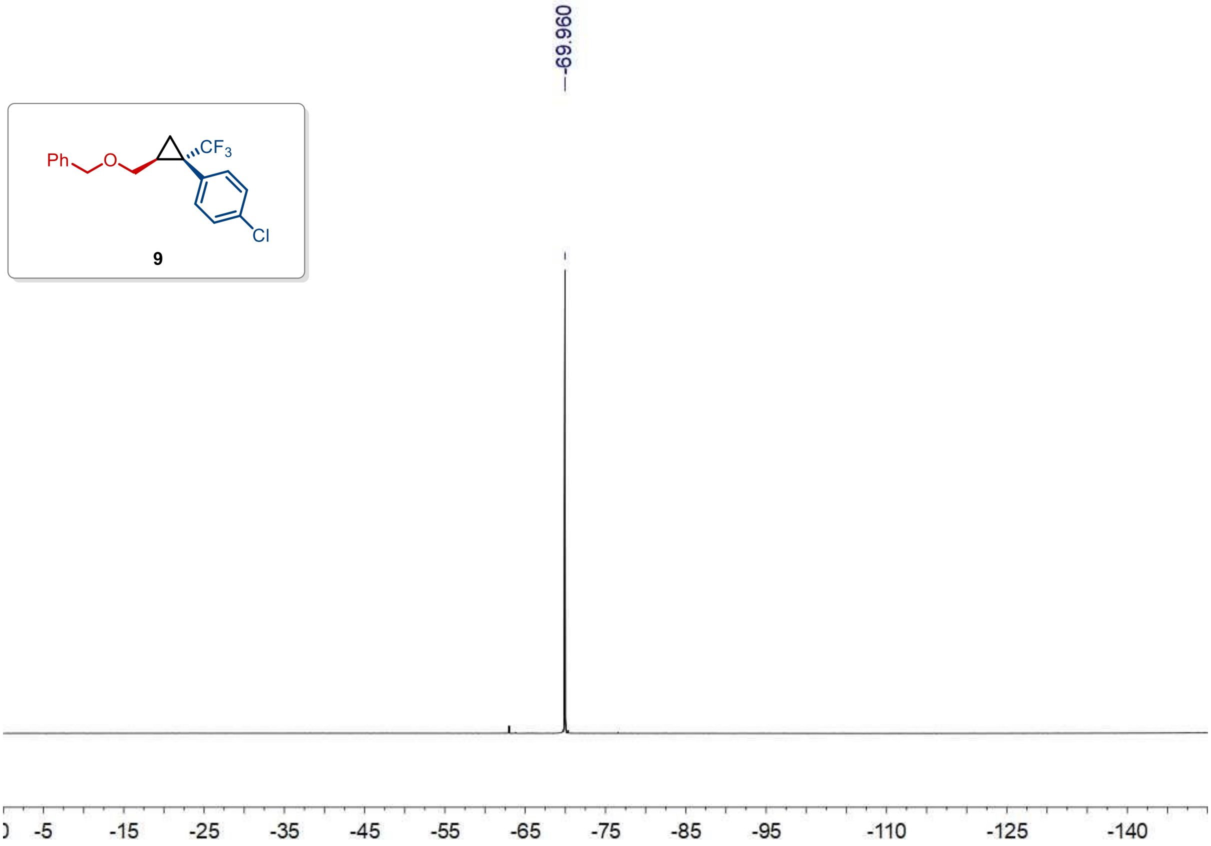

Supplementary Fig. 23 ^{19}F NMR (564 MHz, $CDCl_3$) spectrum of compound **9**.

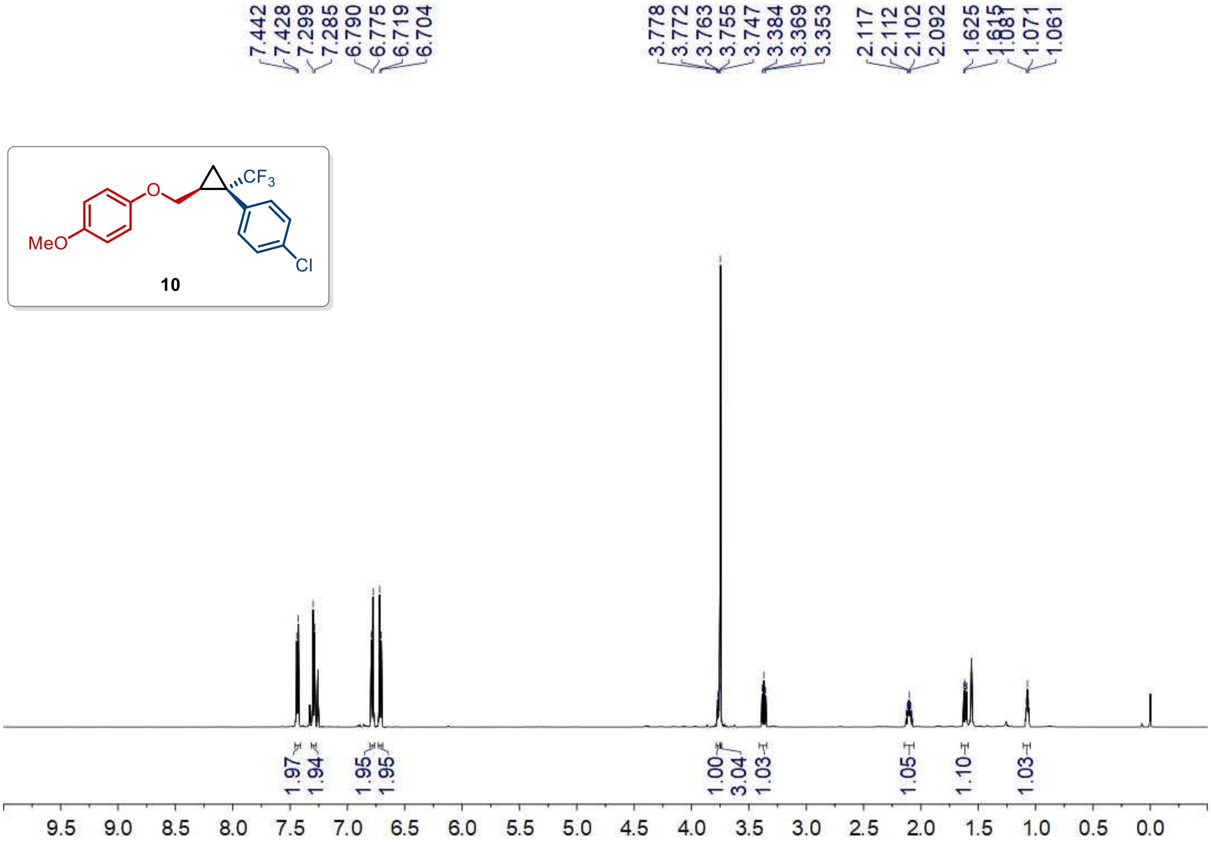

Supplementary Fig. 24 ^{1}H NMR (600 MHz, $CDCl_3$) spectrum of compound **10**.

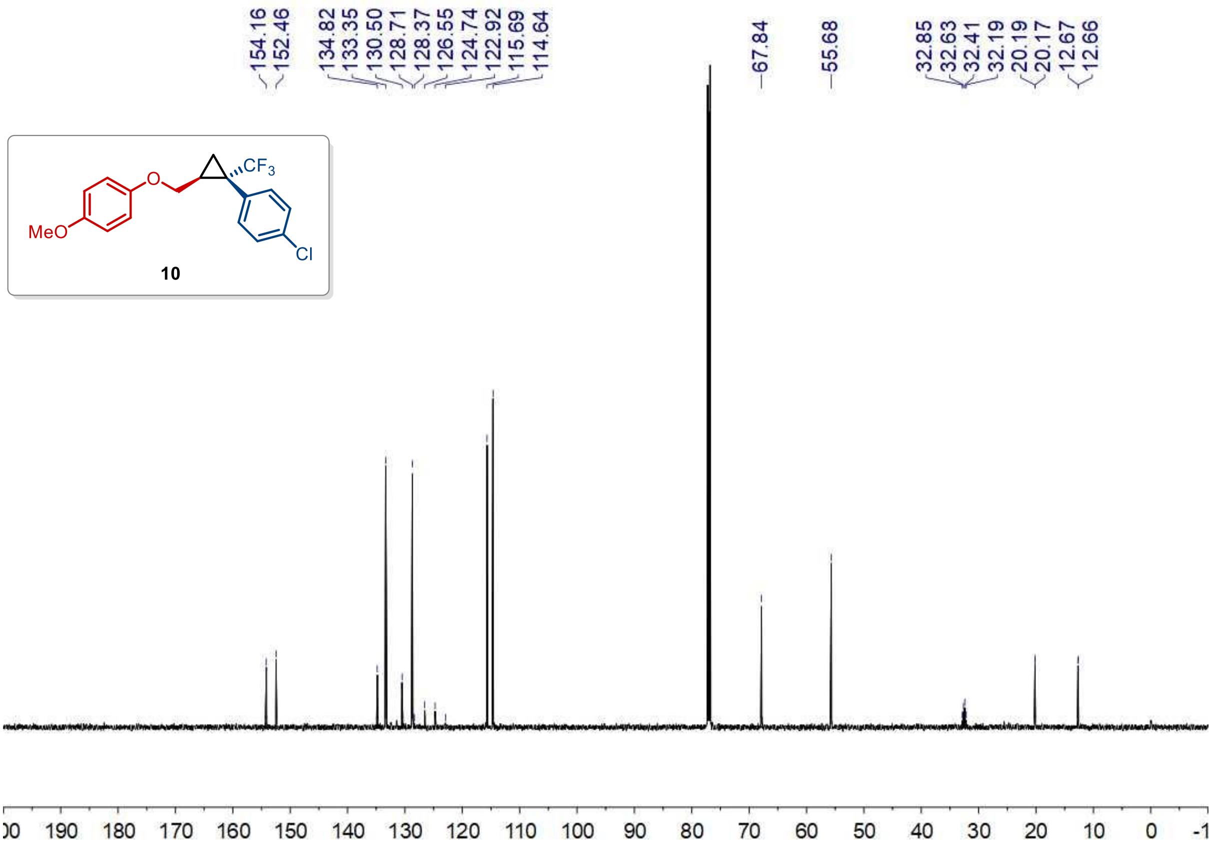

Supplementary Fig. 25 ^{13}C NMR (150 MHz, $CDCl_3$) spectrum of compound **10**.

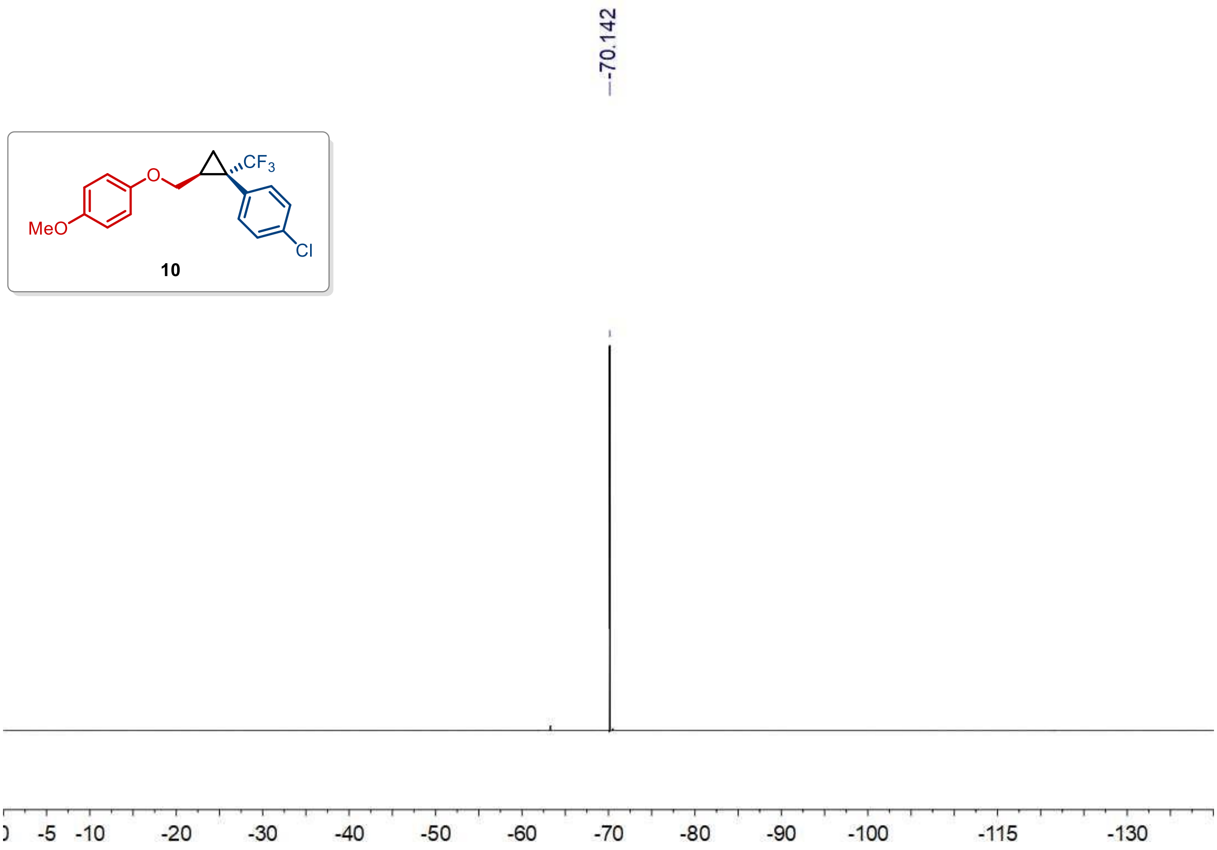

Supplementary Fig. 26 ^{19}F NMR (564 MHz, $CDCl_3$) spectrum of compound **10**.

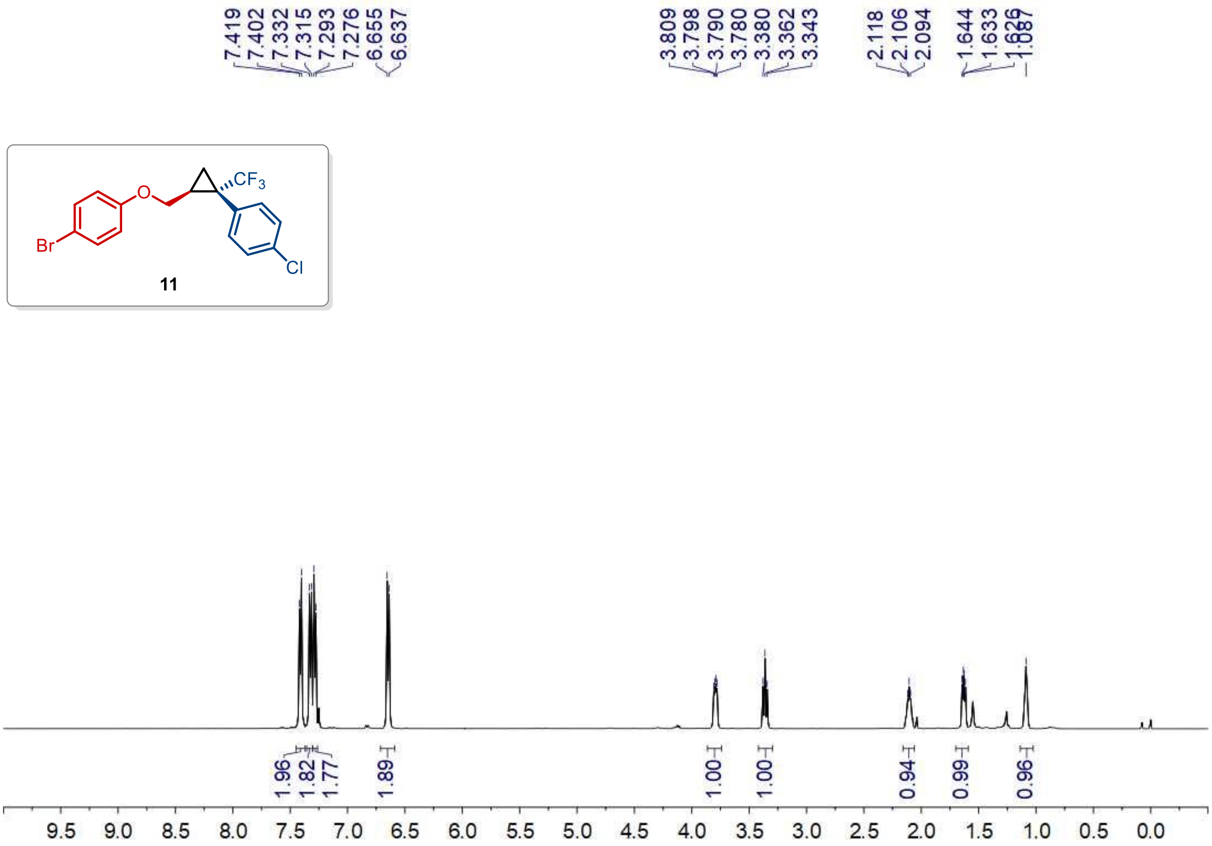

Supplementary Fig. 27 ^{1}H NMR (500 MHz, CDCl$_3$) spectrum of compound **11**.

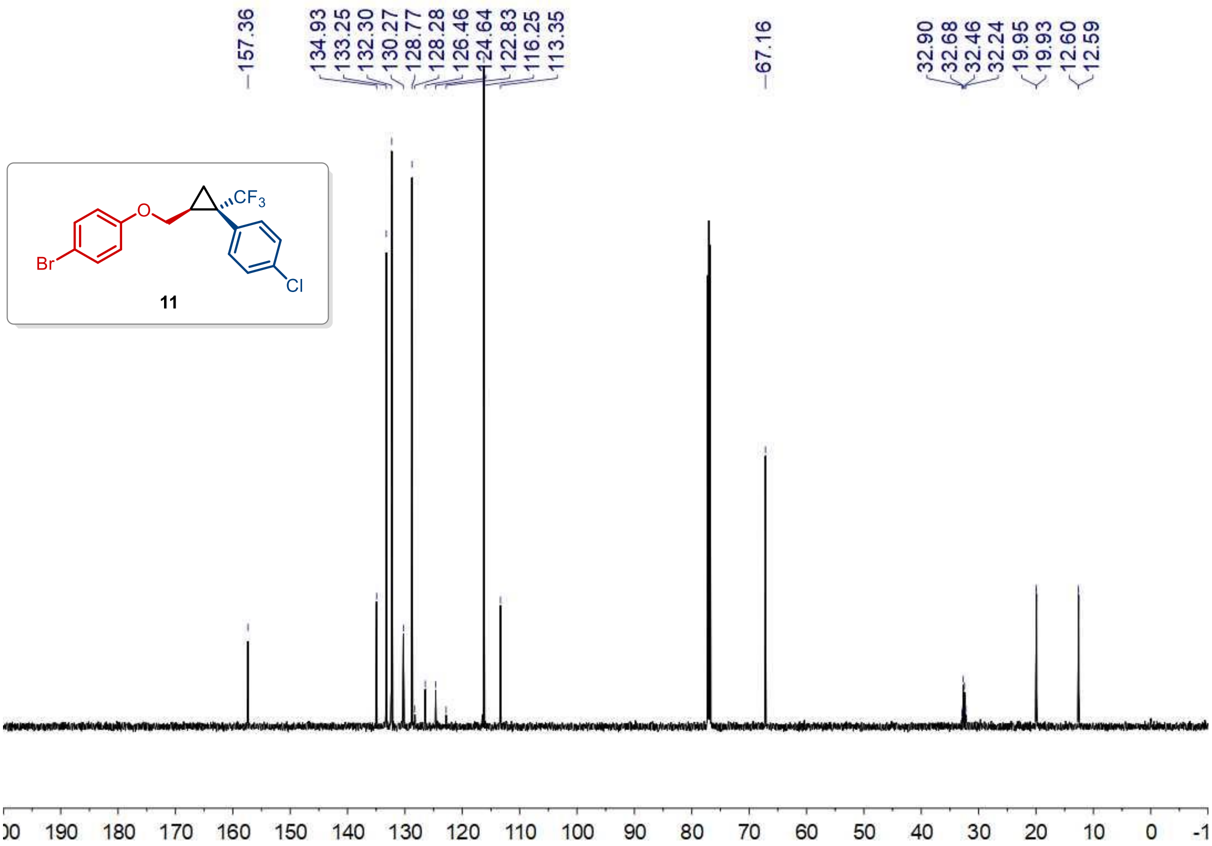

Supplementary Fig. 28 ^{13}C NMR (150 MHz, CDCl$_3$) spectrum of compound **11**.

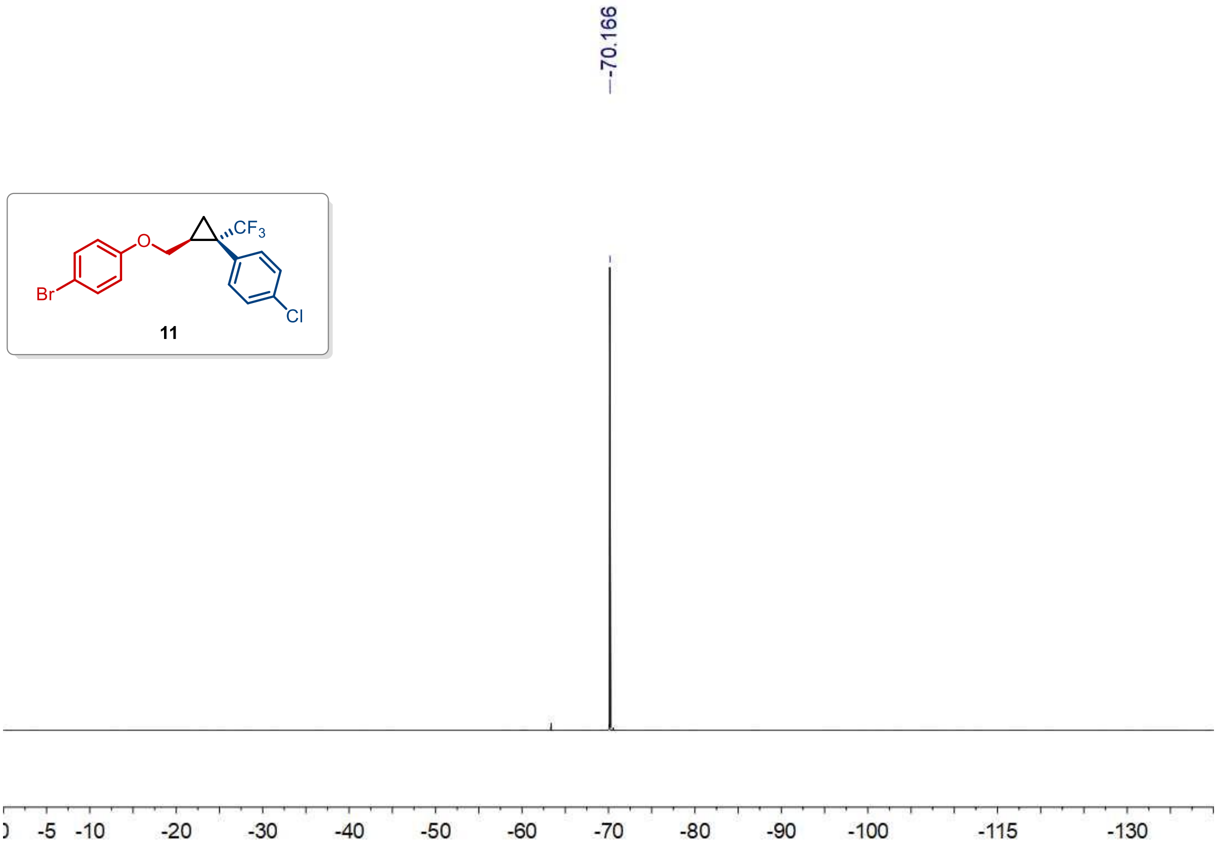

Supplementary Fig. 29 ^{19}F NMR (564 MHz, $CDCl_3$) spectrum of compound **11**.

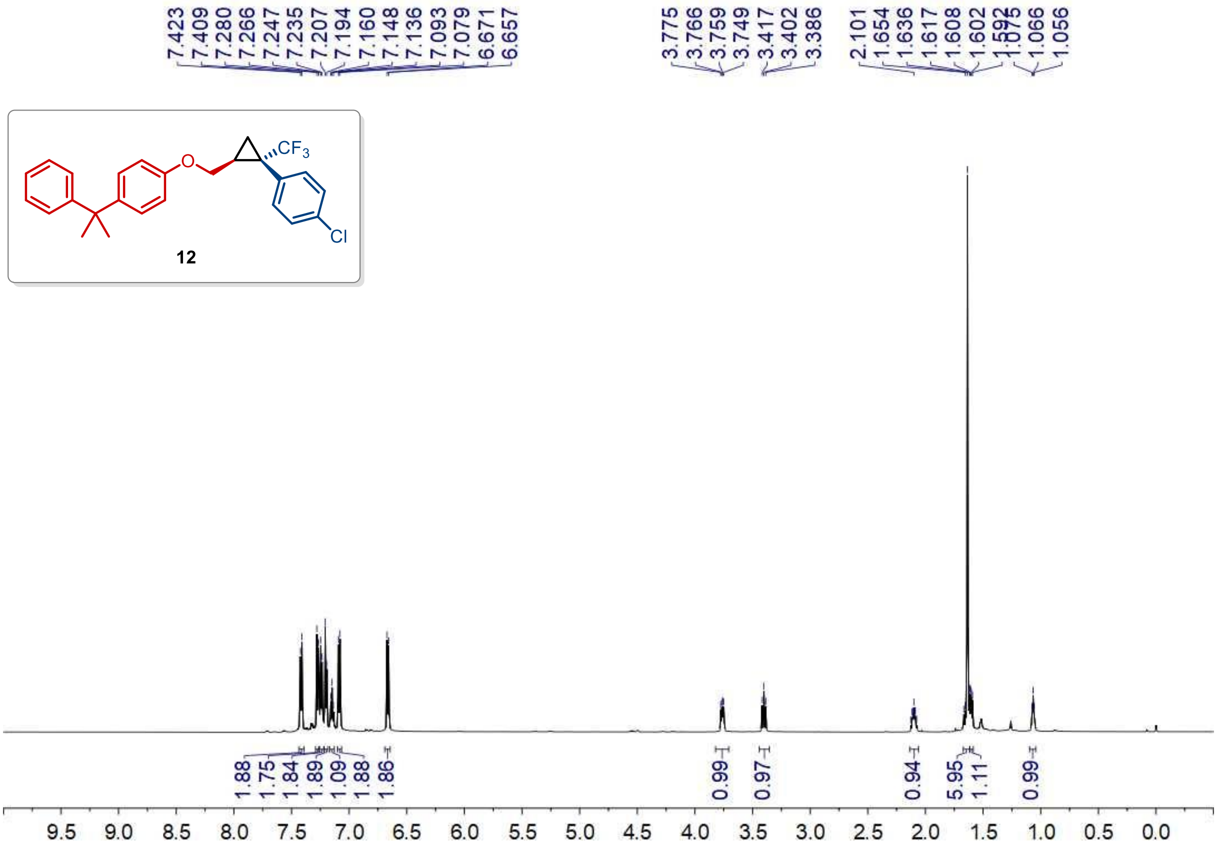

Supplementary Fig. 30 ^{1}H NMR (600 MHz, $CDCl_3$) spectrum of compound **12**.

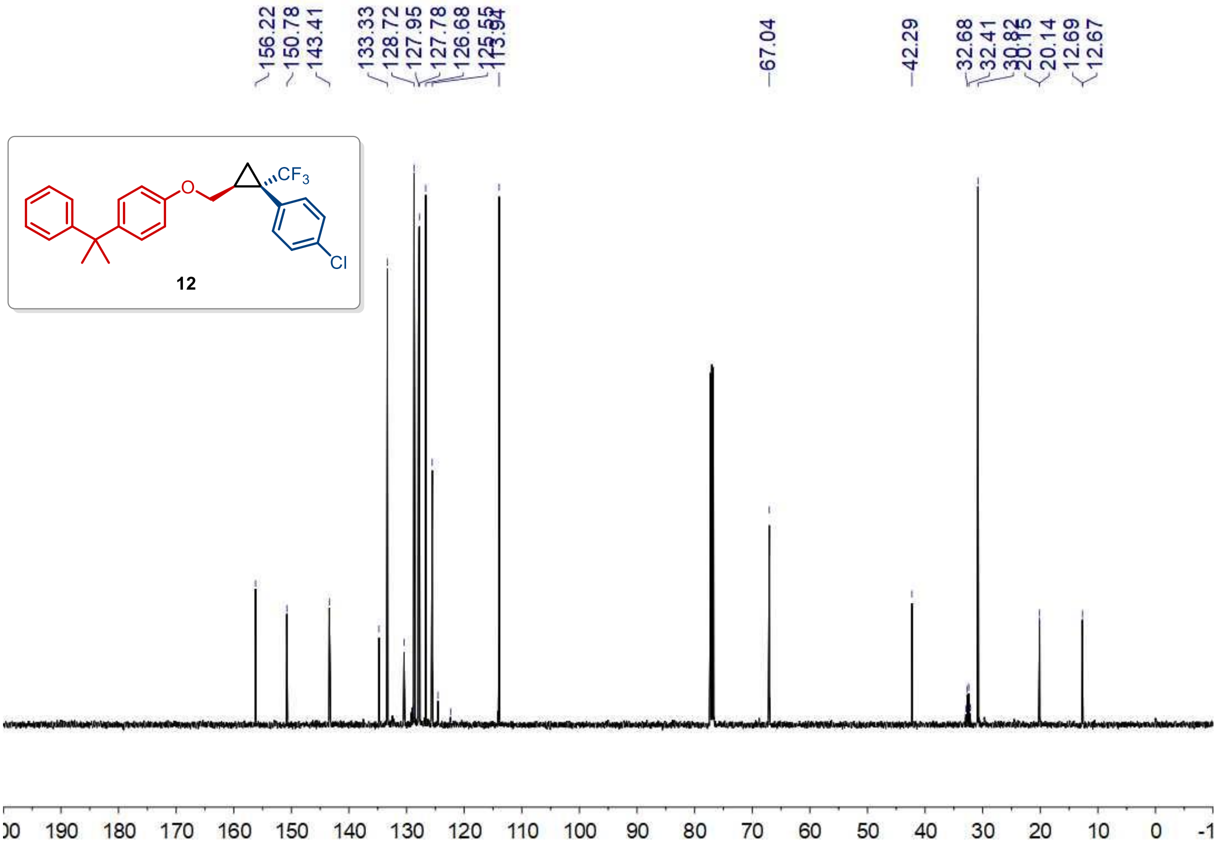

Supplementary Fig. 31 ^{13}**C NMR** (125 MHz, $CDCl_3$) spectrum of compound **12**.

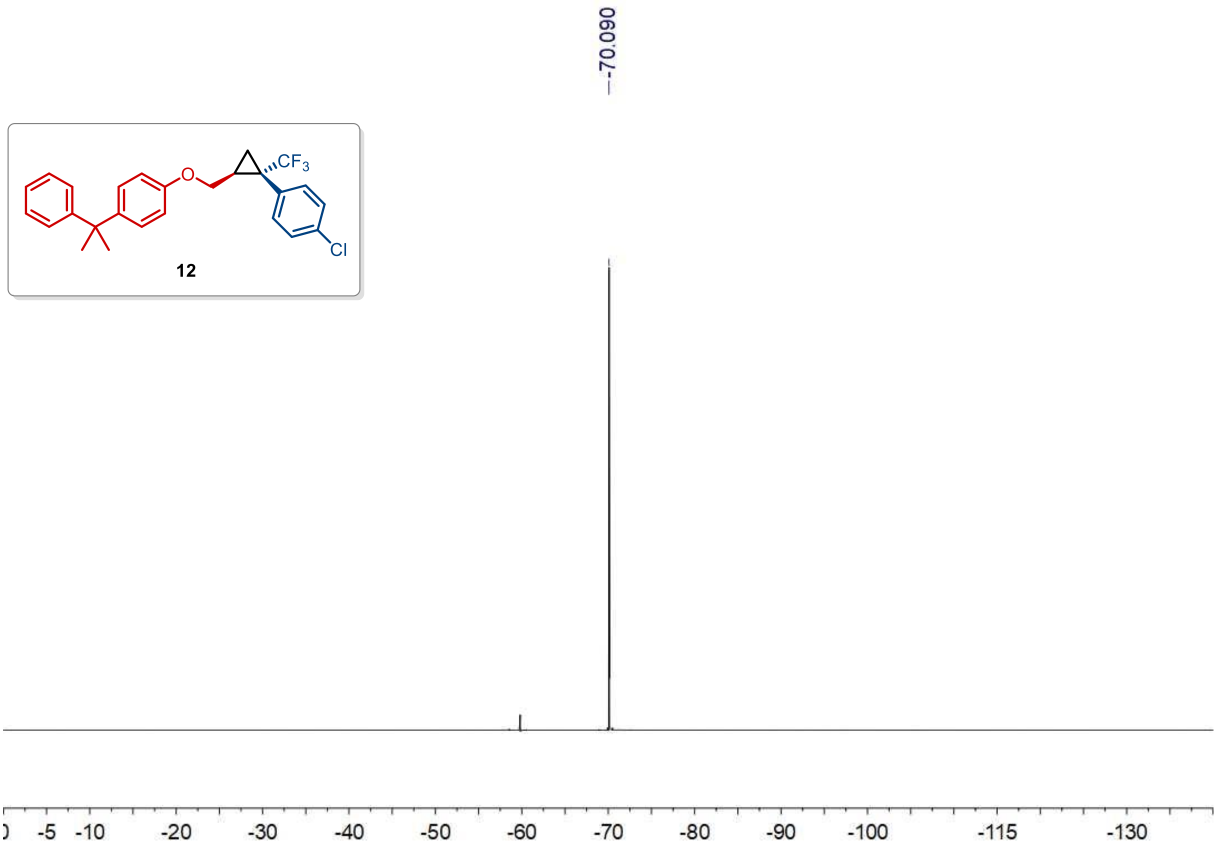

Supplementary Fig. 32 ^{19}**F NMR** (470 MHz, $CDCl_3$) spectrum of compound **12**.

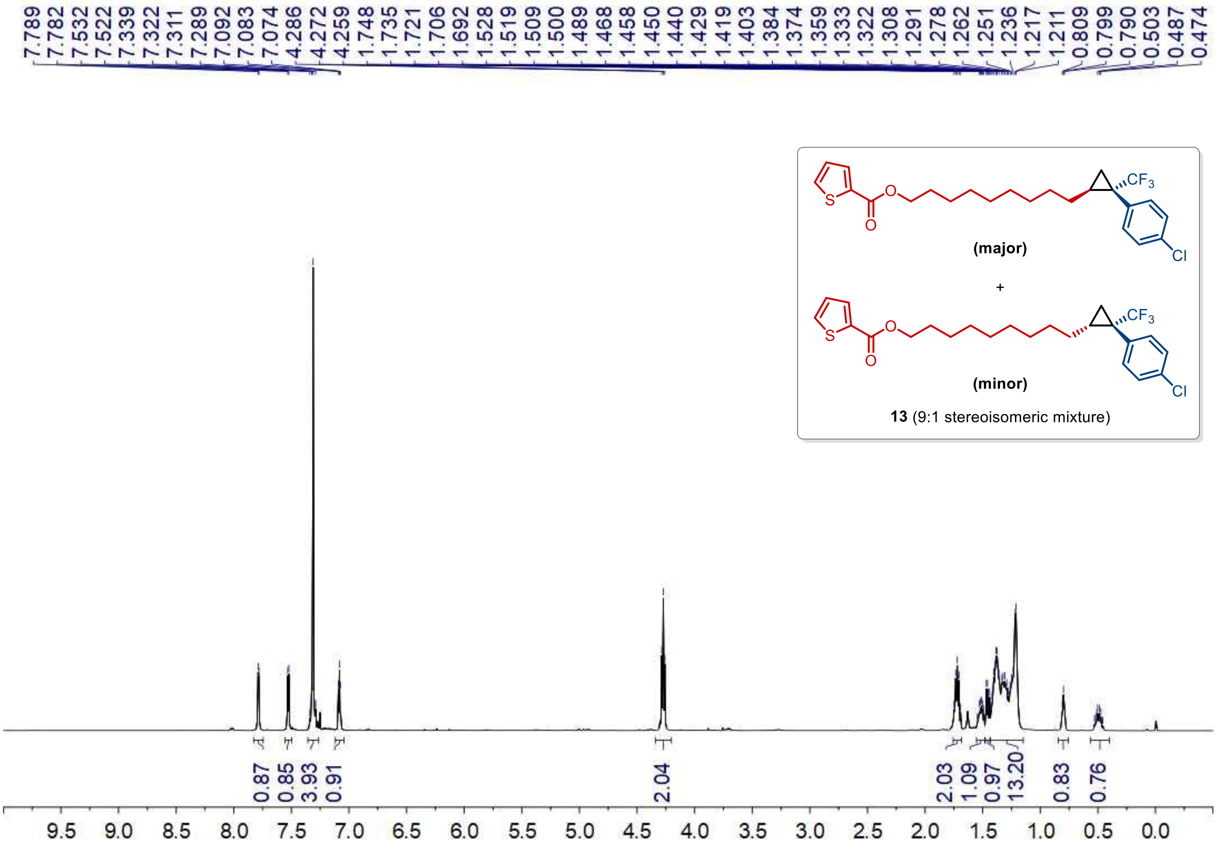

Supplementary Fig. 33 ^{1}**H NMR** (500 MHz, $CDCl_3$) spectrum of compound **13**.

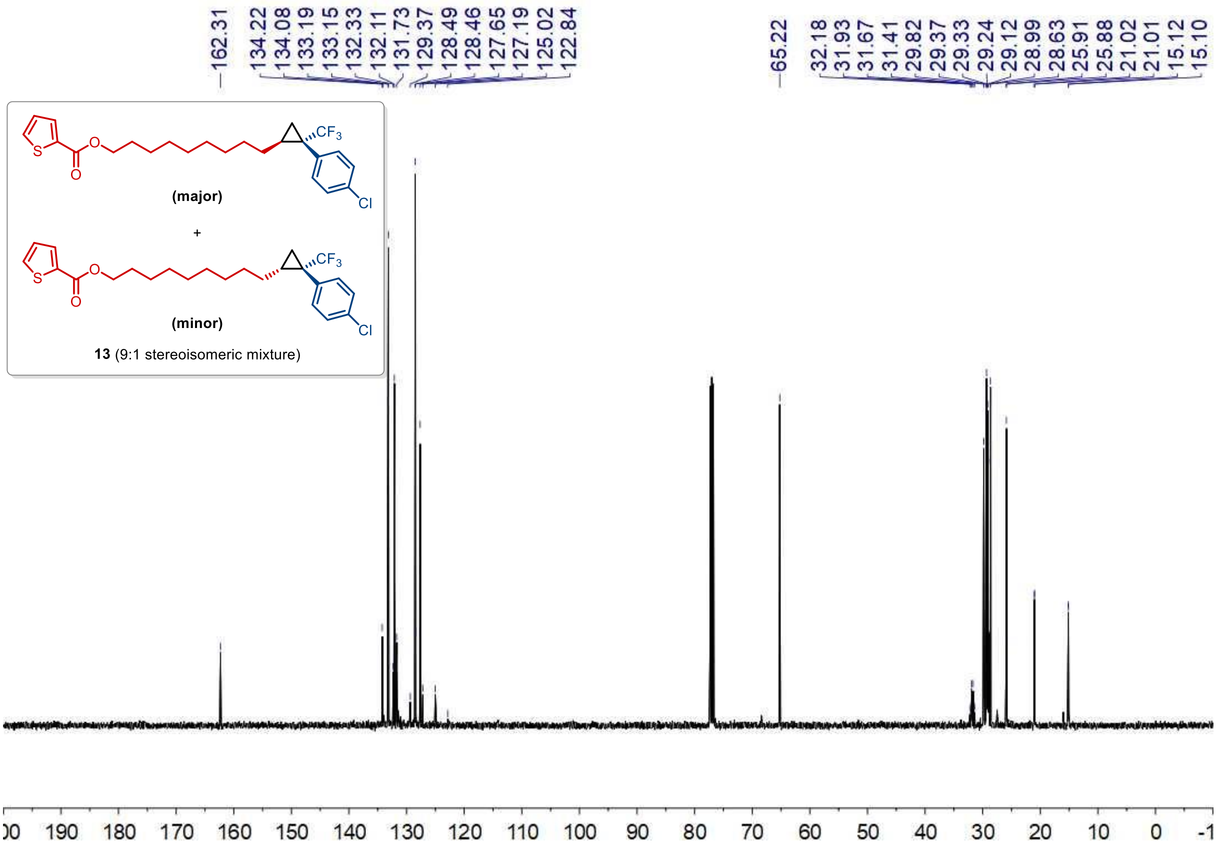

Supplementary Fig. 34 ^{13}**C NMR** (125 MHz, $CDCl_3$) spectrum of compound **13**.

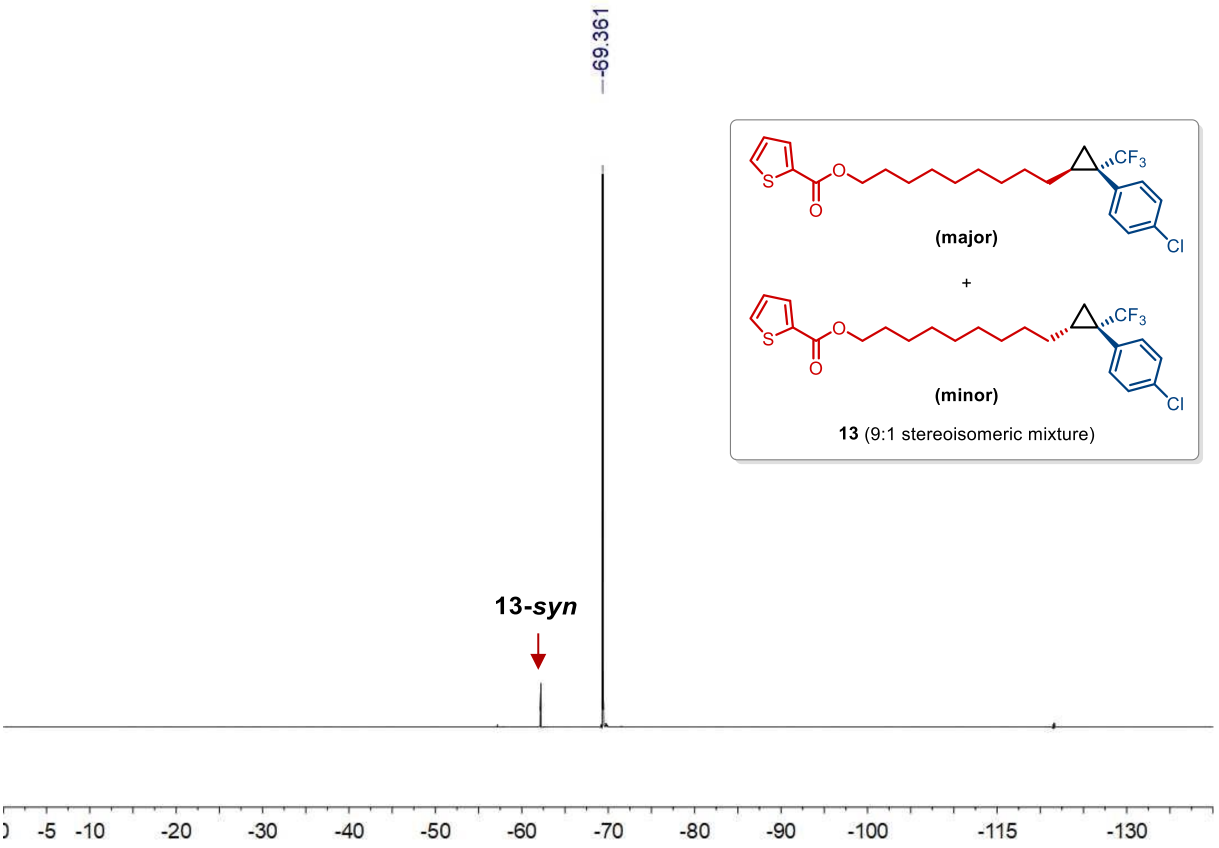

Supplementary Fig. 35 ^{19}**F NMR** (470 MHz, $CDCl_3$) spectrum of compound **13**.

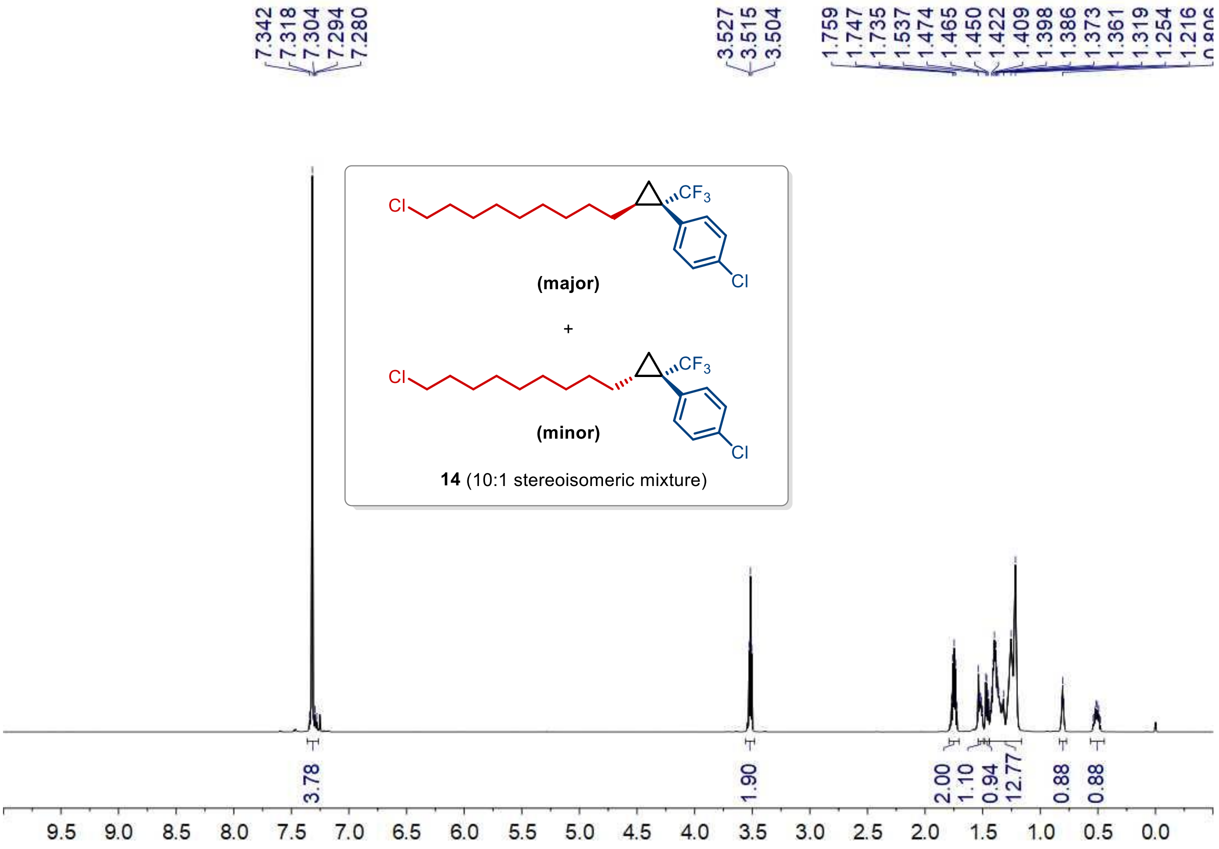

Supplementary Fig. 36 ^{1}**H NMR** (600 MHz, $CDCl_3$) spectrum of compound **14**.

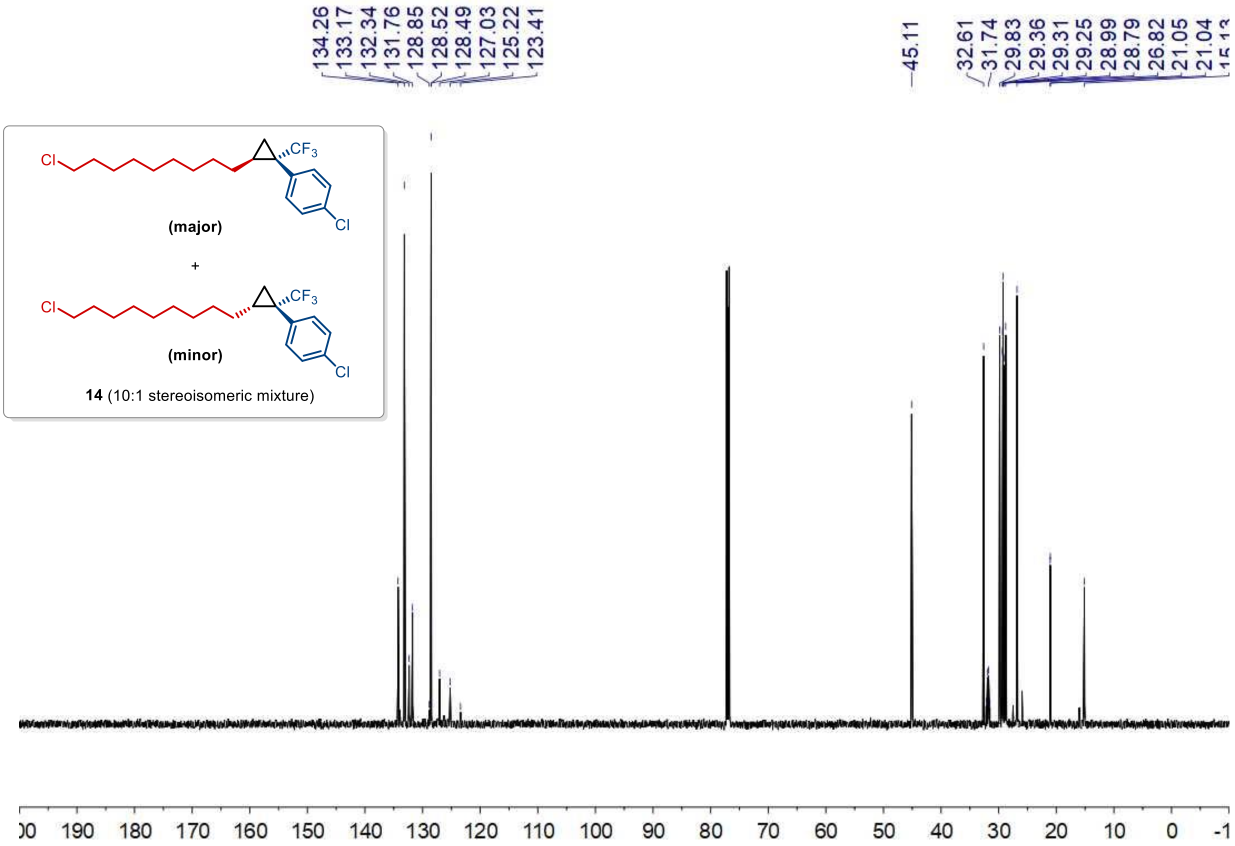

Supplementary Fig. 37 ^{13}**C NMR** (150 MHz, $CDCl_3$) spectrum of compound **14**.

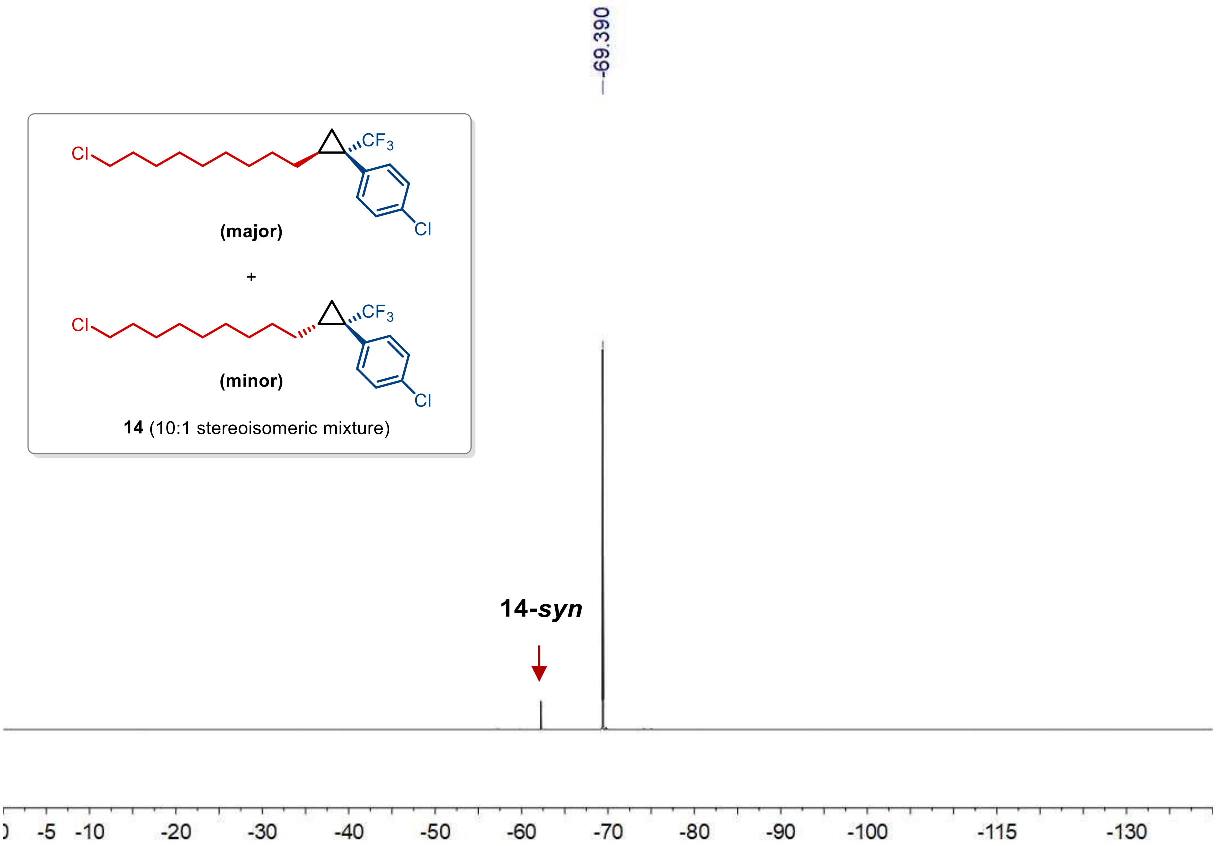

Supplementary Fig. 38 ^{19}**F NMR** (564 MHz, $CDCl_3$) spectrum of compound **14**.

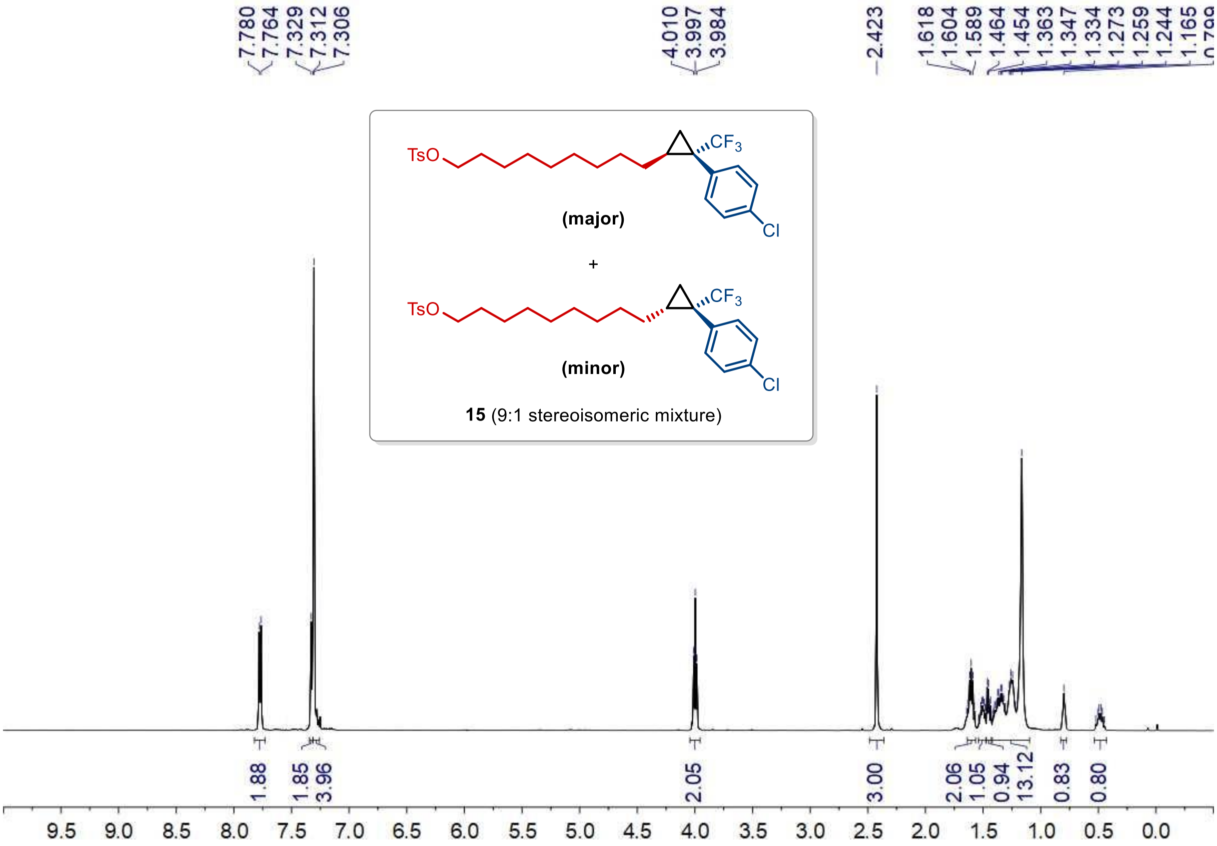

Supplementary Fig. 39 ^{1}H NMR (500 MHz, $CDCl_3$) spectrum of compound **15**.

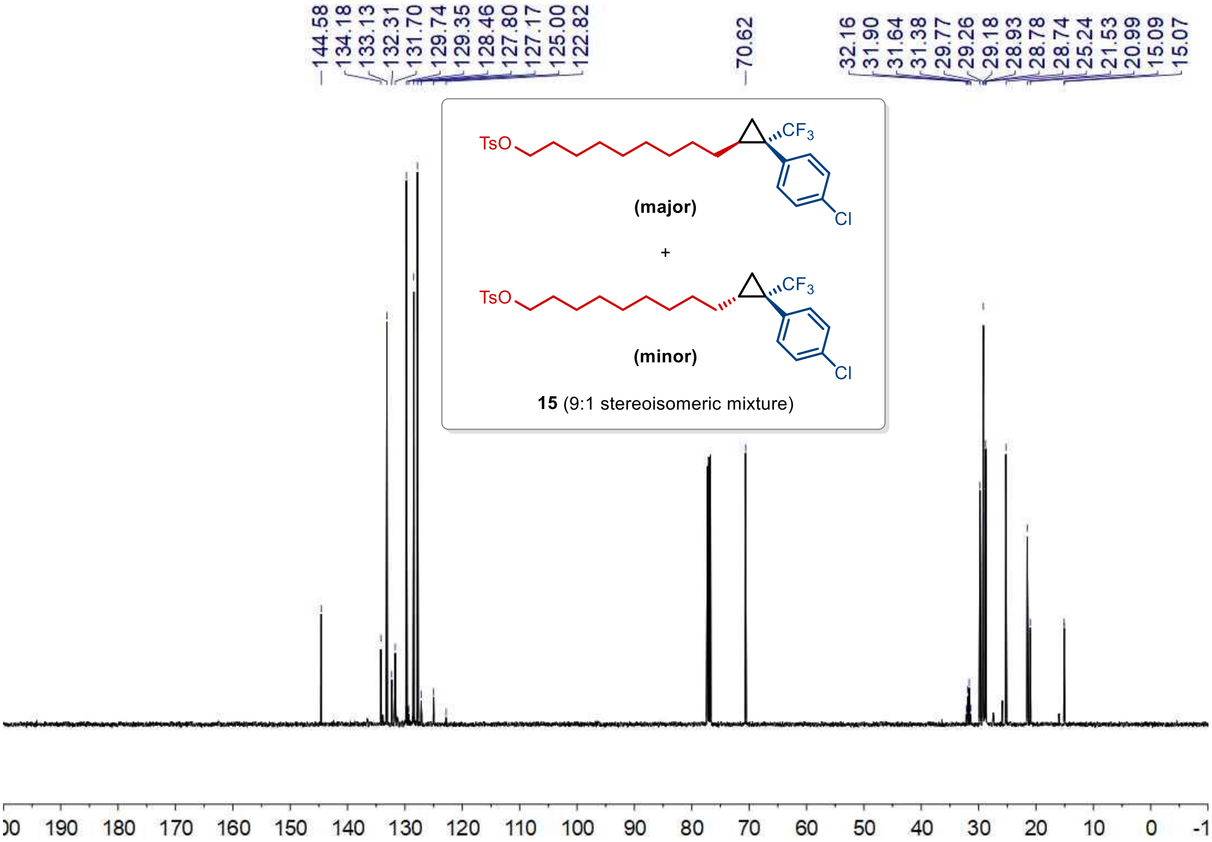

Supplementary Fig. 40 ^{13}C NMR (125 MHz, $CDCl_3$) spectrum of compound **15**.

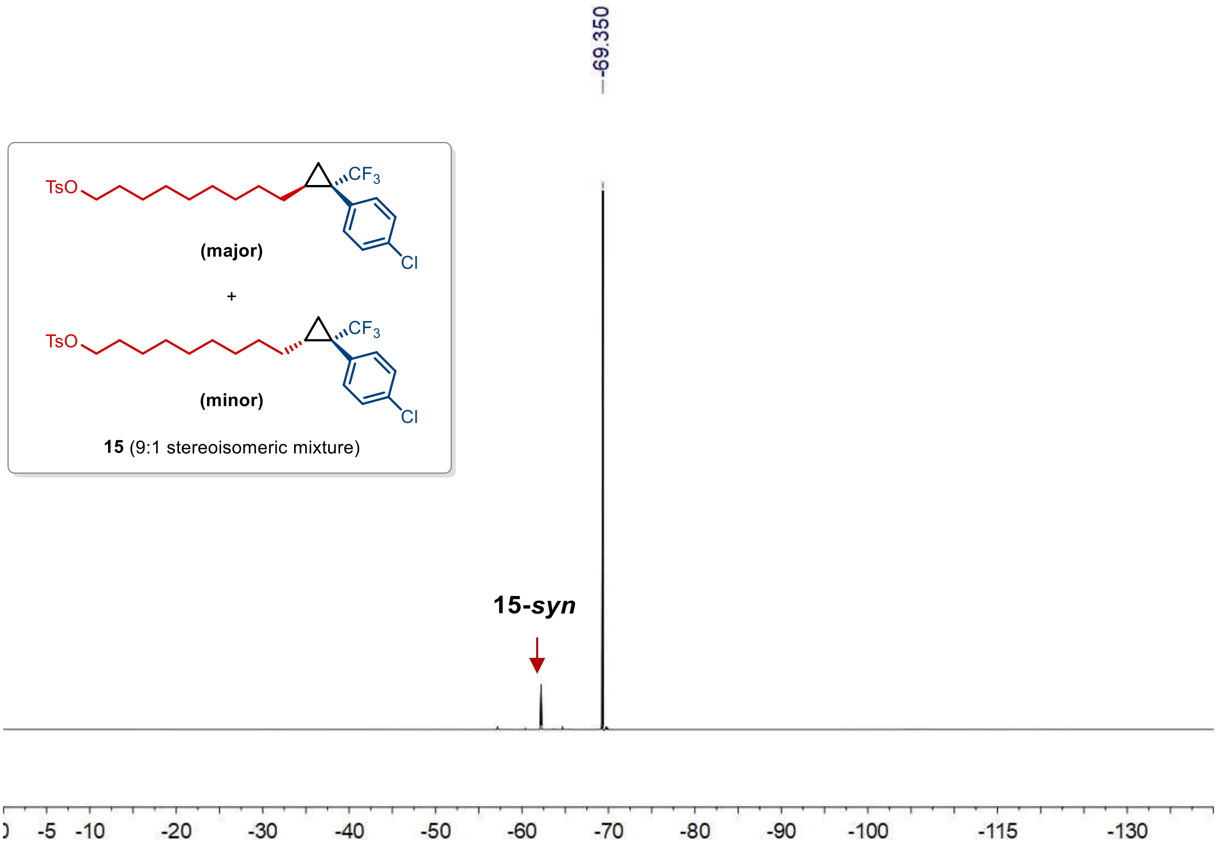

Supplementary Fig. 41 **^{19}F NMR** (470 MHz, $CDCl_3$) spectrum of compound **15**.

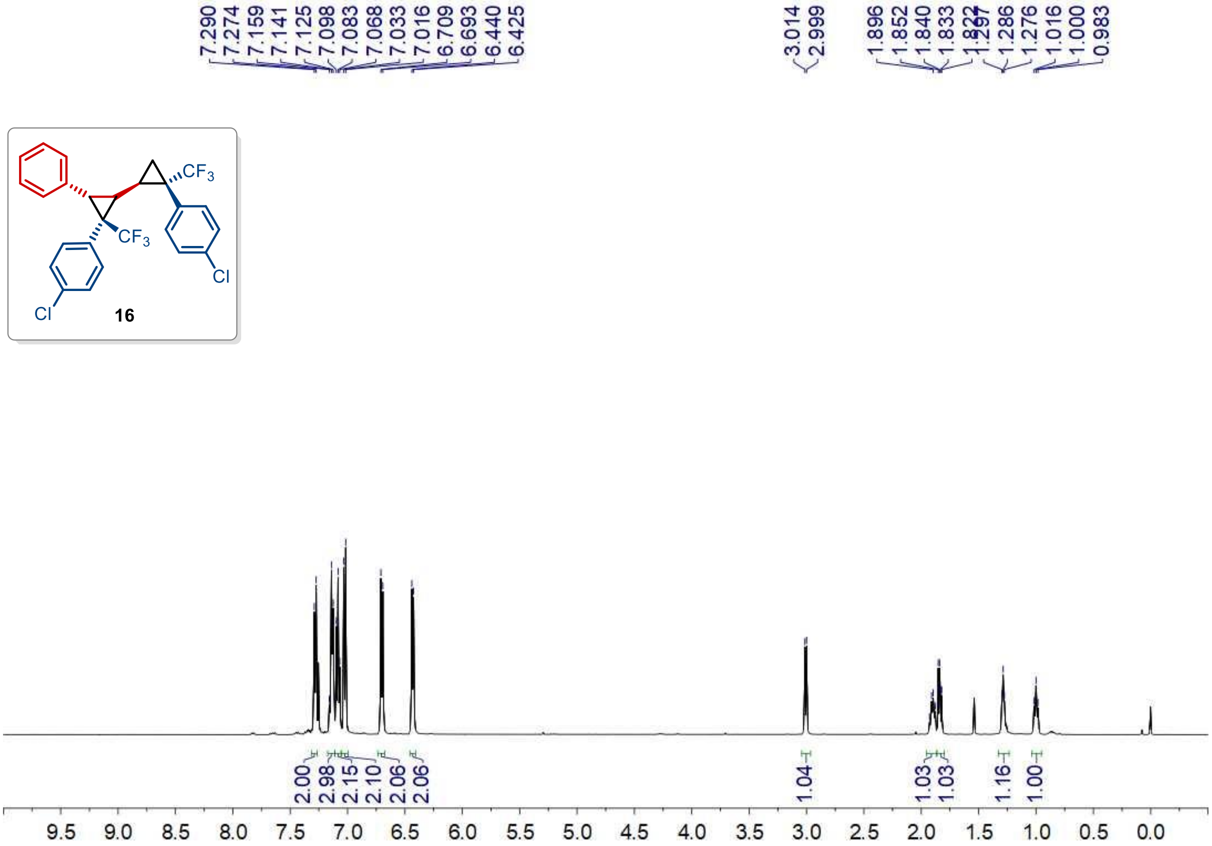

Supplementary Fig. 42 **^{1}H NMR** (500 MHz, $CDCl_3$) spectrum of compound **16**.

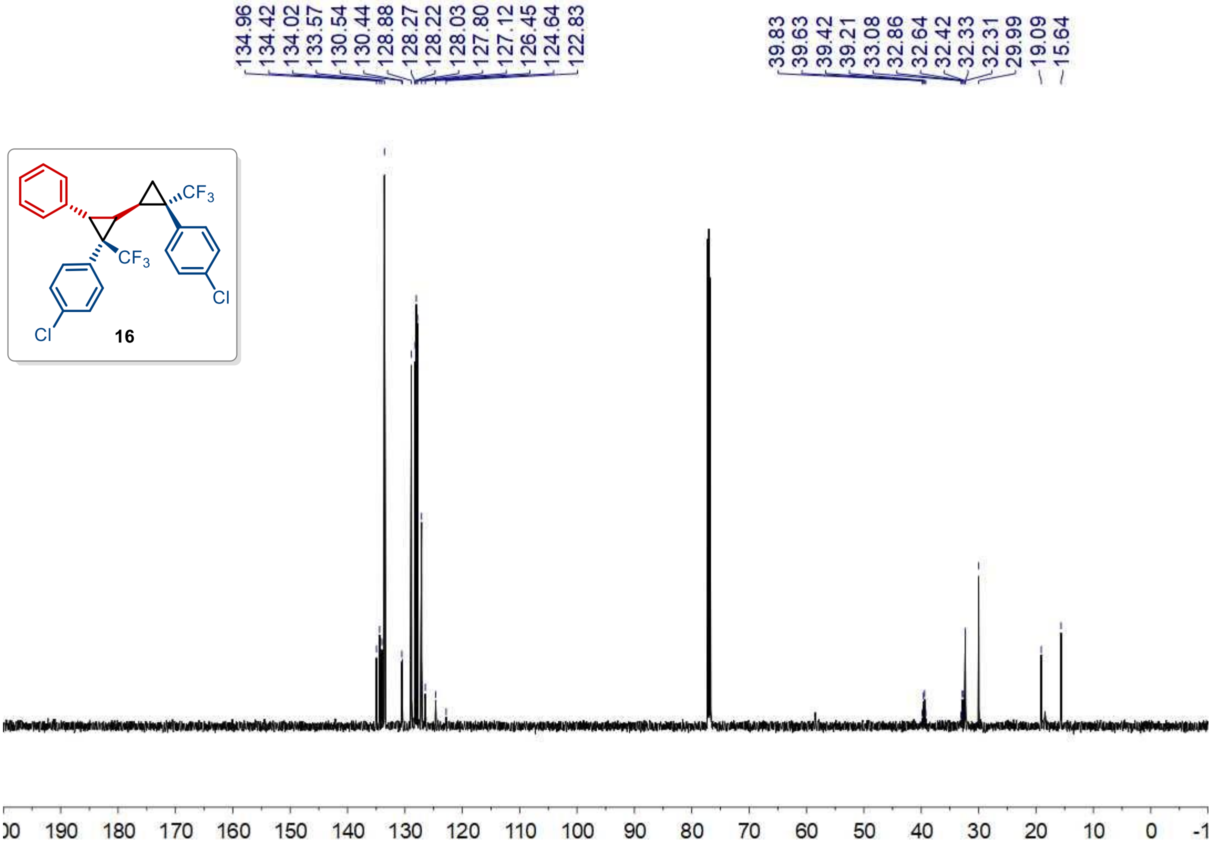

Supplementary Fig. 43 ^{13}**C NMR** (150 MHz, $CDCl_3$) spectrum of compound **16**.

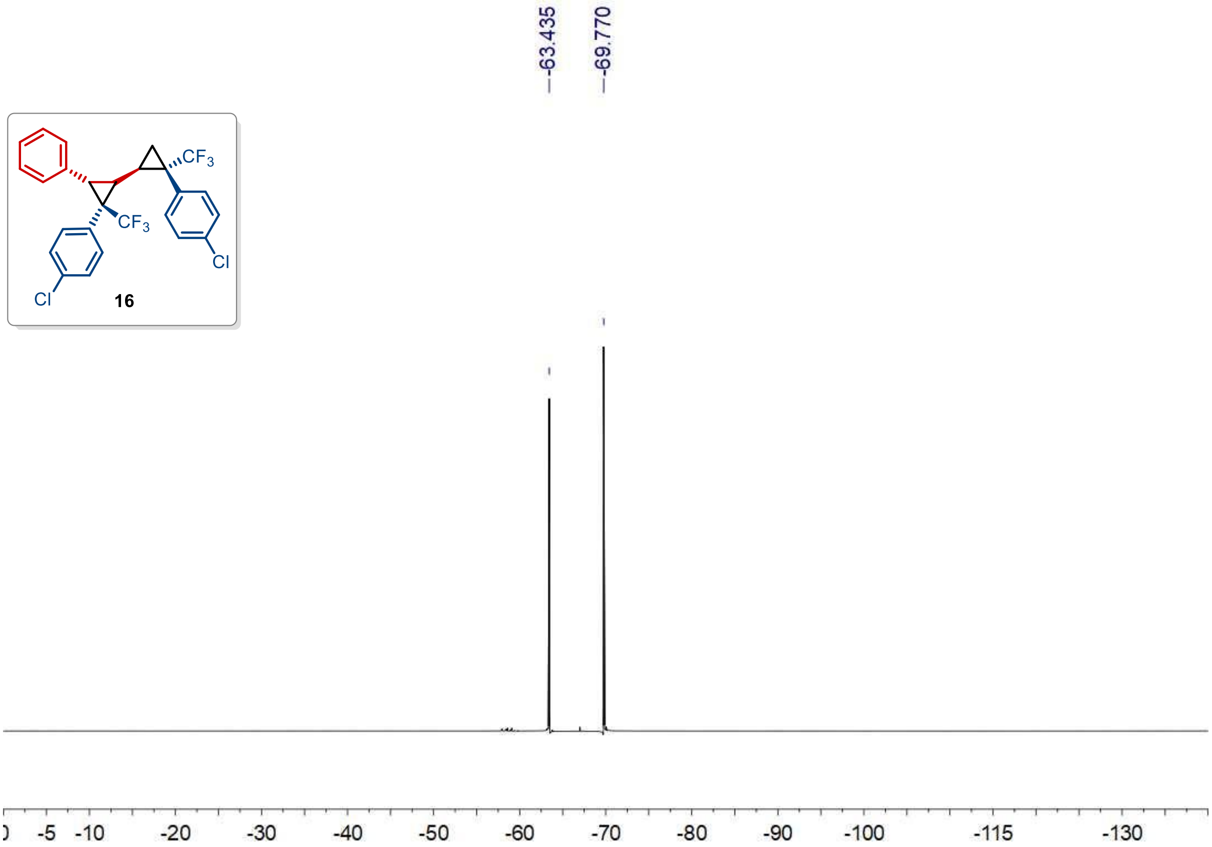

Supplementary Fig. 44 ^{19}**F NMR** (564 MHz, $CDCl_3$) spectrum of compound **16**.

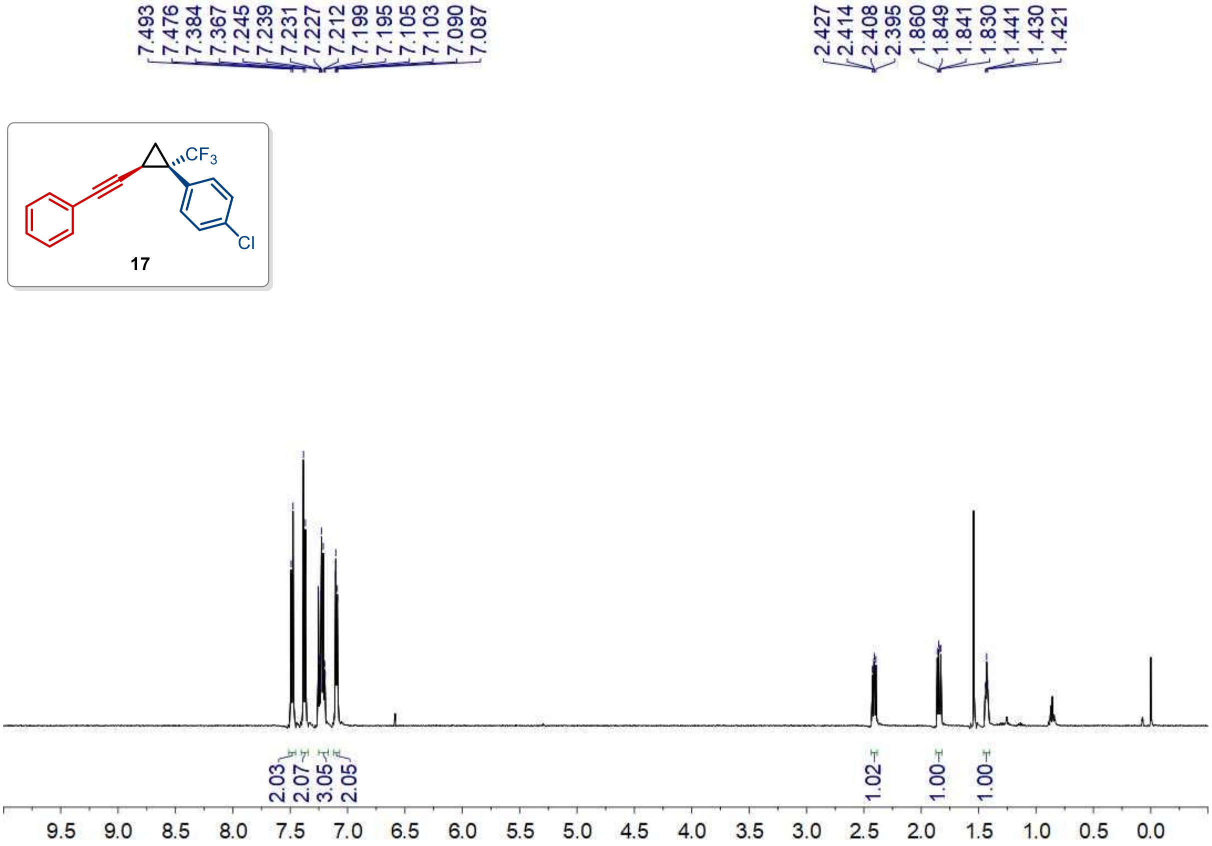

Supplementary Fig. 45 ^{1}H NMR (500 MHz, $CDCl_3$) spectrum of compound **17**.

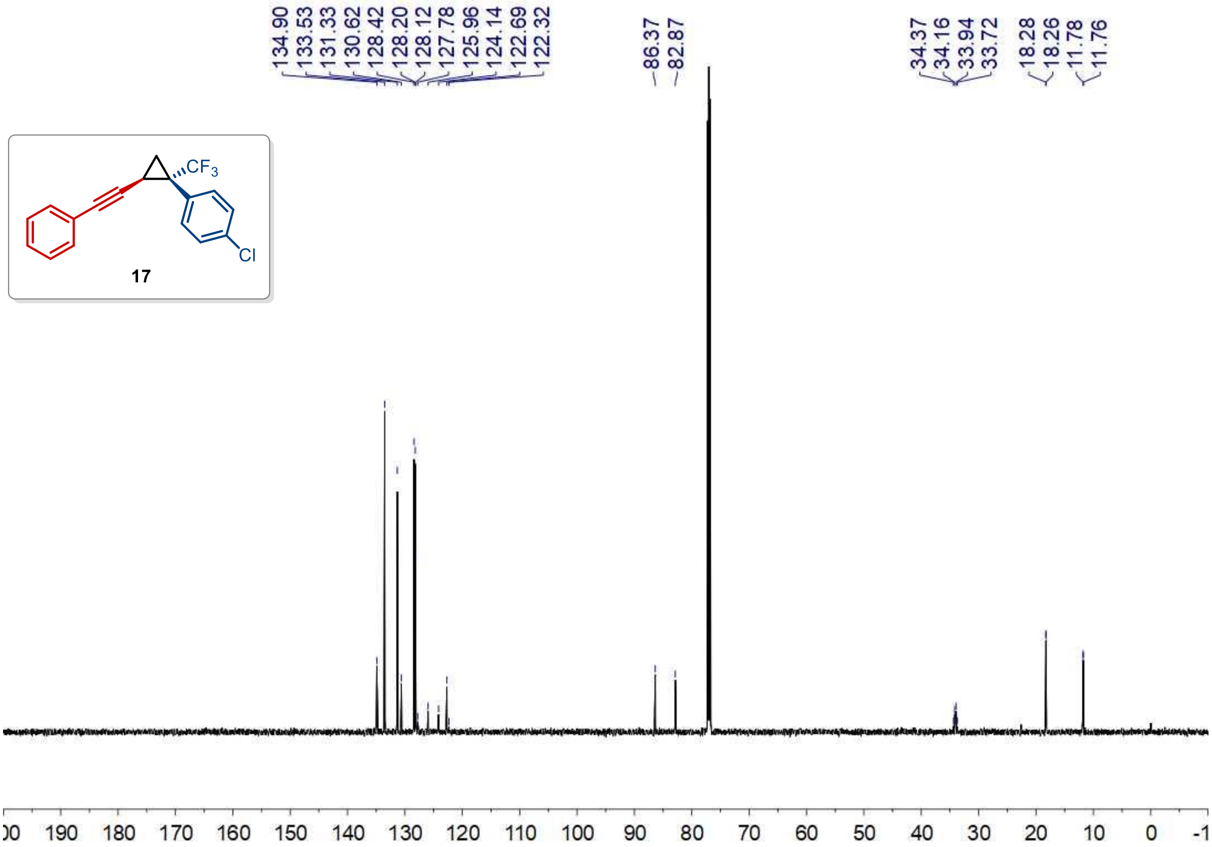

Supplementary Fig. 46 ^{13}C NMR (150 MHz, $CDCl_3$) spectrum of compound **17**.

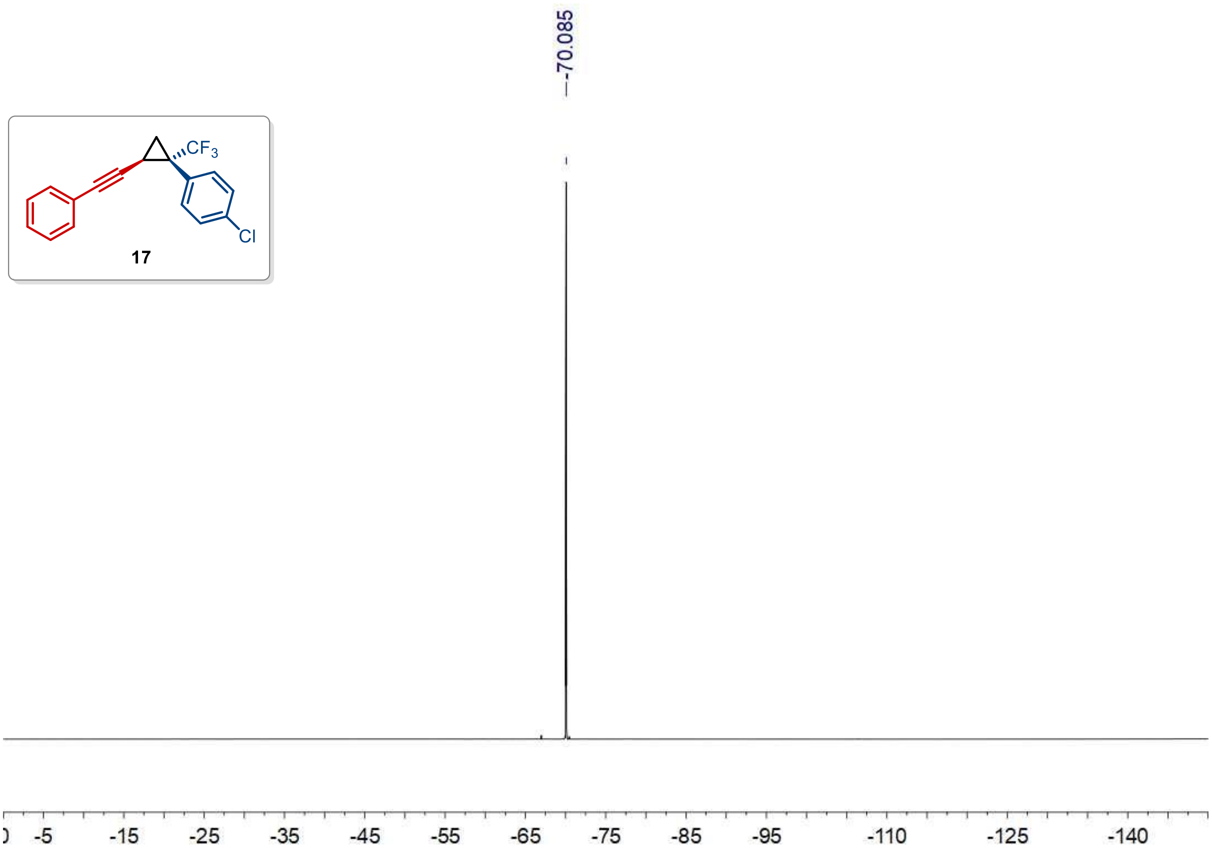

Supplementary Fig. 47 ^{19}**F NMR** (564 MHz, $CDCl_3$) spectrum of compound **17**.

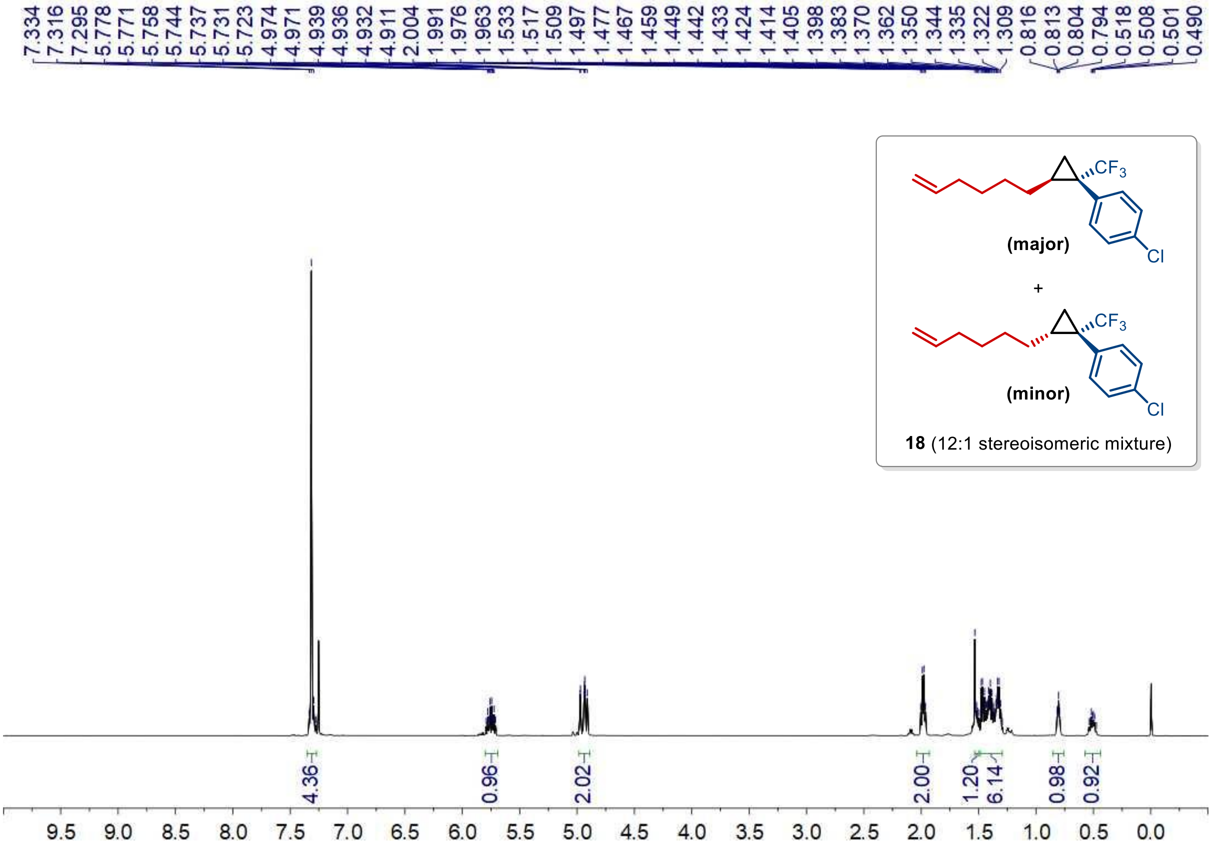

Supplementary Fig. 48 ^{1}**H NMR** (500 MHz, $CDCl_3$) spectrum of compound **18**.

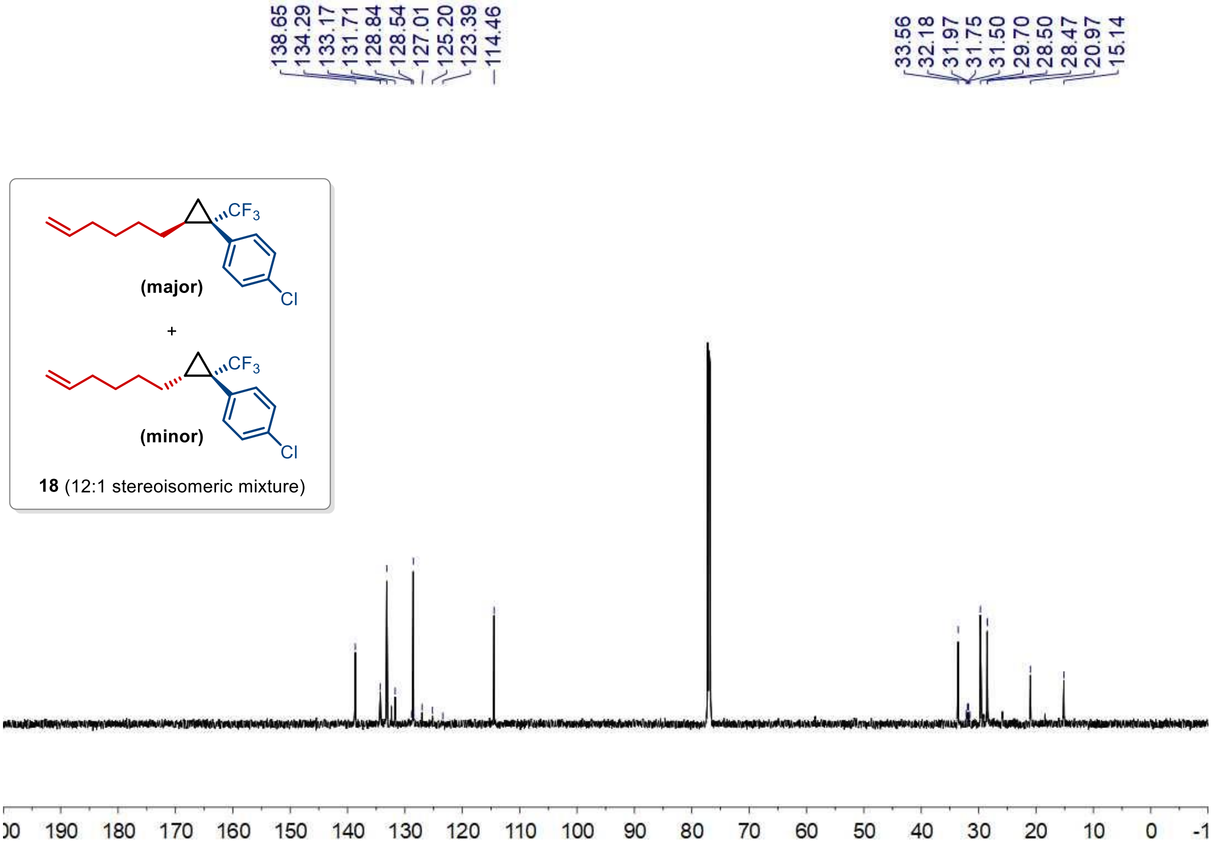

Supplementary Fig. 49 ^{13}**C NMR** (150 MHz, $CDCl_3$) spectrum of compound **18**.

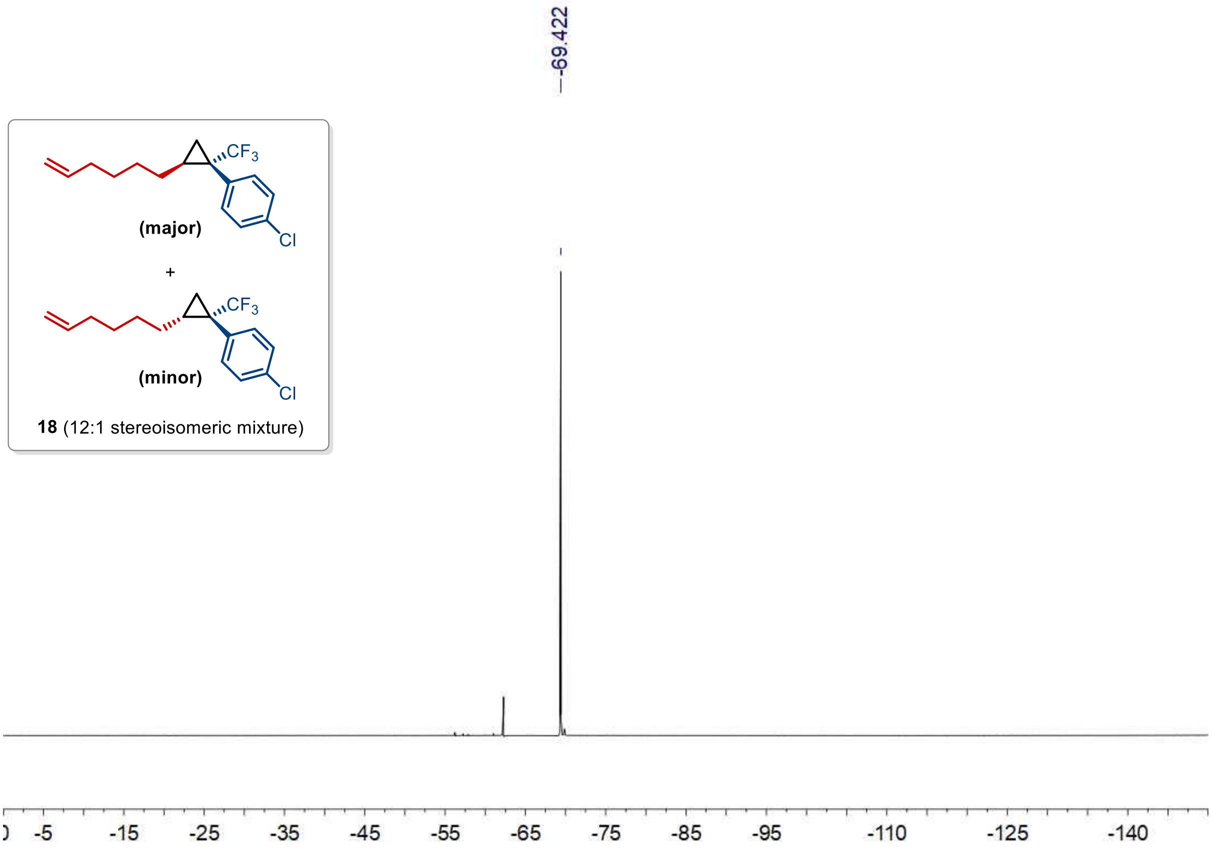

Supplementary Fig. 50 ^{19}**F NMR** (564 MHz, $CDCl_3$) spectrum of compound **18**.

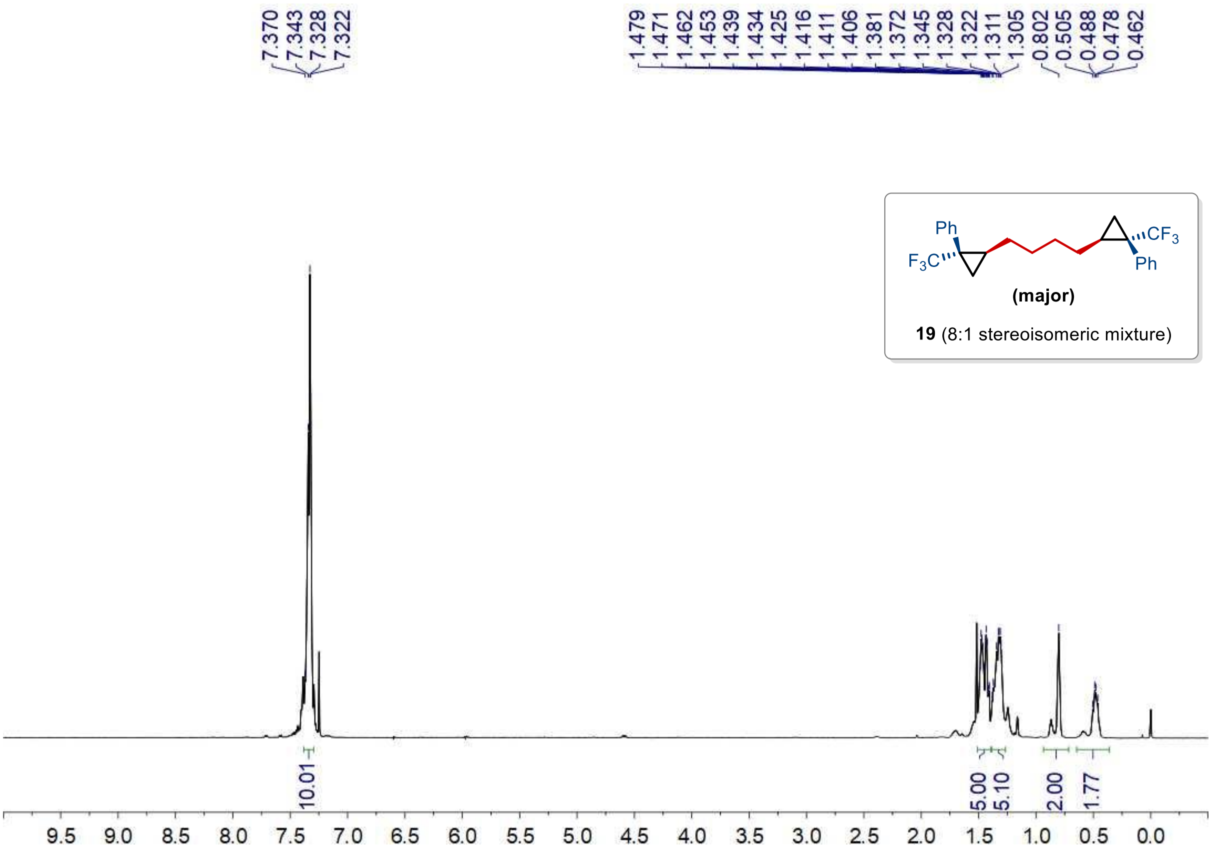

Supplementary Fig. 51 ^{1}**H NMR** (500 MHz, $CDCl_3$) spectrum of compound **19**.

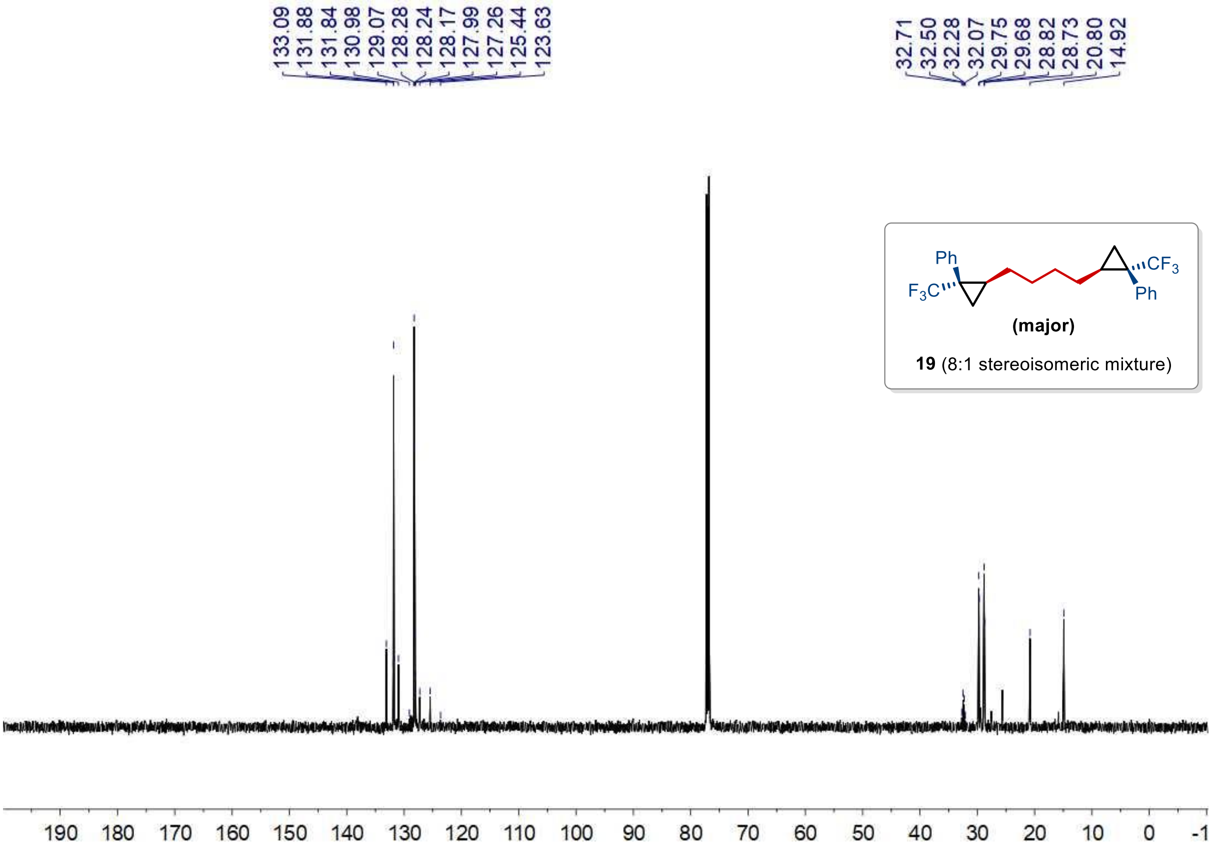

Supplementary Fig. 52 ^{13}**C NMR** (150 MHz, $CDCl_3$) spectrum of compound **19**.

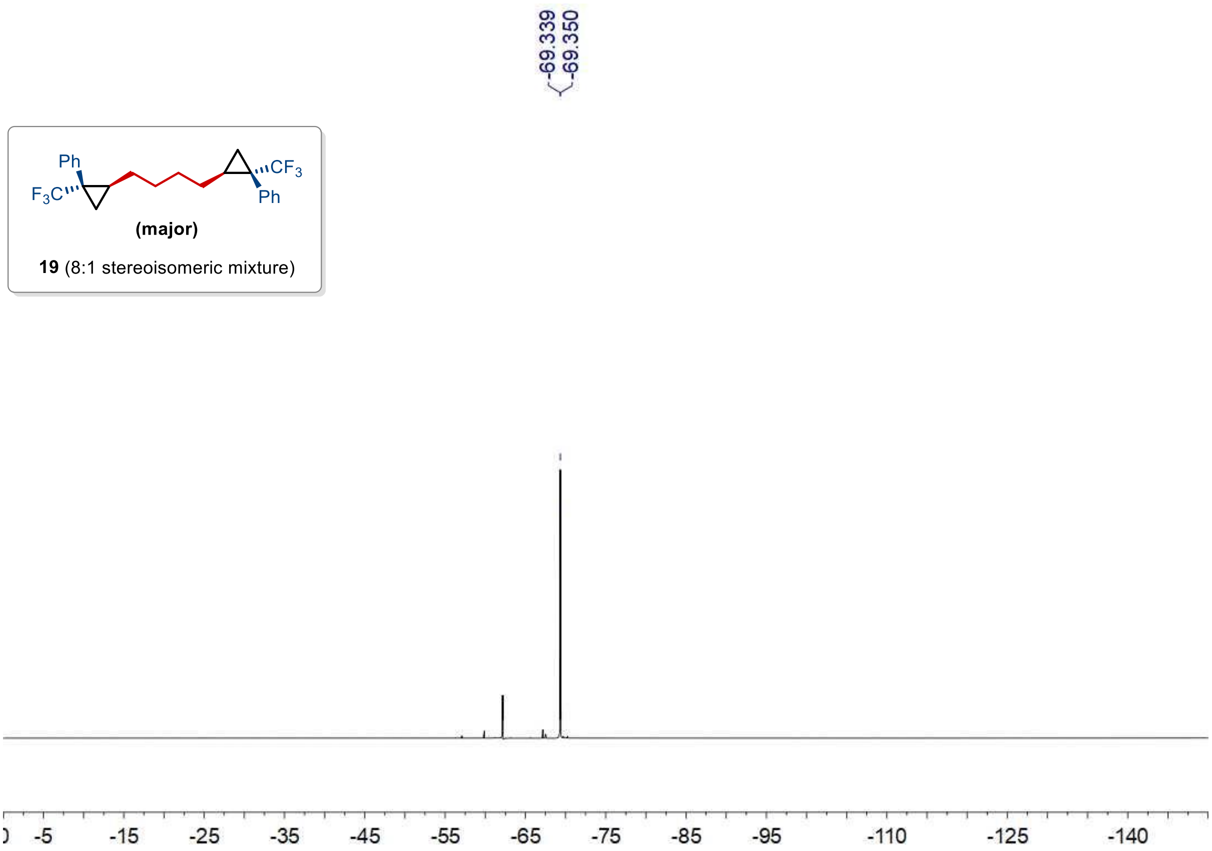

Supplementary Fig. 53 ^{19}F NMR (564 MHz, $CDCl_3$) spectrum of compound **19**.

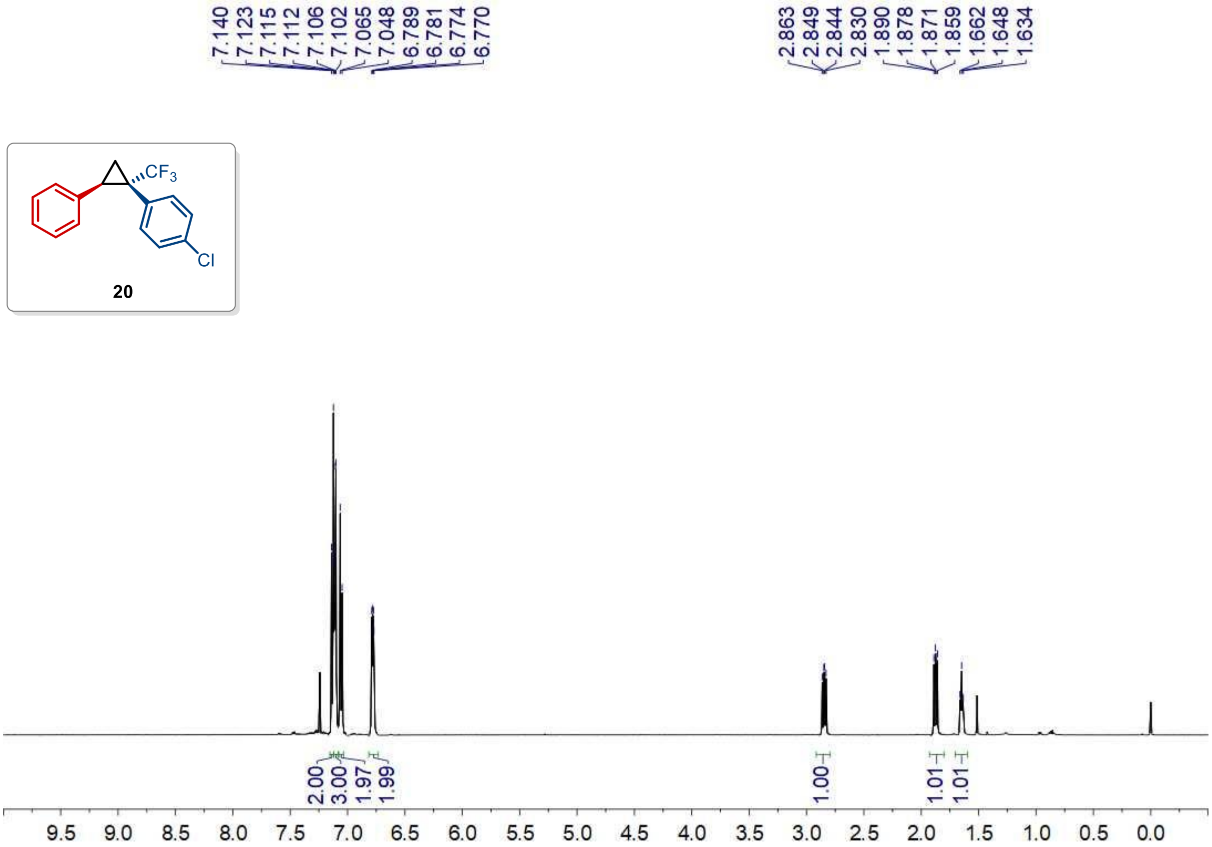

Supplementary Fig. 54 ^{1}H NMR (500 MHz, $CDCl_3$) spectrum of compound **20**.

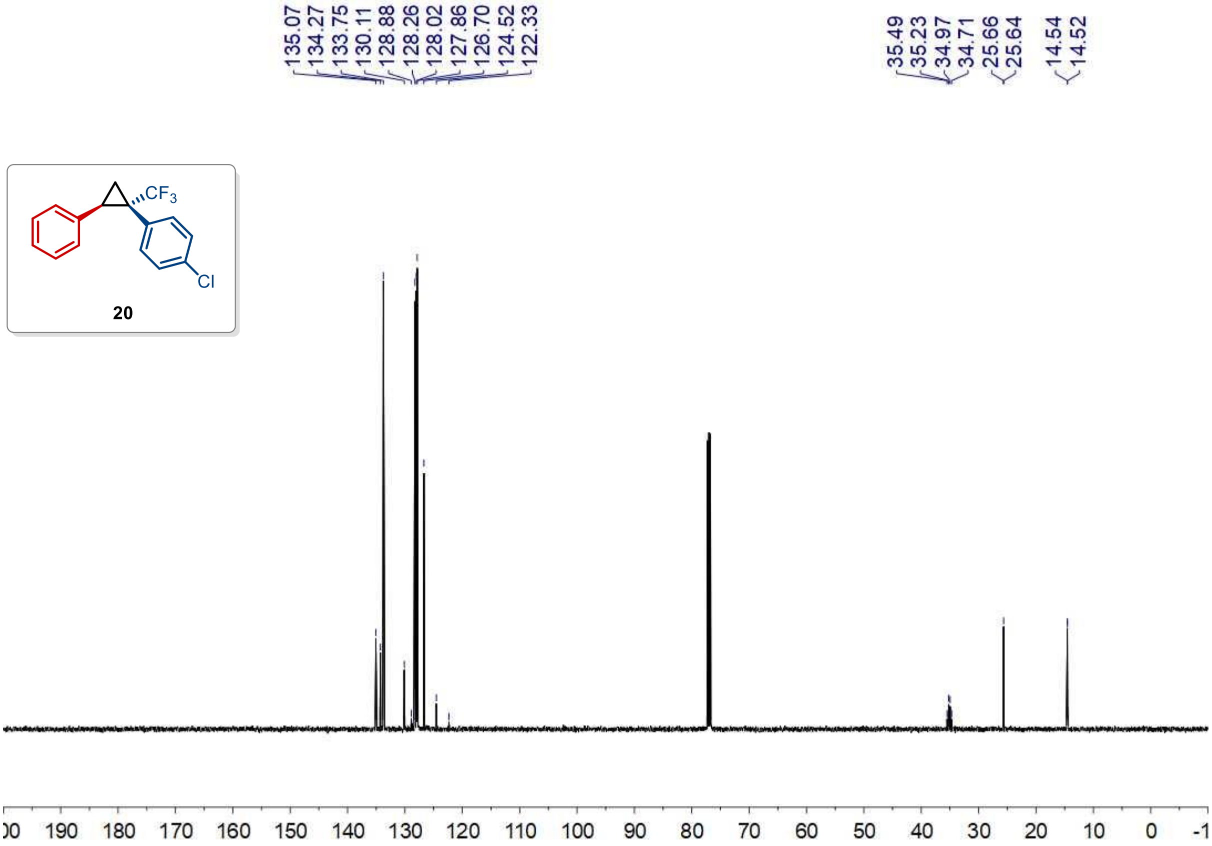

Supplementary Fig. 55 ^{13}**C NMR** (125 MHz, $CDCl_3$) spectrum of compound **20**.

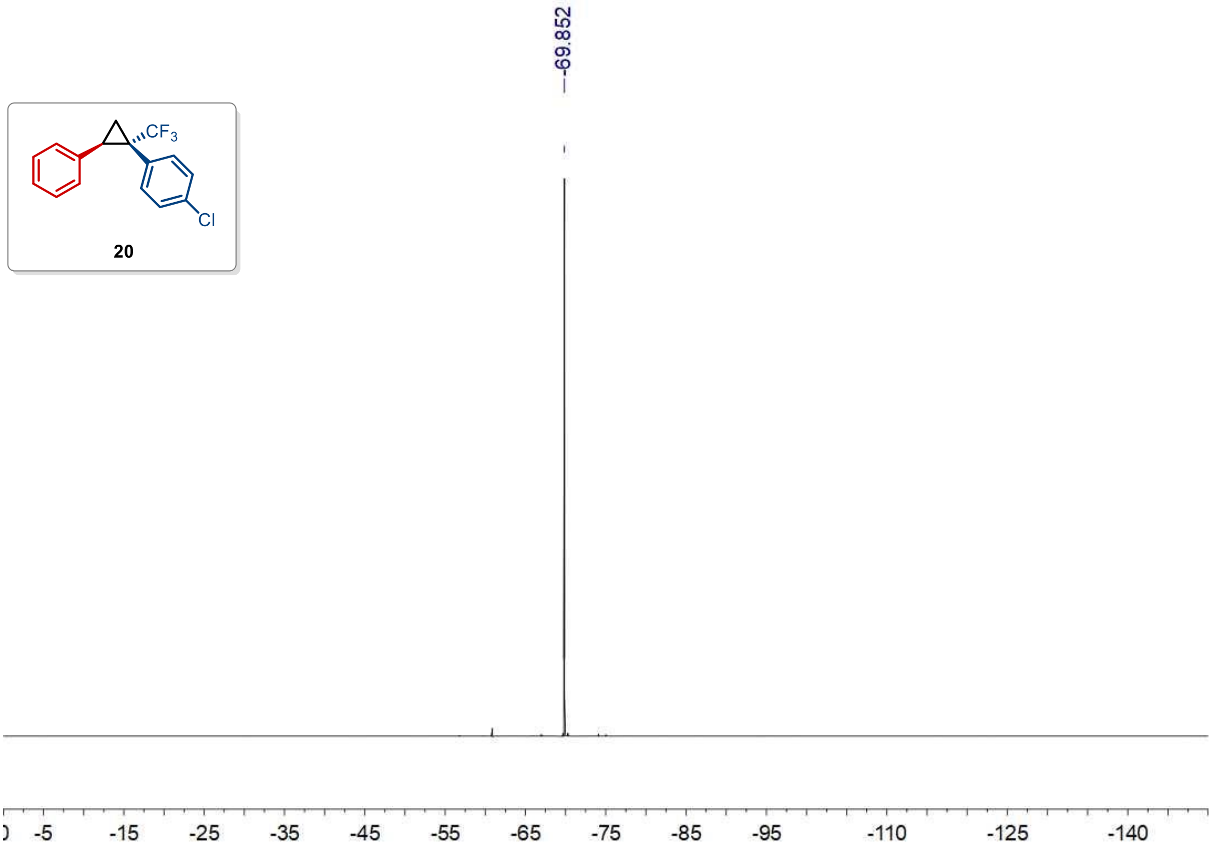

Supplementary Fig. 56 ^{19}**F NMR** (470 MHz, $CDCl_3$) spectrum of compound **20**.

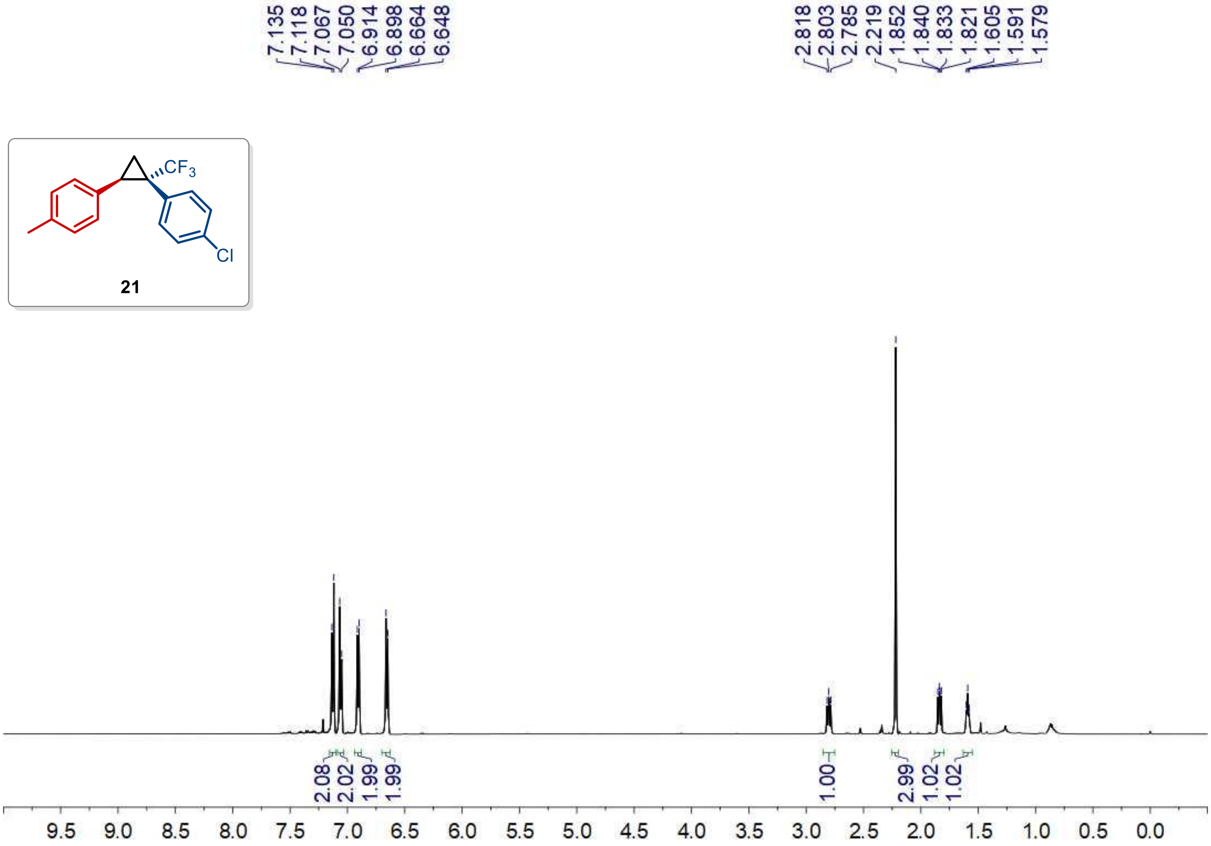

Supplementary Fig. 57 ^{1}**H NMR** (500 MHz, $CDCl_3$) spectrum of compound **21**.

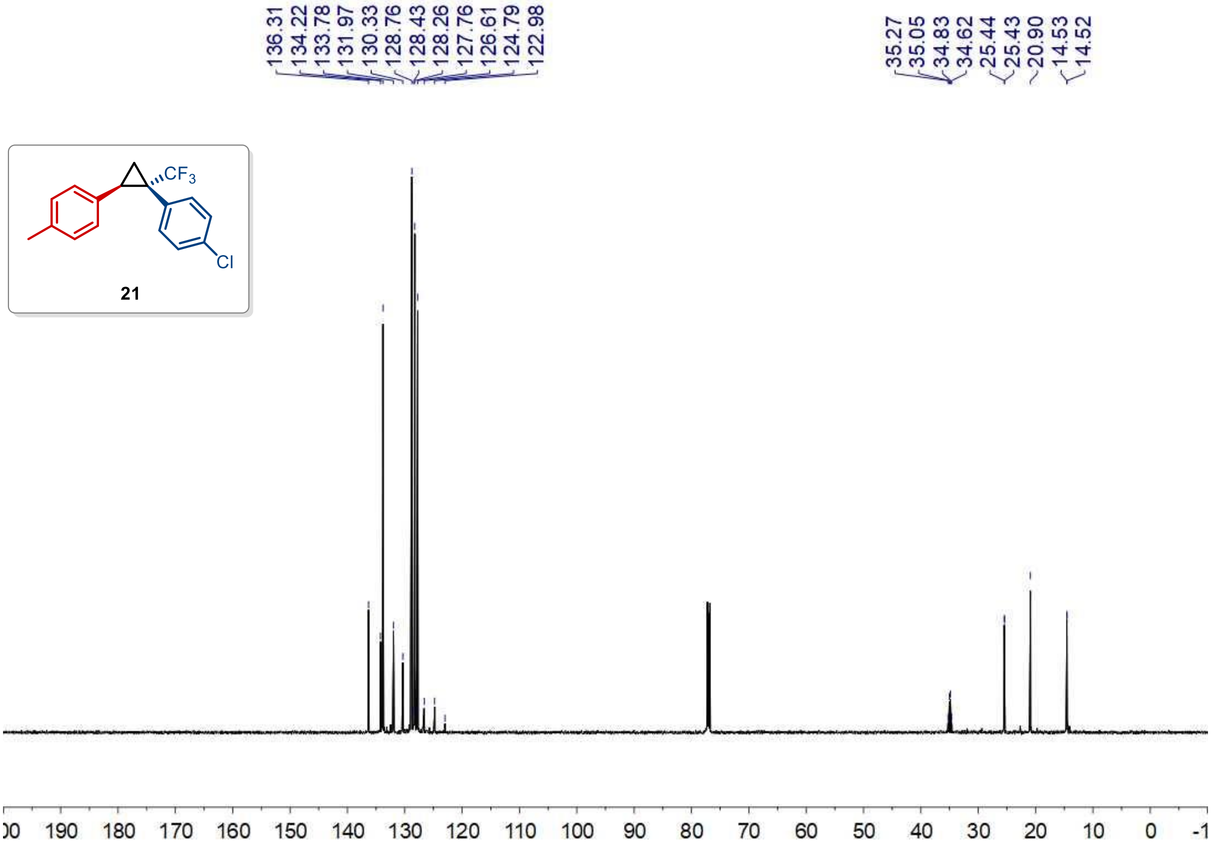

Supplementary Fig. 58 ^{13}**C NMR** (150 MHz, $CDCl_3$) spectrum of compound **21**.

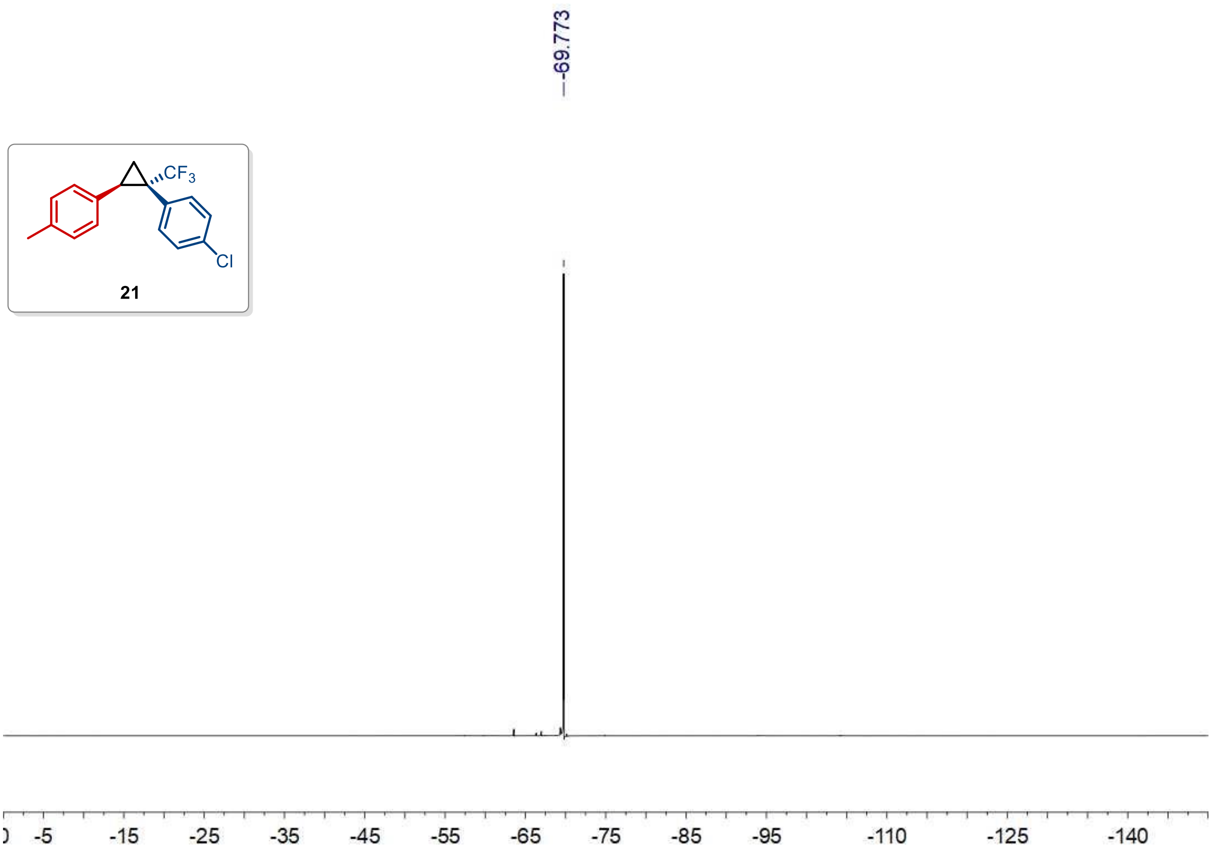

Supplementary Fig. 59 ^{19}**F NMR** (564 MHz, $CDCl_3$) spectrum of compound **21**.

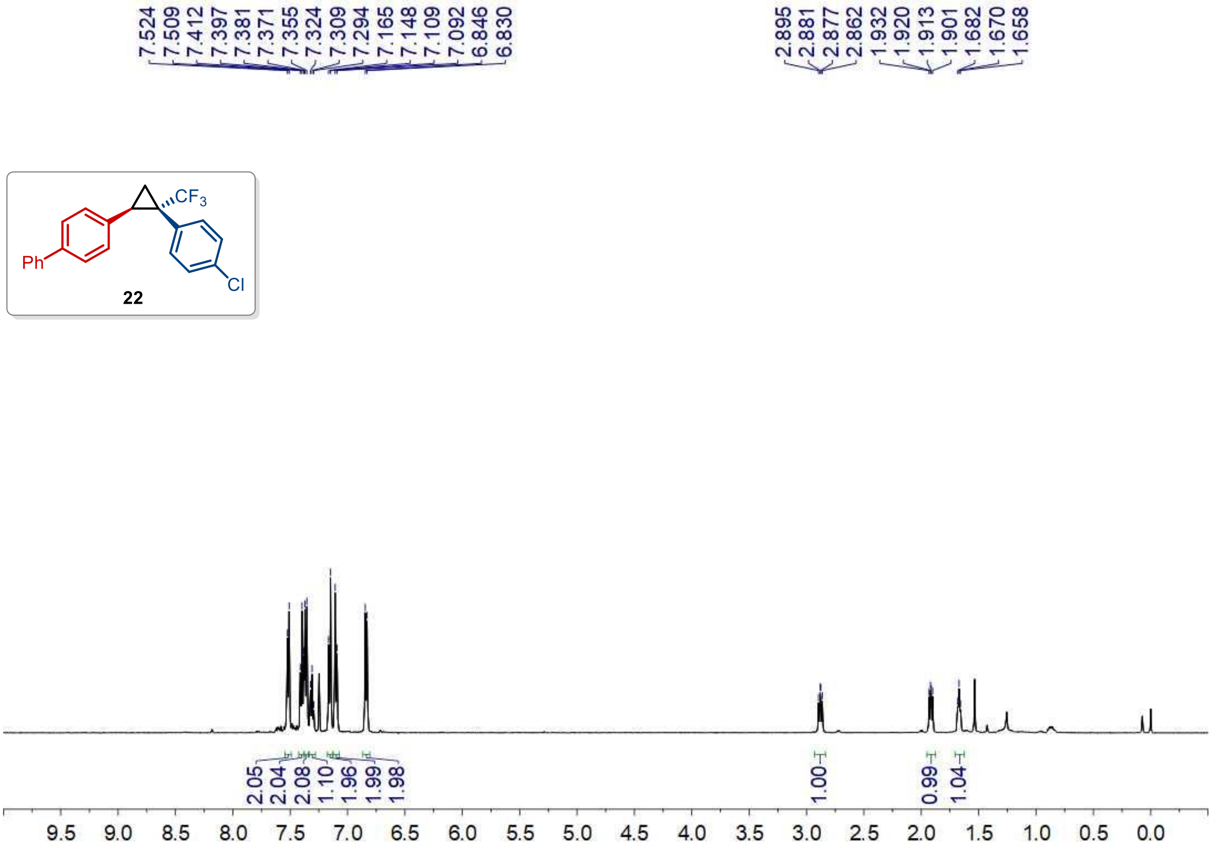

Supplementary Fig. 60 ^{1}**H NMR** (500 MHz, $CDCl_3$) spectrum of compound **22**.

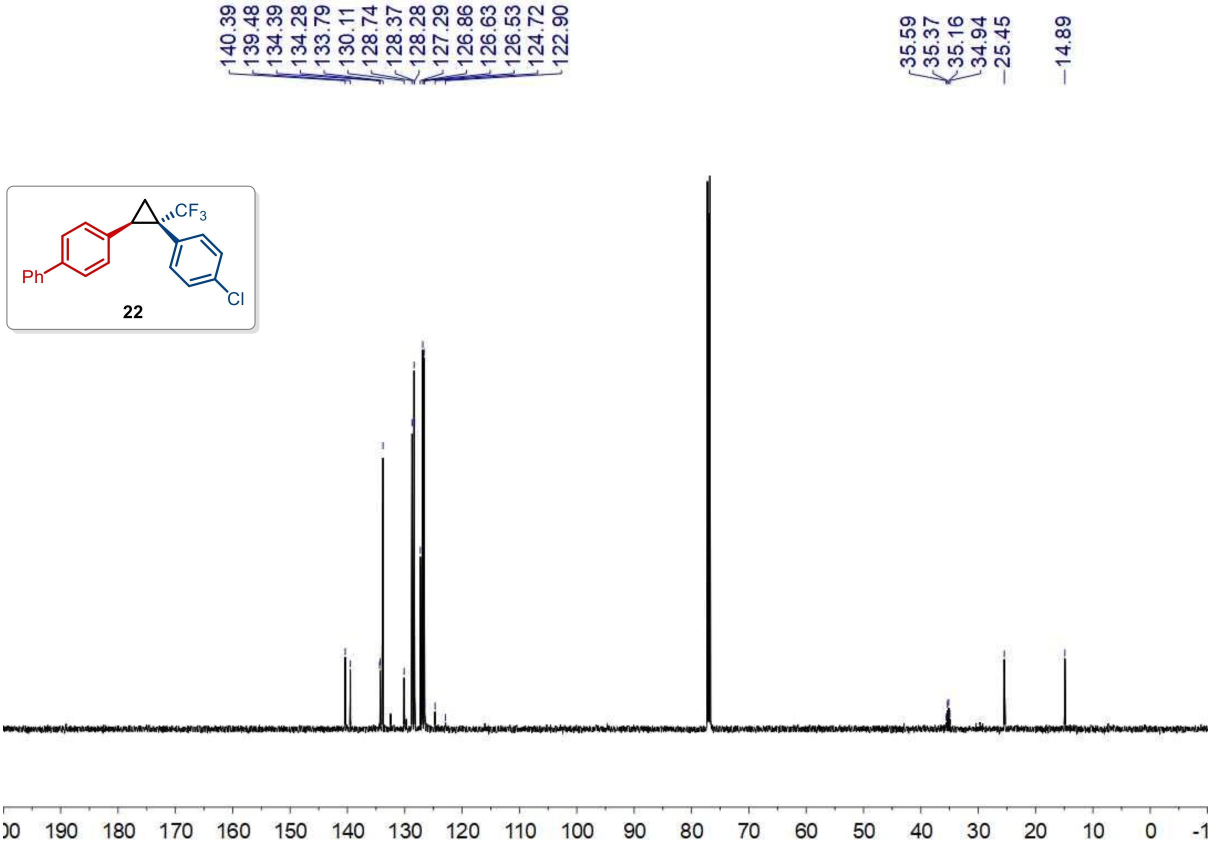

Supplementary Fig. 61 ^{13}**C NMR** (150 MHz, $CDCl_3$) spectrum of compound **22**.

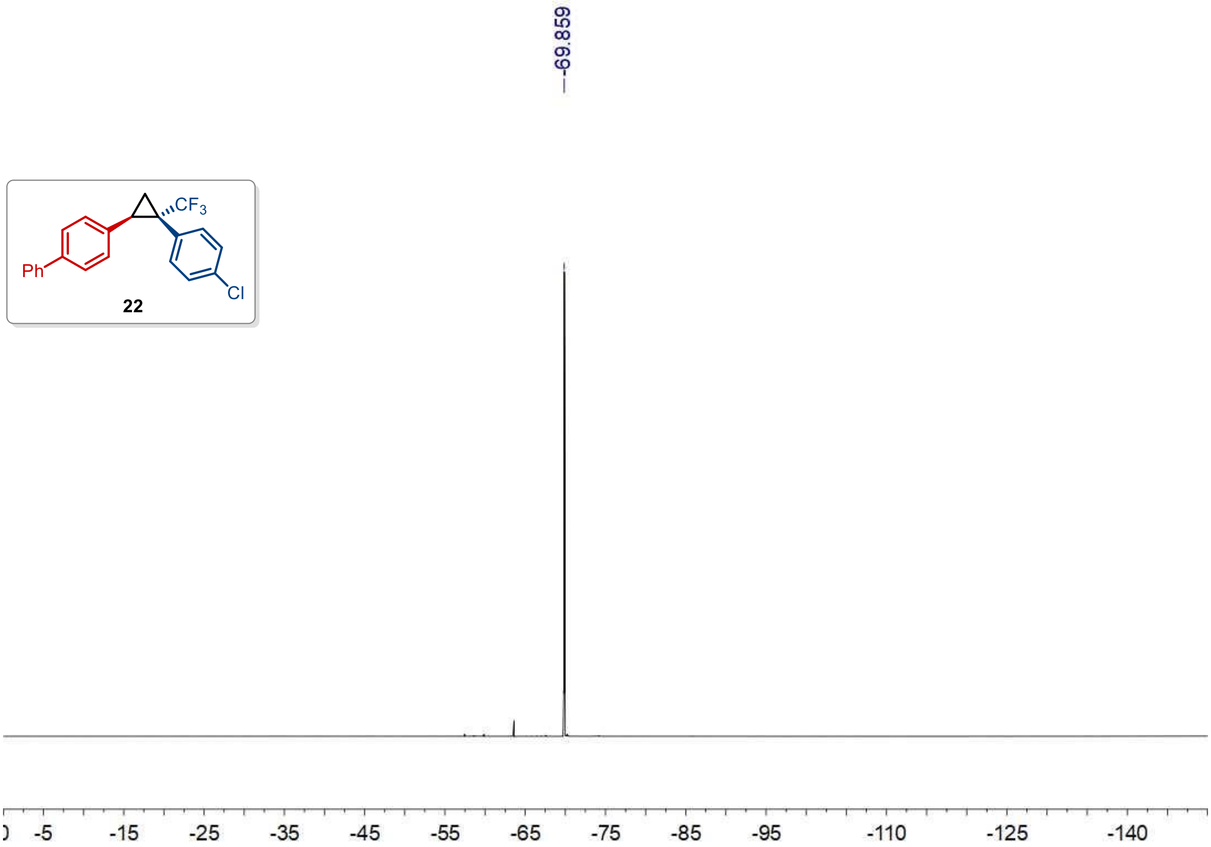

Supplementary Fig. 62 ^{19}**F NMR** (564 MHz, $CDCl_3$) spectrum of compound **22**.

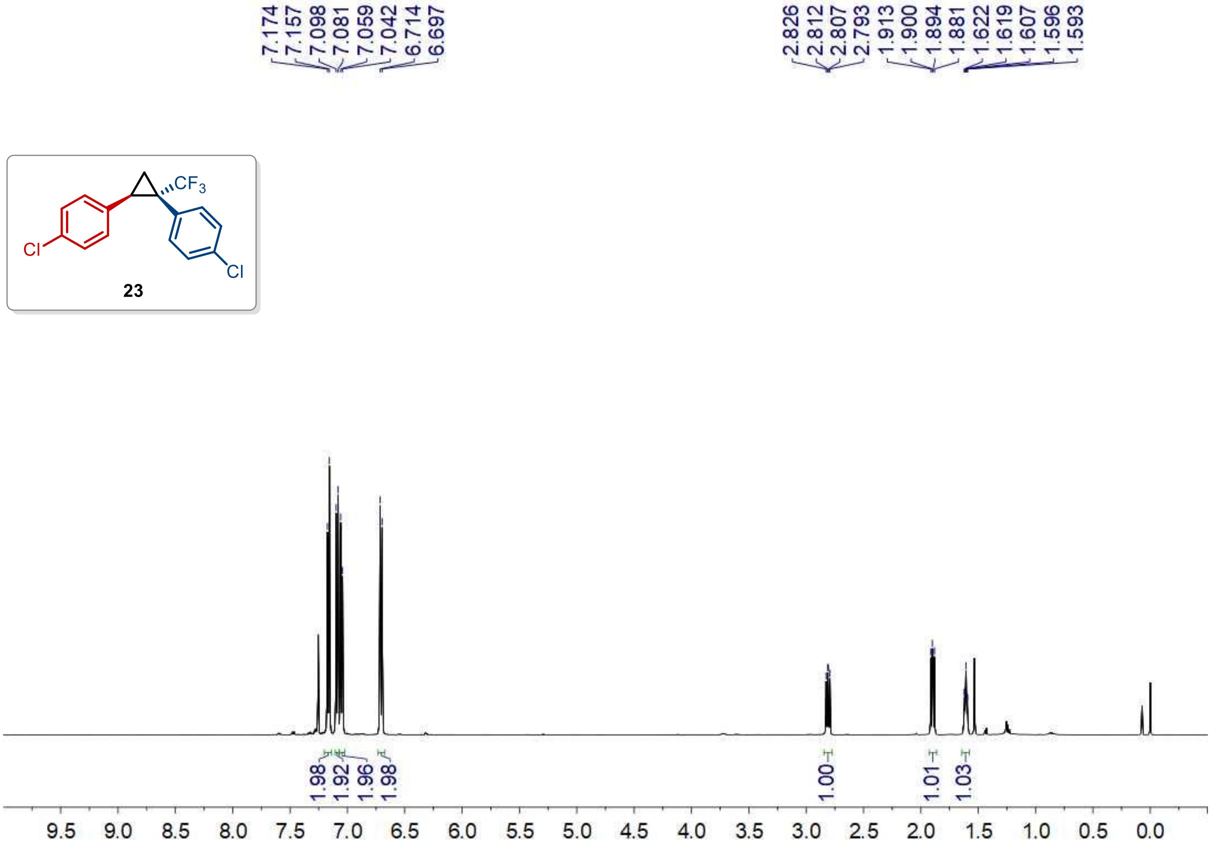

Supplementary Fig. 63 ^{1}**H NMR** (500 MHz, $CDCl_3$) spectrum of compound **23**.

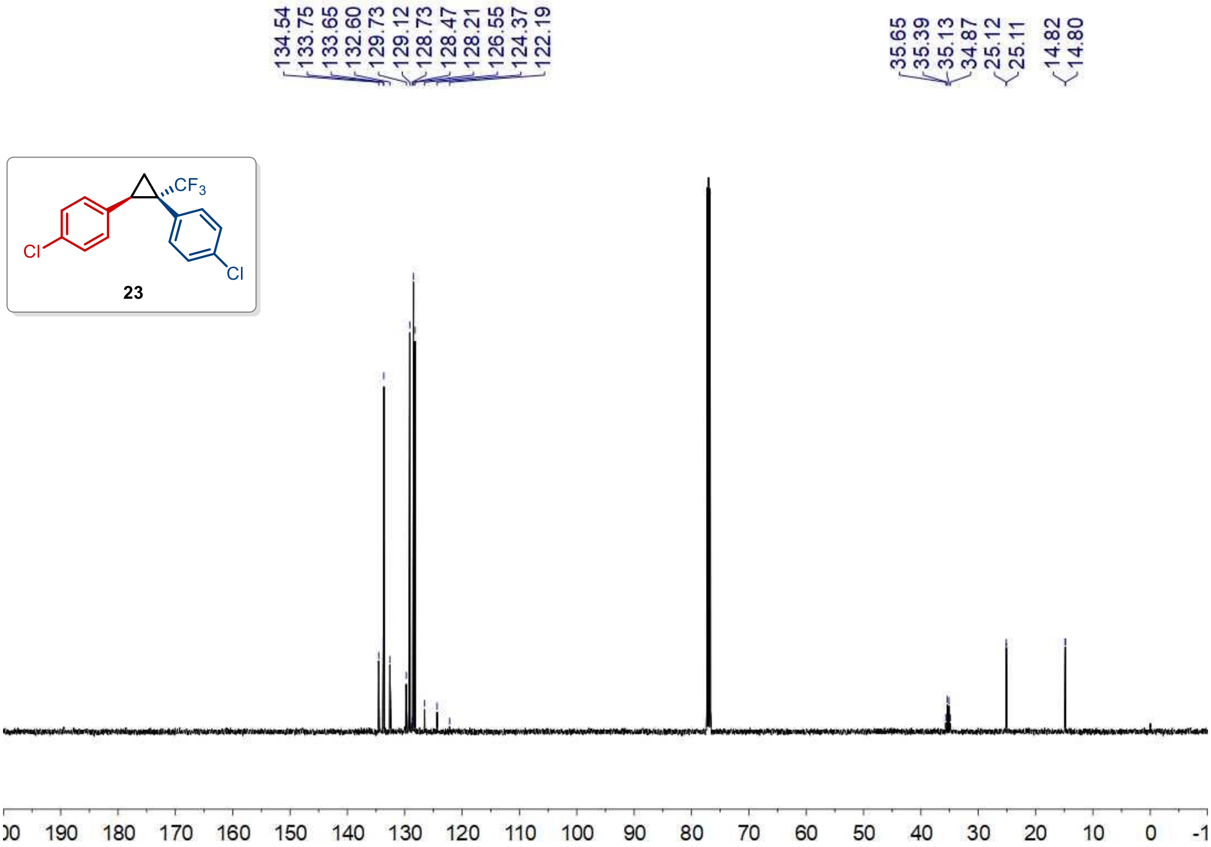

Supplementary Fig. 64 ^{13}**C NMR** (125 MHz, $CDCl_3$) spectrum of compound **23**.

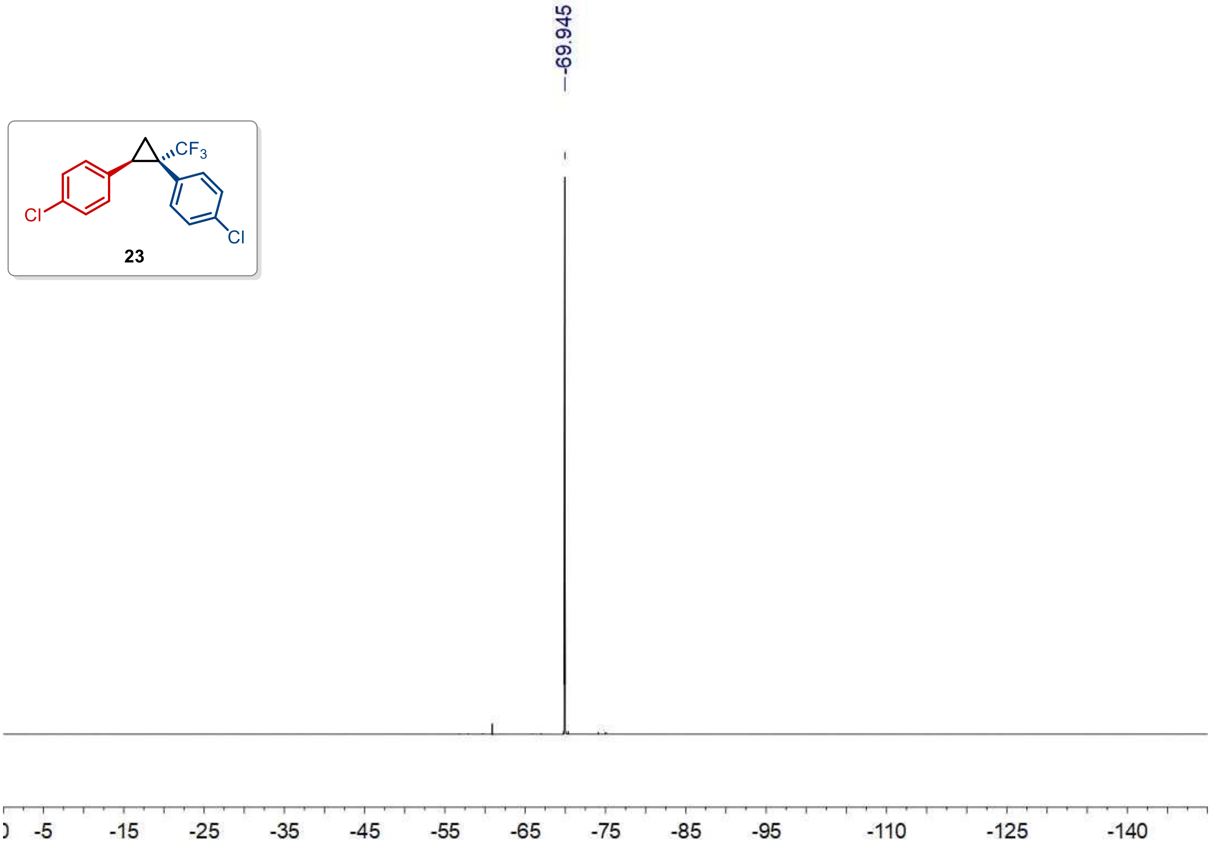

Supplementary Fig. 65 ^{19}F NMR (470 MHz, $CDCl_3$) spectrum of compound **23**.

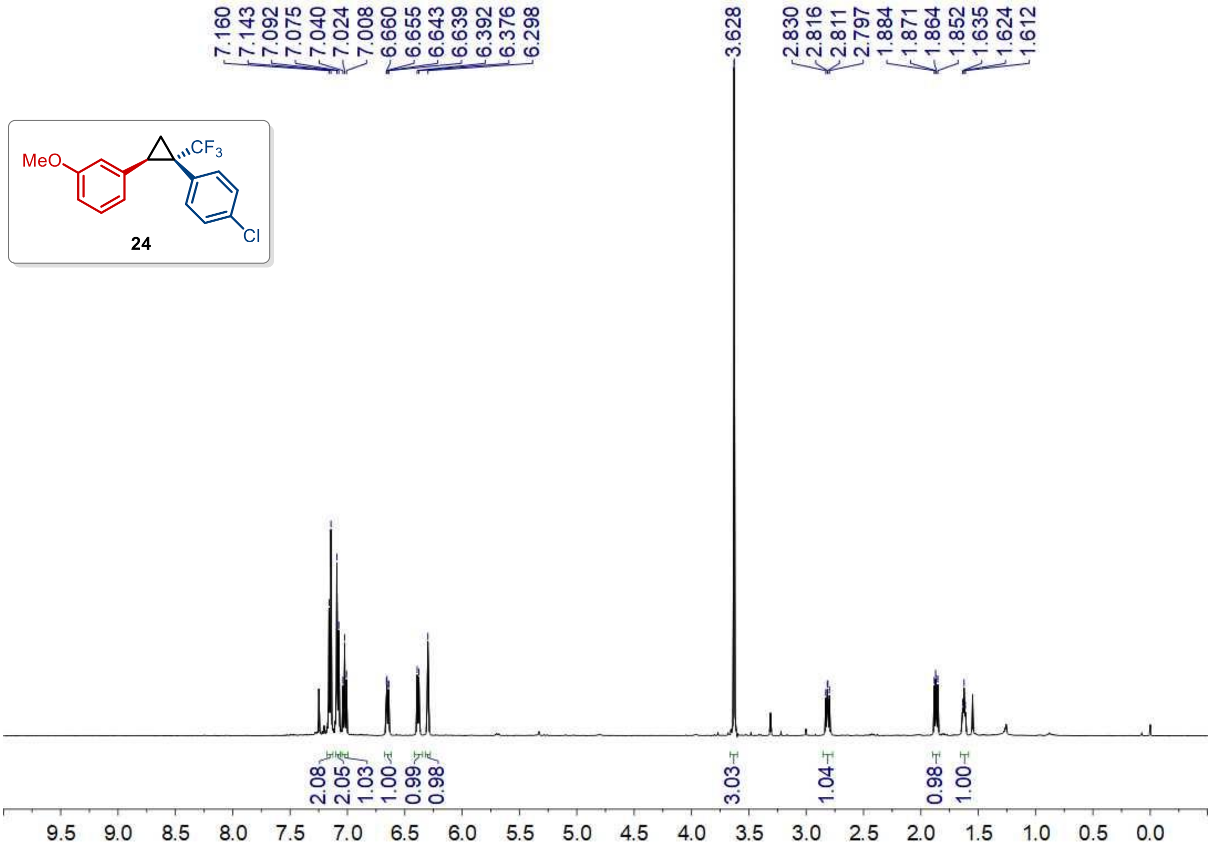

Supplementary Fig. 66 ^{1}H NMR (500 MHz, $CDCl_3$) spectrum of compound **24**.

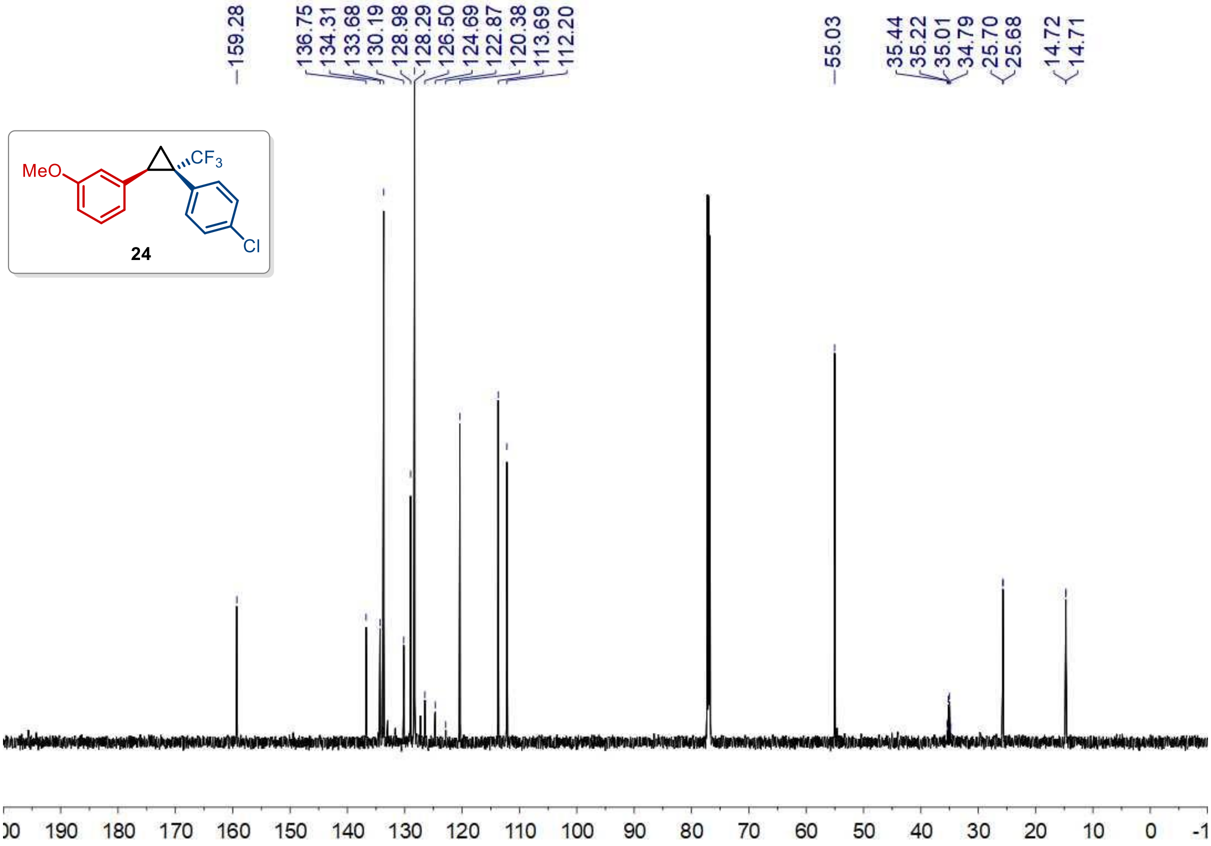

Supplementary Fig. 67 ^{13}**C NMR** (150 MHz, $CDCl_3$) spectrum of compound **24**.

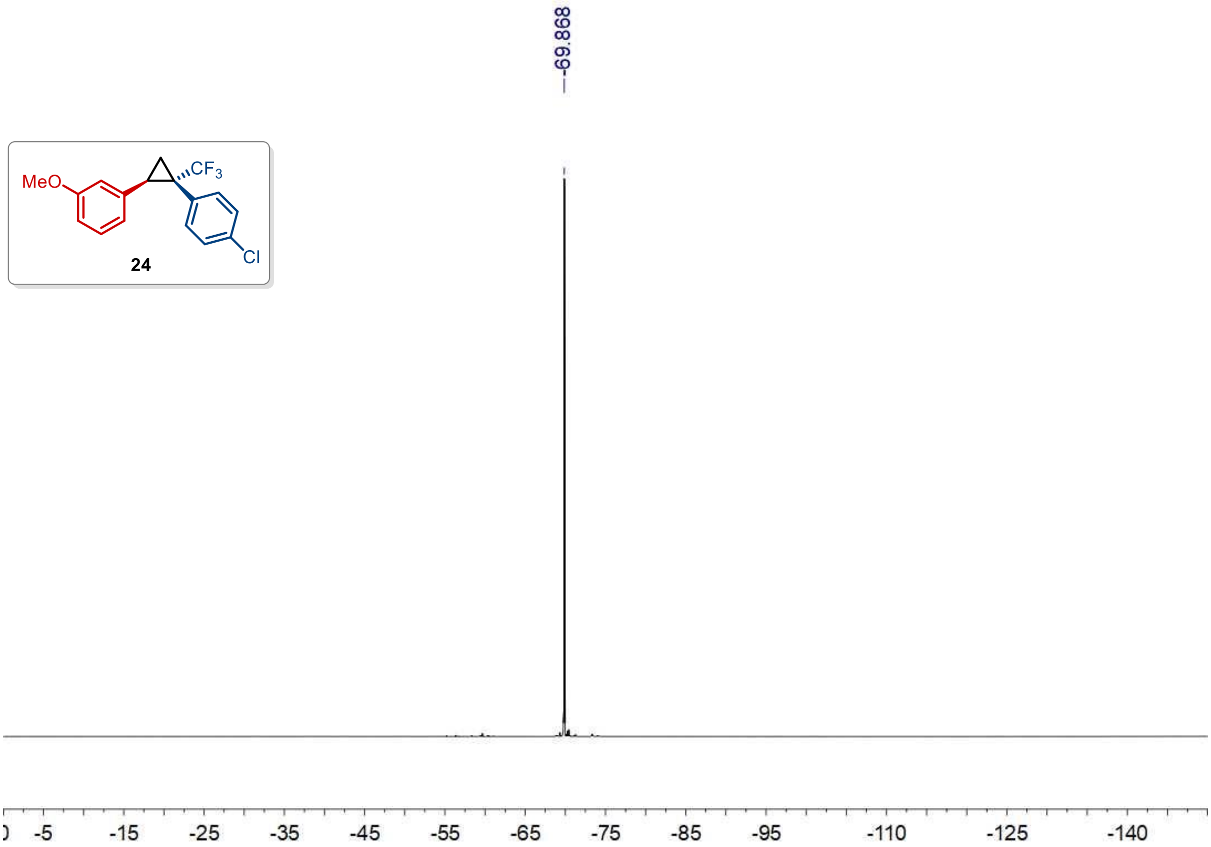

Supplementary Fig. 68 ^{19}**F NMR** (564 MHz, $CDCl_3$) spectrum of compound **24**.

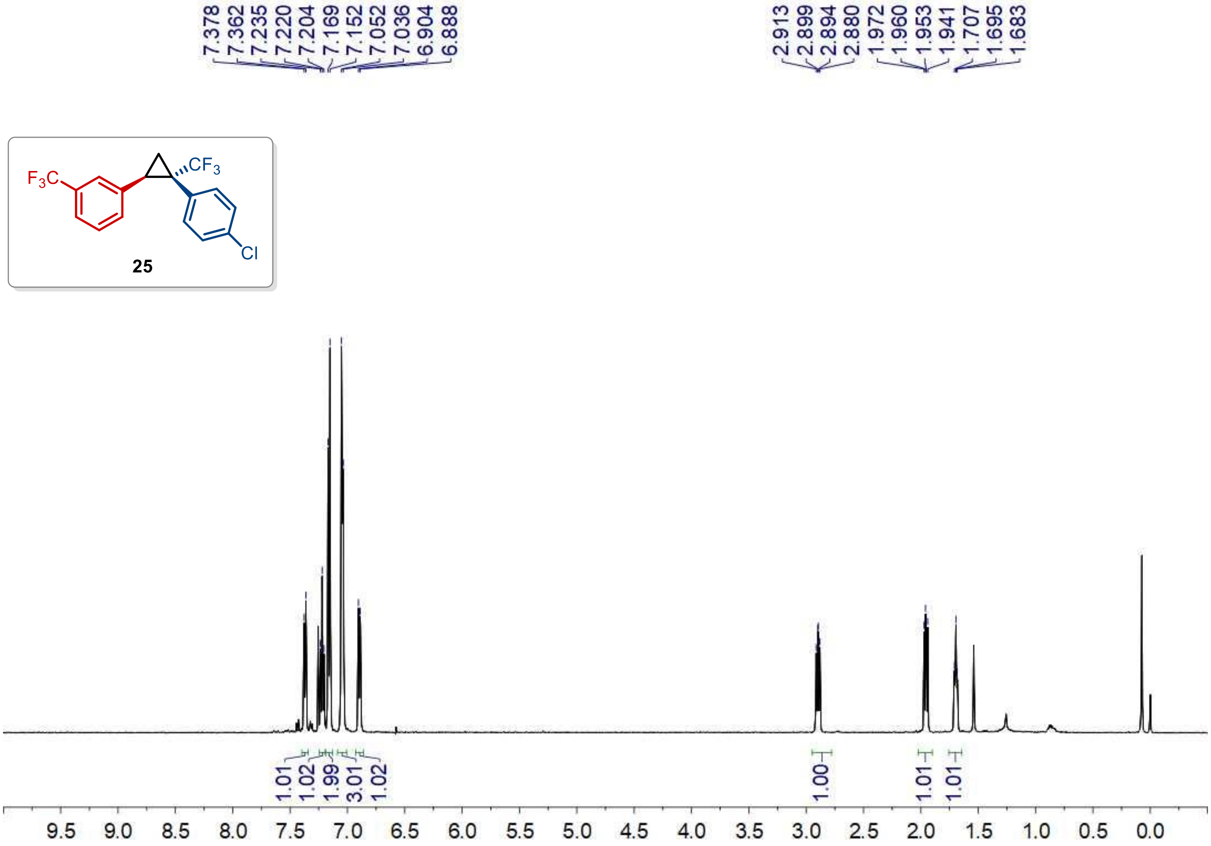

Supplementary Fig. 69 ^{1}**H NMR** (500 MHz, $CDCl_3$) spectrum of compound **25**.

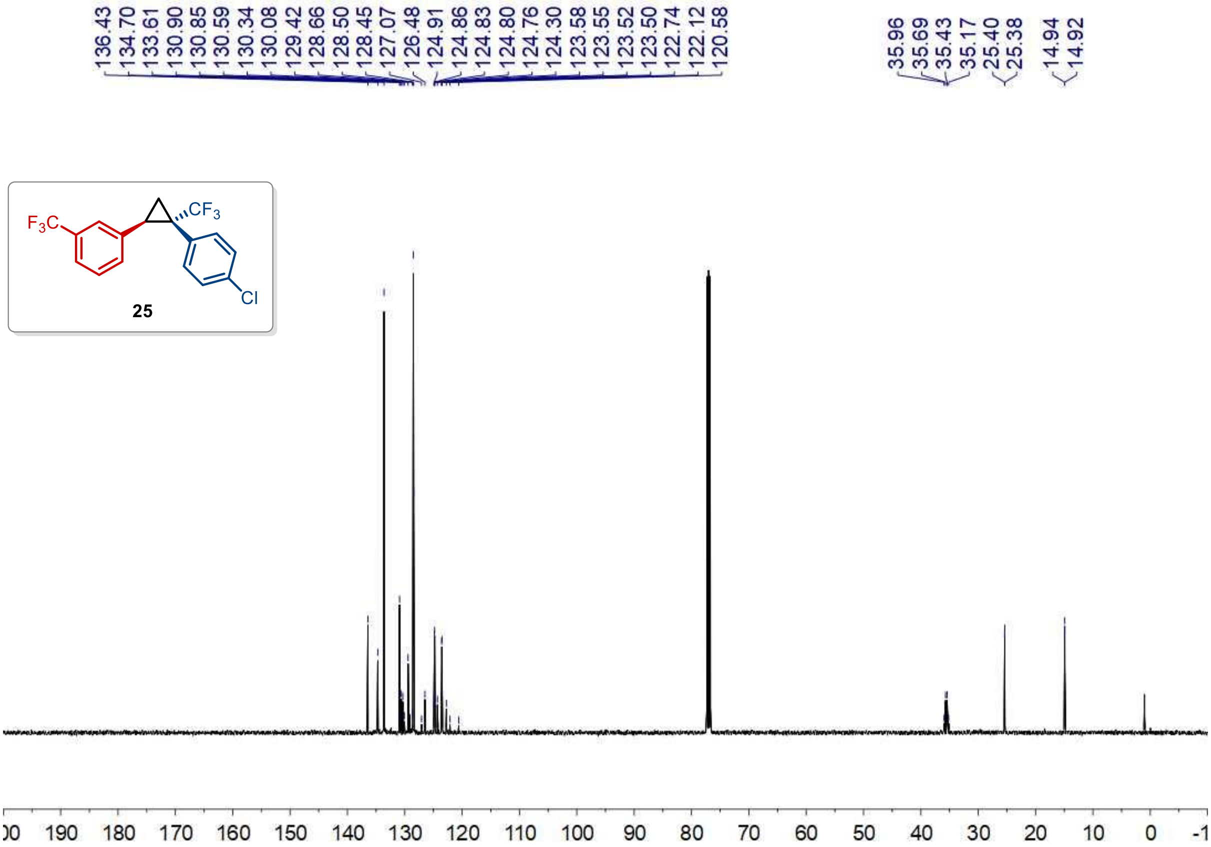

Supplementary Fig. 70 ^{13}**C NMR** (125 MHz, $CDCl_3$) spectrum of compound **25**.

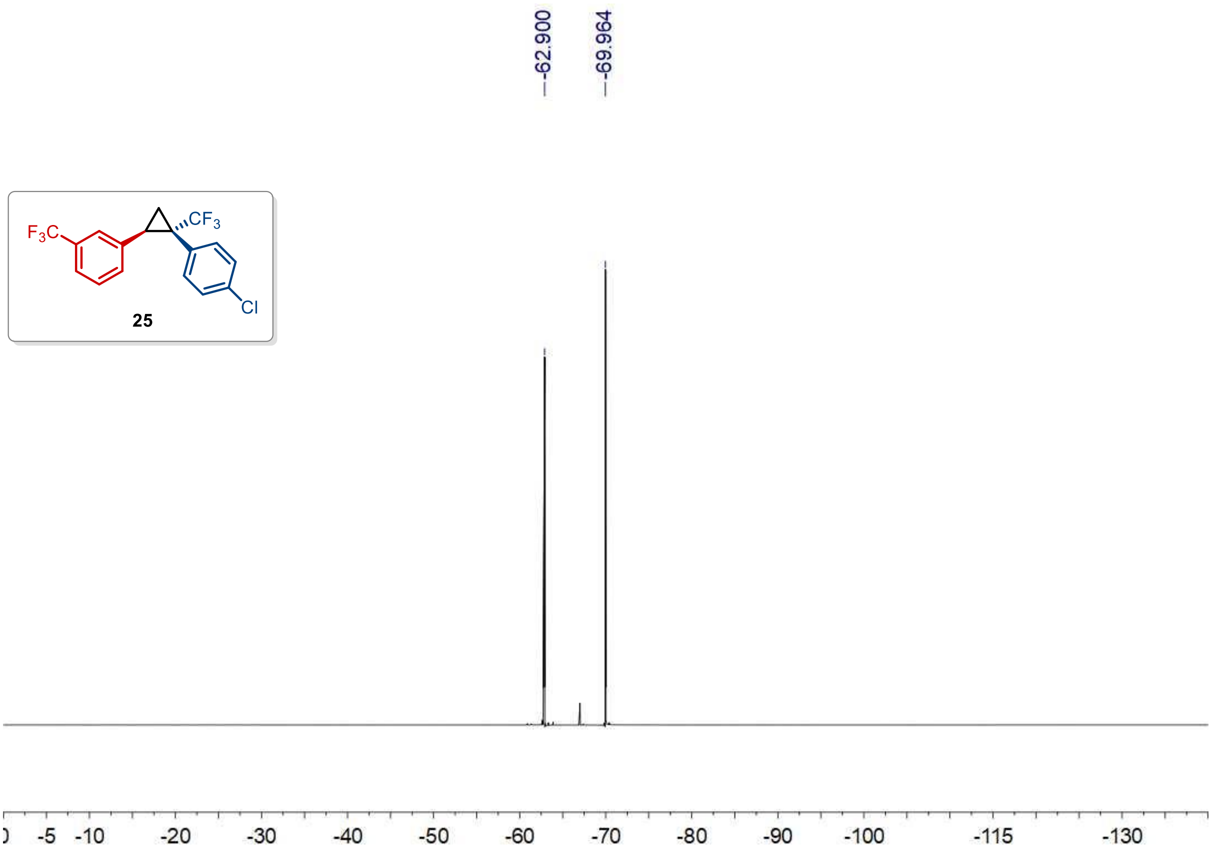

Supplementary Fig. 71 ^{19}**F NMR** (470 MHz, $CDCl_3$) spectrum of compound **25**.

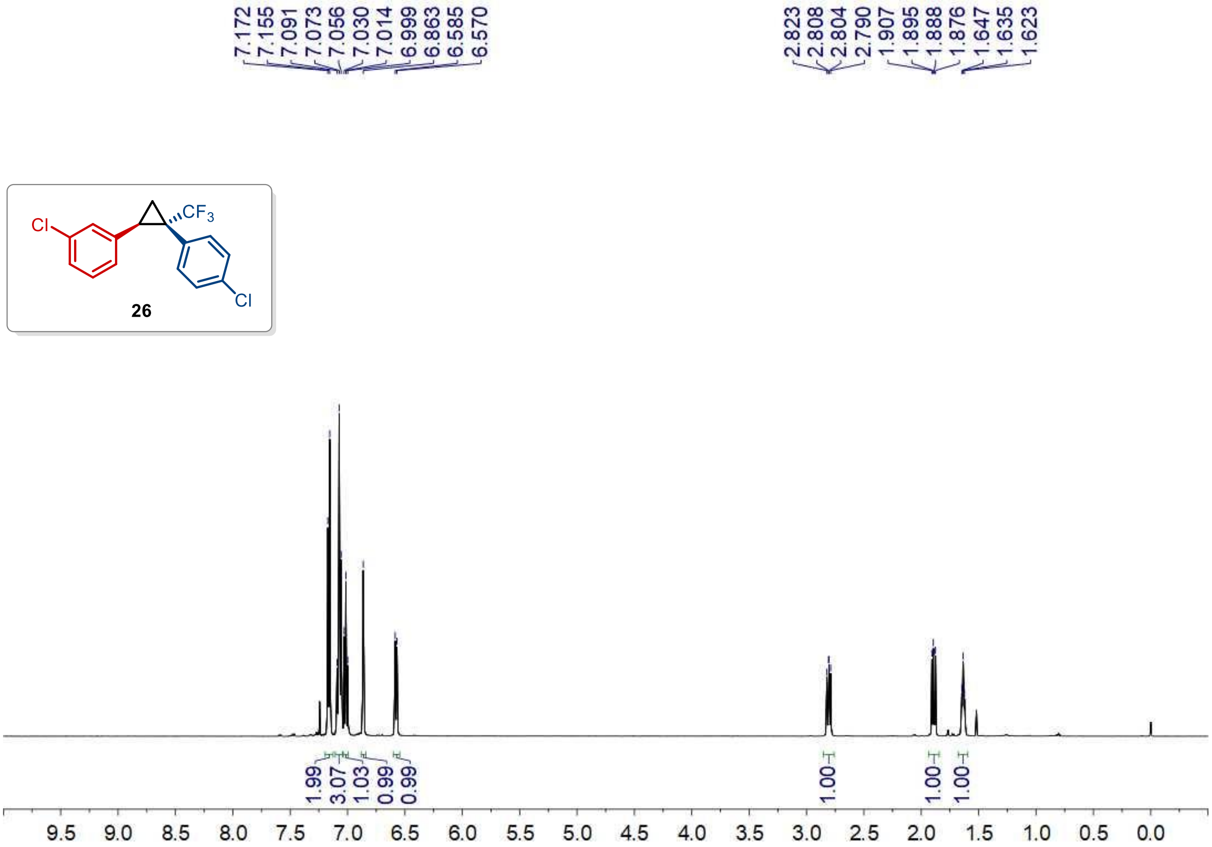

Supplementary Fig. 72 ^{1}**H NMR** (500 MHz, $CDCl_3$) spectrum of compound **26**.

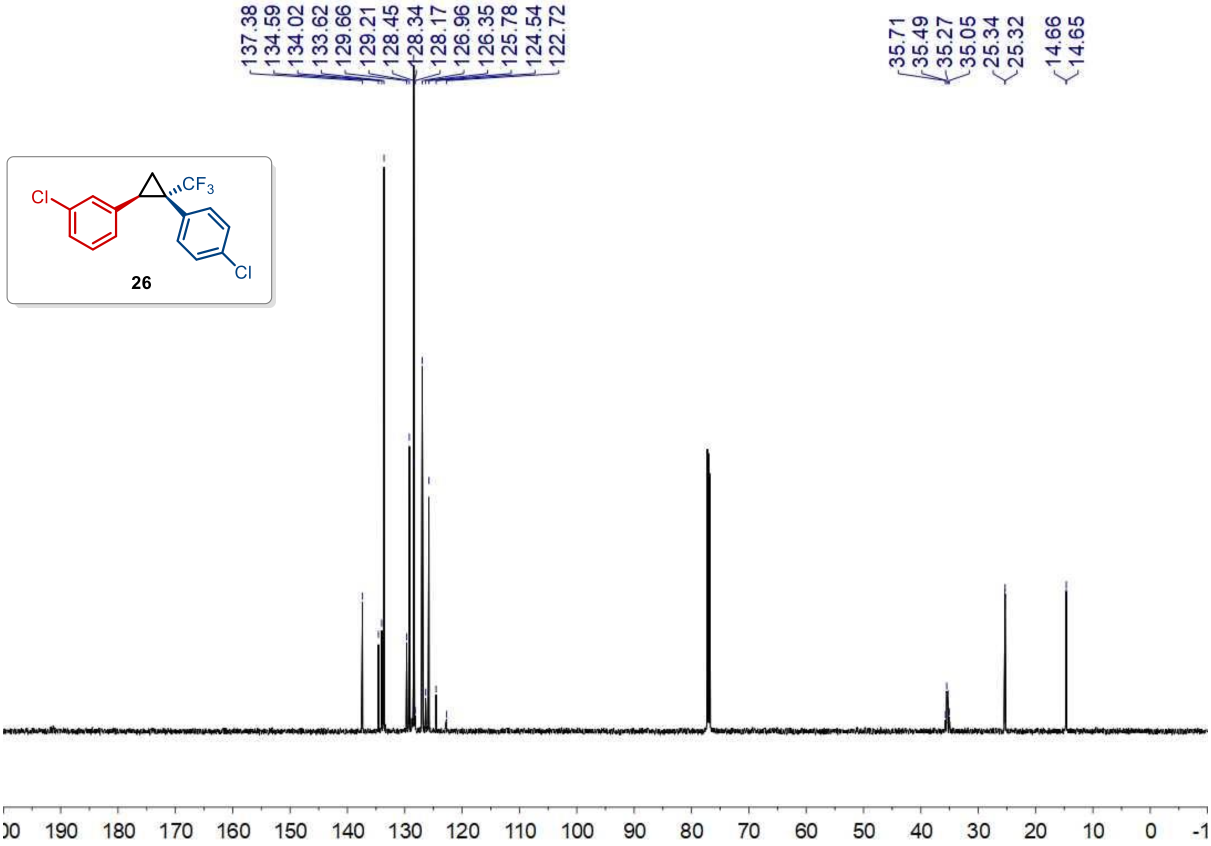

Supplementary Fig. 73 ^{13}**C NMR** (150 MHz, $CDCl_3$) spectrum of compound **26**.

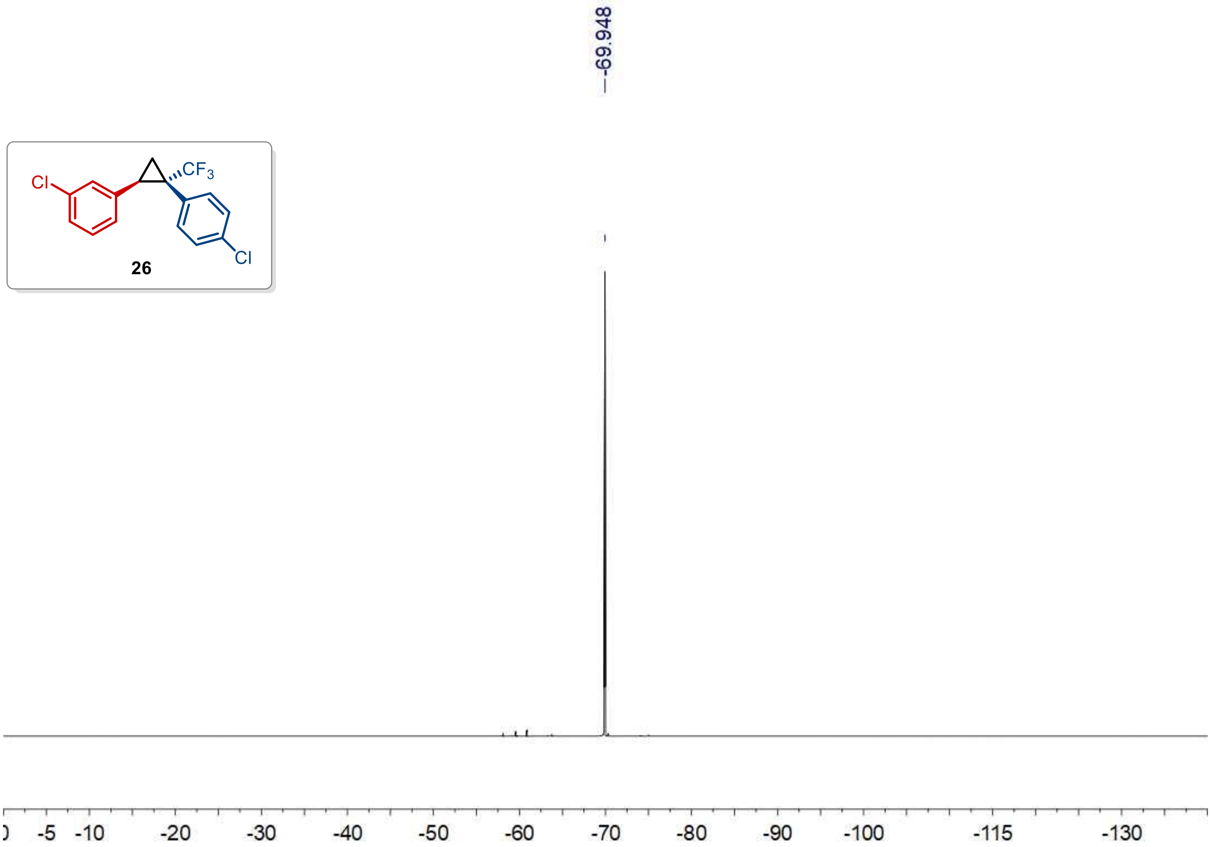

Supplementary Fig. 74 ^{19}**F NMR** (564 MHz, $CDCl_3$) spectrum of compound **26**.

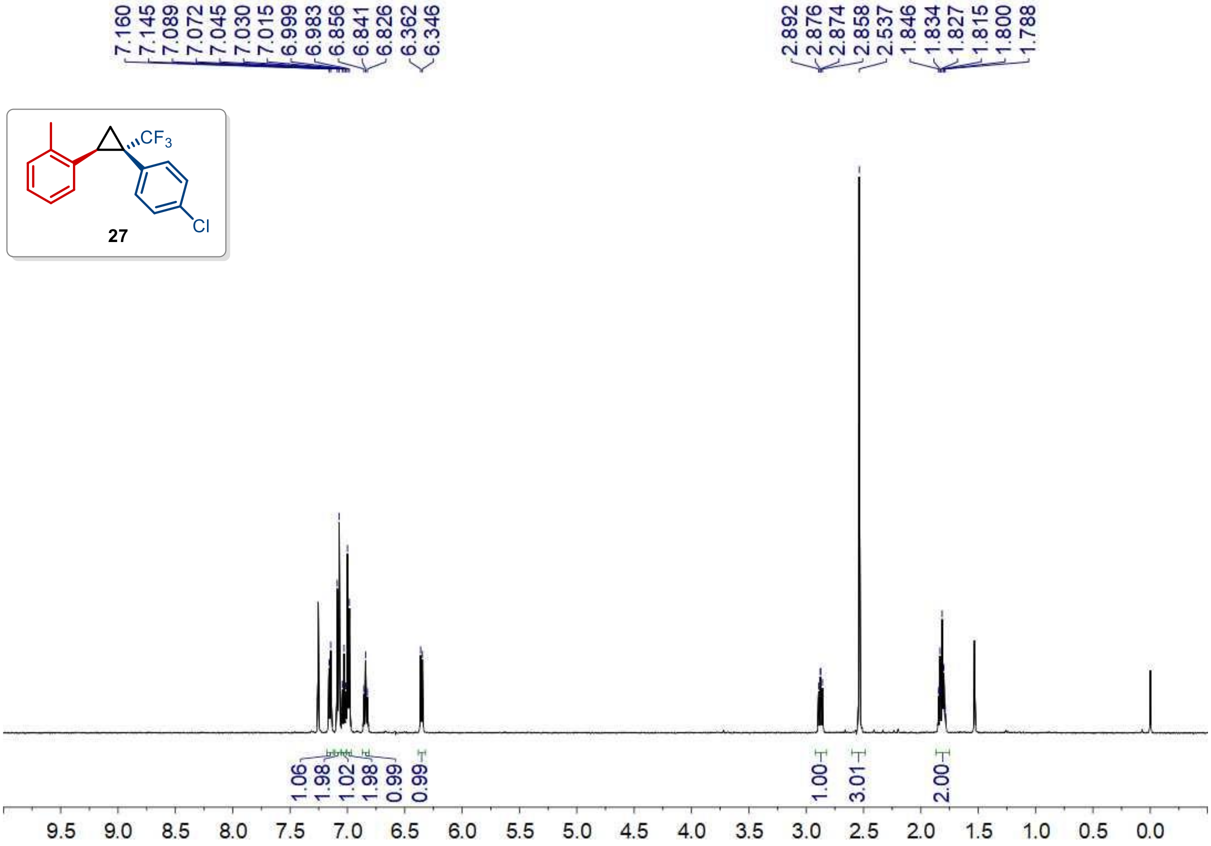

Supplementary Fig. 75 ^{1}H NMR (500 MHz, $CDCl_3$) spectrum of compound **27**.

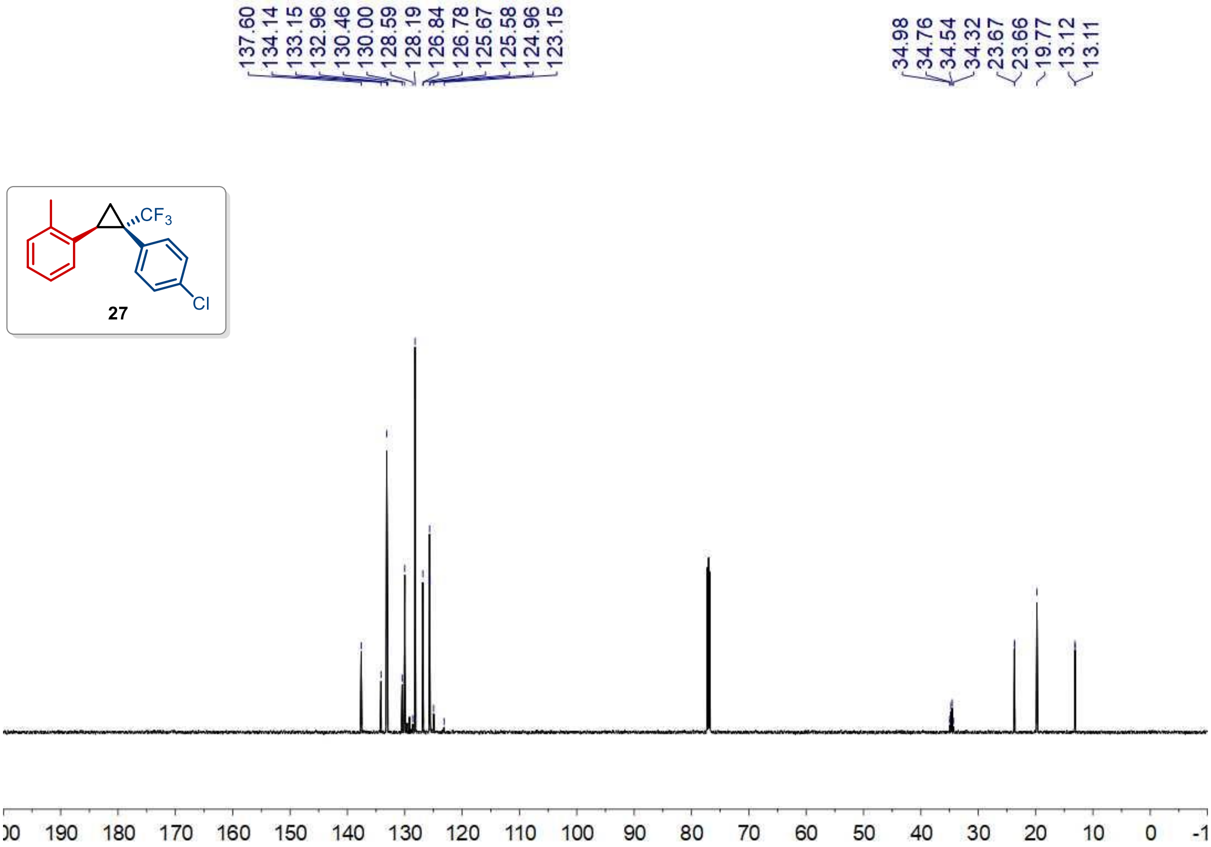

Supplementary Fig.S76 ^{13}C NMR (150 MHz, $CDCl_3$) spectrum of compound **27**.

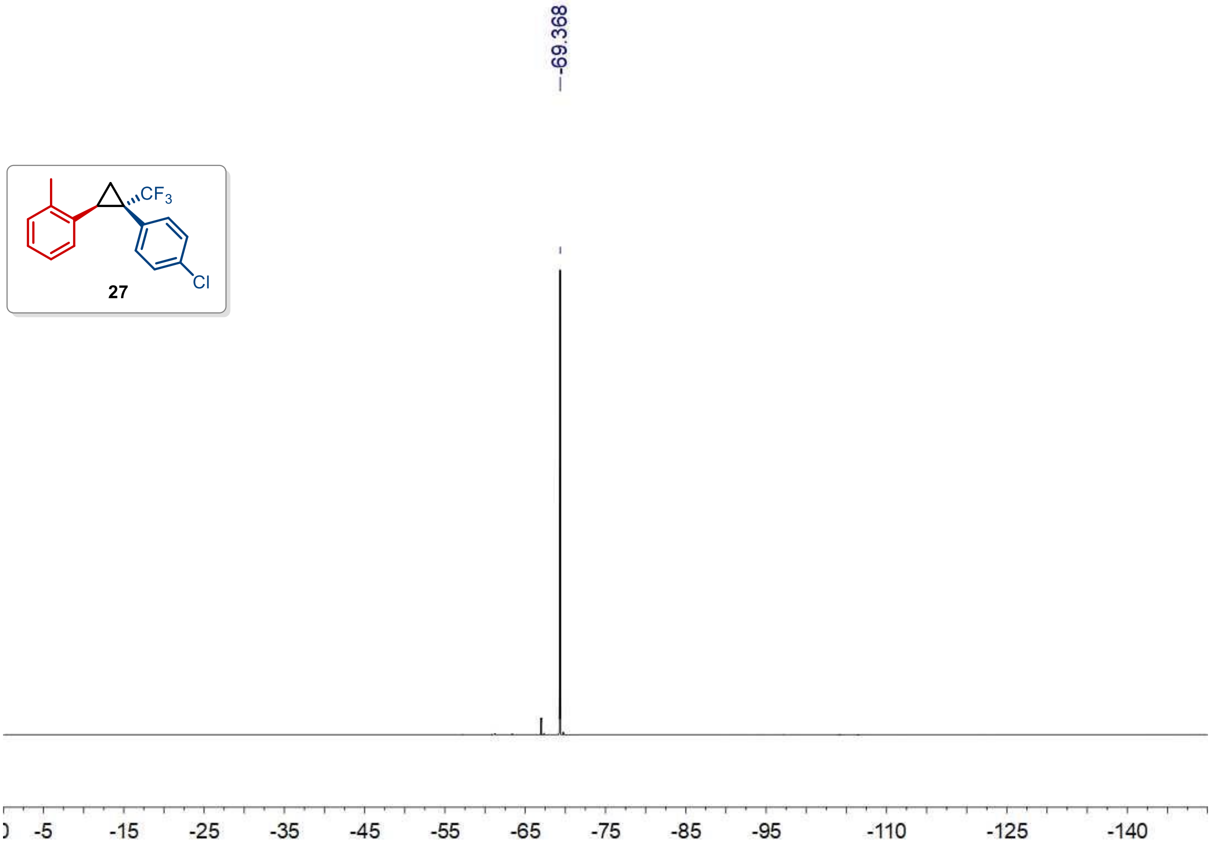

Supplementary Fig. 77 ^{19}F NMR (564 MHz, $CDCl_3$) spectrum of compound **27**.

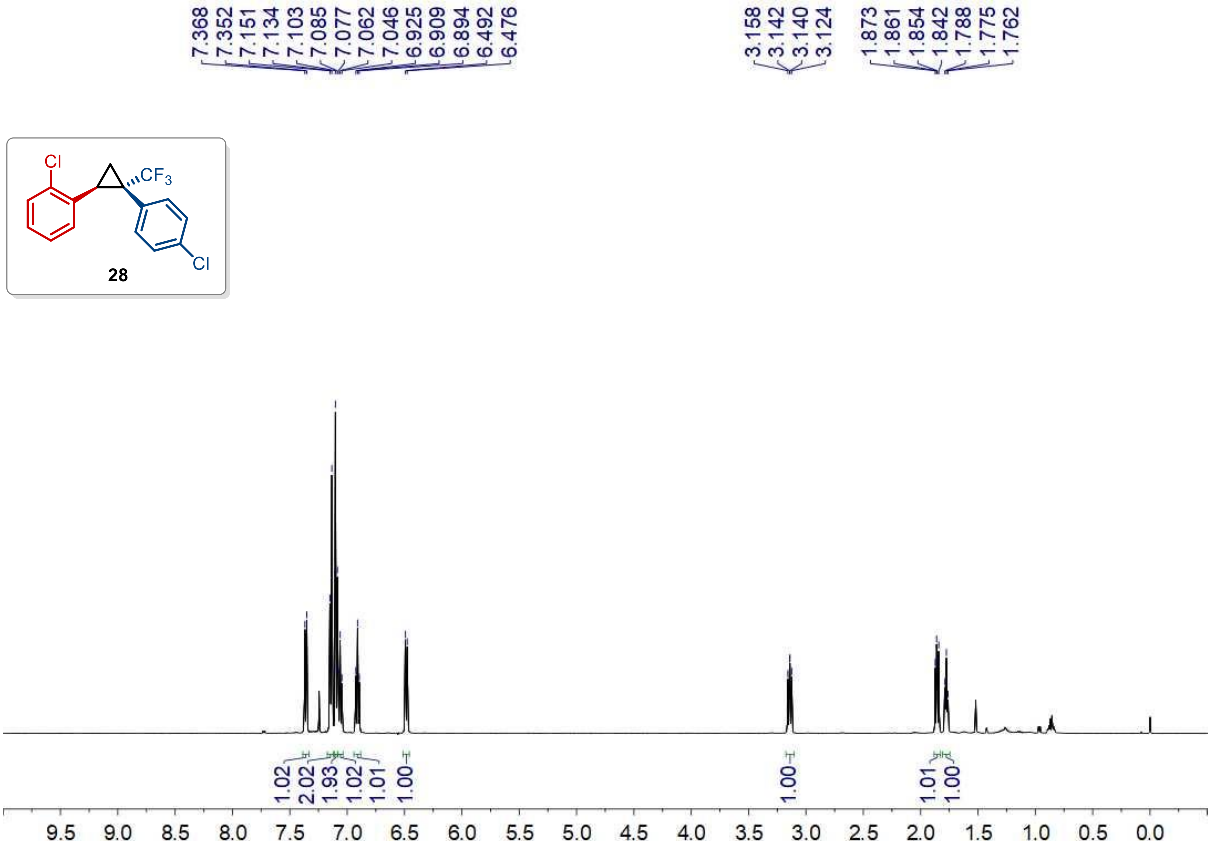

Supplementary Fig. 78 ^{1}H NMR (500 MHz, $CDCl_3$) spectrum of compound **28**.

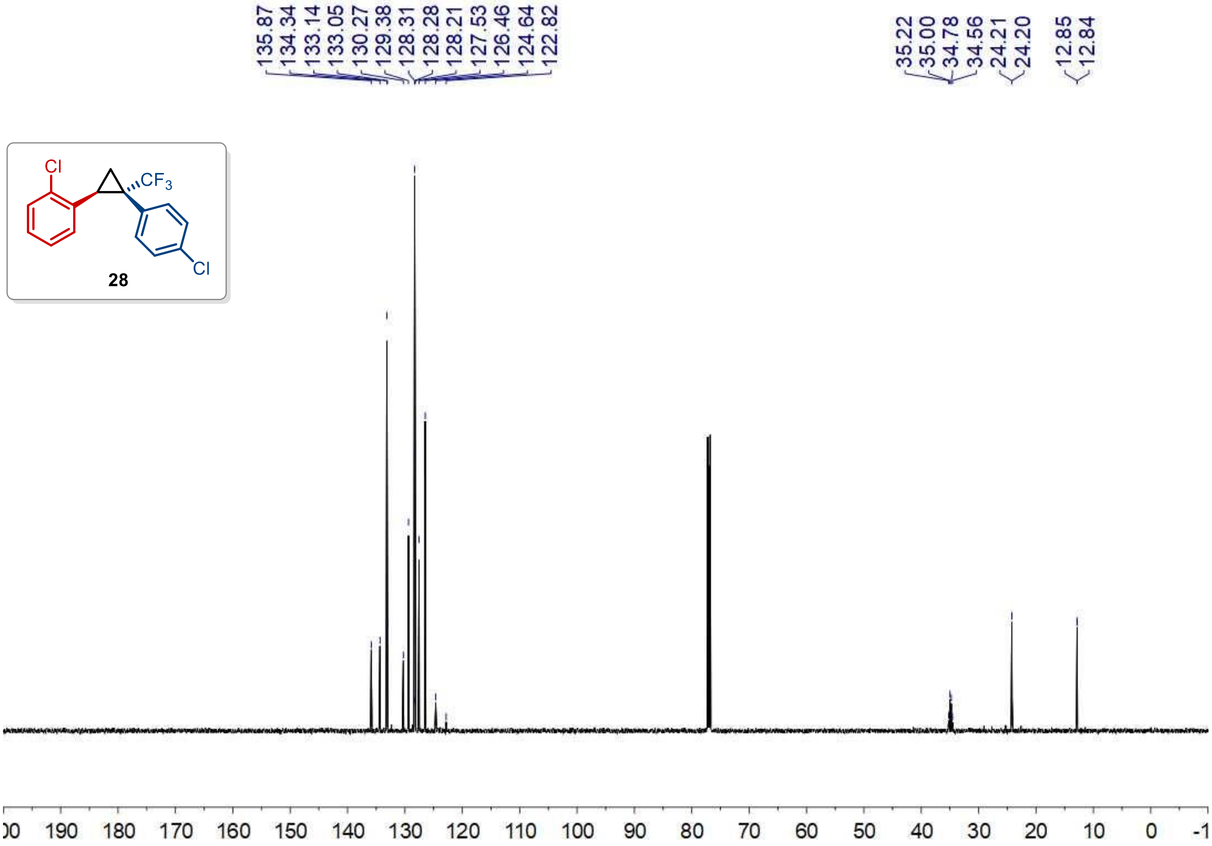

Supplementary Fig. 79 ^{13}**C NMR** (150 MHz, $CDCl_3$) spectrum of compound **28**.

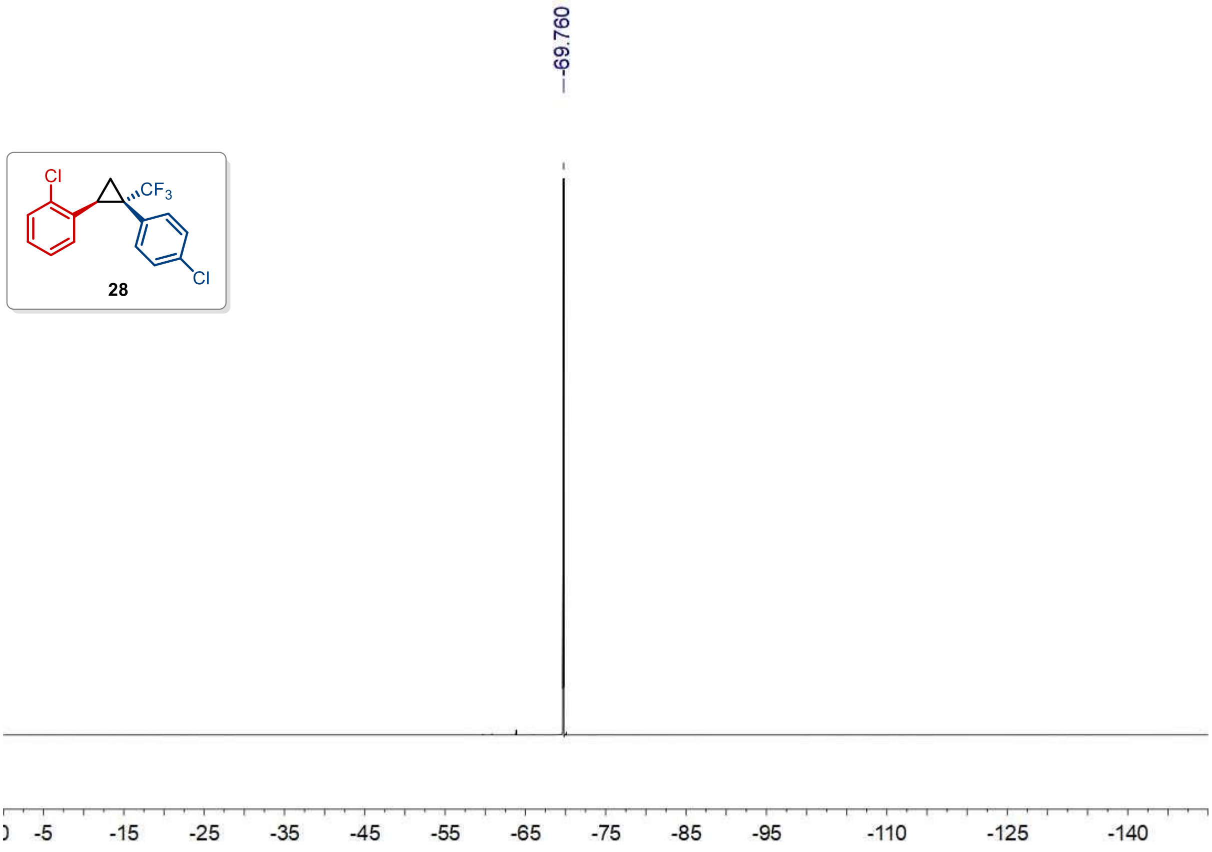

Supplementary Fig. 80 ^{19}**F NMR** (564 MHz, $CDCl_3$) spectrum of compound **28**.

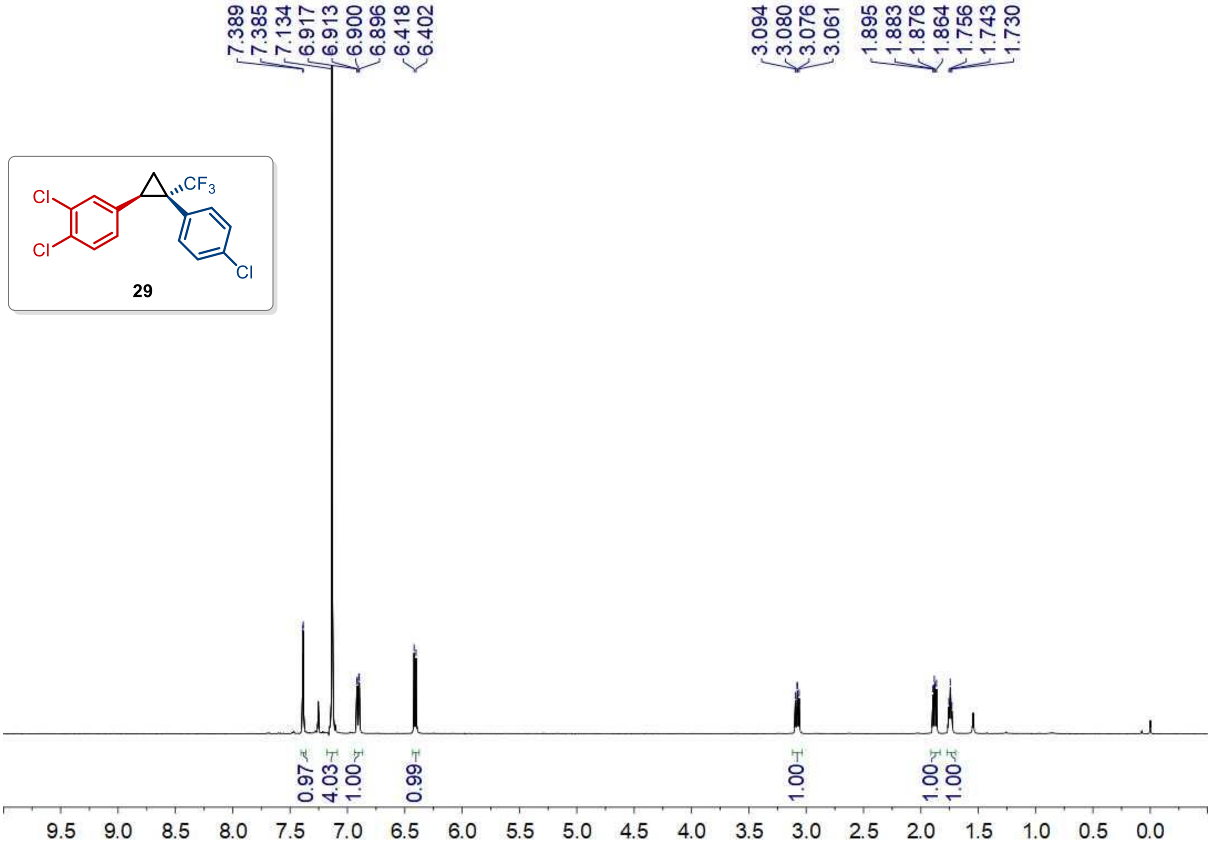

Supplementary Fig. 81 ^{1}H NMR (500 MHz, $CDCl_3$) spectrum of compound **29**.

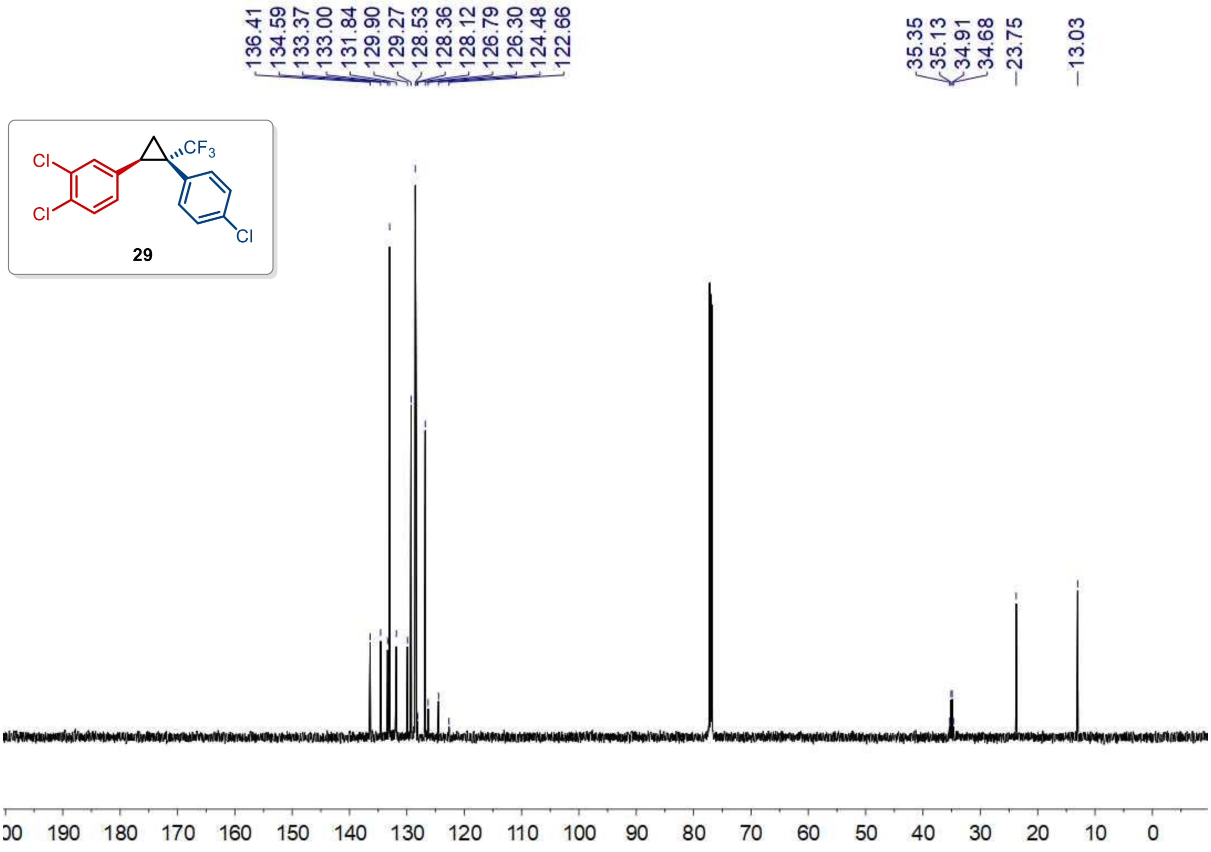

Supplementary Fig. 82 ^{13}C NMR (150 MHz, $CDCl_3$) spectrum of compound **29**.

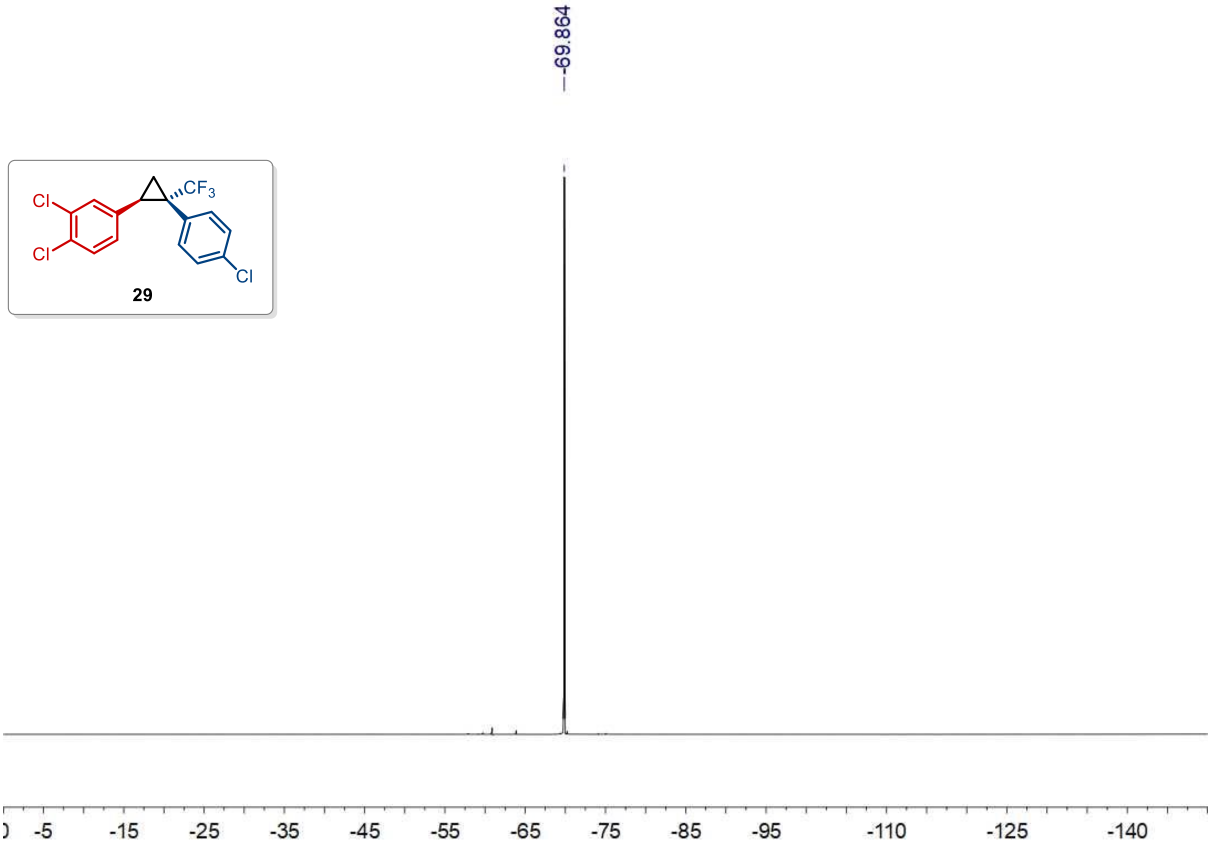

Supplementary Fig. 83 ^{19}**F NMR** (564 MHz, $CDCl_3$) spectrum of compound **29**.

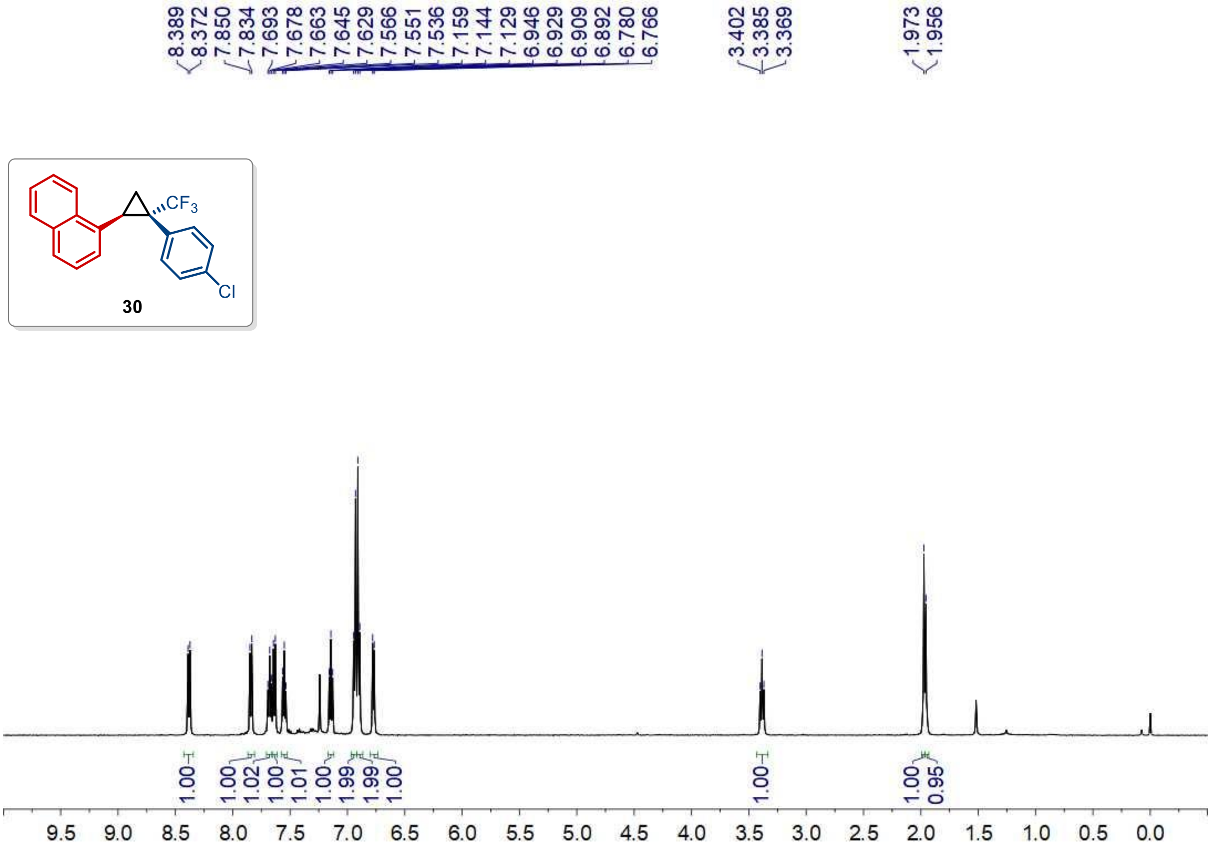

Supplementary Fig. 84 ^{1}**H NMR** (500 MHz, $CDCl_3$) spectrum of compound **30**.

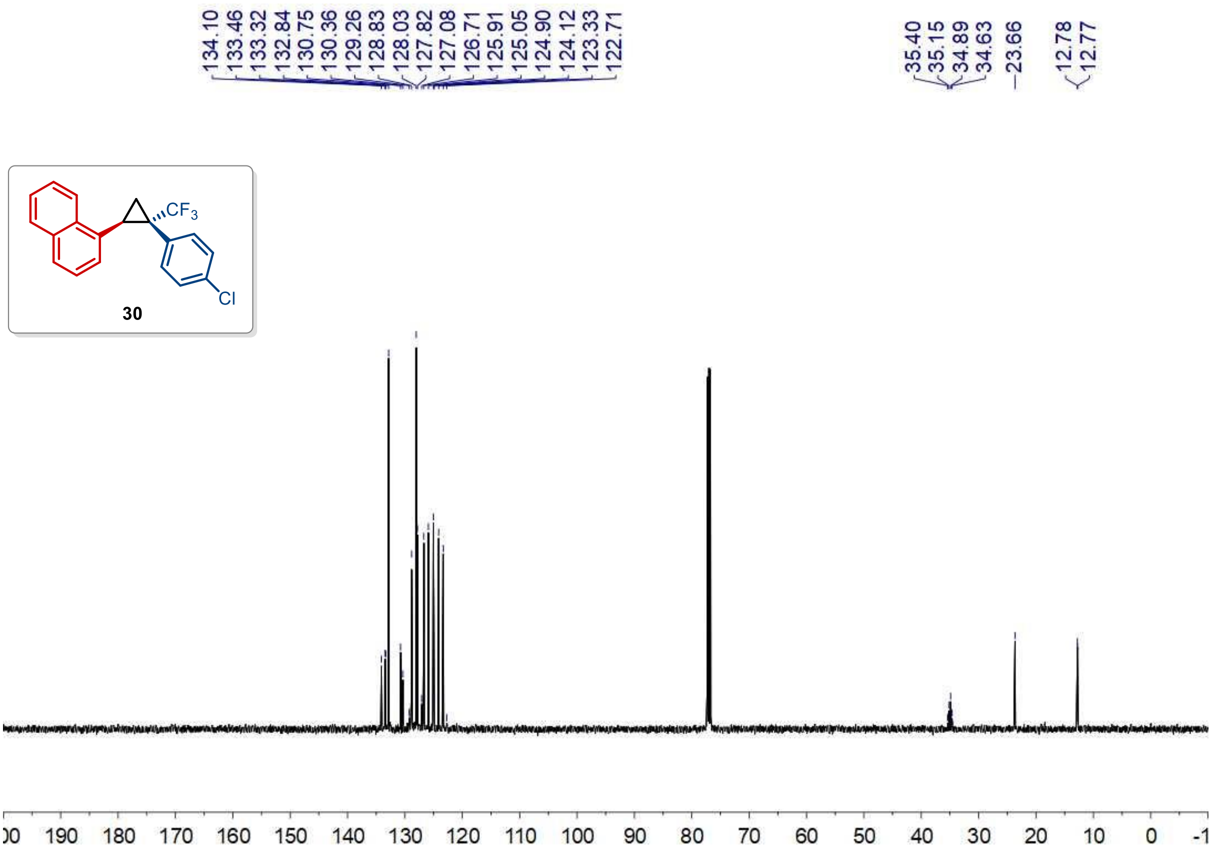

Supplementary Fig. 85 ^{13}**C NMR** (125 MHz, $CDCl_3$) spectrum of compound **30**.

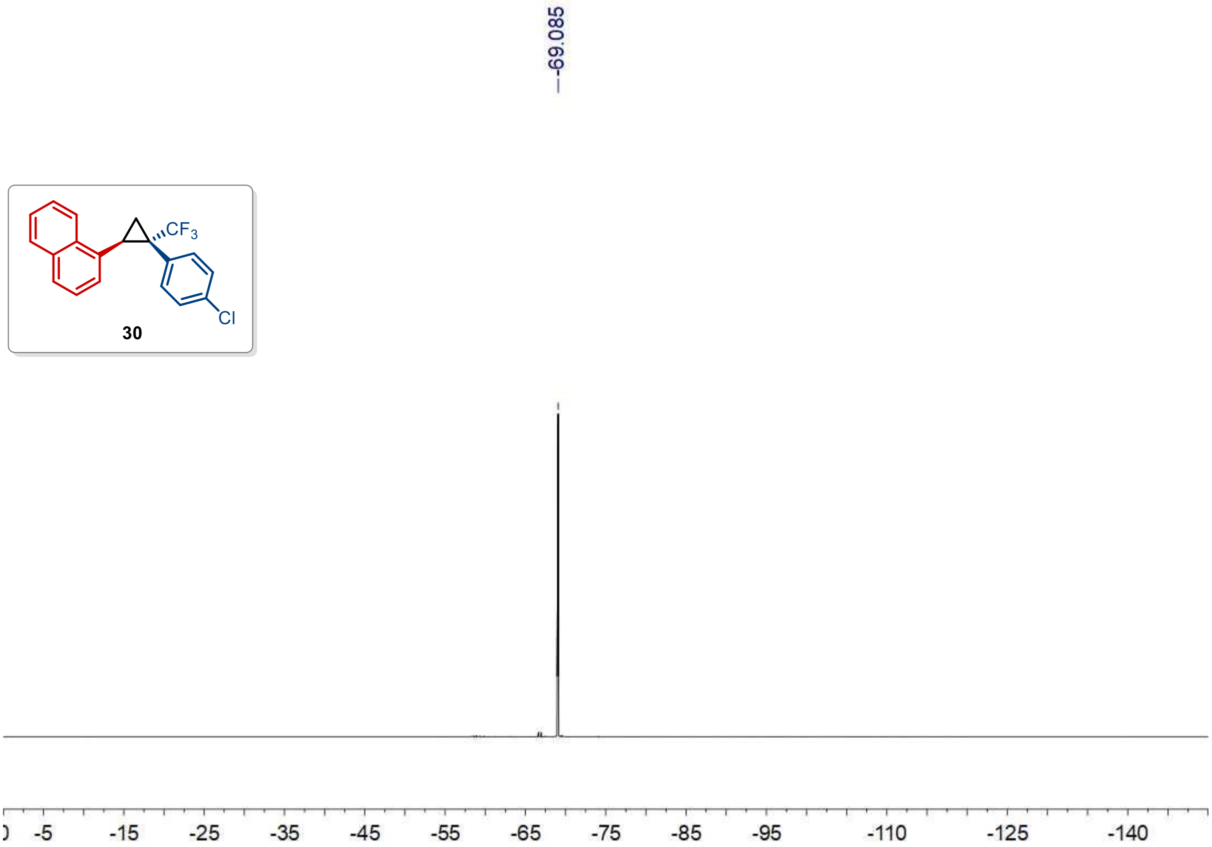

Supplementary Fig. 86 ^{19}**F NMR** (564 MHz, $CDCl_3$) spectrum of compound **30**.

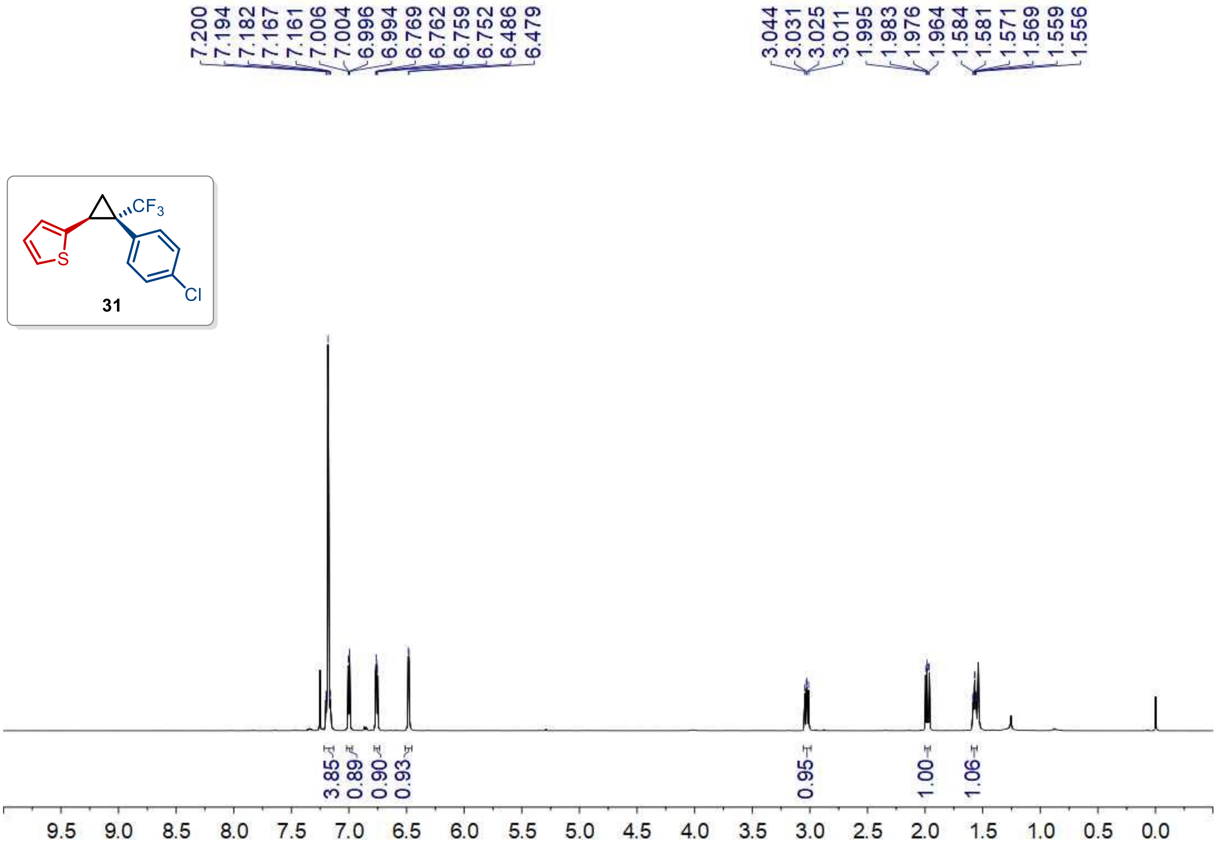

Supplementary Fig. 87 ^{1}**H NMR** (500 MHz, $CDCl_3$) spectrum of compound **31**.

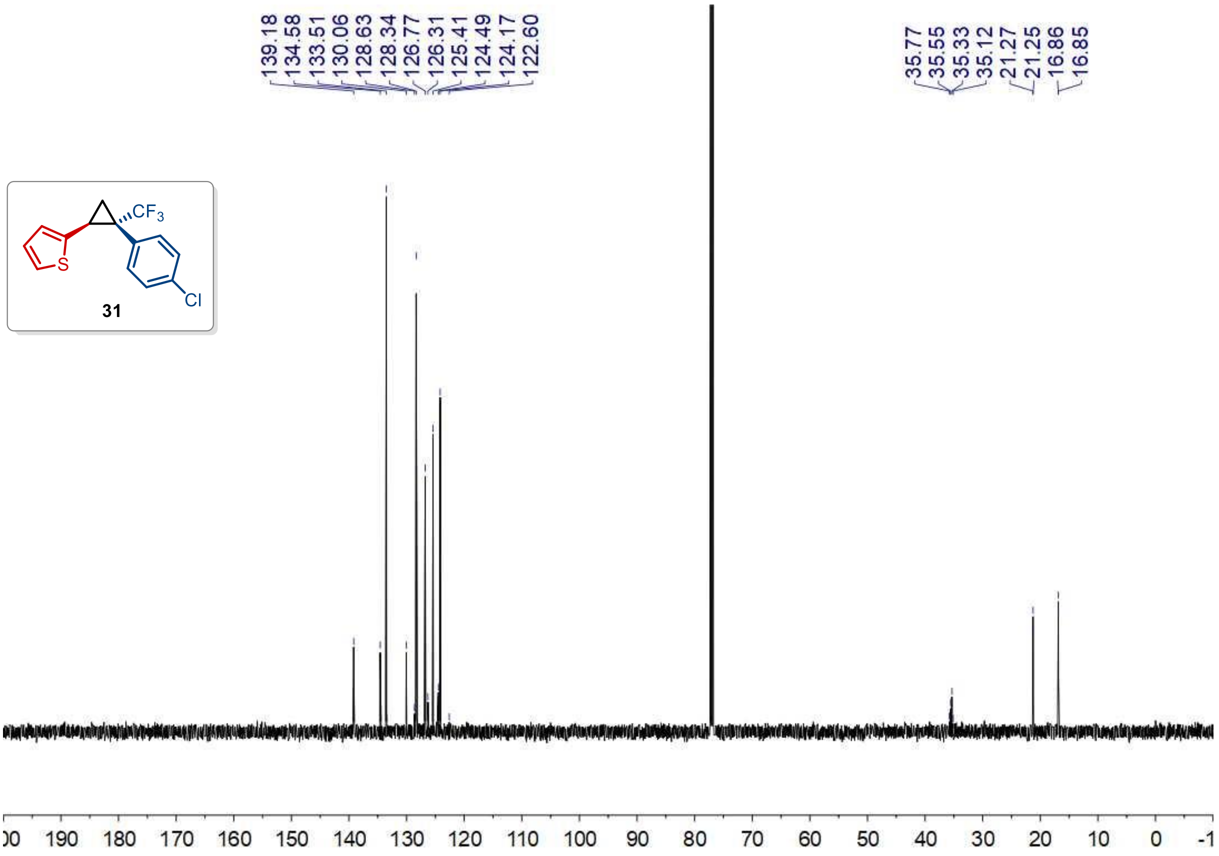

Supplementary Fig. 88 ^{13}**C NMR** (150 MHz, $CDCl_3$) spectrum of compound **31**.

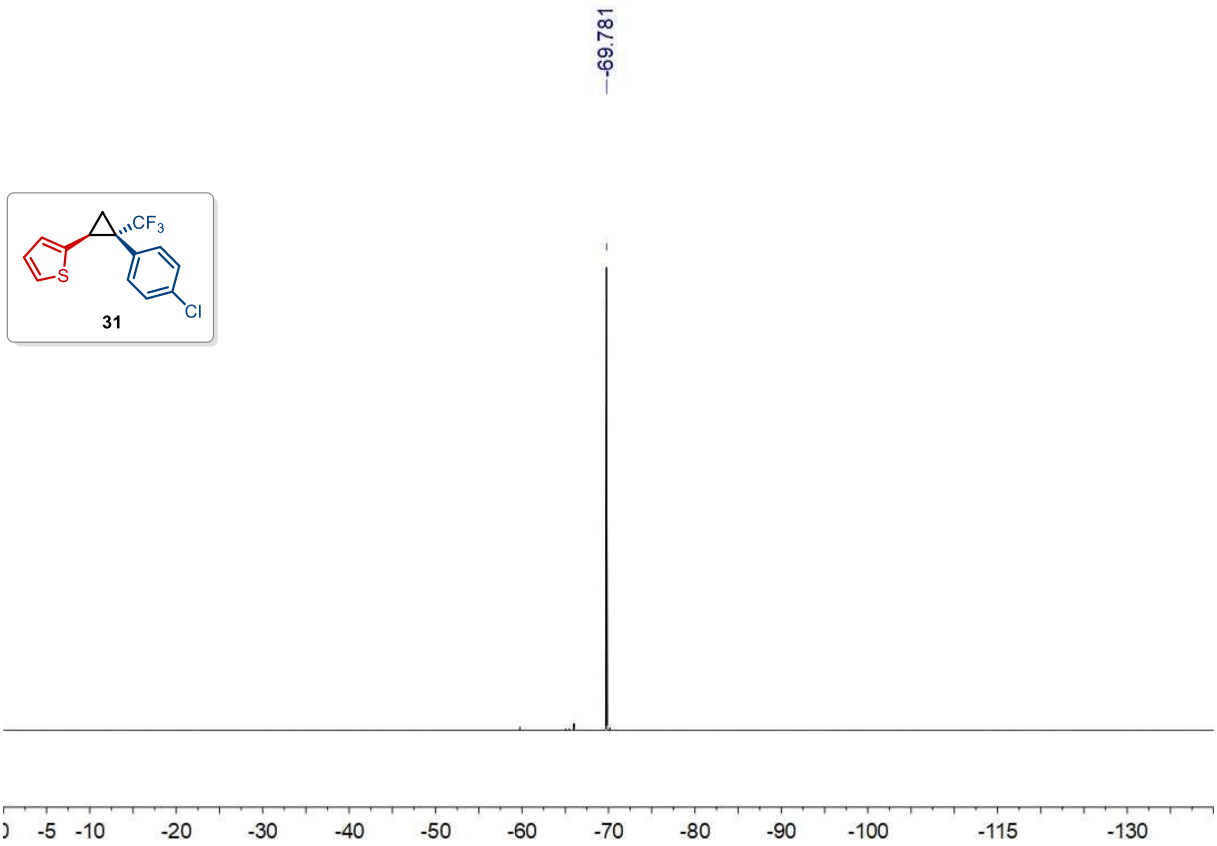

Supplementary Fig. 89 ^{19}F NMR (564 MHz, $CDCl_3$) spectrum of compound **31**.

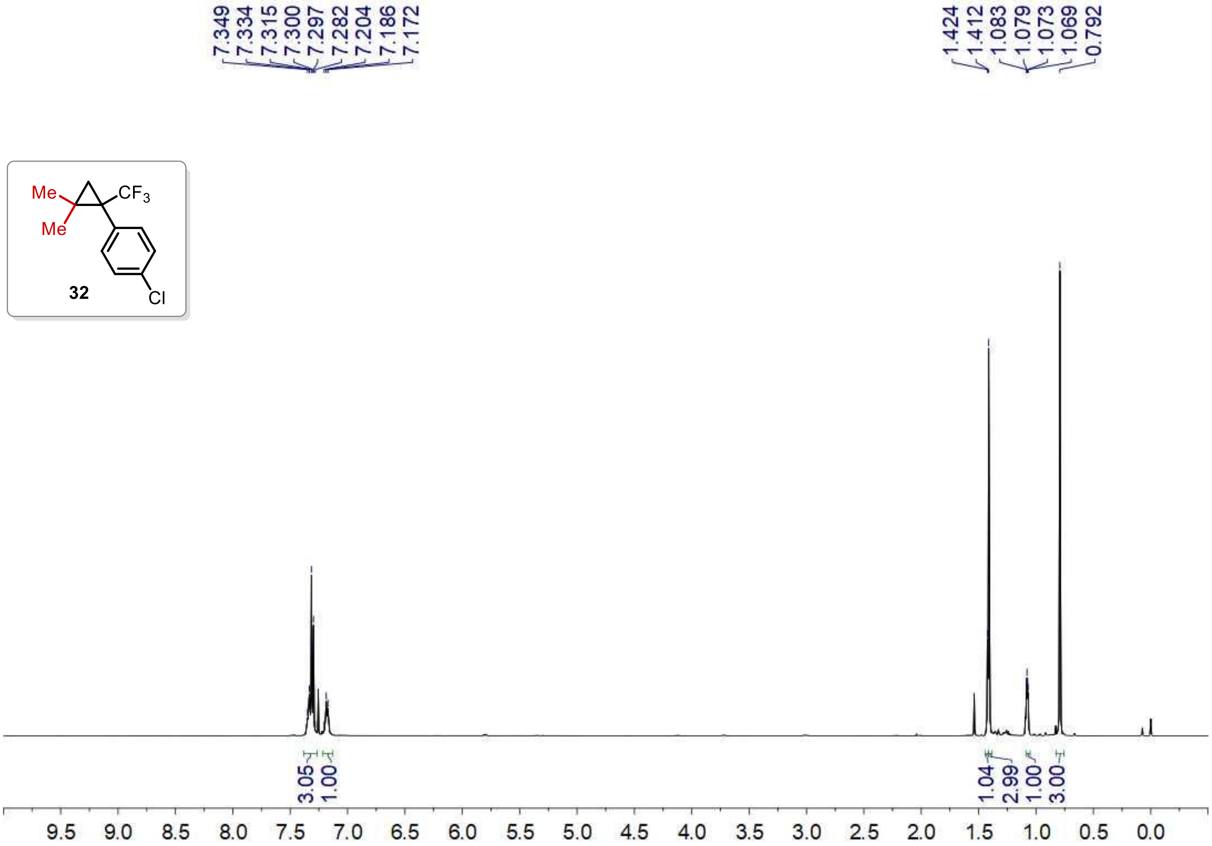

Supplementary Fig. 90 ^{1}H NMR (500 MHz, $CDCl_3$) spectrum of compound **32**.

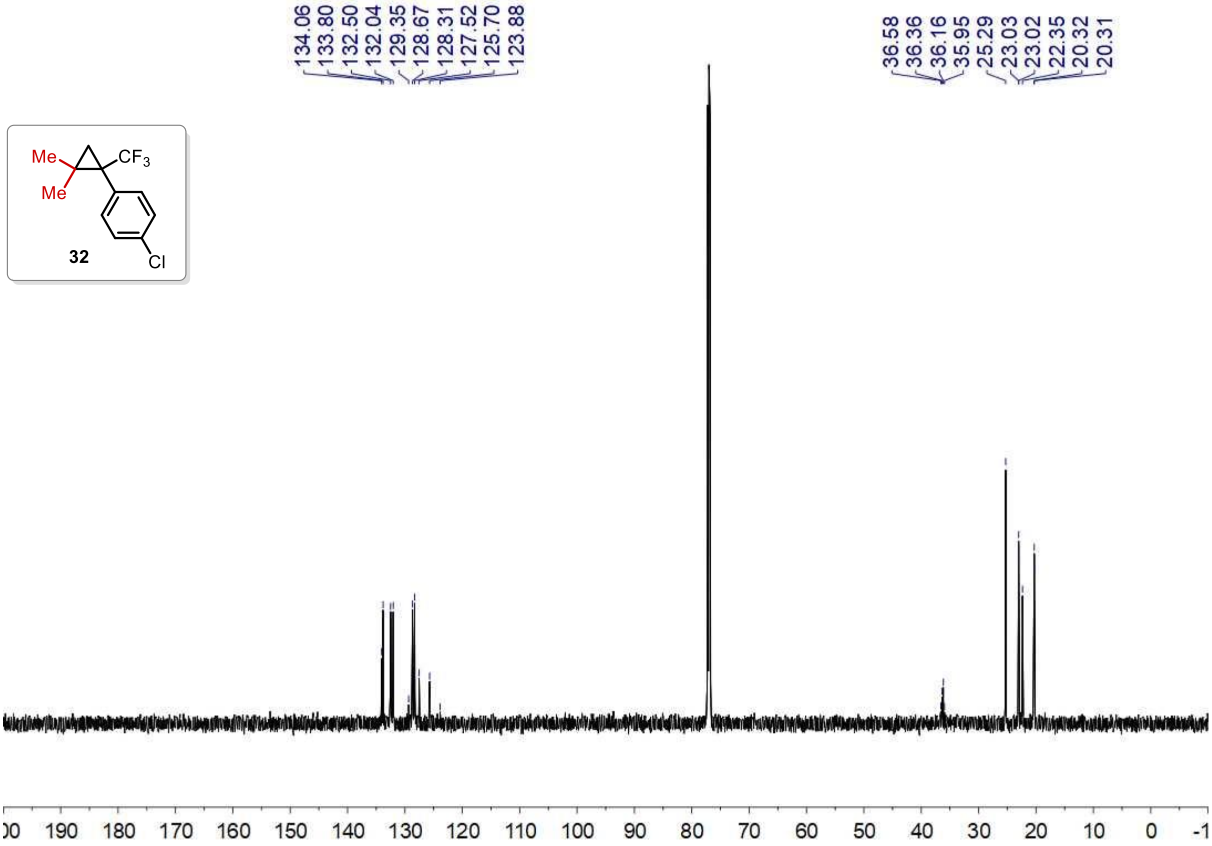

Supplementary Fig. 91 ^{13}**C NMR** (150 MHz, $CDCl_3$) spectrum of compound **32**.

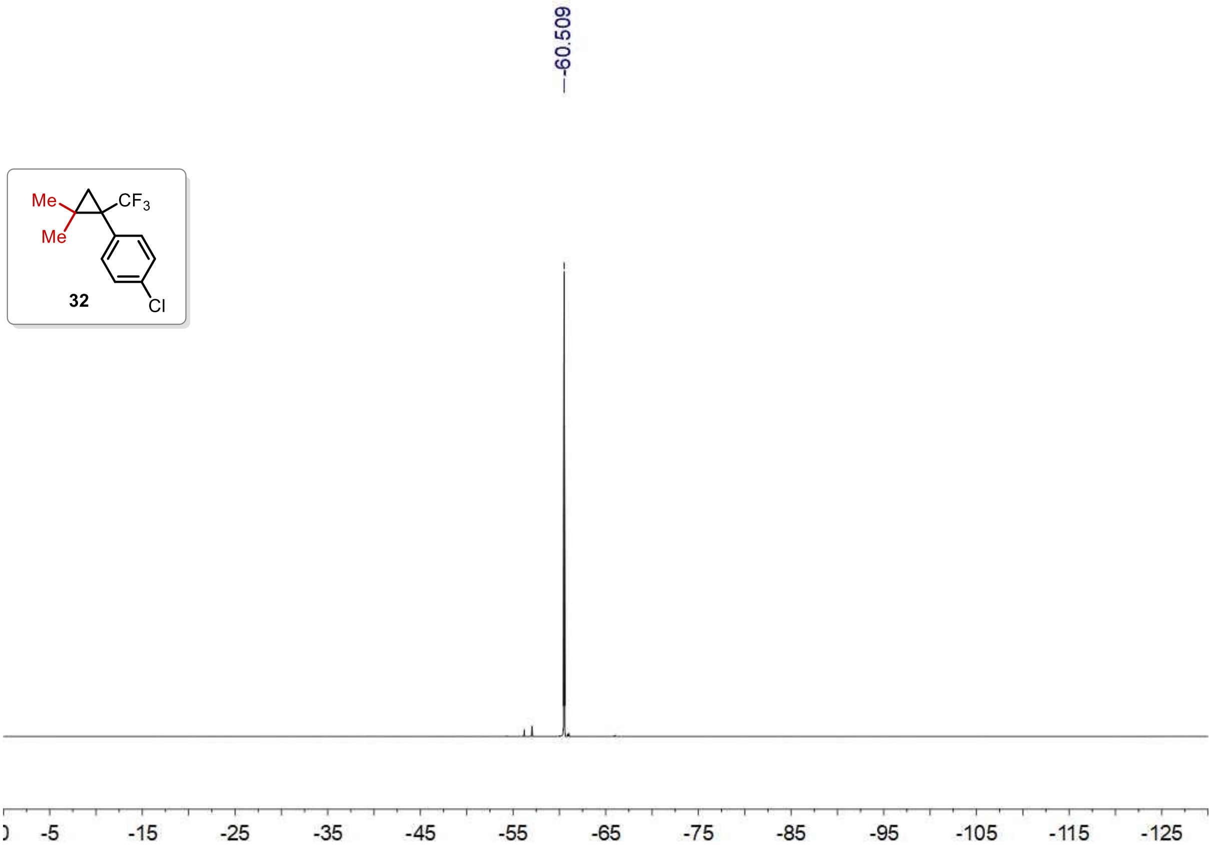

Supplementary Fig. 92 ^{19}**F NMR** (564 MHz, $CDCl_3$) spectrum of compound **32**.

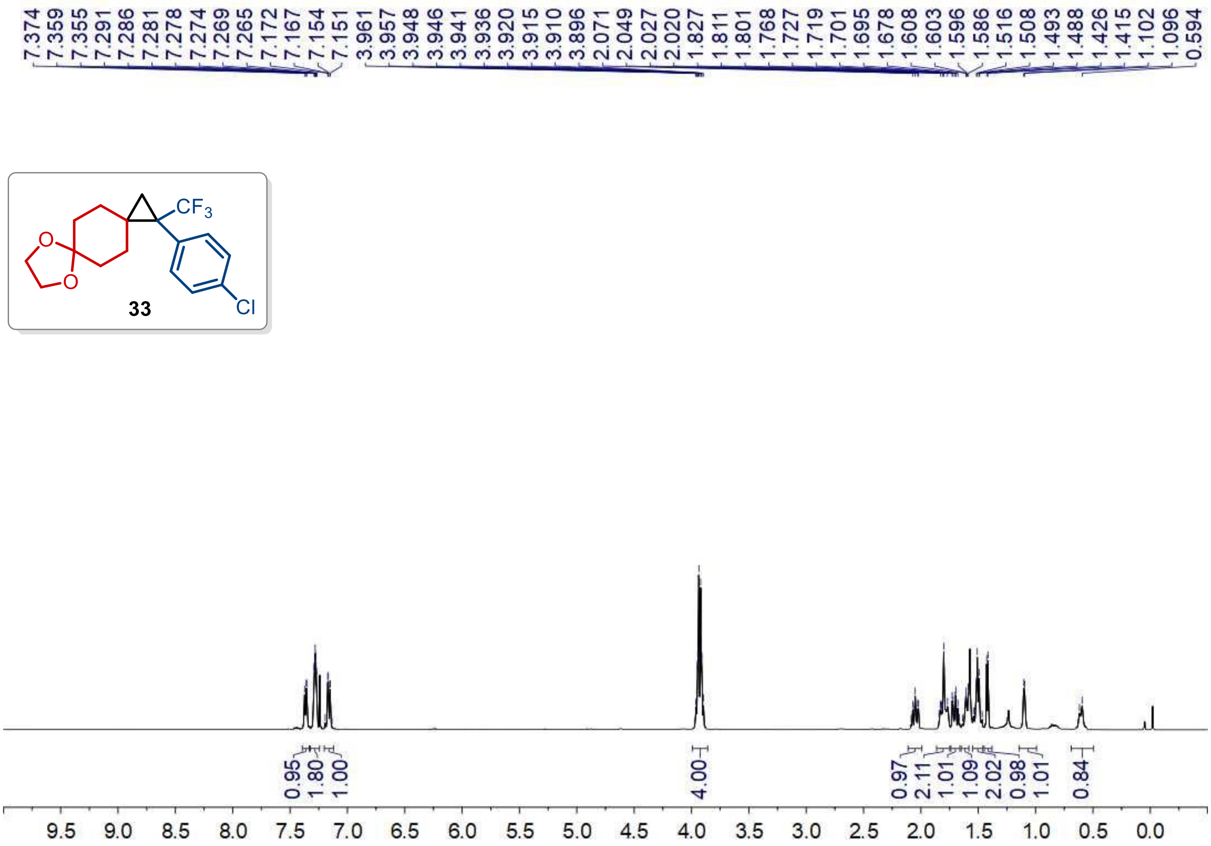

Supplementary Fig. 93 ^{1}H NMR (500 MHz, $CDCl_3$) spectrum of compound **33**.

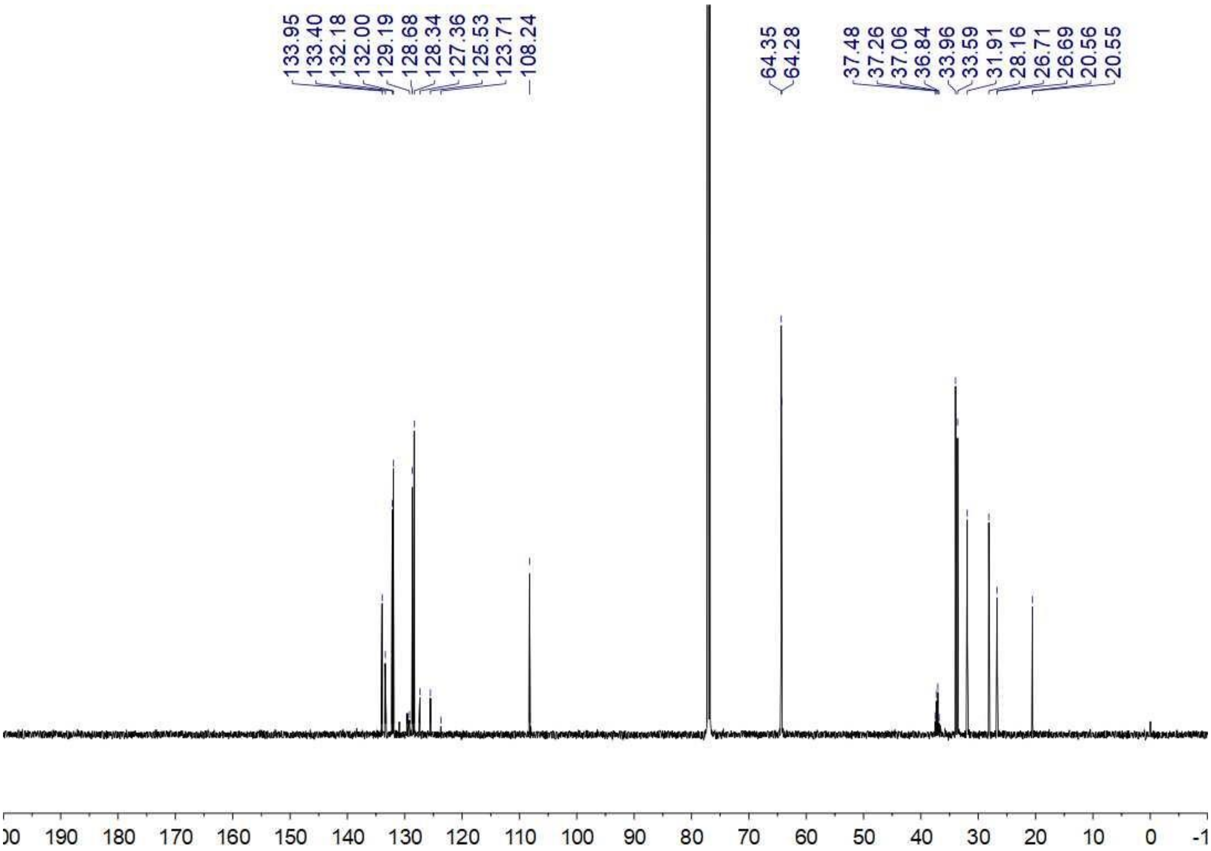

Supplementary Fig. 94 ^{13}C NMR (150 MHz, $CDCl_3$) spectrum of compound **33**.

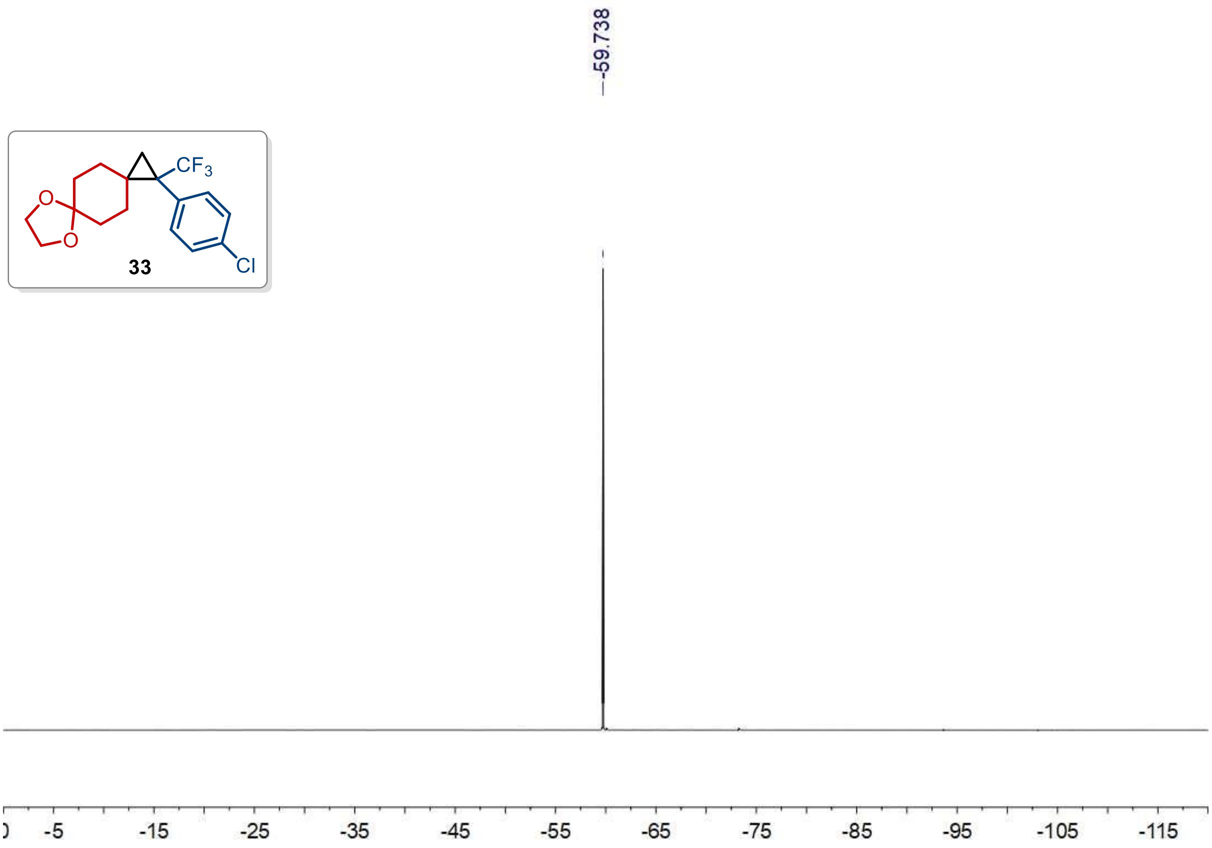

Supplementary Fig. 95 ^{19}**F NMR** (564 MHz, $CDCl_3$) spectrum of compound **33**.

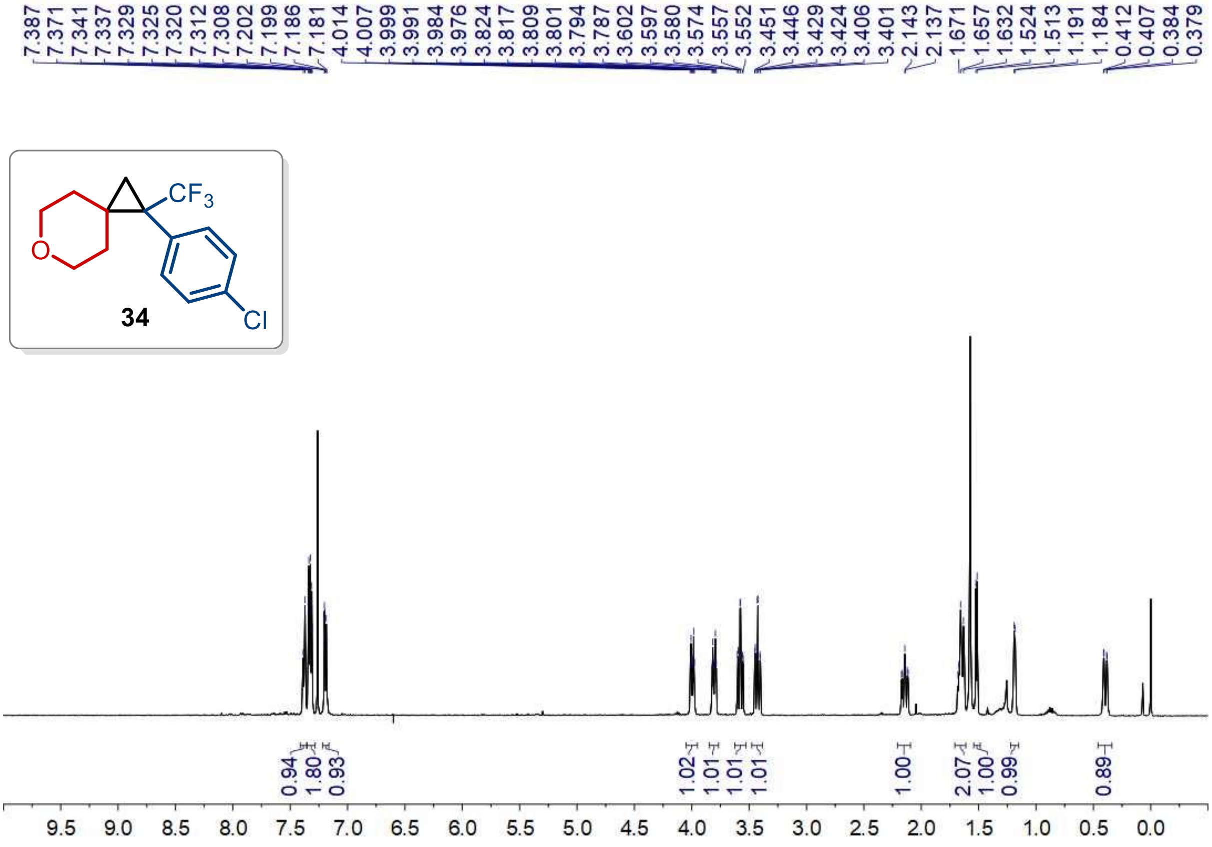

Supplementary Fig. 96 ^{1}**H NMR** (500 MHz, $CDCl_3$) spectrum of compound **34**.

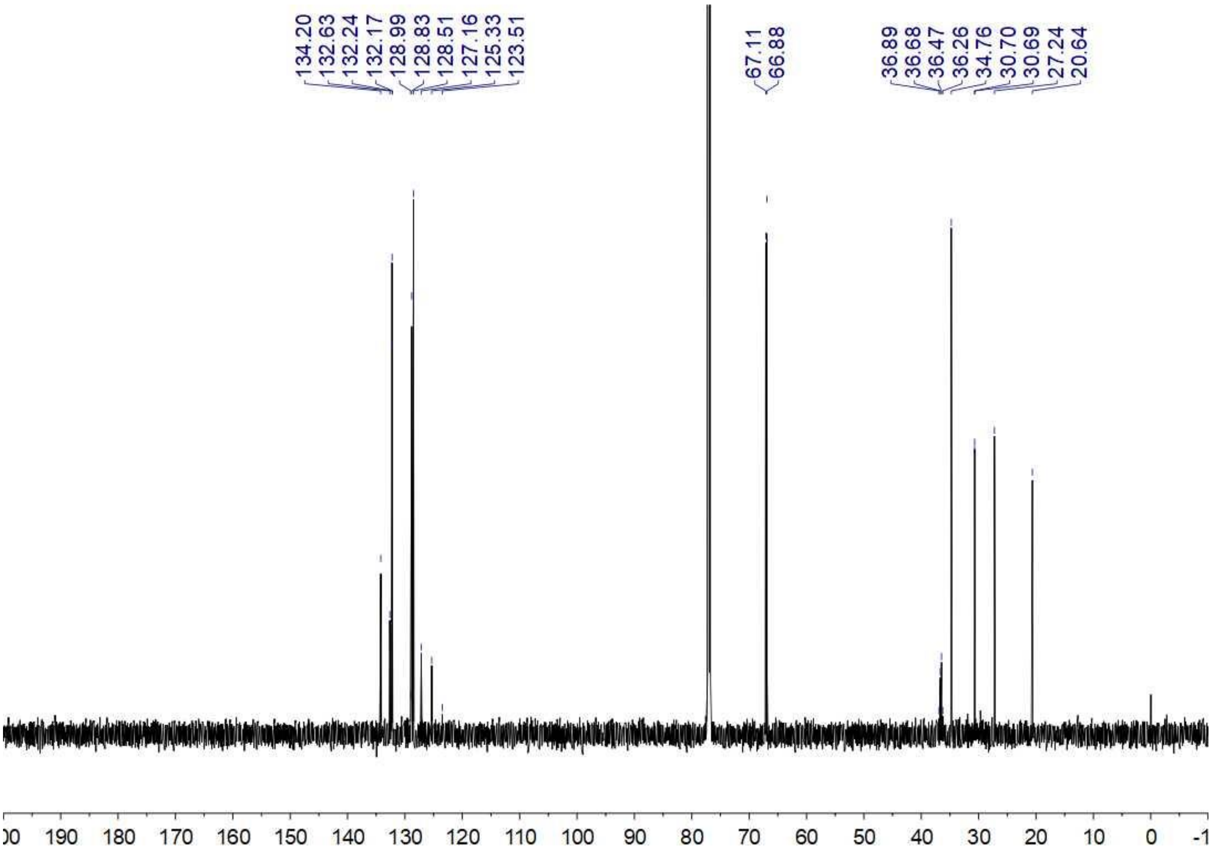

Supplementary Fig. 97 ^{13}C NMR (150 MHz, $CDCl_3$) spectrum of compound **34**.

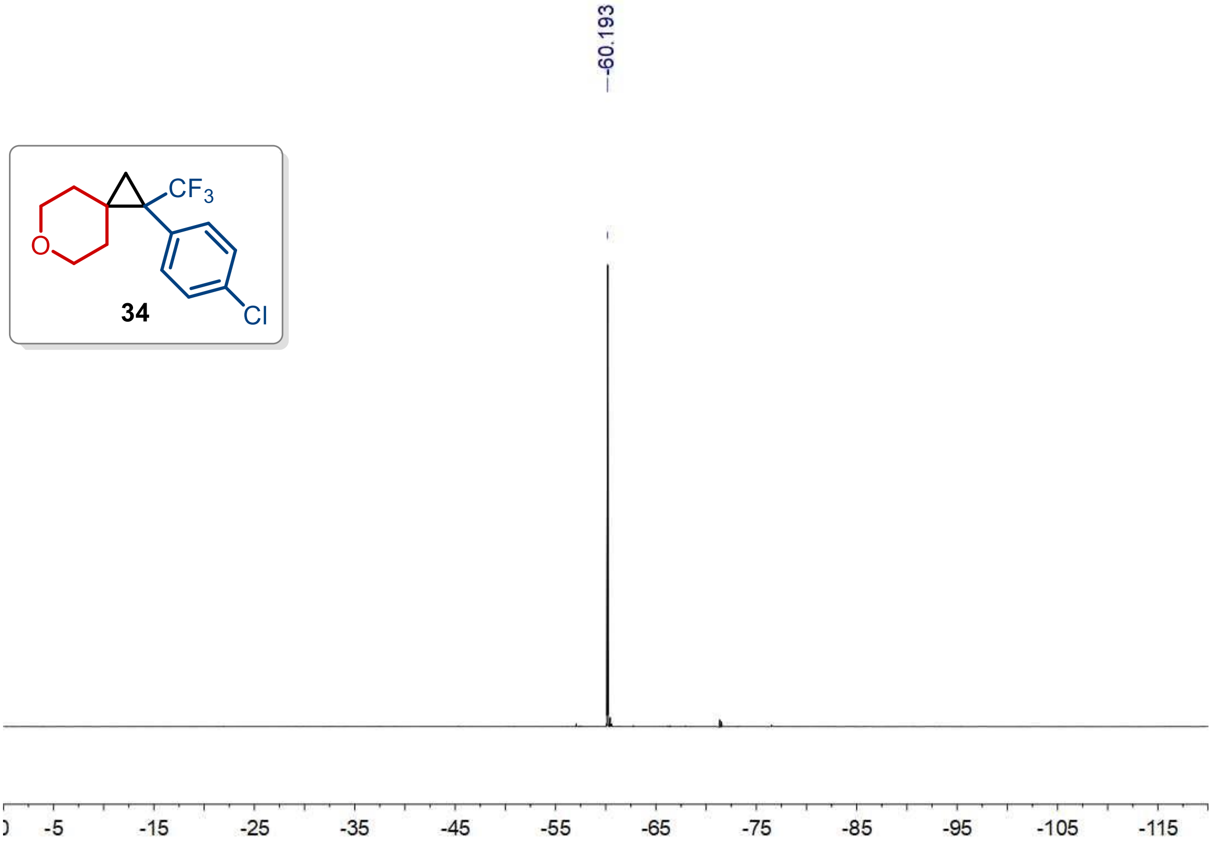

Supplementary Fig. 98 ^{19}F NMR (564 MHz, $CDCl_3$) spectrum of compound **34**.

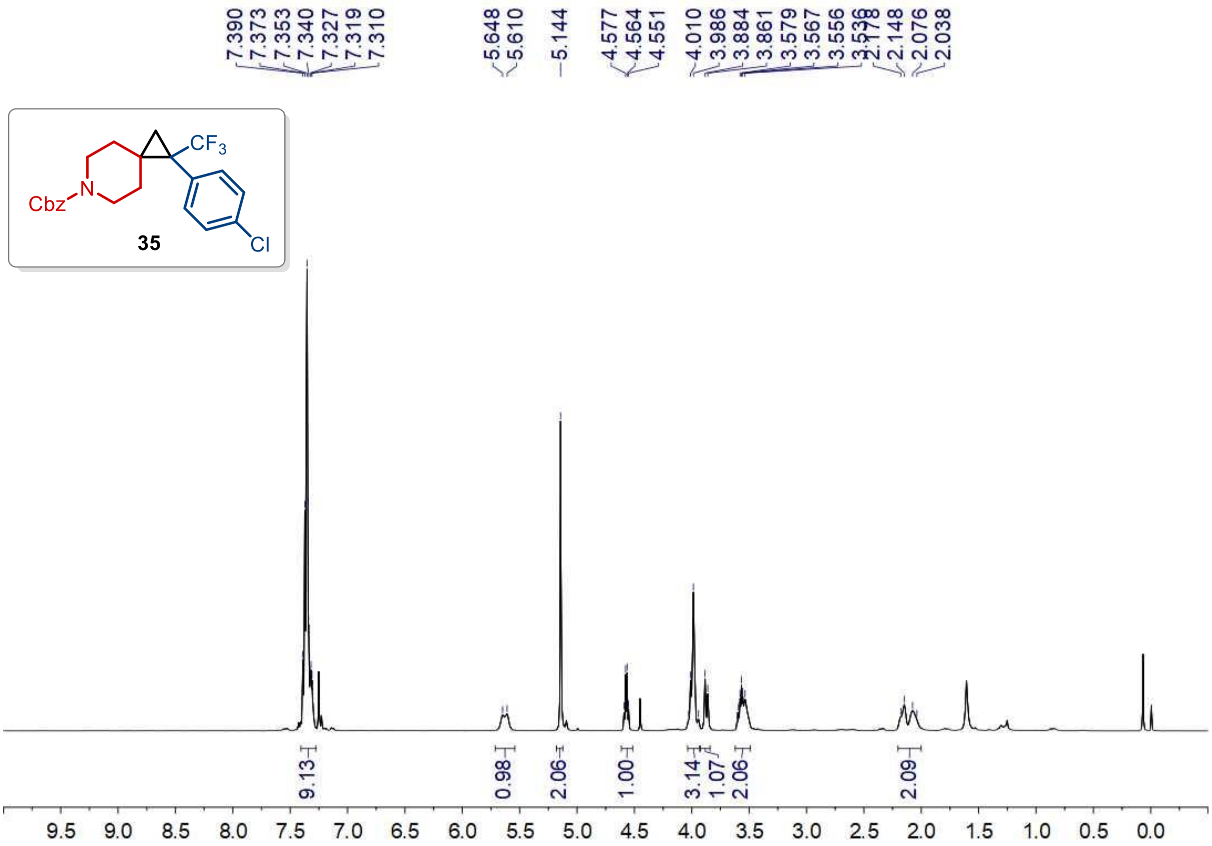

Supplementary Fig. 99 ^{1}**H NMR** (500 MHz, $CDCl_3$) spectrum of compound **35**.

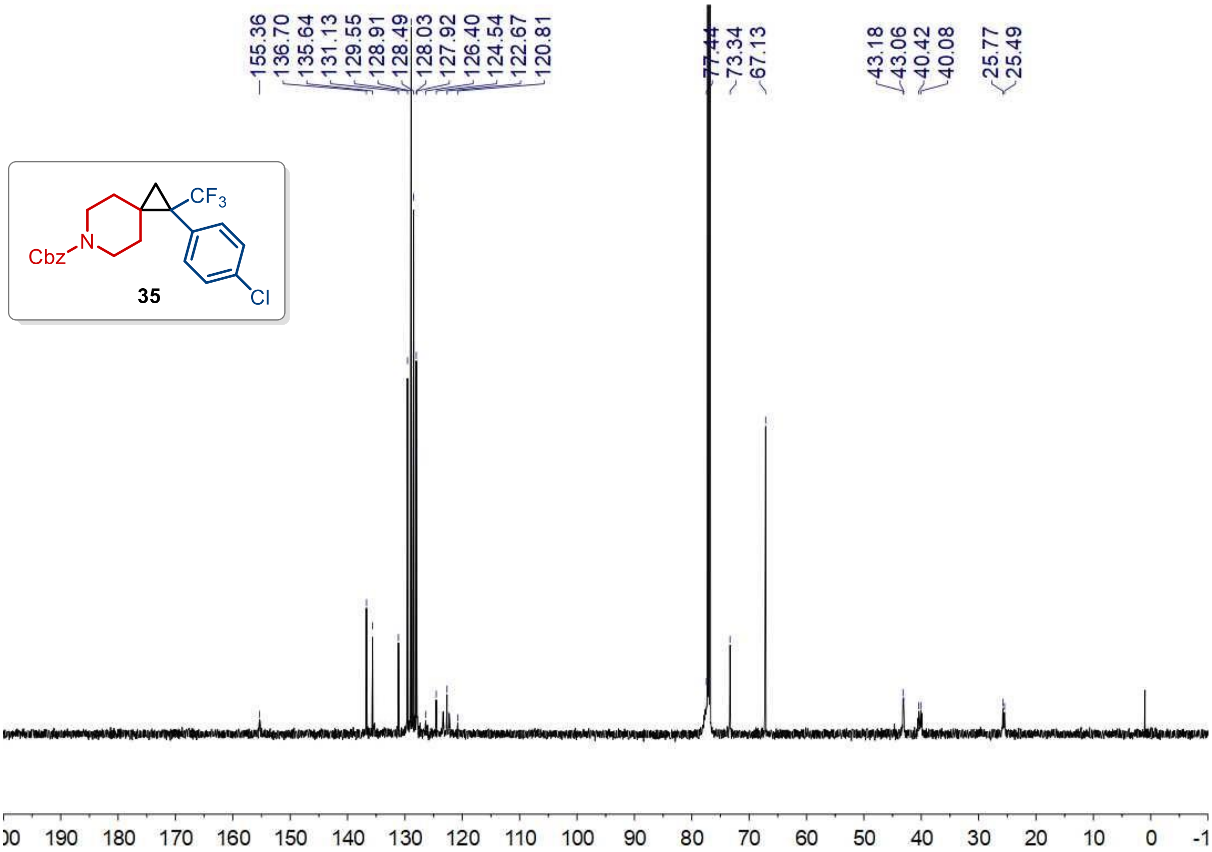

Supplementary Fig. 100 ^{13}**C NMR** (150 MHz, $CDCl_3$) spectrum of compound **35**.

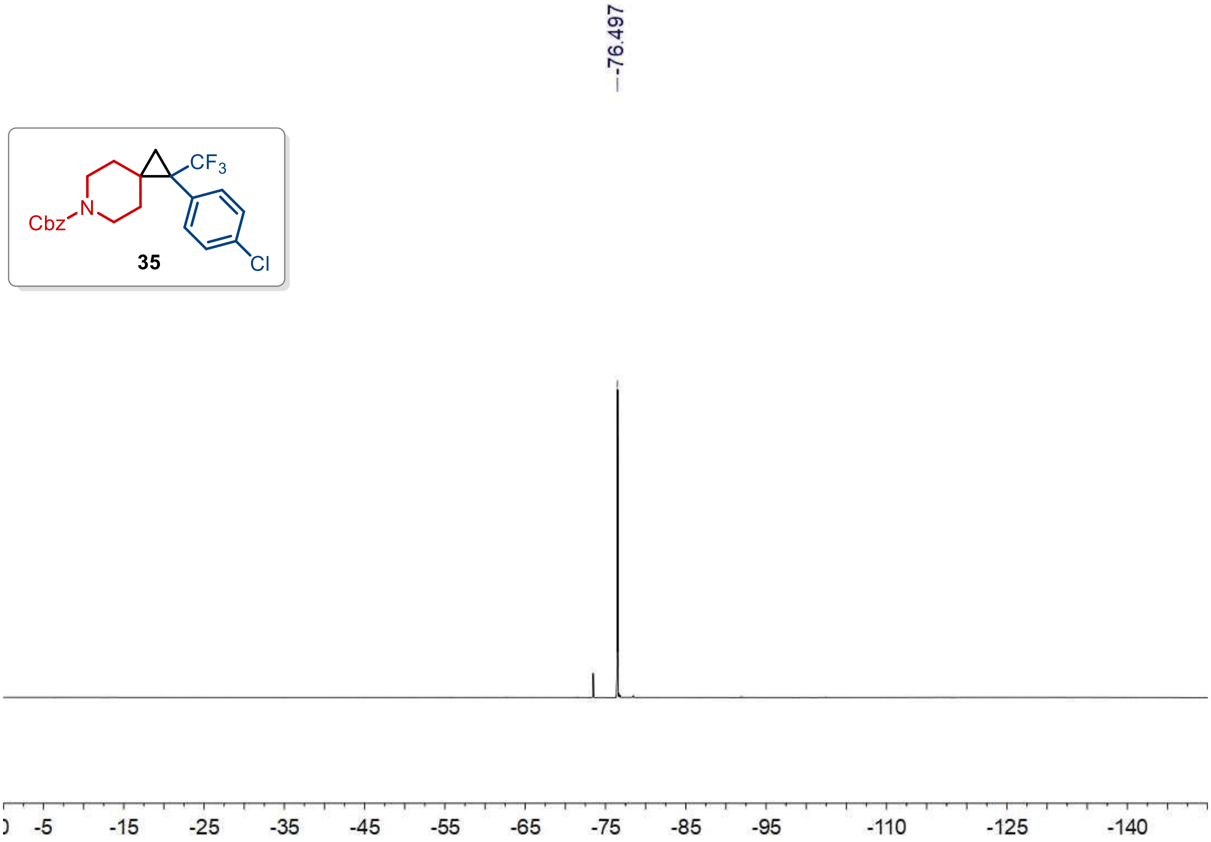

Supplementary Fig. 101 ^{19}**F NMR** (564 MHz, $CDCl_3$) spectrum of compound **35**.

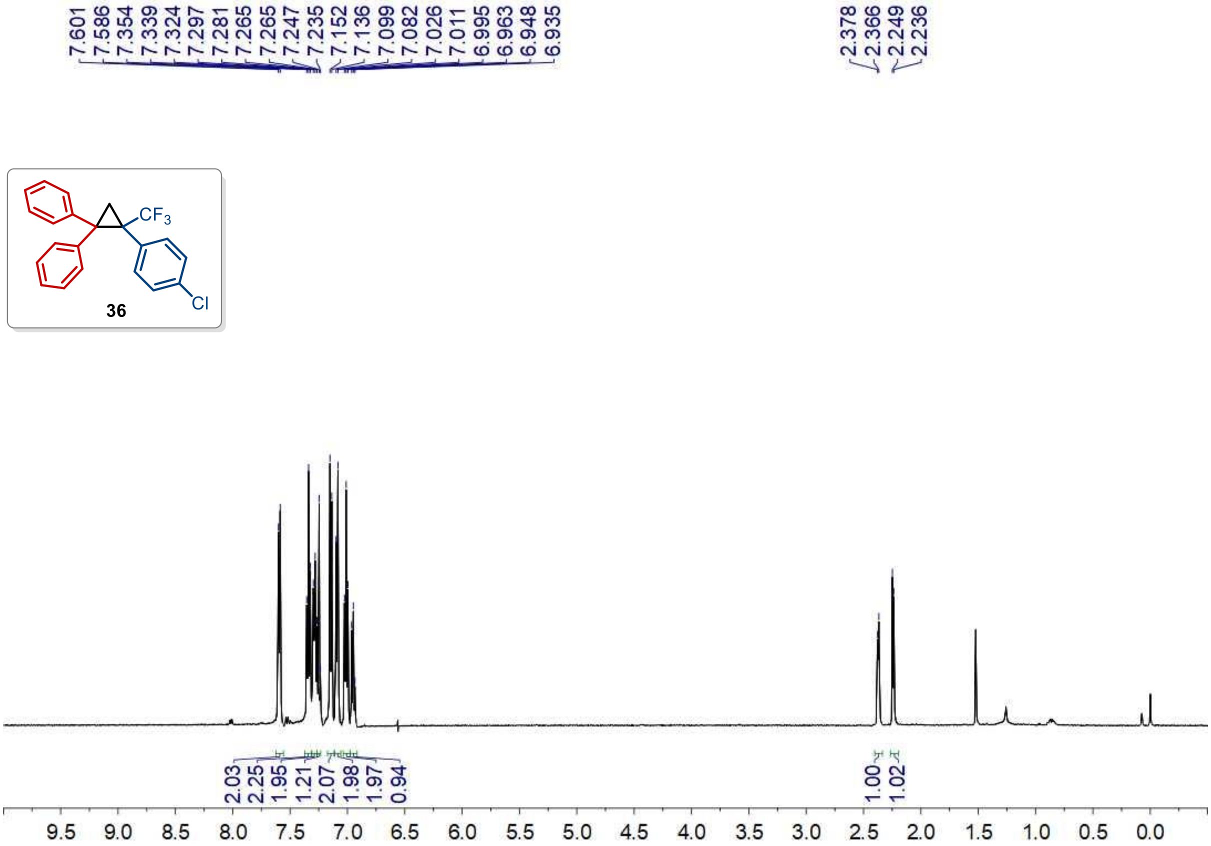

Supplementary Fig. 102 ^{1}**H NMR** (500 MHz, $CDCl_3$) spectrum of compound **36**.

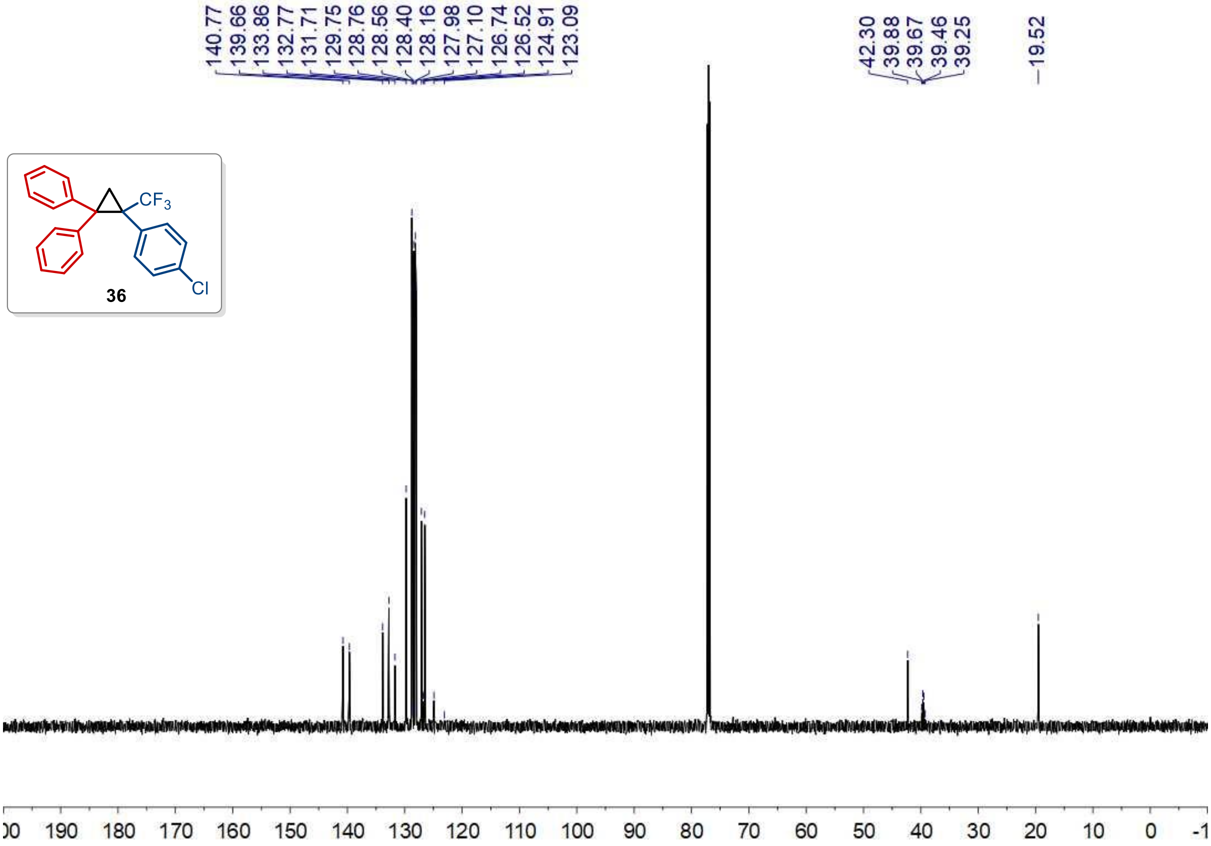

Supplementary Fig. 103 ^{13}**C NMR** (150 MHz, $CDCl_3$) spectrum of compound **36**.

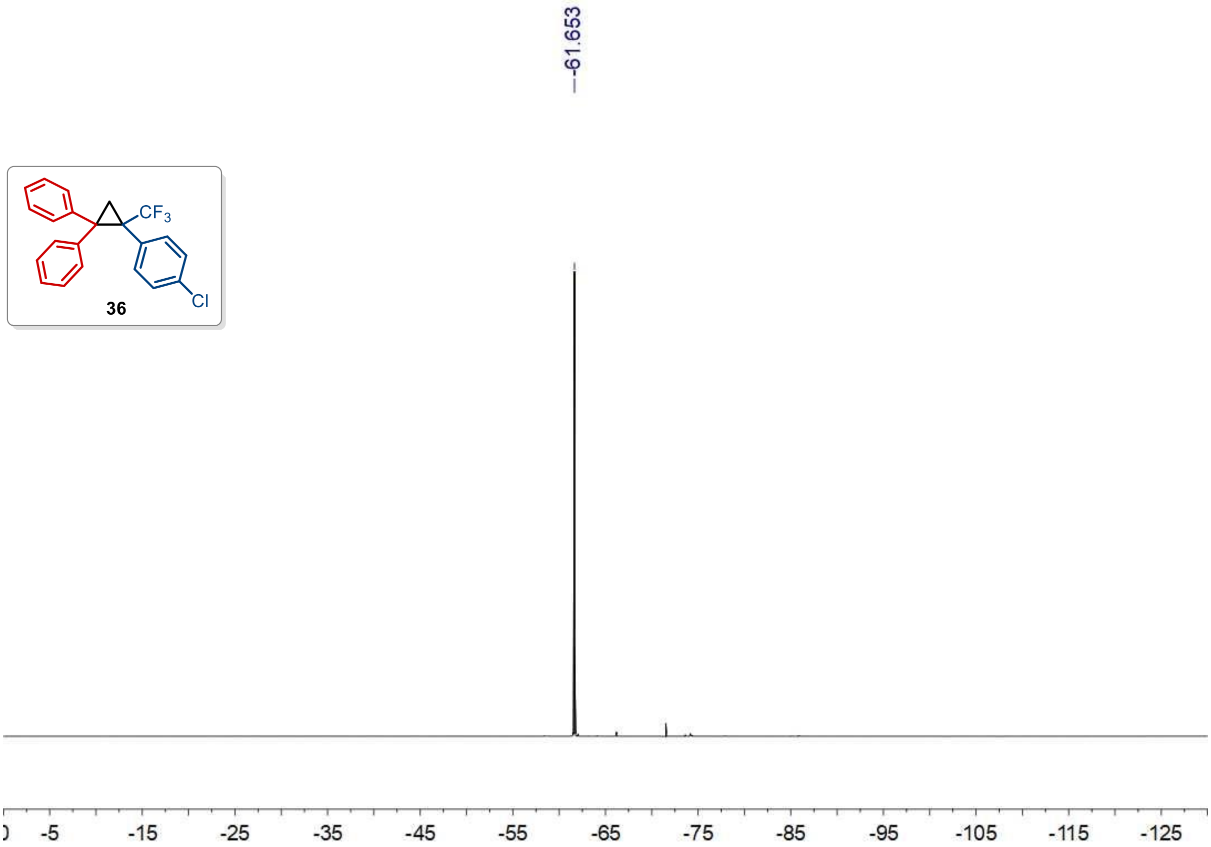

Supplementary Fig. 104 ^{19}**F NMR** (564 MHz, $CDCl_3$) spectrum of compound **36**.

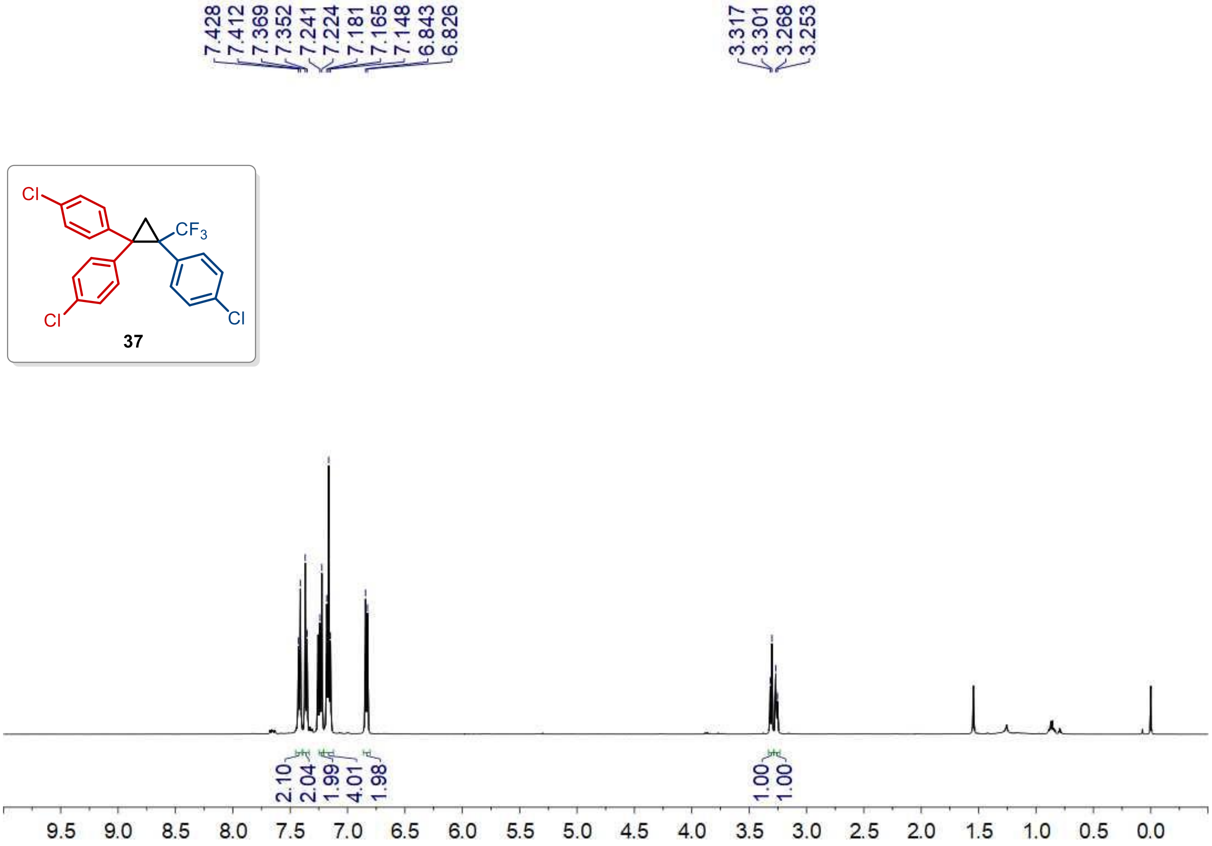

Supplementary Fig. 105 ^{1}H NMR (500 MHz, $CDCl_3$) spectrum of compound **37**.

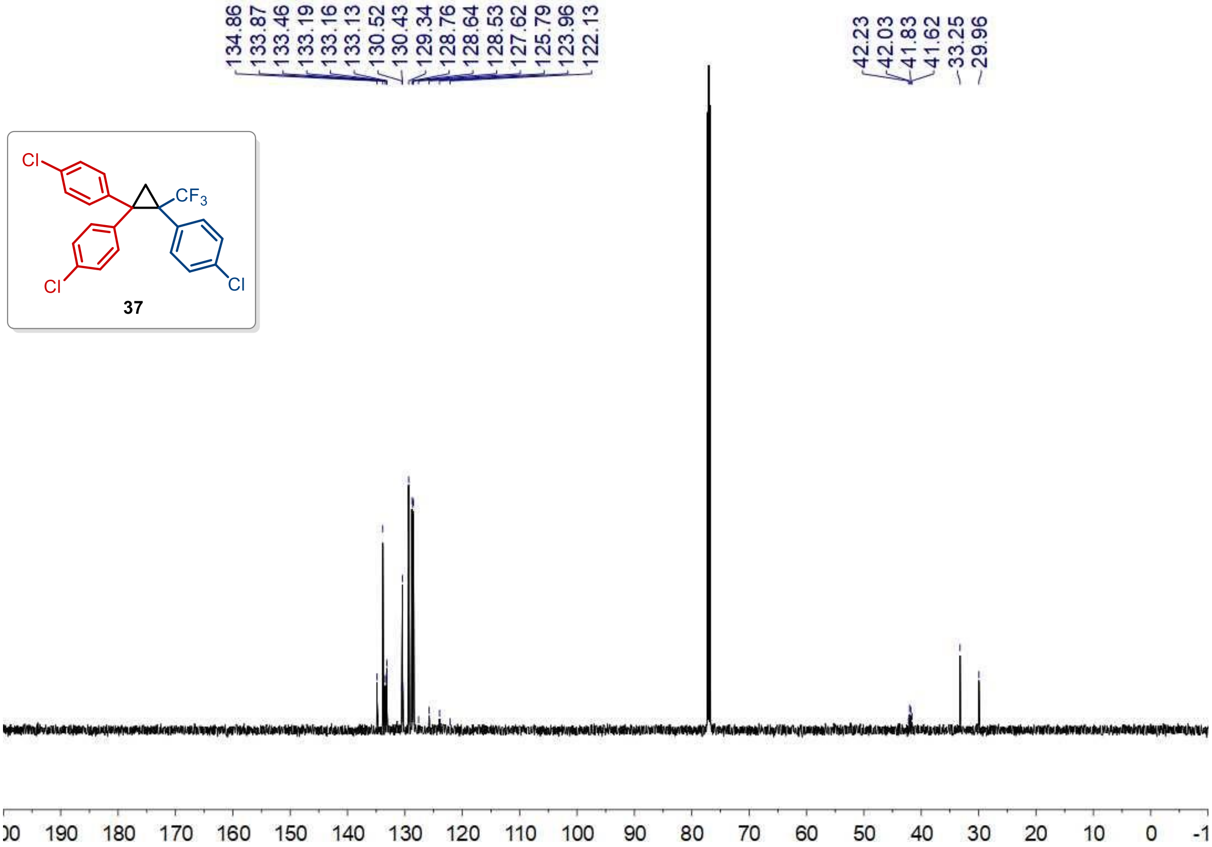

Supplementary Fig. 106 ^{13}C NMR (150 MHz, $CDCl_3$) spectrum of compound **37**.

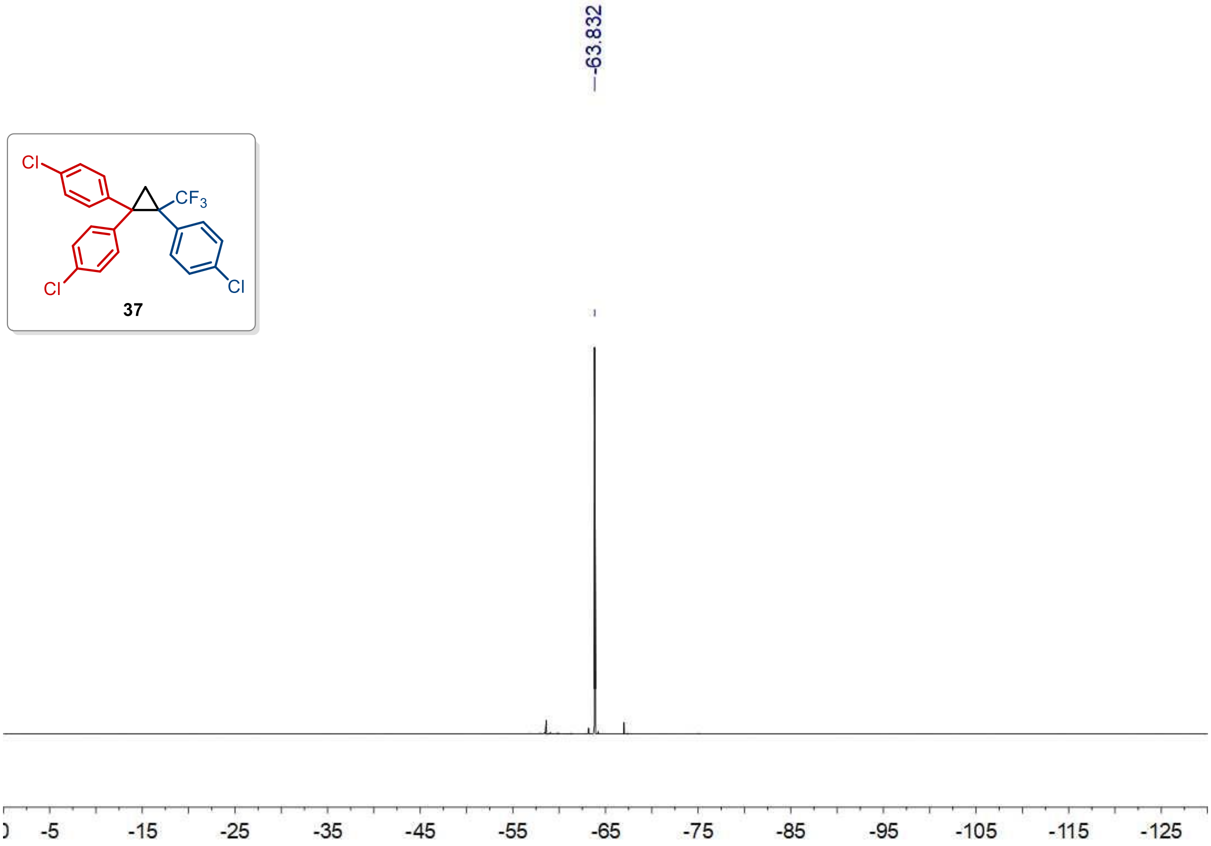

Supplementary Fig. 107 ^{19}**F NMR** (564 MHz, $CDCl_3$) spectrum of compound **37**.

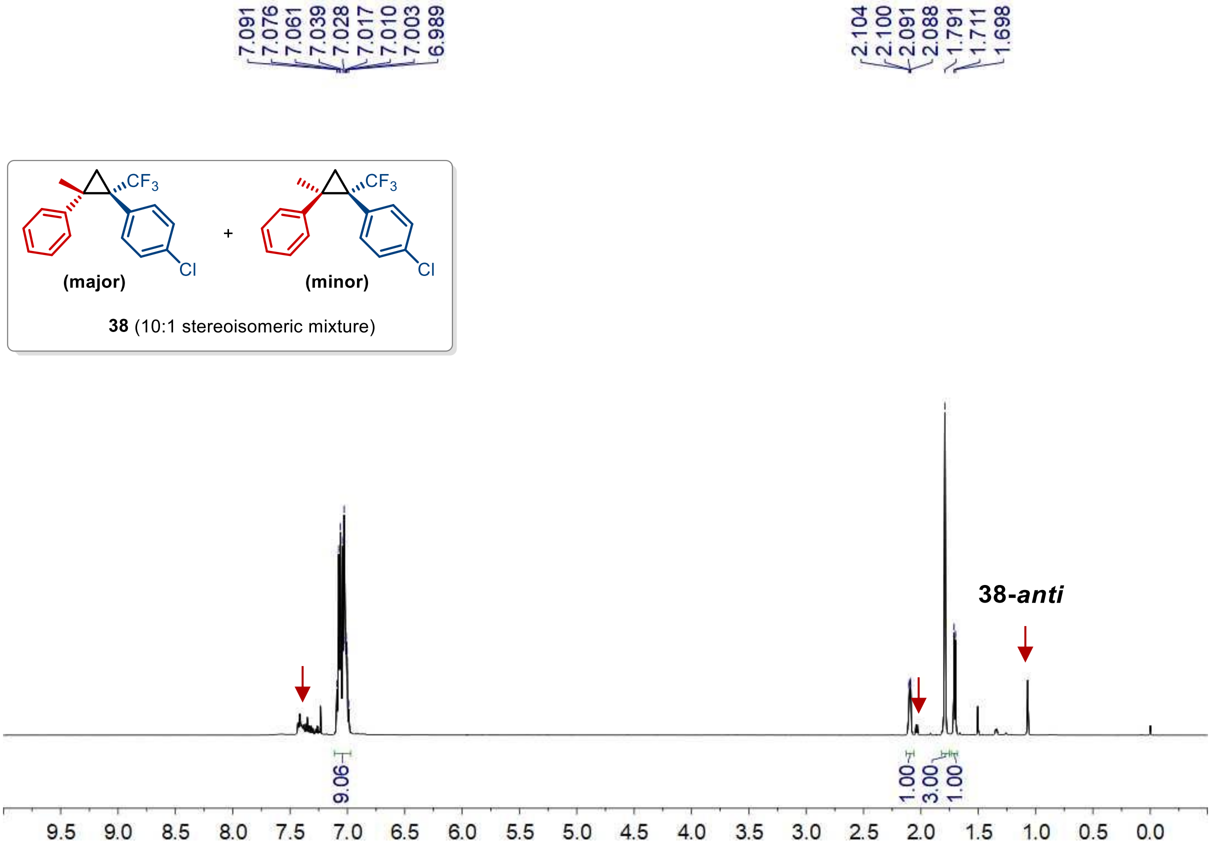

Supplementary Fig. 108 ^{1}**H NMR** (500 MHz, $CDCl_3$) spectrum of compound **38**.

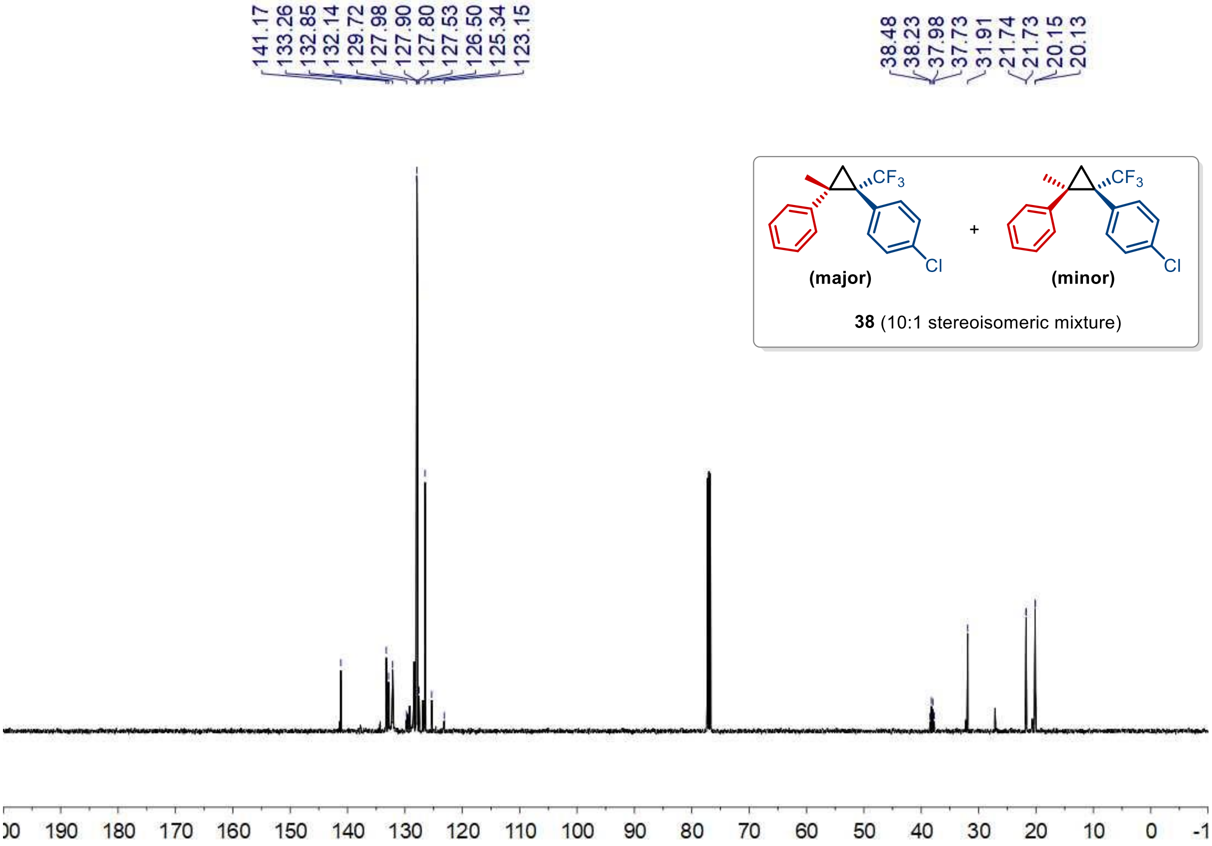

Supplementary Fig. 109 **^{13}C NMR** (125 MHz, $CDCl_3$) spectrum of compound **38**.

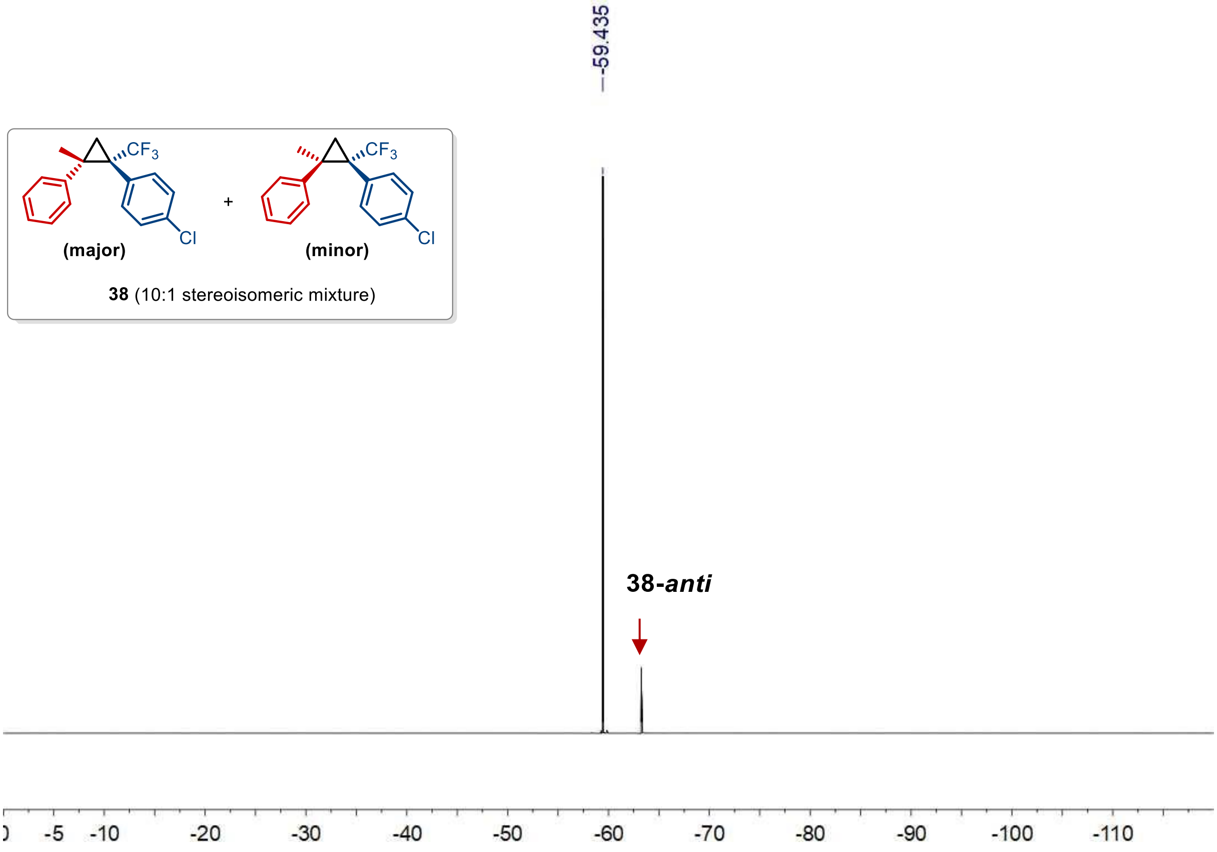

Supplementary Fig. 110 **^{19}F NMR** (470 MHz, $CDCl_3$) spectrum of compound **38**.

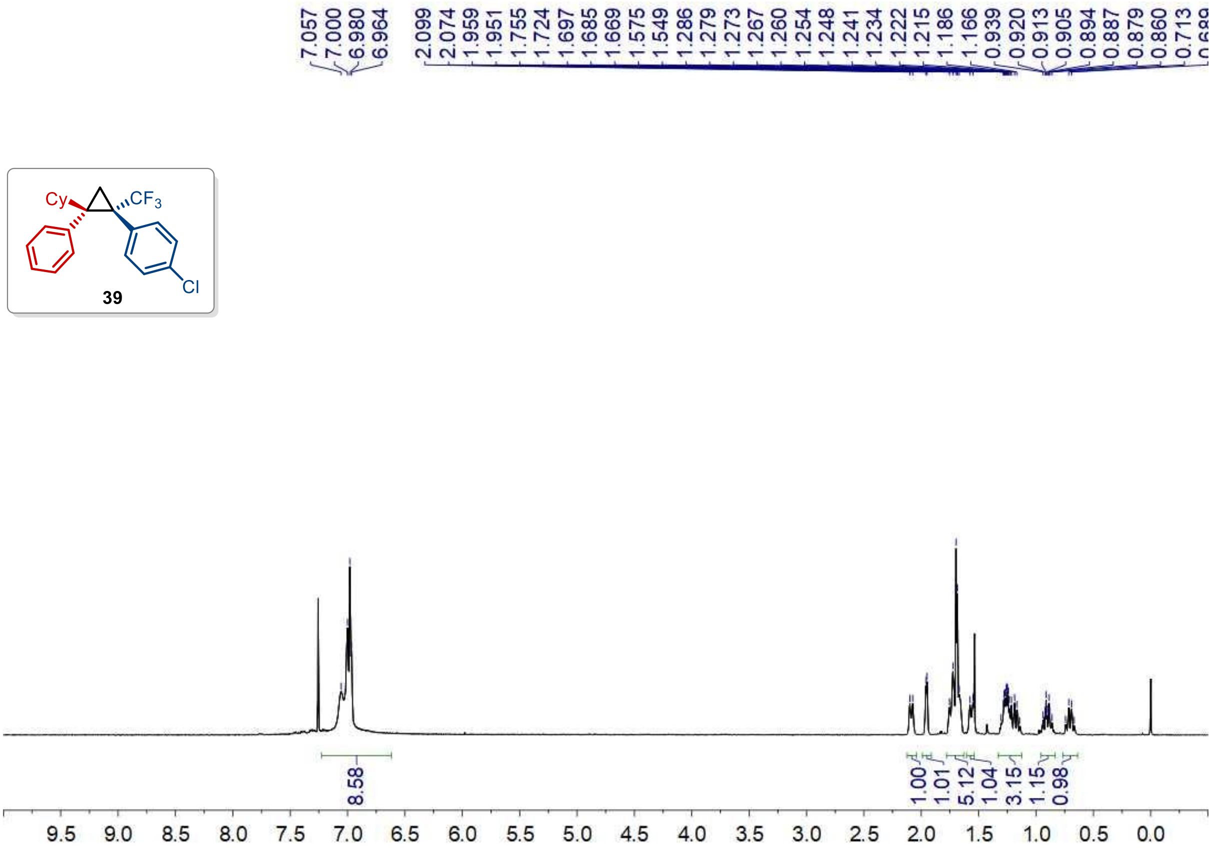

Supplementary Fig. 111 **^{1}H NMR** (500 MHz, $CDCl_3$) spectrum of compound **39**.

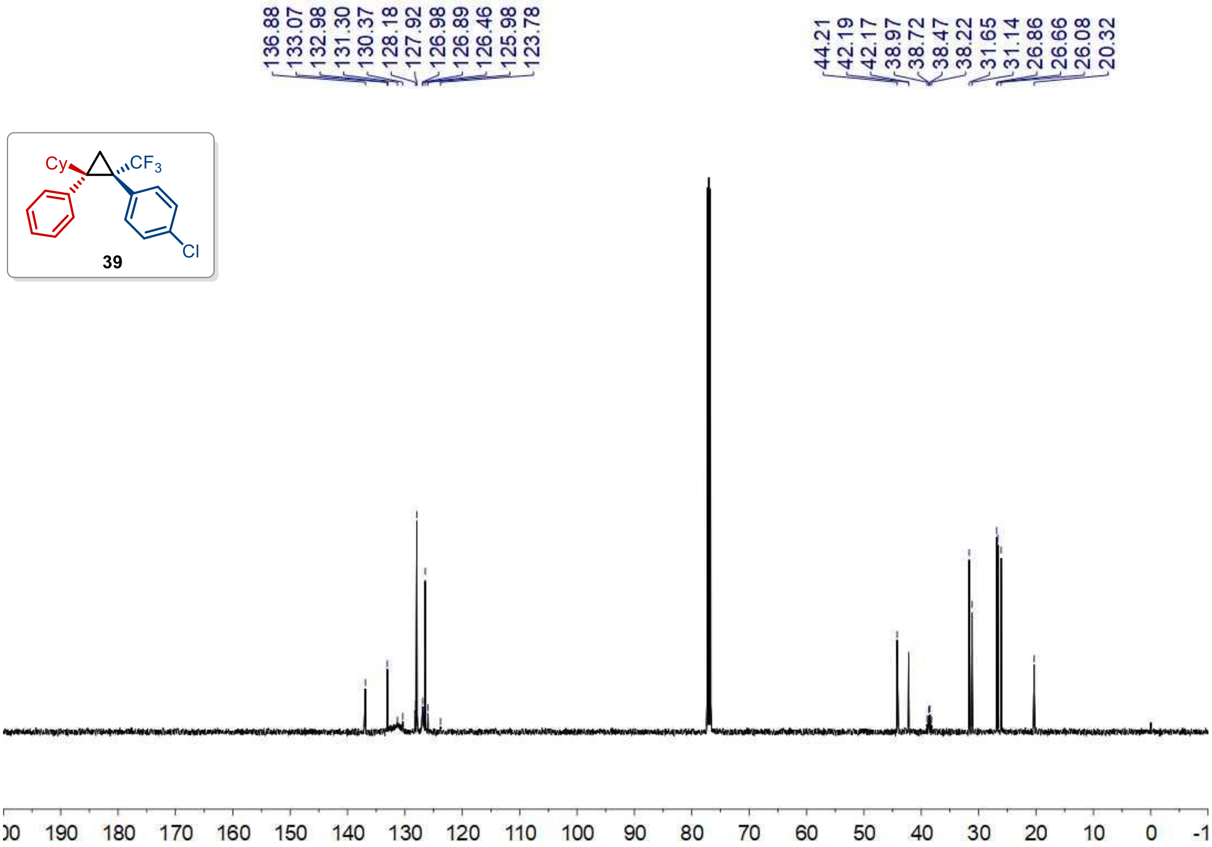

Supplementary Fig. 112 **^{13}C NMR** (125 MHz, $CDCl_3$) spectrum of compound **39**.

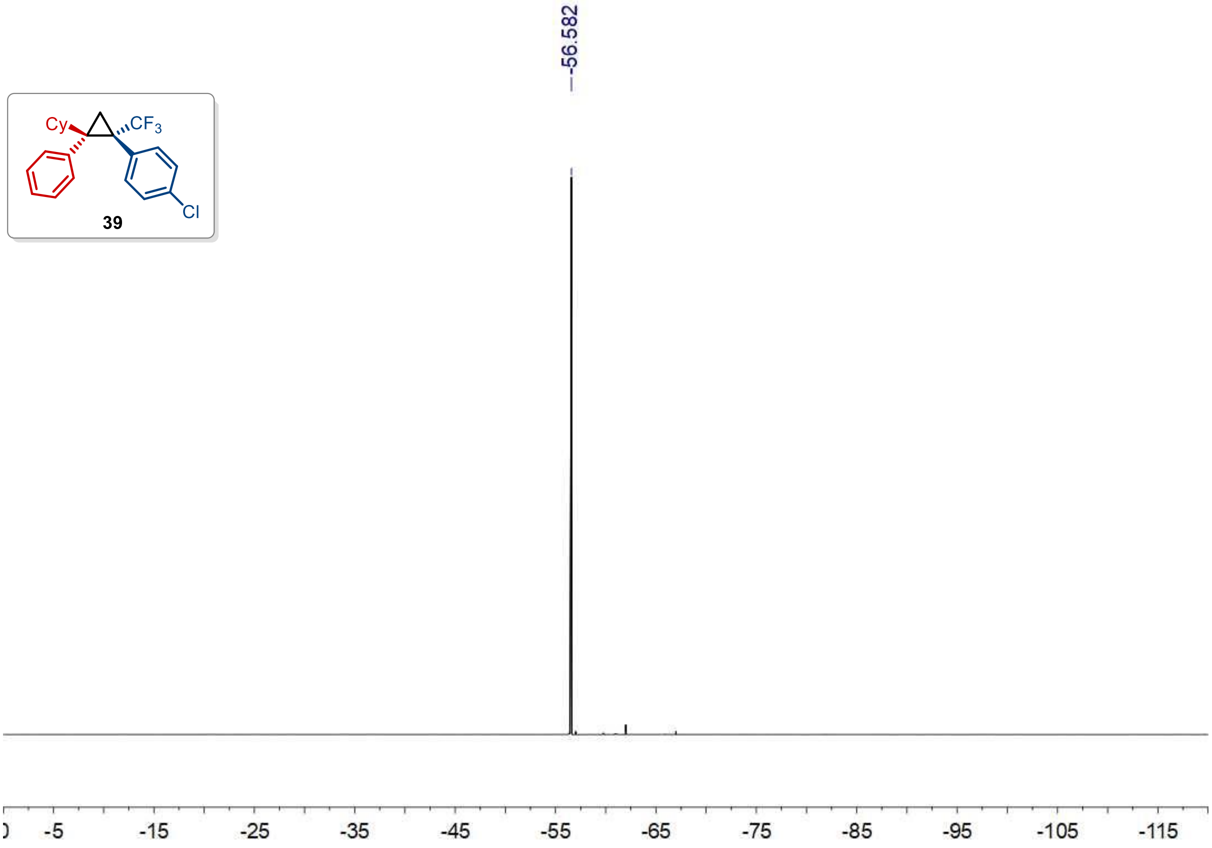

Supplementary Fig. 113 ^{19}**F NMR** (564 MHz, $CDCl_3$) spectrum of compound **39**.

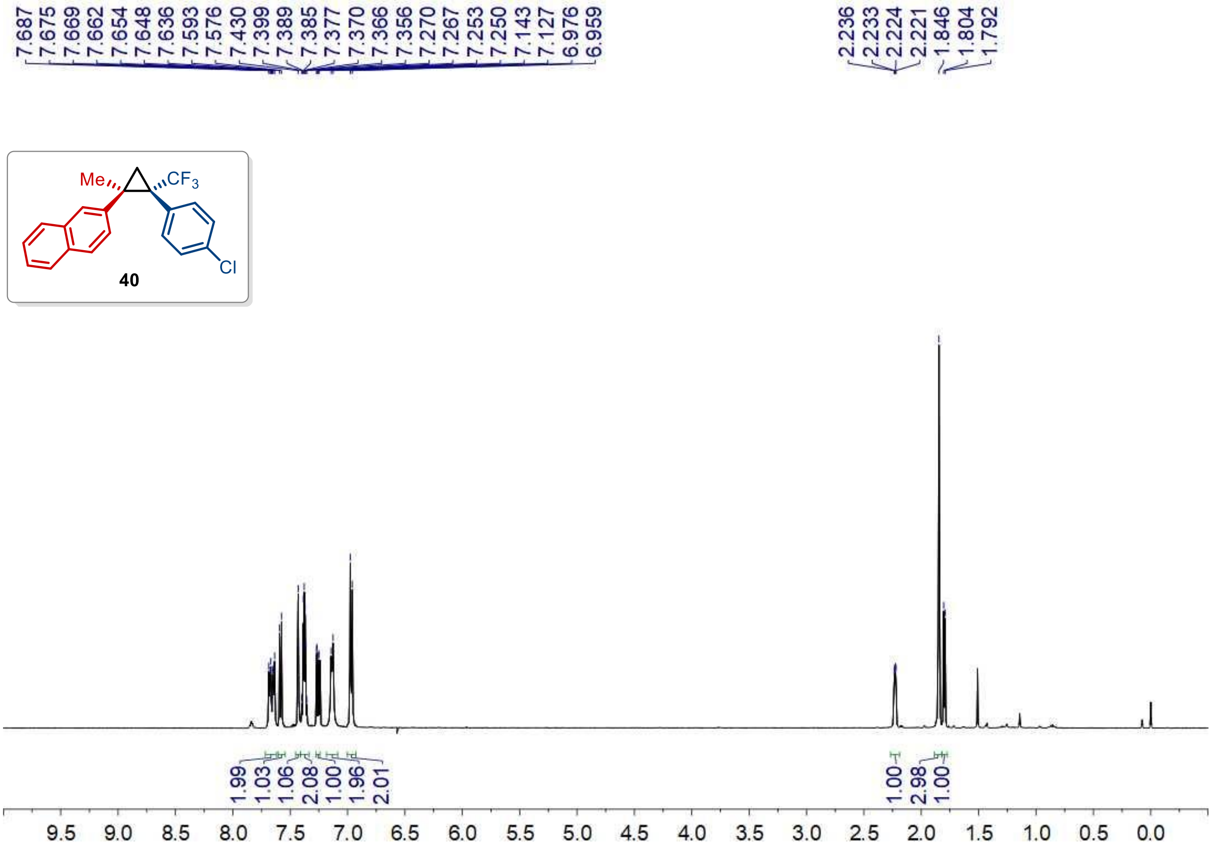

Supplementary Fig. 114 ^{1}**H NMR** (500 MHz, $CDCl_3$) spectrum of compound **40**.

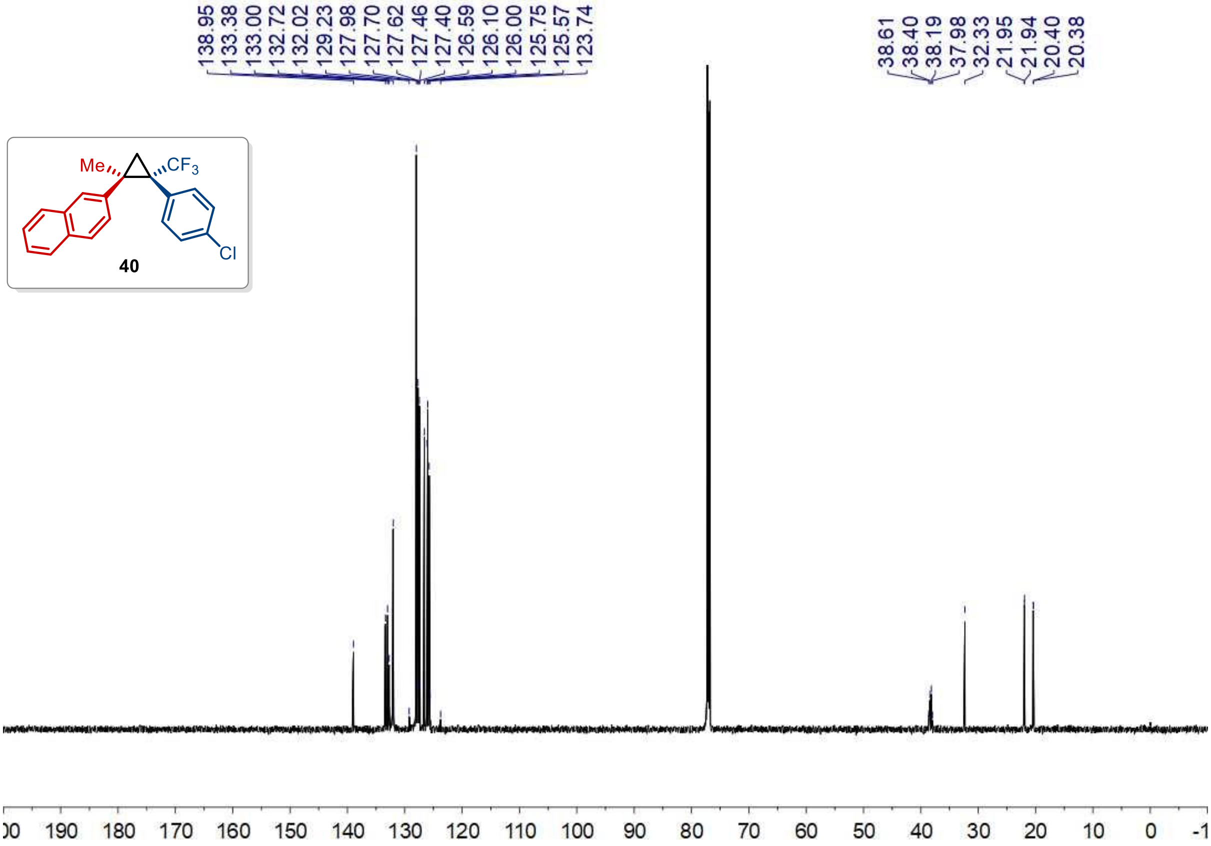

Supplementary Fig. 115 ^{13}**C NMR** (150 MHz, $CDCl_3$) spectrum of compound **40**.

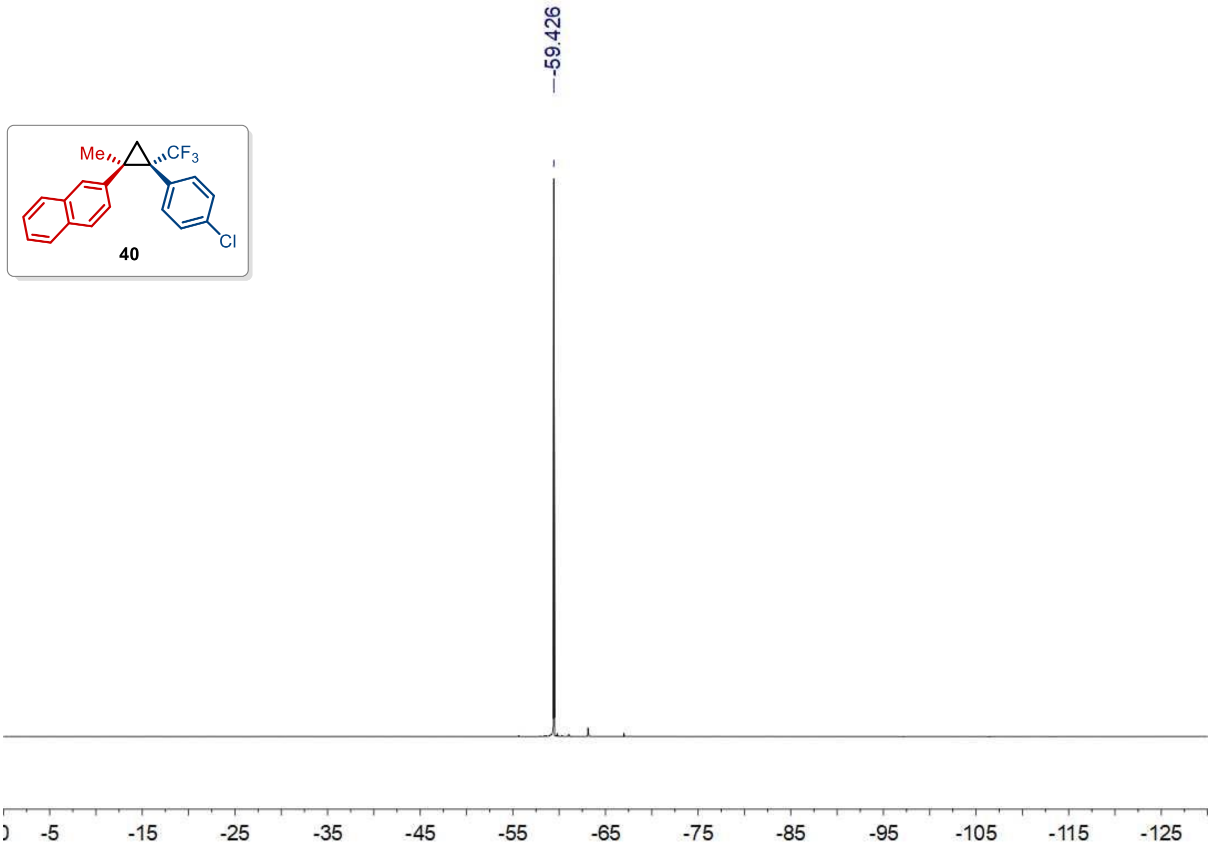

Supplementary Fig. 116 ^{19}**F NMR** (564 MHz, $CDCl_3$) spectrum of compound **40**.

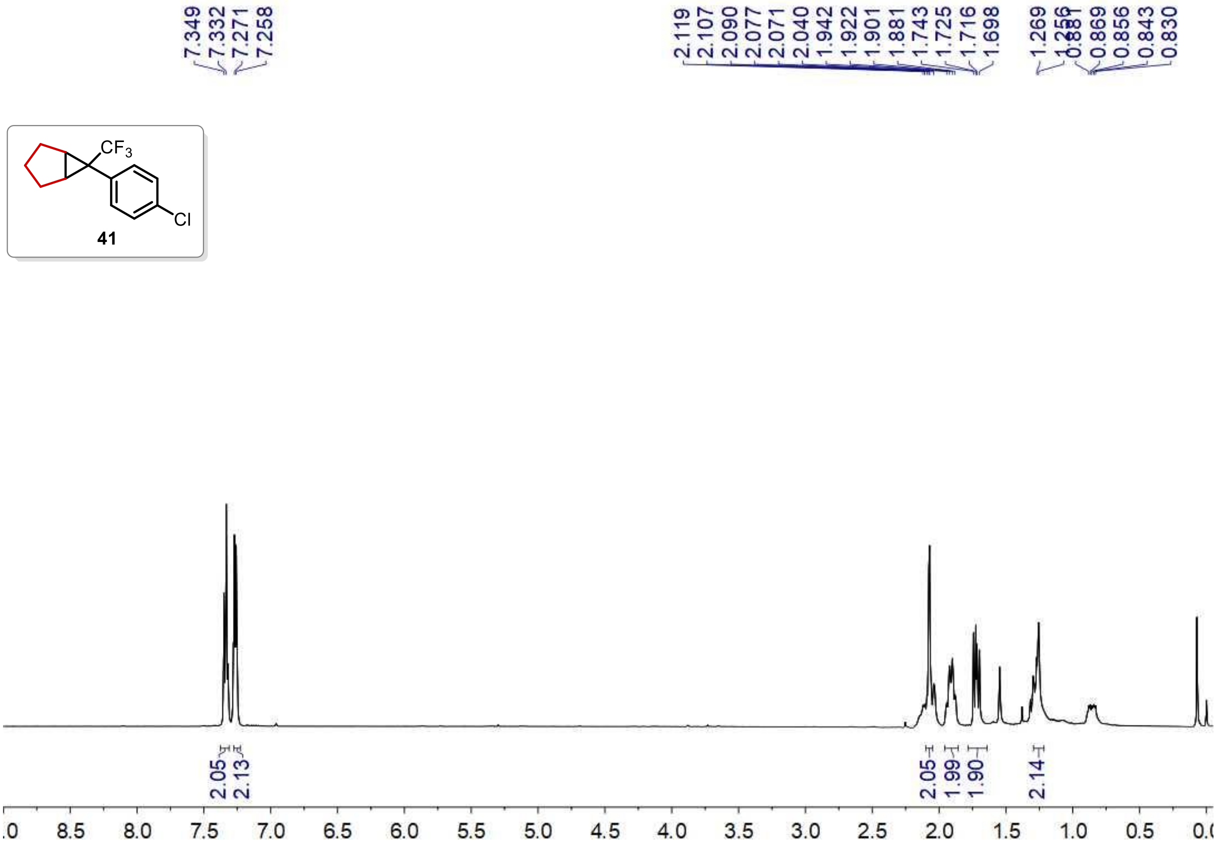

Supplementary Fig. 117 ^{1}**H NMR** (500 MHz, $CDCl_3$) spectrum of compound **41**.

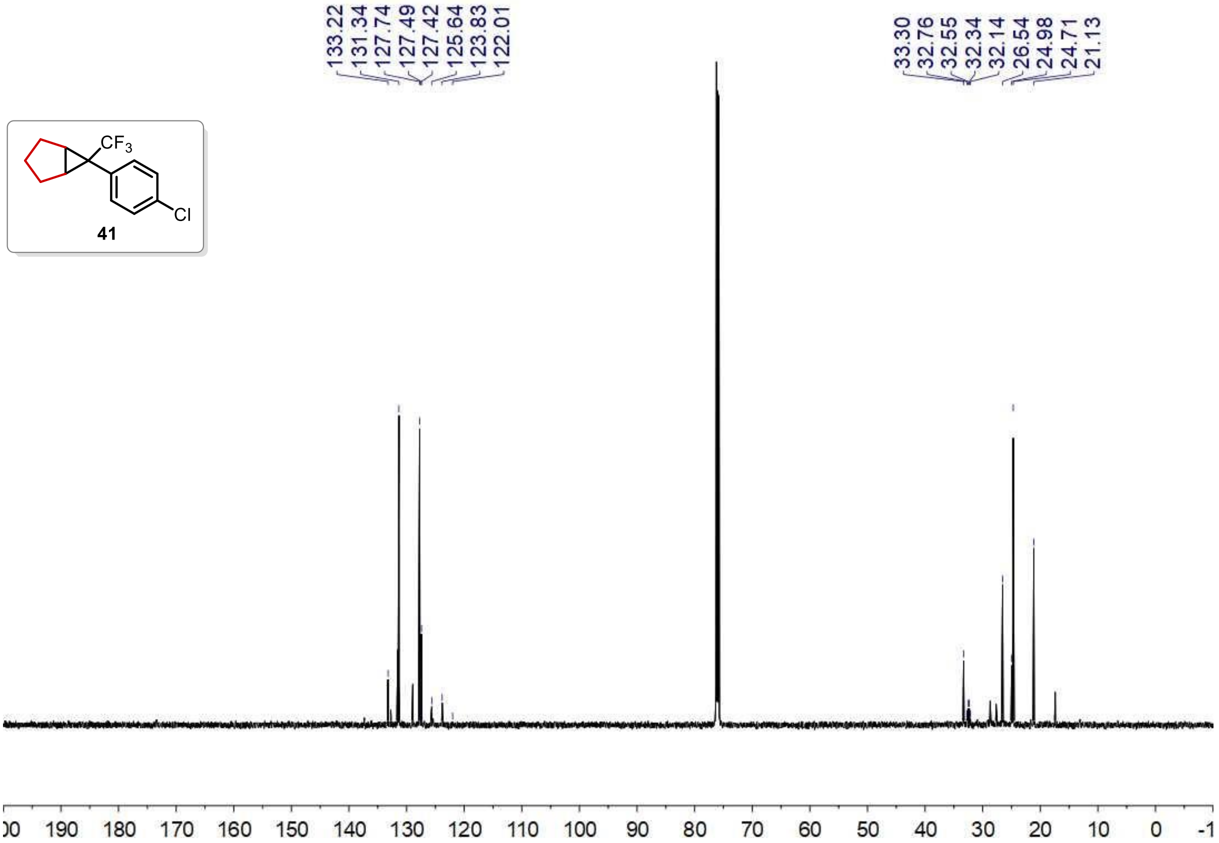

Supplementary Fig. 118 ^{13}**C NMR** (150 MHz, $CDCl_3$) spectrum of compound **41**.

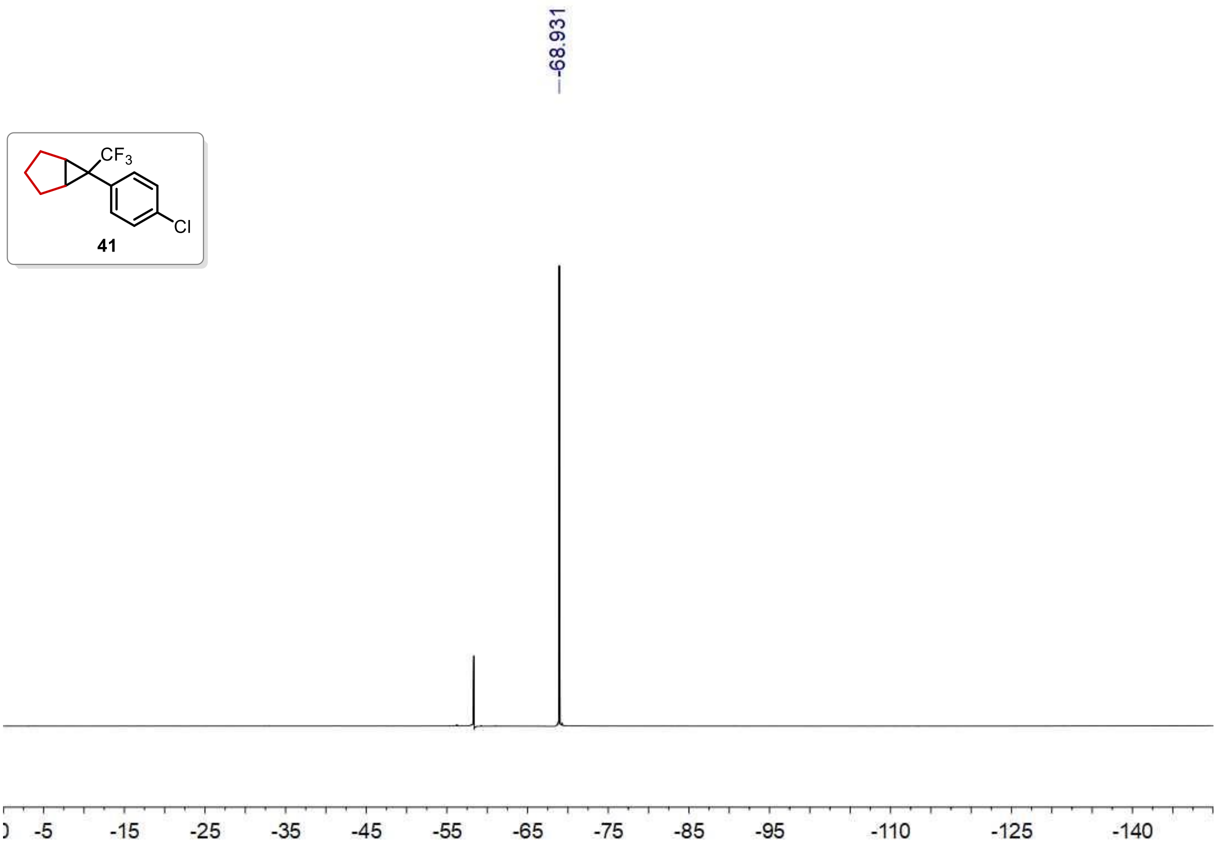

Supplementary Fig. 119 ^{19}**F NMR** (564 MHz, $CDCl_3$) spectrum of compound **41**.

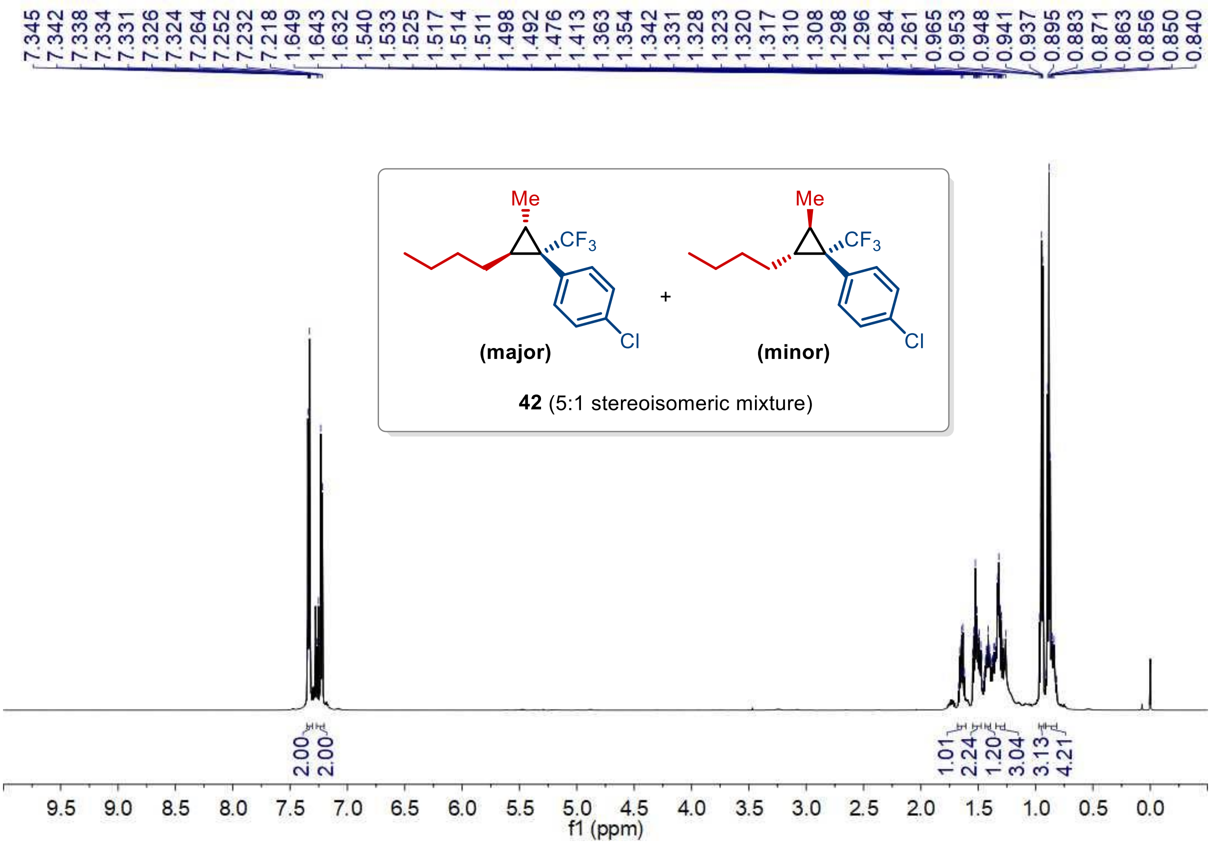

Supplementary Fig. 120 ^{1}**H NMR** (500 MHz, $CDCl_3$) spectrum of compound **42**.

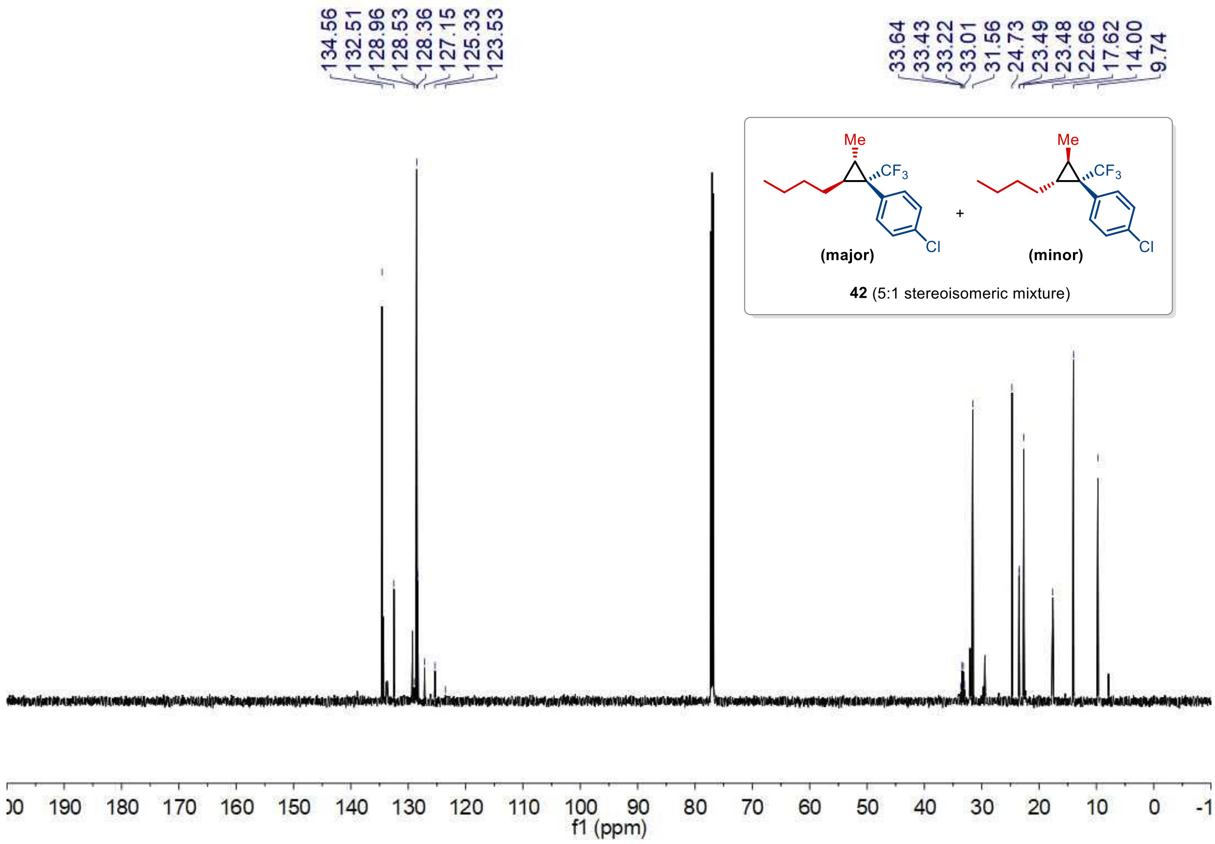

Supplementary Fig. 121 ^{13}**C NMR** (150 MHz, $CDCl_3$) spectrum of compound **42**.

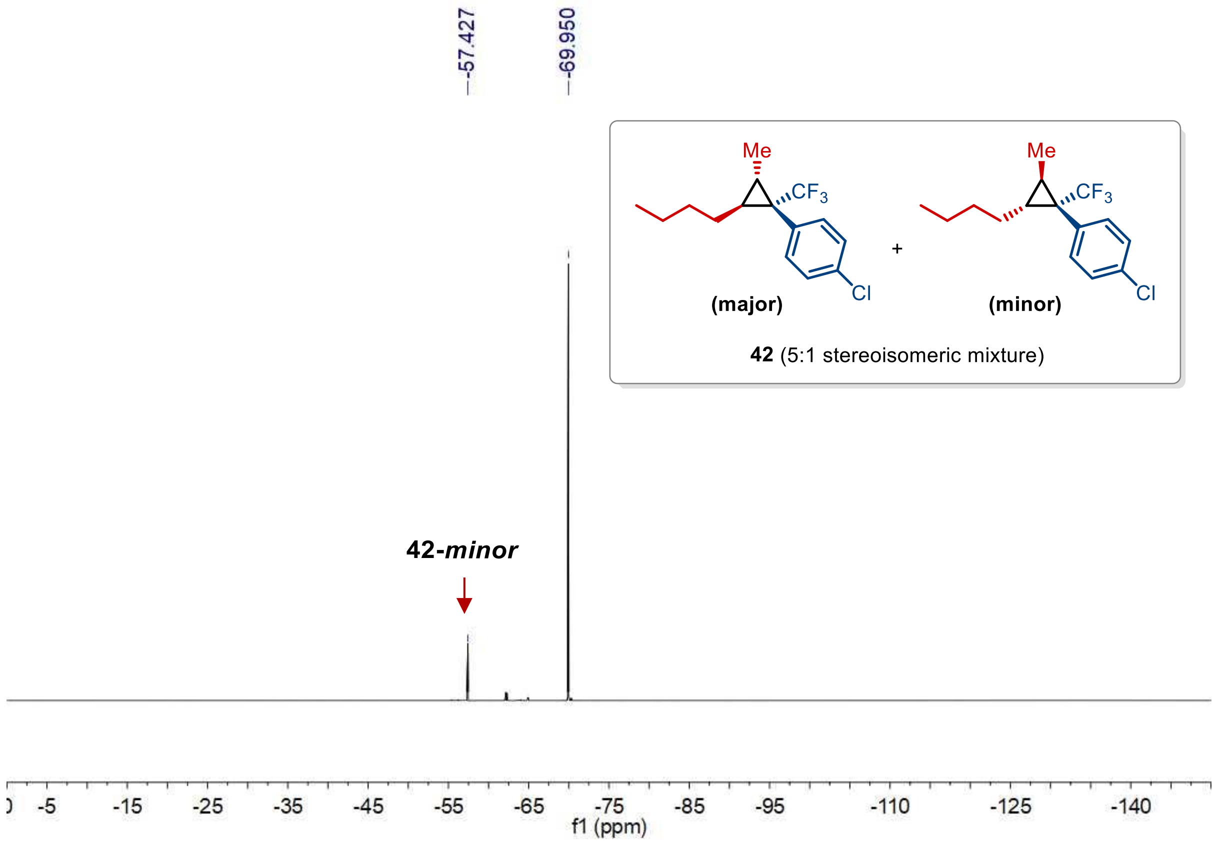

Supplementary Fig. 122 ^{19}**F NMR** (564 MHz, $CDCl_3$) spectrum of compound **42**.

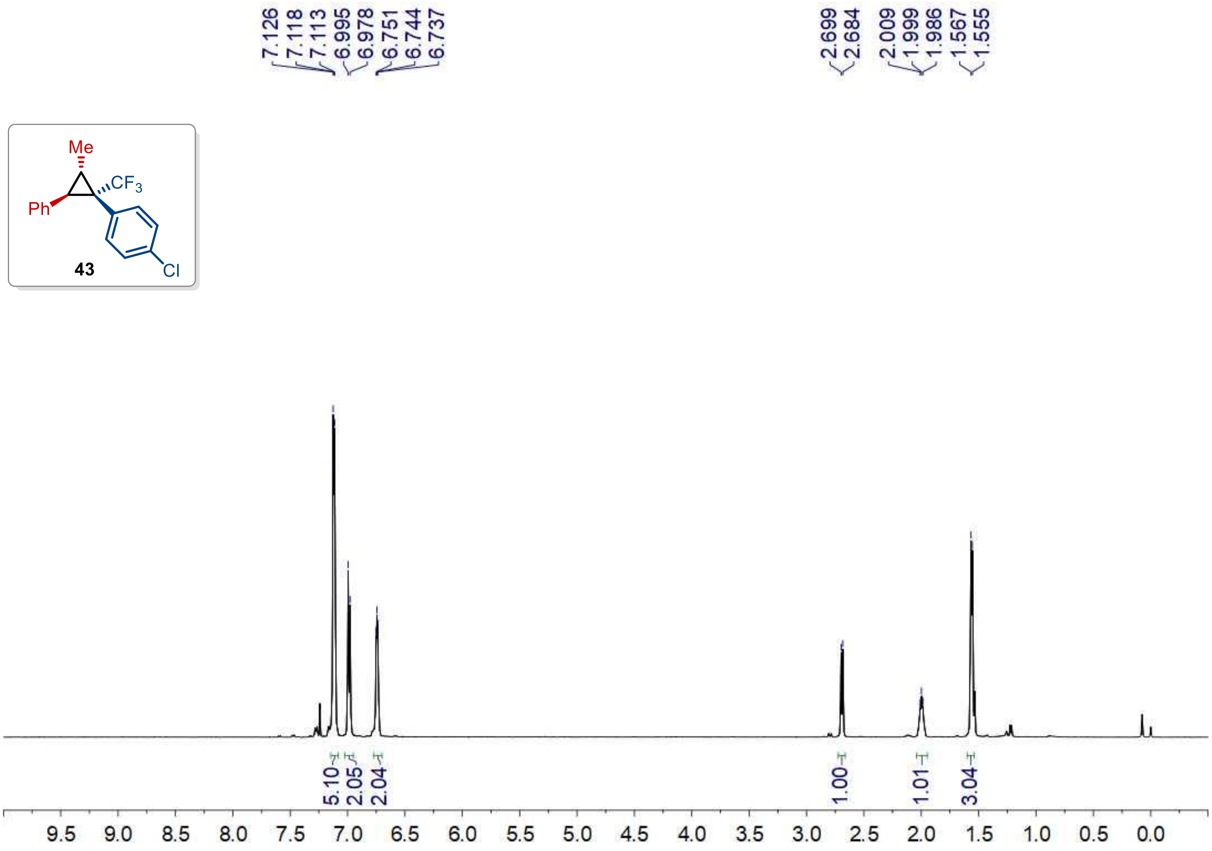

Supplementary Fig. 123 ^{1}H NMR (500 MHz, $CDCl_3$) spectrum of compound **43**.

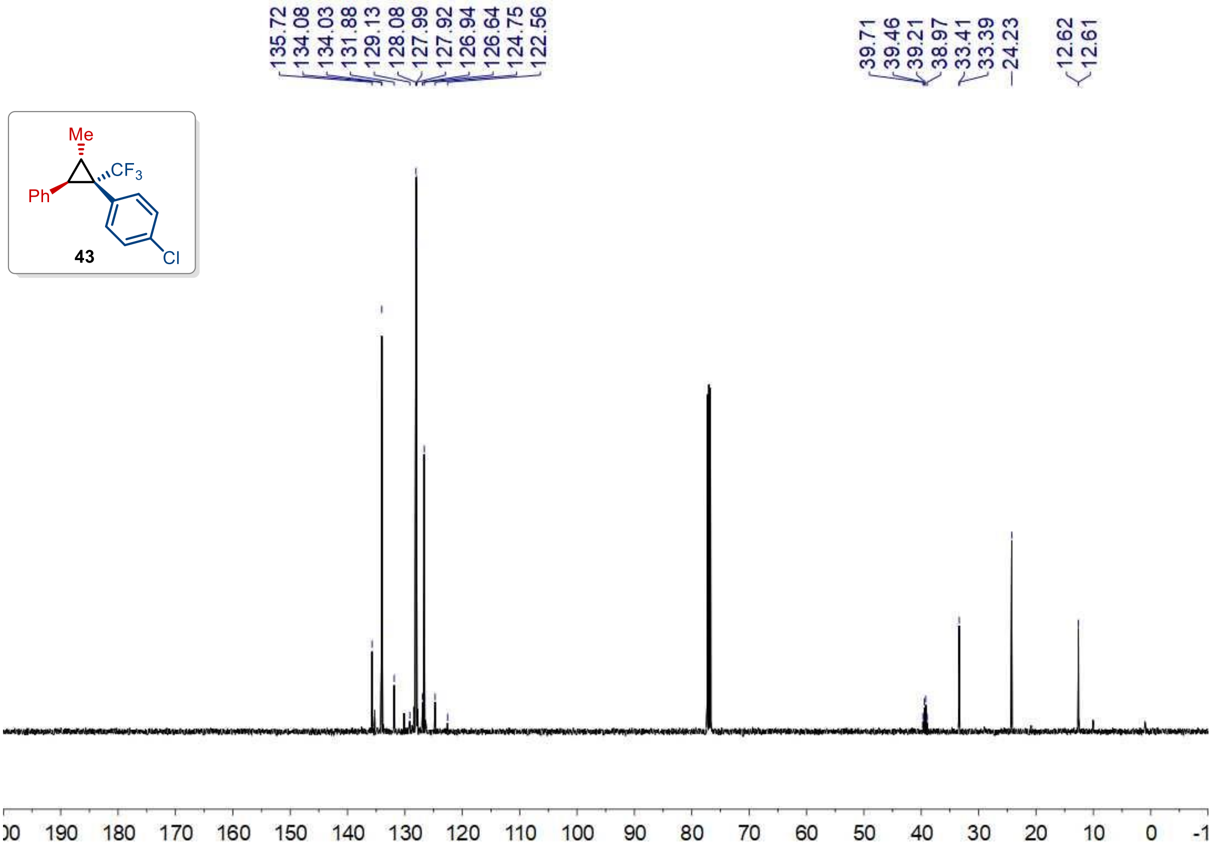

Supplementary Fig. 124 ^{13}C NMR (125 MHz, $CDCl_3$) spectrum of compound **43**.

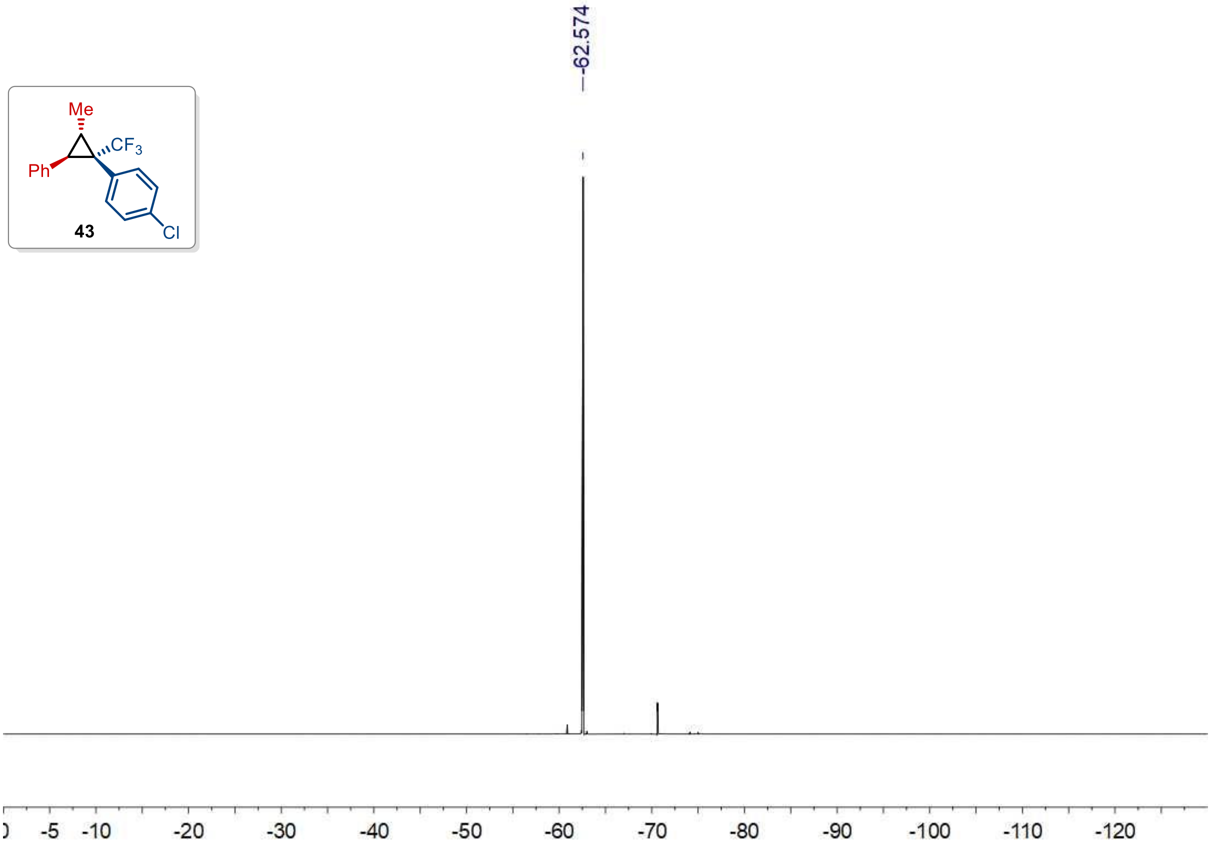

Supplementary Fig. 125 ^{19}**F NMR** (470 MHz, $CDCl_3$) spectrum of compound **43**.

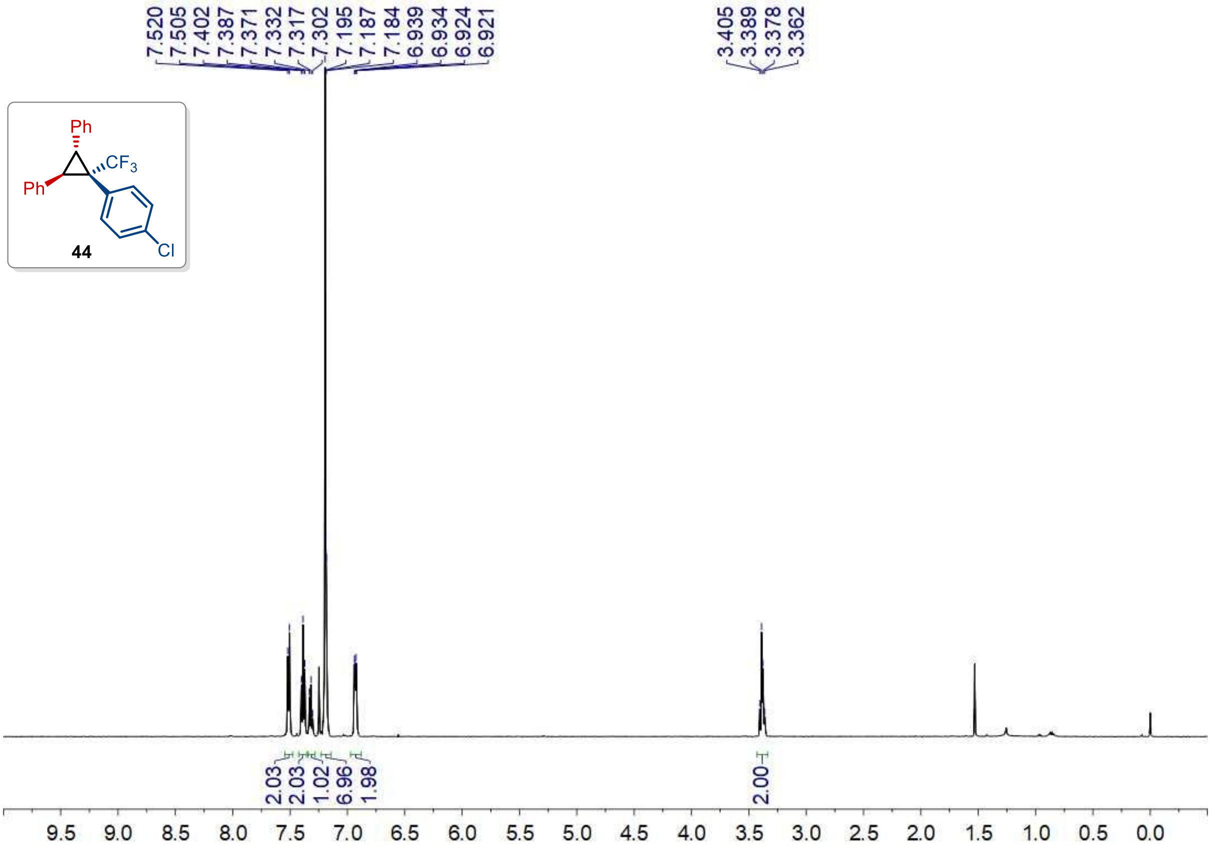

Supplementary Fig. 126 ^{1}**H NMR** (500 MHz, $CDCl_3$) spectrum of compound **44**.

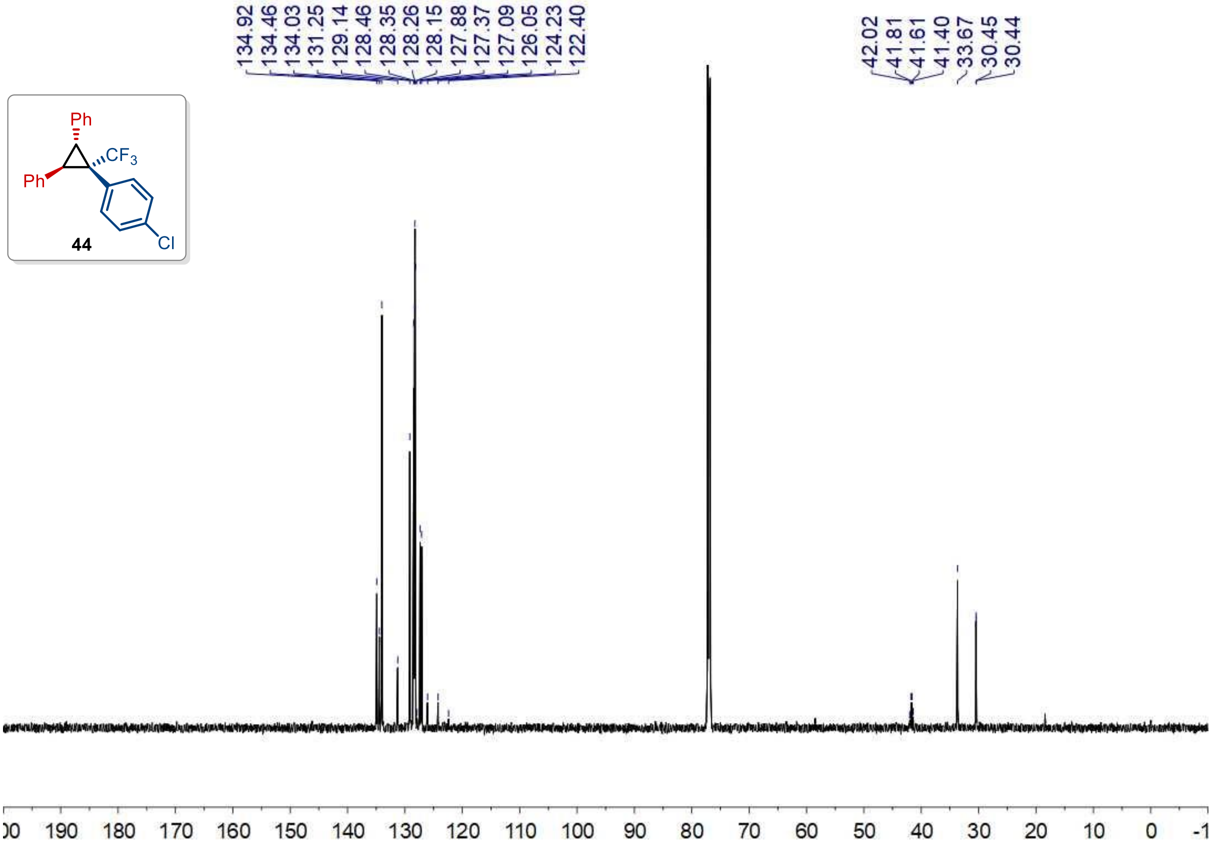

Supplementary Fig. 127 ^{13}**C NMR** (150 MHz, $CDCl_3$) spectrum of compound **44**.

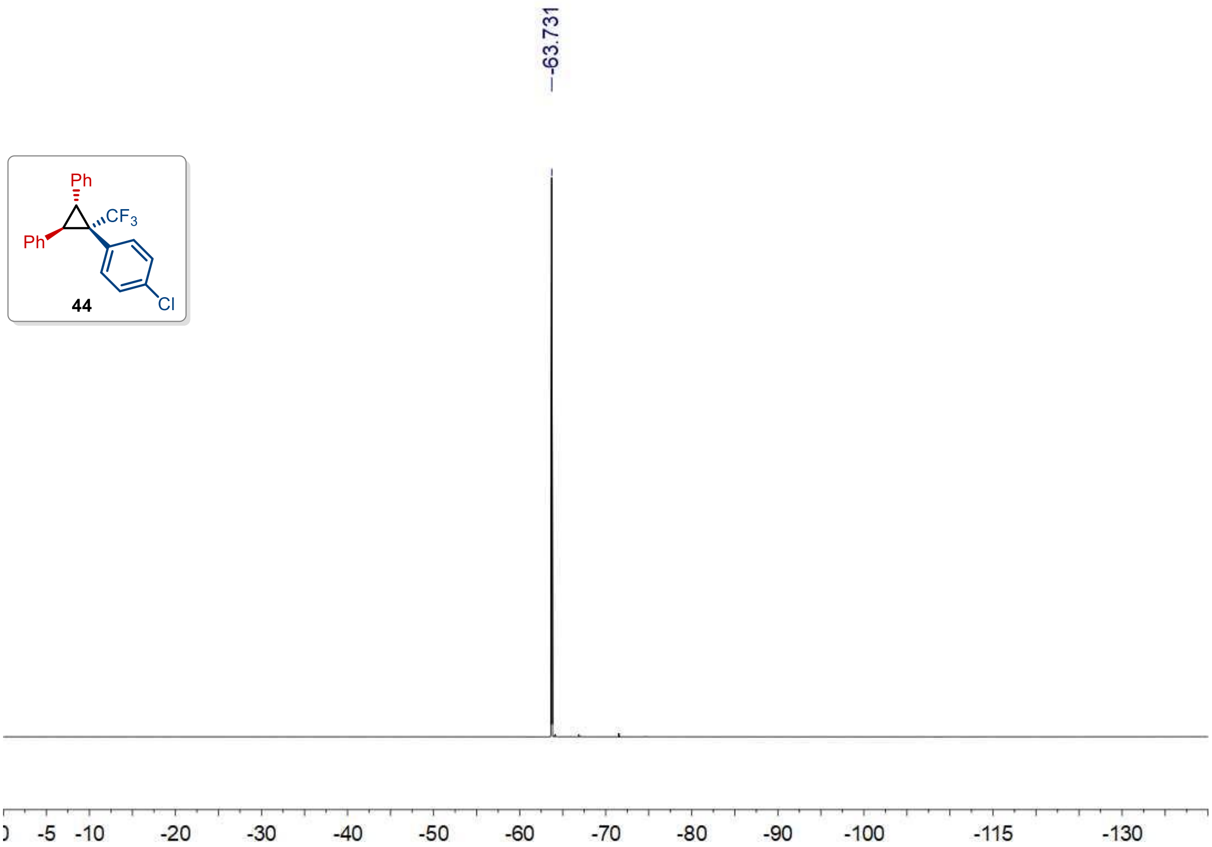

Supplementary Fig. 128 ^{19}**F NMR** (564 MHz, $CDCl_3$) spectrum of compound **44**.

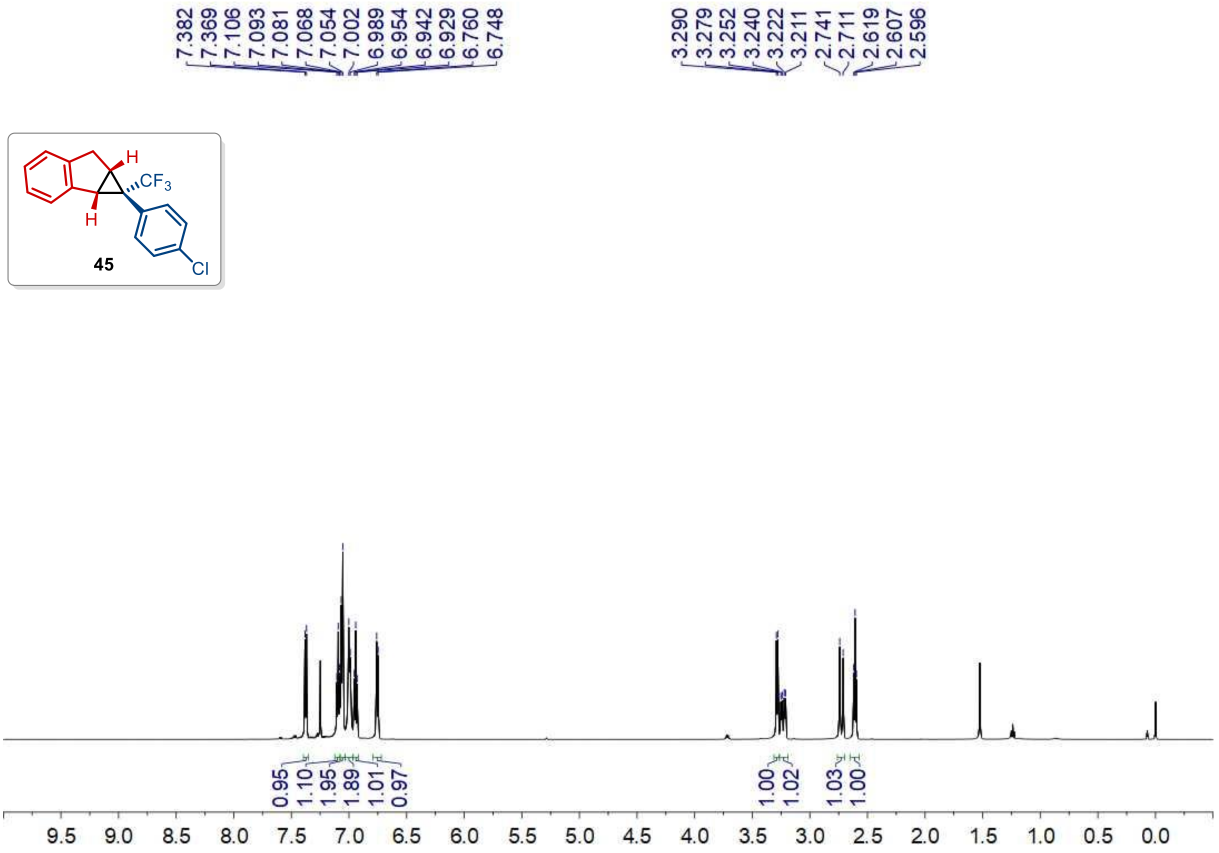

Supplementary Fig. 129 ^{1}H NMR (500 MHz, $CDCl_3$) spectrum of compound **45**.

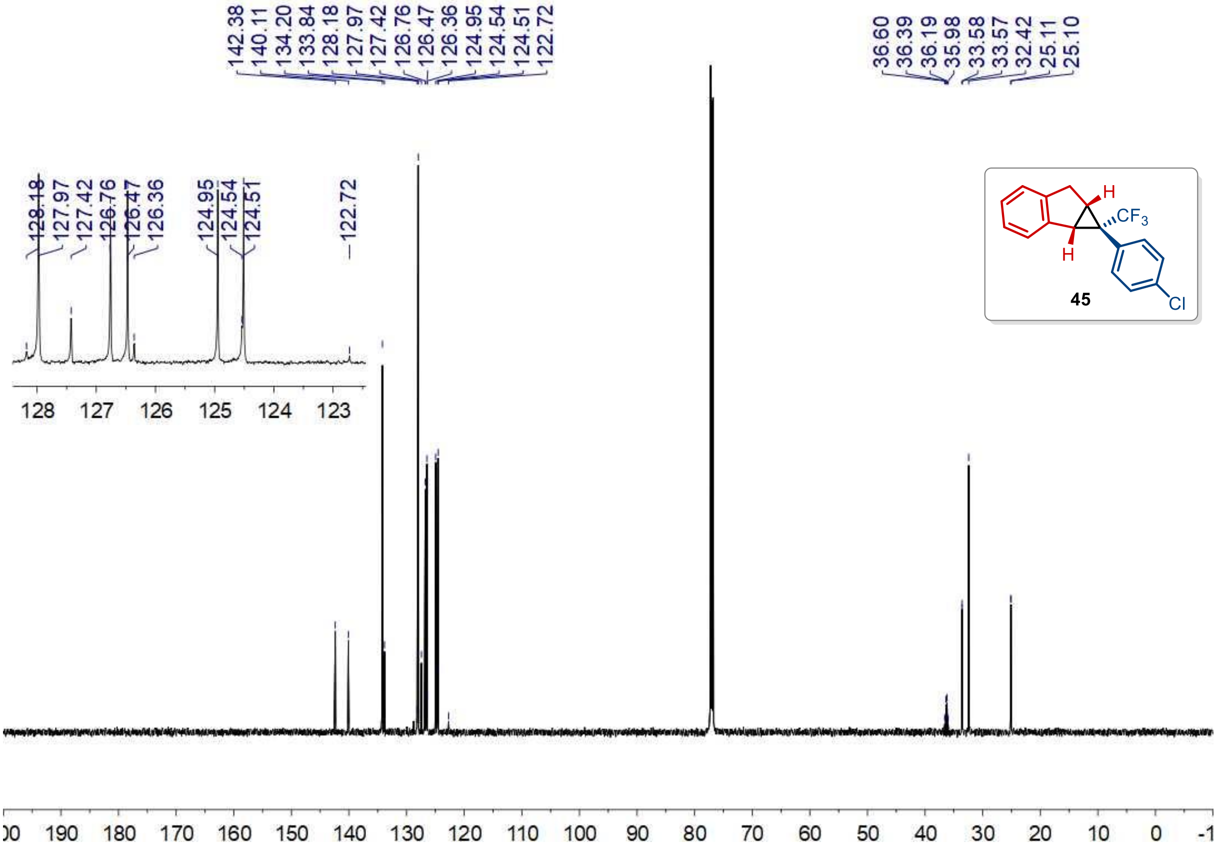

Supplementary Fig. 130 ^{13}C NMR (150 MHz, $CDCl_3$) spectrum of compound **45**.

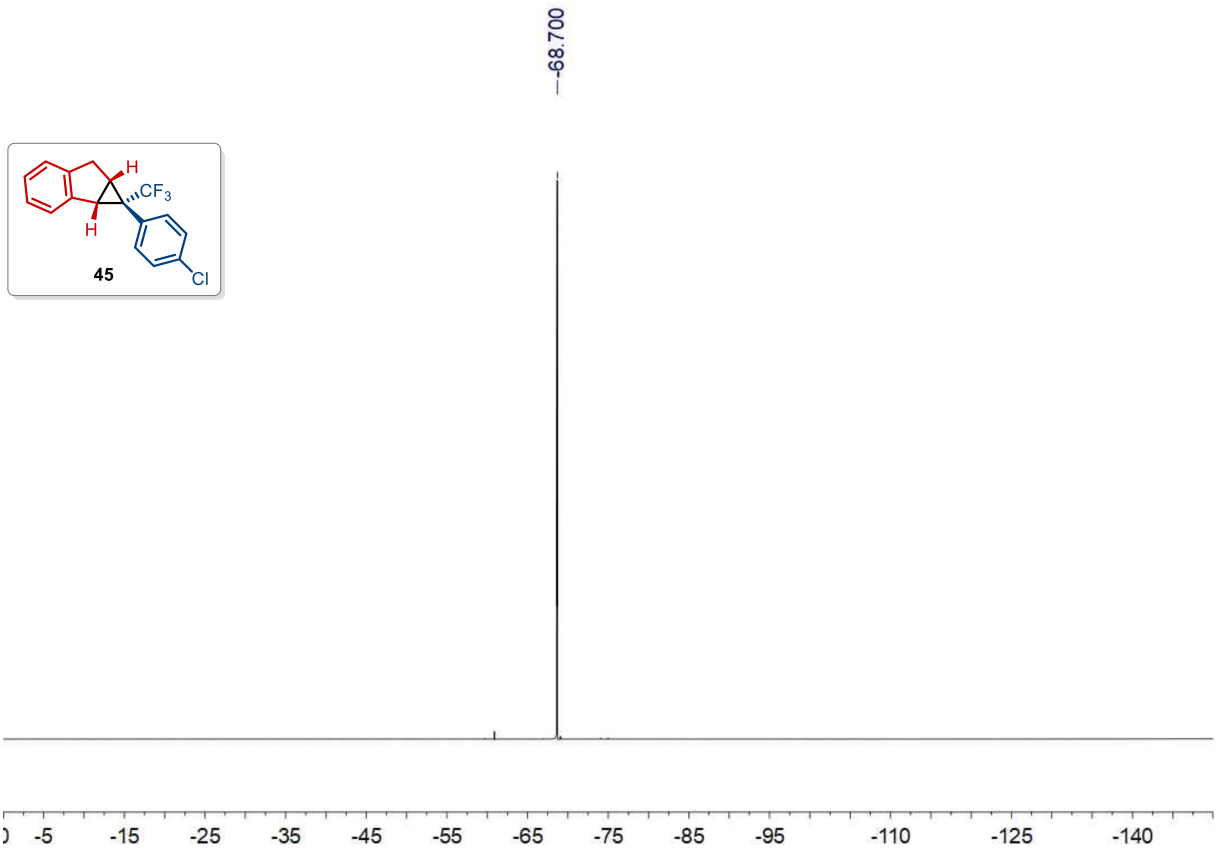

Supplementary Fig. 131 ^{19}**F NMR** (564 MHz, $CDCl_3$) spectrum of compound **45**.

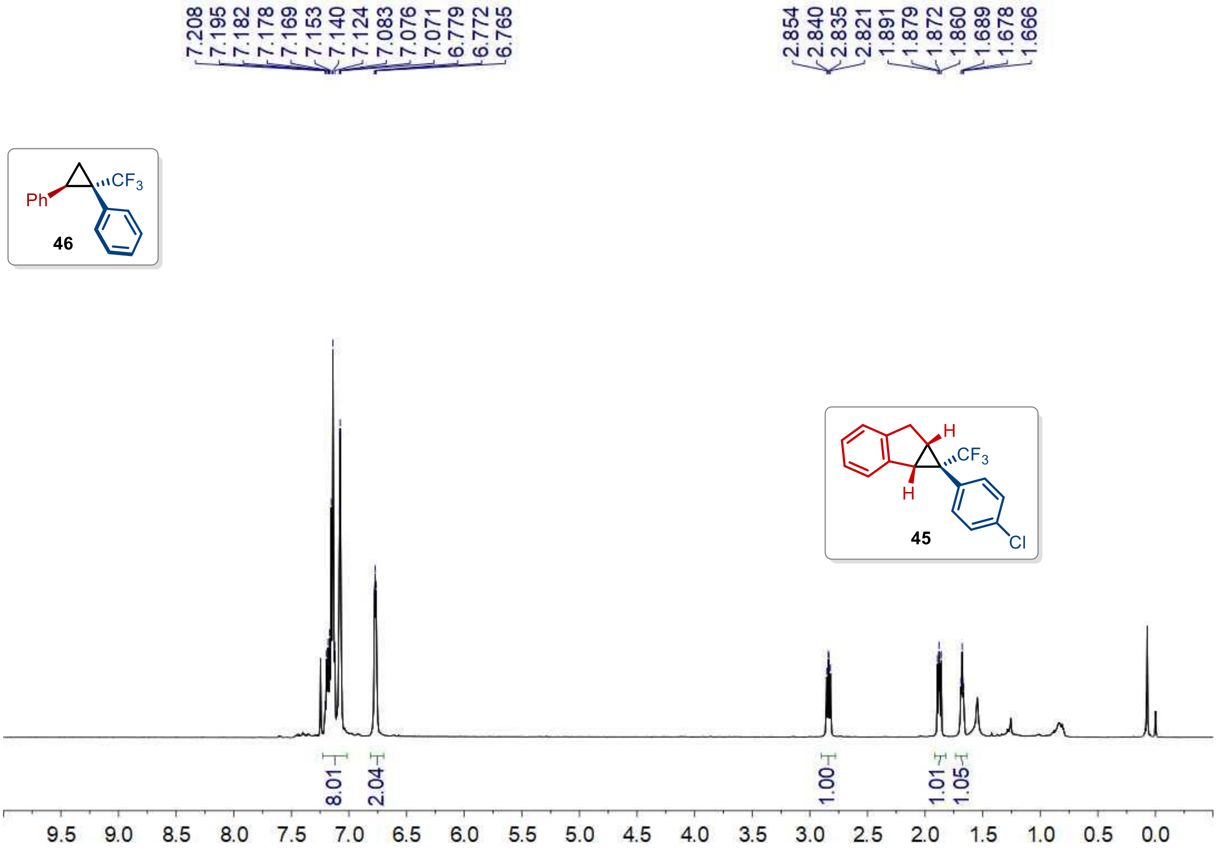

Supplementary Fig. 132 ^{1}**H NMR** (500 MHz, $CDCl_3$) spectrum of compound **46**.

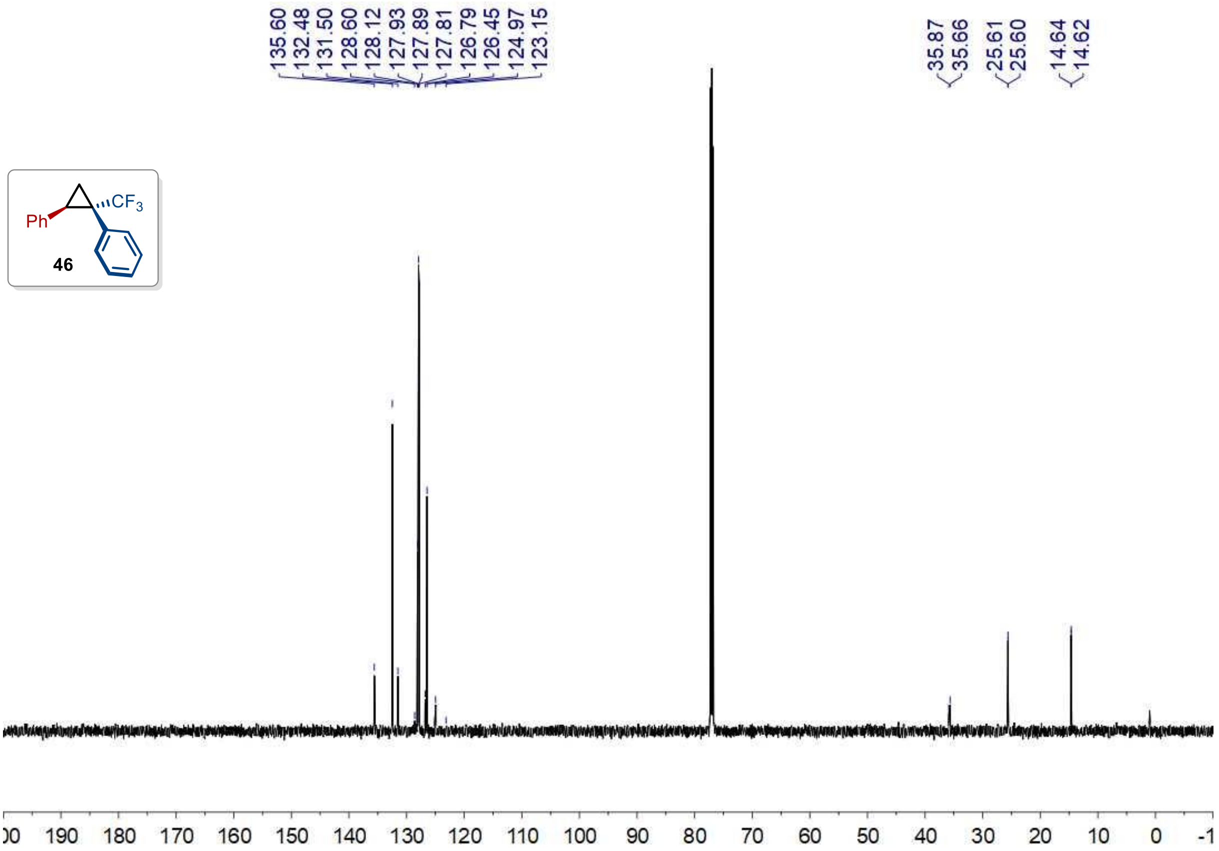

Supplementary Fig. 133 ^{13}**C NMR** (150 MHz, $CDCl_3$) spectrum of compound **46**.

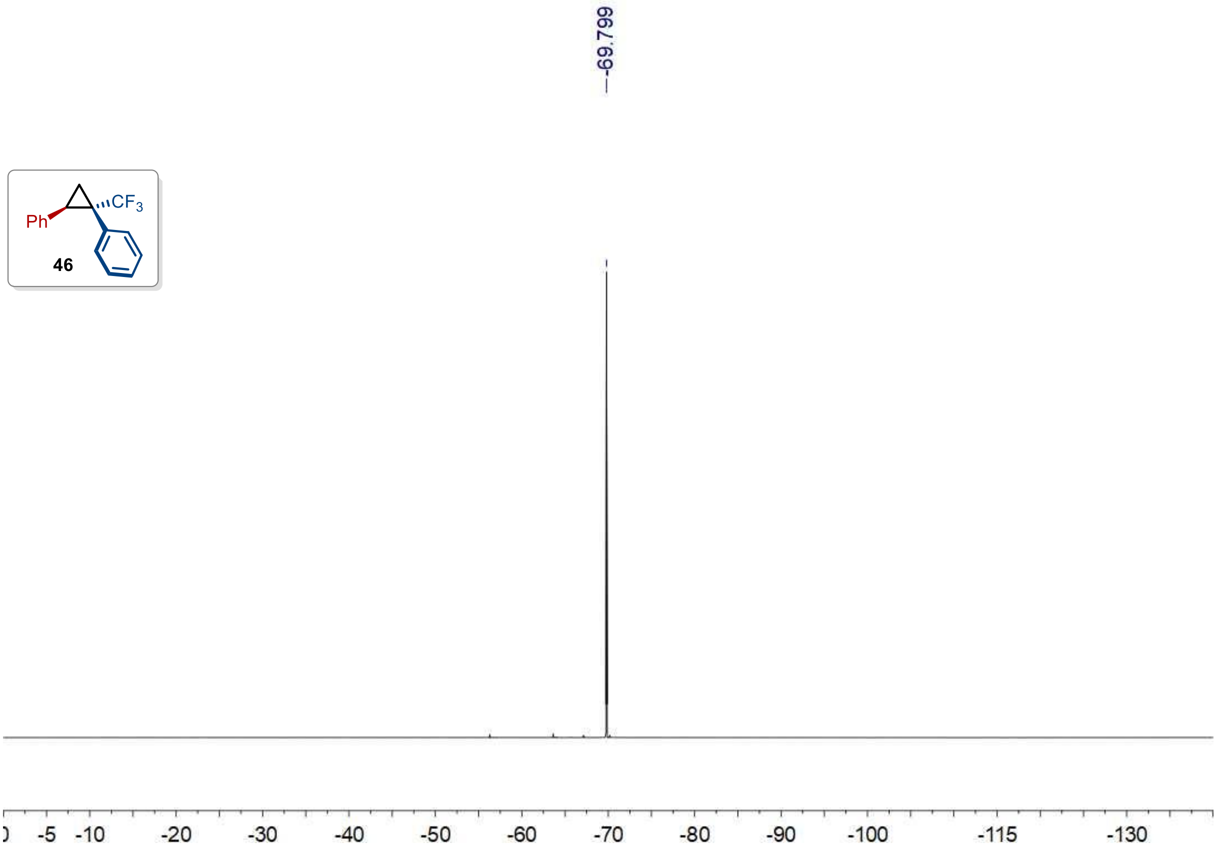

Supplementary Fig. 134 ^{19}**F NMR** (564 MHz, $CDCl_3$) spectrum of compound **46**.

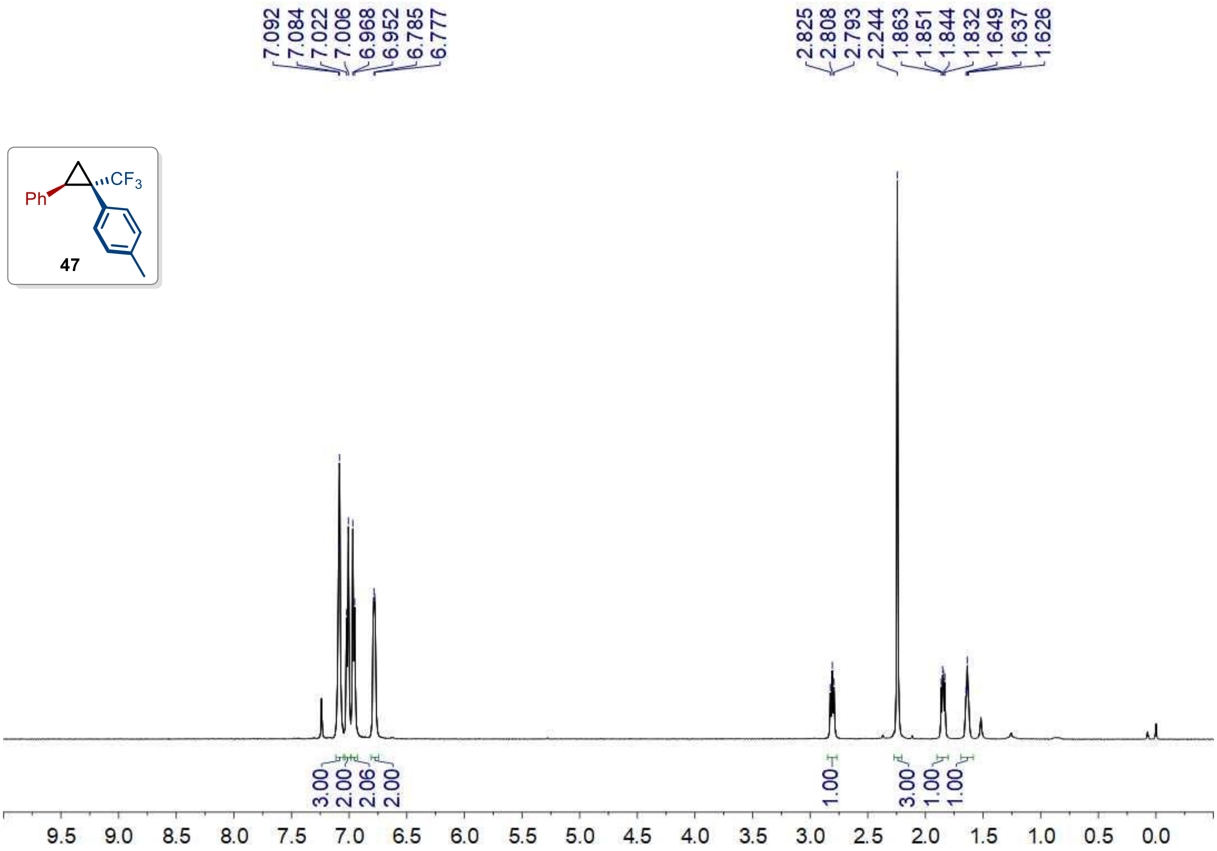

Supplementary Fig. 135 ^{1}**H NMR** (500 MHz, $CDCl_3$) spectrum of compound **47**.

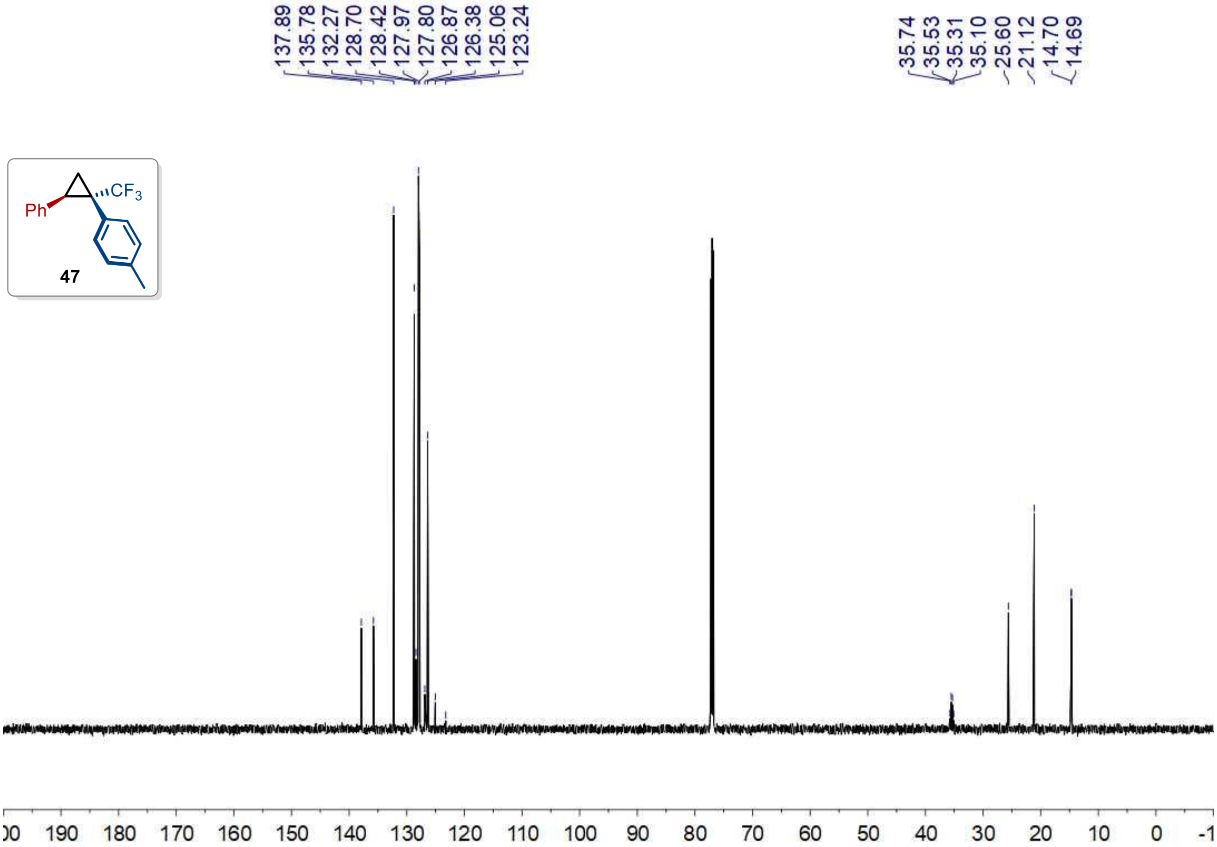

Supplementary Fig. 136 ^{13}**C NMR** (150 MHz, $CDCl_3$) spectrum of compound **47**.

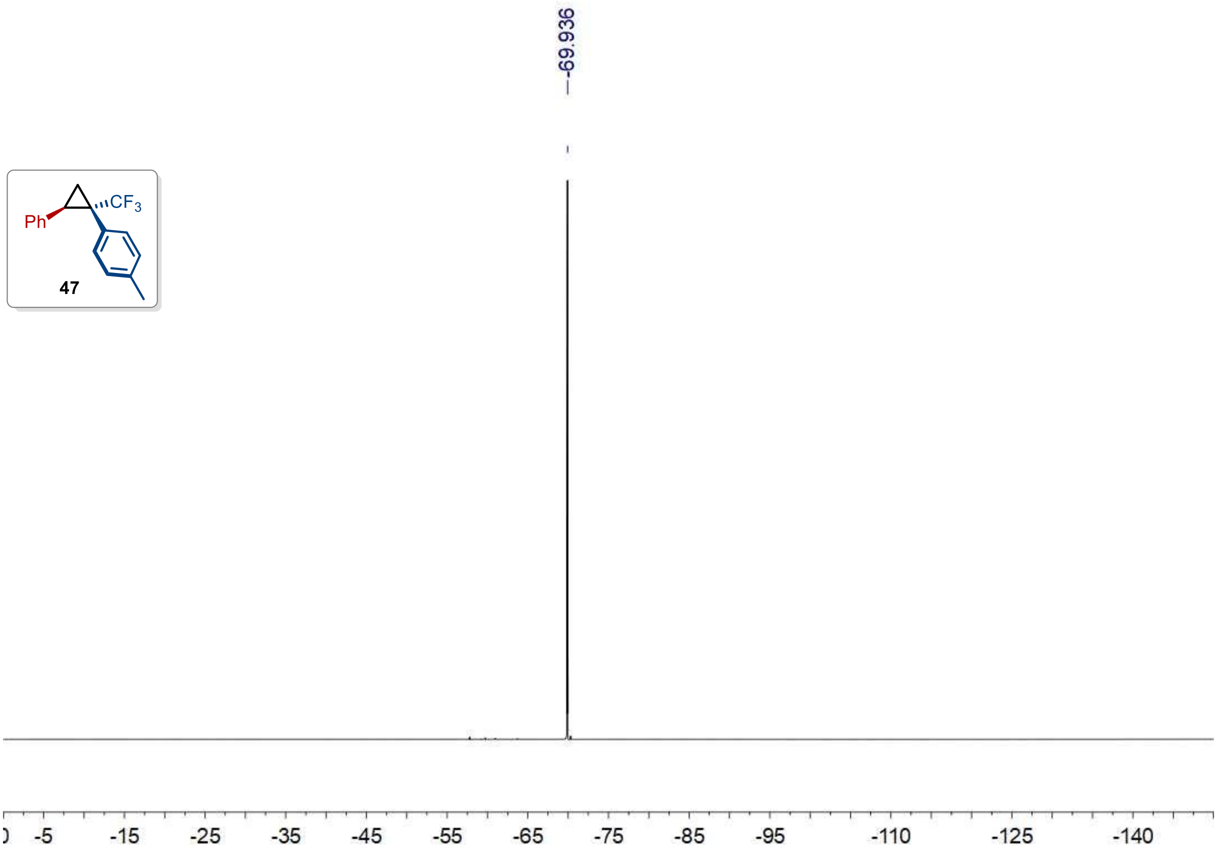

Supplementary Fig. 137 ^{19}F NMR (564 MHz, $CDCl_3$) spectrum of compound **47**.

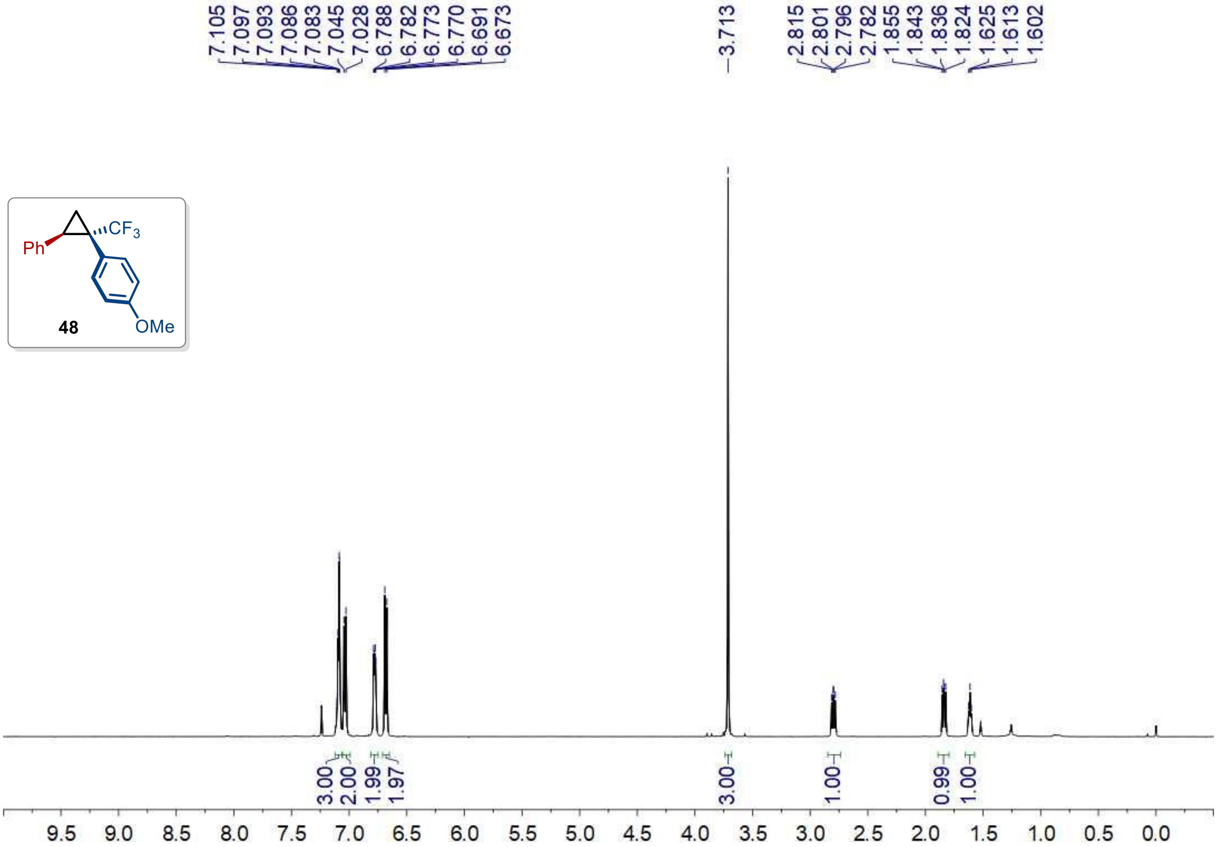

Supplementary Fig. 138 ^{1}H NMR (500 MHz, $CDCl_3$) spectrum of compound **48**.

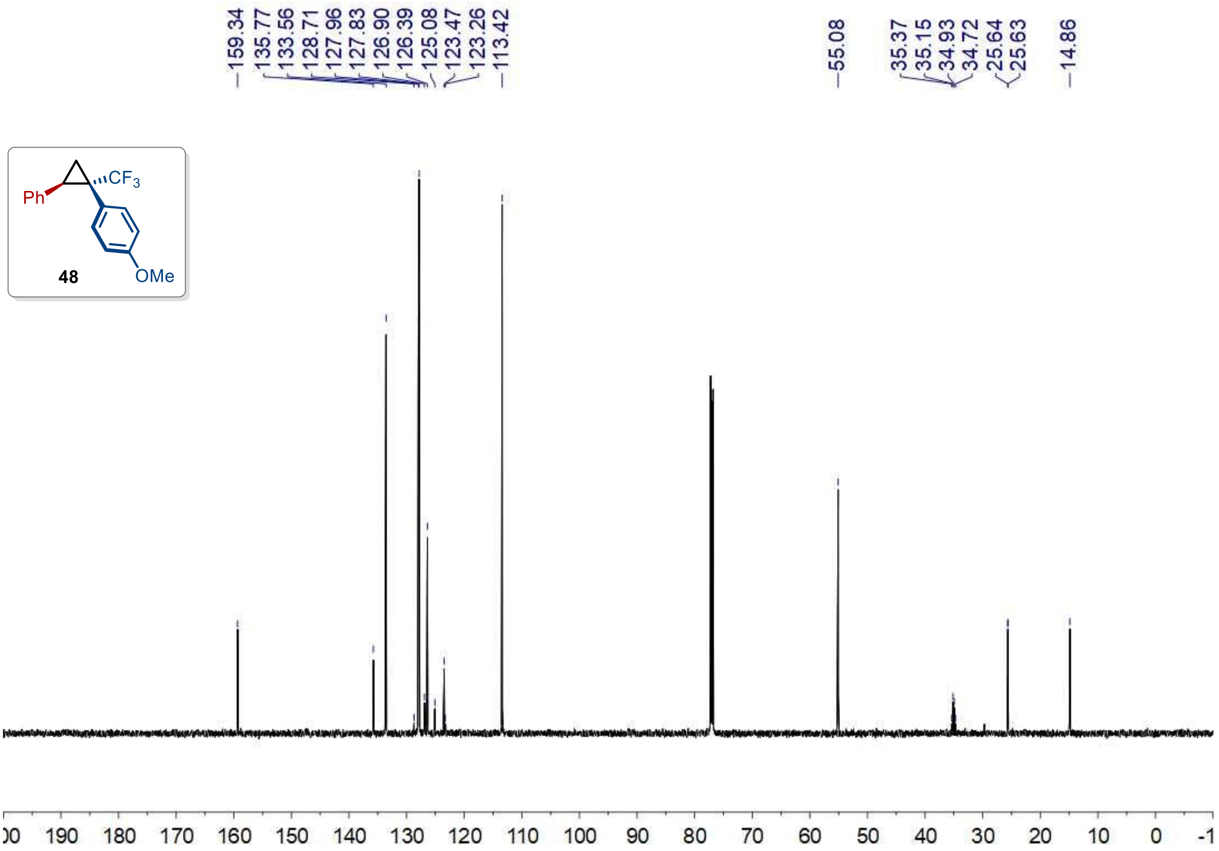

Supplementary Fig. 139 ^{13}**C NMR** (150 MHz, $CDCl_3$) spectrum of compound **48**.

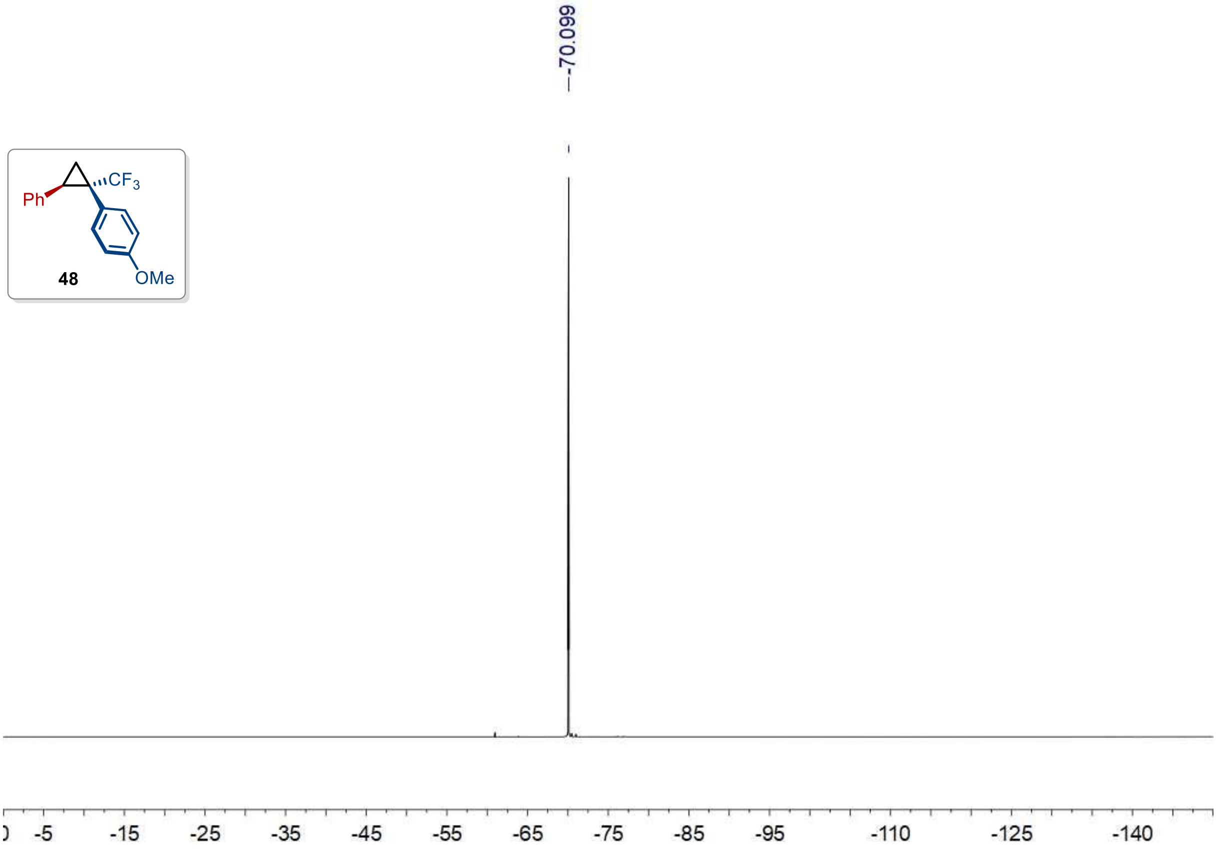

Supplementary Fig. 140 ^{19}**F NMR** (564 MHz, $CDCl_3$) spectrum of compound **48**.

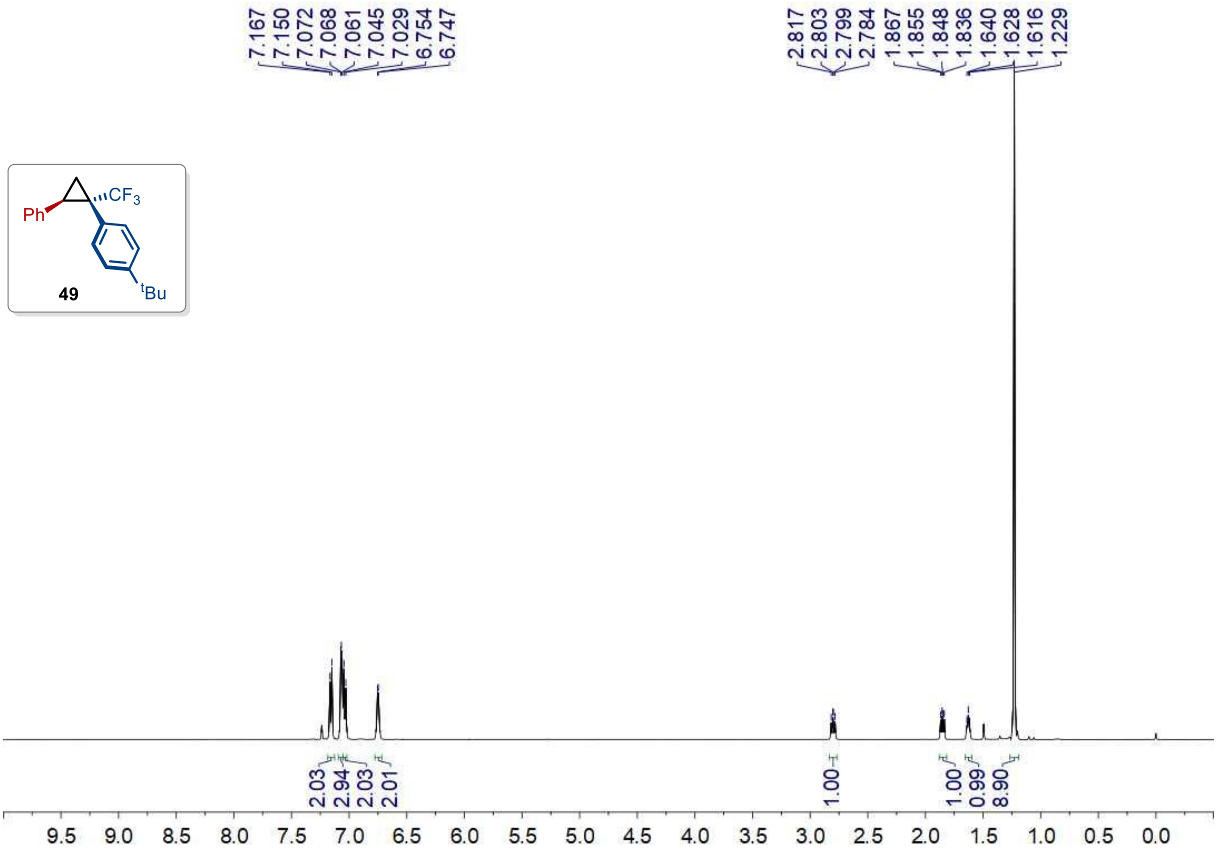

Supplementary Fig. 141 ^{1}**H NMR** (500 MHz, $CDCl_3$) spectrum of compound **49**.

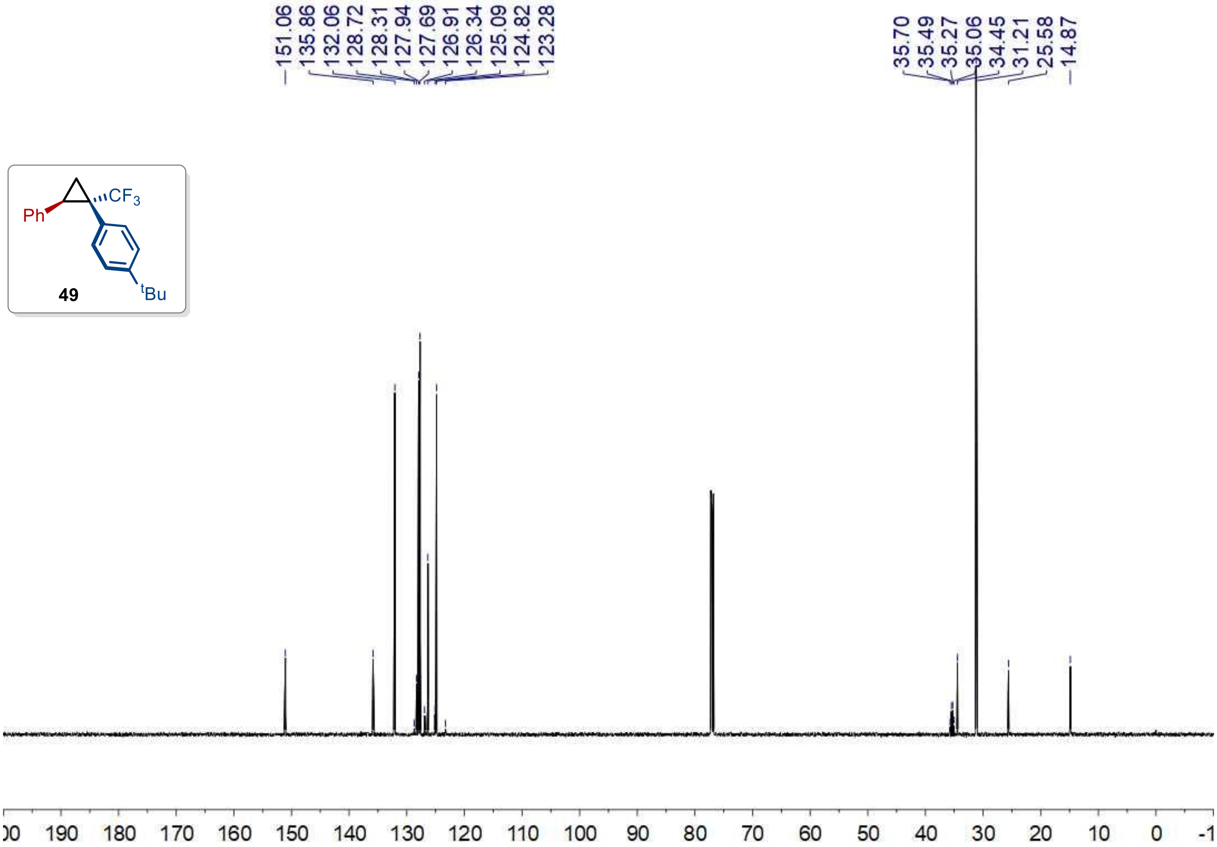

Supplementary Fig. 142 ^{13}**C NMR** (150 MHz, $CDCl_3$) spectrum of compound **49**.

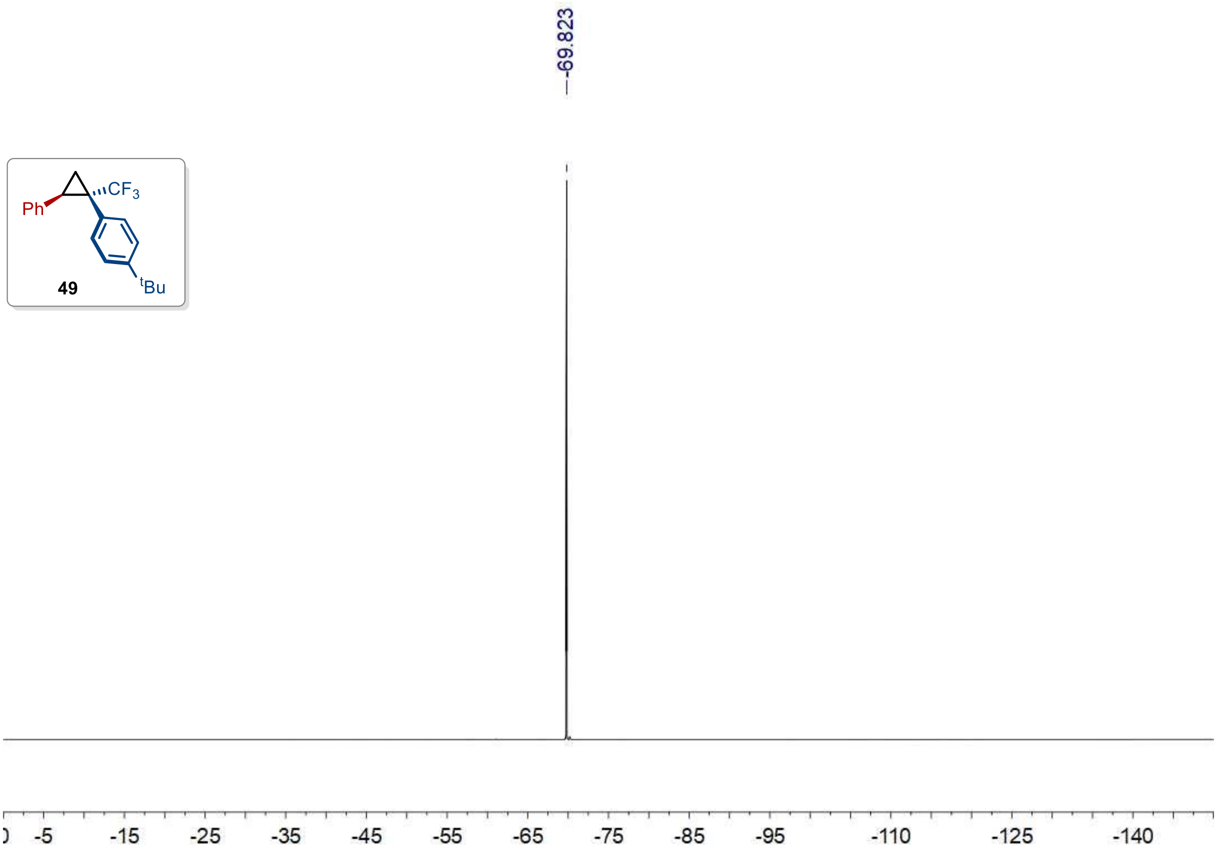

Supplementary Fig. 143 ^{19}F NMR (564 MHz, $CDCl_3$) spectrum of compound **49**.

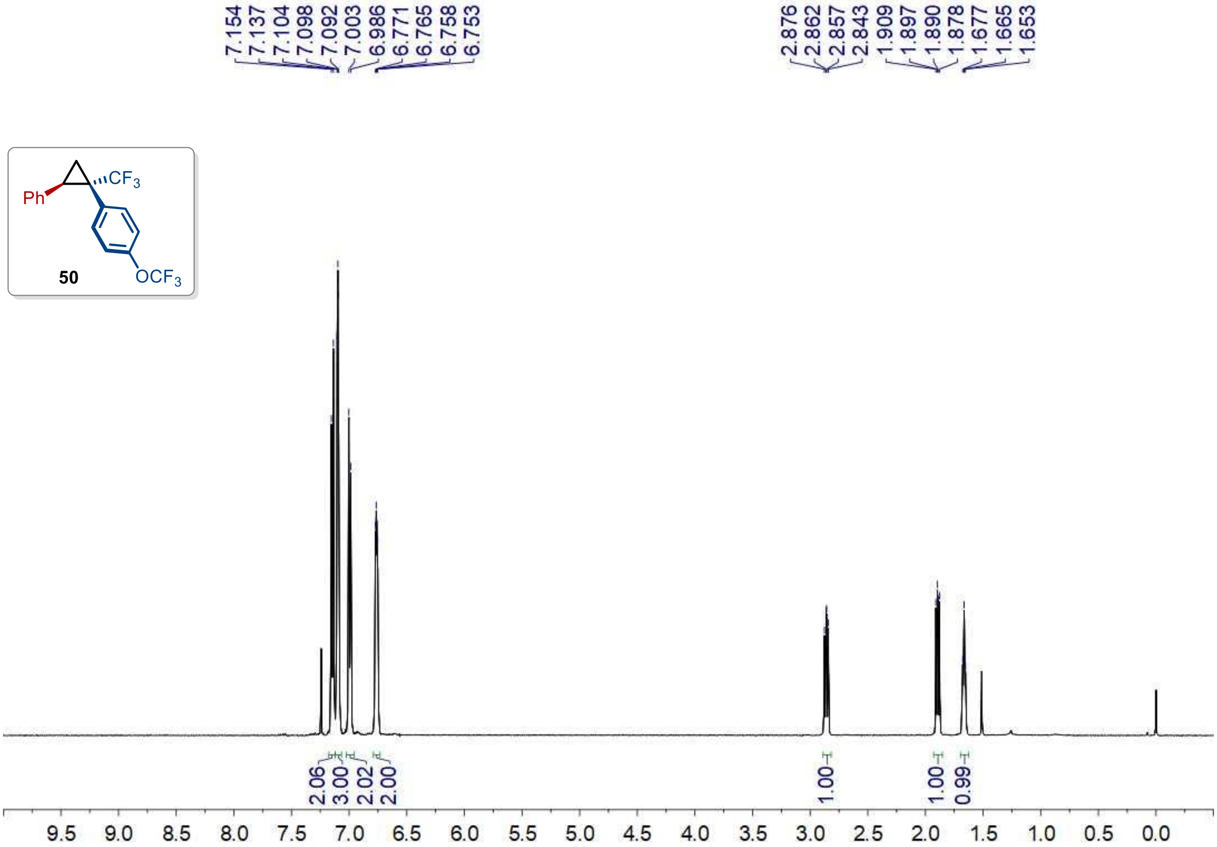

Supplementary Fig. 144 ^{1}H NMR (500 MHz, $CDCl_3$) spectrum of compound **50**.

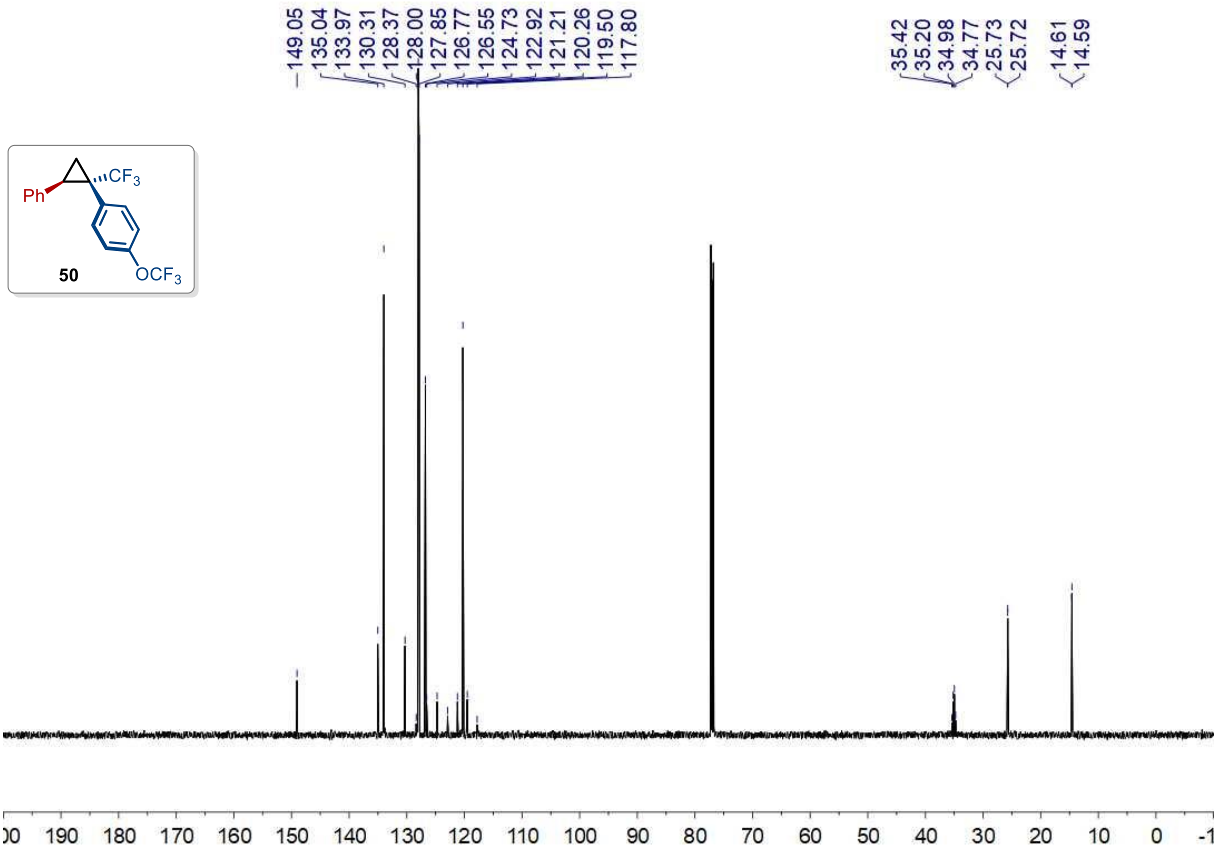

Supplementary Fig. 145 ^{13}**C NMR** (150 MHz, $CDCl_3$) spectrum of compound **50**.

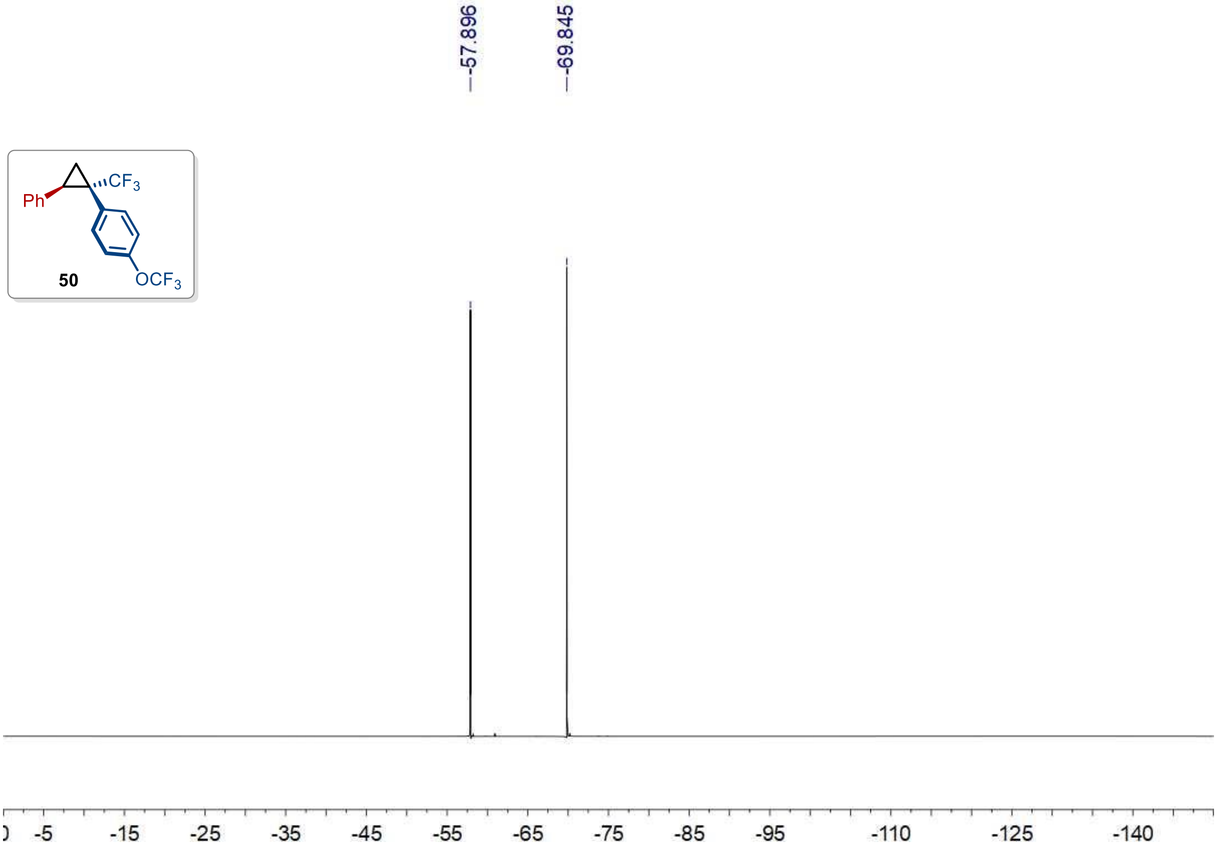

Supplementary Fig. 146 ^{19}**F NMR** (564 MHz, $CDCl_3$) spectrum of compound **50**.

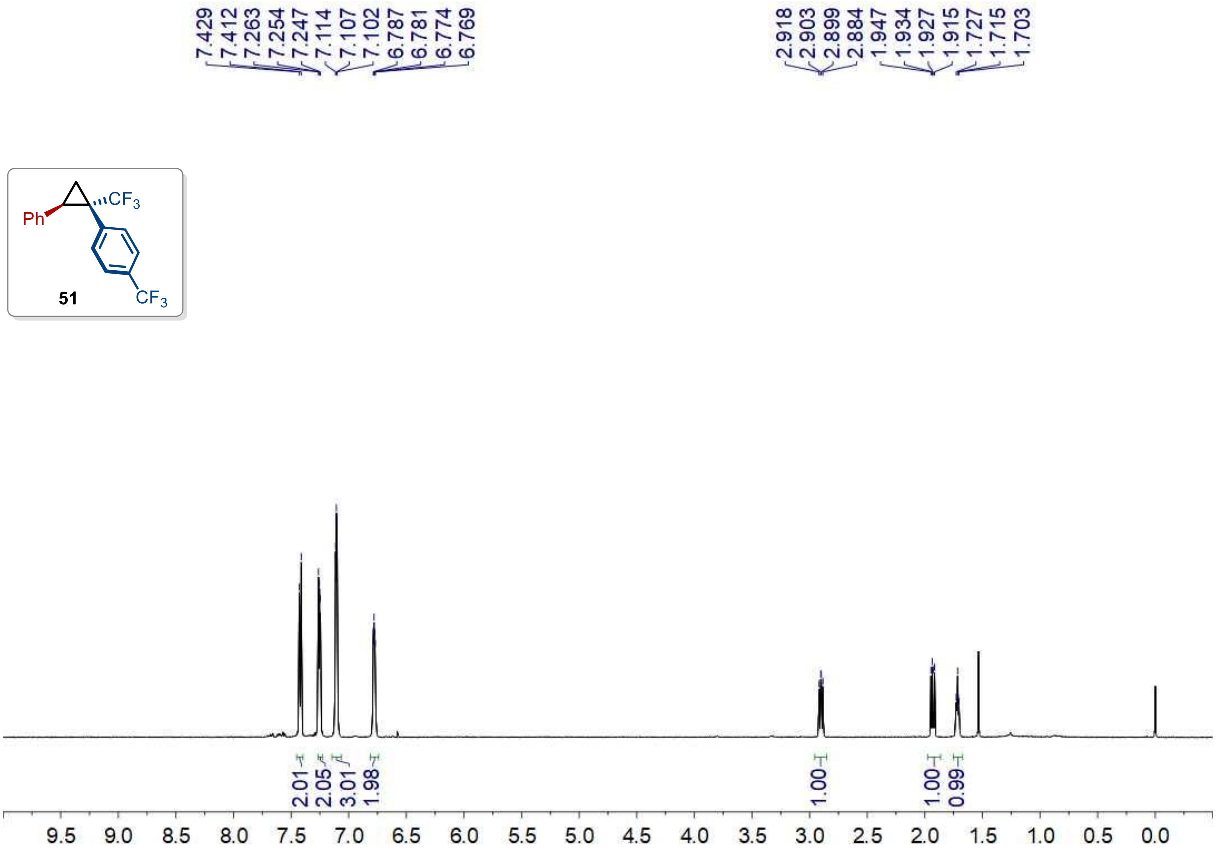

Supplementary Fig. 147 ^{1}H NMR (500 MHz, $CDCl_3$) spectrum of compound **51**.

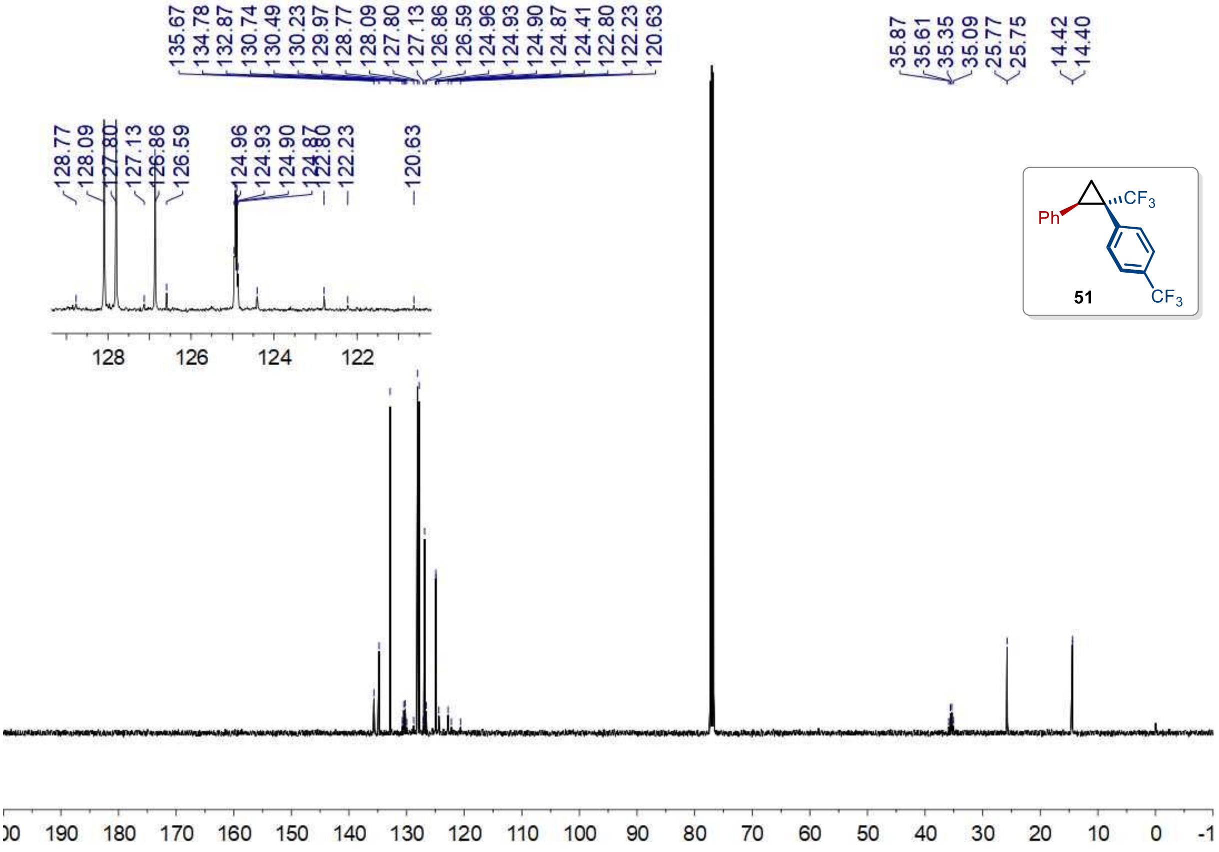

Supplementary Fig. 148 ^{13}C NMR (125 MHz, $CDCl_3$) spectrum of compound **51**.

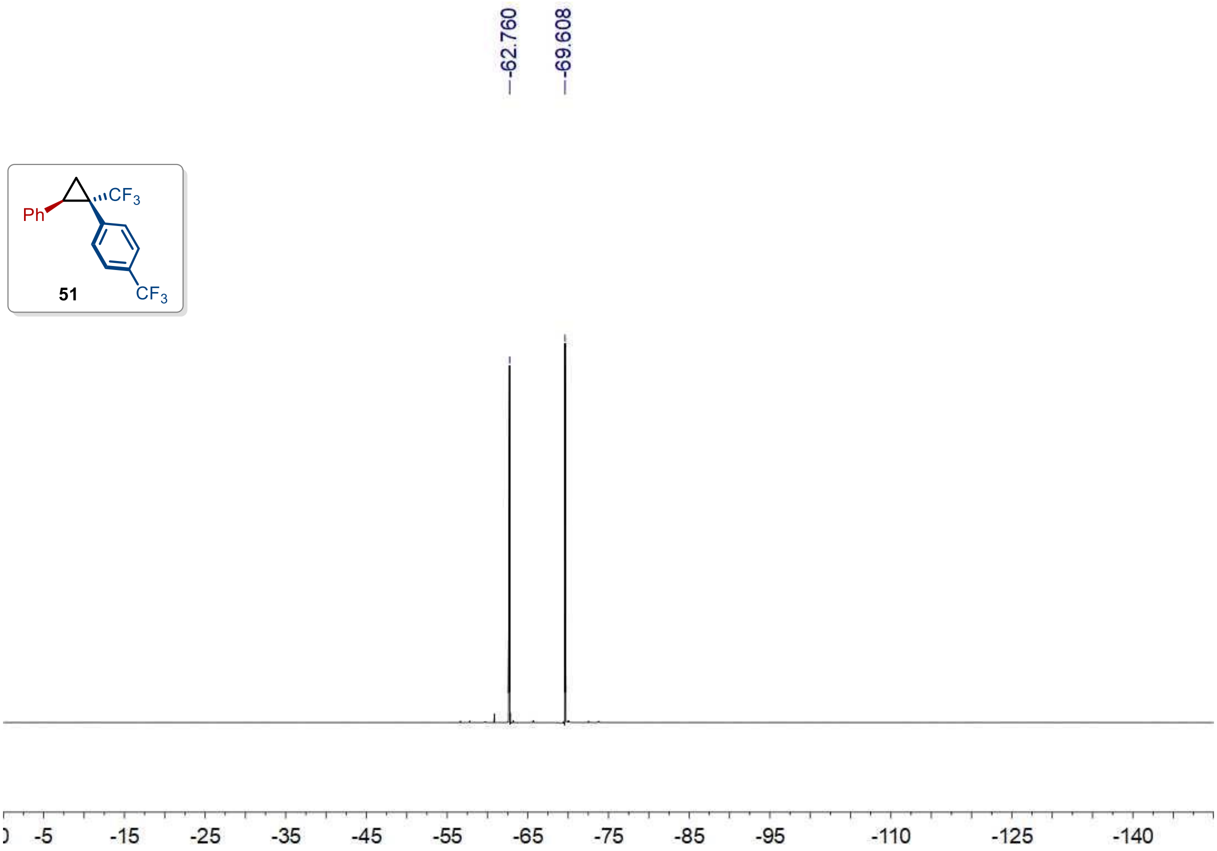

Supplementary Fig. 149 ^{19}**F NMR** (564 MHz, $CDCl_3$) spectrum of compound **51**.

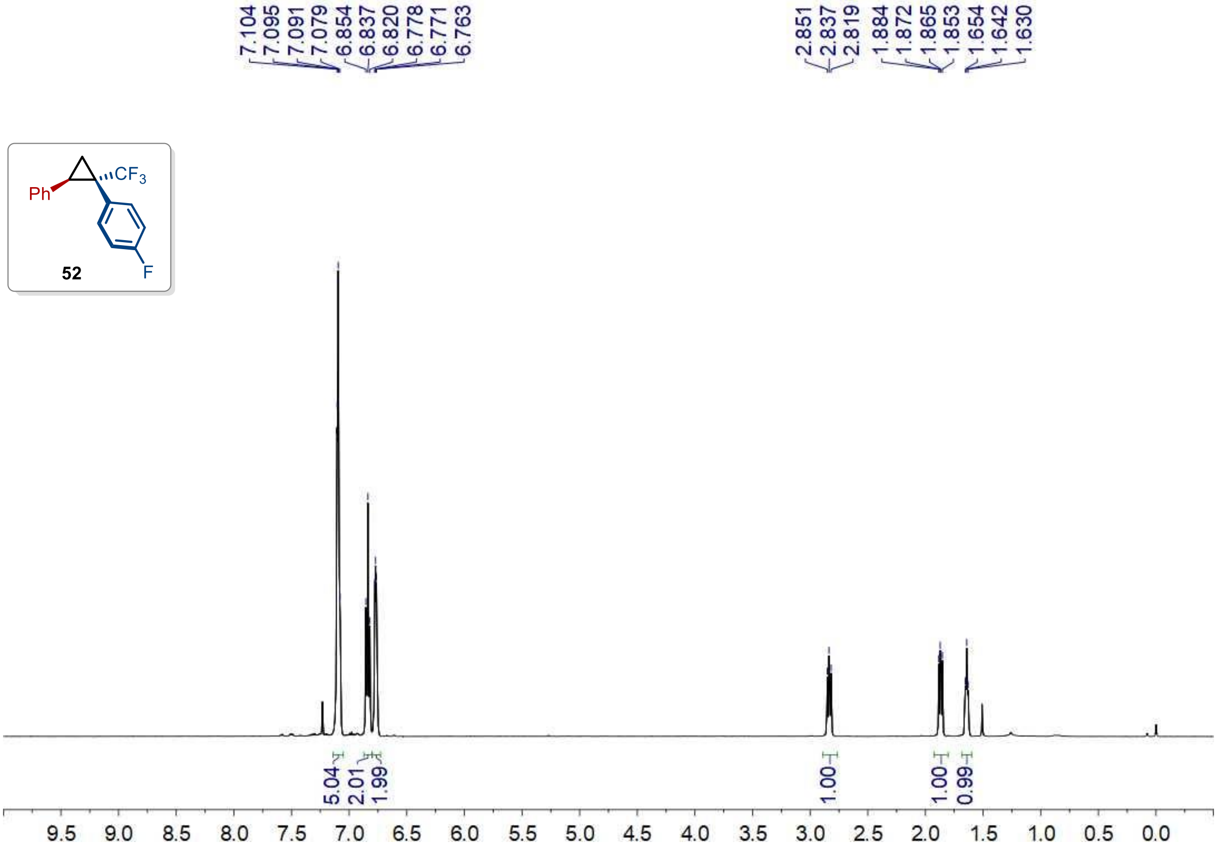

Supplementary Fig. 150 ^{1}**H NMR** (500 MHz, $CDCl_3$) spectrum of compound **52**.

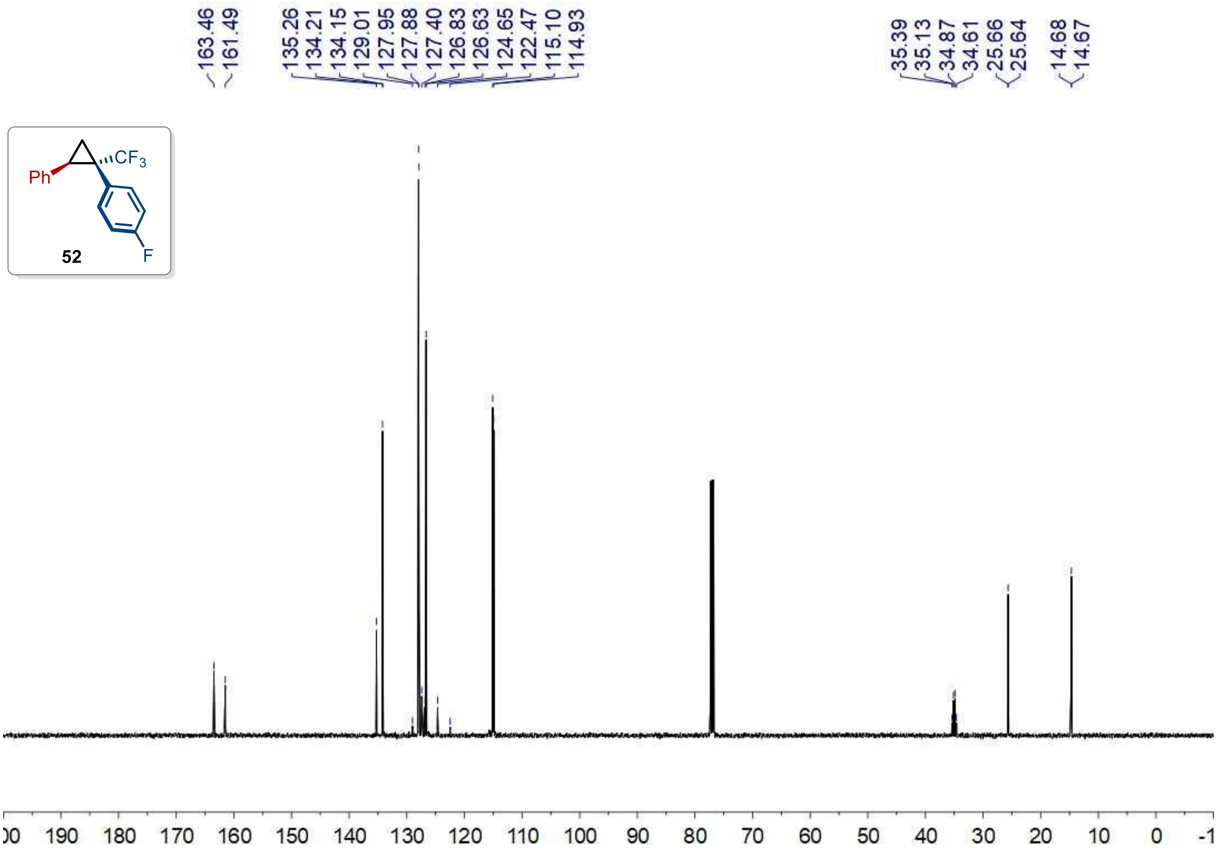

Supplementary Fig. 151 ^{13}**C NMR** (125 MHz, $CDCl_3$) spectrum of compound **52**.

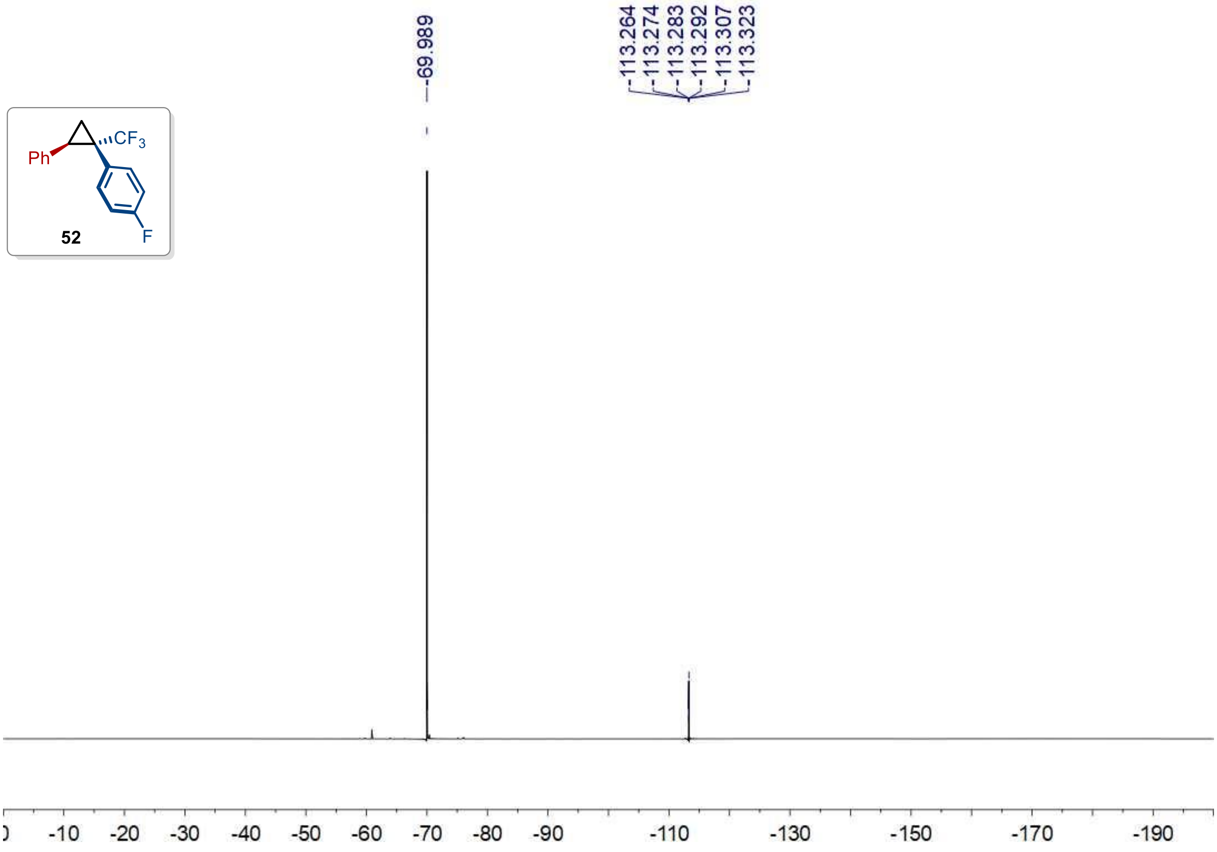

Supplementary Fig. 152 ^{19}**F NMR** (564 MHz, $CDCl_3$) spectrum of compound **52**.

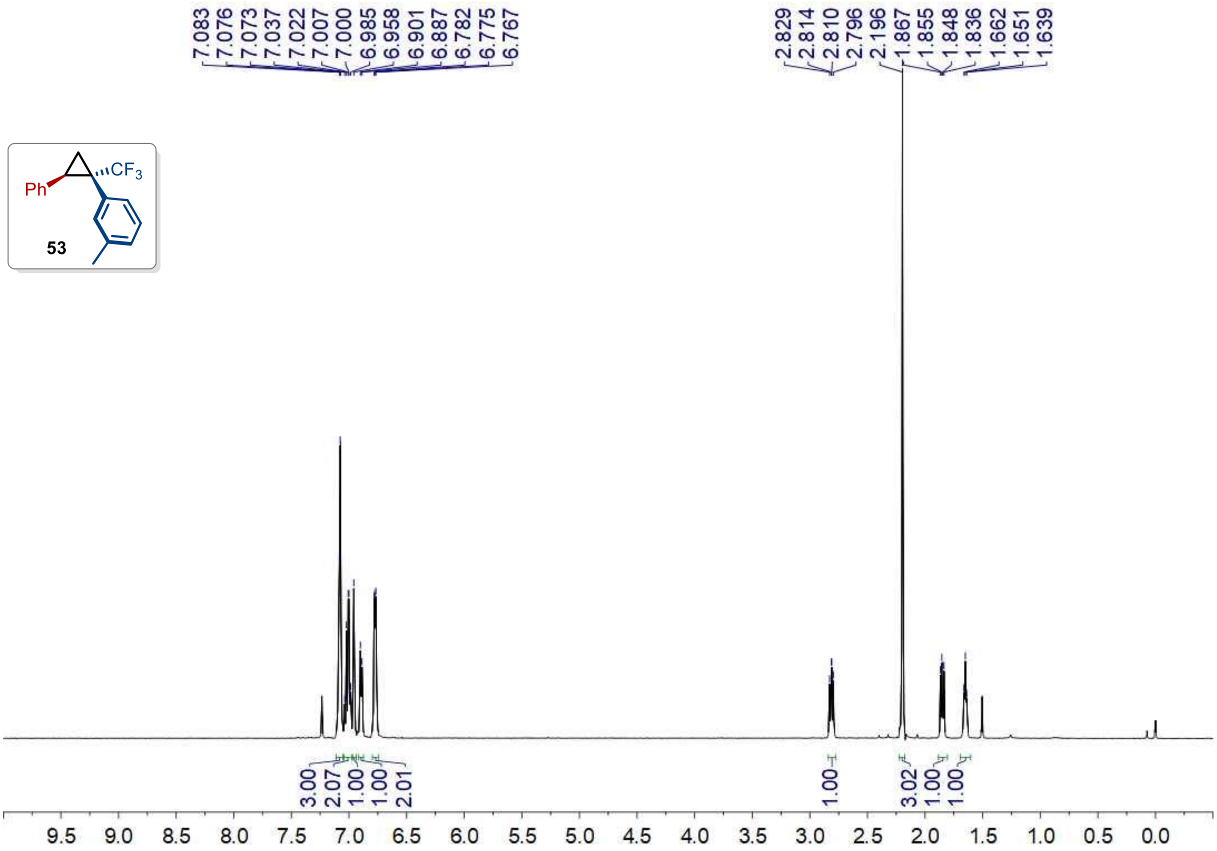

Supplementary Fig. 153 ^{1}**H NMR** (500 MHz, $CDCl_3$) spectrum of compound **53**.

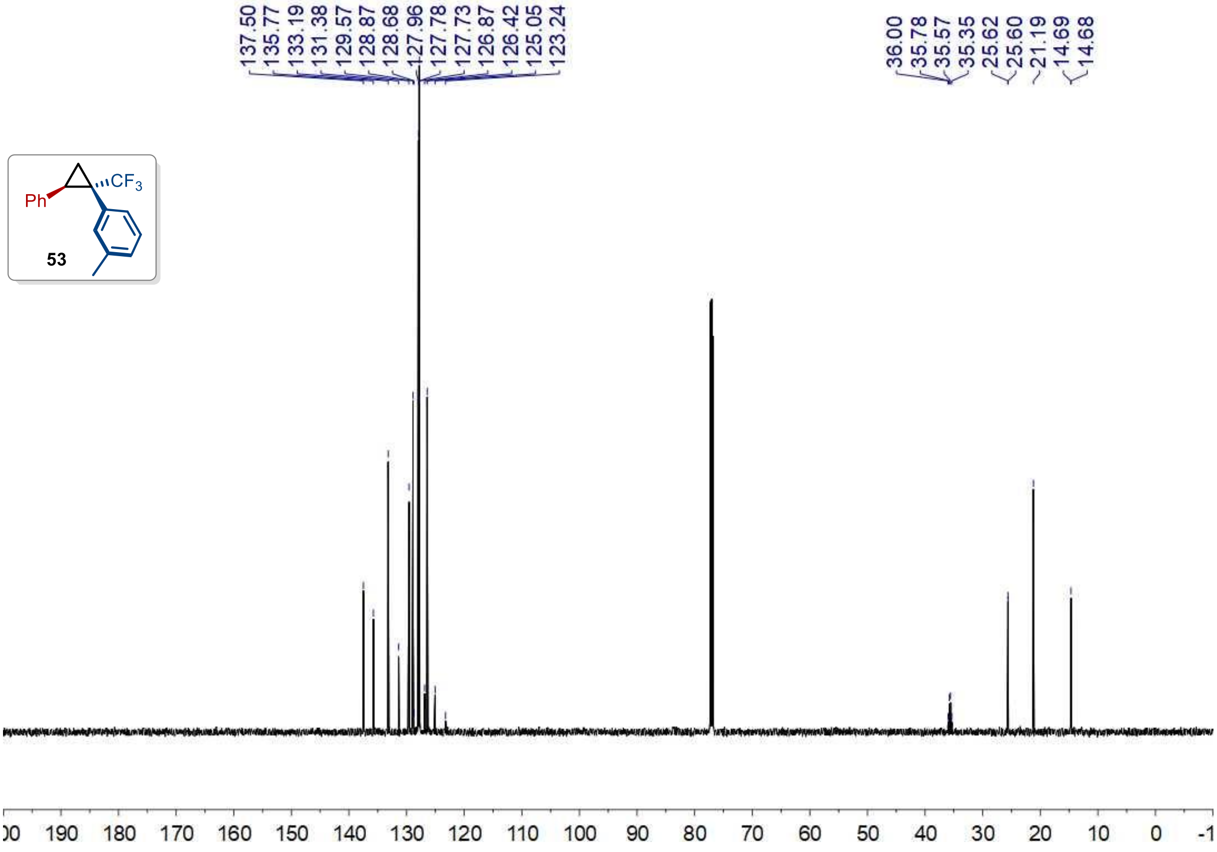

Supplementary Fig. 154 ^{13}**C NMR** (150 MHz, $CDCl_3$) spectrum of compound **53**.

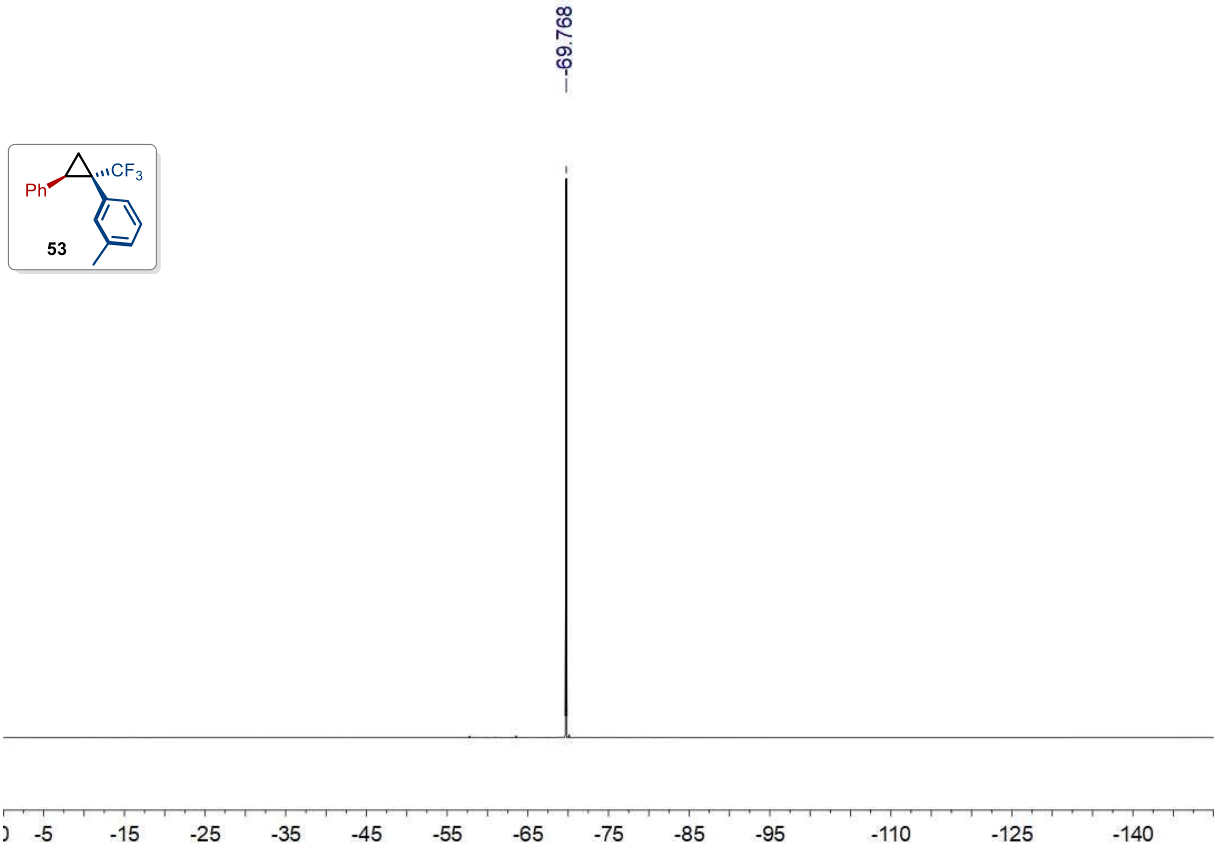

Supplementary Fig. 155 ^{19}**F NMR** (564 MHz, $CDCl_3$) spectrum of compound **53**.

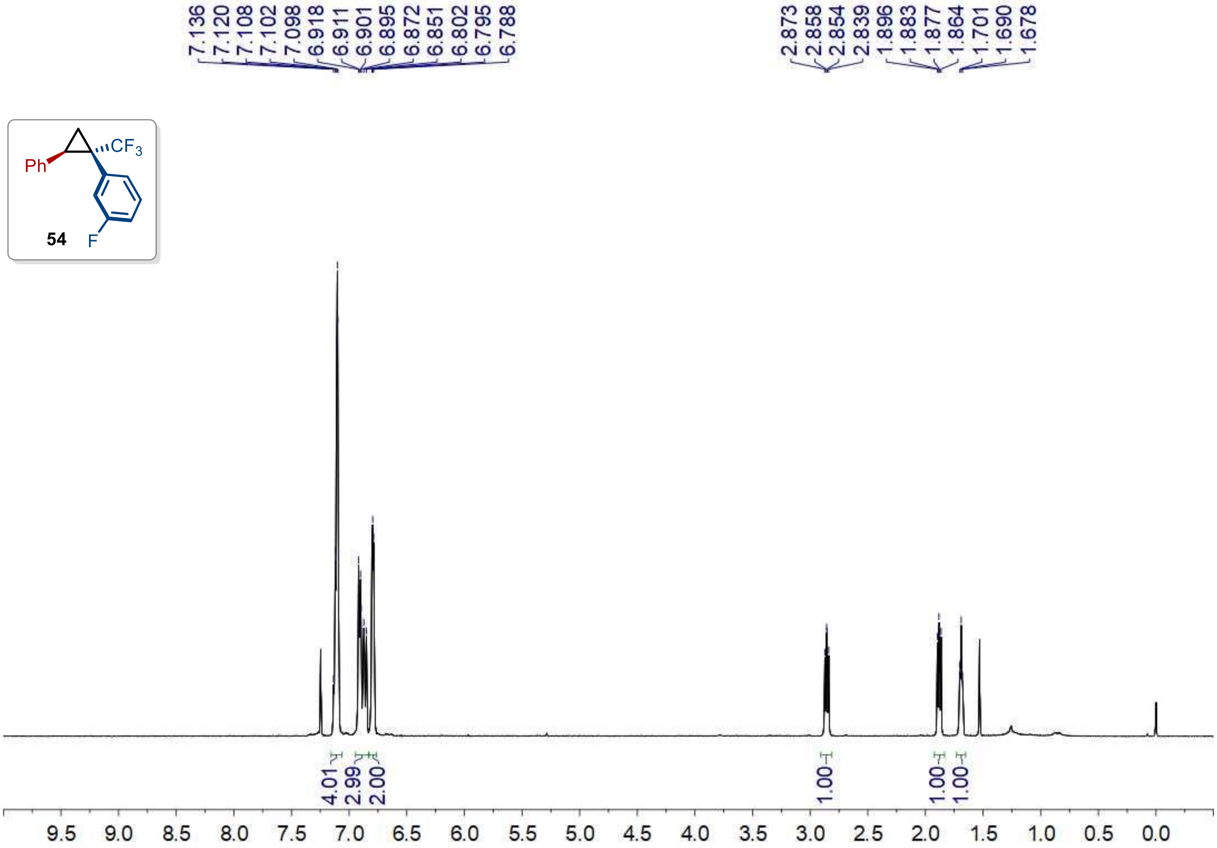

Supplementary Fig. 156 ^{1}**H NMR** (500 MHz, $CDCl_3$) spectrum of compound **54**.

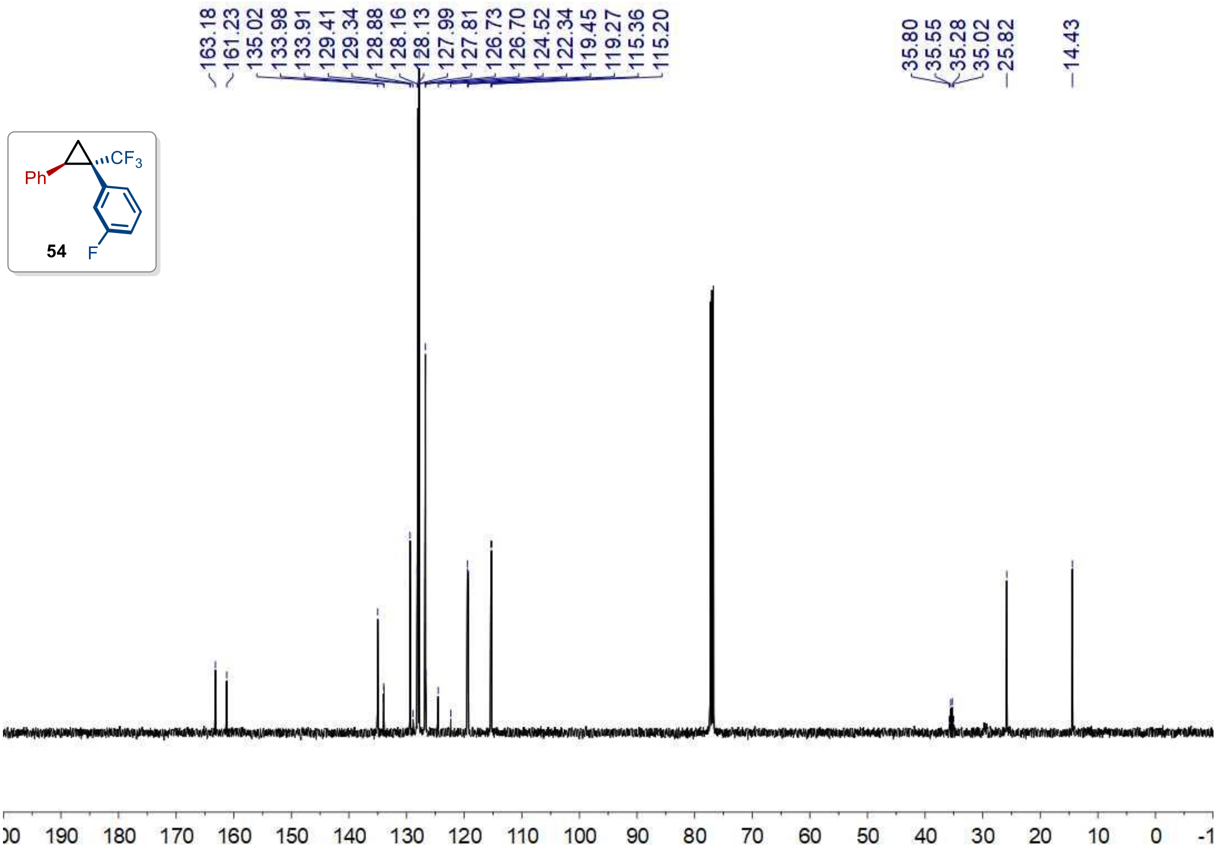

Supplementary Fig. 157 ^{13}C NMR (125 MHz, $CDCl_3$) spectrum of compound **54**.

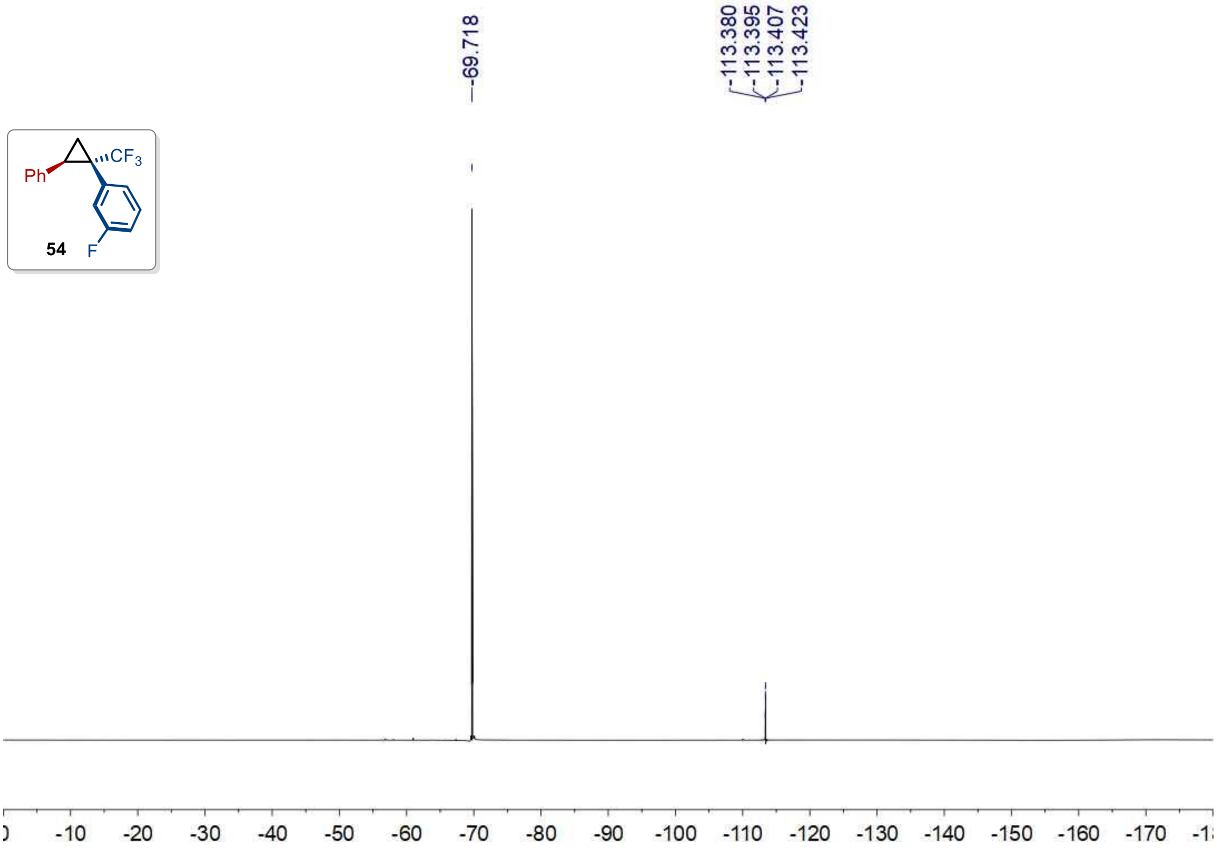

Supplementary Fig. 158 ^{19}F NMR (564 MHz, $CDCl_3$) spectrum of compound **54**.

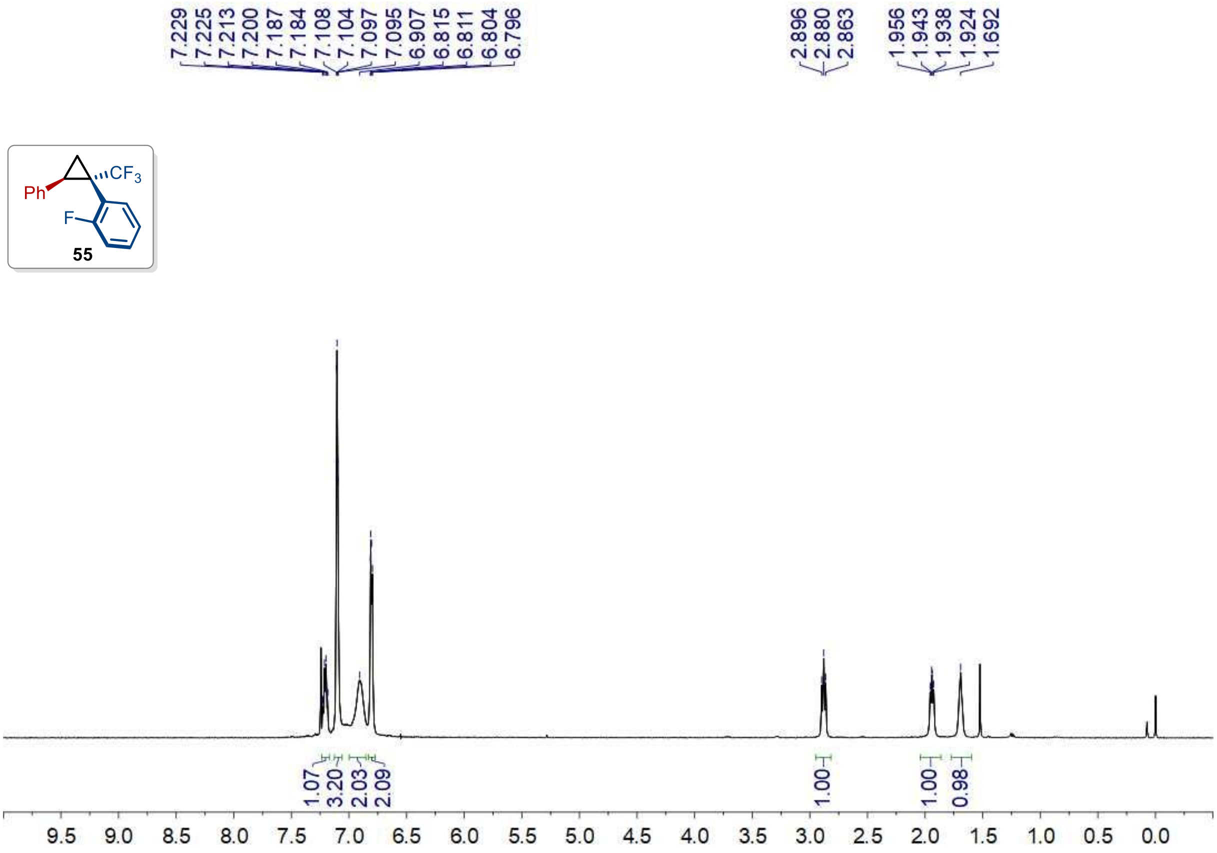

Supplementary Fig. 159 ^{1}H NMR (500 MHz, $CDCl_3$) spectrum of compound **55**.

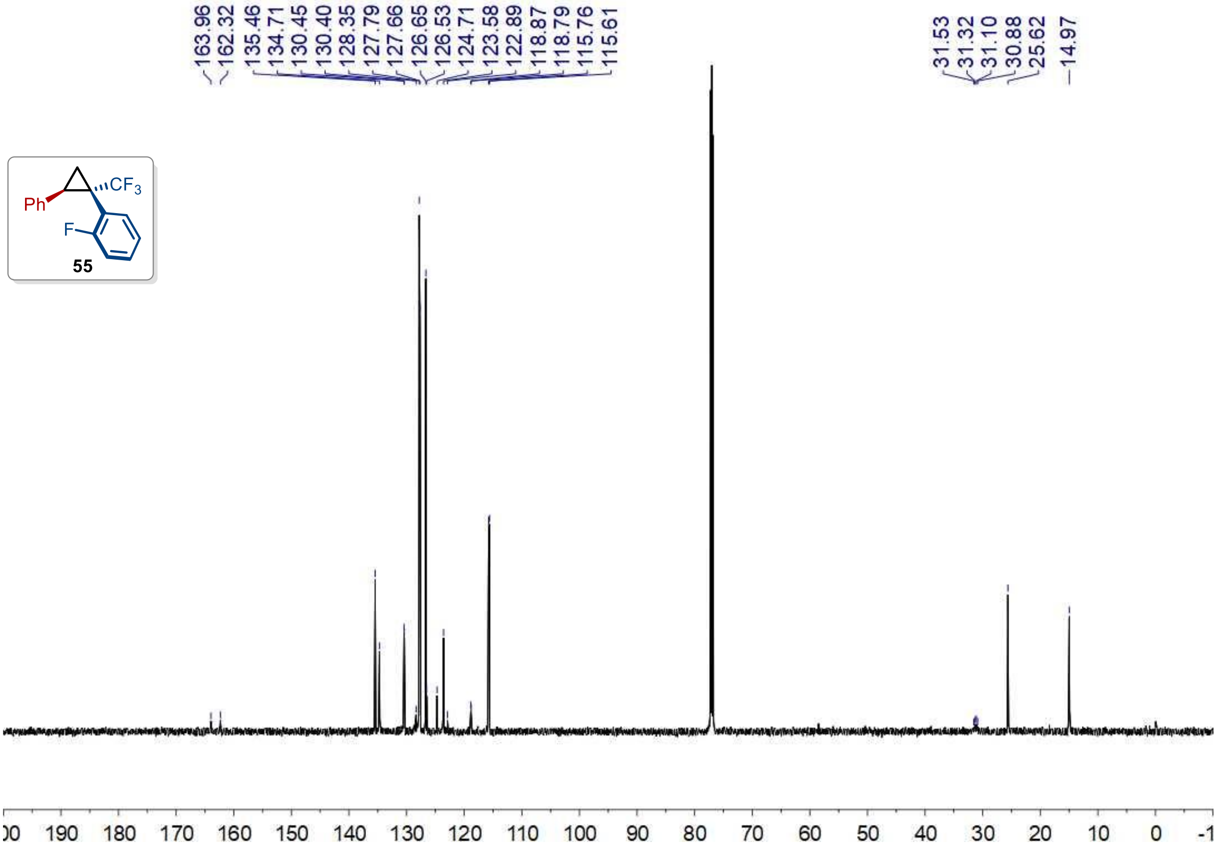

Supplementary Fig. 160 ^{13}C NMR (150 MHz, $CDCl_3$) spectrum of compound **55**.

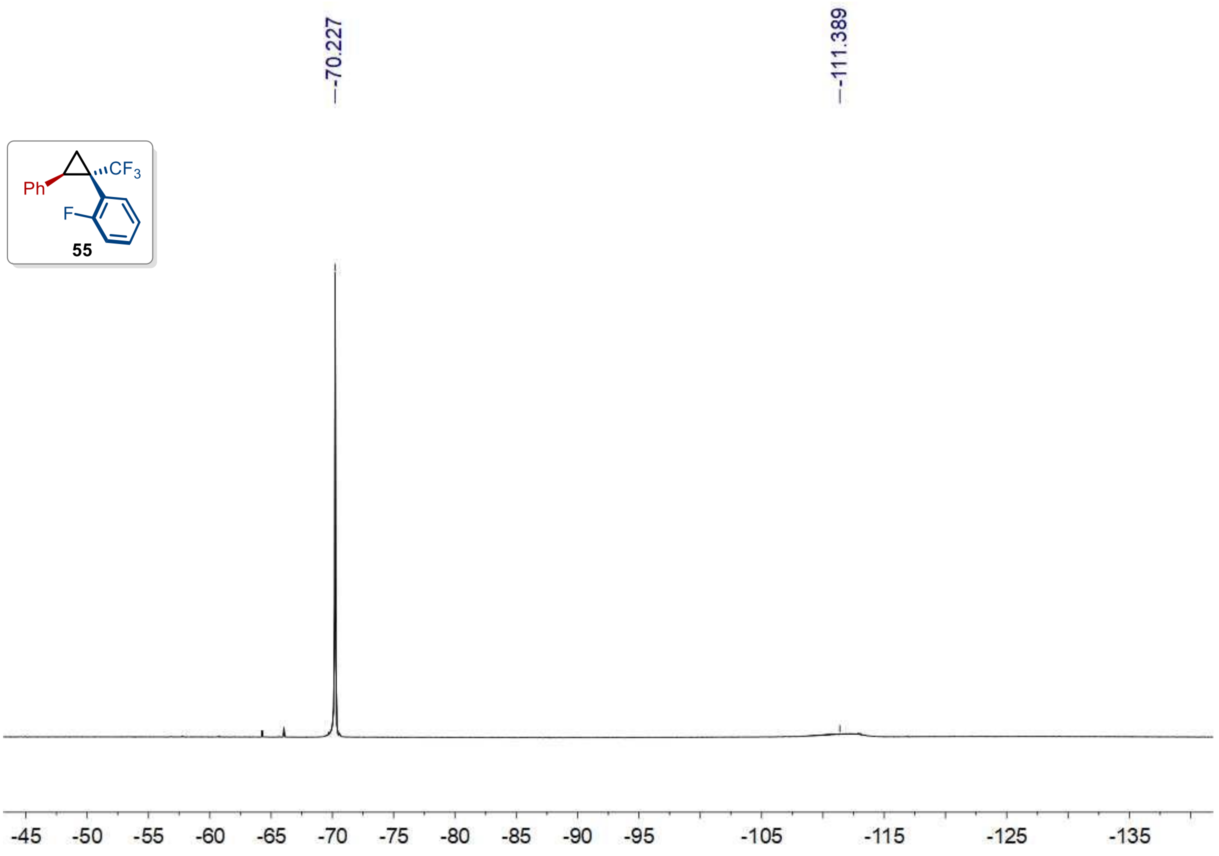

Supplementary Fig. 161 ^{19}**F NMR** (564 MHz, $CDCl_3$) spectrum of compound **55**.

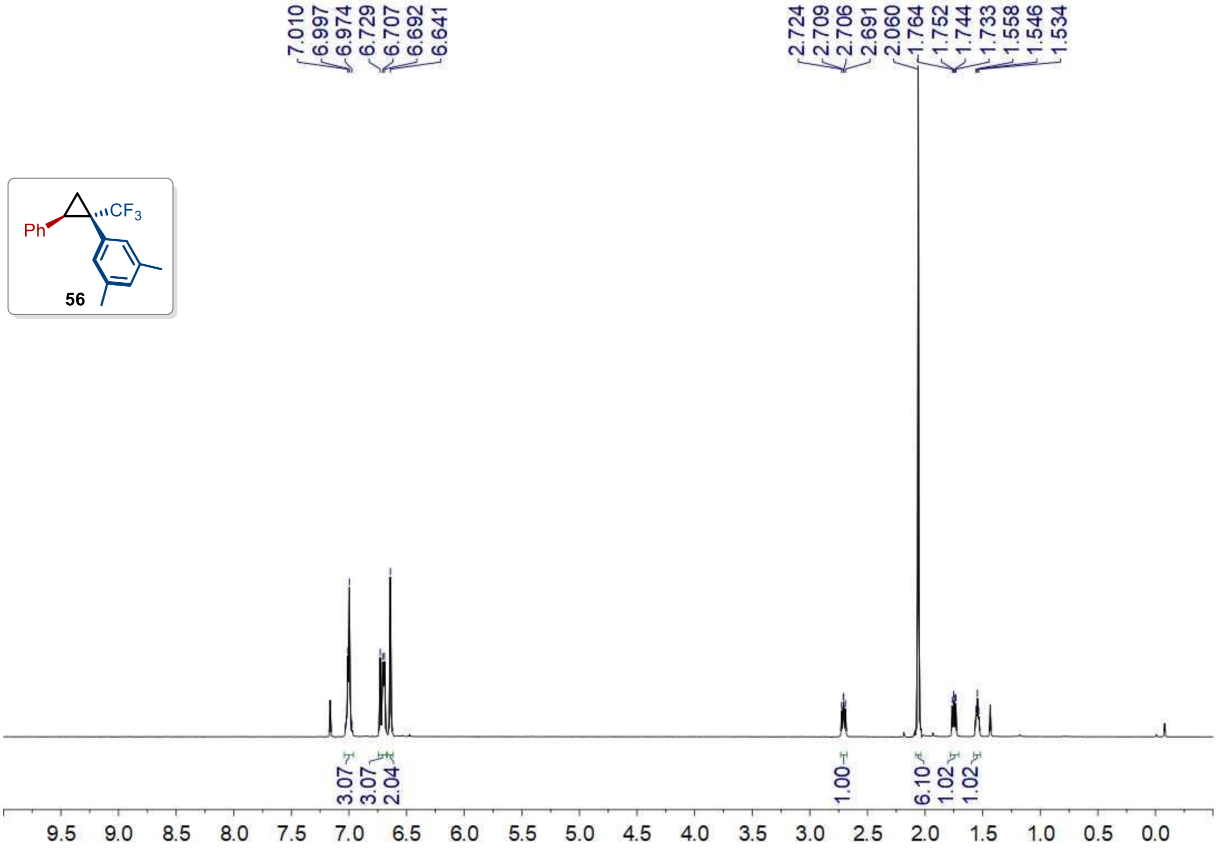

Supplementary Fig. 162 ^{1}**H NMR** (500 MHz, $CDCl_3$) spectrum of compound **56**.

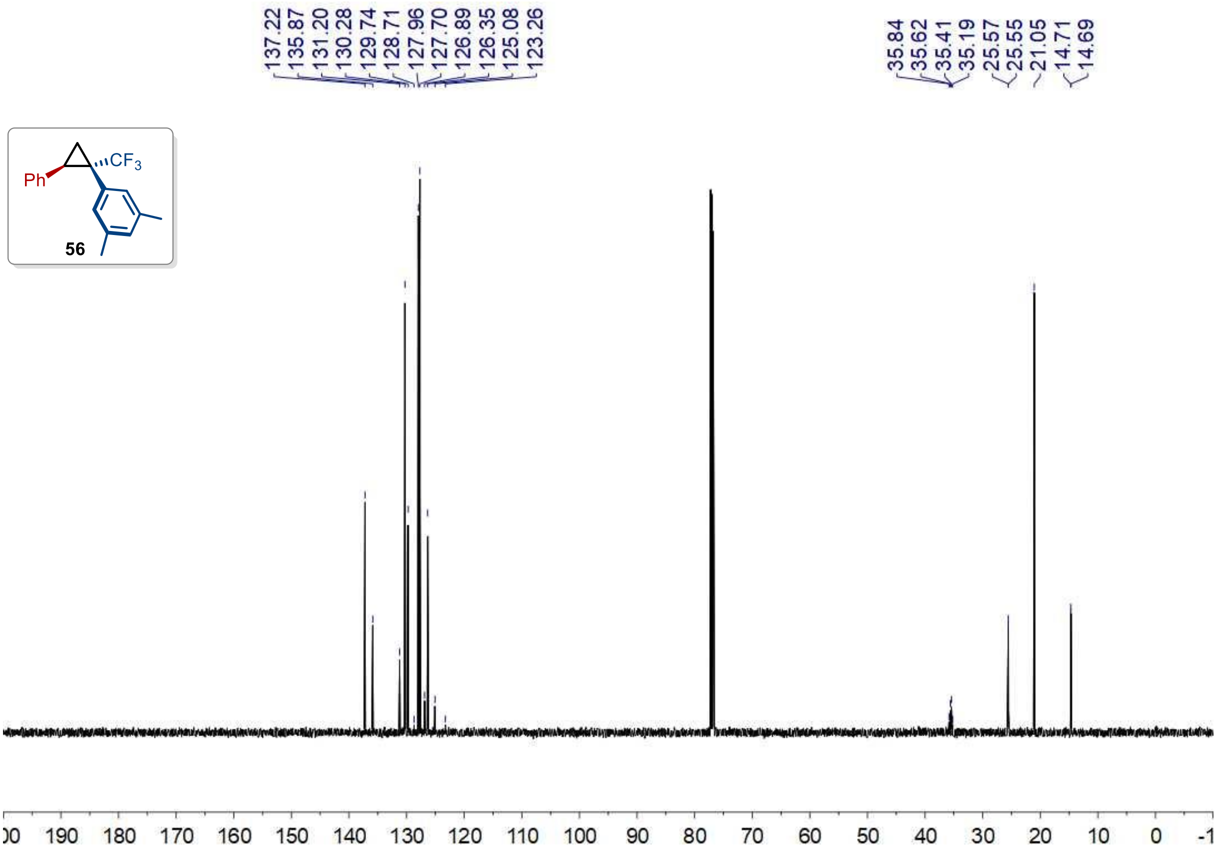

Supplementary Fig. 163 ^{13}**C NMR** (150 MHz, $CDCl_3$) spectrum of compound **56**.

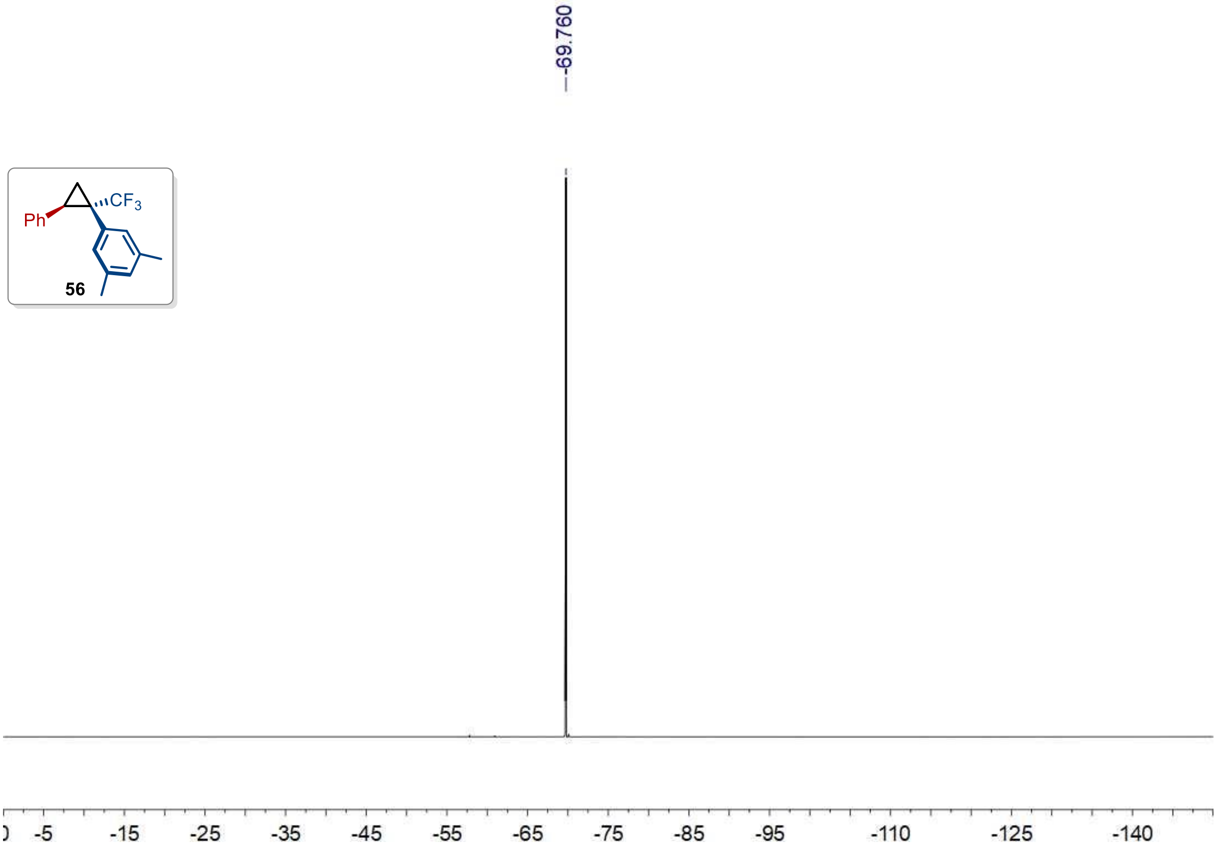

Supplementary Fig. 164 ^{19}**F NMR** (564 MHz, $CDCl_3$) spectrum of compound **56**.

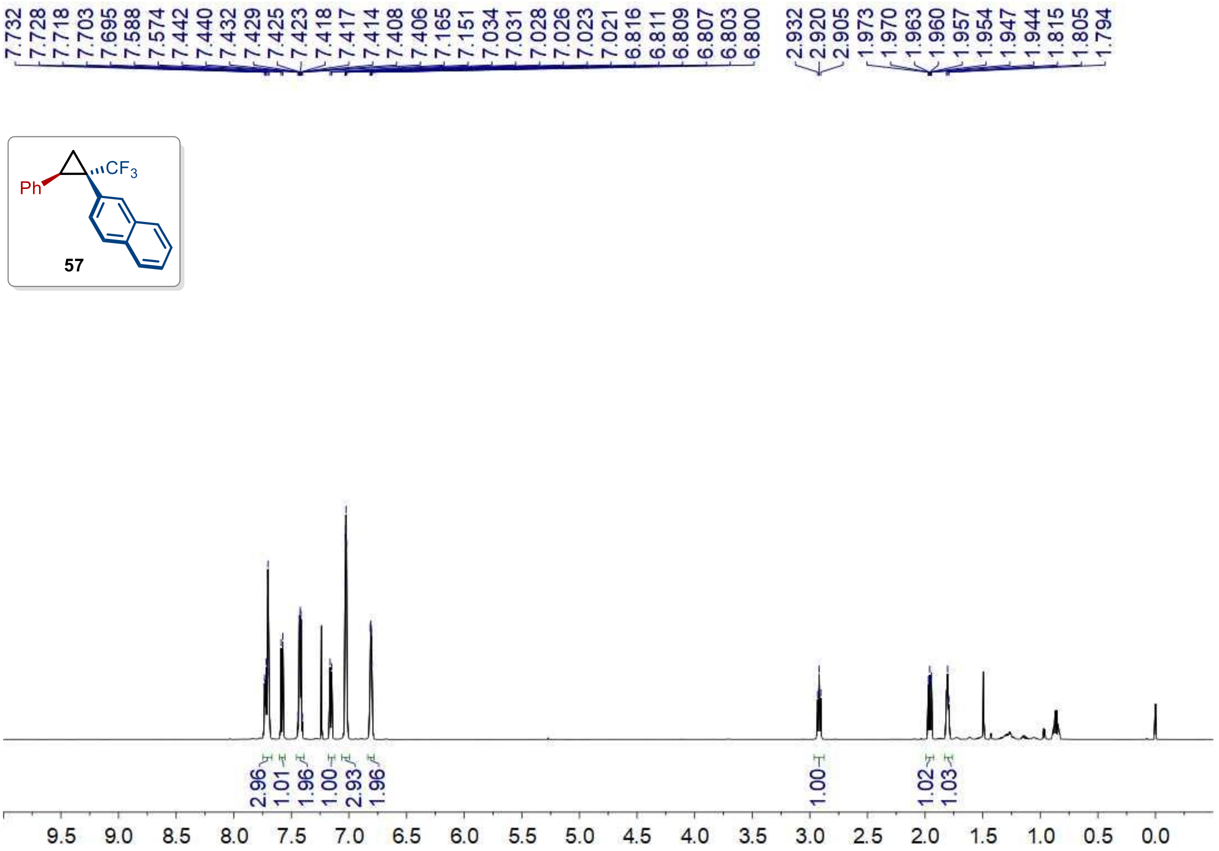

Supplementary Fig. 165 ^{1}**H NMR** (500 MHz, $CDCl_3$) spectrum of compound **57**.

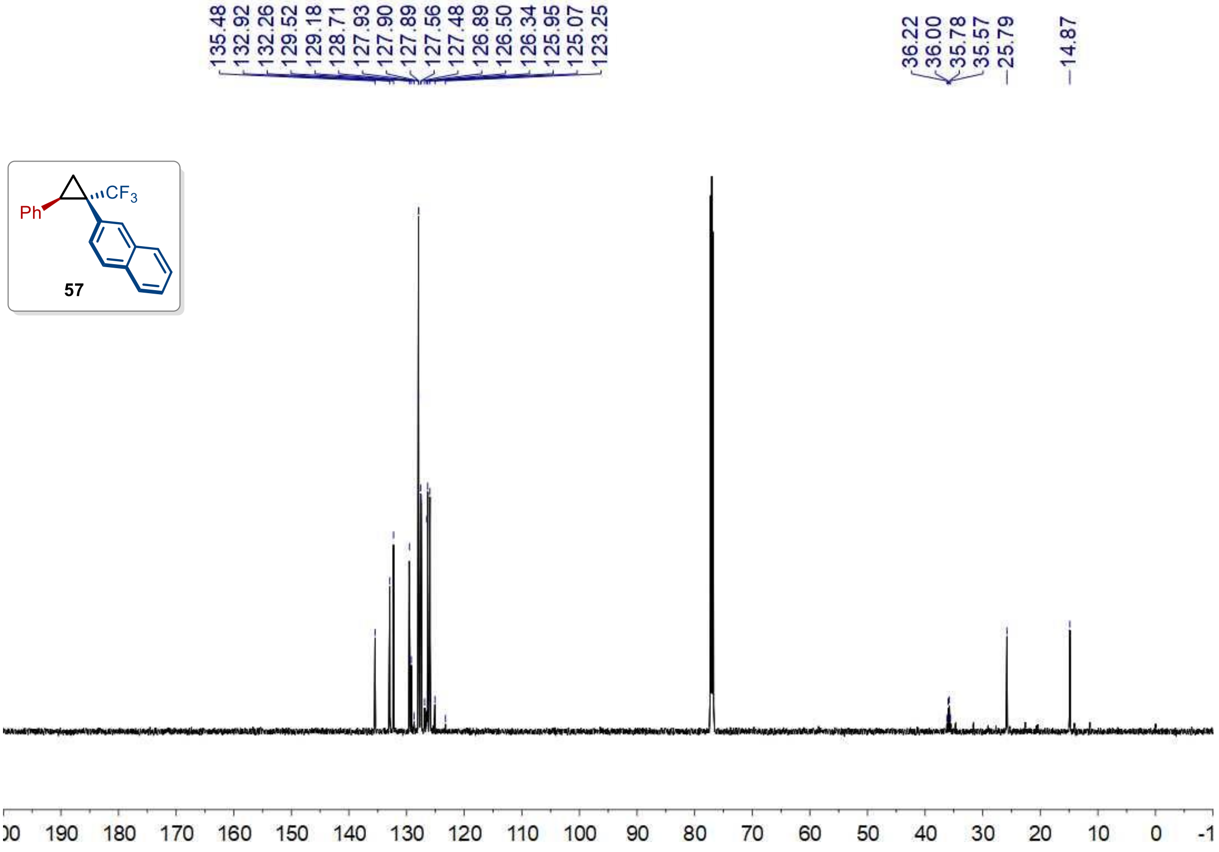

Supplementary Fig. 166 ^{13}**C NMR** (150 MHz, $CDCl_3$) spectrum of compound **57**.

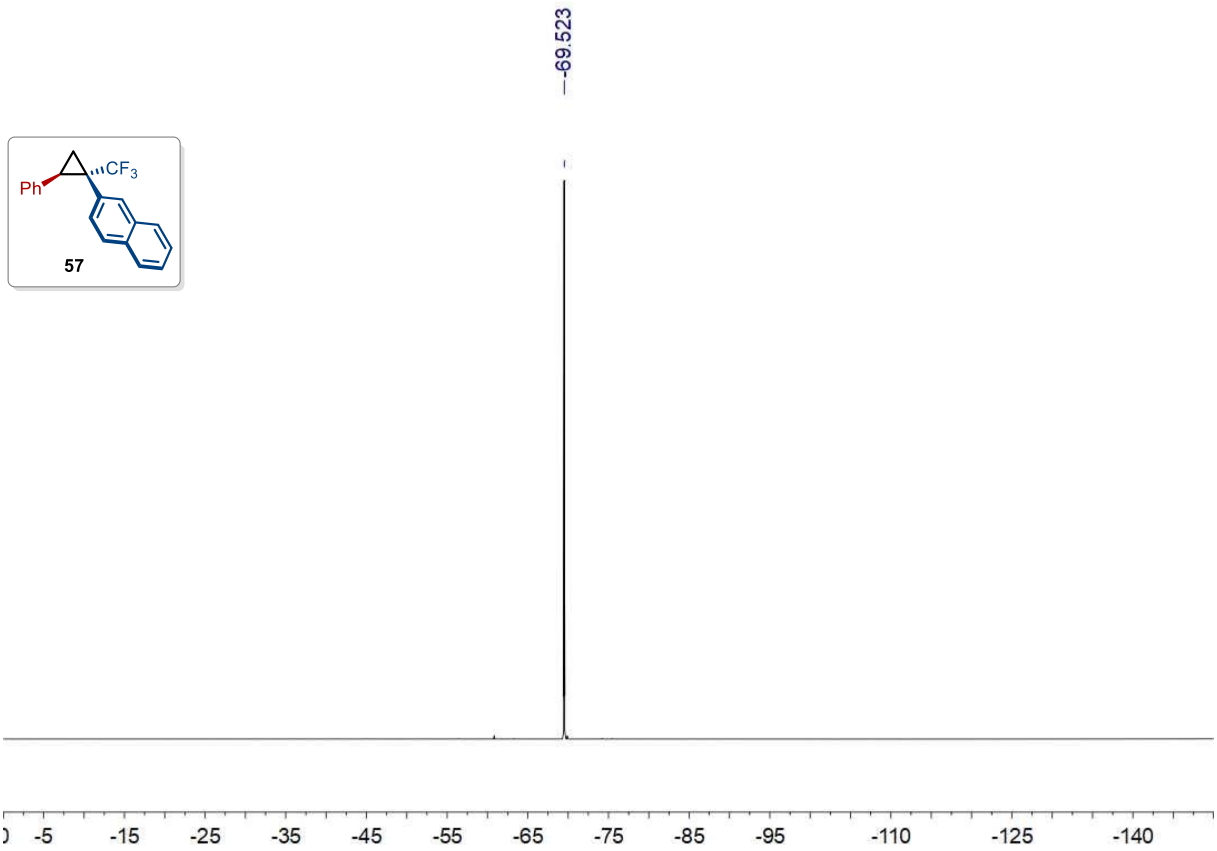

Supplementary Fig. 167 ^{19}**F NMR** (564 MHz, $CDCl_3$) spectrum of compound **57**.

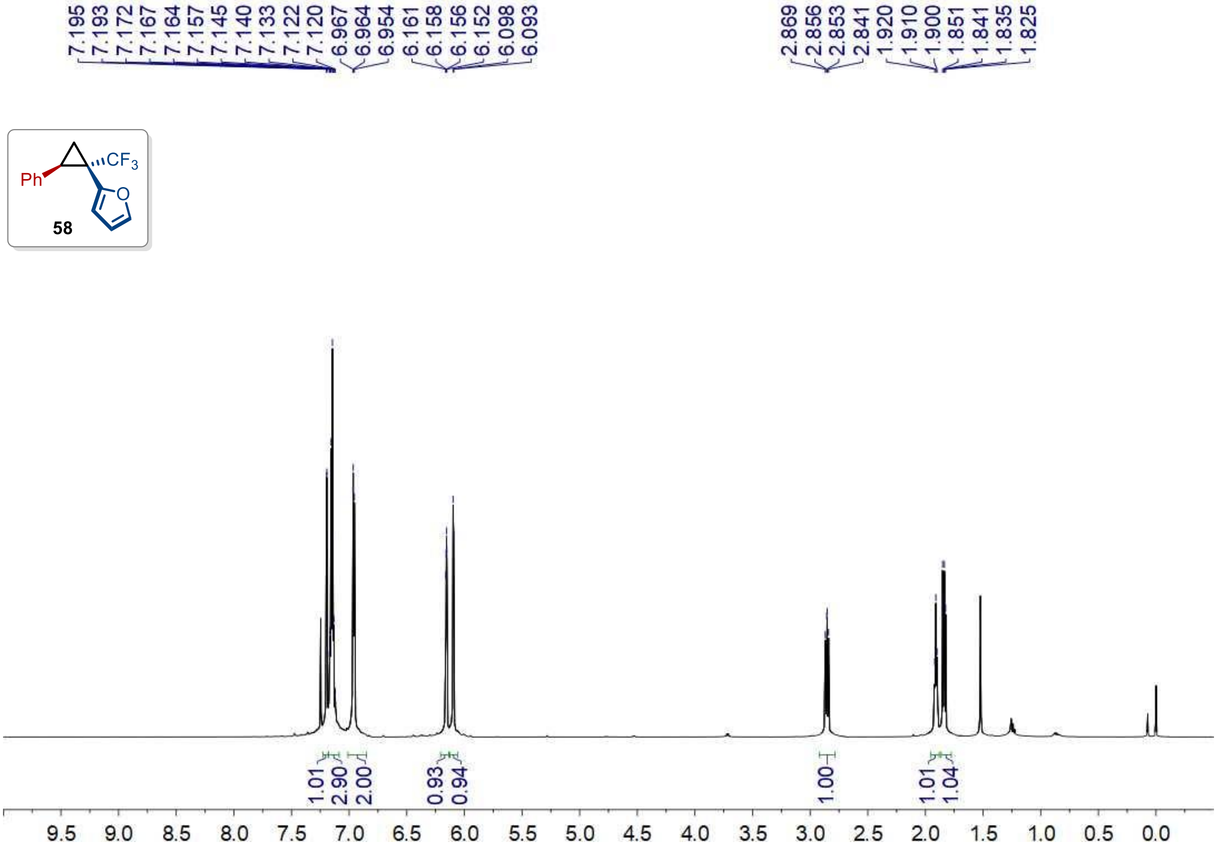

Supplementary Fig. 168 ^{1}**H NMR** (500 MHz, $CDCl_3$) spectrum of compound **58**.

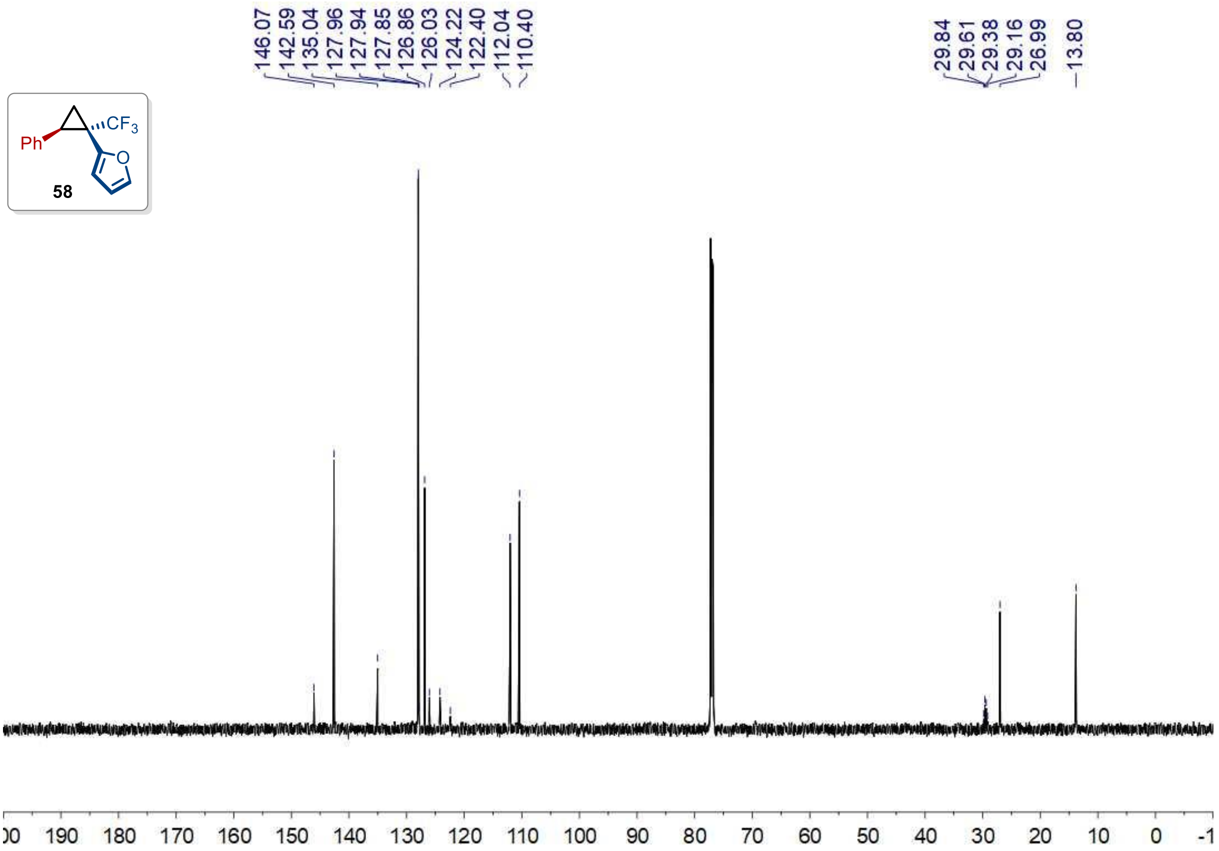

Supplementary Fig. 169 ^{13}**C NMR** (150 MHz, $CDCl_3$) spectrum of compound **58**.

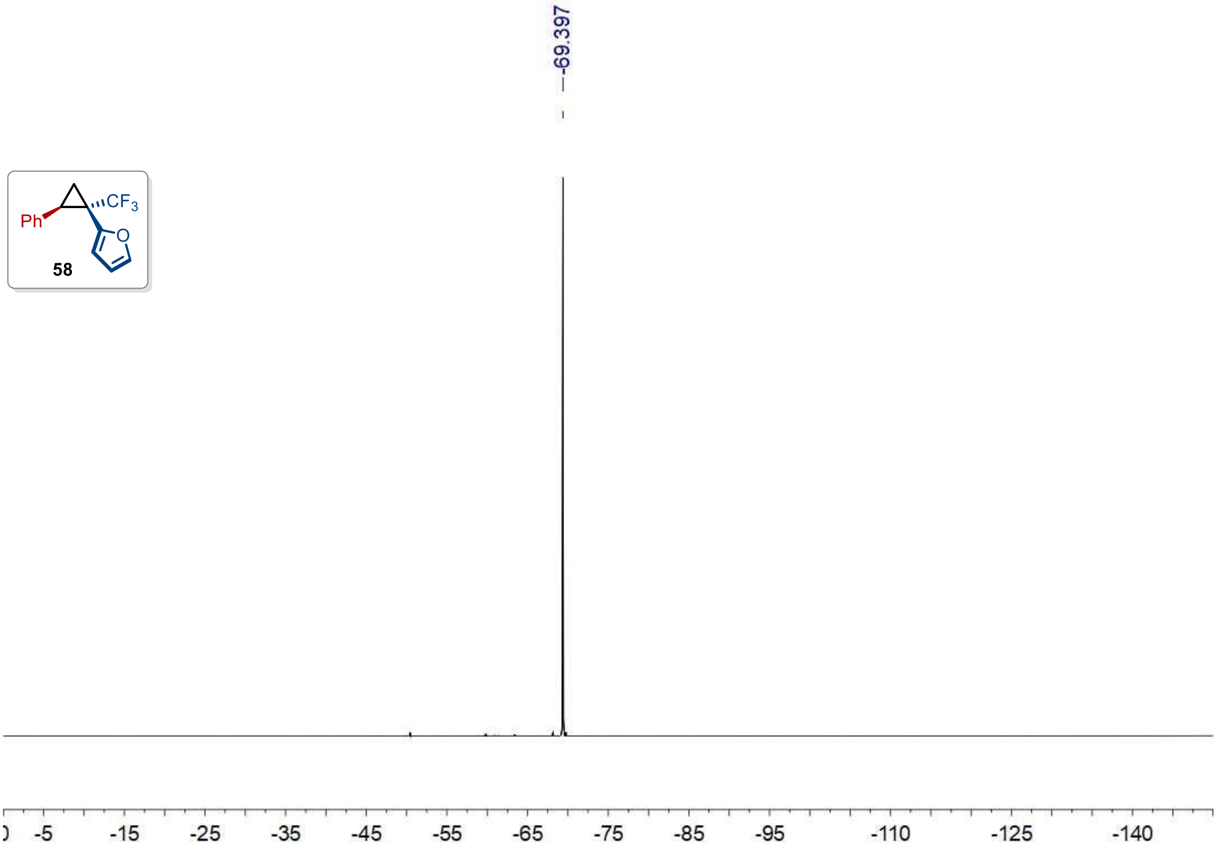

Supplementary Fig. 170 ^{19}**F NMR** (564 MHz, $CDCl_3$) spectrum of compound **58**.

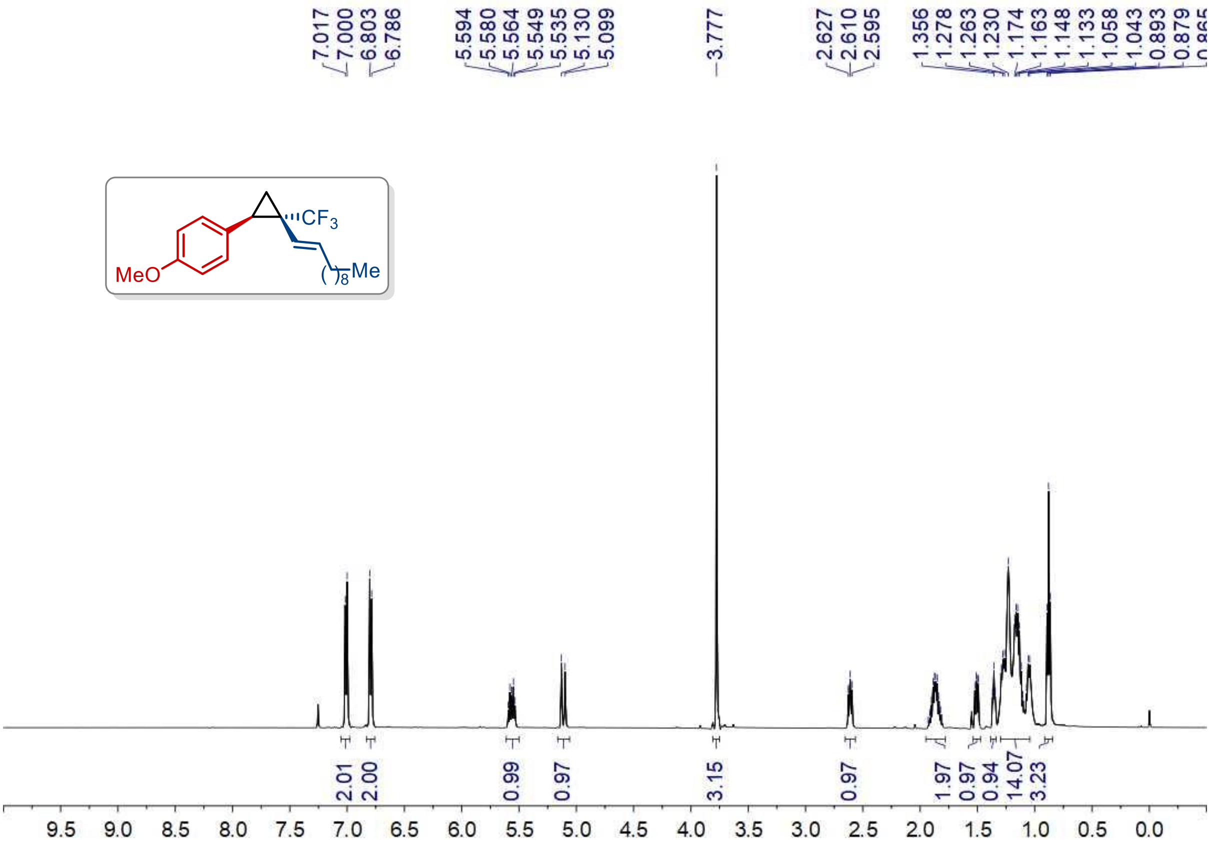

Supplementary Fig. 171 ^{1}**H NMR** (500 MHz, $CDCl_3$) spectrum of compound **59**.

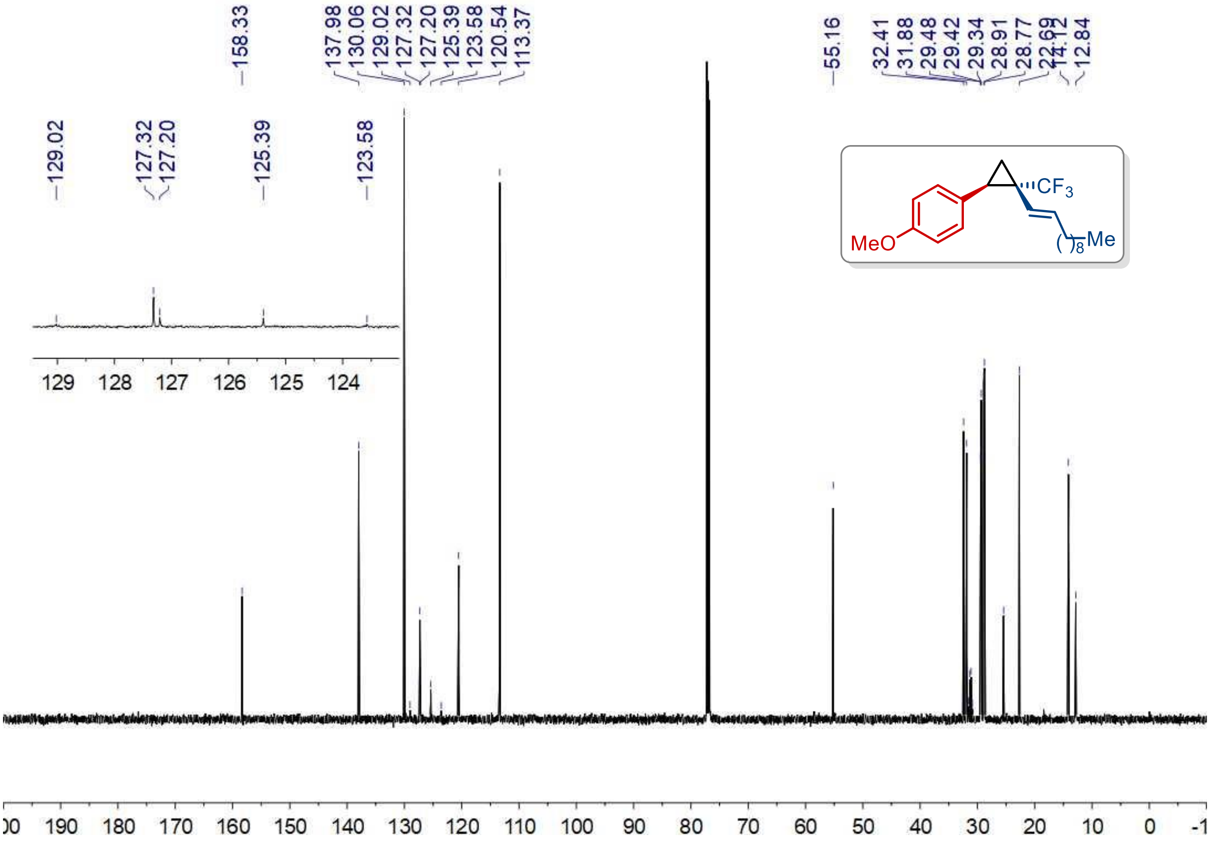

Supplementary Fig. 172 ^{13}**C NMR** (150 MHz, $CDCl_3$) spectrum of compound **59**.

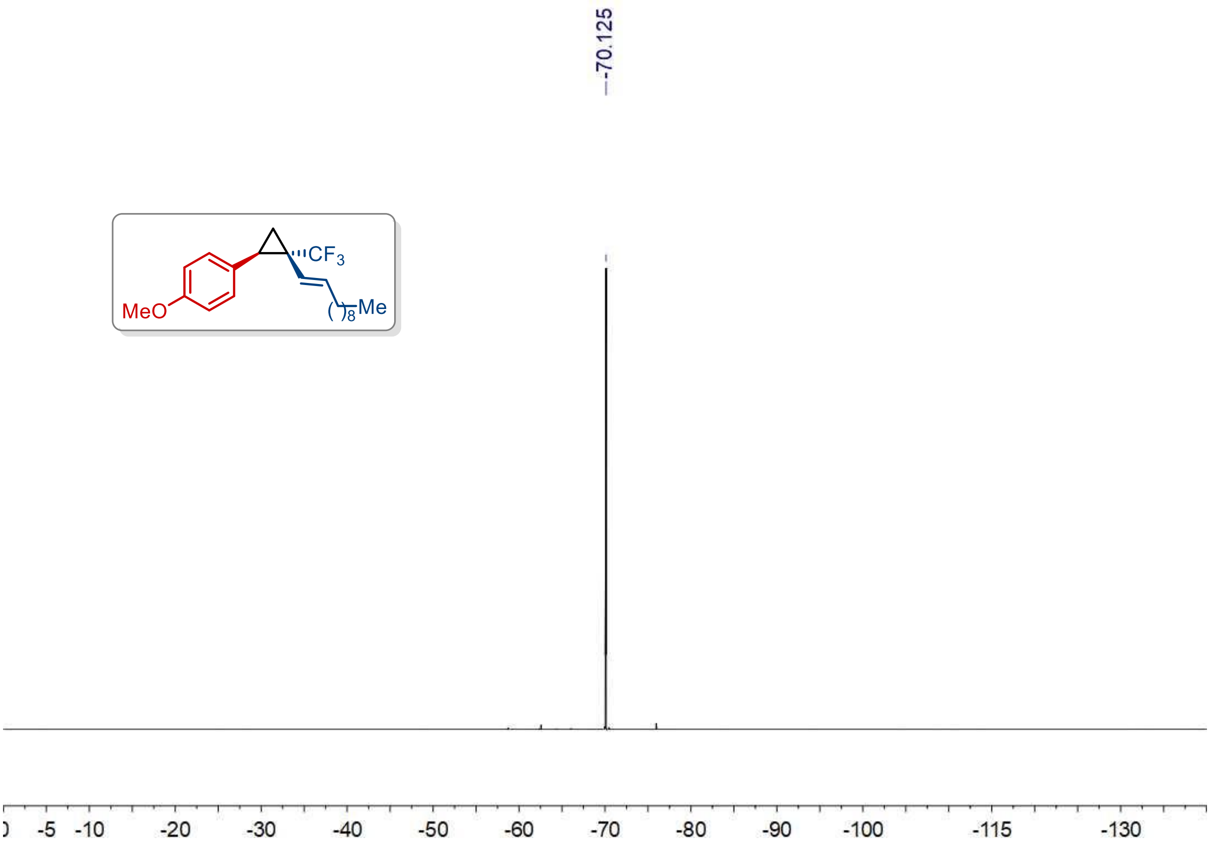

Supplementary Fig. 173 ^{19}F NMR (564 MHz, $CDCl_3$) spectrum of compound **59**.

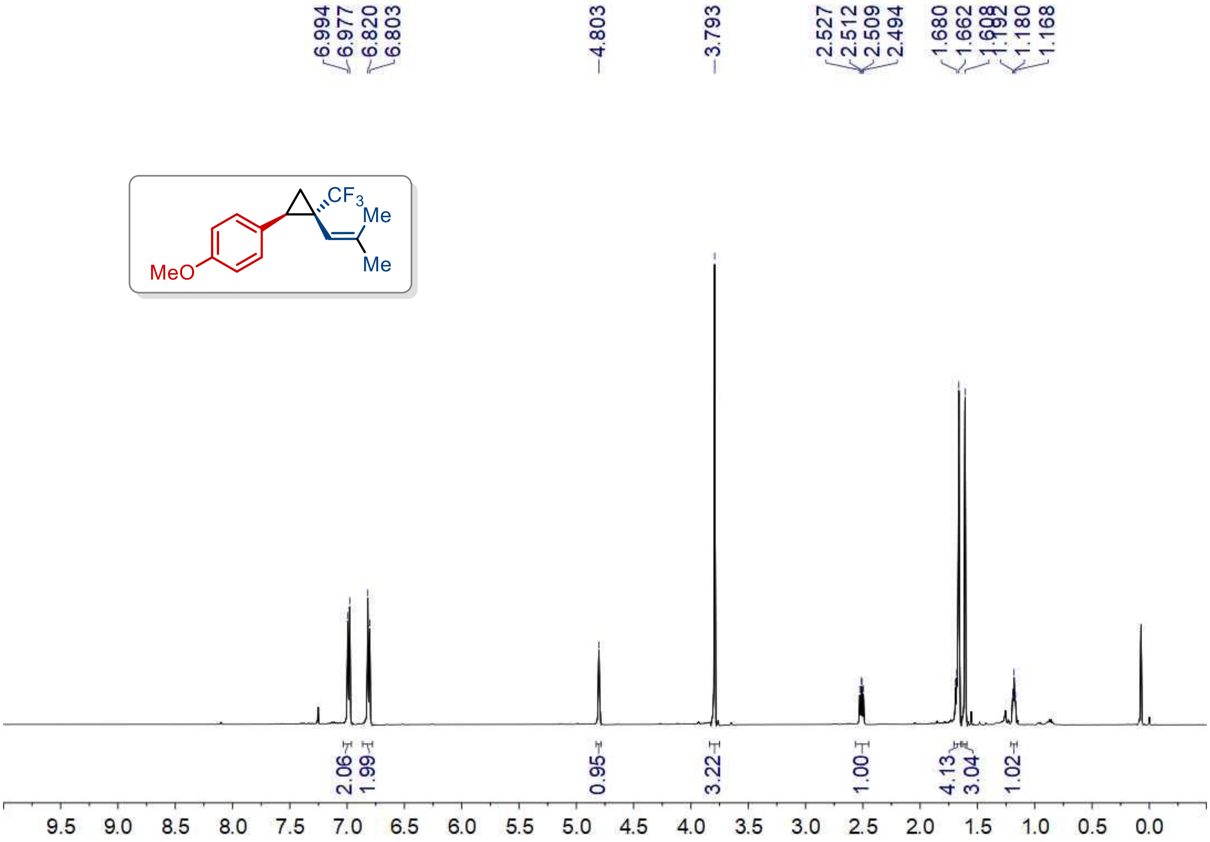

Supplementary Fig. 174 ^{1}H NMR (500 MHz, $CDCl_3$) spectrum of compound **60**.

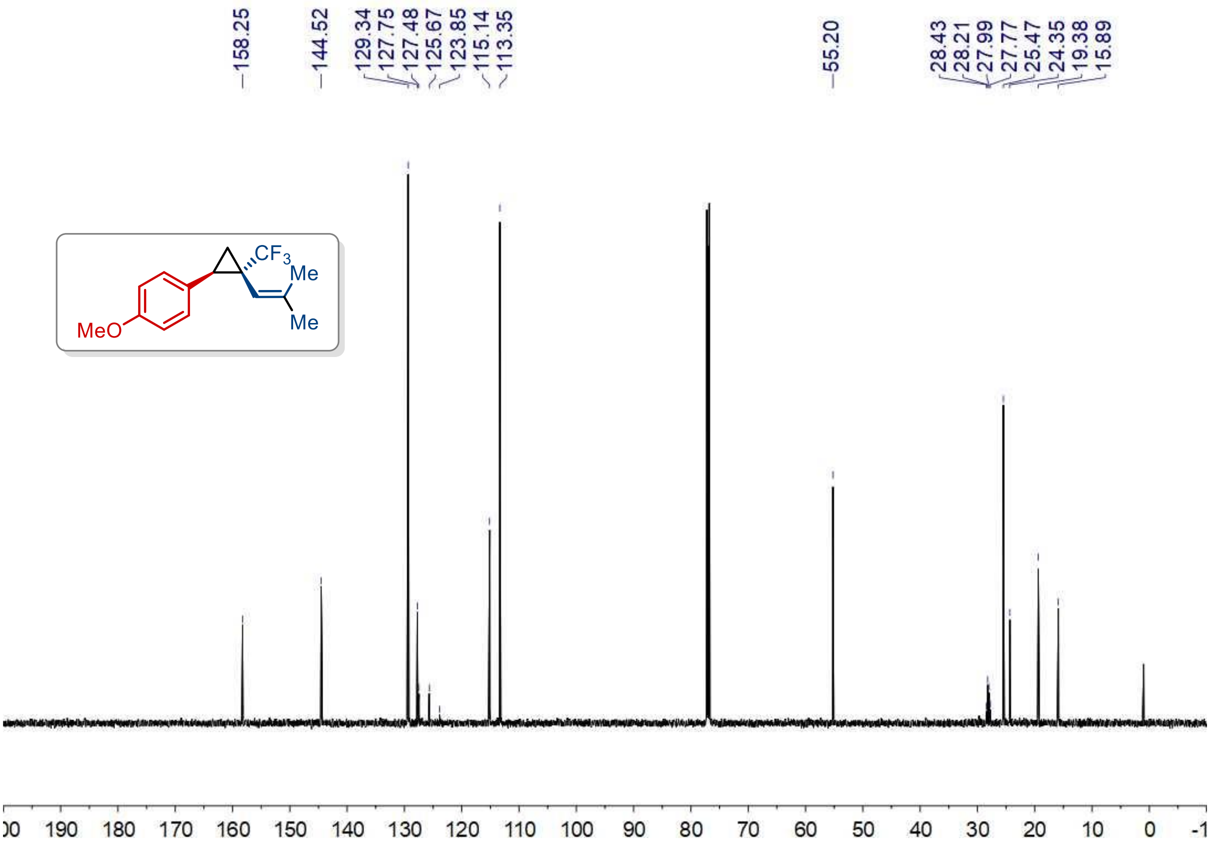

Supplementary Fig. 175 ^{13}**C NMR** (150 MHz, $CDCl_3$) spectrum of compound **60**.

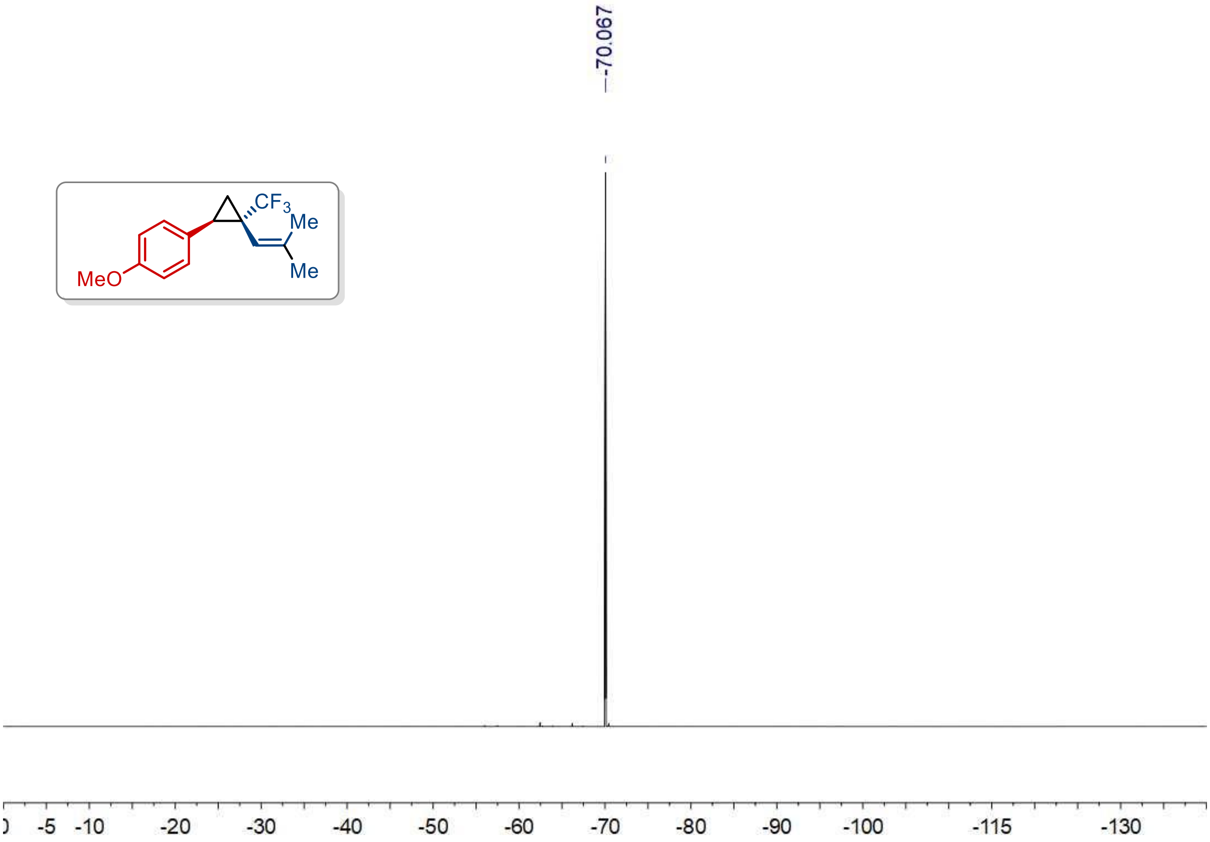

Supplementary Fig. 176 ^{19}**F NMR** (564 MHz, $CDCl_3$) spectrum of compound **60**.

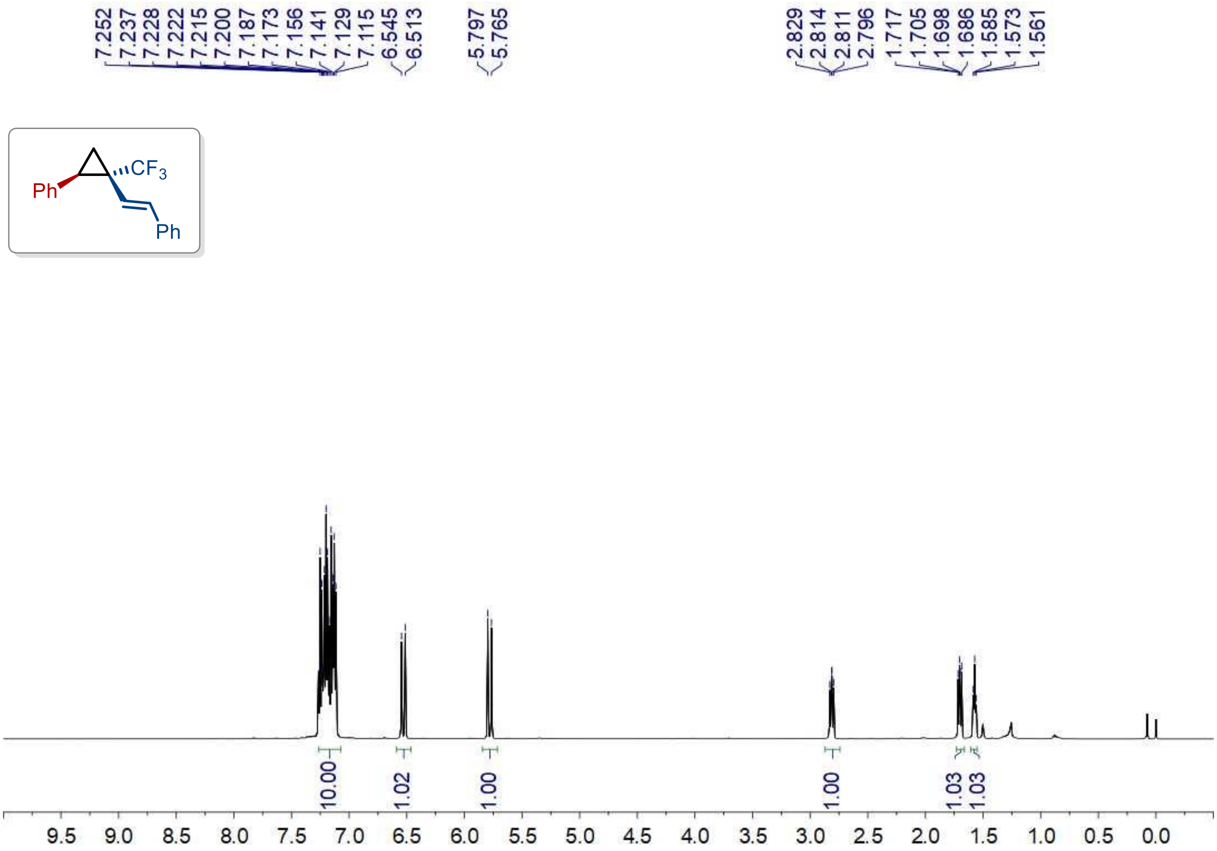

Supplementary Fig. 177 ^{1}H NMR (500 MHz, $CDCl_3$) spectrum of compound **61**.

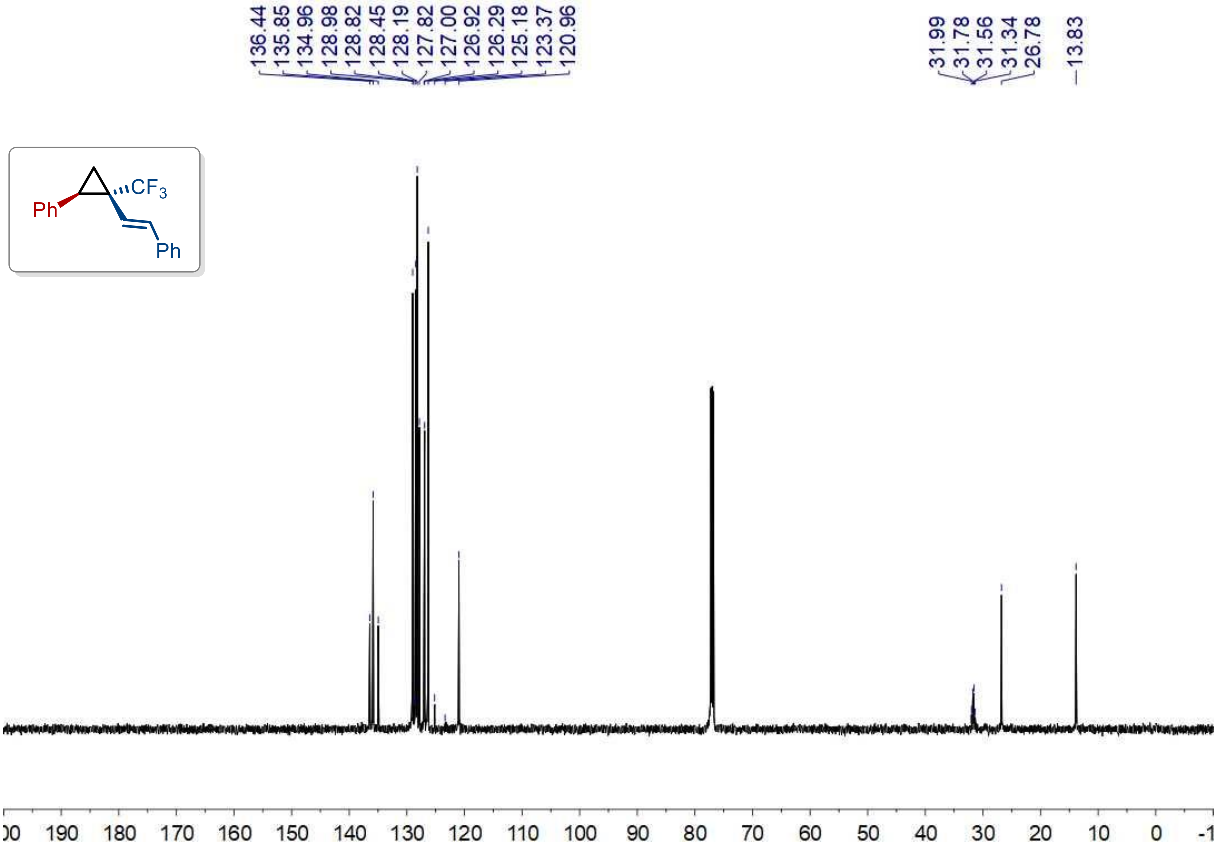

Supplementary Fig. 178 ^{13}C NMR (150 MHz, $CDCl_3$) spectrum of compound **61**.

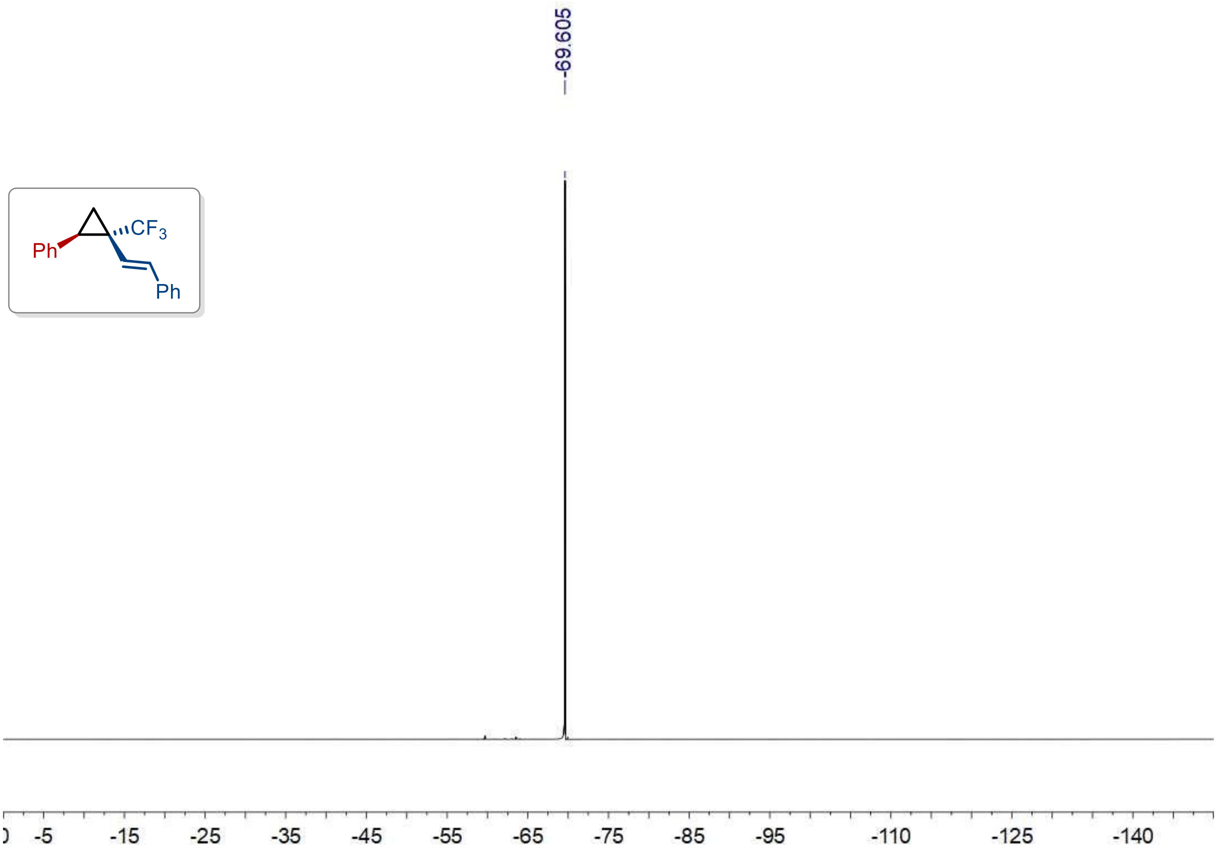

Supplementary Fig. 179 ^{19}**F NMR** (564 MHz, $CDCl_3$) spectrum of compound **61**.

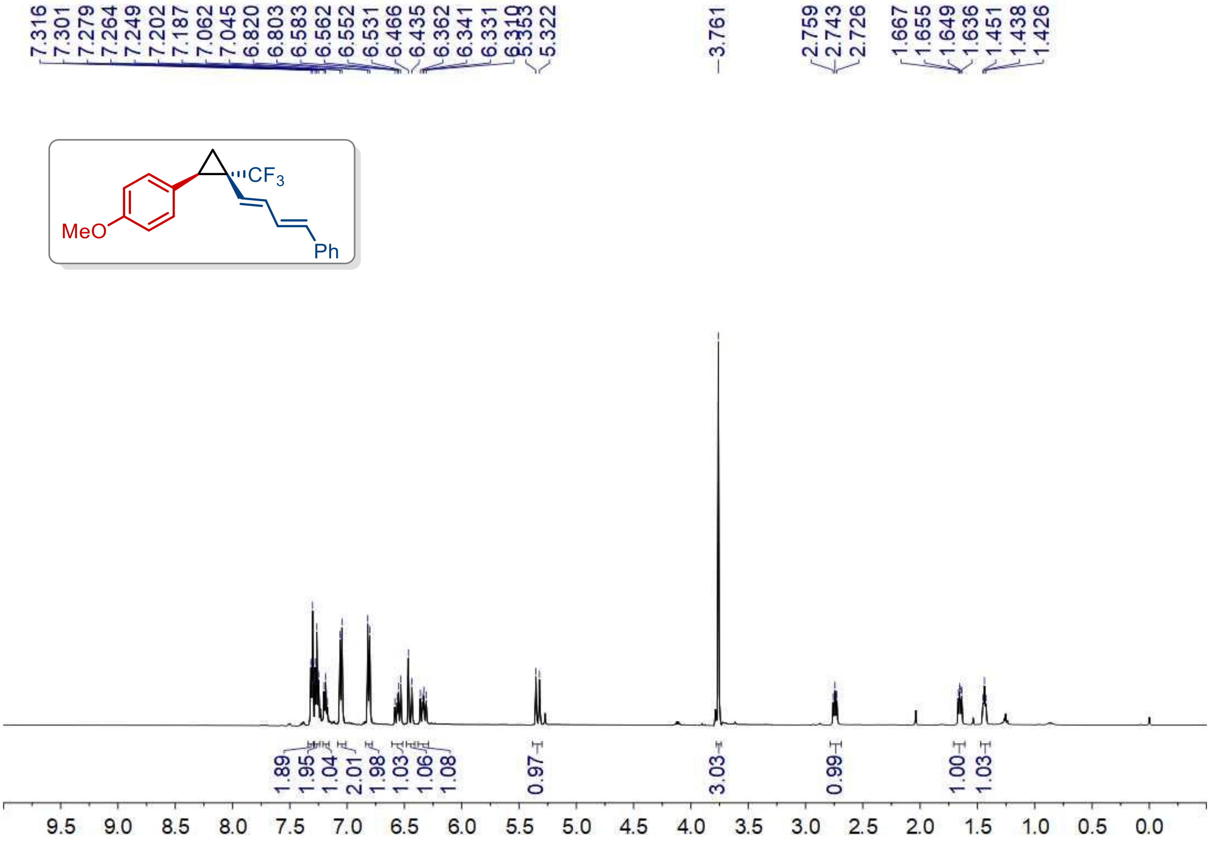

Supplementary Fig. 180 ^{1}**H NMR** (500 MHz, $CDCl_3$) spectrum of compound **62**.

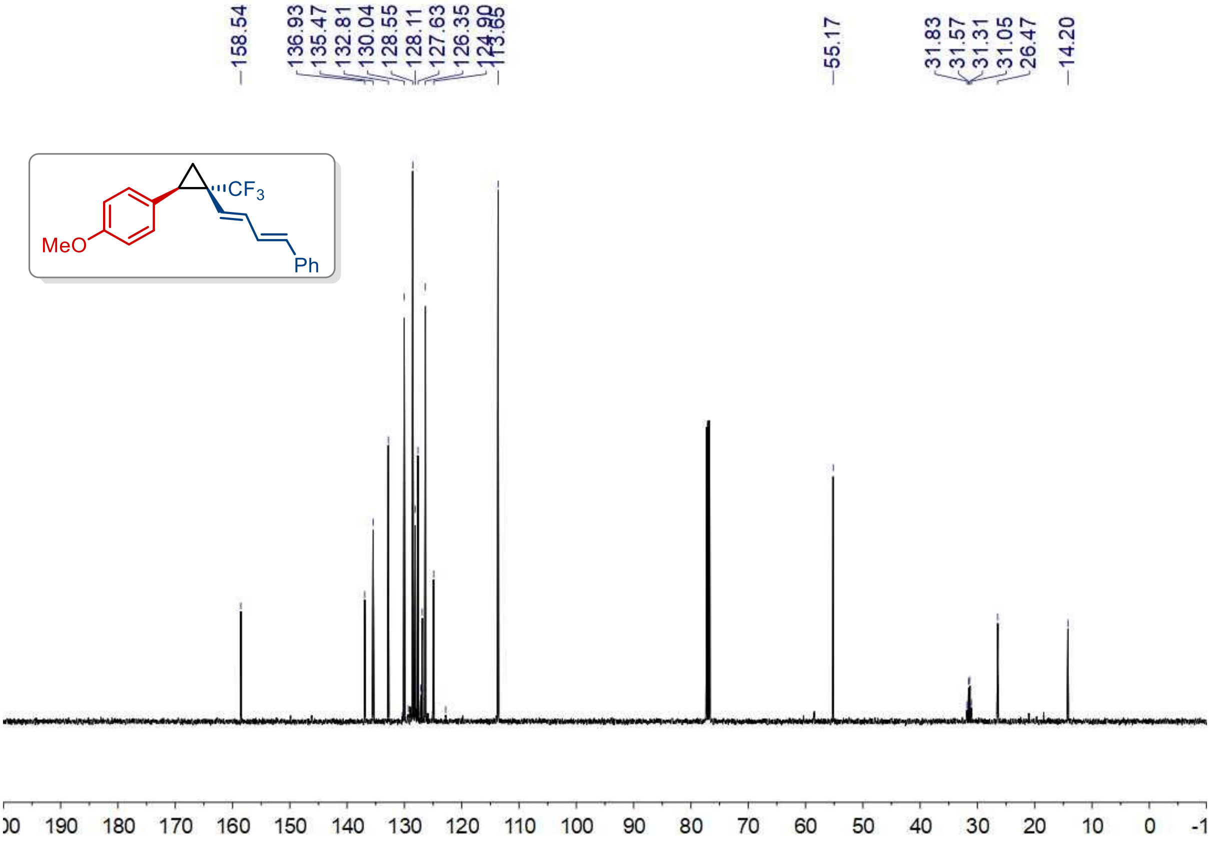

Supplementary Fig. 181 ^{13}**C NMR** (125 MHz, $CDCl_3$) spectrum of compound **62**.

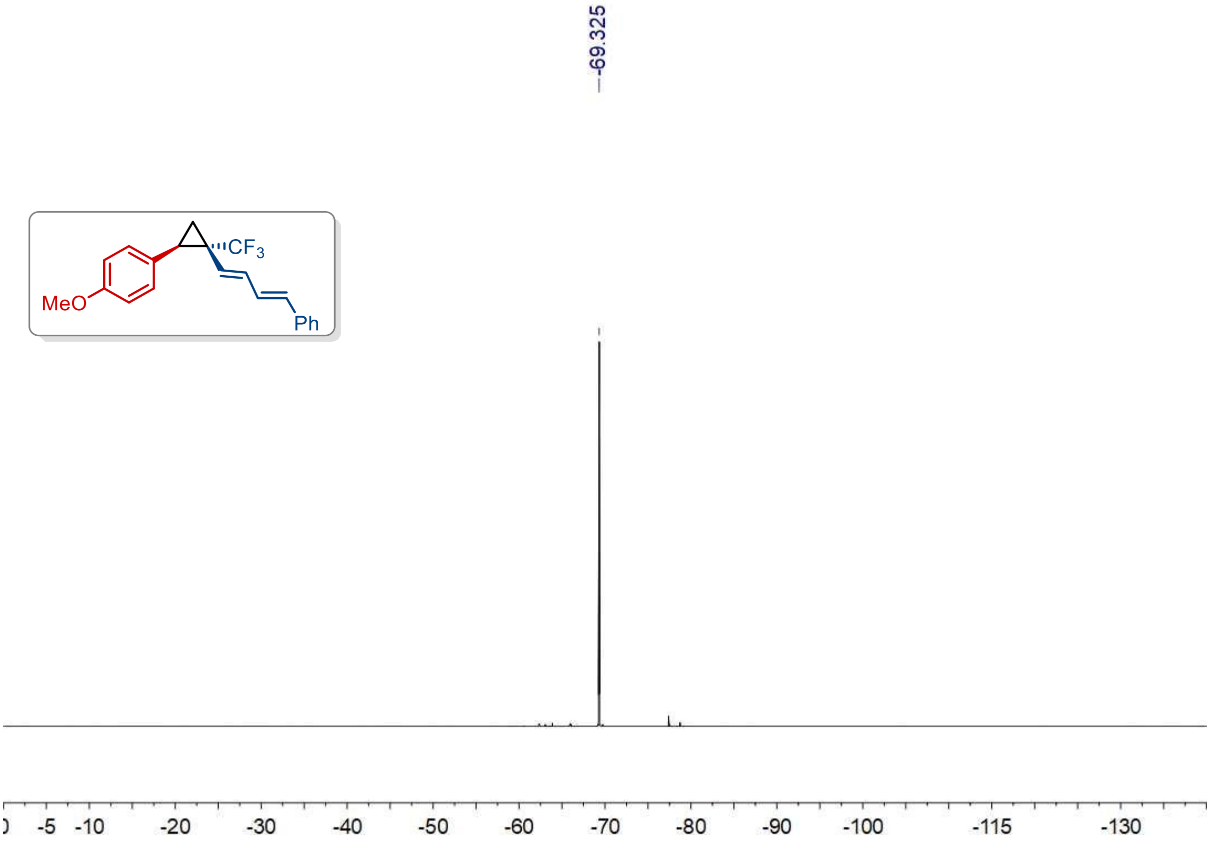

Supplementary Fig. 182 ^{19}**F NMR** (470 MHz, $CDCl_3$) spectrum of compound **62**.

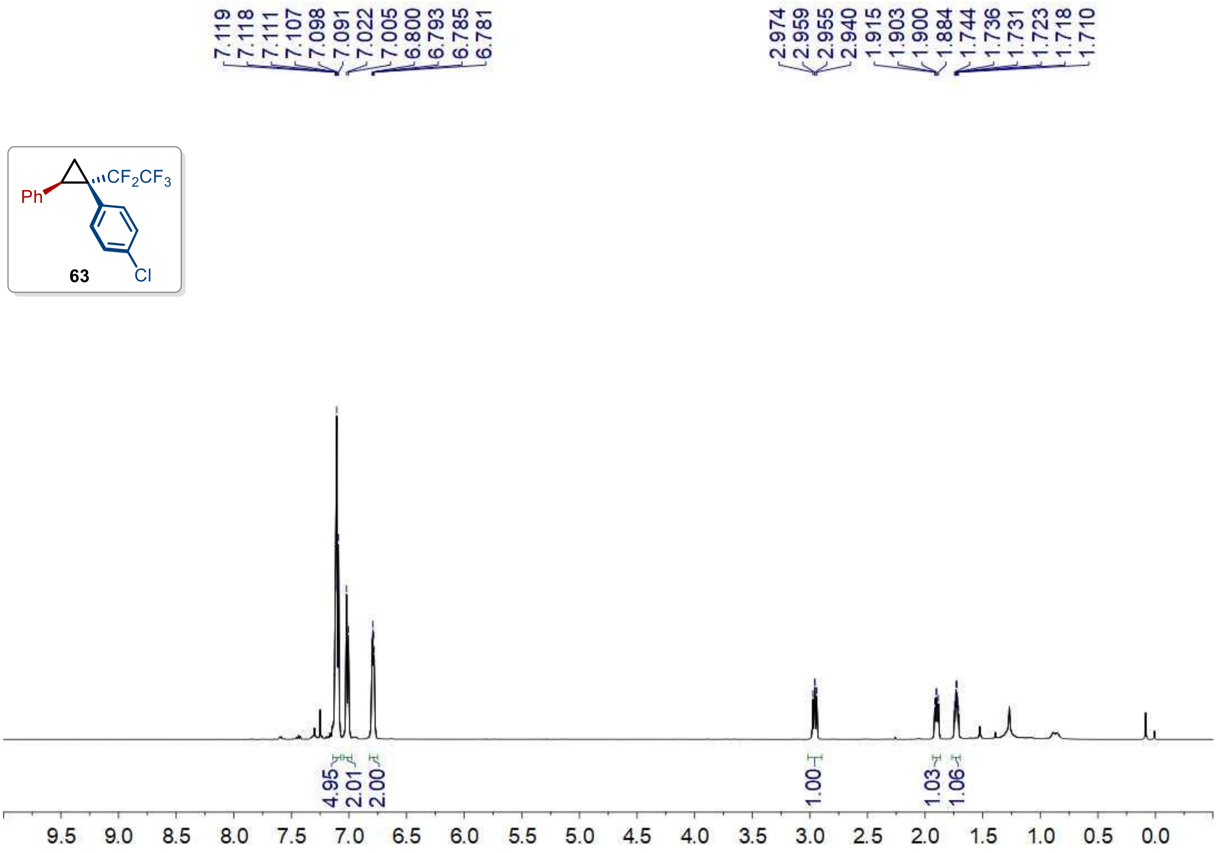

Supplementary Fig. 183 ^{1}**H NMR** (500 MHz, $CDCl_3$) spectrum of compound **63**.

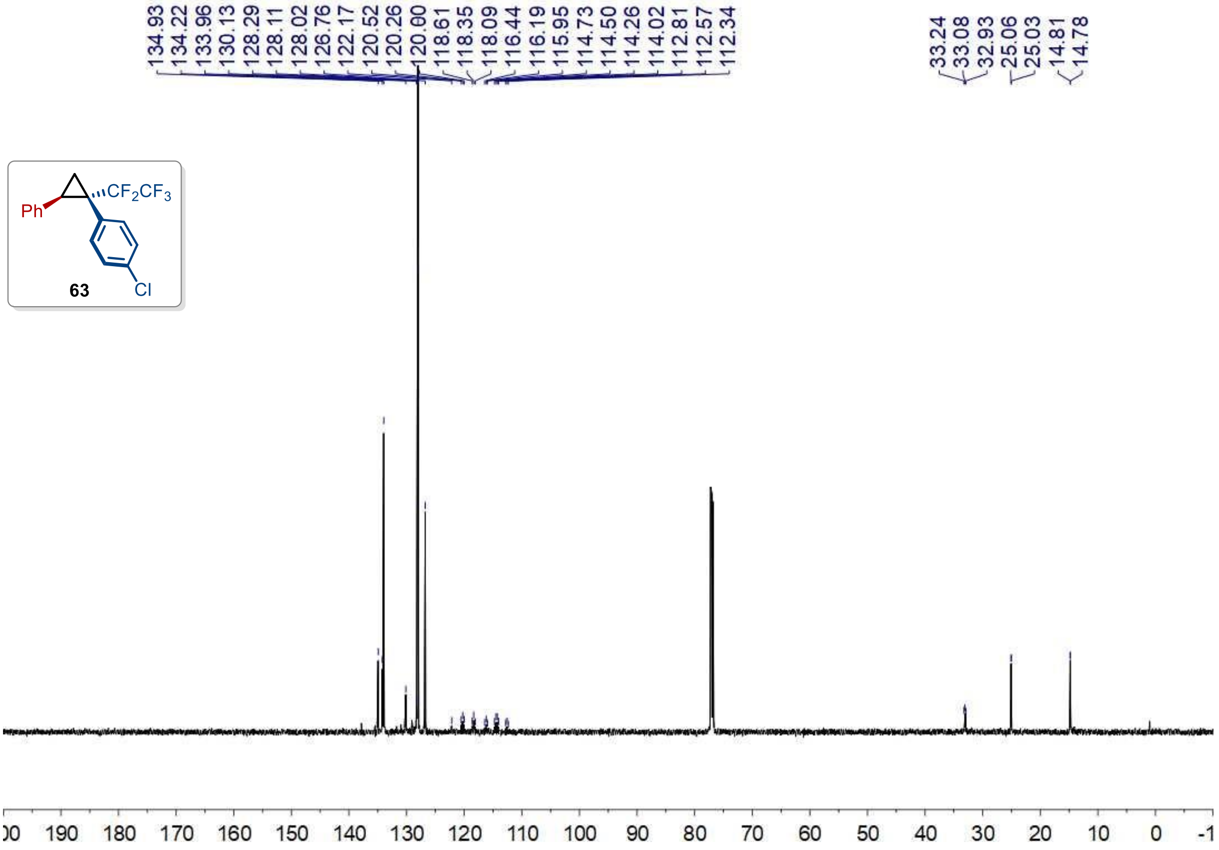

Supplementary Fig. 184 ^{13}**C NMR** (150 MHz, $CDCl_3$) spectrum of compound **63**.

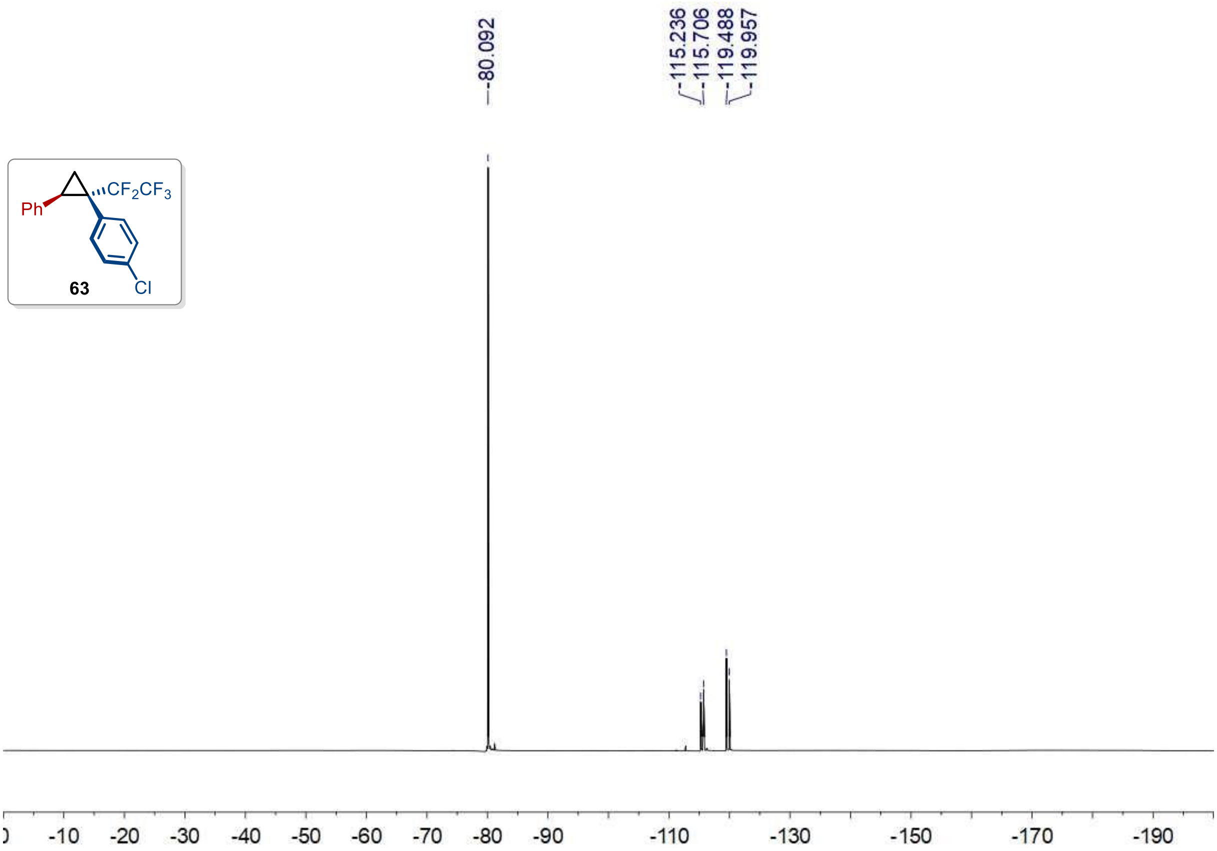

Supplementary Fig. 185 ^{19}**F NMR** (564 MHz, $CDCl_3$) spectrum of compound **63**.

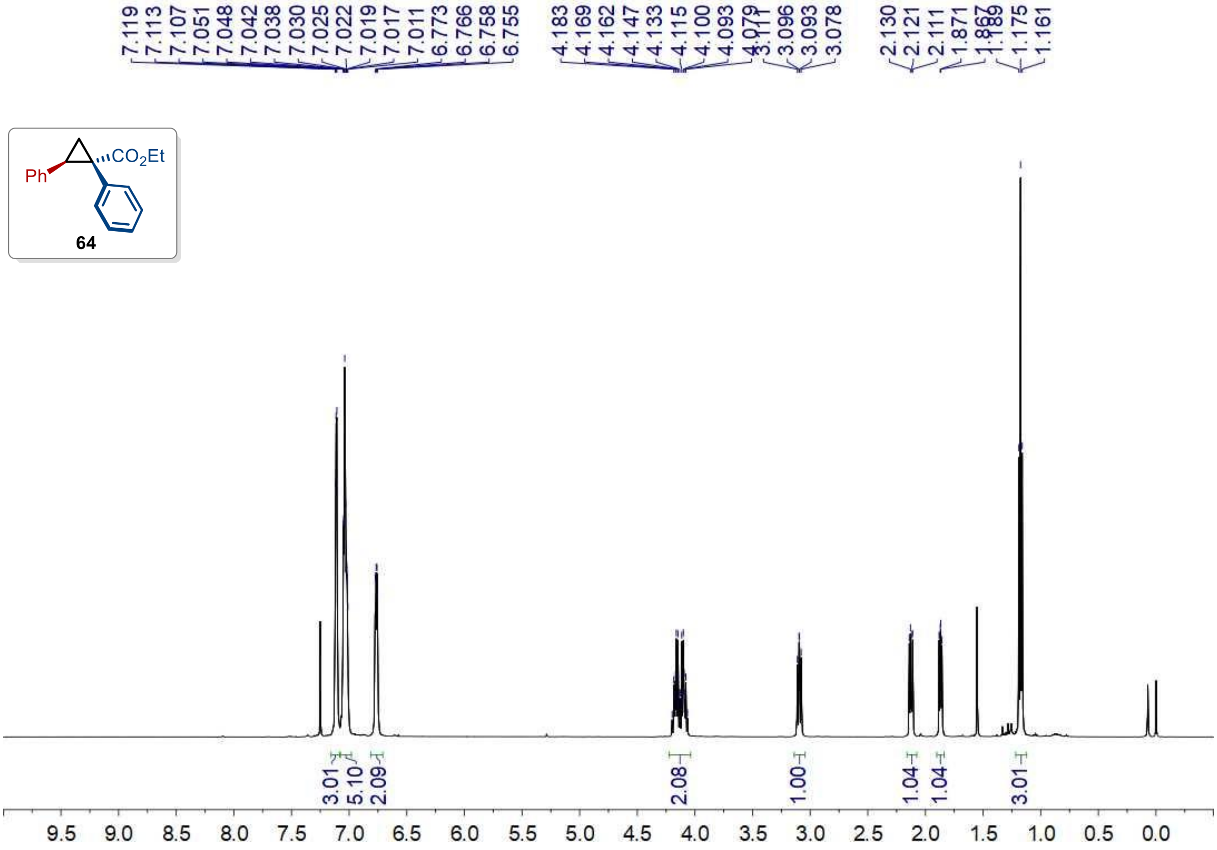

Supplementary Fig. 186 ^{1}**H NMR** (500 MHz, $CDCl_3$) spectrum of compound **64**.

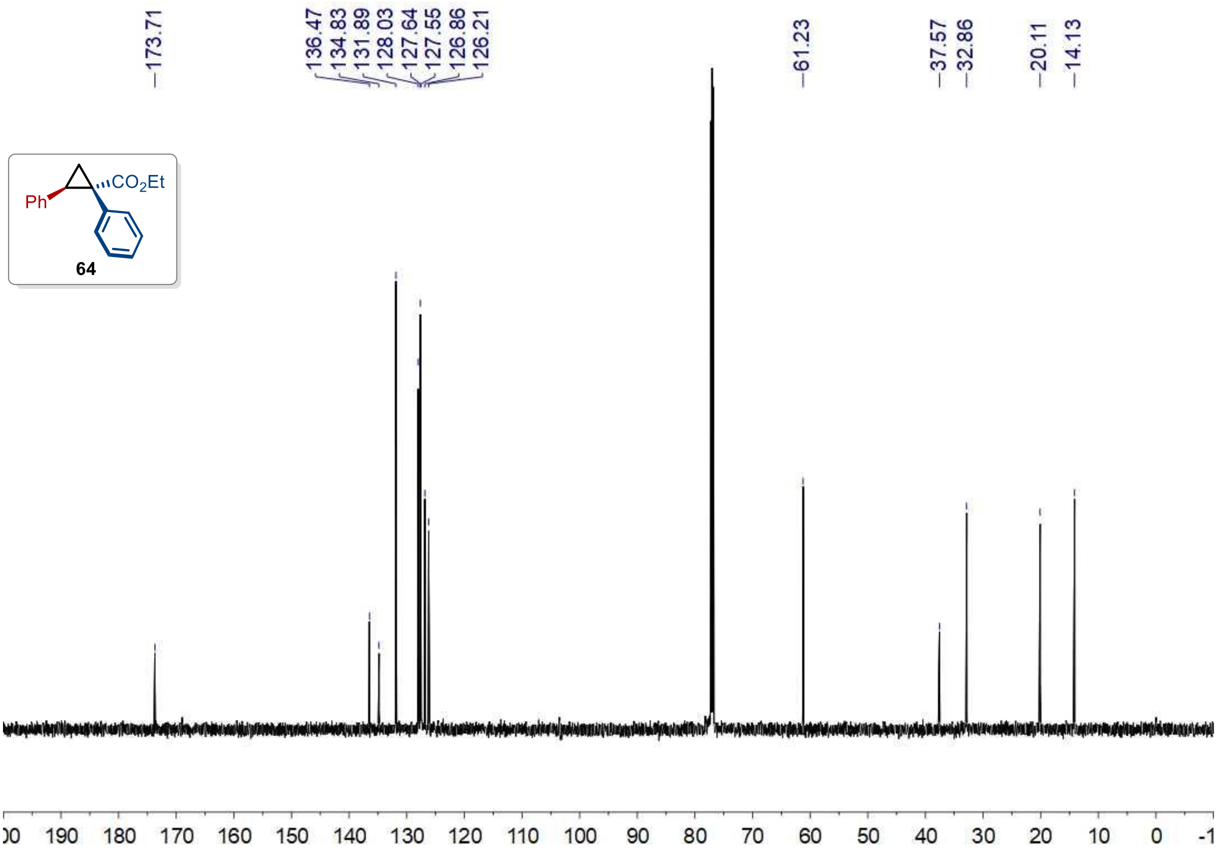

Supplementary Fig. 187 ^{13}**C NMR** (150 MHz, $CDCl_3$) spectrum of compound **64**.

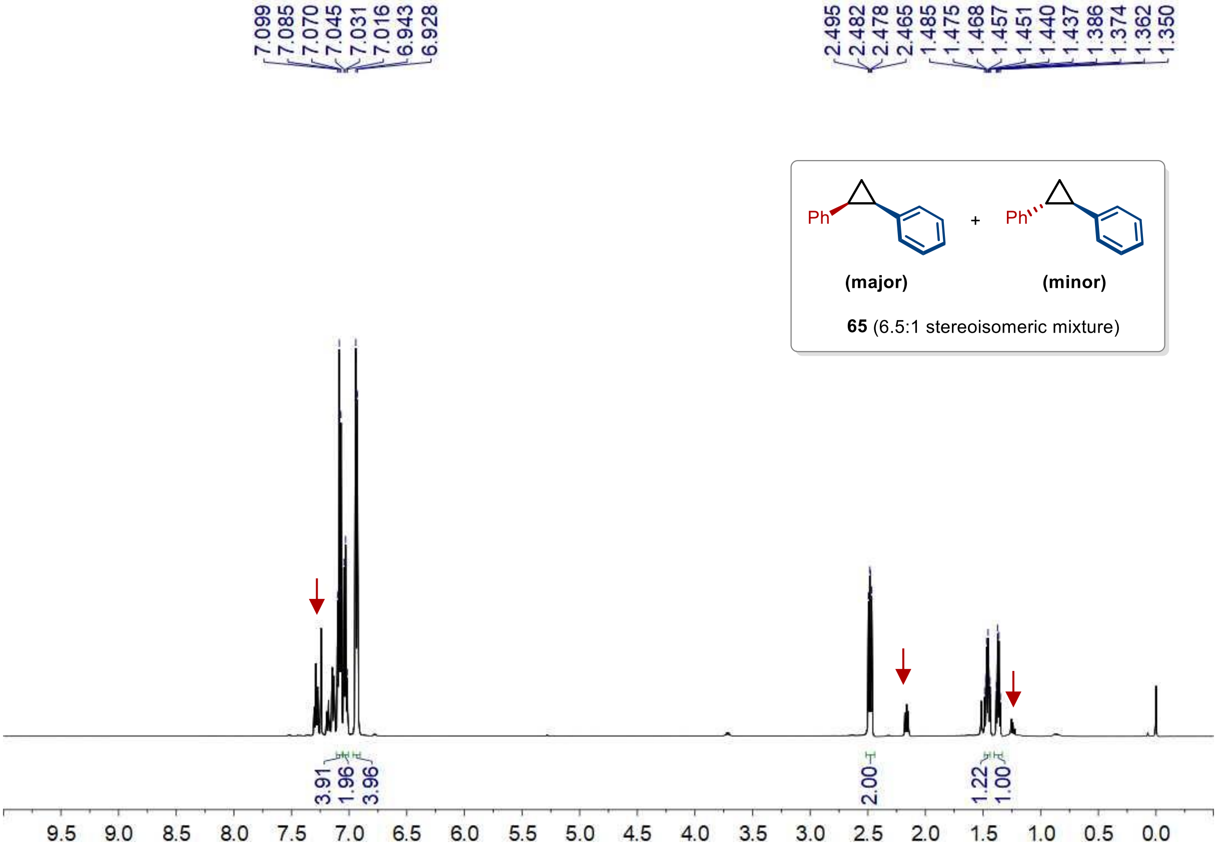

Supplementary Fig. 188 ^{1}**H NMR** (500 MHz, $CDCl_3$) spectrum of compound **65**.

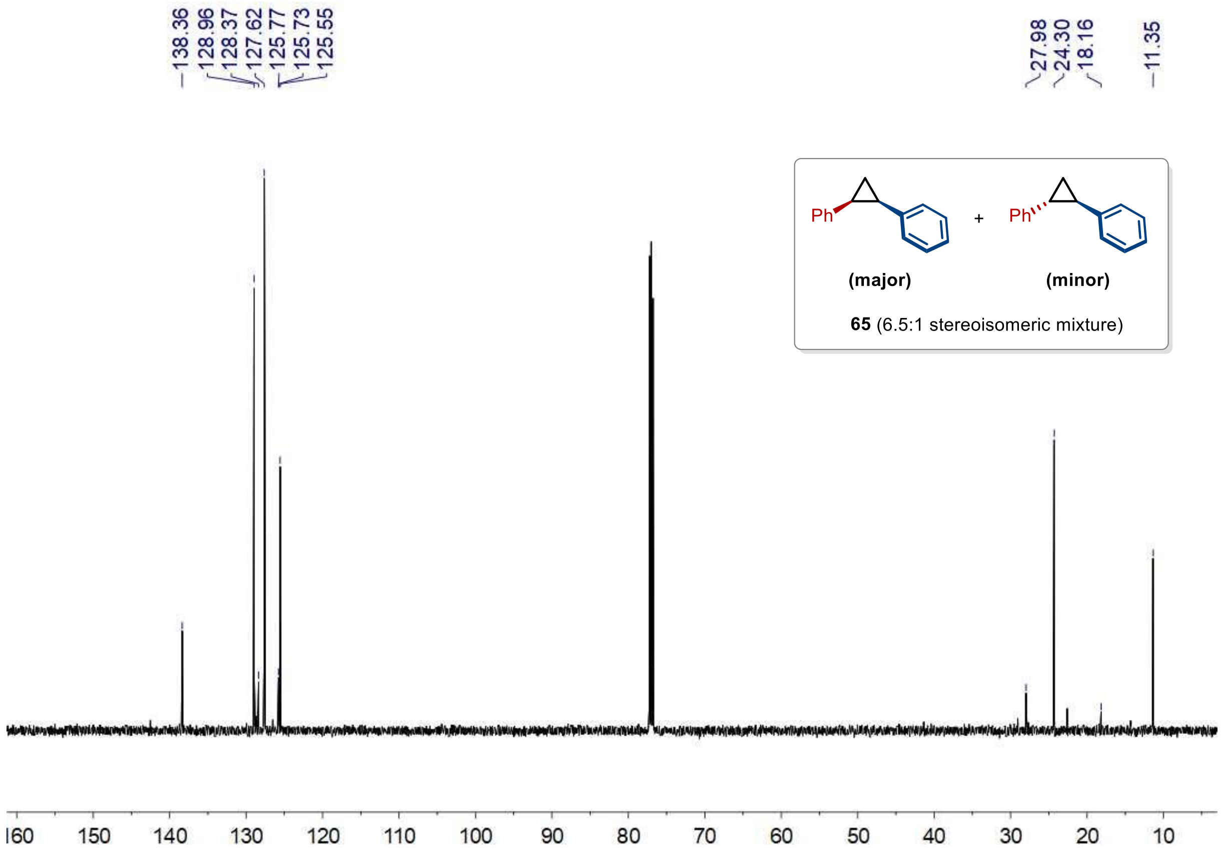

Supplementary Fig. 189 ^{13}**C NMR** (150 MHz, $CDCl_3$) spectrum of compound **65**.

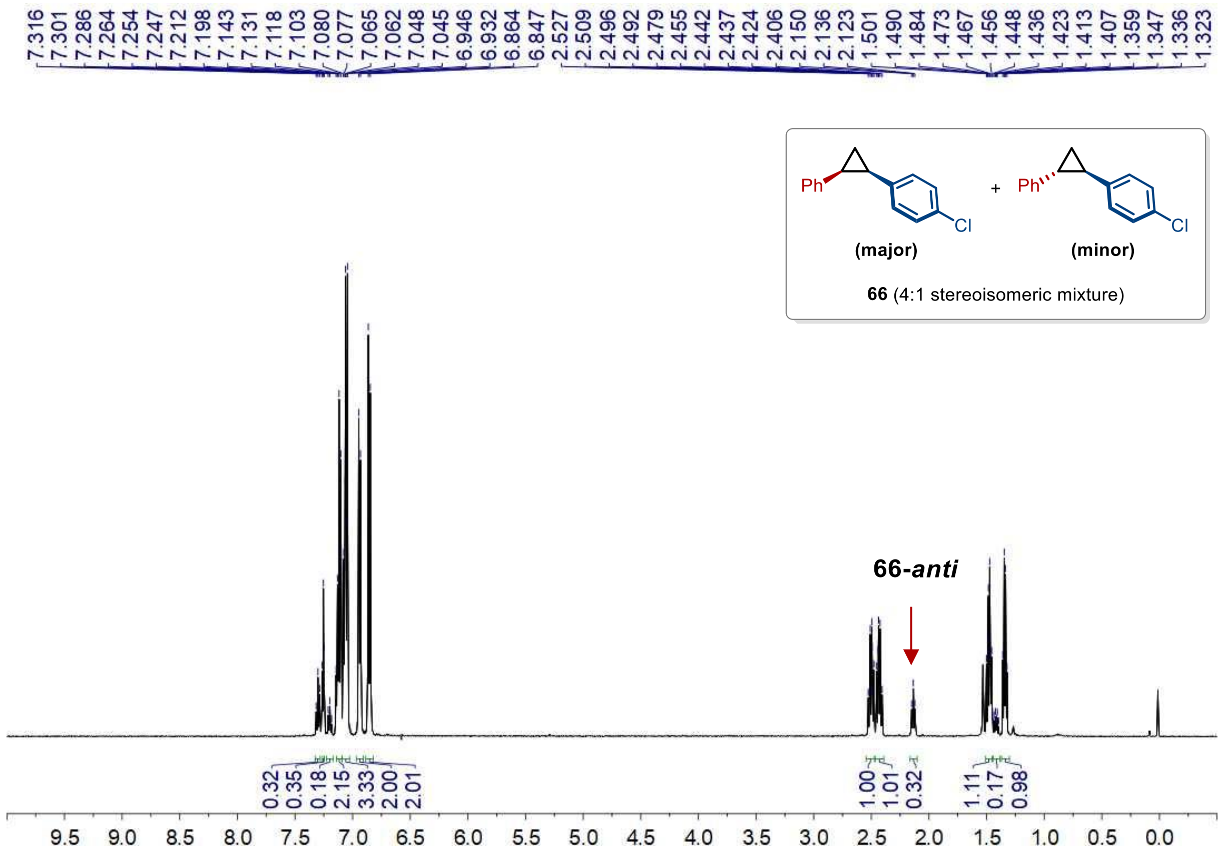

Supplementary Fig. 190 ^{1}**H NMR** (500 MHz, $CDCl_3$) spectrum of compound **66**.

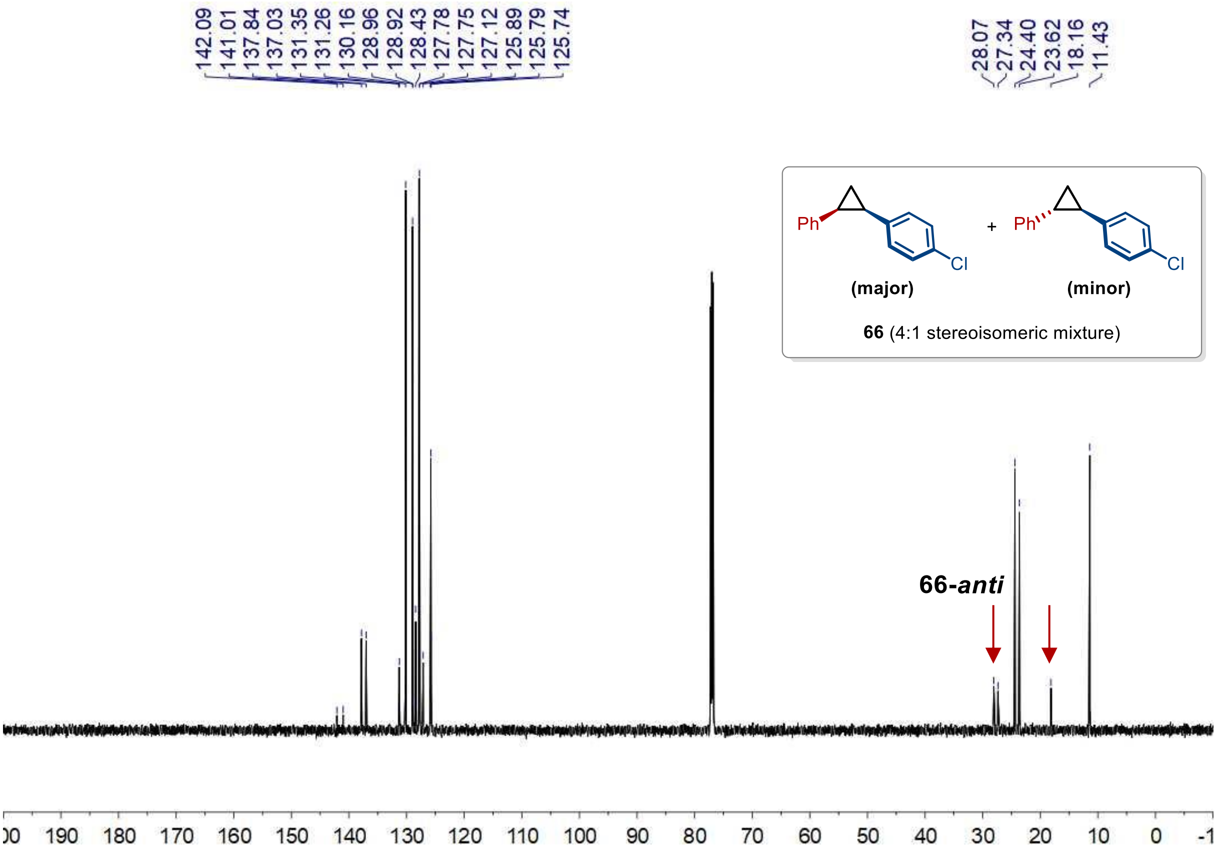

Supplementary Fig. 191 ^{13}**C NMR** (150 MHz, $CDCl_3$) spectrum of compound **66**.

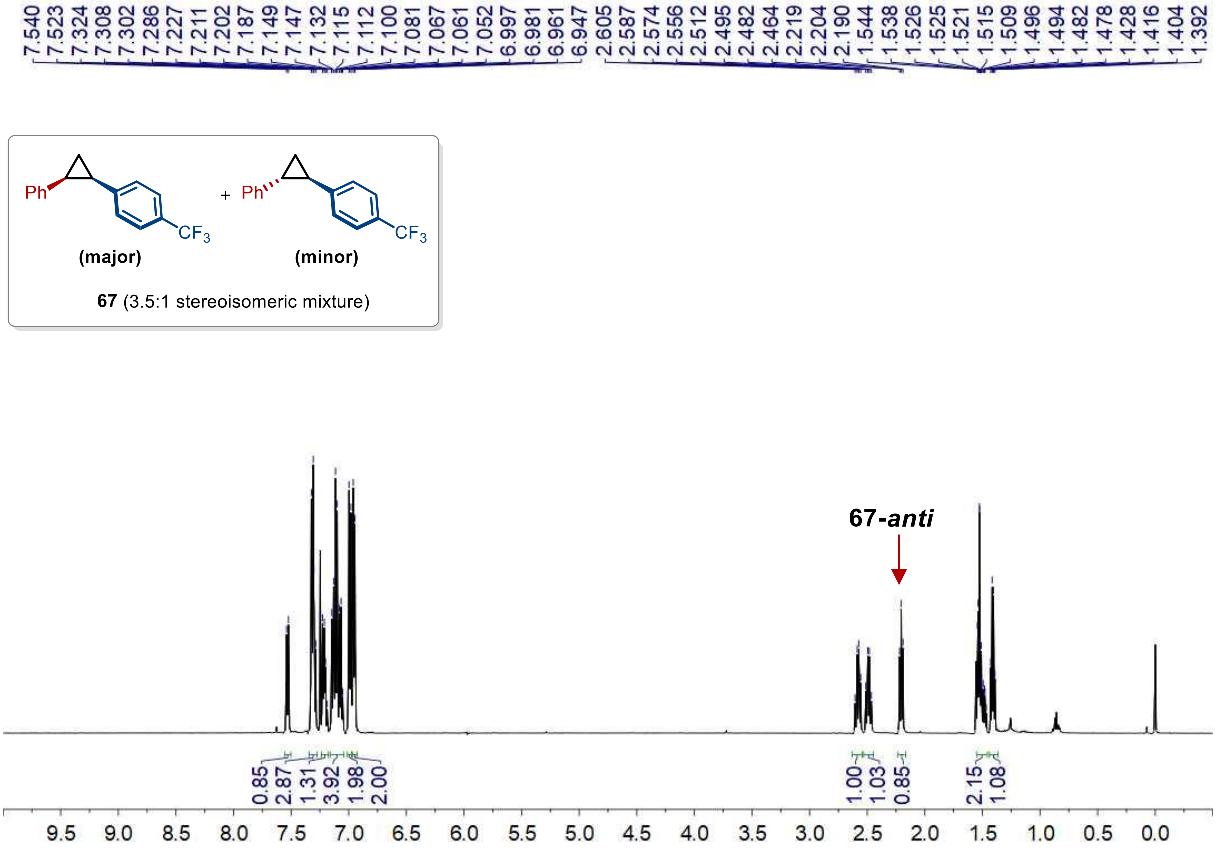

Supplementary Fig. 192 ^{1}**H NMR** (500 MHz, $CDCl_3$) spectrum of compound **67**.

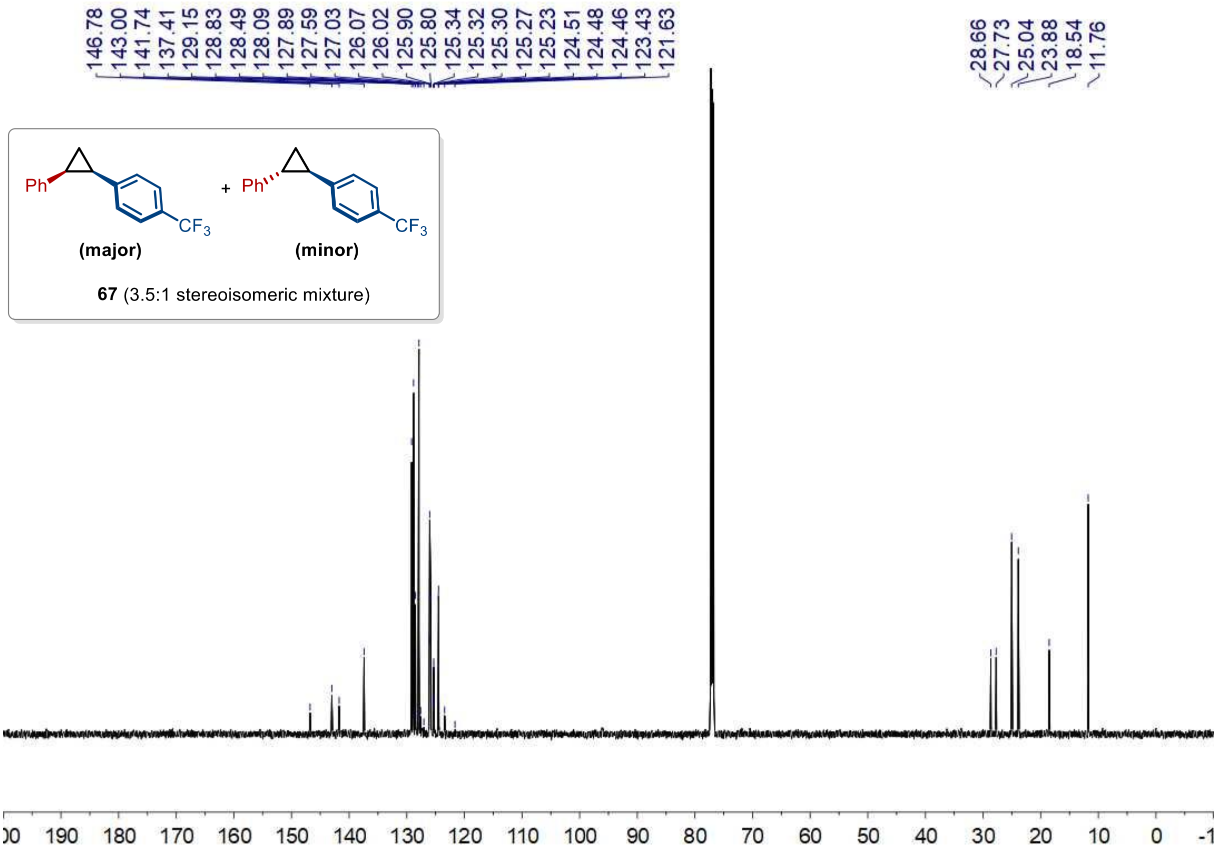

Supplementary Fig. 193 ^{13}**C NMR** (150 MHz, $CDCl_3$) spectrum of compound **67**.

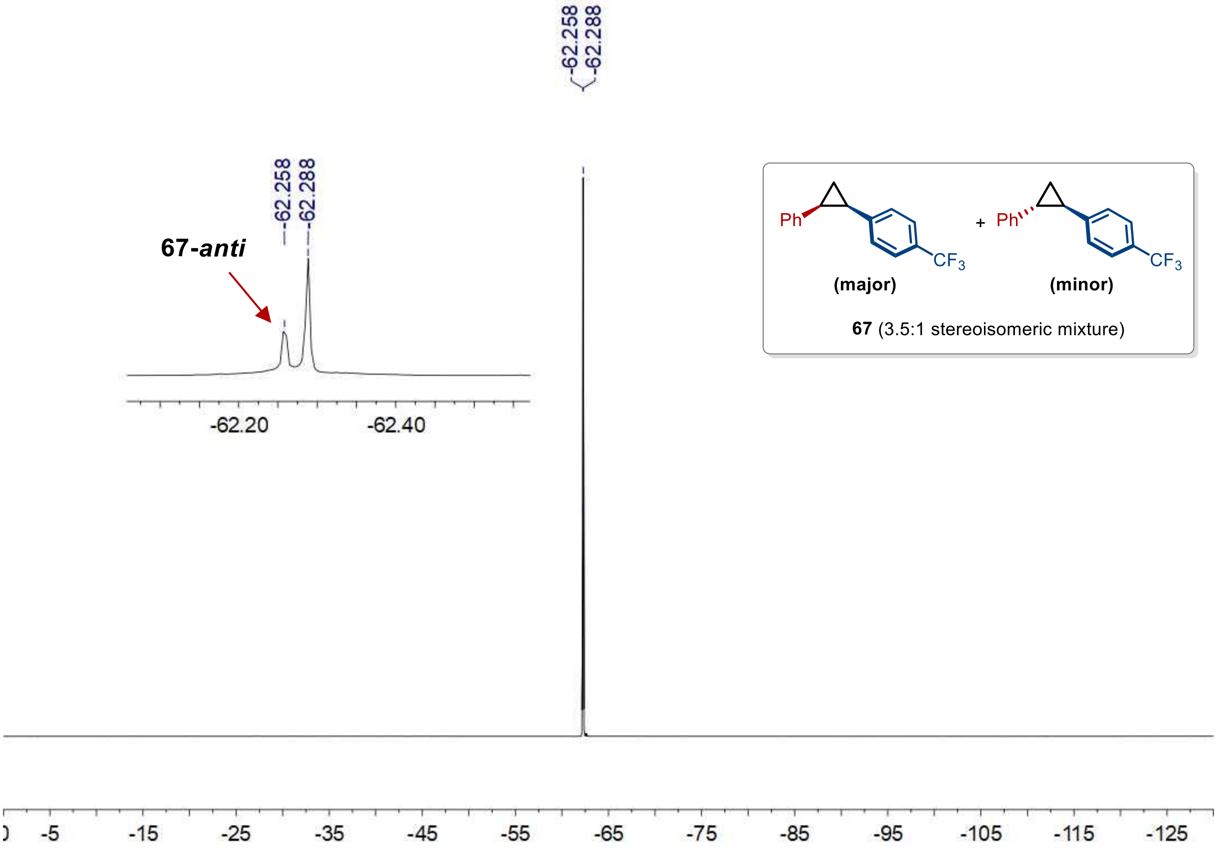

Supplementary Fig. 194 ^{19}**F NMR** (564 MHz, $CDCl_3$) spectrum of compound **67**.

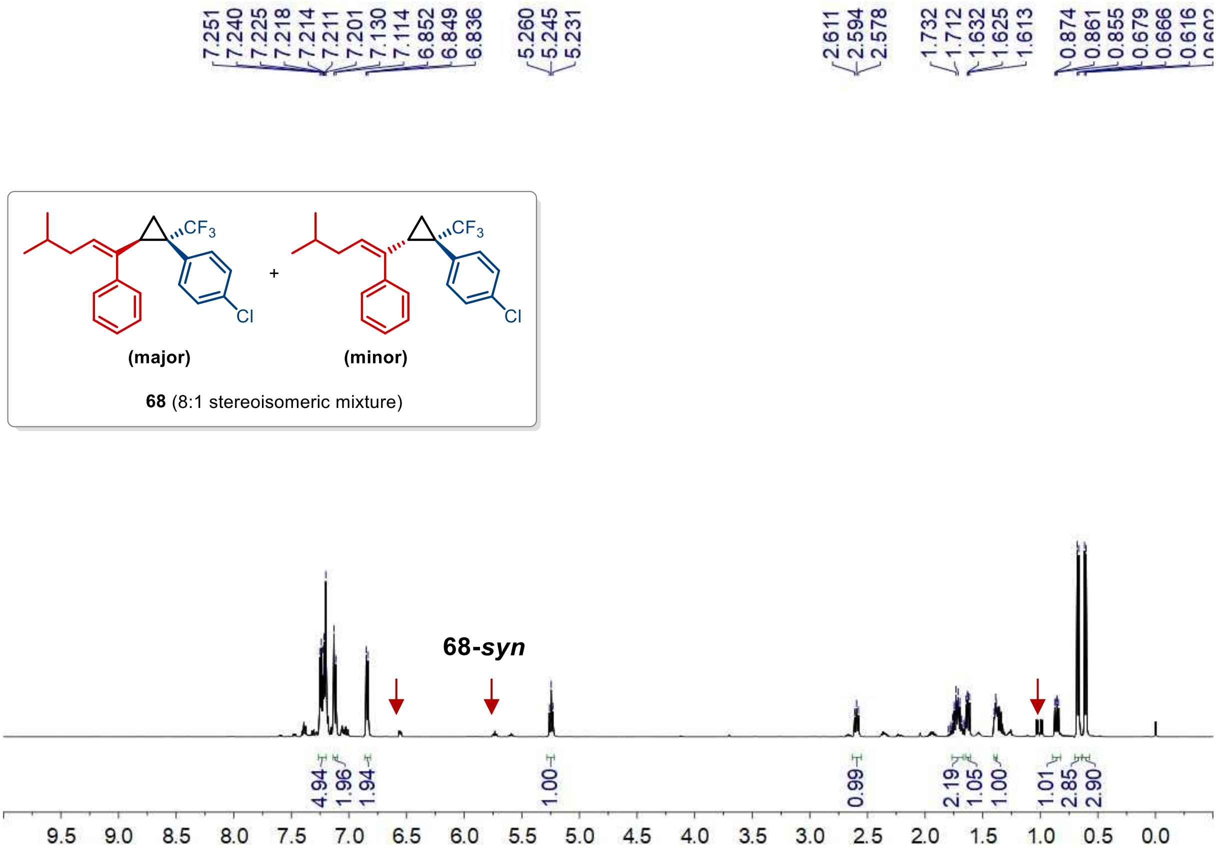

Supplementary Fig. 195 ^{1}H NMR (500 MHz, $CDCl_3$) spectrum of compound **68**.

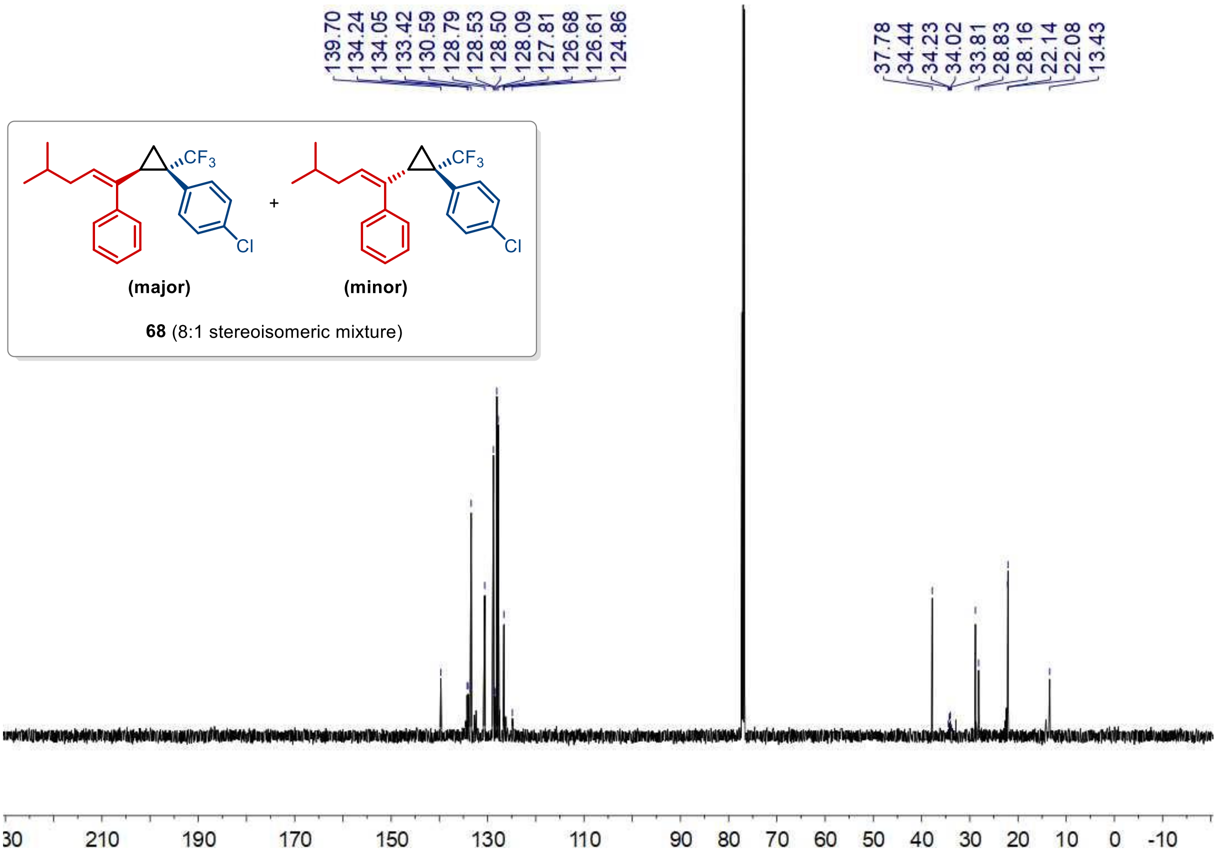

Supplementary Fig. 196 ^{13}C NMR (150 MHz, $CDCl_3$) spectrum of compound **68**.

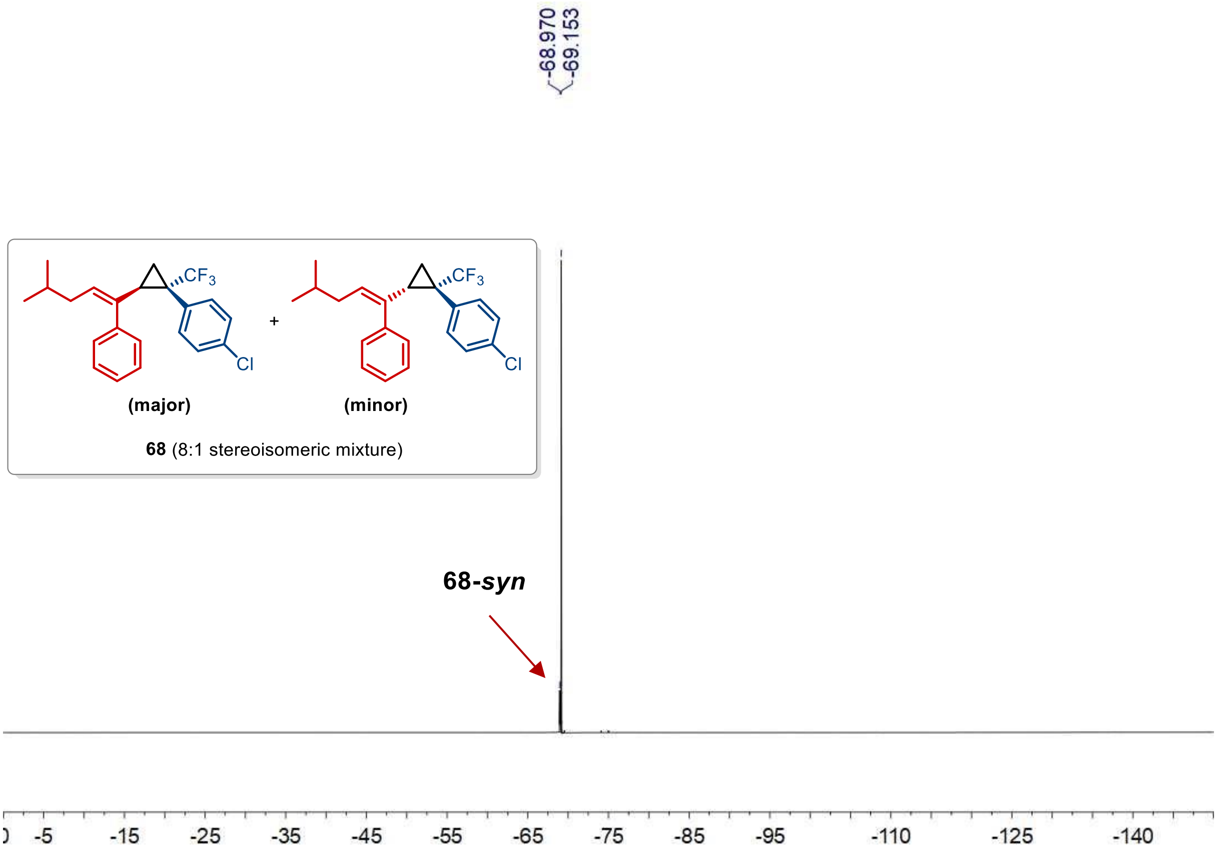

Supplementary Fig. 197 ^{19}**F NMR** (564 MHz, $CDCl_3$) spectrum of compound **68**.

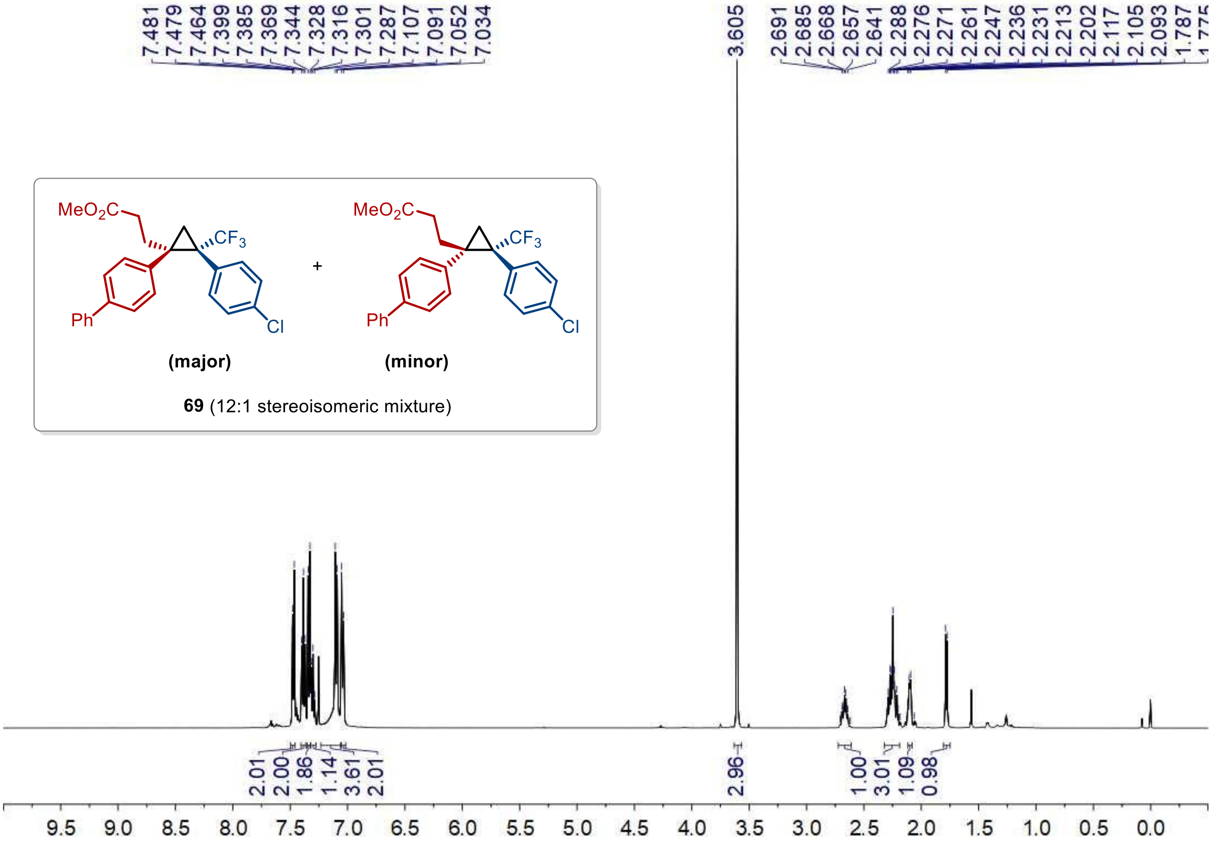

Supplementary Fig. 198 ^{1}**H NMR** (500 MHz, $CDCl_3$) spectrum of compound **69**.

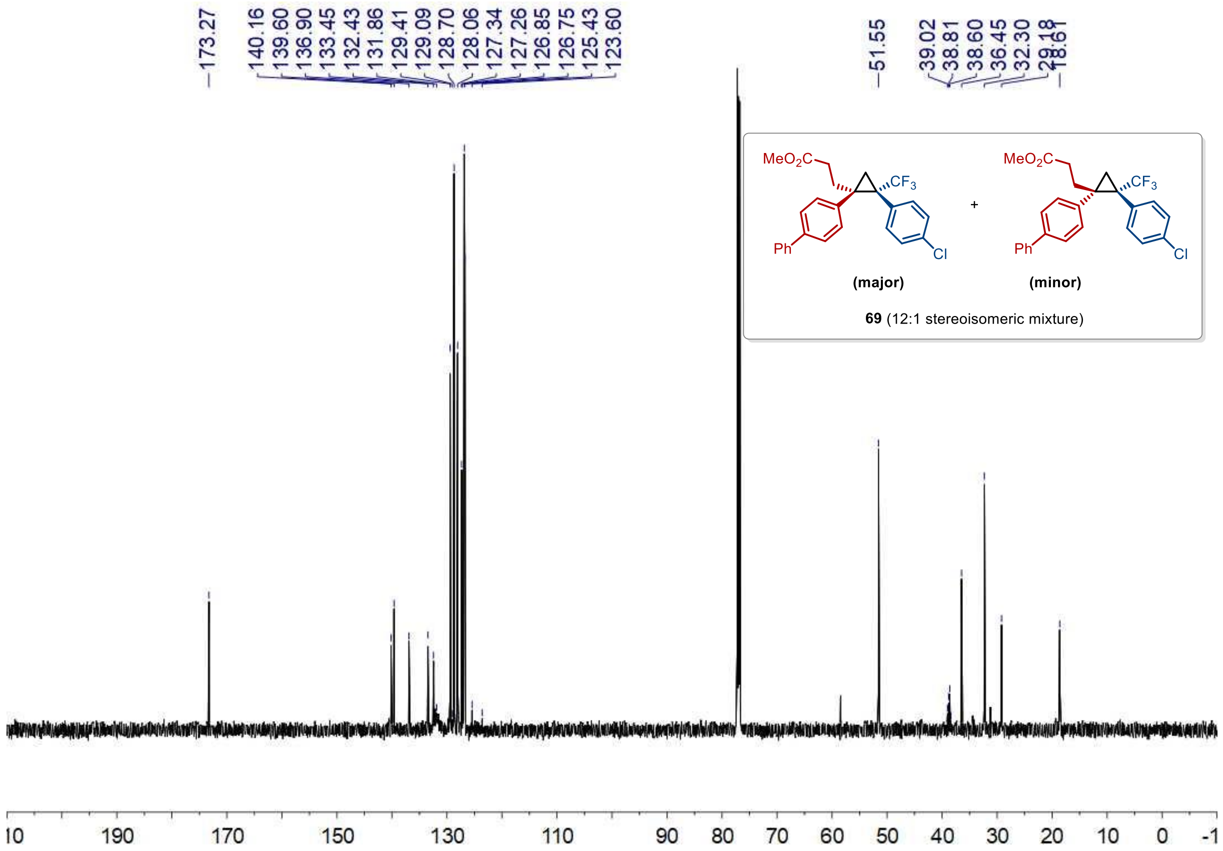

Supplementary Fig. 199 ^{13}**C NMR** (150 MHz, $CDCl_3$) spectrum of compound **69**.

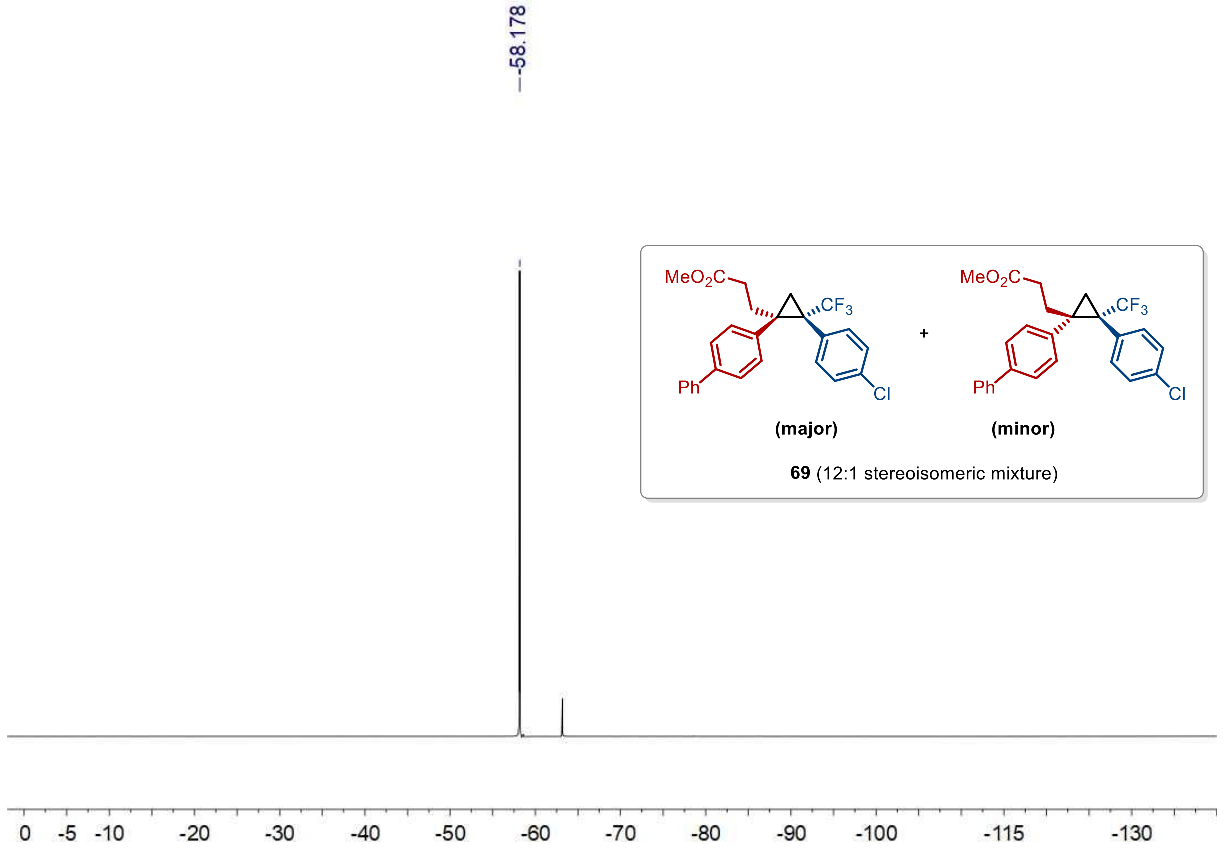

Supplementary Fig. 200 ^{19}**F NMR** (564 MHz, $CDCl_3$) spectrum of compound **69**.

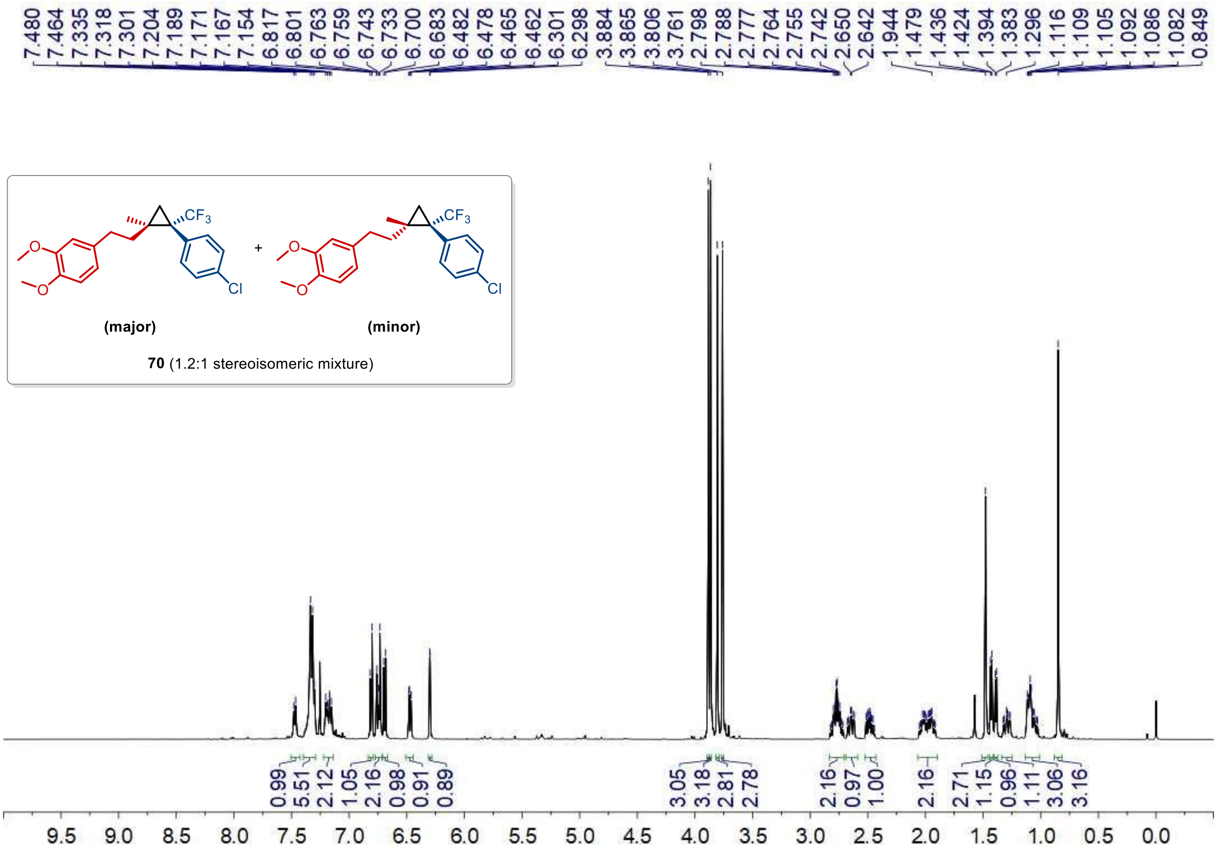

Supplementary Fig. 201 ^{1}**H NMR** (500 MHz, $CDCl_3$) spectrum of compound **70**.

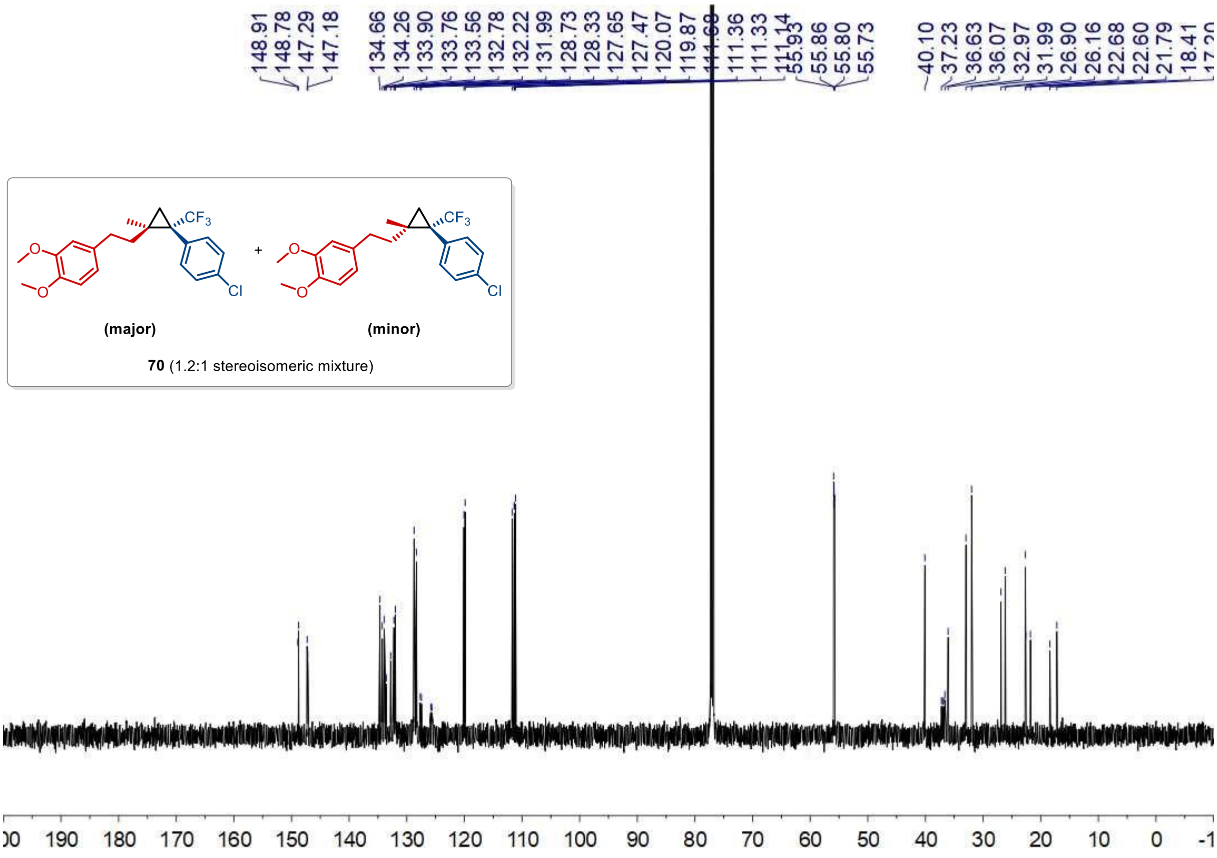

Supplementary Fig. 202 ^{13}**C NMR** (150 MHz, $CDCl_3$) spectrum of compound **70**.

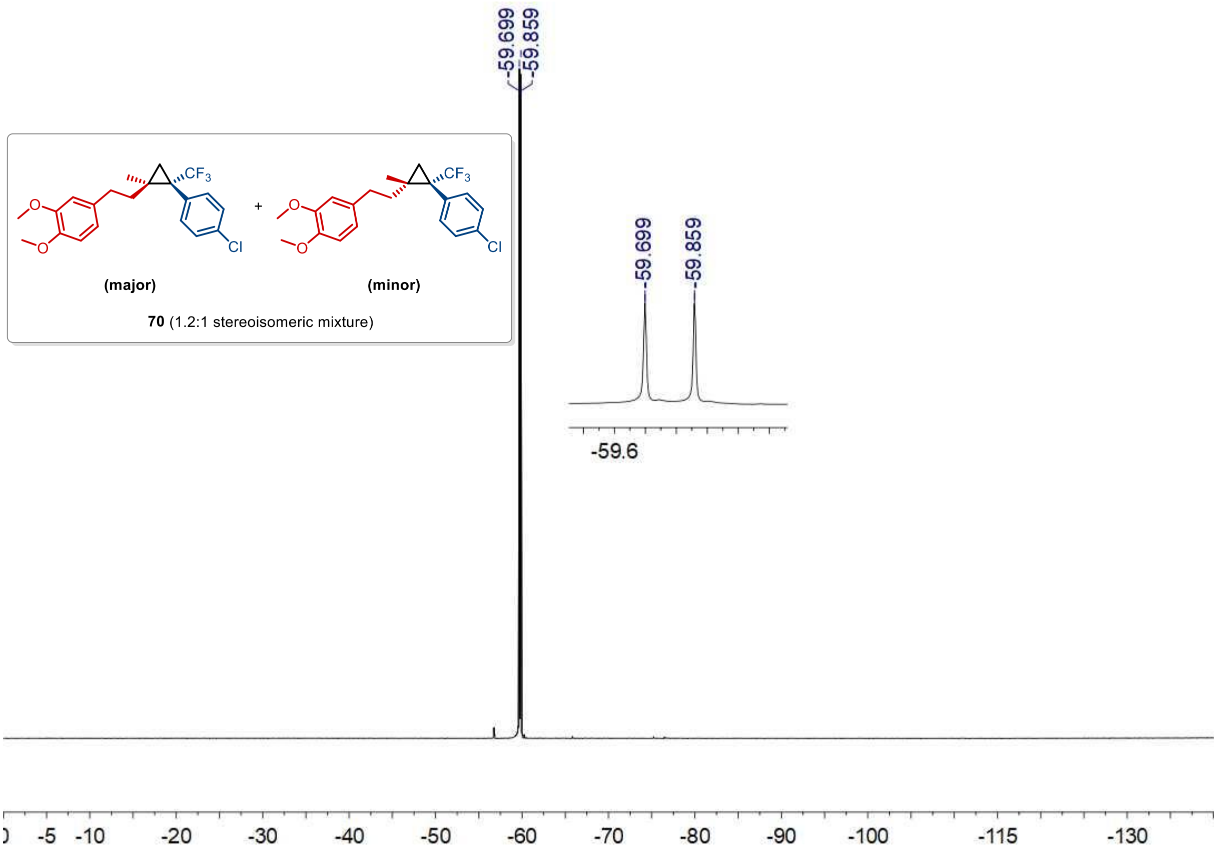

Supplementary Fig. 203 ^{19}**F NMR** (564 MHz, $CDCl_3$) spectrum of compound **70**.

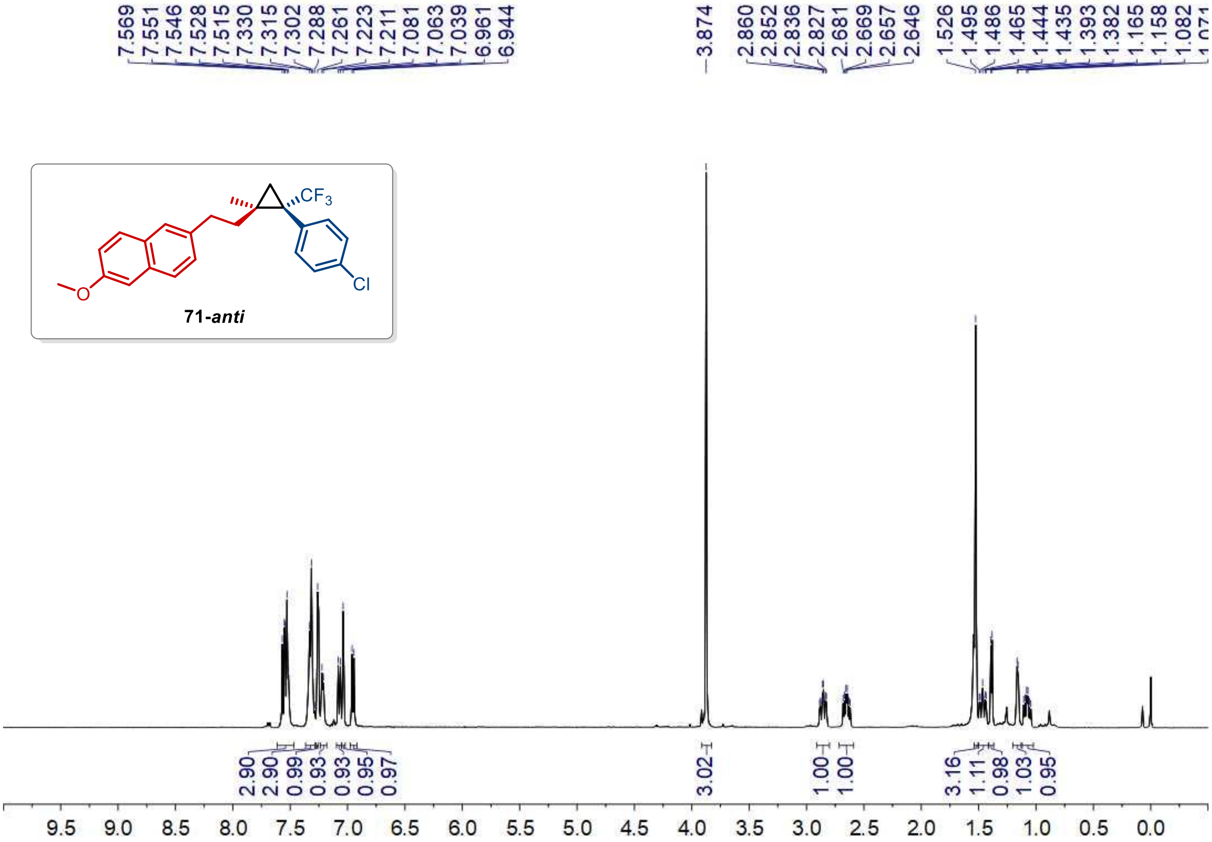

Supplementary Fig. 204 ^{1}**H NMR** (500 MHz, $CDCl_3$) spectrum of compound **71-*anti***.

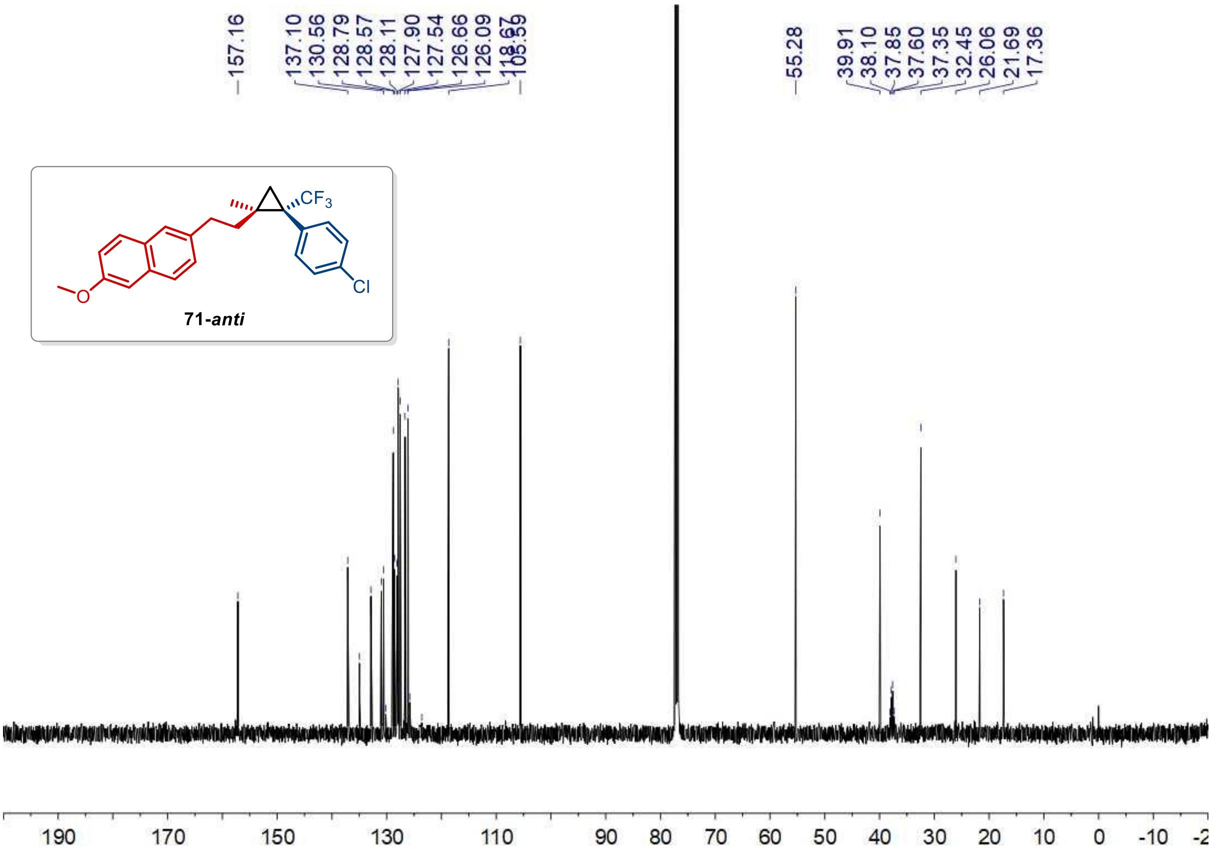

Supplementary Fig. 205 ^{13}**C NMR** (125 MHz, $CDCl_3$) spectrum of compound **71-*anti***.

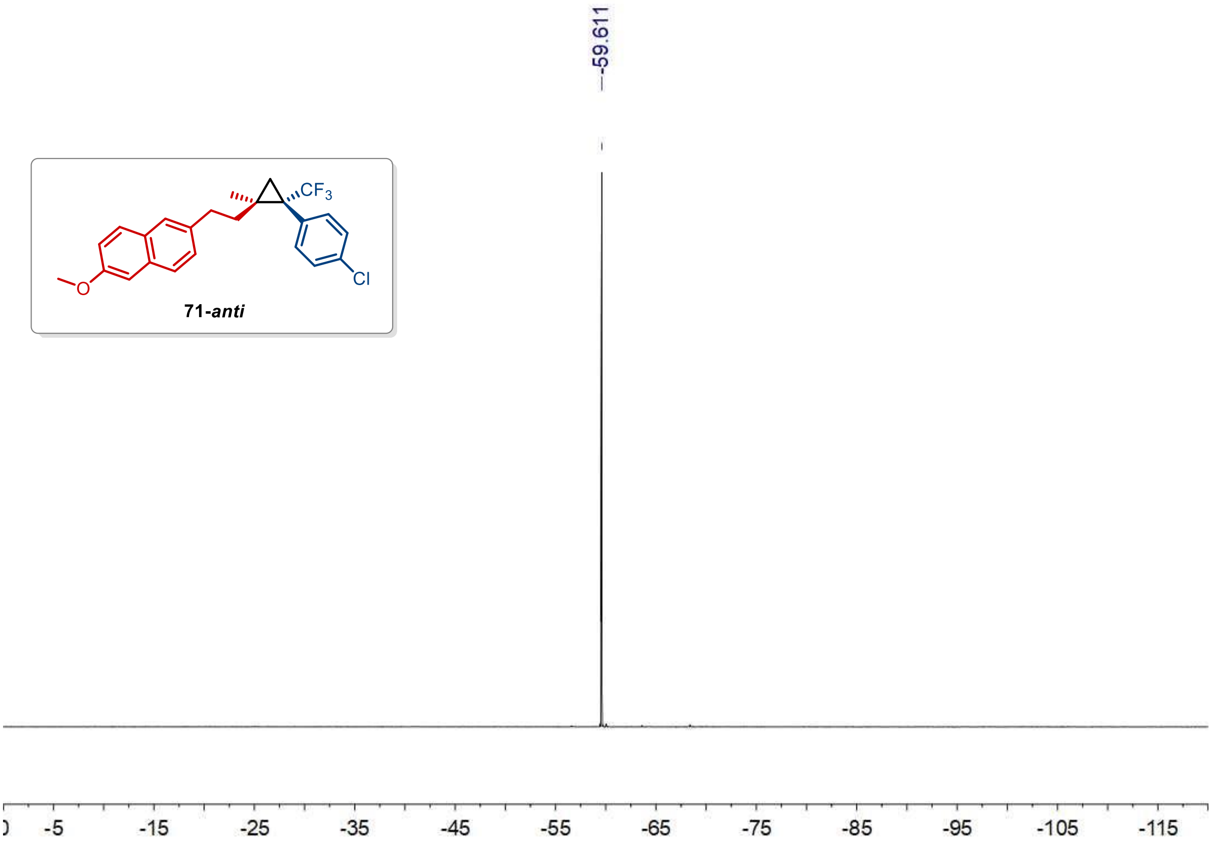

Supplementary Fig. 206 ^{19}**F NMR** (470 MHz, $CDCl_3$) spectrum of compound **71-*anti***.

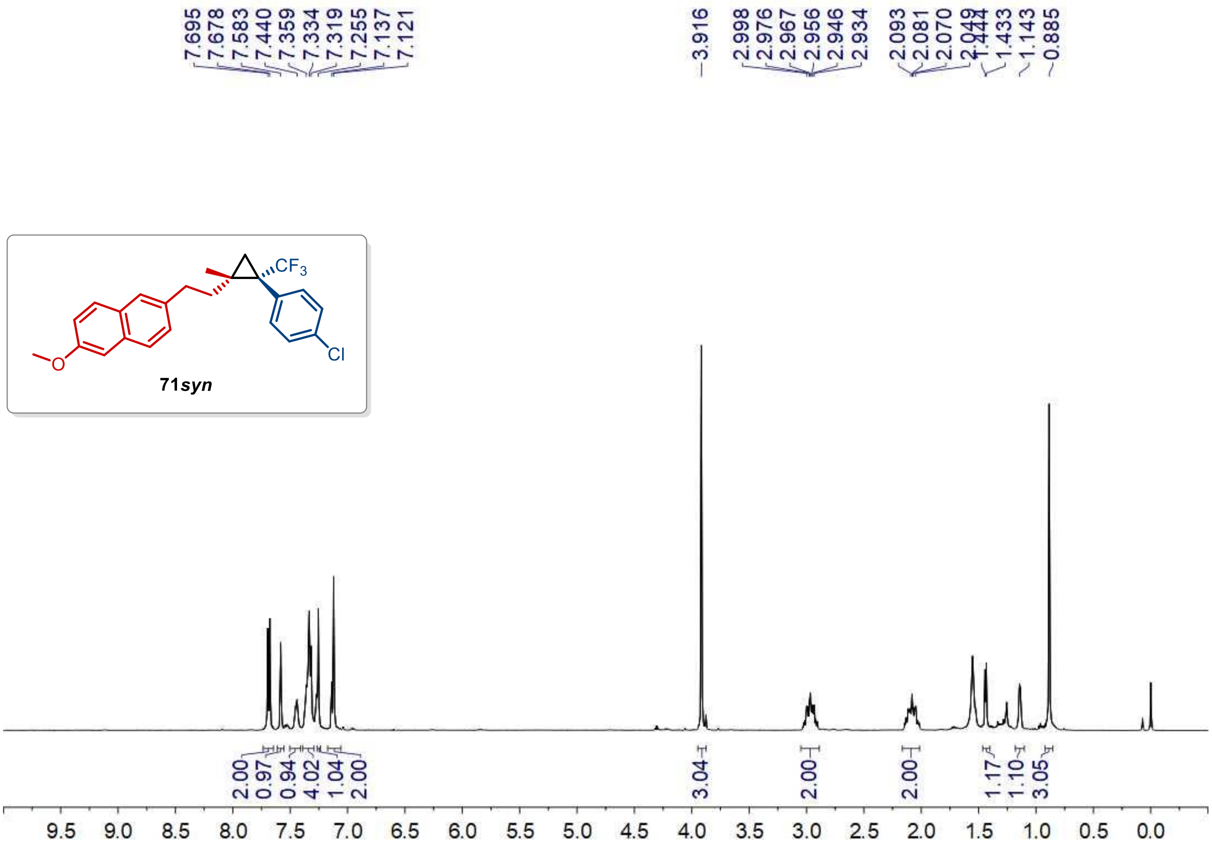

Supplementary Fig. 207 ^{1}**H NMR** (500 MHz, $CDCl_3$) spectrum of compound **71-*syn***.

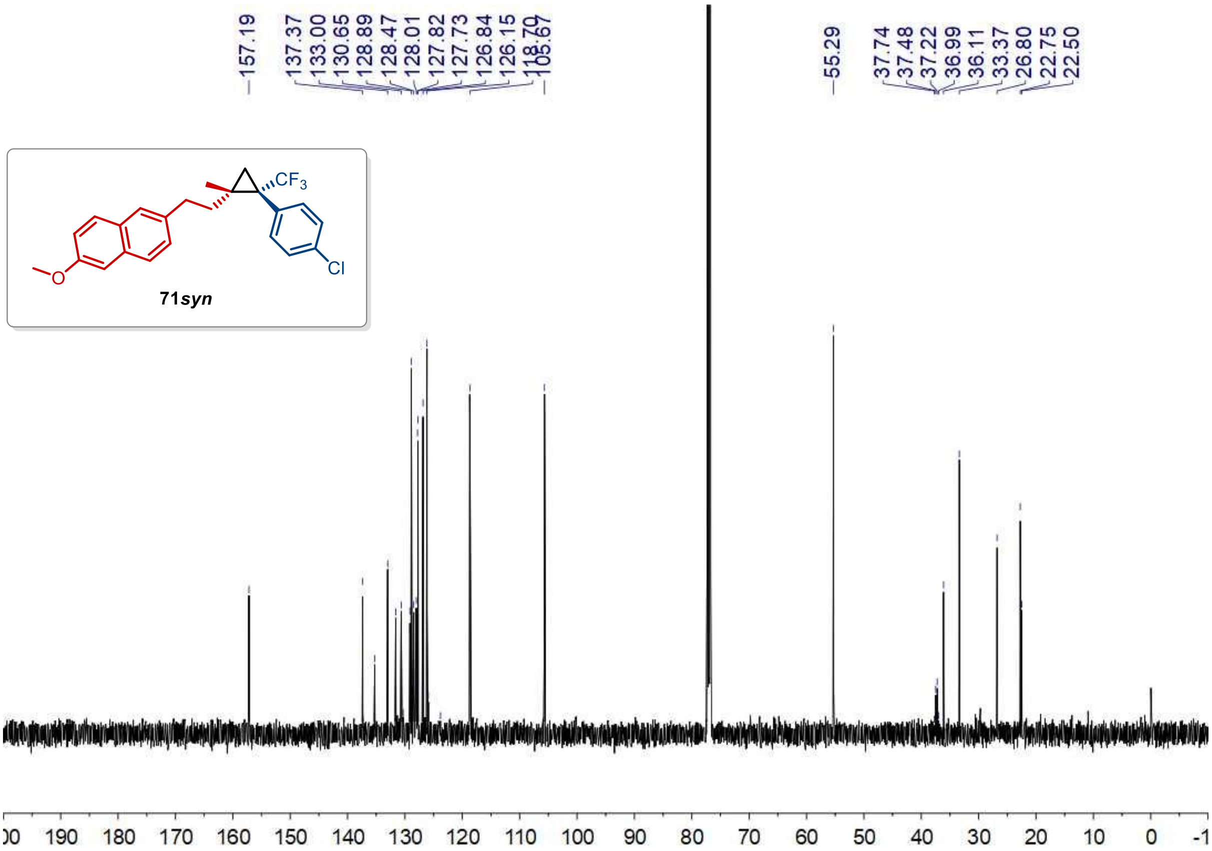

Supplementary Fig. 208 ^{13}**C NMR** (125 MHz, $CDCl_3$) spectrum of compound **71-*syn***.

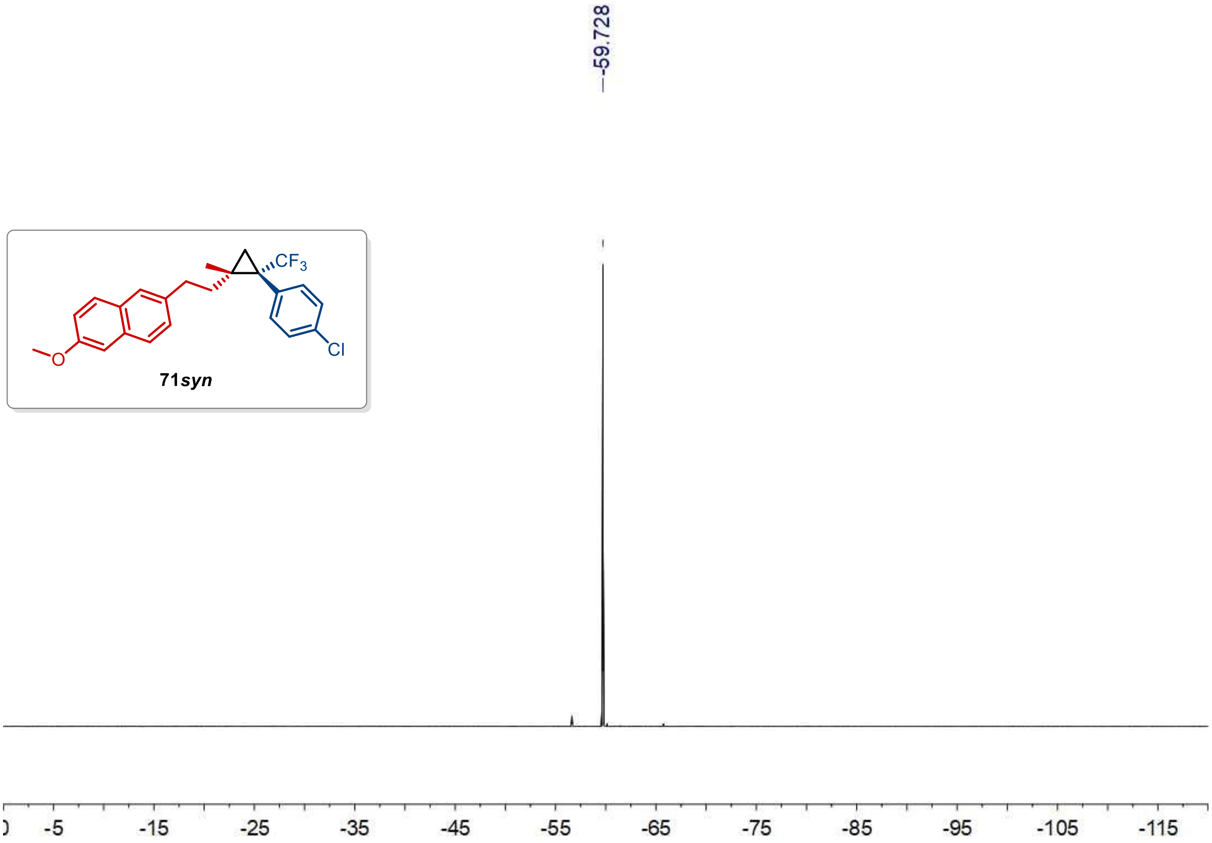

Supplementary Fig. 209 ^{19}**F NMR** (470 MHz, $CDCl_3$) spectrum of compound **71-*syn***.

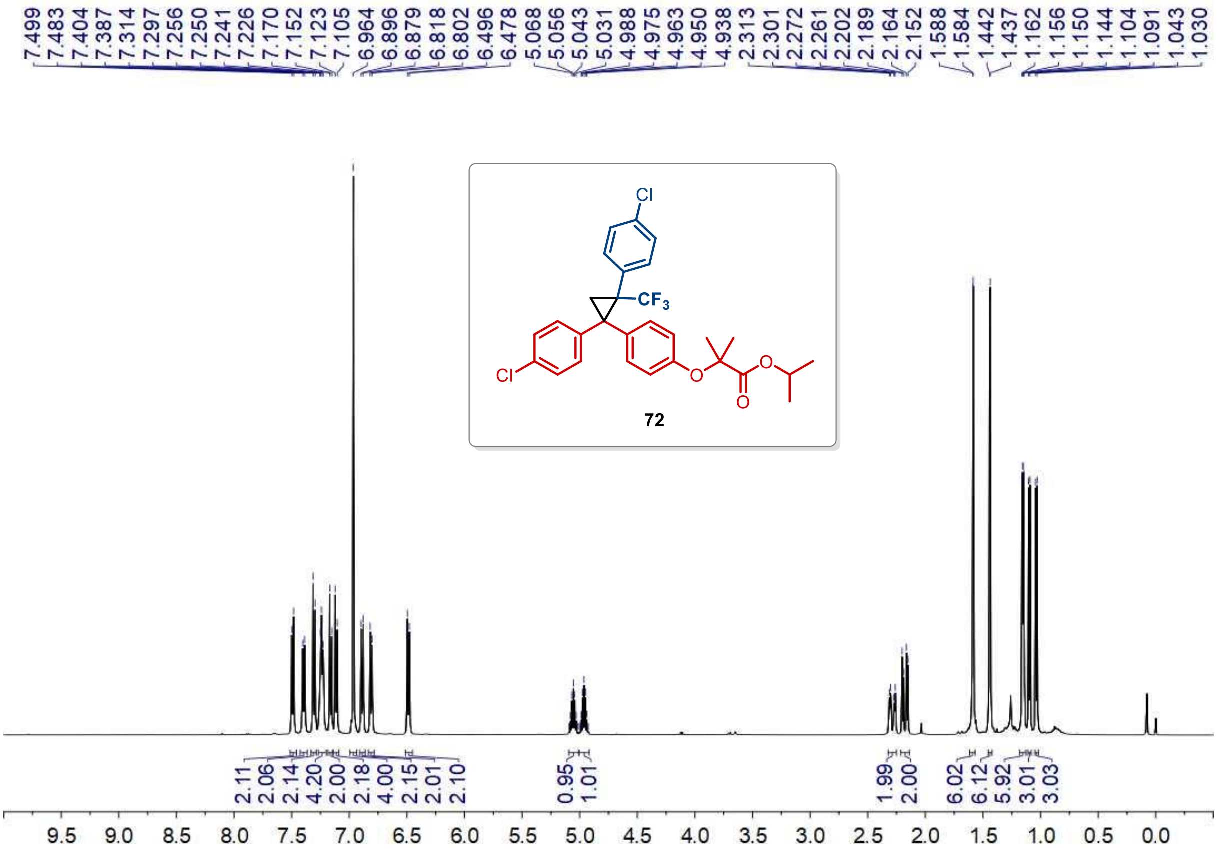

Supplementary Fig. 210 ^{1}**H NMR** (500 MHz, $CDCl_3$) spectrum of compound **72**.

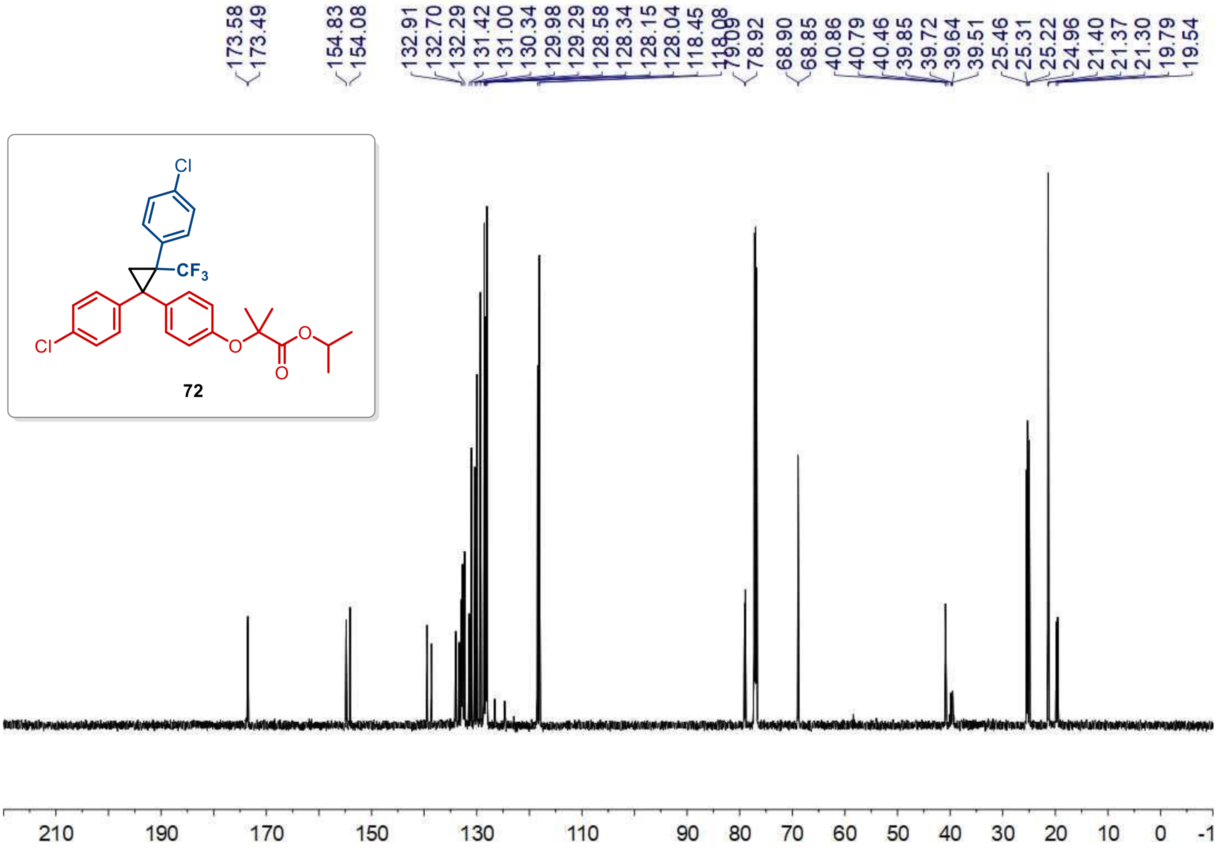

Supplementary Fig. 211 **^{13}C NMR** (150 MHz, $CDCl_3$) spectrum of compound **72**.

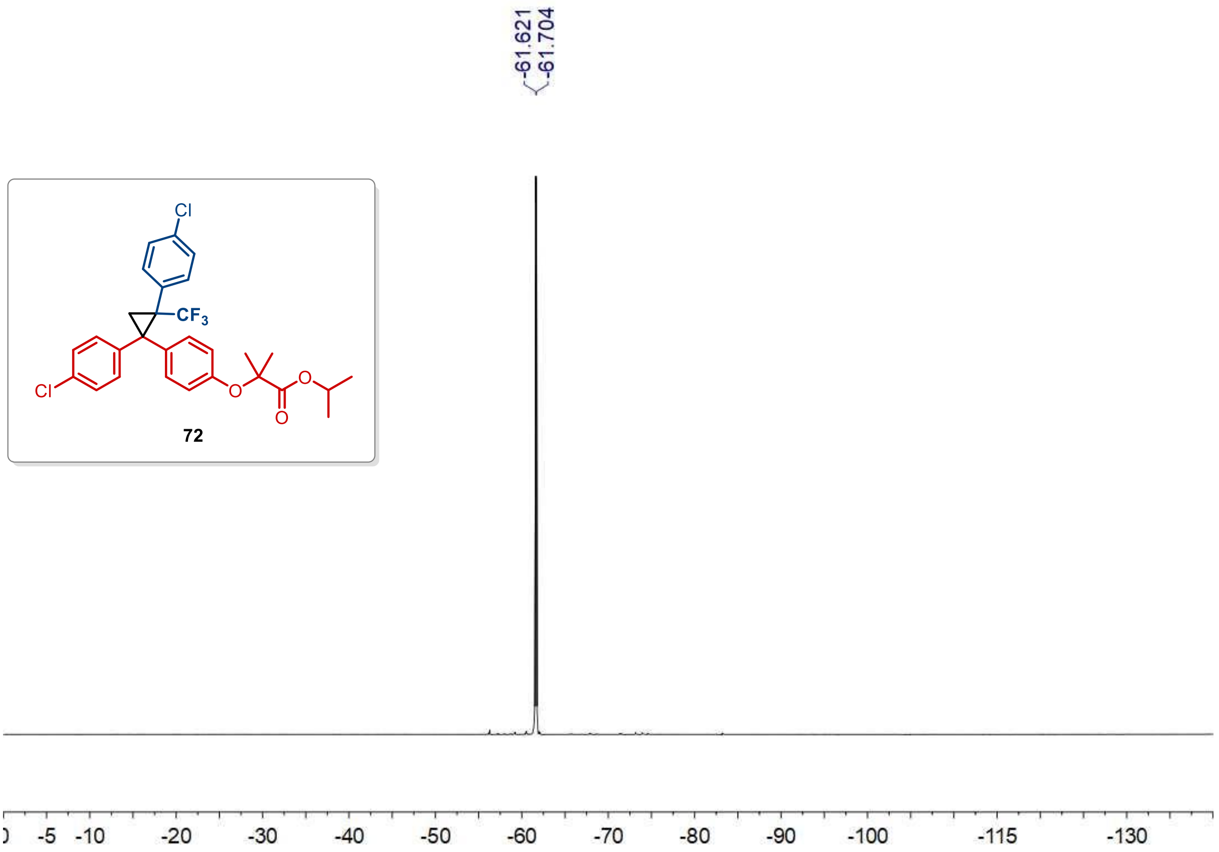

Supplementary Fig. 212 **^{19}F NMR** (564 MHz, $CDCl_3$) spectrum of compound **72**.

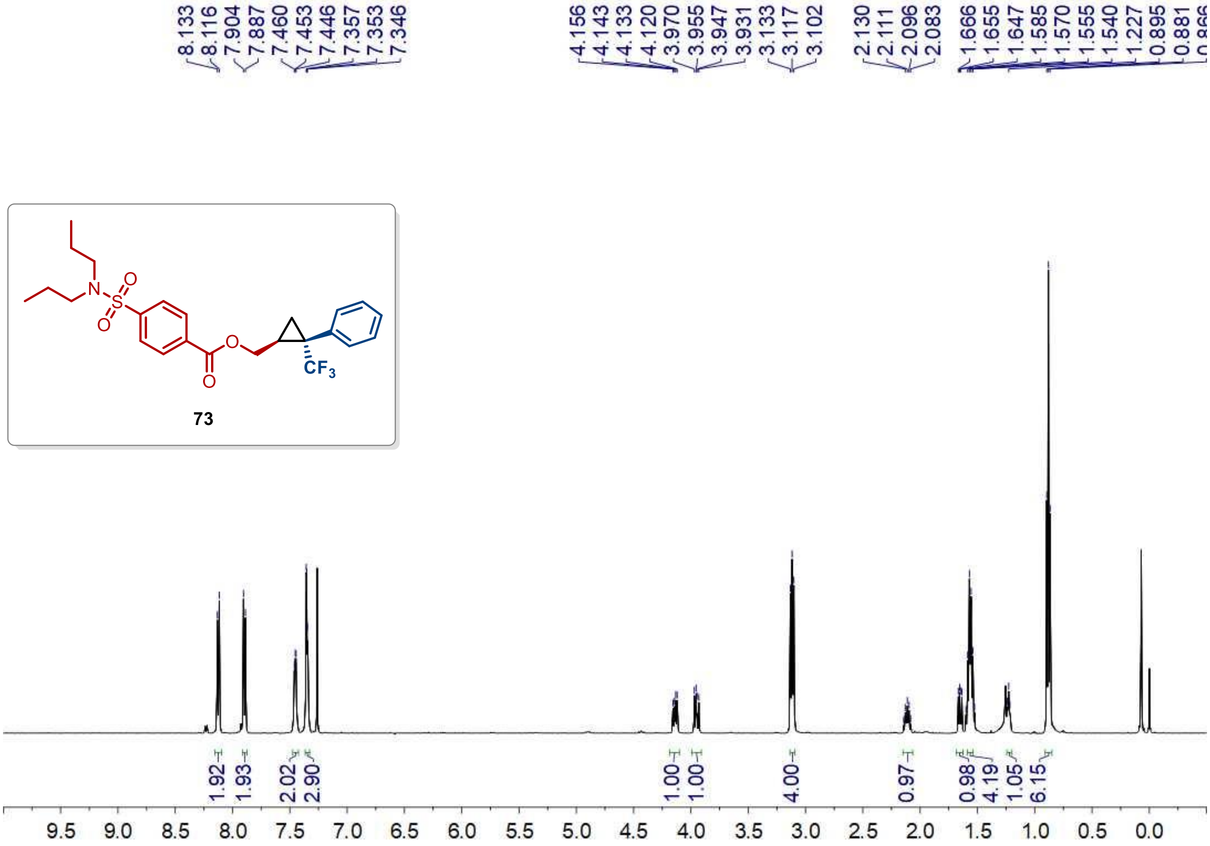

Supplementary Fig. 213 ^{1}H NMR (500 MHz, $CDCl_3$) spectrum of compound **73**.

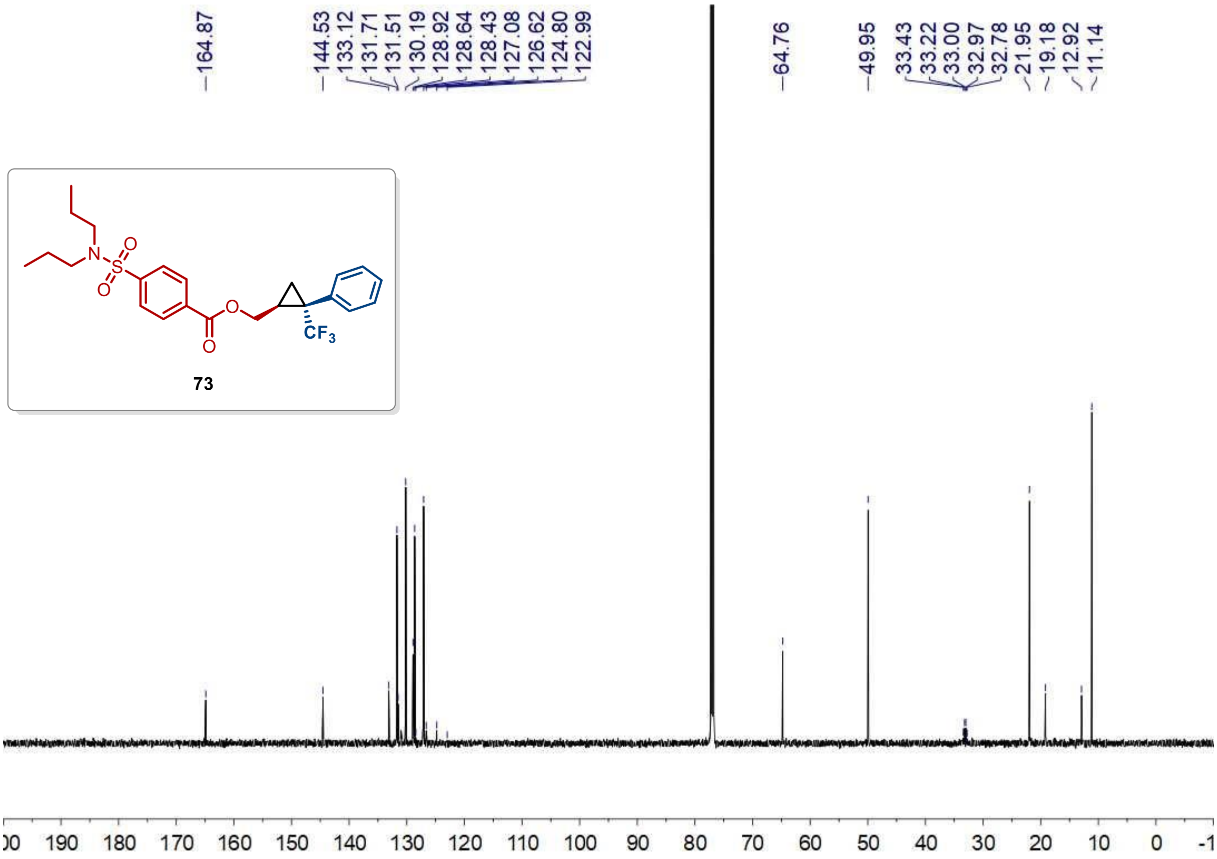

Supplementary Fig. 214 ^{13}C NMR (150 MHz, $CDCl_3$) spectrum of compound **73**.

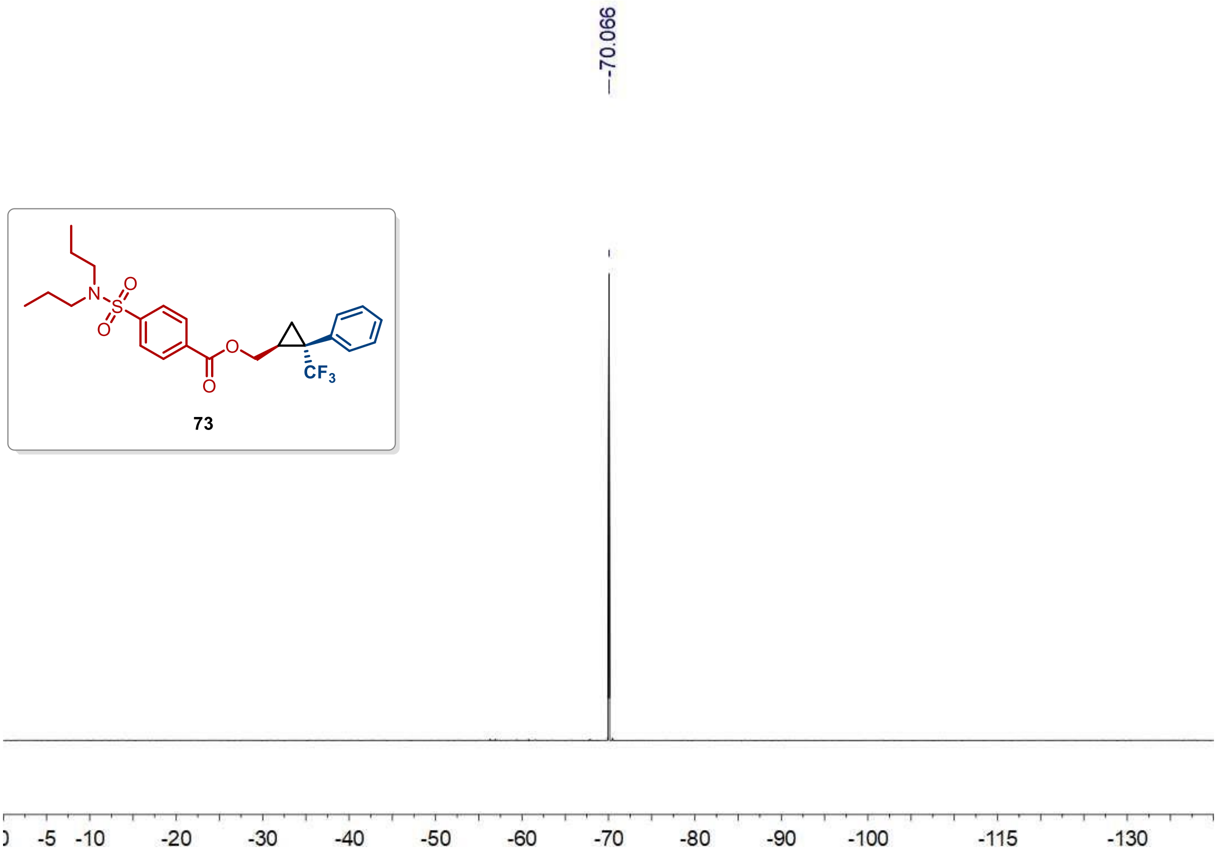

Supplementary Fig. 215 ^{19}**F NMR** (470 MHz, $CDCl_3$) spectrum of compound **73**.

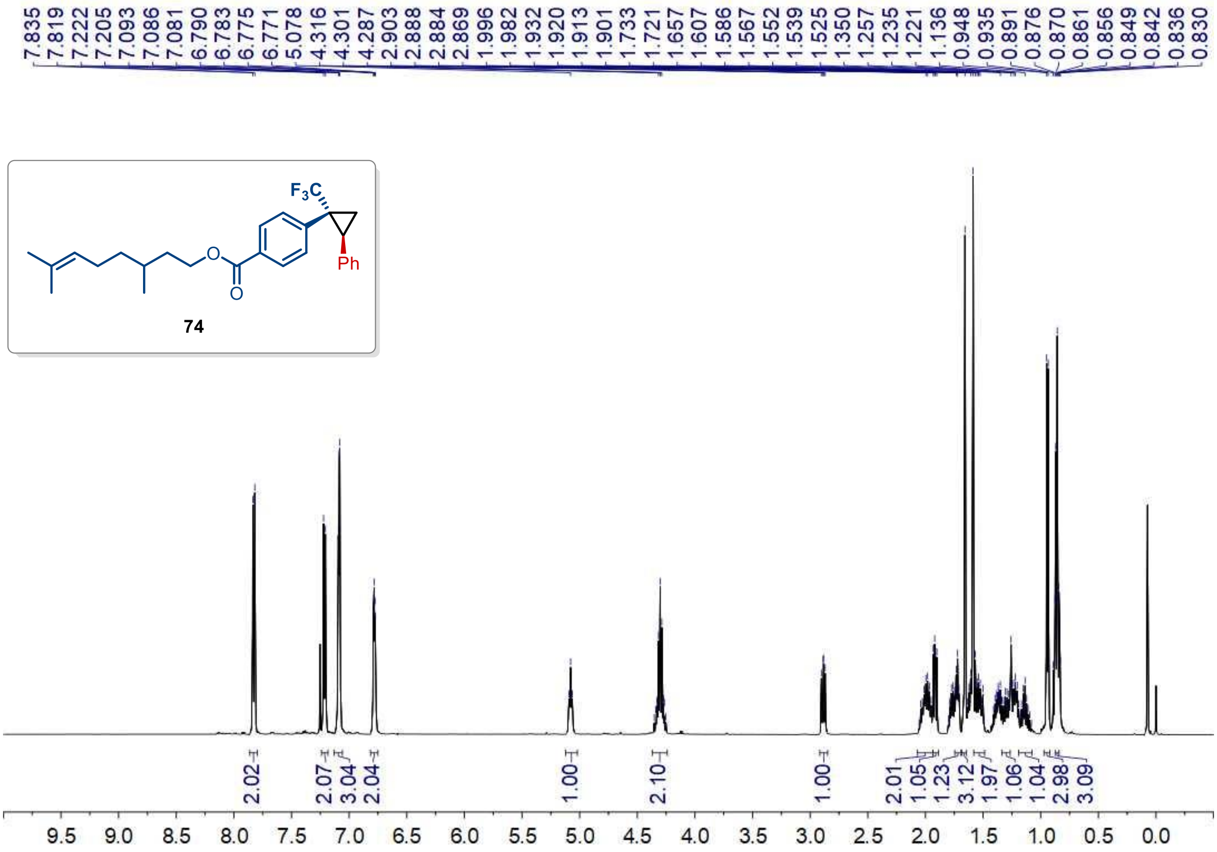

Supplementary Fig. 216 ^{1}**H NMR** (500 MHz, $CDCl_3$) spectrum of compound **74**.

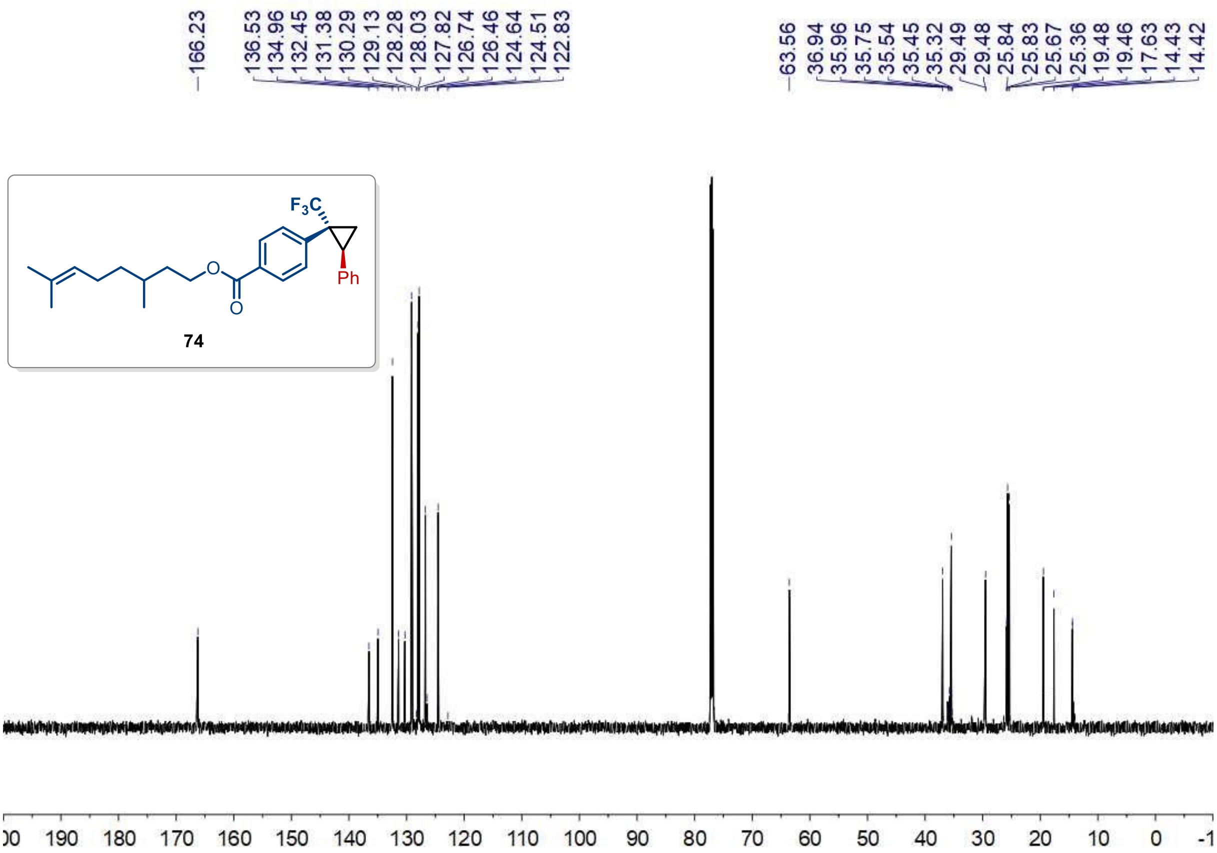

Supplementary Fig. 217 ^{13}**C NMR** (150 MHz, $CDCl_3$) spectrum of compound **74**.

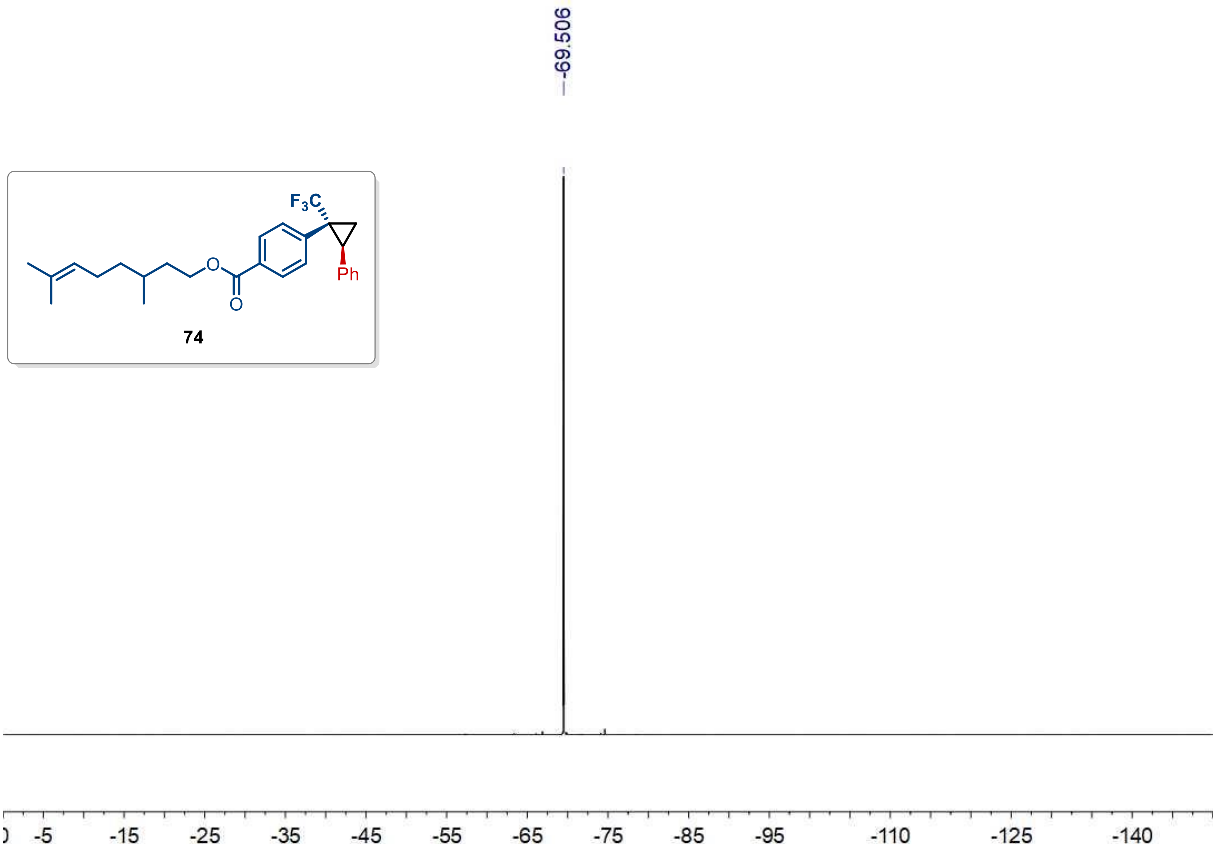

Supplementary Fig. 218 ^{19}**F NMR** (564 MHz, $CDCl_3$) spectrum of compound **74**.

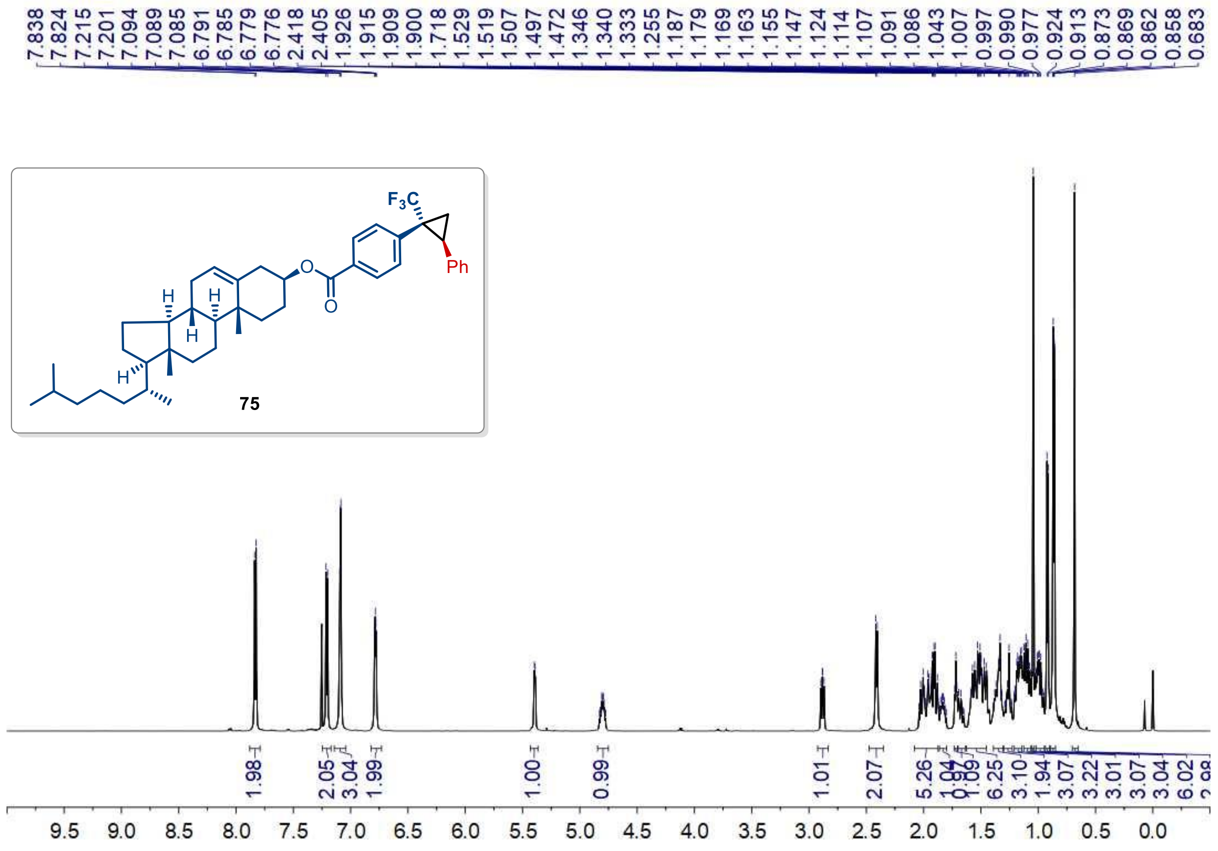

Supplementary Fig. 219 ^{1}H NMR (600 MHz, $CDCl_3$) spectrum of compound **75**.

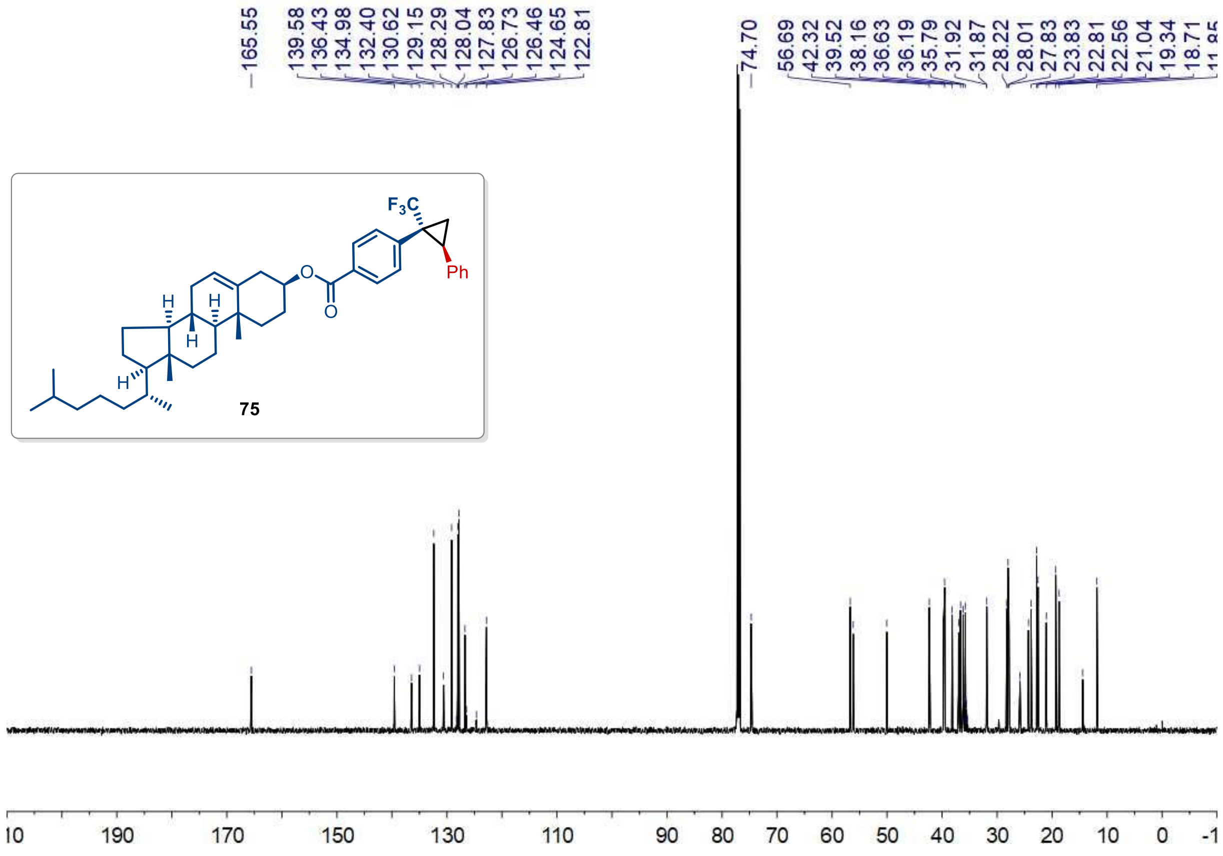

Supplementary Fig. 220 ^{13}C NMR (150 MHz, $CDCl_3$) spectrum of compound **75**.

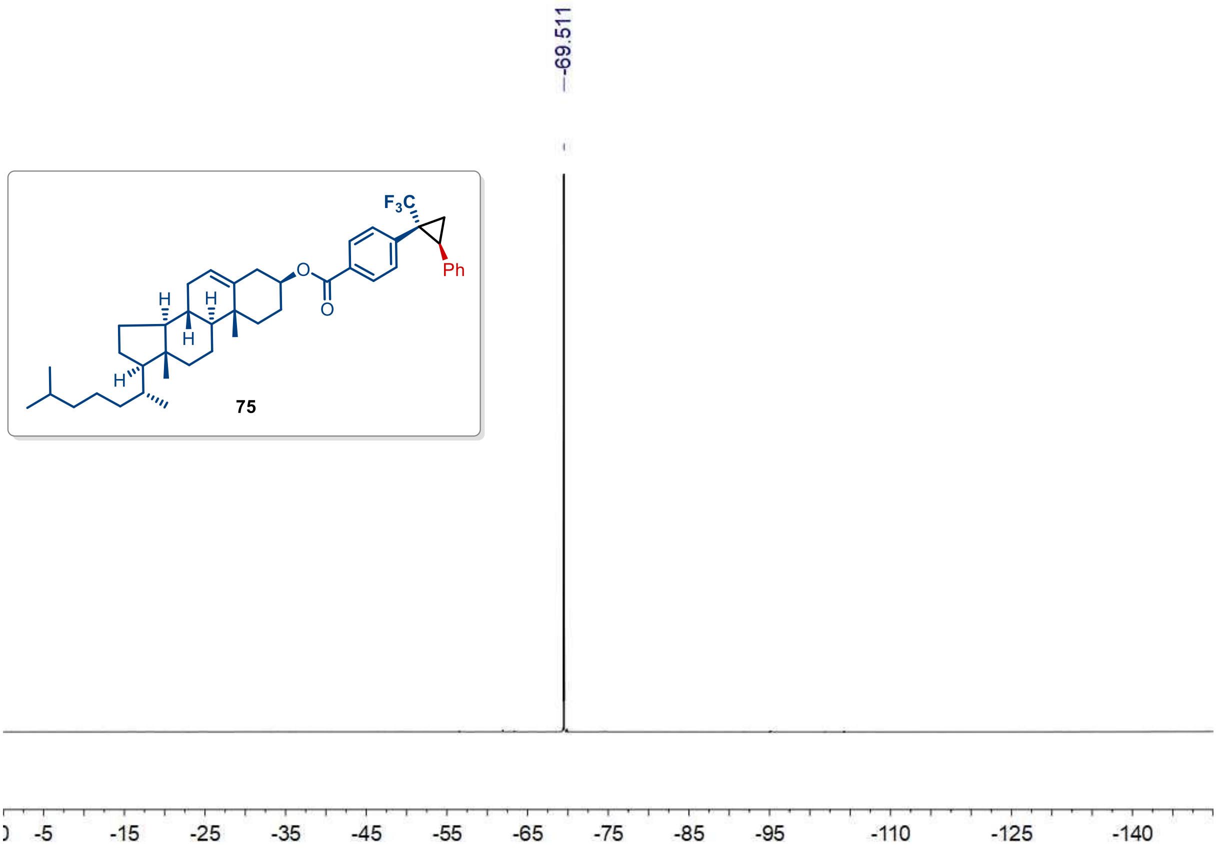

Supplementary Fig. 221 ^{19}**F NMR** (564 MHz, $CDCl_3$) spectrum of compound **75**.

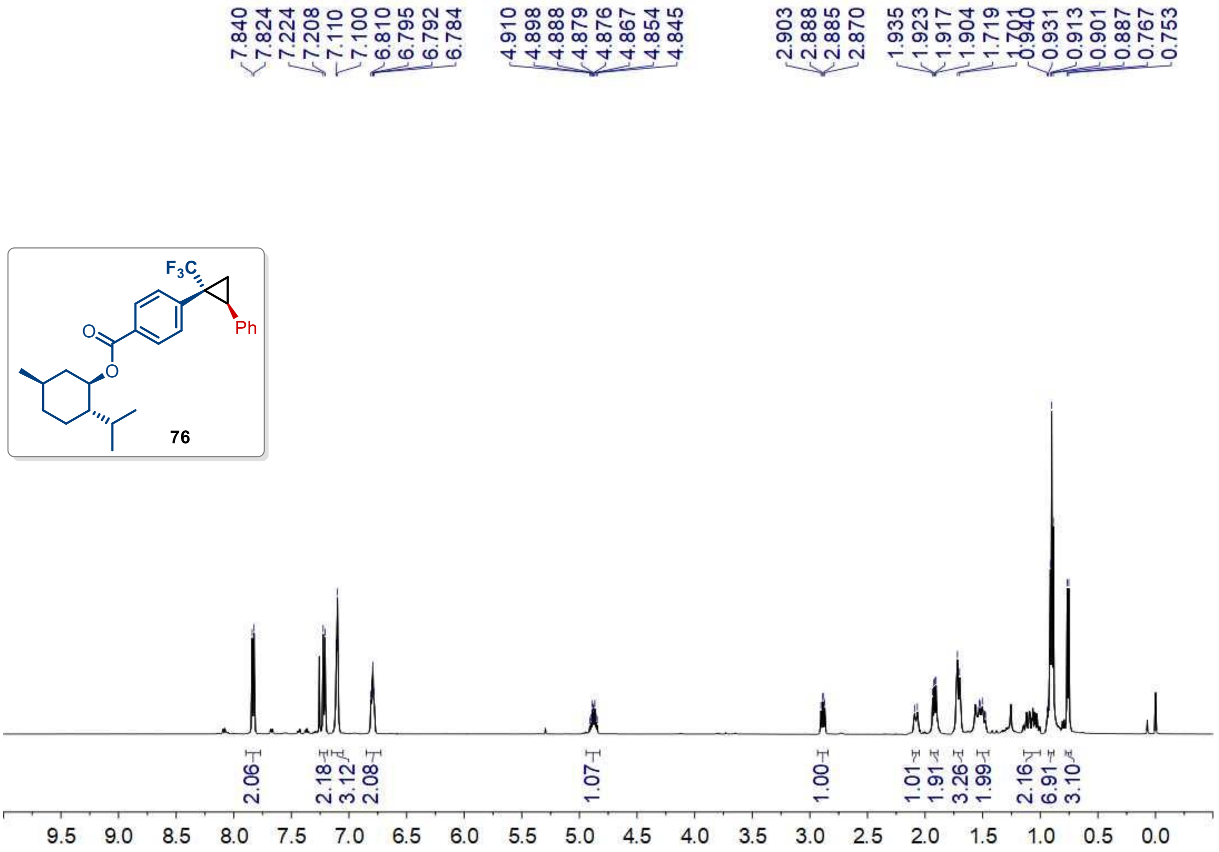

Supplementary Fig. 222 ^{1}**H NMR** (500 MHz, $CDCl_3$) spectrum of compound **76**.

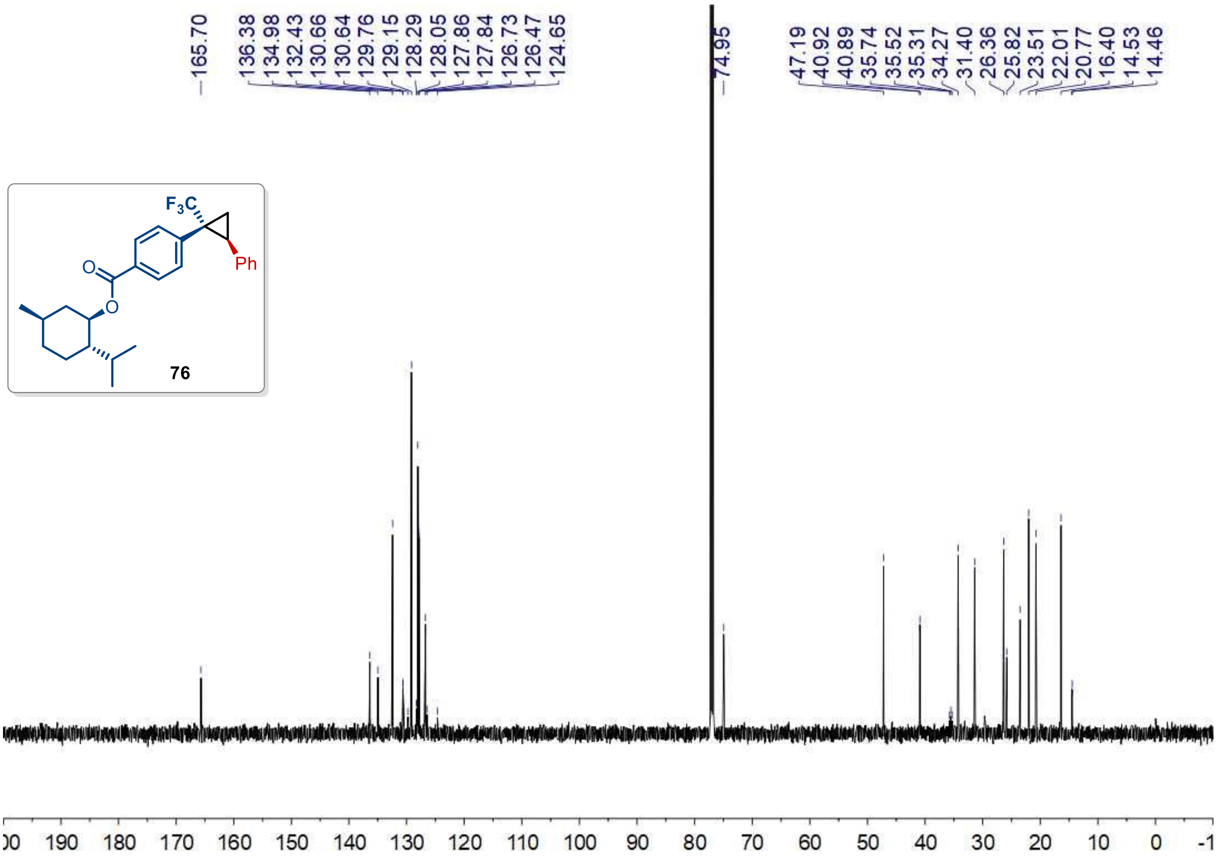

Supplementary Fig. 223 ^{13}**C NMR** (150 MHz, $CDCl_3$) spectrum of compound **76**.

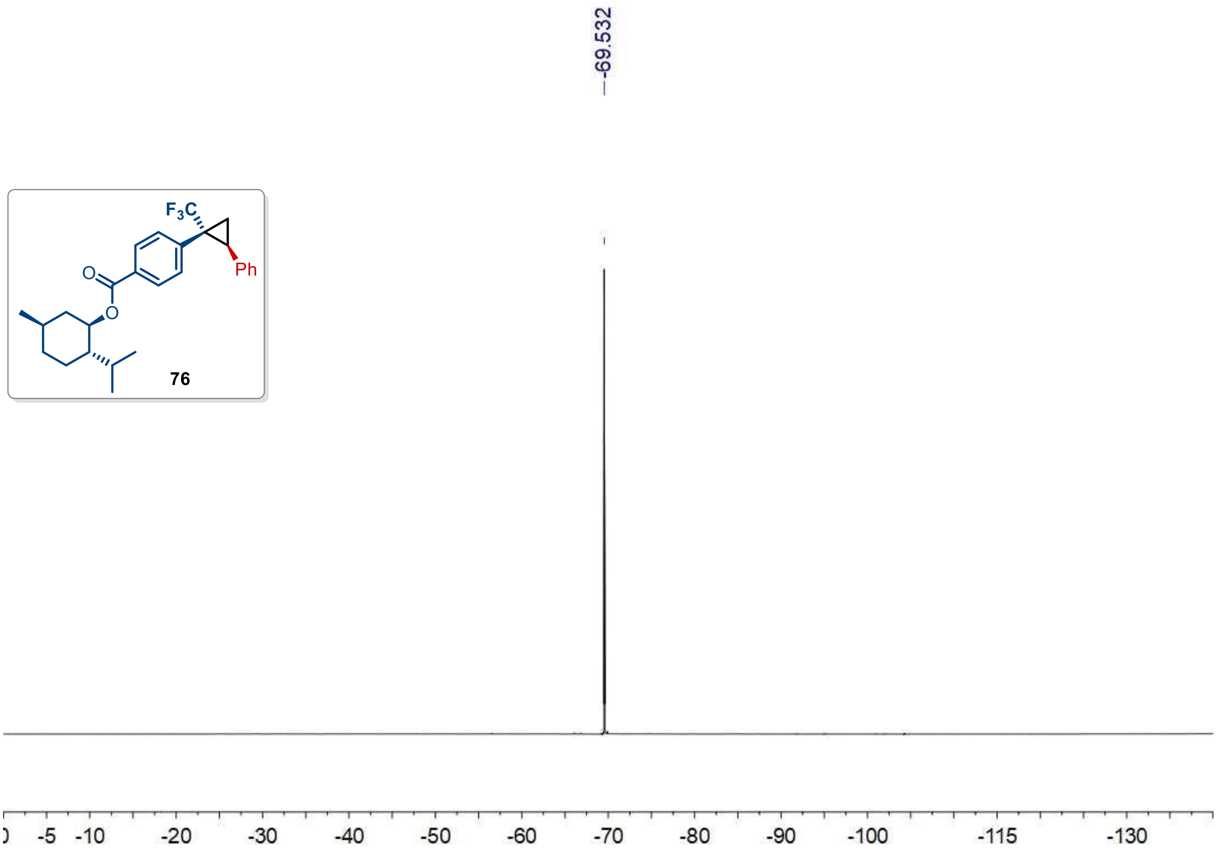

Supplementary Fig. 224 ^{19}**F NMR** (564 MHz, $CDCl_3$) spectrum of compound **76**.

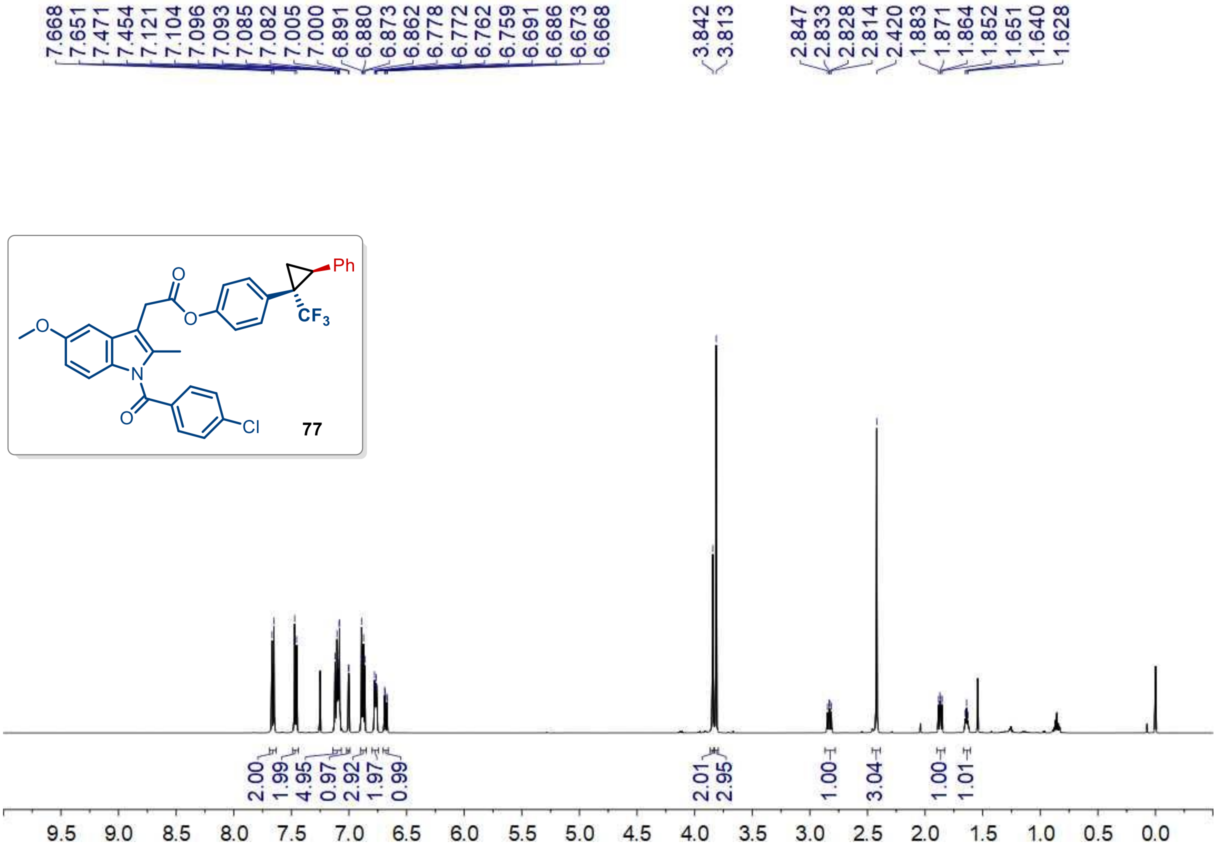

Supplementary Fig. 225 ^{1}**H NMR** (500 MHz, $CDCl_3$) spectrum of compound **77**.

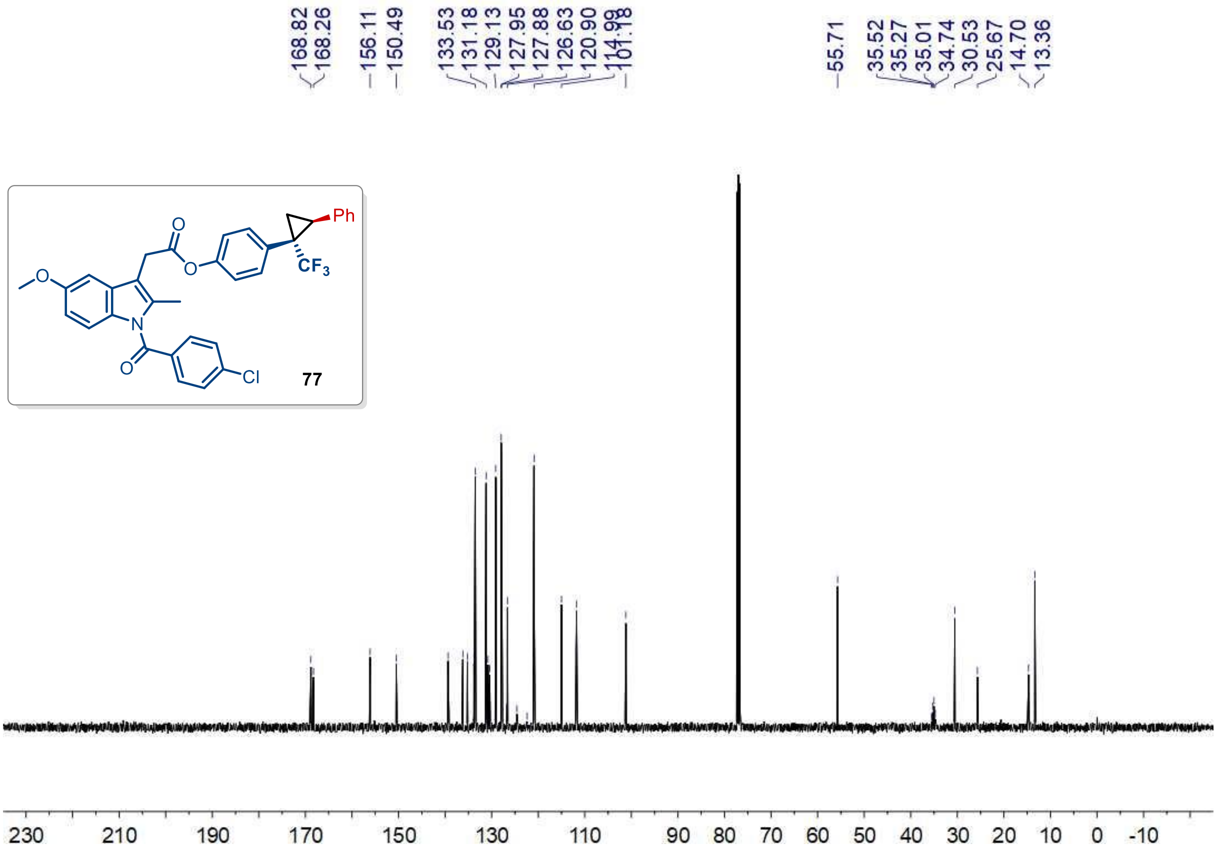

Supplementary Fig. 226 ^{13}**C NMR** (125 MHz, $CDCl_3$) spectrum of compound **77**.

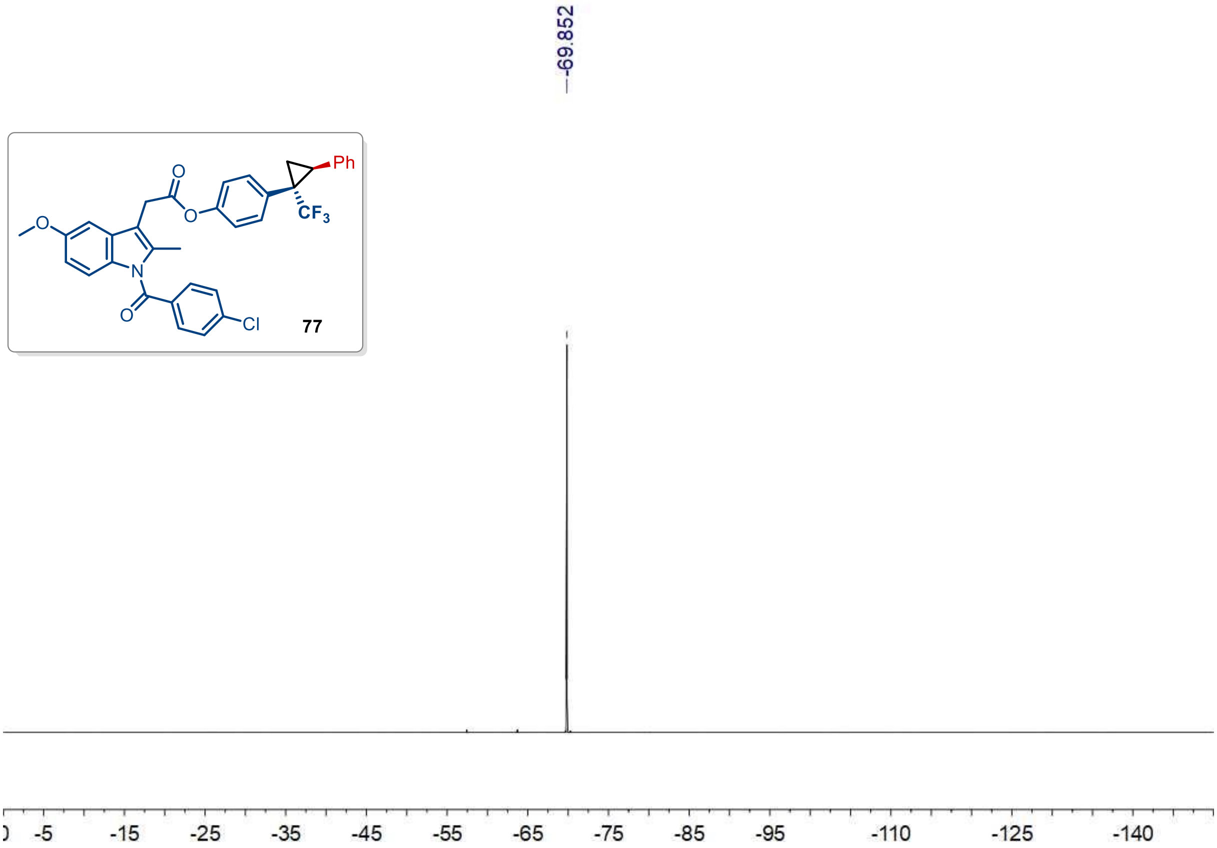

Supplementary Fig. 227 ^{19}**F NMR** (470 MHz, $CDCl_3$) spectrum of compound **77**.

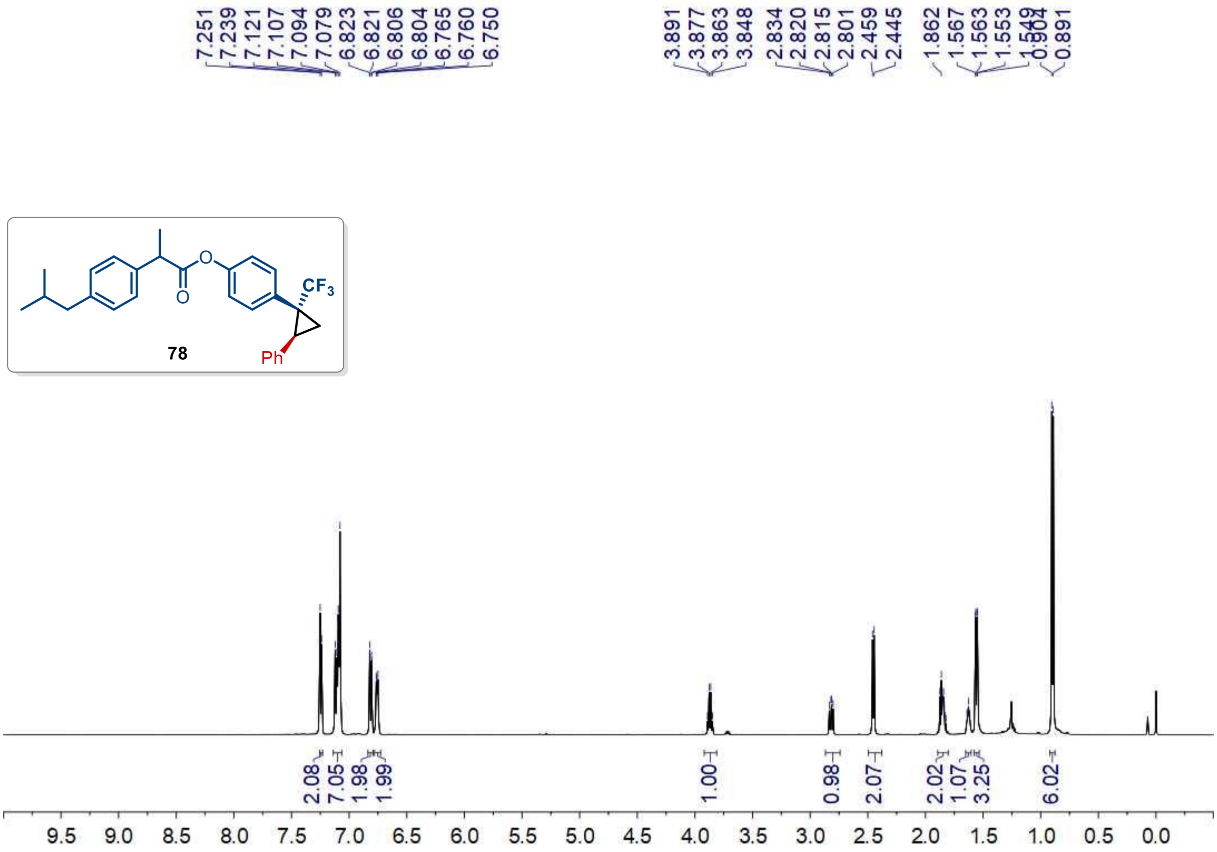

Supplementary Fig. 228 ^{1}**H NMR** (500 MHz, $CDCl_3$) spectrum of compound **78**.

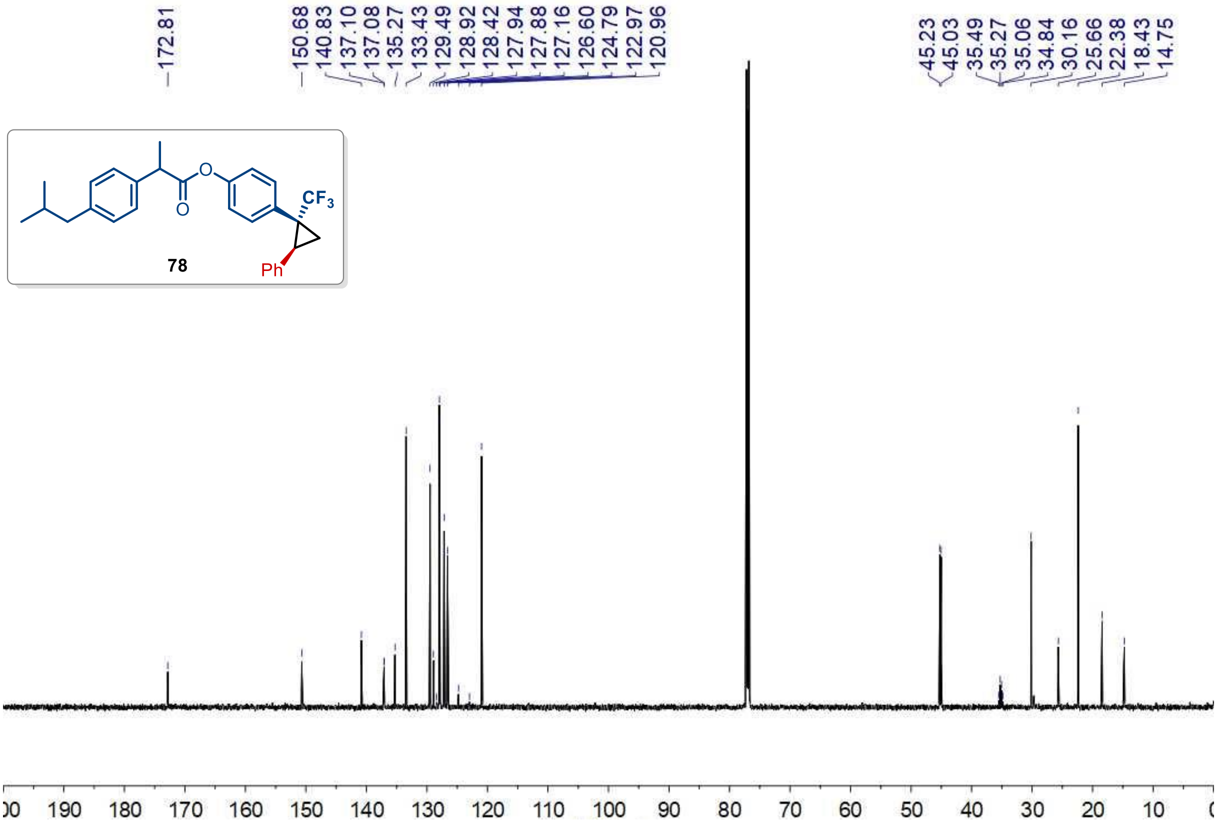

Supplementary Fig. 229 ^{13}**C NMR** (150 MHz, $CDCl_3$) spectrum of compound **78**.

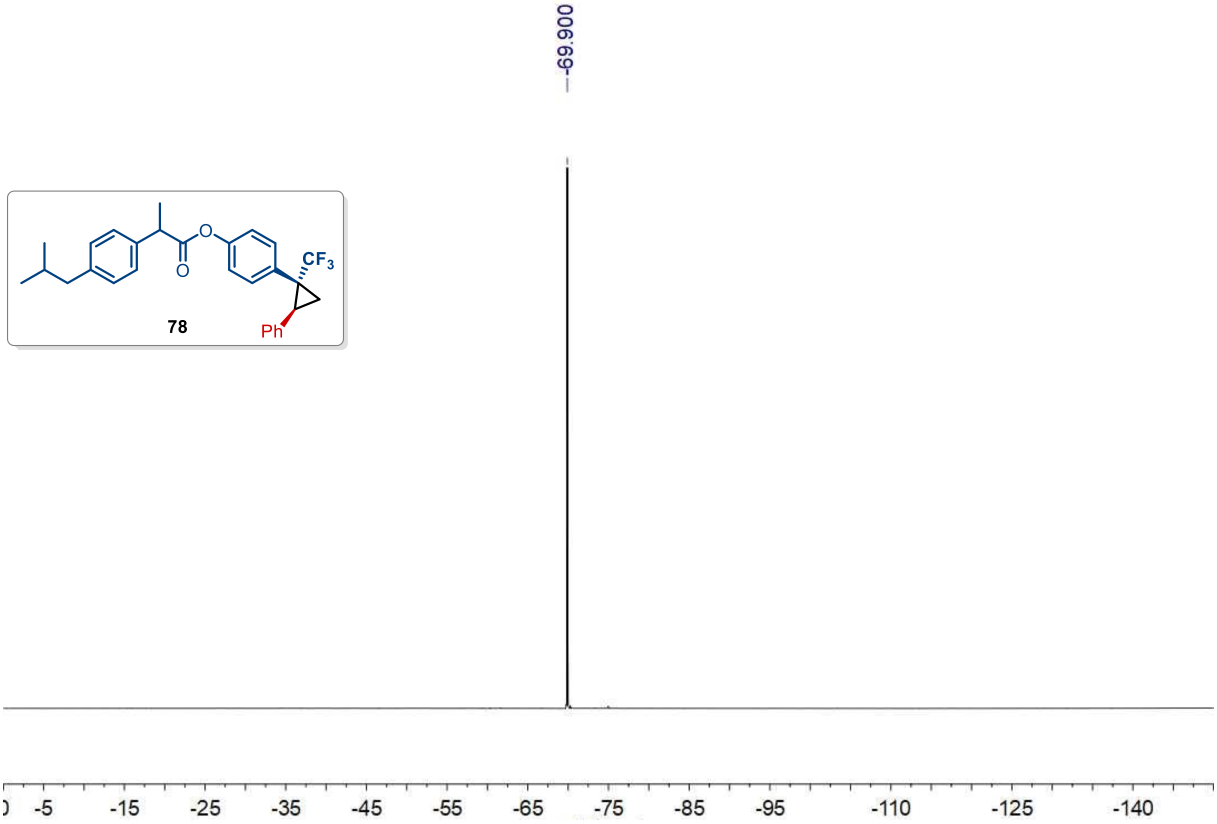

Supplementary Fig. 230 ^{19}**F NMR** (564 MHz, $CDCl_3$) spectrum of compound **78**.

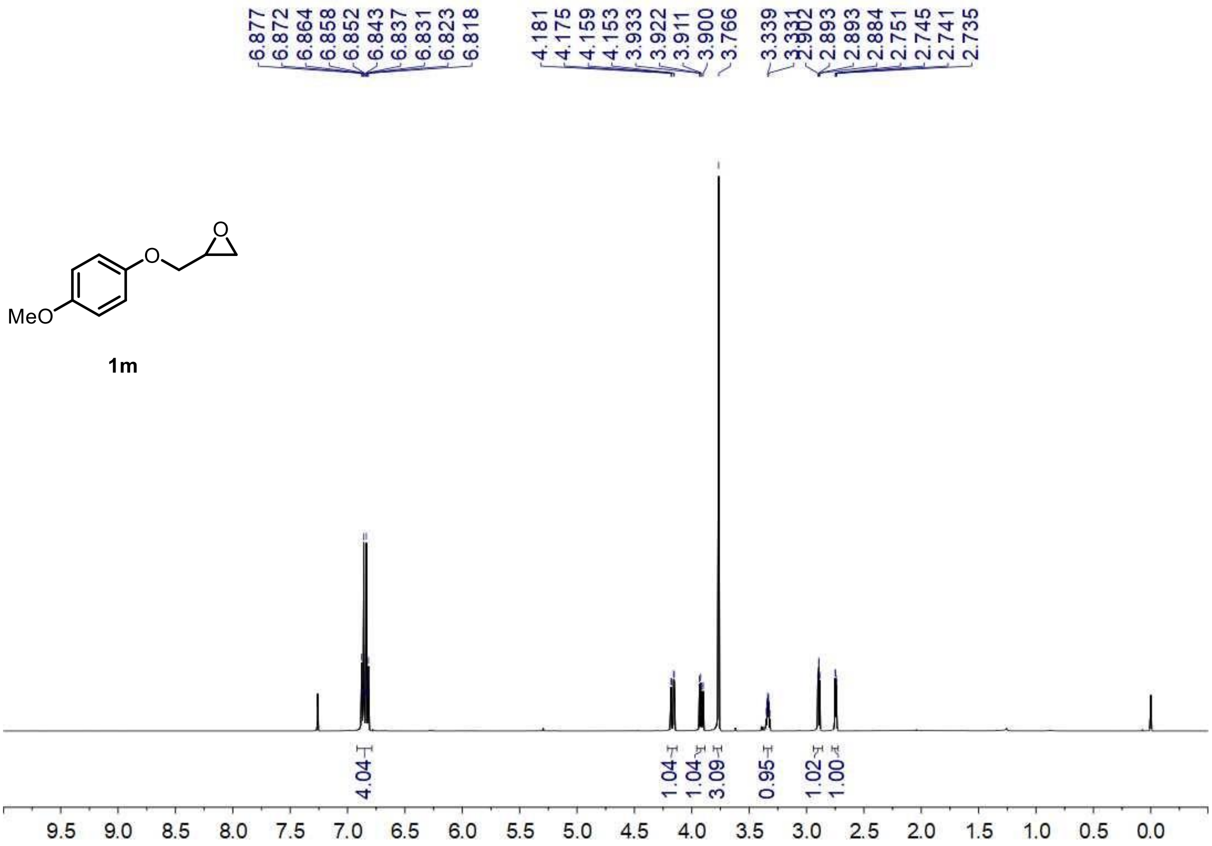

Supplementary Fig. 231 ^{1}**H NMR** (500 MHz, $CDCl_3$) spectrum of compound **1m**.

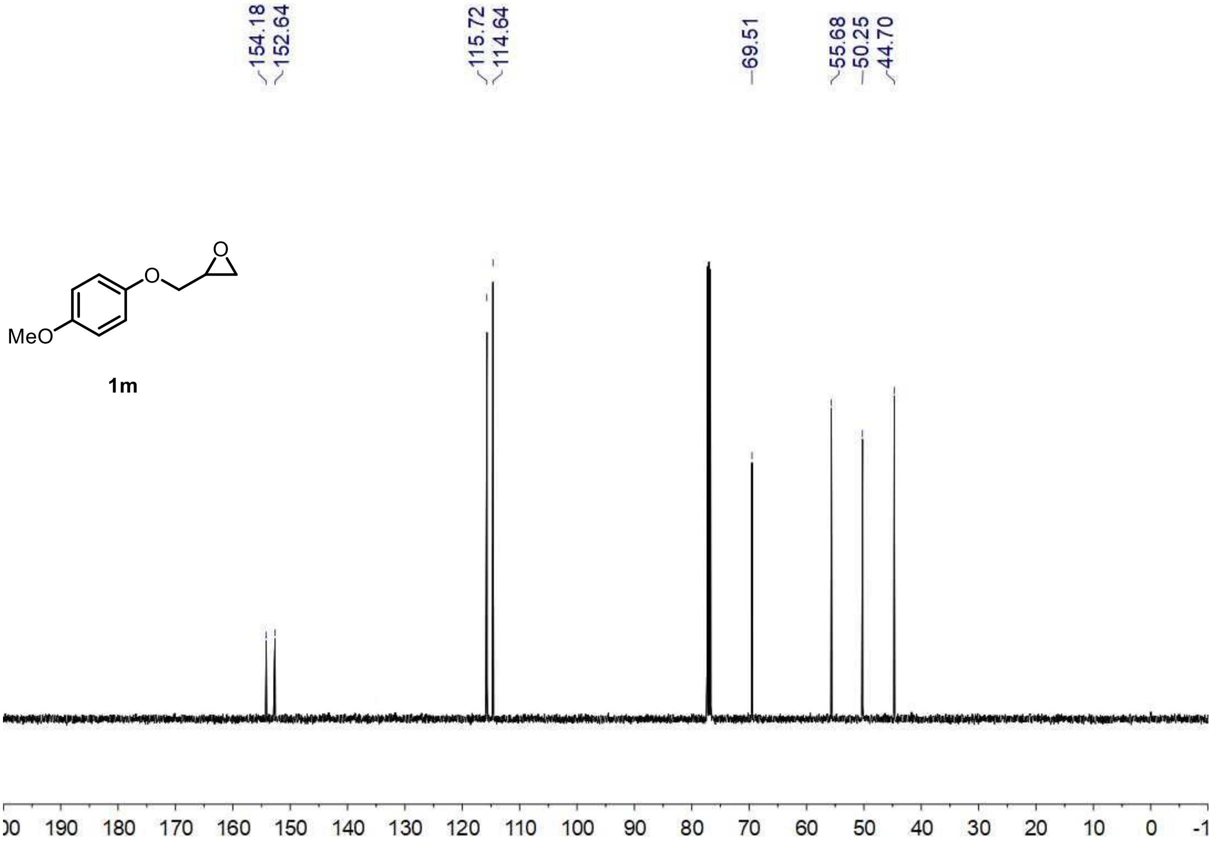

Supplementary Fig. 232 ^{13}**C NMR** (125 MHz, $CDCl_3$) spectrum of compound **1m**.

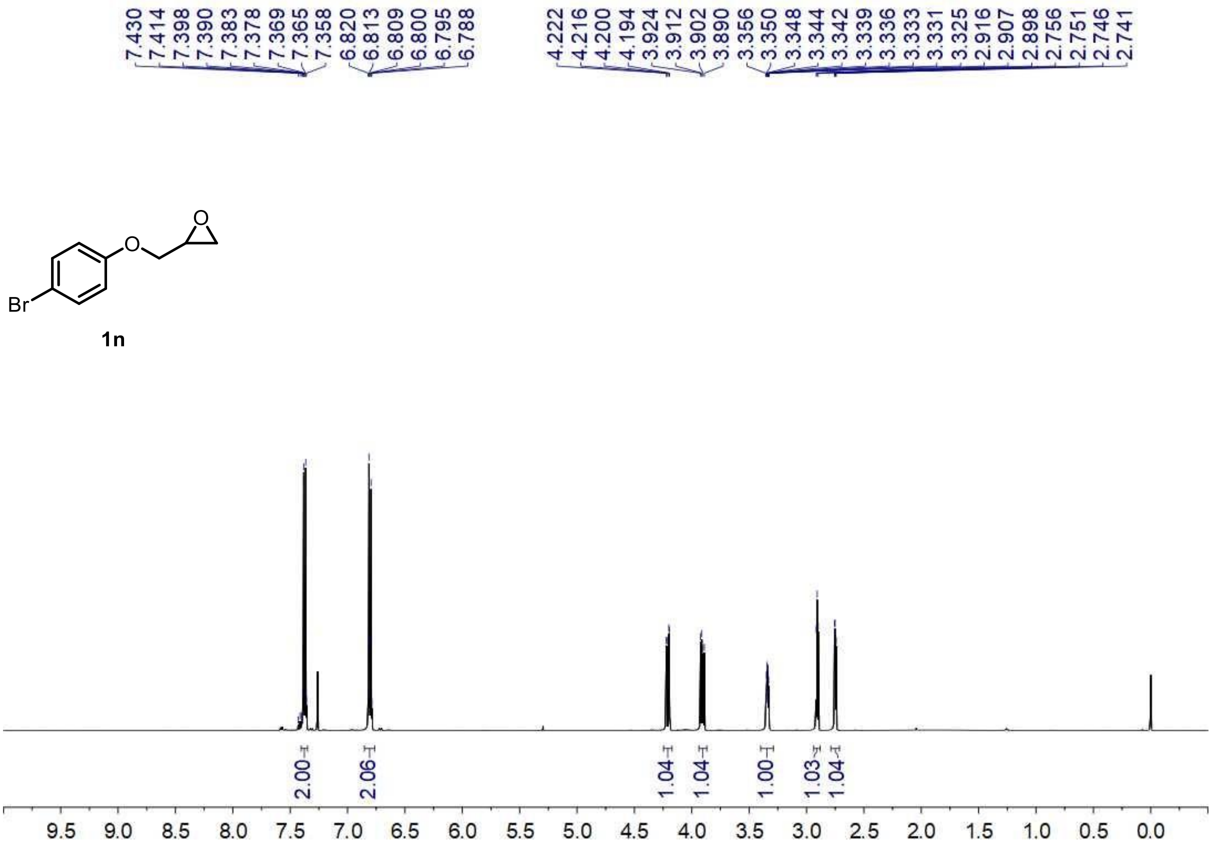

Supplementary Fig. 233 ^{1}H NMR (500 MHz, $CDCl_3$) spectrum of compound **1n**.

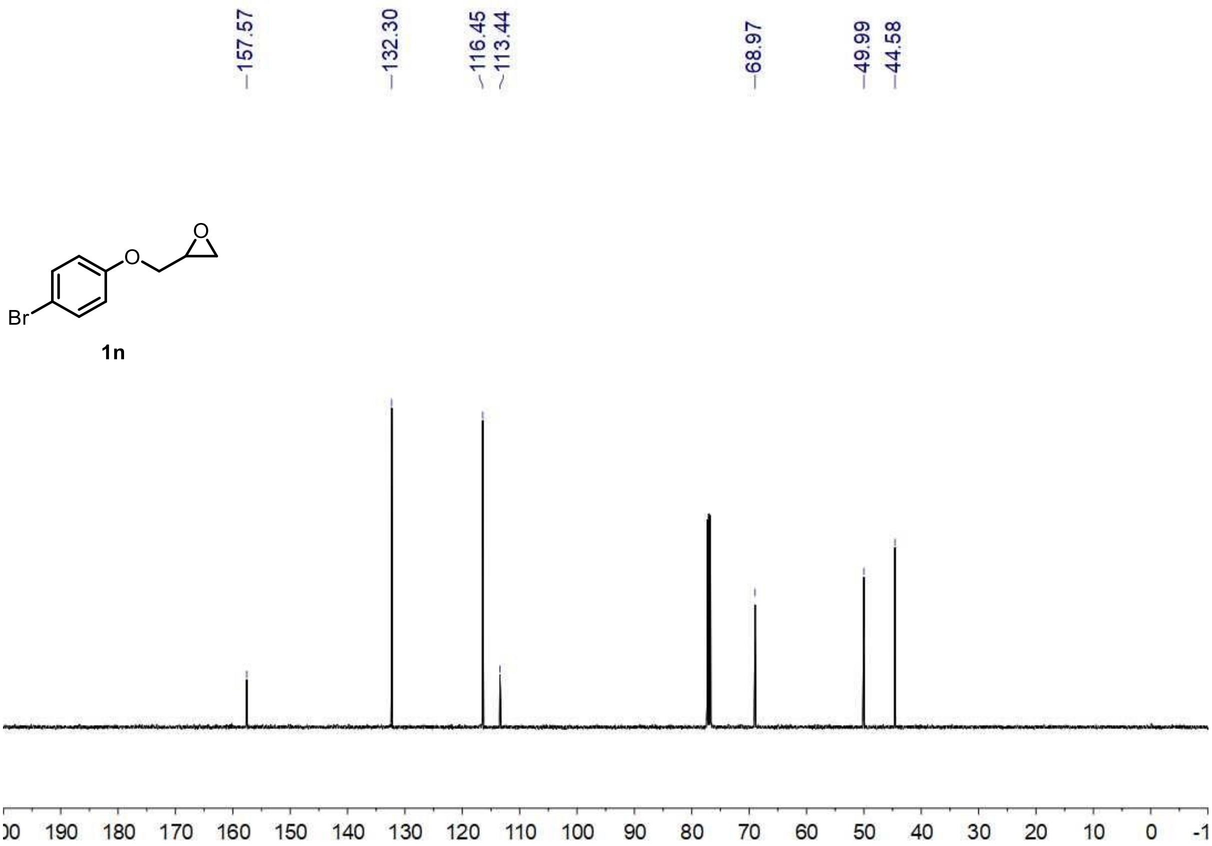

Supplementary Fig. 234 ^{13}C NMR (125 MHz, $CDCl_3$) spectrum of compound **1n**.

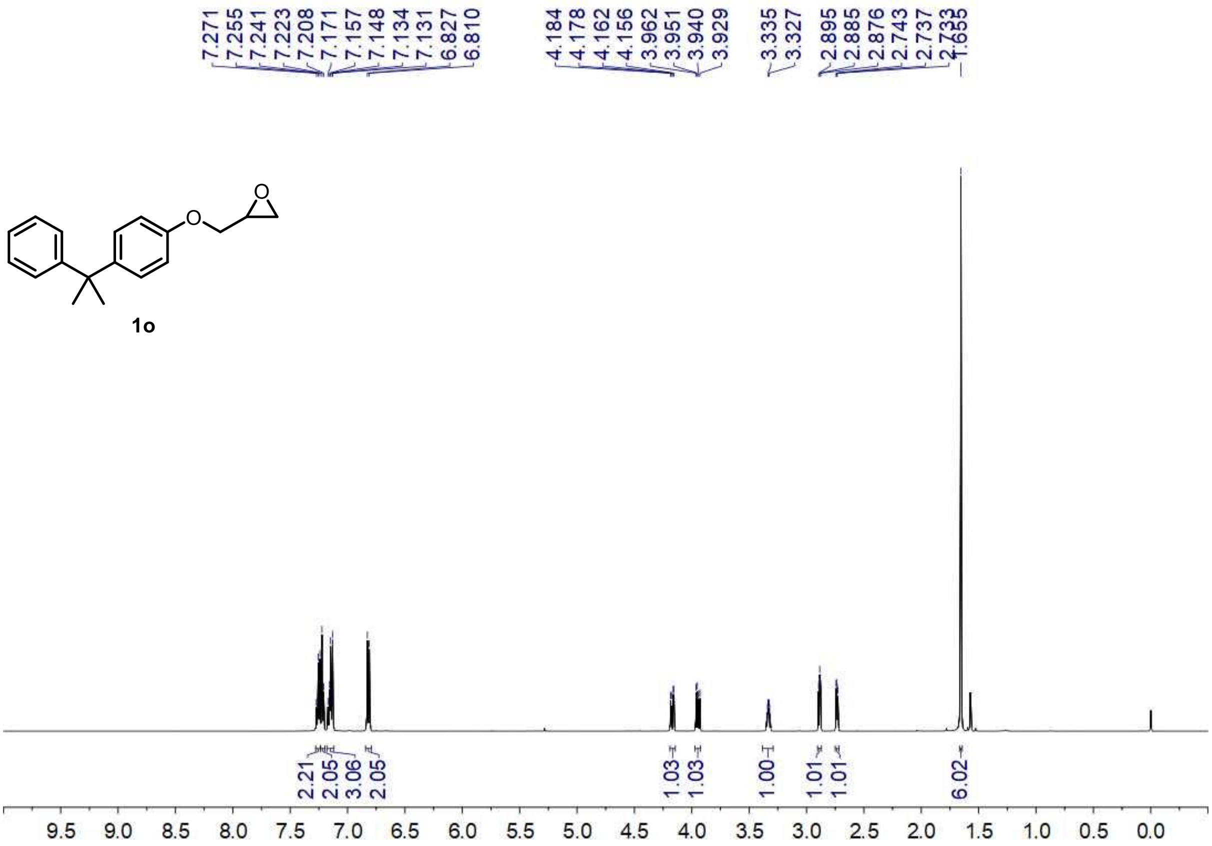

Supplementary Fig. 235 ^{1}H NMR (500 MHz, $CDCl_3$) spectrum of compound **1o**.

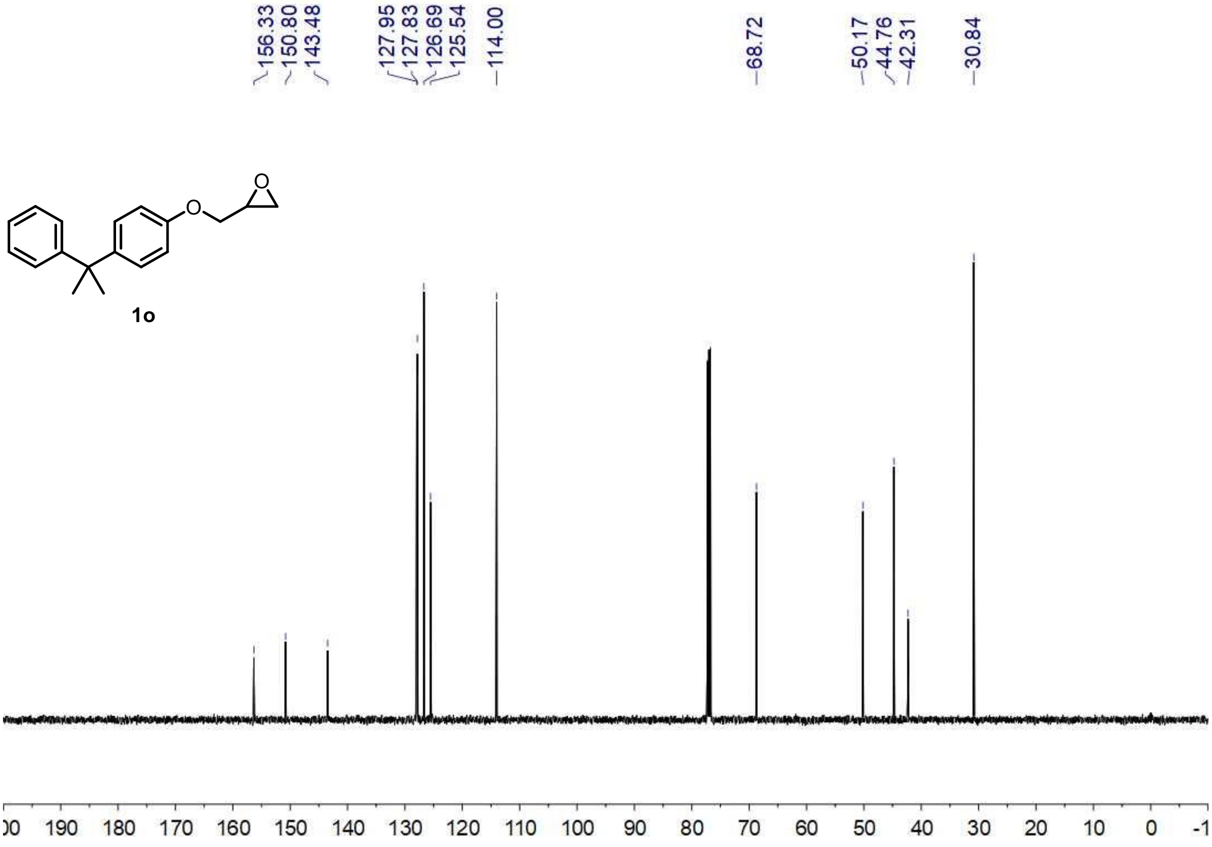

Supplementary Fig. 236 ^{13}C NMR (125 MHz, $CDCl_3$) spectrum of compound **1o**.

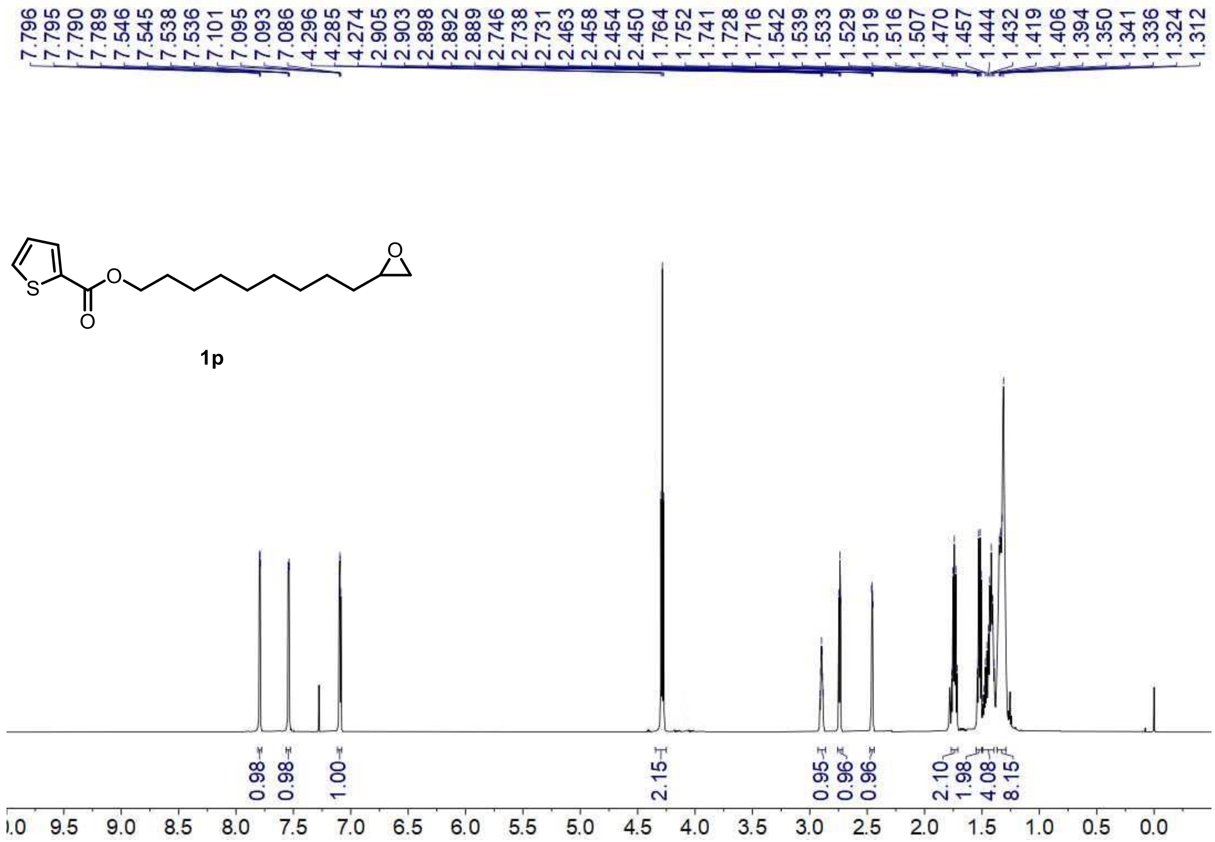

Supplementary Fig. 237 ^{1}**H NMR** (600 MHz, $CDCl_3$) spectrum of compound **1p**.

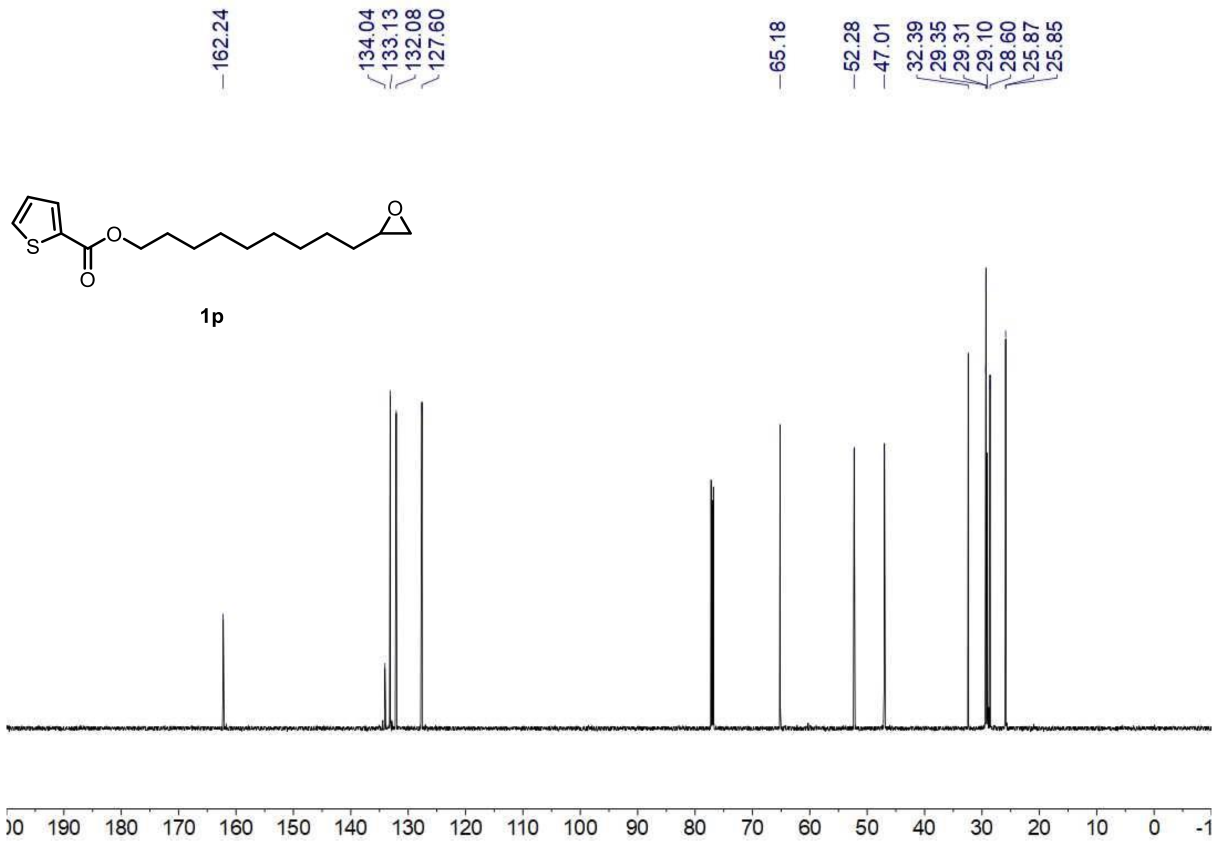

Supplementary Fig. 238 ^{13}**C NMR** (150 MHz, $CDCl_3$) spectrum of compound **1p**.

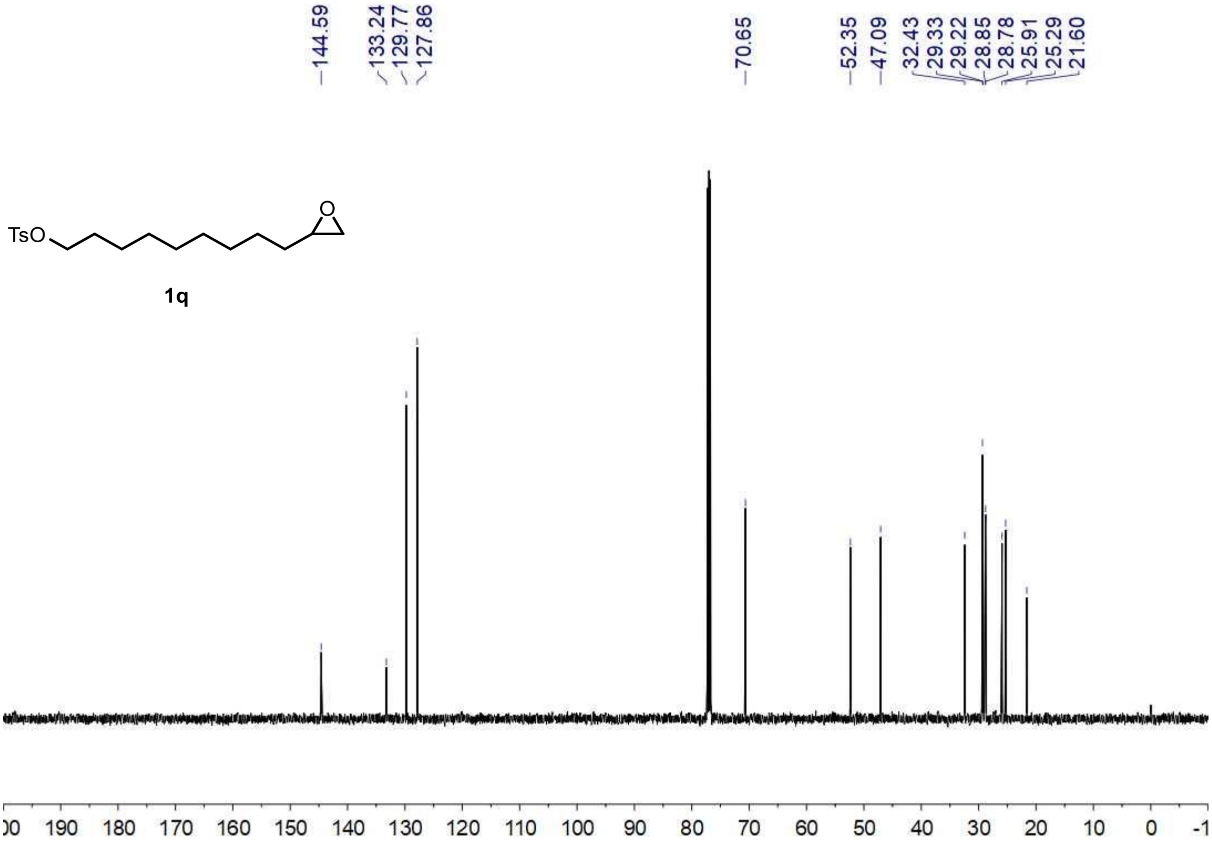

Supplementary Fig. 239 ^{1}H NMR (600 MHz, $CDCl_3$) spectrum of compound **1q**.

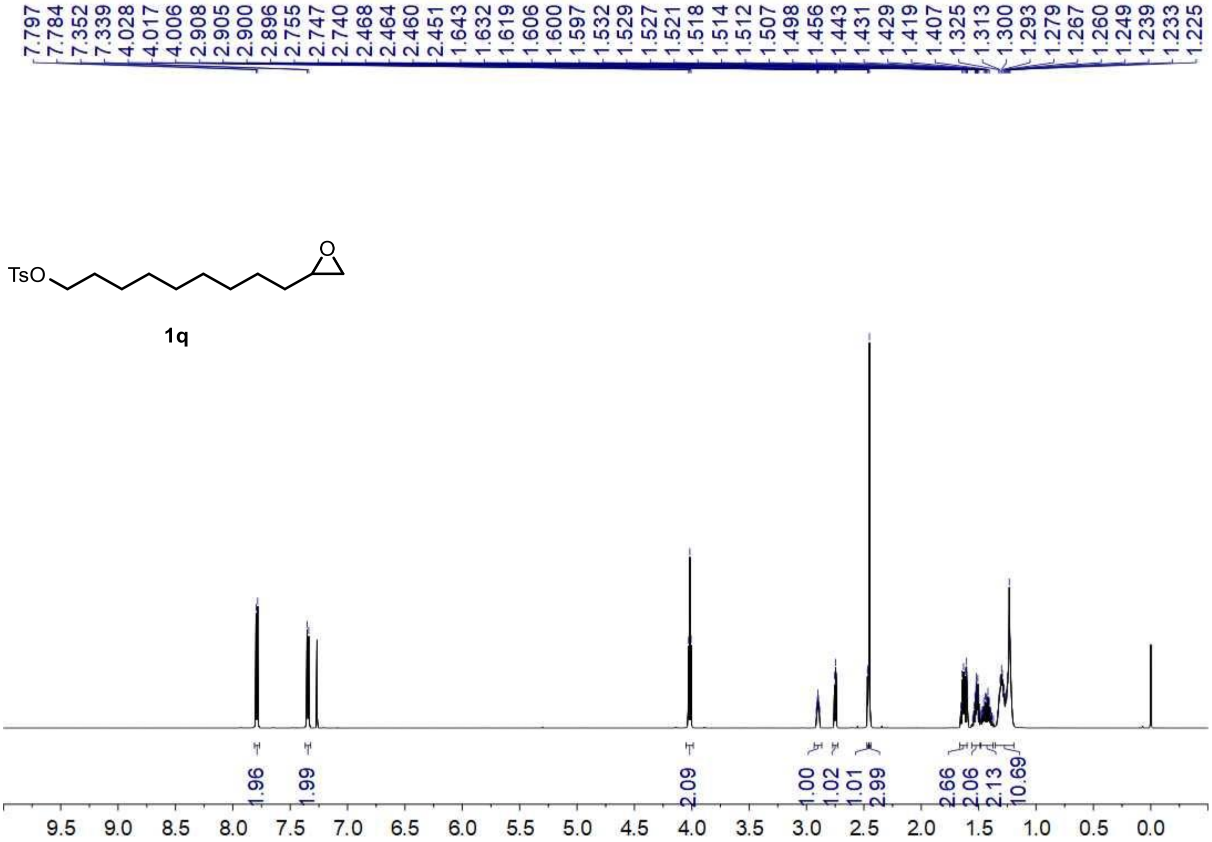

Supplementary Fig. 240 ^{13}C NMR (125 MHz, $CDCl_3$) spectrum of compound **1q**.

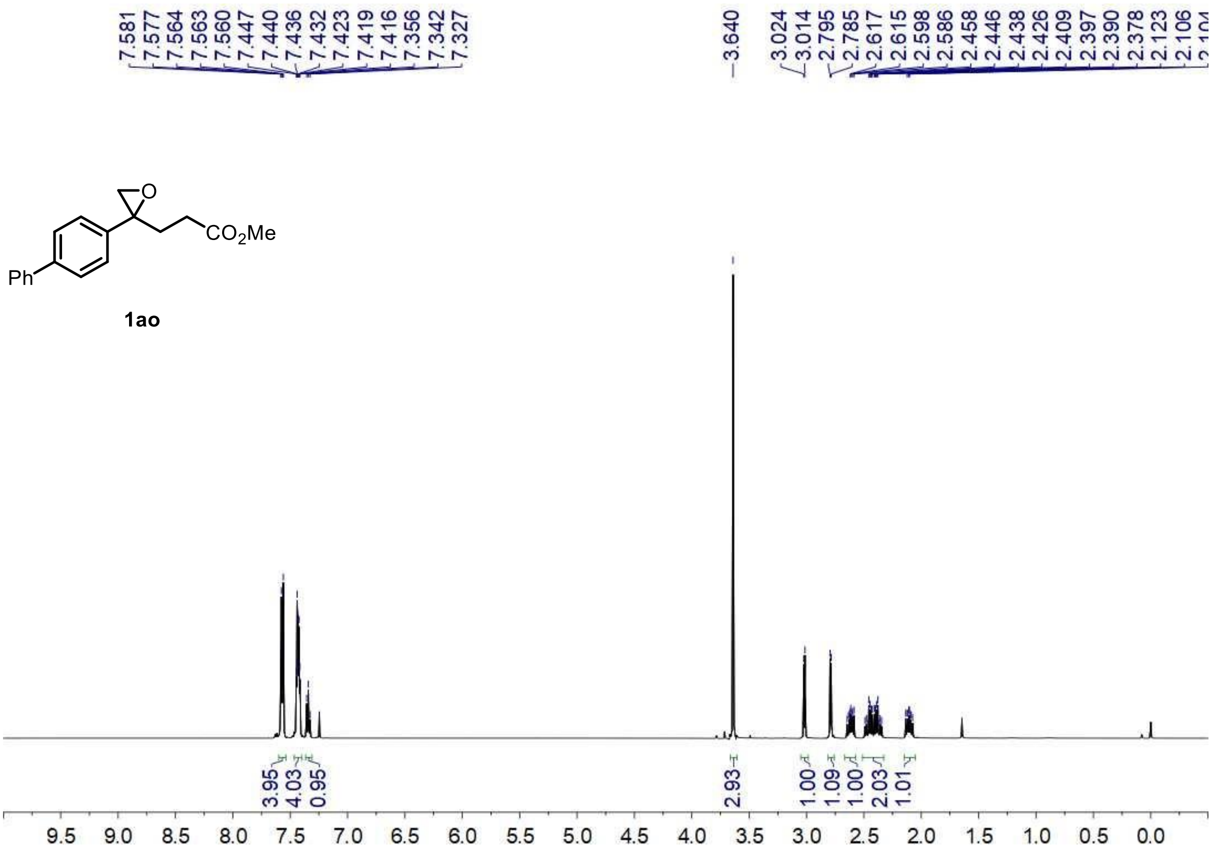

Supplementary Fig. 241 ^{1}**H NMR** (500 MHz, $CDCl_3$) spectrum of compound **1ao**.

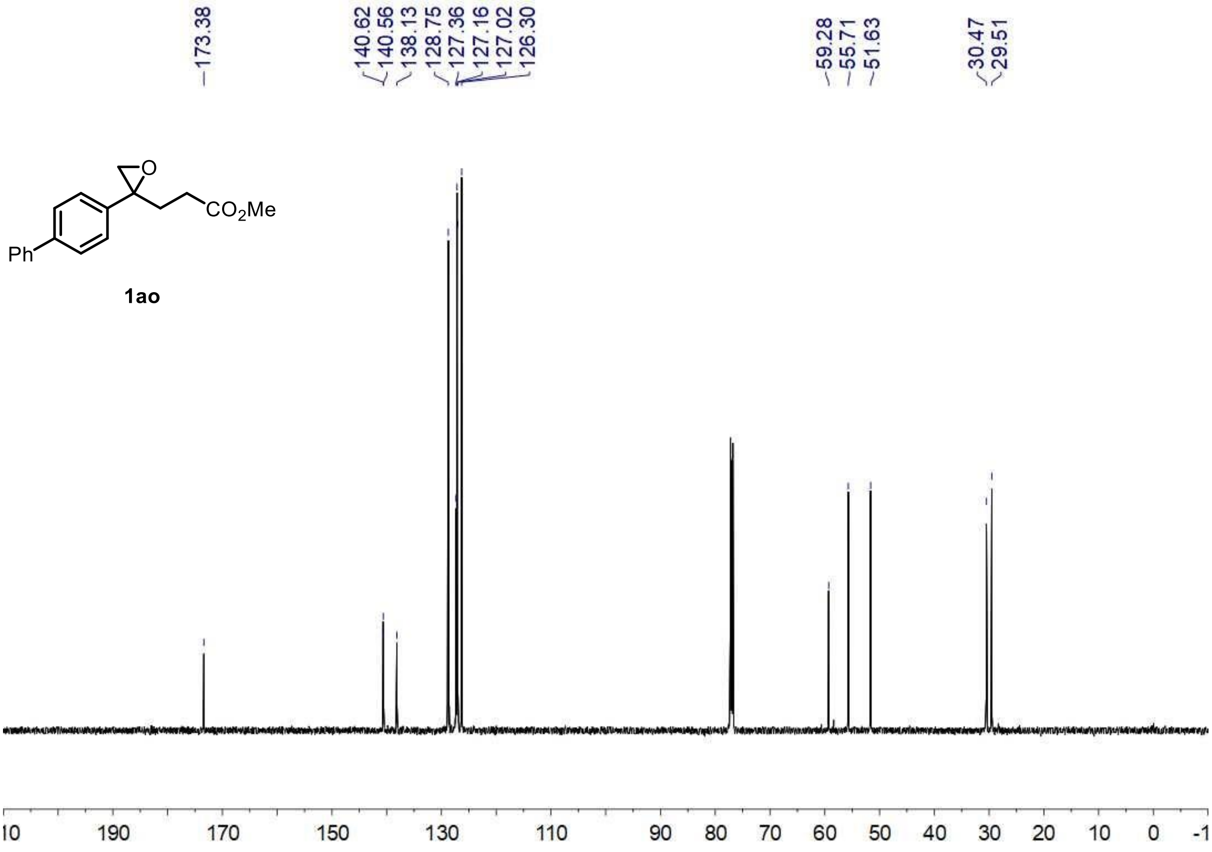

Supplementary Fig. 242 ^{13}**C NMR** (150 MHz, $CDCl_3$) spectrum of compound **1ao**.

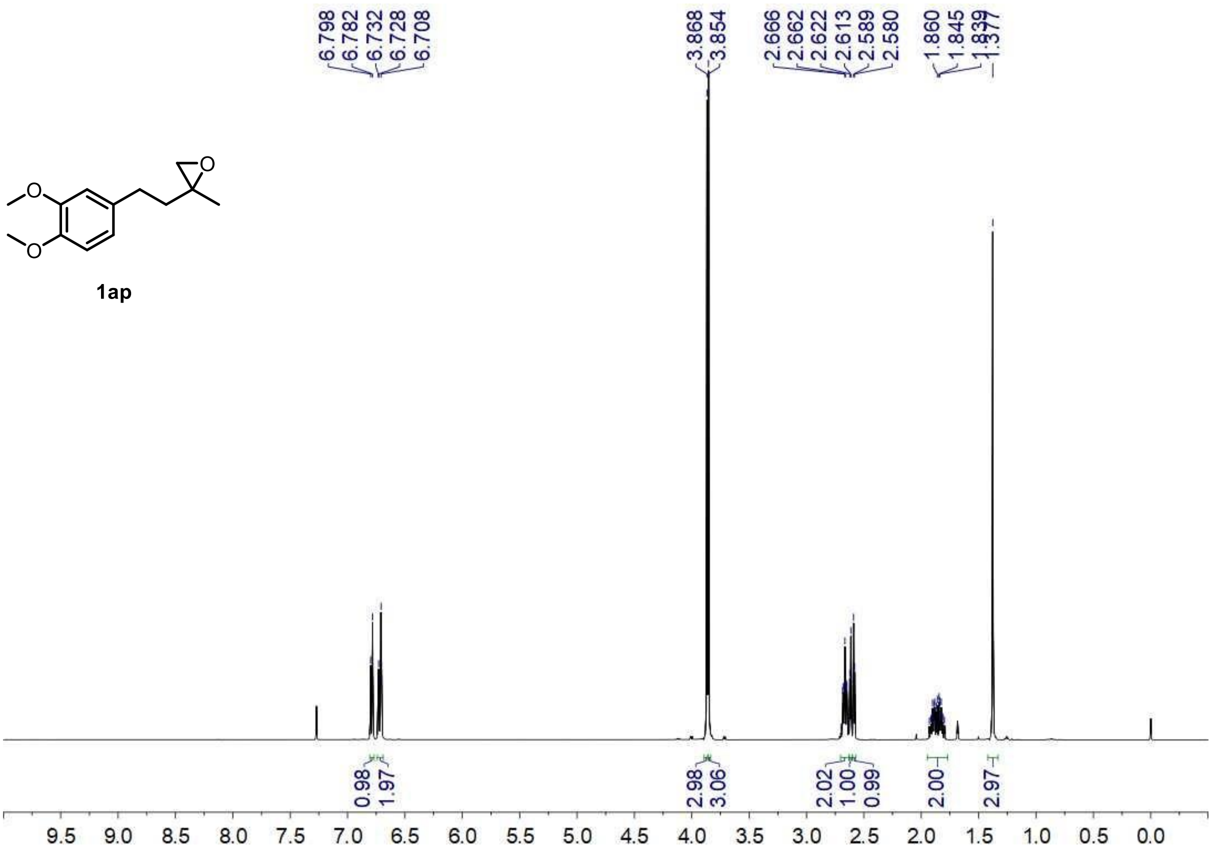

Supplementary Fig. 243 ^{1}H NMR (500 MHz, $CDCl_3$) spectrum of compound **1ap**.

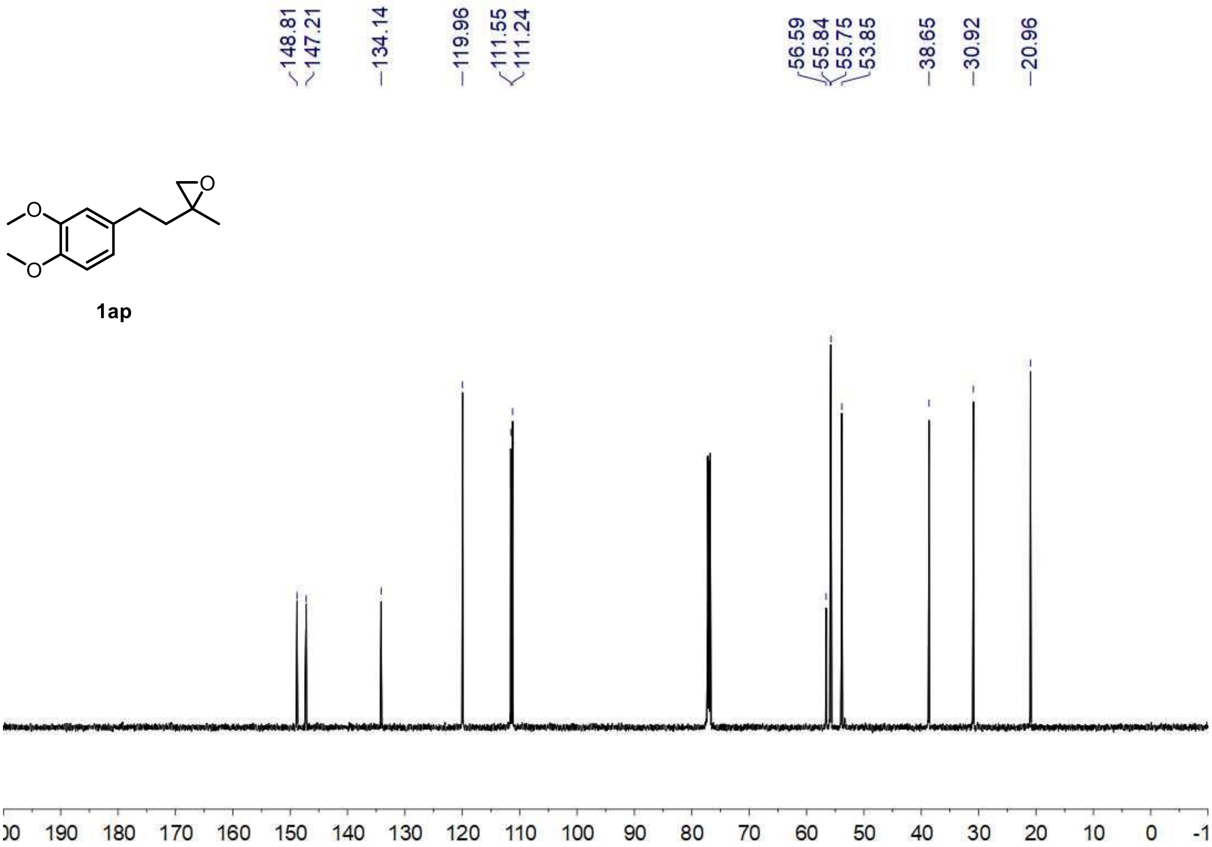

Supplementary Fig. 244 ^{13}C NMR (150 MHz, $CDCl_3$) spectrum of compound **1ap**.

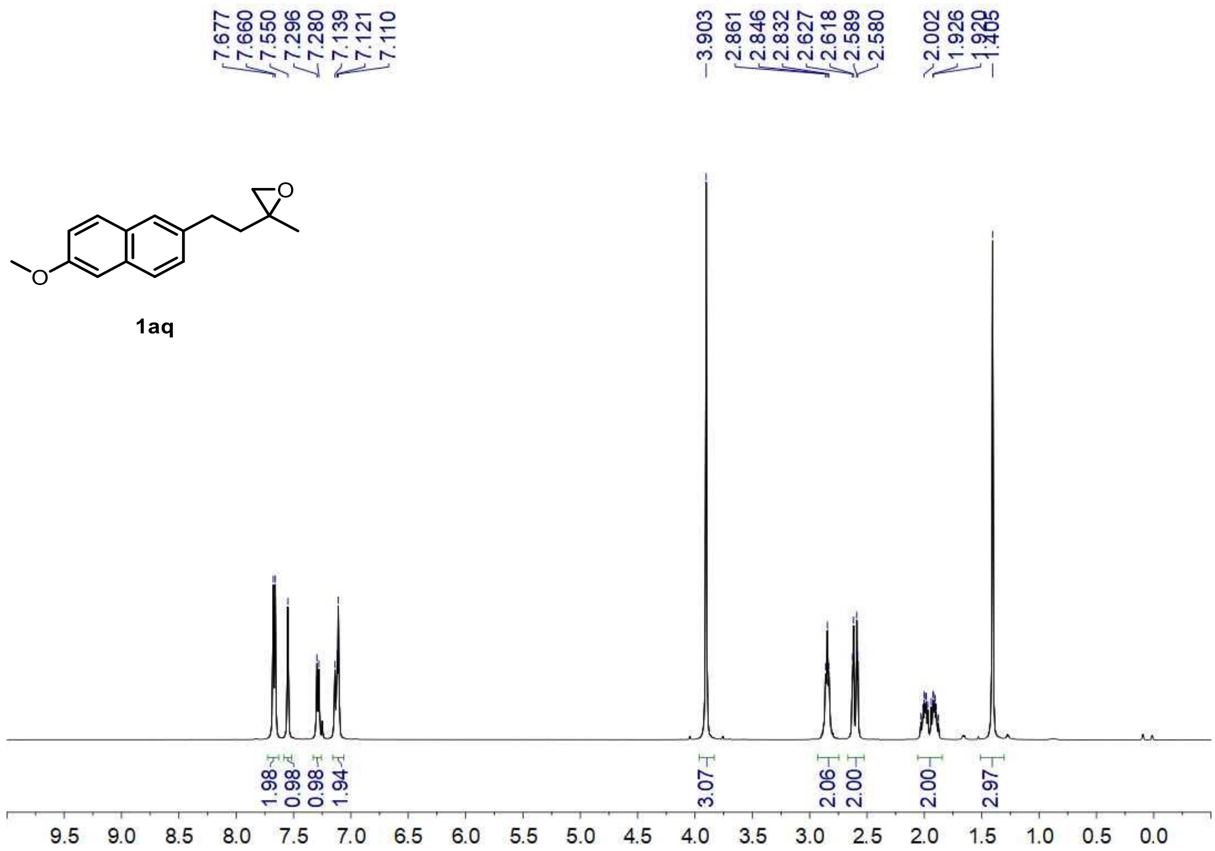

Supplementary Fig. 245 ^{1}H NMR (500 MHz, $CDCl_3$) spectrum of compound **1aq**.

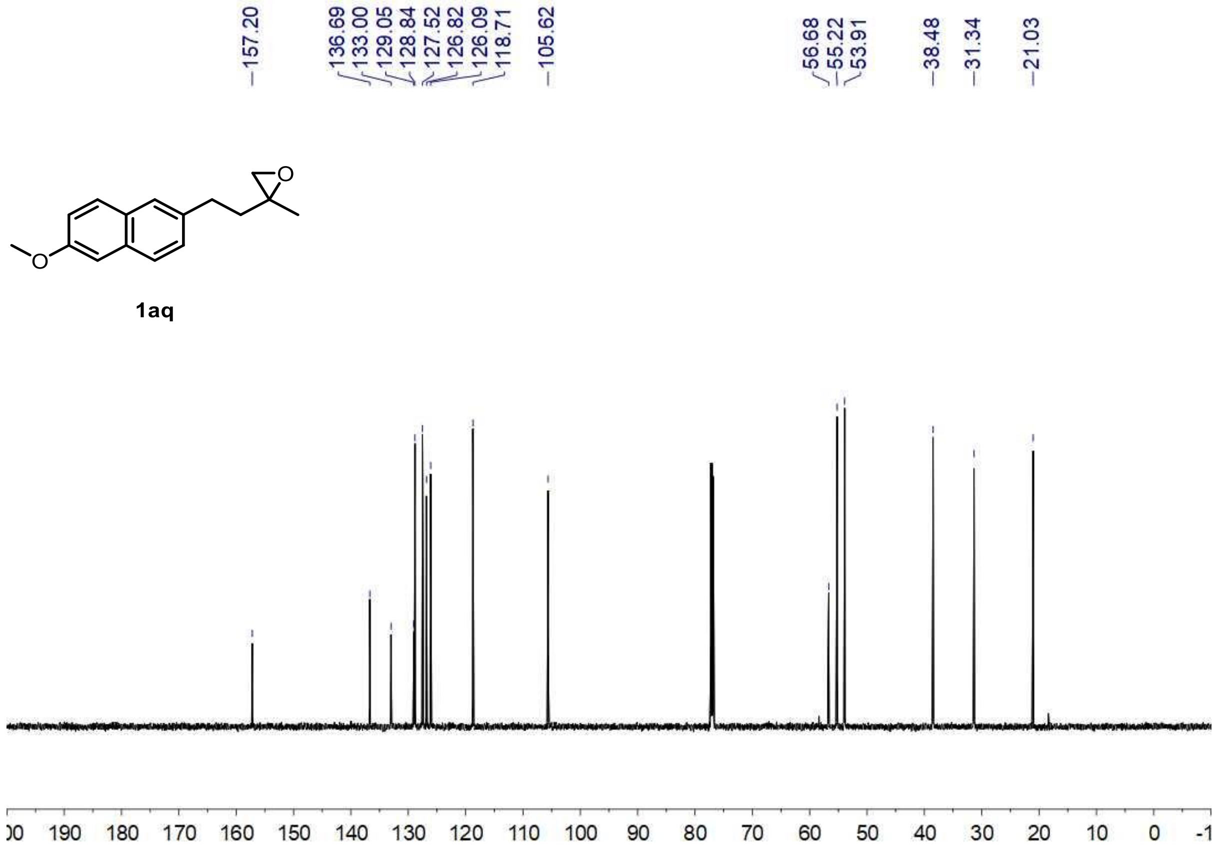

Supplementary Fig. 246 ^{13}C NMR (150 MHz, $CDCl_3$) spectrum of compound **1aq**.

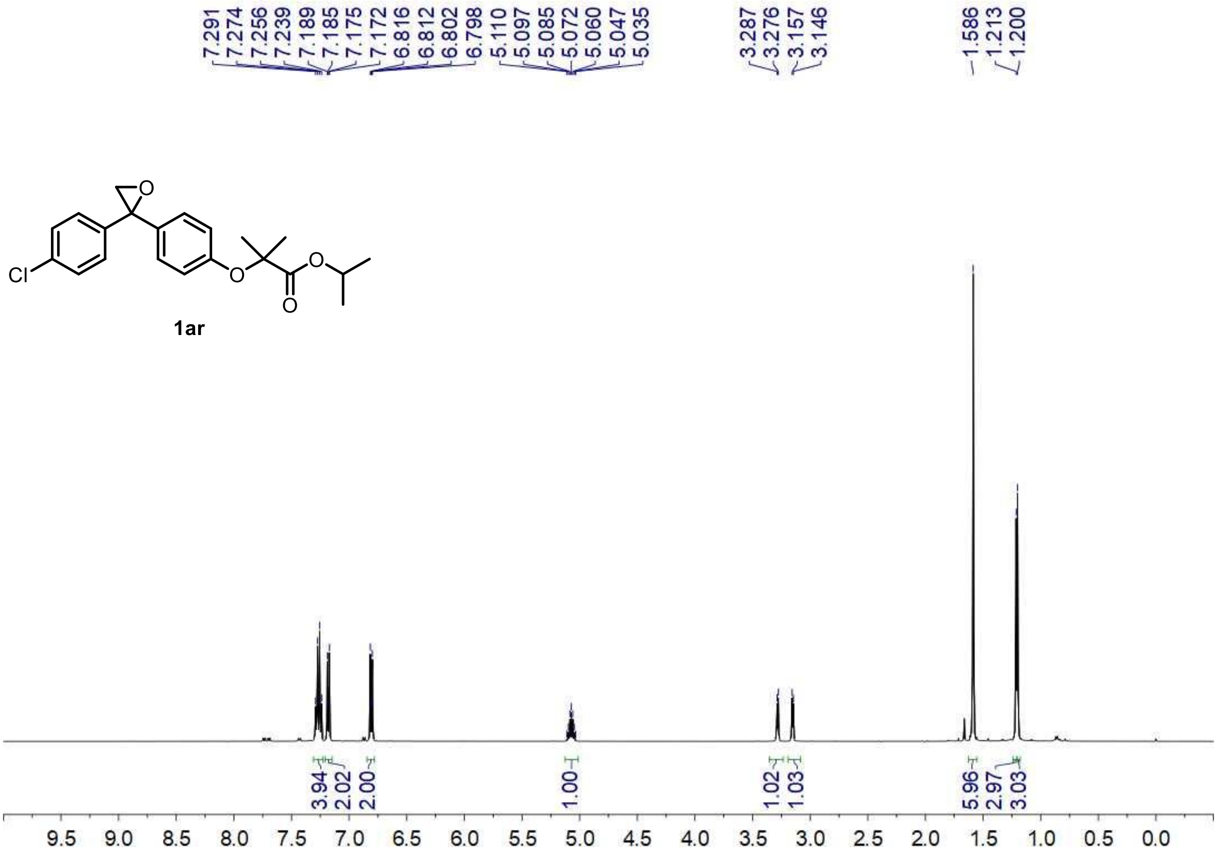

Supplementary Fig. 247 ^{1}H NMR (500 MHz, $CDCl_3$) spectrum of compound **1ar**.

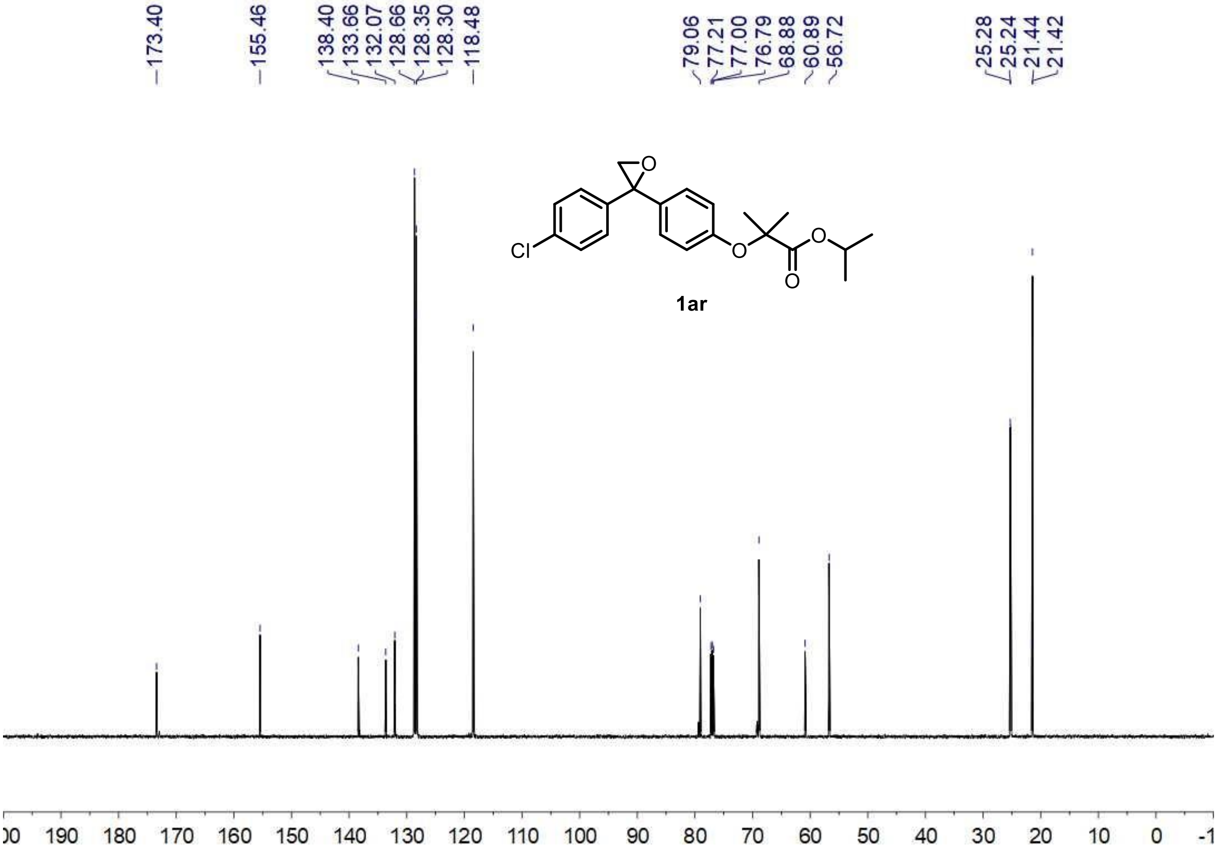

Supplementary Fig. 248 ^{13}C NMR (150 MHz, $CDCl_3$) spectrum of compound **1ar**.

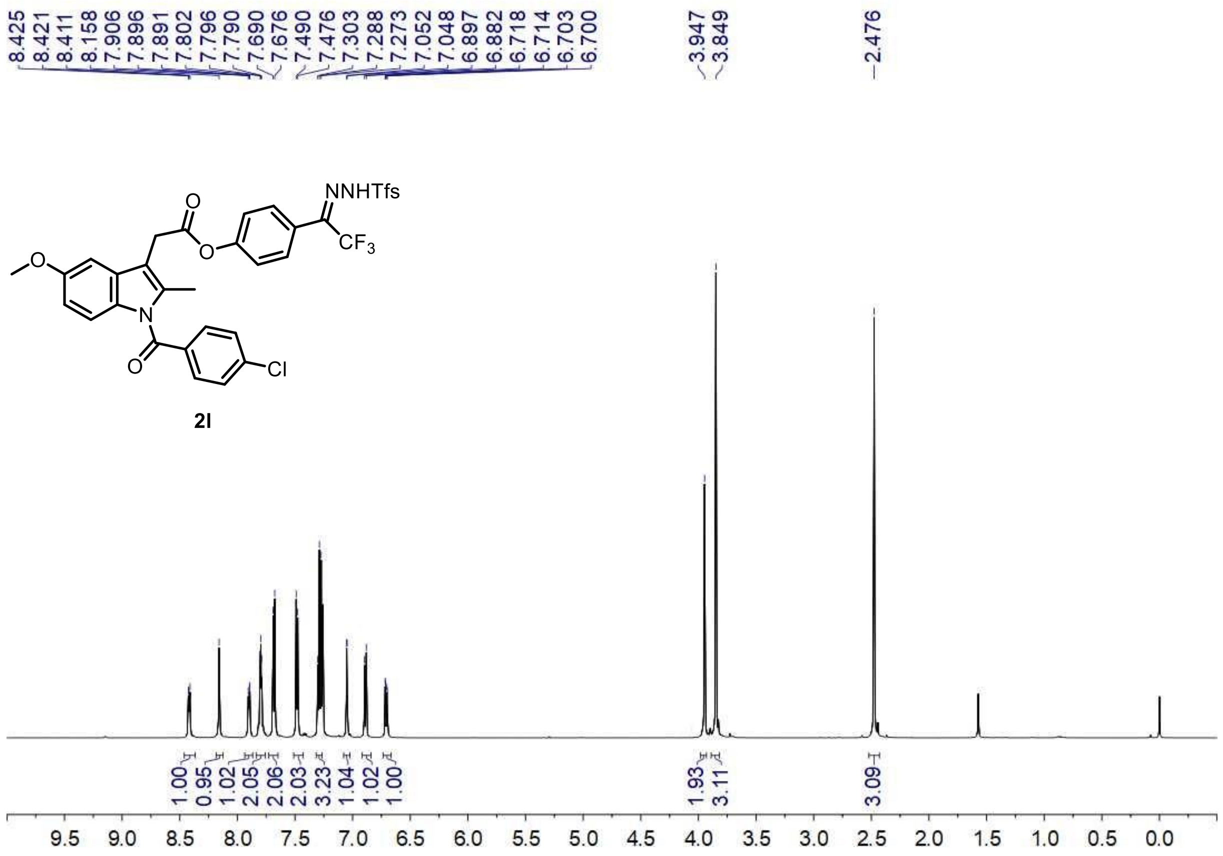

Supplementary Fig. 249 ^{1}H NMR (600 MHz, $CDCl_3$) spectrum of compound **2k**.

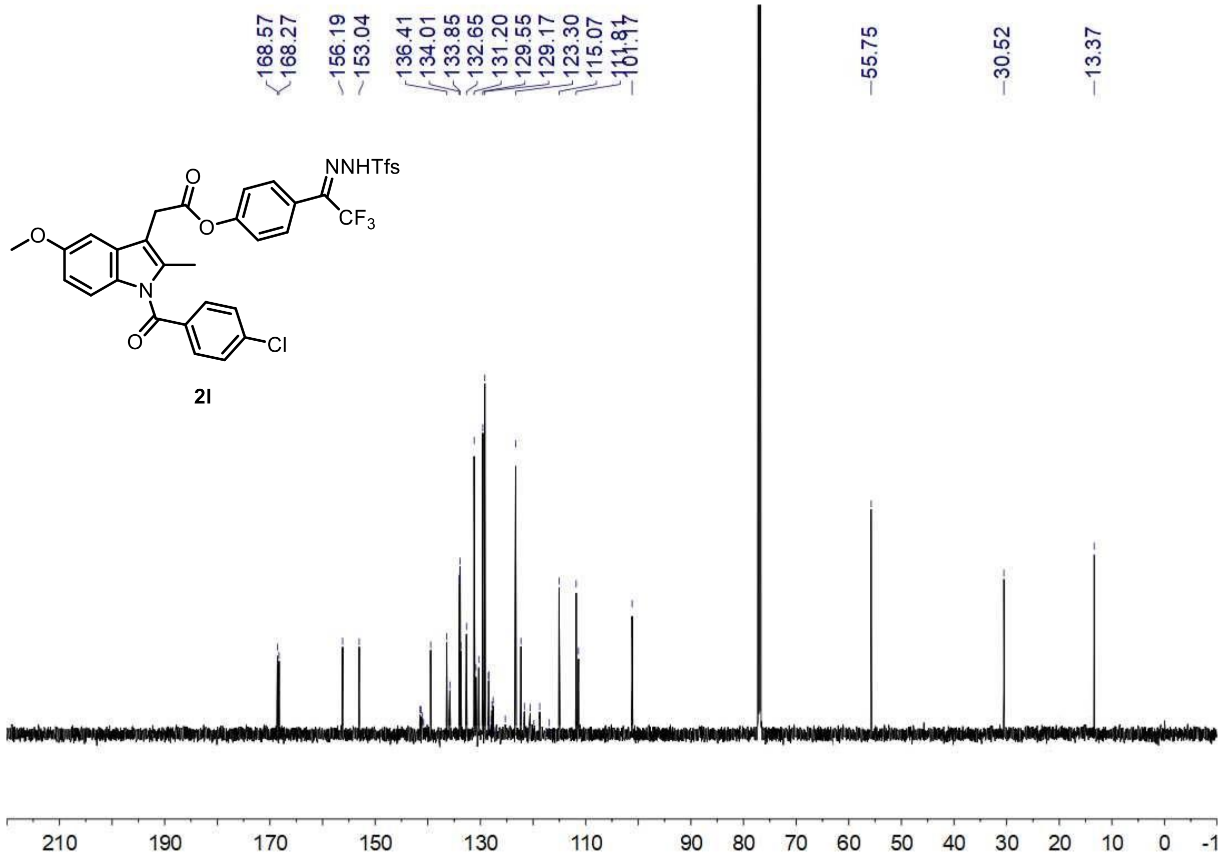

Supplementary Fig. 250 ^{13}C NMR (150 MHz, $CDCl_3$) spectrum of compound **2k**.

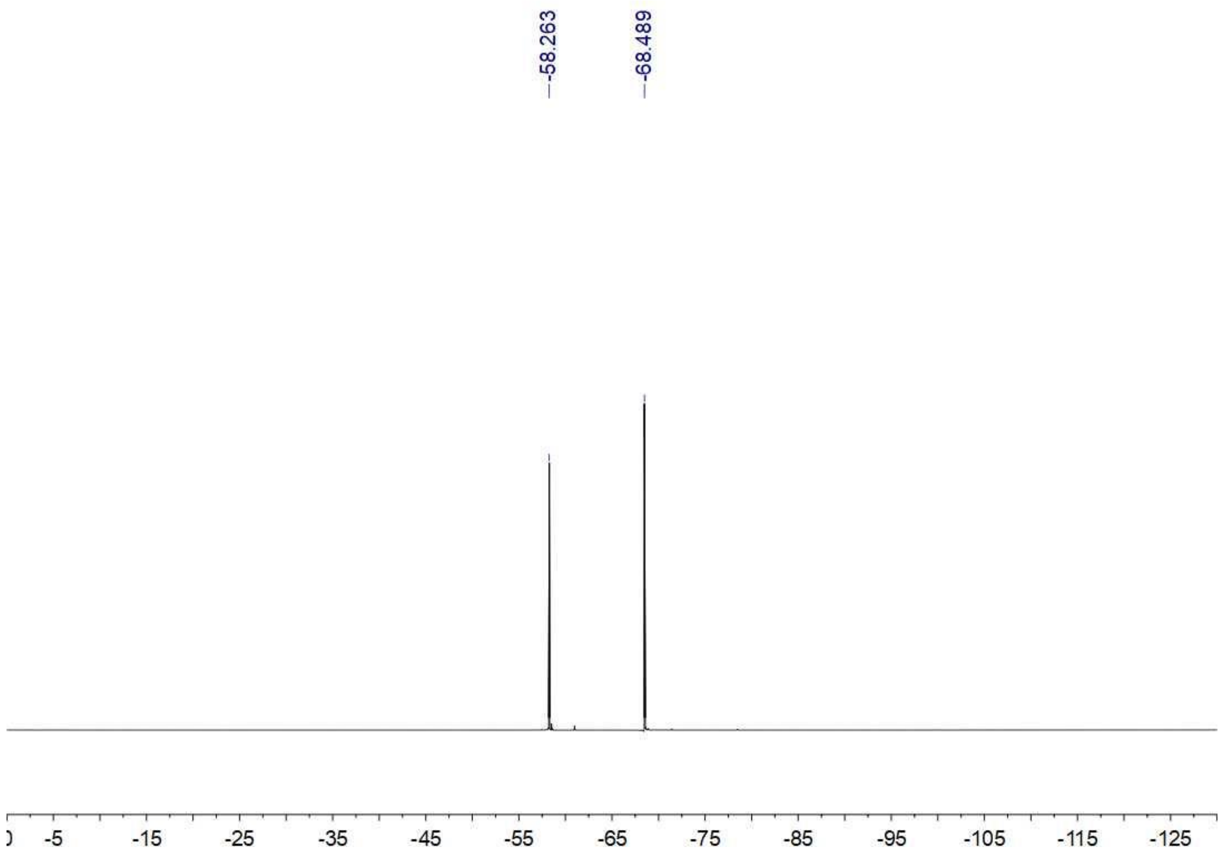

Supplementary Fig. 251 ^{19}F NMR (564 MHz, $CDCl_3$) spectrum of compound **2k**.

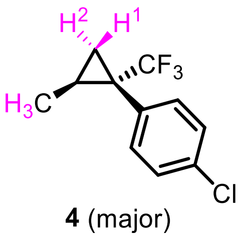

4 (major)

	ppm
H^1	0.78
H^2	1.48
H^{Me}	0.85

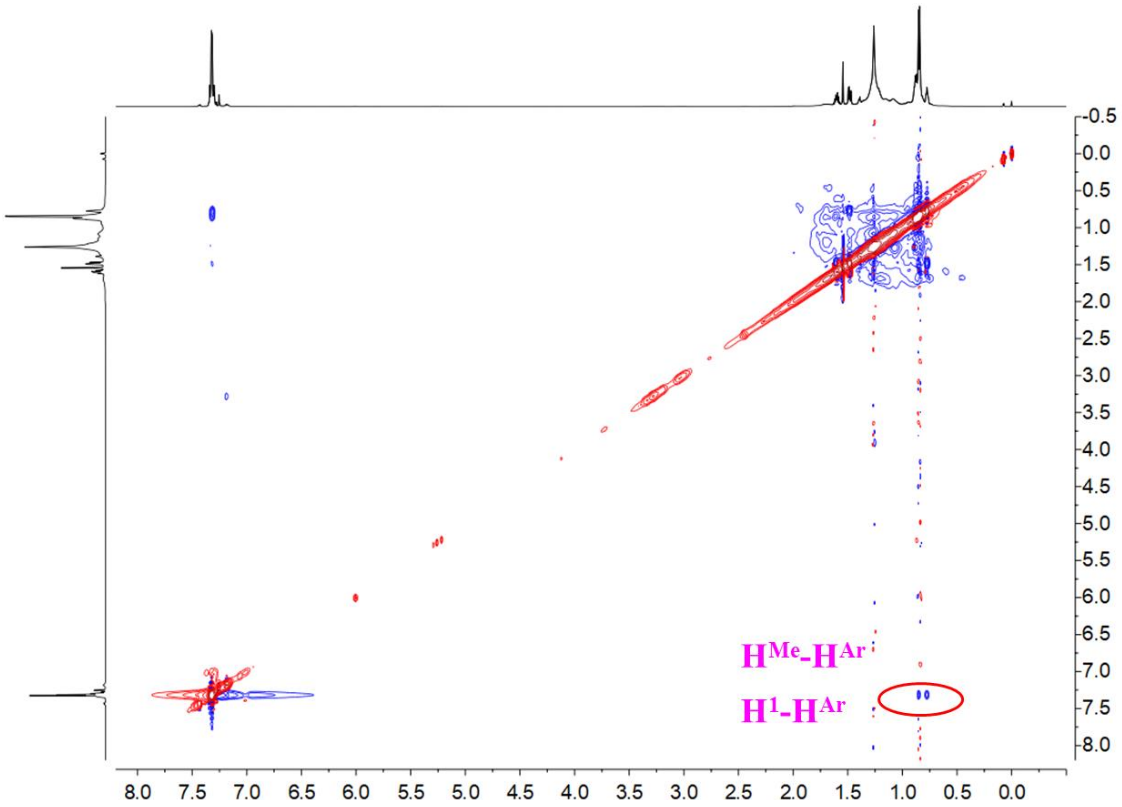

Supplementary Fig. 252 NOESY spectrum of compound **4**.

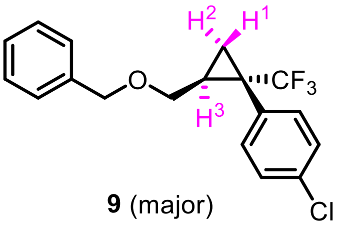

9 (major)

	ppm
H^1	1.02
H^2	1.53
H^3	1.95

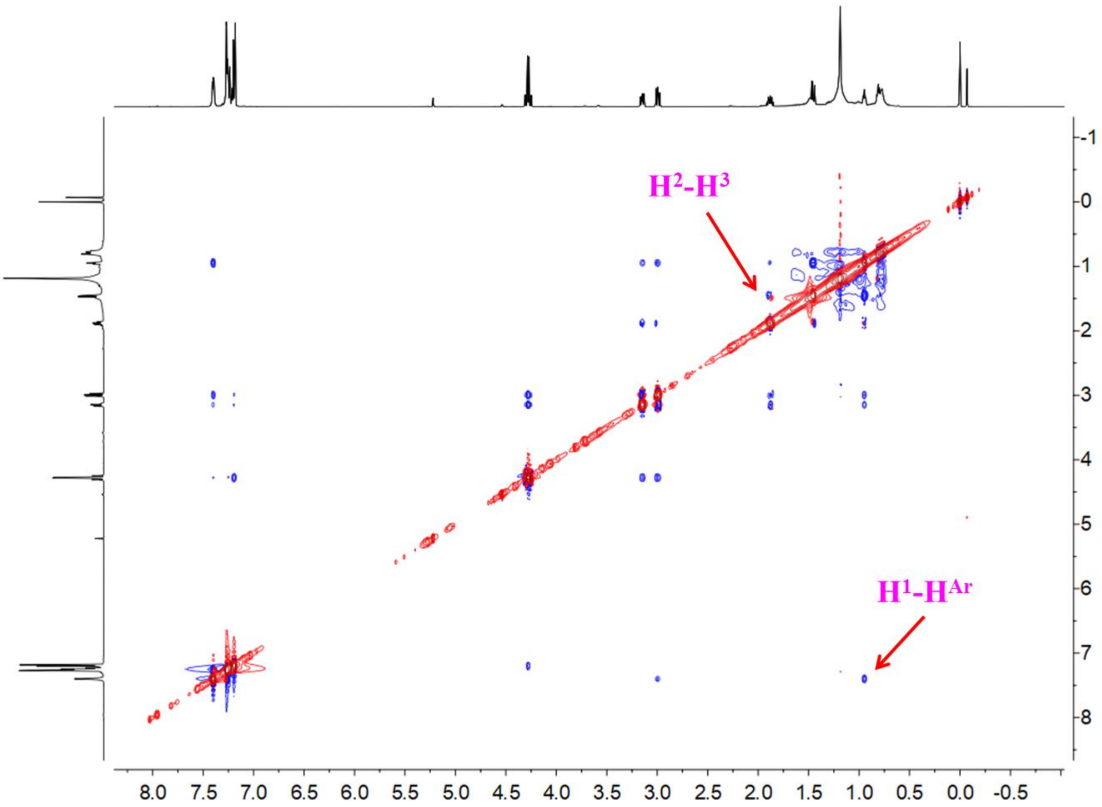

Supplementary Fig. 253 NOESY spectrum of compound **9**.

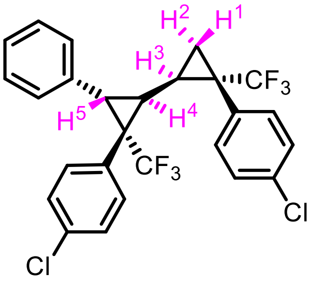

16 (major)

	ppm
H^1	1.00
H^2	1.28
H^3	1.90
H^4	1.83
H^5	3.00

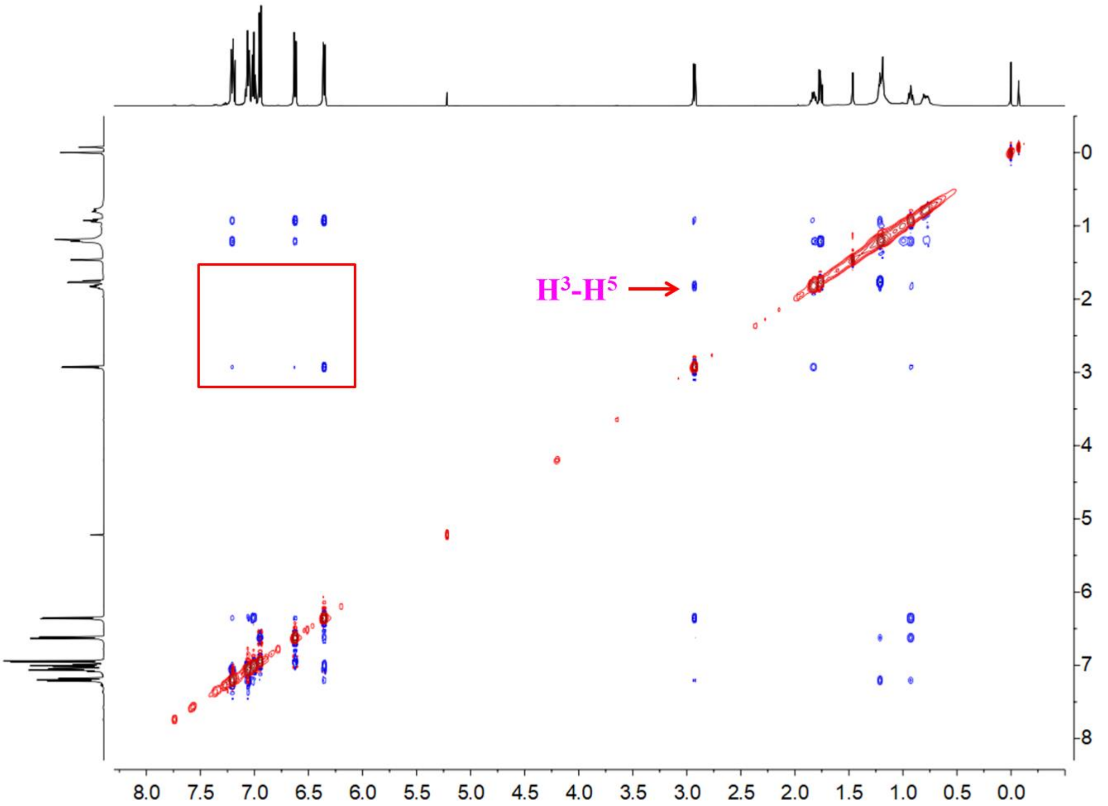

Supplementary Fig. 254 NOESY spectrum of compound **16**.

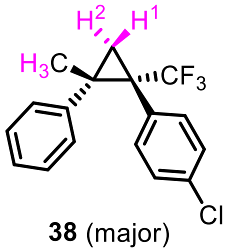

38 (major)

	ppm
H^1	1.71
H^2	2.10
H^{Me}	1.79

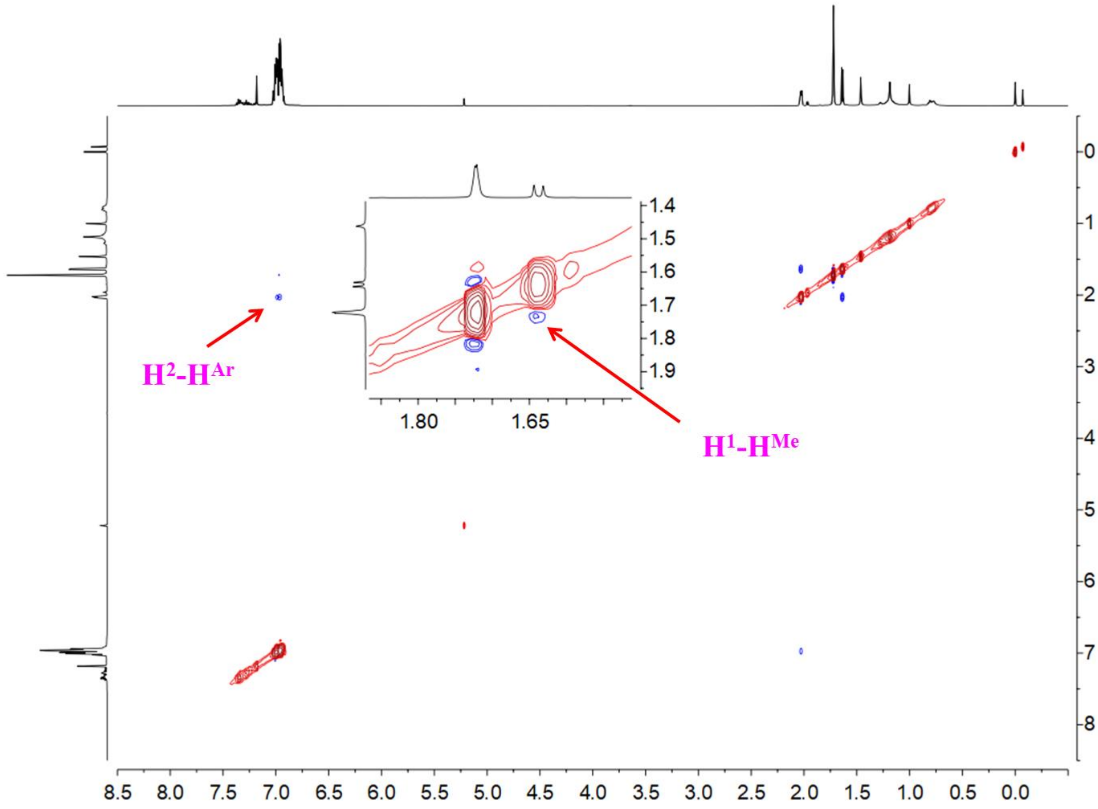

Supplementary Fig. 255 NOESY spectrum of compound **38**.

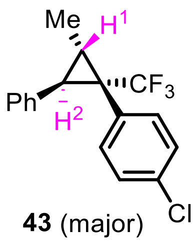

	ppm
H1	2.00
H2	2.69

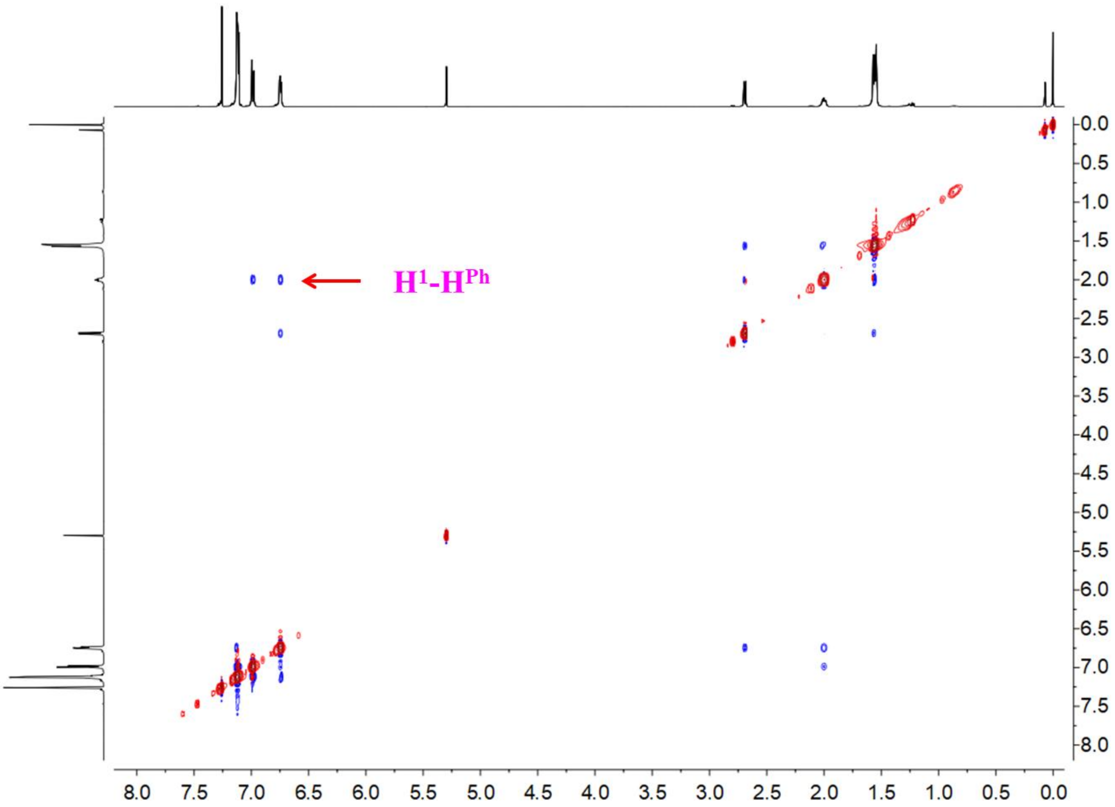

Supplementary Fig. 256 NOESY spectrum of compound **43**.

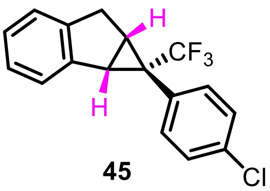

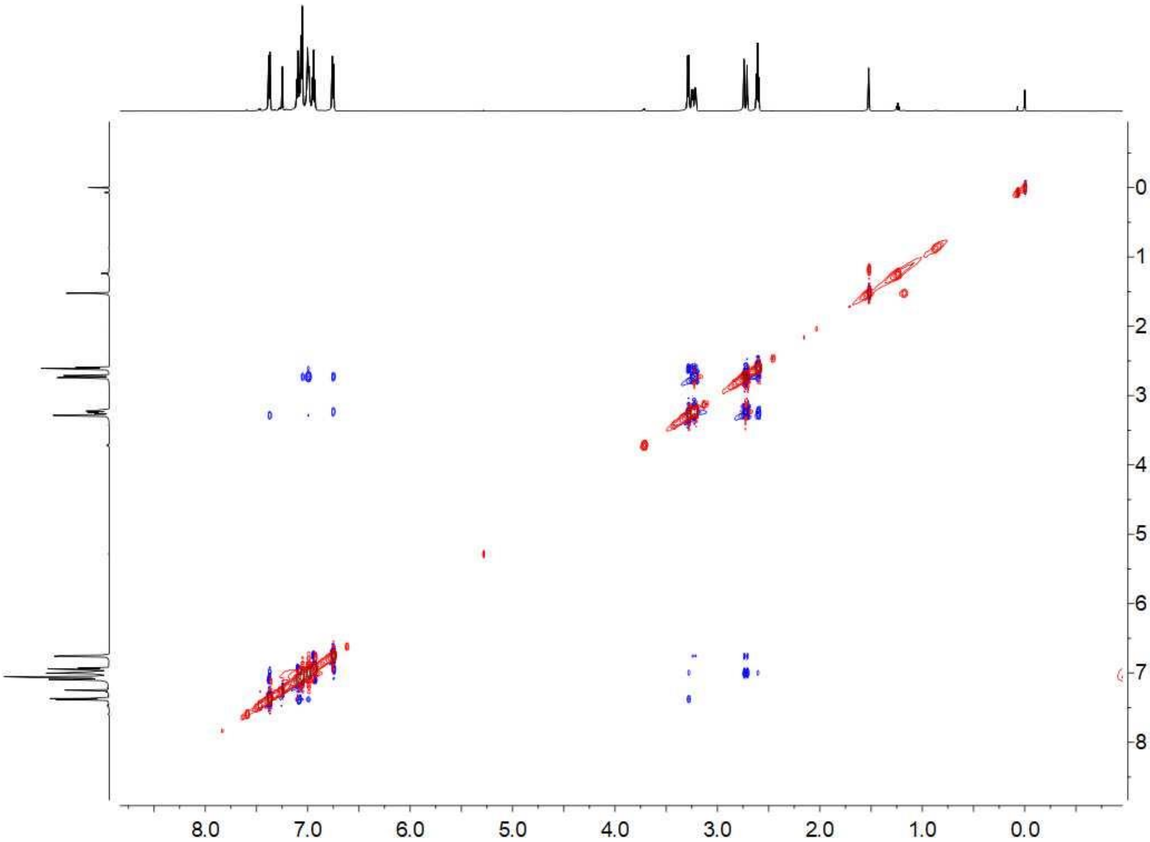

Supplementary Fig. 257 NOESY spectrum of compound **45**.

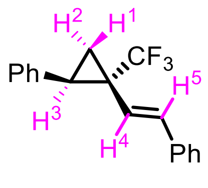

63 (major)

	ppm
H^1	1.58
H^2	1.71
H^3	2.82

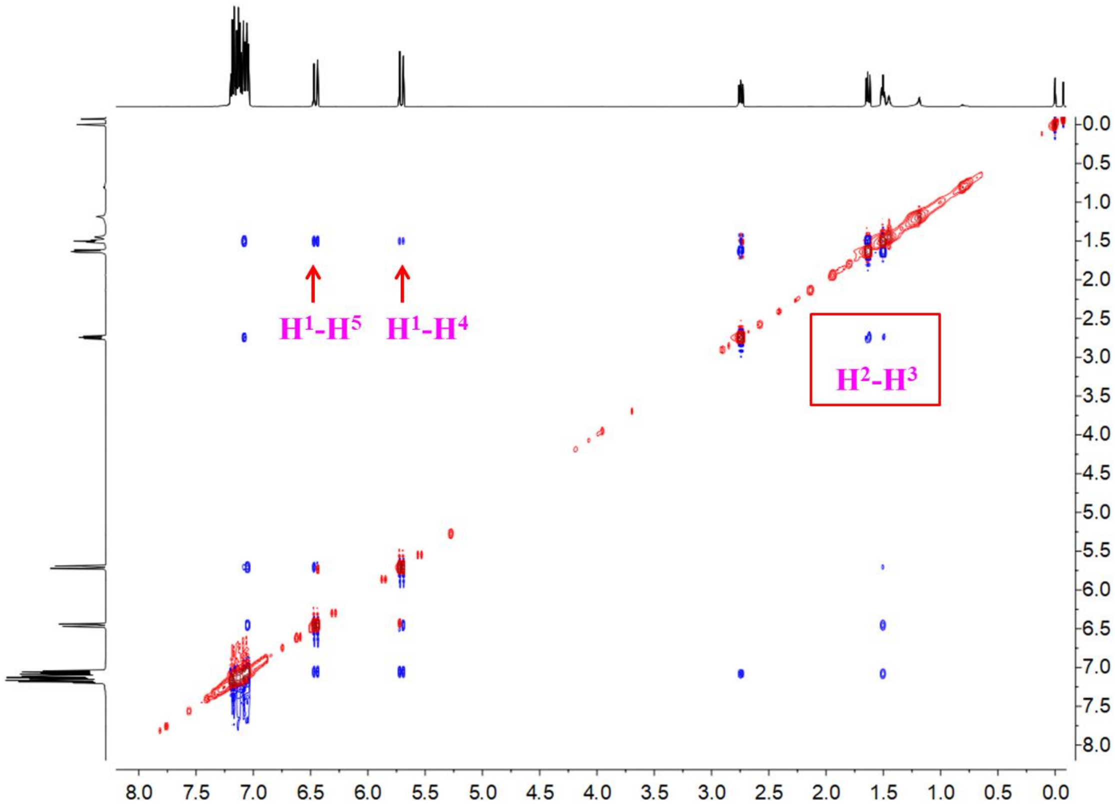

Supplementary Fig. 258 NOESY spectrum of compound **61**.

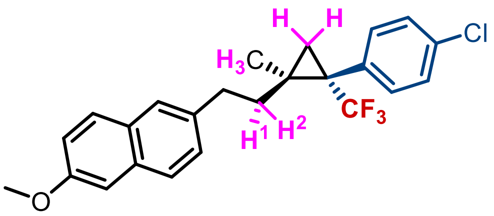

68-*anti* (major)

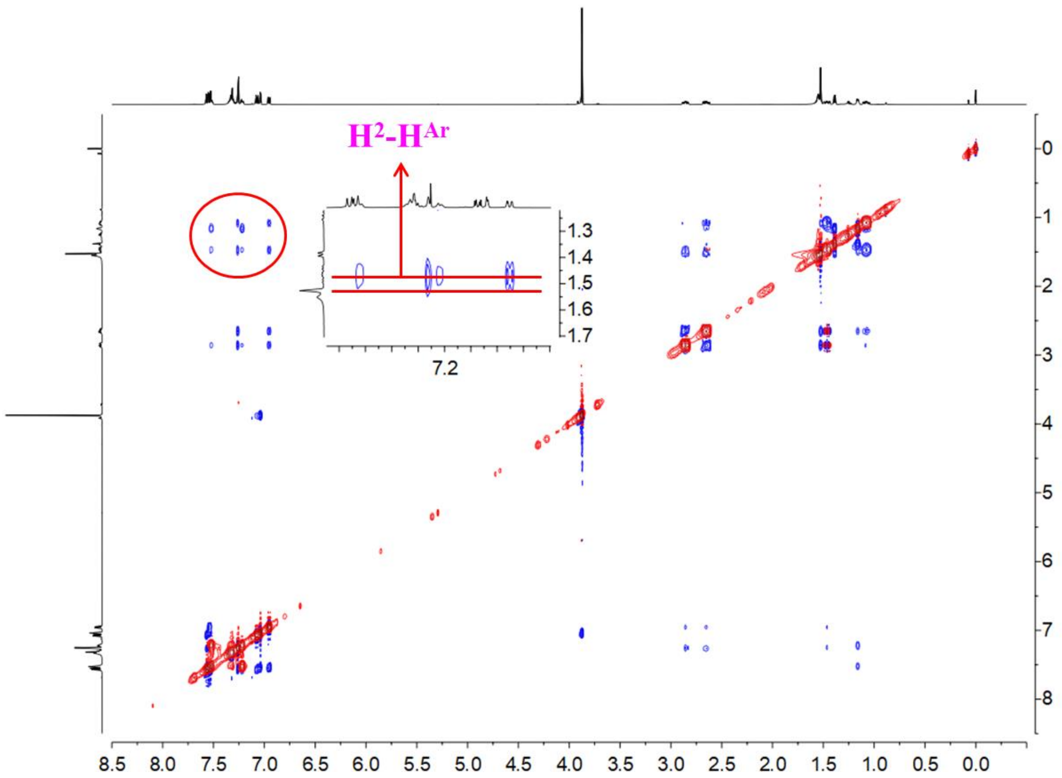

Supplementary Fig. 259 NOESY spectrum of compound **71**.

8 References

1. Vyas, D. J., Larionov, E., Besnard, C., Guenée, L. & Mazet, C. Isomerization of terminal epoxides by a [Pd−H] catalyst: a combined experimental and theoretical mechanistic Study. *J. Am. Chem. Soc.* **135**, 6177–6183 (2013).
2. Cabré, A. et al. Mild iridium-catalysed isomerization of epoxides. computational insights and application to the synthesis of β-alkyl amines. *Adv. Synth.Catal.* **361**, 3624–3631 (2019).
3. Qu, J. et al. Nickel-catalyzed cross-coupling of epoxides with aryltriflates: rapid and regioselective construction of aryl ketones. *Chem. Commun.,* **58**, 9214–9217 (2022).
4. Monaco, M. R. et al. The activation of carboxylic acids via self-assembly asymmetric organocatalysis: a combined experimental and computational investigation. *J. Am. Chem. Soc.* **138**, 14740–14749 (2016).
5. Aida, K. et al. Catalytic reductive ring opening of epoxides enabled by zirconocene and photoredox catalysis. *Chem* **8**, 1762–1774 (2022).
6. Fujiu, M. et al. Chemo-, regio-, and stereo-selective perfluoroalkylations by a Grignard complex with zirconocene. *Dalton Trans.,* **44**, 19464–19468 (2015).
7. Liu, Z. et al. Site-selective C–H benzylation of alkanes with *N*-triftosylhydrazones leading to alkyl aromatics. *Chem* **6**, 2110–2124 (2020).
8. Dias, H. V. R., Jin, W., Kim, H.-J. & Lu, H.-L. Polyfluorinated tris(pyrazolyl)borates. syntheses and spectroscopic and structural characterization of group 1 and group 11 metal complexes of $[HB(3,5\text{-}(CF_3)_2Pz)_3]^-$ and $[HB(3\text{-}(CF_3)Pz)_3]^-$. *Inorg. Chem.* **35**, 2317–2328 (1996).
9. Bruce, M. I. & Walsh, J. D. Chemistry of the group 1B elements. XIII. complexes of copper(I) or silver(I) containing only poly(pyrazolyl)borate Ligands. *Aust. J. Chem.,* **32**, 2753–2756 (1979).
10. Gaussian 16 Revision C.01, M. J. Frisch, G. W. Trucks, H. B. Schlegel, G. E. Scuseria, M. A. Robb, J. R. Cheeseman, G. Scalmani, V. Barone, G. A. Petersson, H. Nakatsuji, X. Li, M. Caricato, A. V. Marenich, J. Bloino, B. G. Janesko, R. Gomperts, B. Mennucci, H. P. Hratchian, J. V. Ortiz, A. F. Izmaylov, J. L. Sonnenberg, D. Williams-Young, F. Ding, F. Lipparini, F. Egidi, J. Goings, B. Peng, A. Petrone, T. Henderson, D. Ranasinghe, V. G. Zakrzewski, J. Gao, N. Rega, G. Zheng, W. Liang, M. Hada, M. Ehara, K. Toyota, R. Fukuda, J. Hasegawa, M. Ishida, T. Nakajima, Y. Honda, O. Kitao, H. Nakai, T. Vreven, K. Throssell, J. A. Montgomery, Jr., J. E. Peralta, F. Ogliaro, M. J. Bearpark, J. J. Heyd, E. N. Brothers, K. N. Kudin, V. N. Staroverov, T. A. Keith, R. Kobayashi, J. Normand, K. Raghavachari, A. P. Rendell, J. C. Burant, S. S. Iyengar, J. Tomasi, M. Cossi, J. M. Millam, M. Klene, C. Adamo, R. Cammi, J. W. Ochterski, R. L. Martin, K. Morokuma, O. Farkas, J. B. Foresman, and D. J. Fox, Gaussian, Inc., Wallingford CT, (2019**)**.
11. Becke, A. D. *J. Chem. Phys*., **98**, 5648 (1993).
12. Perdew, J. P. & Wang, Y. *Phys. Rev. B*, **45**, 13244 (1992).
13. Grimme, S., Ehrlich S. & Goerigk, L. *J. Comp. Chem.,* **32**, 1456 (2011).
14. Tomasi, J., Mennucci, B. & Cammi, R. Quantum mechanical continuum solvation models. *Chem. Rev.* **105**, 2999–3093 (2005).
15. Küchle, W., Dolg, M., Stoll, H. & Preuss, H. *J. Chem. Phys*., **100**, 7535 (1994).
16. Cao, X., Dolg, M. & Stoll, H. *J. Chem. Phys*., **118**, 487 (2003).
17. Andzelm, J. & Huzinaga, S. Gaussian Basis Sets for Molecular Calculations, Elsevier Science, New York, (1984).
18. Hehre, W. J., Ditchfield, R. & Pople, J. A. *J. Chem. Phys.* **56**, 2257 (1972).

19. Dill, J. D. & Pople, J. A. *J. Chem. Phys.* **62**, 2921 (1975).
20. Gonzalez, C. & Schlegel, H. B. *J. Chem. Phys*., **90**, 2154 (1989).
21. Gonzalez, C. & Schlegel, H. B. *J. Chem. Phys*., **94**, 5523 (1990).
22. Legault, C. Y. CYLview, 1.0b; Université de Sherbrooke: Canada, Available at (2009).
23. a) Denton, J. R., Sukumaran, D. & Davies, H. M. Enantioselective synthesis of trifluoromethyl-substituted cyclopropanes. *Org. Lett.* **14**, 2625 (2007). b) Zhang, X. et al. Fluoroalkyl *N*-triftosylhydrazones as easily decomposable diazo surrogates for asymmetric [2+1] cycloaddition: synthesis of chiral fluoroalkyl cyclopropenes and cyclopropanes. *ACS Catal.* **11**, 8527−8537 (2021).
24. a) Verdecchia, M., Tubaro, C. & Biffis, A. Olefin cyclopropanation with aryl diazocompounds upon catalysis by a dirhodium(II) complex. *Tetrahedron Letters,* **52**, 1136−1139 (2011). b) Levesque, É., Goudreau, S. R. & Charette, A. B. Improved zinc-catalyzed Simmons-Smith Reaction: access to various 1,2,3-trisubstituted cyclopropanes. *Org. Lett.,* **16**, 1490−1493 (2014).
25. Yan, K. et al. Blue light-promoted cyclopropenizations of *N*-tosylhydrazones in water. *Chinese Chemical Letters,* **32**, 3984−3987 (2021).
