# Supplementary material for: Silver-catalyzed direct conversion of epoxides into cyclopropanes using N-triftosylhydrazones
Source: Nat Commun. 2024 Mar 2;15:1951. doi: 10.1038/s41467-024-46188-w (PMC10908805; doi:10.1038/s41467-024-46188-w)
Supplement: Supplementary file 3 — Description of Additional Supplementary Files [file 41467_2024_46188_MOESM3_ESM.pdf]

Description of Additional Supplementary Files

Supplementary Data Legend

Supplementary Data 1: Cartesian coordinates of all optimized structures
